# Genome-wide analysis of NBS-encoding disease resistance genes in *Cucumis sativus* and phylogenetic study of NBS-encoding genes in Cucurbitaceae crops

**DOI:** 10.1186/1471-2164-14-109

**Published:** 2013-02-19

**Authors:** Hongjian Wan, Wei Yuan, Kailiang Bo, Jia Shen, Xin Pang, Jinfeng Chen

**Affiliations:** 1 State Key Laboratory of Crop Genetics and Germplasm Enhancement, College of Horticulture, Nanjing Agricultural University, Nanjing 210095, People’s Republic of China; 2 Institute of Vegetables, Zhejiang Academy of Agricultural Sciences, Hangzhou, 310021, People’s Republic of China

**Keywords:** NBS-LRR, Cucumber, Cucurbitaceae, Phylogenetic relationship

## Abstract

**Background:**

Plant nucleotide-binding site (NBS)-leucine-rich repeat (LRR) proteins encoded by resistance genes play an important role in the responses of plants to various pathogens, including viruses, bacteria, fungi, and nematodes. In this study, a comprehensive analysis of NBS-encoding genes within the whole cucumber genome was performed, and the phylogenetic relationships of NBS-encoding resistance gene homologues (RGHs) belonging to six species in five genera of Cucurbitaceae crops were compared.

**Results:**

Cucumber has relatively few NBS-encoding genes. Nevertheless, cucumber maintains genes belonging to both Toll/interleukine-1 receptor (TIR) and CC (coiled-coil) families. Eight commonly conserved motifs have been established in these two families which support the grouping into TIR and CC families. Moreover, three additional conserved motifs, namely, CNBS-1, CNBS-2 and TNBS-1, have been identified in sequences from CC and TIR families. Analyses of exon/intron configurations revealed that some intron loss or gain events occurred during the structural evolution between the two families. Phylogenetic analyses revealed that gene duplication, sequence divergence, and gene loss were proposed as the major modes of evolution of NBS-encoding genes in Cucurbitaceae species. Compared with NBS-encoding sequences from the *Arabidopsis thaliana* genome, the remaining seven TIR familes of NBS proteins and RGHs from Cucurbitaceae species have been shown to be phylogenetically distinct from the TIR family of NBS-encoding genes in *Arabidopsis*, except for two subfamilies (TIR4 and TIR9). On the other hand, in the CC-NBS family, they grouped closely with the CC family of NBS-encoding genes in *Arabidopsis*. Thus, the NBS-encoding genes in Cucurbitaceae crops are shown to be ancient, and NBS-encoding gene expansions (especially the TIR family) may have occurred before the divergence of Cucurbitaceae and *Arabidopsis*.

**Conclusion:**

The results of this paper will provide a genomic framework for the further isolation of candidate disease resistance NBS-encoding genes in cucumber, and contribute to the understanding of the evolutionary mode of NBS-encoding genes in Cucurbitaceae crops.

## Background

Cucumbers (*Cucumis sativus* L.) of the Cucurbitaceae plant family are among the most important vegetable crops in the world. However, susceptibility to multiple pathogens hinders their production increase and quality improvement [1-5] The NBS-LRR resistance (R) genes, which encode proteins containing nucleotide binding sites (NBS) and leucine-rich repeat (LRR) domains, form the largest R-gene family among plant genomes [6]. Therefore, a systematic evaluation of NBS-encoding genes is required in order to better understand cucumber resistance and susceptibility. Previously, NBS and *Pto* analogues had been isolated and characterized using degenerate primers in cucumbers [7-9]. However, this experimental approach failed to detect all the members of the gene families in the cucumbers. Fortunately, the cucumber genome was sequenced by researchers who worked on the ‘Chinese Long’ inbred line 9930 and the gynoecious inbred line ‘Gy14’, which provided an opportunity to conduct a comprehensive overview of the NBS-encoding gene superfamily at the genome level [10]. Recently, Kang et al. localized the cucumber scab R gene *Ccu* into an R-gene cluster located in a 670 kb region of cucumber chromosome 2 [11]. Four resistance gene homologues (RGHs) were located in the region delimited by the molecular markers Indel 01 and Indel 02, and thus were possible *Ccu* candidates [11]. Genome-wide analysis of cucumber NBS-encoding genes played an important role in R-gene mapping and cloning.

Aside from cucumber, the Cucurbitaceae plant family also includes many important vegetable crops such as the bottle gourd *Lagenaria siceraria* (Mol.) Standl.], luffa *Luffa cylindrica* (L.) Roem.], squash (*Cucurbita moschata* Duch.), melon (*Cucumis melo*. L), and watermelon *Citrullus lanatus* (Thunb) Mansfeld]. For these species in the Cucurbitaceae family, several studies concerning their phylogenetic relationships have been reported [12-16]. These studies have shown that a wide genetic distance exists between the *Citrullus* and *Cucumis* groups. Phylogenetic relationships among *Citrullus* species and subspecies are closer in comparison with those among most *Cucumis* species [14]. Moreover, Lagenaria et al. [16] reported the history of Cucurbitaceae using a multigene phylogeny for 114 of the 115 genera and 25% of the 960 species worldwide, and found that *Cucumis* and *Cucurbita* are more closely related to each other than any of them are to *Luffa*. However, few R-related genetic and genomic resources are available for the improvement of these crops. Therefore, the analysis of R-genes or RGHs will contribute to their timely application in disease resistance breeding in Cucurbitaceae crops.

Currently, numerous disease R-genes which confer resistance to a wide range of pathogens, including viruses, bacteria, fungi, nematodes and aphids, have been cloned through map-based cloning and transposon-tagging from many dicotyledonous and monocotyledonous plants [17-19]. Although the mechanisms of infection of these organisms differ significantly, R-gene products are remarkably similar to one another. Most R-genes seem to encode putative NBS-LRR domains. It is well known that NBS-LRR R-proteins in plants recognize the presence of the pathogen through two different types of perception mechanisms [17,20,21]. One is direct recognition between R-proteins in plants and avirulence proteins in the pathogen, and the other is the indirect perception mechanism postulated by the Guard Model [17,22]. The Guard Model proposes that NBS-LRR R-proteins act by monitoring the plant effector target against pathogen effector proteins, and explains that a single R-protein is able to perceive multiple effectors. Therefore, it is theorized that few R-genes are capable of targeting the broad diversity of pathogens in plants [17]. Genome-wide analysis of a complete set of NBS-LRR R-proteins in the plant genome will provide new insights into the genetic diversity of the R-genes available in this species.

To date, NBS-LRR R-genes may be divided into two families, distinguished by the presence or absence of a TIR domain at the N-terminal [23]. The first is known as the TIR NBS-LRR family, which is homologous to the Toll protein and interleukin-1 receptor at the N-terminal domain, and the other is known as the non-TIR NBS-LRR family. Generally, non-TIR NBS-LRR R-proteins include a putative coiled-coil domain at the N-terminal domain. Thus, they are also referred to as CC NBS-LRR R-proteins. Eight conserved motifs have been identified in the NBS domains of these two R-gene families [23], some of which are specific to the non-TIR and the TIR NBS-LRR families [24]. Degenerate primers have been designed based on these conserved motifs, and a large number of NBS-encoding RGHs have been isolated from different plant species via polymerase chain reaction (PCR) [25-30]. These RGHs have high sequence similarity with R-proteins cloned from different plant species.

In this paper, an *in silico* search of cucumber genome databases was conducted to identify members of the cucumber NBS-encoding gene family. A total of 57 members were identified from the phytozome database (http://www.phytozome.net/). A phylogenetic tree was constructed and the NBS-encoding genes were separated into two distinct groups, namely the TIR and CC families. Conserved motifs were analyzed in these two families to support the partition. In addition, 158 NBS-encoding RGHs from the other five Cucurbitaceae crop genomes were also identified via degenerate PCR amplification and database mining. These genes, together with the RGHs, were used for the study of their phylogenetic relationship in Cucurbitaceae crops. Finally, a comparative analysis between the NBS-encoding genes from *Arabidopsis thaliana* and those from the Cucurbitaceae crops was performed in order to determine their evolutionary origin. The findings will provide a strong groundwork for the isolation of candidate R-genes in cucumber and contribute to understanding the evolution of NBS-encoding genes in Cucurbitaceae crops.

## Results

### Sequence and database search for NBS-encoding genes in *Cucumis sativus*

The availability of the complete cucumber genome sequences facilitated the search for NBS-encoding genes. At present, two cucumber inbred lines, 9930 (the ‘Chinese long’, commonly used in modern cucumber breeding) and Gy14 (the gynoecious inbred line), have been sequenced. The former was sequenced using a combination of traditional Sanger and next-generation Illumina GA sequencing technologies [10] and a database has been established for the sequence formation (http://cucumber.genomics.org.cn/page/cucumber/index.jsp). The latter was sequenced *de novo* with an appropriate mixture of random shotgun and paired-end shotgun reads using a 454-XLR technology; these sequences have been uploaded to the JGI Genome database (http://genome.jgi-psf.org/cucumber/cucumber.home.html). In this study, the two databases were used to search for NBS-encoding genes.

NBS-encoding genes were identified for the first time in three steps using the JGI *Cucumis sativus* Genome database. The first step involved a BLASTN search using *A. thaliana* and rice NBS-encoding sequences [31-33] as the query. The second step aimed at a complete search of the candidate NBS-encoding genes in the cucumber gynoecious inbred line Gy14, and was performed using the amino acid sequence of the nucleotide-binding adaptor shared by APAF-1, R-proteins, and the CED-4 (NB-ARC) domain (Pfam: PF00931) as a query to find possible encoded homologues [22]. In the third step, based on the above results, the search of candidate NBS-encoding genes in the cucumber genome was repeated using BLASTN searches. The overall analysis reveals that the NBS-encoding gene family is composed of 57 members in the ‘Gy14’ cucumber gynoecious inbred line (Table 1). The predicted nucleotide and protein sequences of all 57 NBS-encoding genes from ‘Gy14’ are provided in Additional file 1.

**Table 1:** NBS-encoding or RGH genes in cucurbitaceous crops

**Name**	**Scientific name**	**JGI/GenBank accession numbers**		**Database/References**
		**TIR-NBS**	**CC-NBS**	
Cucumber	*Cucumis sativus*	Cacsa.089350, Cacsa.091460, Cacsa.091470, Cacsa.091680, Cacsa.091690, Cacsa.091710, Cacsa.091780, Cacsa.091820, Cacsa.091840, Cacsa.178450, Cacsa.155730, Cacsa.237390, Cacsa.237410, Cacsa.237440, Cacsa.237520, Cacsa.237530, Cacsa.237540, Cacsa.237560, Cacsa.249360, Cacsa.275630, Cacsa.292710, Cacsa.338650, Cacsa.338660	Cacsa.017460, Cacsa.017490, Cacsa.088220, Cacsa.091880, Cacsa.094560, Cacsa.094580, Cacsa.094650, Cacsa.094660, Cacsa.094670, Cacsa.102240, Cacsa.123410, Cacsa.128030, Cacsa.128100, Cacsa.128110, Cacsa.128130, Cacsa.128140, Cacsa.132370, Cacsa.133510, Cacsa.163670, Cacsa.178360, Cacsa.178620, Cacsa.189390, Cacsa.237070, Cacsa.239860, Cacsa.248810, Cacsa.251930, Cacsa.277260, Cacsa.318890, Cacsa.326910, Cacsa.328080, Cacsa.337180, Cacsa.37190, Cacsa.338110, Cacsa.338190,	http://genome.jgi-psf.org/
Melon	*Cucumis melo*	AF354505, AF354506, AF354510, AF354507, AF354516, AF354511, AF354504, AF354513	AF354515, AF354509, AF354514, AF354508*, AF354512*	[56]
			AY583855	[39]
		JN230661-JN230670		In this study
Bottle gourd	*Lagenaria siceraria*	JN230598, JN230599, JN230601, JN230602, JN230604, JN230606, JN230607, JN230608, JN230609, JN230612, JN230614, JN230615, JN230618, JN230620-JN230633, JN230635, JN230636, JN230637, JN230638, JN230639,	JN230600, JN230603, JN230605, JN230610, JN230611, JN230613, JN230616, JN230617, JN230619, JN230634, JN230640,	In this study
Luffa	*Luffa cylindrica*	JN230641- JN230660		In this study
Watermelon	*Citrullus lanatus*		DQ156558- DQ156564	http://www.ncbi.nlm.nih.gov/
		GU124539,GU124541, GU124544, GU124545,GU124546, GU124547, GU124548,GU124550, GU124551, GU124556,GU124557, GU124559, GU124562, GU124563,	GU124540, GU124542, GU124543, GU124553^#^, GU124554^#^, GU124560*	http://www.ncbi.nlm.nih.gov/
		JN230671-JN230676, JN230678- JN230701	JN230677	In this study
	*Citrullus colocynthis*	GU124549, GU124558, GU124561, GU124564,	GU124552*, GU124555*,	http://www.ncbi.nlm.nih.gov/
Squash	*Cucurbita moschata*	EF199755-EF199758, EF101660-EF101666	EF199760, EF199759, EF101667	http://www.ncbi.nlm.nih.gov/

*These sequences are pseudogenes. ^#^These genes contained no complete conserved motifs of P-loop to GLPL.

To classify these NBS-encoding proteins, each was identified based on N terminal CC motifs and TIR domains, as well as LRRs. A total of seven categories (TNL, CNL, TN, CN, N, NL and RPW8-NL) were identified (Table 2). Twenty-three proteins were shown to possess only NBS-LRRs. Two proteins, Cucsa.102240 and Cucsa.123410, were predicted to have a domain with a sequence similar to the *RPW8 Arabidopsis* powdery mildew resistance gene family [34]. Thirteen genes were identified with TIR domains at the N-terminal, and two of them, Cucsa.338660 and Cucsa.091460, were shown to lack LRRs. Eighteen genes were predicted to encode the CC motifs, and one of these genes lacked LRRs. The gene, Cucsa.237530, possesses only NBS domains (Table 2; Additional file 2).

**Table 2:** **Numbers of each family of NBS-encoding genes in *Cucumis sativus***

**Predicted protein domains**	**Letter code**	**Number of genes**
TIR-NBS-LRR	TNL	11
CC-NBS-LRR	CNL	17
TIR-NBS	TN	2
CC-NBS	CN	1
NBS	N	1
NBS-LRR	NL	23
RPW8-NBS-LRR	RPW8-NL	2
Total NBS genes		57

Previously, the number of NBS-encoding genes from the cucumber inbred line ‘Chinese Long’ 9930 had already been reported [10]. In this study, the methods mentioned above were also implemented to identify NBS-encoding genes from the ‘Chinese Long’ cucumber 9930 database (http://cucumber.genomics.org.cn/page/cucumber/index.jsp). These NBS-encoding genes are shown in Additional file 1. A comparison of Gy14 and 9930 cucumber NBS-encoding genes showed that most of the genes were highly similar to one another (Additional file 3). Therefore, in order to perform a comprehensive analysis of the NBS-encoding genes within the cucumber genome for comparison purposes, only NBS-encoding genes from the cucumber ‘Gy14’ genome were selected for further analyses.

### Phylogenetic analysis of cucumber NBS-encoding genes and exon-intron configurations

**Figure 1:**
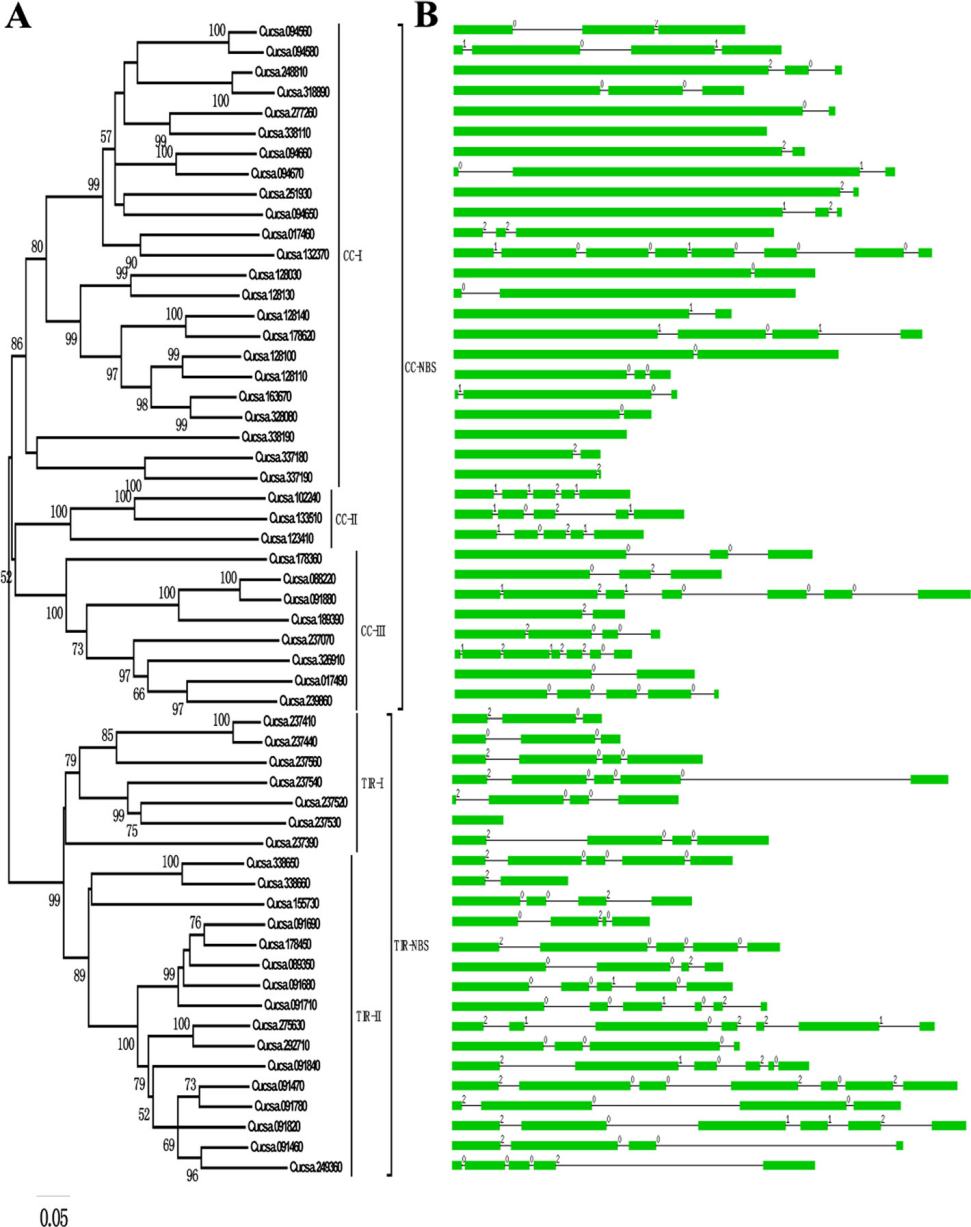
**Phylogenetic analysis and intron/exon configurations of NBS-encoding genes in cucumbers.** A phylogenetic tree of NBS-encoding genes was constructed using MEGA 5.0. Introns and exons are drawn to scale with the full encoding regions of their respective genes. Boxes indicate the exon, and lines indicate the intron. 0 = intron phrase 0; 1 = intron phrase 1; 2 = intron phrase 2.

Generally, 5’ region preceding the NBS and 3' region following the NBS have high variability and are not included for construction. The NBS region, however, is highly conserved and is often used to generate multiple sequence alignments and phylogenetic tree constructions [30]. In order to elucidate the relationships among the cucumber NBS-encoding genes, sequences of the NBS domain of these genes were used to construct a neighbor-joining (NJ) phylogenetic tree. These genes were divided into two families, the CC-NBS and TIR-NBS, which are supported by the high bootstrap values (Figure 1A). This was consistent with the results reported by Pan et al., which showed that both the TIR-NBS and CC-NBS families of genes occur in dicotyledonous plant species [24]. Moreover, each of these two families was separated into two subfamilies (CC-I/CC-II and TIR-I/TIR-II) with high bootstrap values (Figure 1A). In addition, it was observed that the members in CC-NBS were more numerous than those in the TIR-NBS family, which are composed of 34 and 23 members, respectively. This distribution is incongruent with that found in *Arabidopsis*. In the *Arabidopsis* genome, the member of the CC-NBS family was less than that of the TIR-NBS family [31]. Recently, the melon genome sequence was available [35], this distribution was also found in the NBS-LRR family (data not shown).

The exon/intron positions and phases of the 57 NBS-encoding genes in cucumber were further analyzed. The exon/intron structures were obtained using the online Gene Structure Display Server (GSDS: http://gsds.cbi.pku.edu.cn) with both coding sequences and genomic sequences [36]. Figure 1B provides a detailed illustration of the relative lengths of the introns and conservation of the corresponding exon sequences within each NBS-encoding gene in the cucumbers. The number of introns in all of these genes ranged from zero to seven. No intron was found in the Cucsa.338110, Cucsa.338190 or Cucsa.237530, whereas seven were found in Cucsa.132370. More than half of the genes had one to three introns (Additional file 4). Moreover, in the CC-I subfamily, all of the genes contained the lowest amount of introns (zero to three), except for Cucsa.132370, which contained seven introns. The genes in the TIR-II had the greatest number of introns (three to six), except for Cucsa.338660, which had only one intron. In the remaining two subfamilies, CC-II and TIR-I, the NBS-encoding genes contained zero to six introns (Figure 1B). These findings, together with the phylogenetic tree, indicate that some intron loss and intron gain events may have occurred during the structural evolution between the two families of cucumber NBS-encoding genes.

### Architectural diversity of NBS-encoding genes in *Cucumis sativus*

In order to understand the fine structure of the NBS-encoding genes in the cucumbers, CC- and TIR-NBS families were analyzed separately using the MEME and Clustal X programs. Moreover, these proteins usually have an N-terminal region, NBS domain, and LRR and C-terminal regions. Therefore, each of the two families of proteins were separated into three parts and subjected to protein domain and motif analyses.

#### The CC-NBS family

Three, ten and seven conserved motifs were identified in the N-terminal region (CC domain), NBS domain, and LRR and C-terminal regions, respectively (Table 3). Three motifs (C1, C2, and C3) were identified in the N-terminal region of most of the CC-NBS families of the cucumber NBS-encoding genes (Additional file 5). Most of the sites in these motifs were poorly conserved (Table 3, Additional file 6A). The NBS domain showed lower sequence diversity than the N-terminal domain. The eight motifs (P-loop, RNBS-A, Kinase-2, RNBS-B, RNBS-C, GLPL, RNBS-D, and MHDV) which were previously identified to occur widely in the NBS region of the known NBS-encoding genes in plant species [31], were also conserved in cucumber CC-NBS R-proteins with the exceptions of Cucsa.318890, Cucsa.091880, Cucsa.326910, Cucsa.132370, Cucsa.237070, Cucsa.094560 and Cucsa.094580, all of which have lost some conserved motifs (Additional file 5). In addition, two other conserved motifs (CNBS-1 and CNBS-2) in the NBS domain of the CC-NBS R-proteins were identified in the cucumbers. However, most of the sites in these two motifs were weakly conserved. Seven LRR-related motifs were found in CC-NBS R-proteins (Additional file 5). In contrast to the N-terminal and NBS domain, the LRR motif patterns were variable. Almost all proteins showed different LRR motif patterns. Among them, the conserved motifs L1 and L2 existed widely in all CC-NBS R proteins (Additional file 5).

**Table 3:** Major MEME motifs in predicted cucumber CC-NBS family of proteins

**Domain**	**Motif number nnunumber**	**Motif**	Motif sequence*	**Length**	**E-Value**
CC	Motif10	C1	xxaVkxWvxkLxdxxYe/dad/eDLLDExsYExLRRxVxxxxx	39	3.0e-183
	Motif20	C2	ntKqVRIFFSKSNQiaFrxK/rMxxkiKxi/vrEkLDaIxxe/dKtqfhLxxxxre	50	1.5e-090
	Motif16	C3	xxxxxxxxETxSf/si/llexeViGRe/dxe/dv/kxxIvxxl/vlDxsxxe	40	1.7e-121
NBS	Motif01	P-loop	xxIxGmGGxGKItLAkxxxxx	21	1.5e-278
	Motif06	RNBS-A-nonTIR	xFdxxiwVcVSxxFdxxxIlx	21	6.1e-201
	Motif03	Kinase-2	gKkYf/lLVl/mDDVWNexxxlWxxLKxxLmxx	29	2.9e-255
	Motif14	RNBS-B	xxGs/nxIlvTTRSxxvaxxxxt	21	7.4e-153
	Motif11	RNBS-C	hxlxxLxxxxswxlFxxxaxx	21	1.1e-180
	Motif08	GLPL	xexvxxxxGxPLaxxxxGxxl	21	2.9e-195
	Motif02	RNBS-D-nonTIR	xxxxlKxCFxyCSxFPkDxxi/f	21	6.5e-234
	Motif15	CNBS-1	xkxxLIxxWmAqGFiqxxxxx	21	1.2e-144
	Motif09	CNBS-2	xmEdi/vGe/dxYFxeLlsRxl/fFqdxxxxxxxx	29	4.5e-195
	Motif05	MHDV	xxKMHDlxh/rDxAxxixxxxxx	21	3.0e-213
LRR&C-terminal	Motif17	L1	lpxxixxLxhLryLdxsxx	19	8.7e-118
	Motif04	L2	LPxxixxLxxLxxLxlxxCxxLxxlPxxx	29	1.1e-250
	Motif07	L3	TLsxFvxGfxkGxki/lxELxxLxnLkGxLxlxxLexvx	37	4.6e-198
	Motif13	L4	xxxxxDxxVLEGLqPHxNlxxlxIxxf/yxG	29	2.8e-171
	Motif12	L5	pxxxFVENLVxIxLxxCxxcExLPmlgqL	29	6.6e-156
	Motif18	L6	xxxxxxxxxxfxxLxxlxixxCxxLxxxp	29	3.8e-095
	Motif19	L7	xxFPxLkxl/fxixxmxnLexWw	21	2.8e-108

*If the bit value (Fig. S1A) of the amino acid at this position is less than 1, it is represented with an x; 2 < bits ≤ 1, with lowercase; 3 < bits ≤ 4, with capital letter; bits ≥ 3, with underlined capital letters.

#### The TIR-NBS family

MEME was used to find motifs in the TIR region, NBS domain, and LRR region of the cucumber TIR-NBS family of NBS-encoding genes. A total of four, nine, and seven motifs were found, respectively. In the TIR region, four motifs (T1, T2, T3, and T4) existed in almost half of these proteins (Table 4). The conservation of the NBS domain in the TIR-NBS family was higher than that in the CC-NBS family. In this family, two proteins, Cucsa.237530 and Cucsa.338660, lacked the RNBS-D-TIR and MHDL domains, whereas Cucsa.249360 lacked the RNBS-C domain (Additional file 5). Compared with the CC-NBS family, only one additional motif (TNBS-1) was identified in the NBS domain of the TIR-NBS family (Additional file 5). In addition, although the same number of LRR-related motifs was identified in the TIR-NBS family, the CC-NBS and TIR-NBS families do not have identical motifs. Moreover, most of the sites of these motifs were poorly conserved (Table 4, Additional file 6B).

**Table 4:** Major MEME motifs in predicted cucumber TIR-NBS family of proteins

**Domain**	**Motif number**	**motif**	Motif sequence*	**Length**	**E-Value**
TIR	Motif03	T1	sxxxwxYDVFLSFRGeDTRxnFtxhLxxALrxxGi/vnvFi/rDx	41	1.1e-238
	Motif06	T2	CLxELVKIxxCkkxxxQxVLPv/iFYkv/iDPSxVRKQx	35	2.8e-187
	Motif13	T3	xxkvqxWRxAl/mtxa/vanlsGWxlxxxxxex	29	5.4e-095
	Motif18	T4	xIsxxLxkxIxxSxxsiVi/vfSexYAsSxW	29	4.7e-090
NBS	Motif01	P-loop	xGm/iGGIGKTTl/iAKalYnxixxxFexcCFL	29	3.6e-378
	Motif15	RNBS-A-TIR	gLvxLQxxLLxxilx	15	1.5e-082
	Motif02	RNBS-B	gxdWFGxGSr/kiIxTTRnxhLLxxxxf	26	2.4e-294
	Motif05	Kinase-2	iIr/kxRLxxKKvLiv/iLDDVDxxxQLxaLa/rG	29	1.8e-261
	Motif07	RNBS-C	iLFLFSwHAFxxxhPsxxYld	21	4.7e-178
	Motif04	GLPL	avxYckGLPLALxvLGSfLxx	21	7.1e-205
	Motif09	RNBS-D-TIR	keIFIdIsCxFvGexxxxvxxxl	23	1.5e-138
	Motif16	TNBS-1	xlxxxixxxLxiSy/fdgLexxx	21	1.2e-087
	Motif08	MHDL	Xxxxr/kxxMHdLIqxMGxxIvx	21	1.0e-160
LRR&C-terminal	Motif19	L1	xxxkrsRLWlxxdxxxxlxxxxgxxxxxxix	31	4.1e-073
	Motif20	L2	S/pxxLRwxnWhGf/yPxxxLPxxfxmxxLxeLxLpxSxixxfw	40	3.9e-066
	Motif10	L3	LxxxPDl/fSxaxNLexLxLxxCxxLxxxHxSv/igsL	34	9.8e-178
	Motif14	L4	lxlxxcxnlxxlPSxLxLKSLxxLxlxxCxkxExxPxfxenMKSLxxlxl	50	7.7e-092
	Motif12	L5	xtxixxLxxSIxxLxxLxxLxlxxCxxLxxLPxxIxxLxsL	41	1.8e-144
	Motif17	L6	TxLxLxxCn/kIt/sNxdFLEtlxxvapsLxxLxLSxNxFc/sxLPS	41	4.6e-082
	Motif11	L7	l/ixxf/lxsLxxLlxnCxLxxIpklPxxlxxxxaxgx	36	3.3e-136

*If the bit value (see Fig. S1B) of the amino acid at this position is less than 1, it is represented with x; 2 < bits ≤ 1, with lowercase; 3 < bits ≤ 4, with capital letter; bits ≥ 3, with underlined capital letters.

### Chromosomal distribution of cucumber NBS-encoding genes

**Figure 2:**
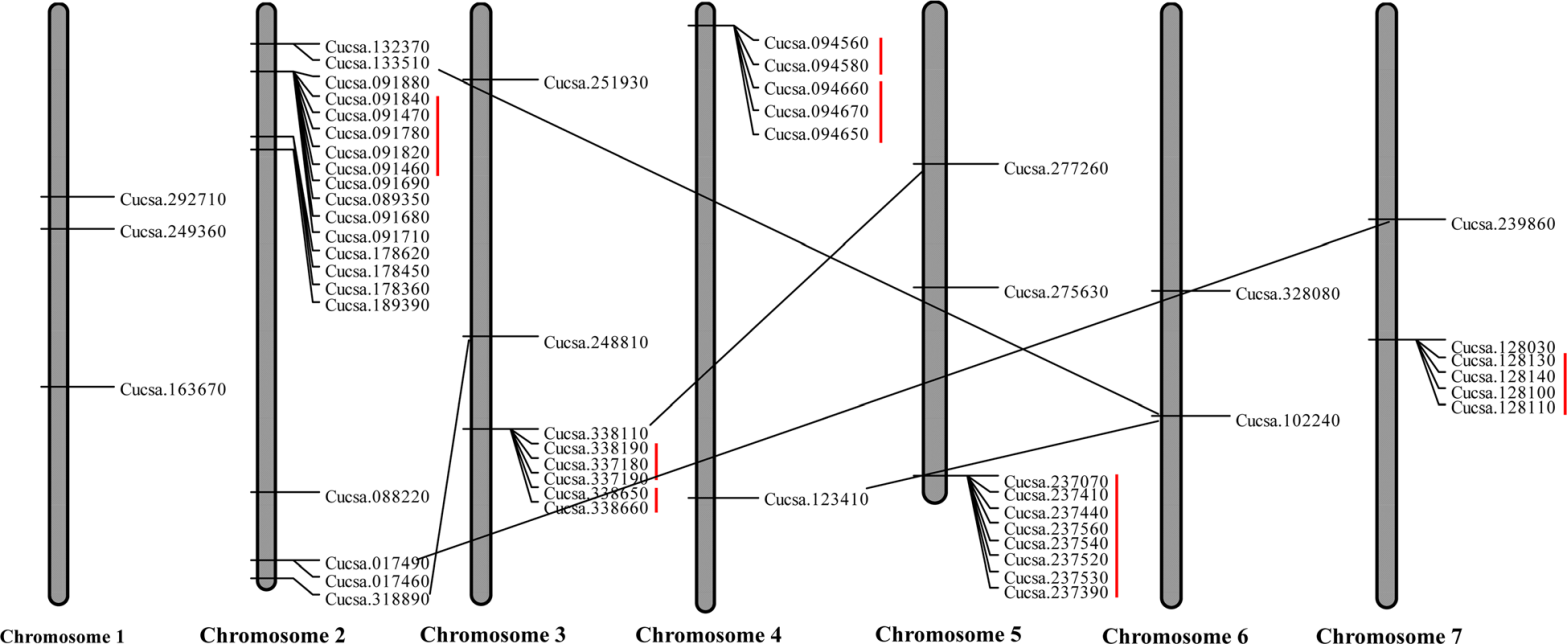
**Position of NBS-encoding genes on the cucumber chromosomes.** Chromosome numbers are indicated at the bottom end of the chromosome. Those located on sequence scaffolds are not shown. The straight lines connecting the NBS-encoding genes present on duplicated chromosomal segments, whereas the tandem duplicated gene clusters are indicated by red lines.

The GY14 cucumber genome encoded 57 members. Of them, 55 NBS-encoding genes could be located on all seven chromosomes (Figure 2). The remaining two genes (Cucsa.326910 and Cucsa.155730) could not be located on any chromosomes and were assigned to Scaffold03139 and Scaffold03138, respectively. As in *Arabidopsis* and rice, the distribution of cucumber NBS-encoding genes among chromosomes is nonrandom [31-33]. There were 10 and 20 genes which were localized on chromosomes 5 and 2, respectively, whereas only five genes were located on chromosomes 1 and 6. The remaining 20 NBS-encoding genes were located on chromosomes 3, 4 and 7.

In addition, it was determined that the NBS-encoding gene clusters by a combination of three approaches described in previous studies [31,37,38]. A gene cluster is defined as a region in which two neighboring homologous genes are less than 200 kb and fewer than eight non-NBS-encoding genes between NBS-encoding genes. It was also found that most of the NBS-encoding genes were clustered on the chromosomes. A total of 33 genes were localized within nine clusters. The largest cluster contained 10 NBS-encoding genes, while the smallest included only two genes, both of which were located on chromosome 2. Shared phylogenetic clades confirmed the NBS-encoding gene chromosomal duplication, with four and seven NBS-encoding gene segment and tandem duplication events in cucumber (Figure 2), and these genes concern chromosomes 2, 3, 6 and 7.

### Identification of NBS-encoding RGHs in other Cucurbitaceae crops

To gain an insight into the phylogenetic relationship of NBS-encoding genes in Cucurbitaceae crops, database mining and PCR amplification were employed to identify the NBS-encoding genes in melon, bottle gourd, luffa, watermelon, and squash (Table 1, Additional file 7). A total of 165 NBS sequences were obtained. Among these sequences, 43 belonged to bottle gourd, 20 to luffa, 24 to melon, 64 to watermelon, and 14 to squash. For bottle gourd and luffa, the NBS sequences were obtained by PCR amplification; for squash, the NBS sequences were derived from database mining; for melon and watermelon, 10 and 31 sequences were respectively drawn from PCR amplification, and the remaining 14 and 33 sequences were obtained by database mining. The NBS sequences from watermelon originated from two species. One was *Citrullus lanatus*, consisting of 58 NBS sequences. Among them, one NBS sequence, GU124560, is a pseudogene (gene with stop codon), and two NBS sequences, GU124553 and GU124554, lacked the complete conserved motifs from P-loop to GLPL. The remaining six NBS sequences were found in *Citrullus colocynthis*, in which GU124552 and GU124555 were pseudogenes. Among the 24 NBS sequences from melon, two are pseudogenes (AF354508 and AF354512), which were found in the *C*. *melo* species. The AY583855 sequence has been identified as a *Fusarium* R gene [39]. In addition, the NBS sequences were obtained from bottle gourd (*L. siceraria*), luffa (*L. cylindrica*), and squash (*C. moschata*). In the current study, the pseudogenes and sequences without conserved domains from P-loop to GLPL were excluded from further analysis. Among the 165 NBS sequences from the Cucurbitaceae crops, 103 NBS sequences (JN230598 to JN230701) were identified via degenerate PCR amplification. The remaining 62 were derived from the GeneBank database (Table 1).

### Phylogenetic analysis of NBS-encoding genes and RGHs in Cucurbitaceae crops

**Figure 3:**
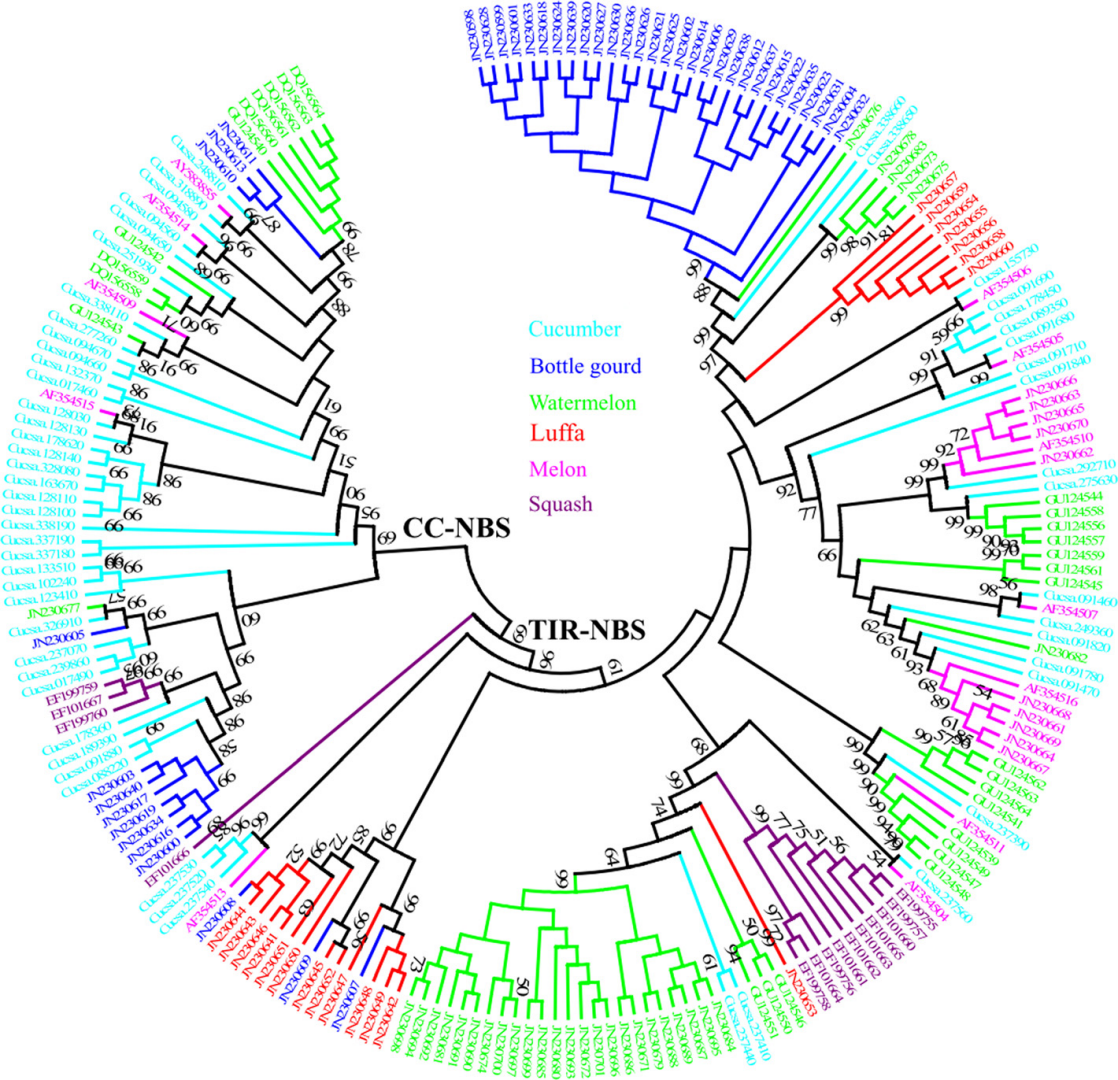
**Phylogenetic comparison of Cucurbitaceae NBS-encoding genes and RGHs.** TIR-NBS and CC-NBS families are distinguished. The scale represents the average number of substitutions per site. The numbers on the branches indicate the percentage of 1000 bootstrap replicates that support the node with only values > 50% reported. The detailed phylogenetic tree is shown in Additional file 8.

Phylogenetic analysis via the NJ method was conducted in order to determine the relationships among NBS-encoding genes and RGHs in the Cucurbitaceae crops. The consensus phylogenetic tree (Figures 3, Additional file 8) indicated that there were two distinct families, namely TIR-NBS and CC-NBS, which were consistent with the pattern previously described [23,40]. TIR-NBS and CC-NBS were further subdivided into nine and four distinct subfamilies (TIR1 to TIR9 and CC1 to CC4), respectively.

TIR1, TIR3 and TIR6 were the largest among the subfamilies, which were composed of 43, 35 and 40 sequences, respectively. TIR1 contained 29 sequences from bottle gourd, 5 from watermelon, 7 from luffa, and 2 from cucumber. The TIR3 subfamily included 13 sequences from cucumber, 14 from melon, and 8 from watermelon. The TIR6 subfamily contained 27 sequences from watermelon, 10 from squash, 2 from cucumber, and 1 from luffa. The TIR2, TIR5, TIR8 and TIR9 subfamilies are small, composed of 2, 2, 4, and 1 sequence, respectively. The remaining two subfamilies, TIR4 and TIR7, consisted of 10 and 15 sequences, respectively. Within the CC-NBS family, the largest subfamilies, CC1 and CC4, consist of 20 and 28 sequences, respectively, while CC2 and CC3 were relatively small subfamilies, composed of 3 and 12 sequences, respectively (Figures 3 and S4).

Interestingly, all NBS sequences obtained via PCR amplification in luffa belonged to the TIR (TIR1, TIR6 and TIR7) families, whereas the NBS sequences from the other five species were distributed widely among TIR- and CC-NBS families (Figures 3, Additional file 8).

### Comparison of NBS-encoding genes and RGHs from Cucurbitaceae and *A. thaliana*

**Figure 4:**
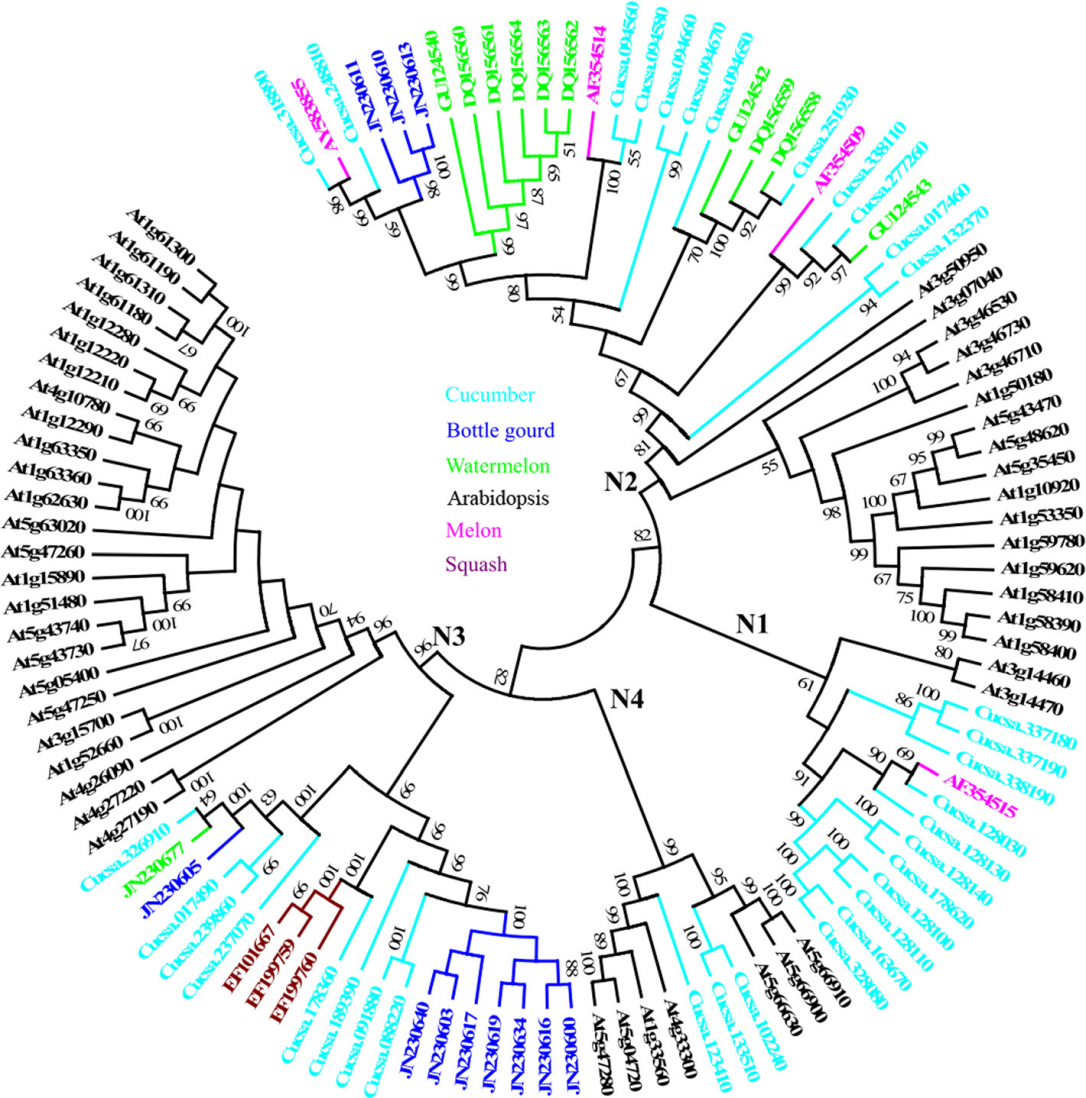
**Phylogenetic comparisons of Cucurbitaceae CC-NBS family of NBS-encoding genes and NBS-encoding genes with those in *Arabidopsis*.** The ancient CC family (N1 to N4) as defined by Cannon et al. [41] is indicated. The scale represents the average number of substitutions per site. The numbers on the branches indicate the percentage of 1000 bootstrap replicates which support the node with only values of > 50% reported. The detailed phylogenetic tree is shown in Additional file 9.

In the following analysis, *A. thaliana*, a member of Brassicaceae and the model system for genomic comparisons, was selected for phylogeny. The phylogenies were constructed using the P-loop to GLPL motifs. Previous phylogenetic analyses showed that all NBS sequences in *A. thaliana* are separated into two distinct families, CC and TIR [23]. A great divergence was observed between the two families. Therefore, separate analyses of these families were performed, and the results are shown in this paper. Figure 4 and Additional file 9 show the results of the phylogenetic analysis of the CC-NBS family between the Cucurbitaceae crops and *A. thaliana*. All members from the CC1, CC2, CC3 and CC4 subfamilies were detected in four clades (N2, N1, N4, and N3, respectively), using reference sequences from *Arabidopsis* as described by Cannon et al. [41]. Among the four clades, N2 was the largest, being composed of 12 members from cucumber, 3 from melon, 3 from bottle gourd, and 10 from watermelon. The other three clades were relatively small. N1 clade included all CC3 subfamilies from the Cucurbitaceae crops and two members, At3g14460 and At3g14470, from *A. thaliana*. However, relatively lower bootstrap values were observed in N1 and N2 clades. Low scores are usually an indication that the observed patterns must be analyzed with caution, and are more often observed for large datasets. Such poor scoring does not necessarily imply unreliable branching, but instead indicates that not all members may be assigned to a particular group with high confidence. The NBS-LRR superfamily accounts for the largest number of known disease resistance genes, and is one of the largest gene families among plant genomes [42]. High diversification of these genes was observed in the plants [30,38,40]. Therefore, it is believed that the diversification of NBS-encoding genes was responsible for the poor bootstrap score. This phenomenon has occurred frequently in constructing phyologenetic trees of NBS-encoding resistance genes from other plant species [7,43,44].

**Figure 5:**
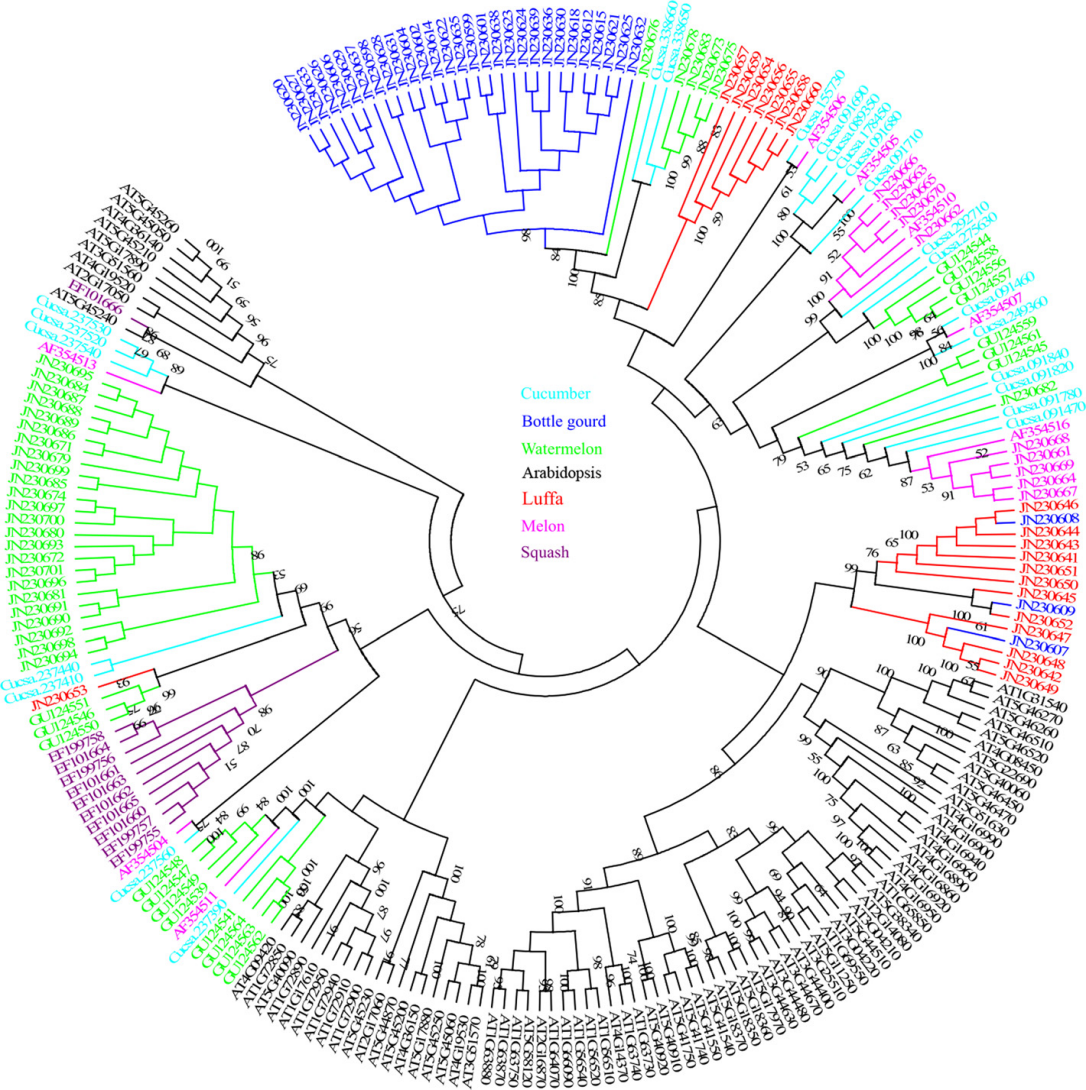
**Phylogenetic comparisons of Cucurbitaceae TIR-NBS family of NBS-encoding genes and NBS-encoding genes to those in *Arabidopsis*.** The scale represents the average number of substitutions per site. The numbers on the branches indicate the percentage of 1000 bootstrap replicates which support the node with only values of > 50% reported. The detailed phylogenetic tree is shown in Additional file 10.

Clade N3 included 8 sequences from cucumber, 8 from bottle gourd, 3 from squash, and 1 from watermelon. Clade N4 only contained three sequences from cucumber. Within the TIR family (Figures 5, Additional file 10), the nine subfamilies from the Cucurbitaceae crops remained different when compared with the TIR family of the sequences from *A. thaliana*. Sequences from TIR4 and TIR9 were detected in the At-TIR-NBS-B and At-TIR-NBS-A subfamilies, respectively. Each of the remaining seven subfamilies from the Cucurbitaceae crops formed a group.

The general features of the phylogenetic tree are presented in Figures 4 and 5. In most cases, each clade in the Cucurbitaceae crops was composed of sequences from more than one species. For example, clade N3 was composed of CC1 sequences from four species (cucumber, bottle gourd, squash, and watermelon), whereas clade N2 consisted of CC4 sequences from cucumber, melon, bottle gourd, and watermelon. A similar phenomenon was also observed in the TIR1 to TIR8 subfamilies. However, in TIR9 and CC2, squash and cucumber were species-specific subfamilies, that is, they were composed of sequences from only one species.

## Discussion

As the first sequenced vegetable crop, the cucumber genome will provide a valuable new resource for the biological research and breeding of cucurbits (http://cucumber.genomics.org.cn/page/cucumber/index.jsp). The Cucurbitaceae family, commonly known as cucurbits and gourds, includes many economically important cultivated plants, such as cucumber (*C. sativus* L.), melon (*C. melo* L.), bottle gourd (*Lagenaria siceraria* var. hispida), luffa *L. cylindrica* (L.) Roem.], watermelon *Citrullus lanatus* (Thunb.) Matsum. & Nakai], squash (*C. moschata* Duch.), and pumpkin (*Cucurbita* spp.) [45]. Developing disease resistance is one of the most important objectives of breeding Cucurbitaceae crops. In the current study, the first large scale analysis of NBS-encoding genes from cucumber was reported, as was that of RGHs from melon, watermelon, luffa, bottle gourd and squash. The results contribute to the identification of candidate R-genes and provide insight into NBS-encoding gene evolution in Cucurbitaceae crops.

### The cucumber genome encodes a small NBS-encoding gene family

Previous studies regarding NBS-encoding genes from *Vitis vinifera*, *Populus trichocarpa*, *Arabidopsis thaliana,* and *Oryza sativa* revealed that there are 535, 416, 174, and 519 genes in these species, respectively [46]. However, the GY14 cucumber genome only encodes 57 NBS-encoding genes, which is less than that in the four sequenced plant genomes (Additional file 11). Recently, Huang et al. identified a total of 59 NBS-encoding genes which were located on seven different chromosomes in the ‘Chinese Long’ cucumber genome [10]. These two cucumber lines have similar numbers of NBS-encoding genes (Additional file 3). Moreover, in these two cucumber genomes, the majority of conserved NBS-LRR R genes is single-copy and/or located as a singleton. The complex clustered NBS-LRR R genes contribute greatly to the rich genetic variation (data not shown). Recently, it was reported that there are 81 NBS-encoding genes in the melon genome [35]. It was found that there are significant differences in R gene counts among them. This result indicated that the degree of interspecific variation is greater than that of intraspecific variation in *Cucumis*.

Recently, Porter et al. reported that only 54 NBS-encoding genes are present in *Carica papaya*[47], which was similar to the number of these genes in the cucumbers (Additional file 11). Although the difference between the genome size and total number of predicted protein encoded genes among the six sequenced plant species was observed clearly (Additional file 11), the number of NBS-encoding genes does not increase or decrease proportionally. The obtained data indicate that, similar to *Carica papaya*, NBS-encoding genes in cucumber are a relatively small gene family. Based on the information regarding the cucumber genome [10], there are at least two explanations to this phenomenon. The first is the absence of the recent whole-genome duplication (WGD) in the small cucumber genome [10]. WGD is common in angiosperm plants and produces a tremendous source of raw materials for gene genesis, therefore the absence of WGD may have led to the small number of NBS-encoding genes in cucumber. The second explanation is the inclusion of a small number of tandem gene duplications and a few segmental duplications in the cucumber genome [10]. These duplications also contribute in part to the small number of NBS-encoding genes in cucumbers.

### Diversity of NBS-encoding genes in cucumber

Cucumber genome encodes 57 NBS-encoding genes and maintains both the TIR and CC families (Additional file 11 and Figure 1A), which suggests that the cucumber has relatively few, albeit diverse, NBS-encoding genes. The sequences of TIR- and CC-NBS family of NBS-encoding genes were aligned separately. The results revealed the presence of six previously identified motifs (P-loop, Kinase-2, RNBS-B, RNBS-C, GLPL, and MHDV) in most genes (Additional file 12). Motifs RNBS-A-TIR and RNBS-D-TIR occur exclusively in the TIR-NBS R proteins (Additional file 12A), whereas RNBS-A-nonTIR and RNBS-D-nonTIR are specific to the CC-NBS R proteins (Additional file 12B). However, some motifs present in the sequence alignment were not detected using the MEME software, suggesting that these motifs were poorly conserved in some of these proteins, such as the P-loop motif in Cucsa.017490, Cucsa.088220, Cucsa.094560, Cucsa.239860 and Cucsa.318890. This phenomenon was observed only in the CC-NBS family of NBS-encoding genes in cucumbers, suggesting that these genes are more conserved in the TIR-NBS family than those in the CC-NBS family. In previous studies, some researchers found that the CC family is highly diverse and originated prior to the split between gymnosperms and angiosperms. In contrast, the TIR family is more homogeneous and was found only in dicotyledon, suggesting that it arose after the divergence of monocotyledon and dicotyledon [41,48,49]. Therefore, the results of this study were consistent with those of previous reports.

The last residue of the kinase-2 motif, D (Aspartate) or W (Tryptophan), in the NBS-encoding genes in plants has also been used to predict (95% accuracy) whether they belong to the TIR- or CC-NBS family of the NBS-encoding genes [24]. In the current paper, the last residue in most of the kinase-2 motifs of TIR-NBS family of genes is “D”, except in Cucsa.237410, Cucsa.237540, Cucsa.237520, and Cucsa.091460, in which it was substituted for “Asparagine”, “Glutamine acid”, “Threonine” and “Asparagine”, respectively. For the CC-NBS family, the last residues in the kinase-2 motifs is “W” except in Cucsa.337190, Cucsa.338110, and Cucsa.338190, in which it was replaced with “Serine”, “Serine” and “Glycine”, respectively (Additional file 12). This class not only supports the results of the above phylogenetic analysis, but also the view that both the TIR- and CC-NBS families of genes occur in dicot species [50].

### Comparative evolutionary analysis of NBS-encoding genes from Cucurbitaceae crops and Arabidopsis

*Arabidopsis* is the model system for genomic comparisons among dicots, due to the fact that a complete draft of its genome is available [51]. In this study, both the TIR- and CC-NBS families were identified in all genes from the Cucurbitaceae crops (Figure 3). Separate phylogenies for the 2 families were subsequently constructed (Figures 4 and 5). The phylogenetic pattern of CC-NBS is shown in Figure 4 and Additional file 9. Subfamilies CC1, CC2, CC3 and CC4 of Cucurbitaceae fall within the subfamilies N3, N4, N1 and N2, respectively, as identified by Cannon et al. [41] No species-specific expansion in the CC-NBS family after the divergence of the Cucurbitaceae species and *Arabidopsis* was observed. In addition, the N1 subfamily contained only two members from *Arabidopsis*, At3g14460 and At3g14470, whereas subfamily CC3 of Cucurbitaceae was grouped into this subfamily. Thus, the analysis has identified a region of chromosome 3 of *Arabidopsis* which is potentially orthologous to the CC3 subfamily of Cucurbitaceae.

The phylogenetic comparison of TIR-NBS sequences from Cucurbitaceae and *Arabidopsis* revealed a degree of change as opposed to the phylogenetic pattern of the CC-NBS family (Figure 5). Similar to the results shown in Figure 3, most subfamilies were also shown to be species-specific in the phylogenetic analysis of TIR-NBS sequences from Cucurbitaceae and *Arabidopsis*, except for TIR9 and TIR4, which were combined into the At-TIR-NBS-A and At-TIR-NBS-B subfamilies, respectively [52]. This observation is similar to those described in Solanaceae and Asteraceae, and may be typical of other plant families as well [24,51], which suggests recent gene radiation from a common ancestral source of NBS-encoding genes or RGHs.

## Conclusions

The results of this study provide a genomic framework for the further isolation of candidate NBS-encoding genes in Cucurbitaceae crops through comparative genomics, and contribute to the understanding of the evolutionary mode of NBS-encoding genes in Cucurbitaceae crops. In 2009, the cucumber genome was sequenced by researchers who worked on the ‘Chinese Long’ inbred line 9930 [10]^,^ and recently Gy14 was sequenced *de novo*. A vast amount of useful information has been collected, and two cucumber genome databases (http://cucumber.genomics.org.cn/; http://genome.jgi-psf.org/cucumber/cucumber.home.html) have been established. However, information regarding other less studied Cucurbitaceae crops is still scarce, including that of melon, watermelon, luffa, bottle gourd, and squash. Thus, obtaining more NBS sequences from these other Cucurbitaceae crops should be the focus of future studies.

## Methods

### Retrieval and identification of cucumber NBS-encoding R genes

Cucumber (*Cucumis sativus* L.) assembly and annotation V1.0 were downloaded from http://www.phytozome.net/cucumber. A TBLASTN search was used to obtain all NBS-encoding genes in the cucumber (*C. sativus* L.) genome. First, a TBLASTN was performed using the protein coding sequences of the NBS domain of NBS-encoding sequences from *A. thaliana* and rice [31-33] as the query against the JGI *Cucumis sativus* genome database (http://genome.jgi-psf.org/cucumber/cucumber.home.html). Second, the amino acid sequence of the NB-ARC domain (Pfam: PF00931) was adopted as a query in TBLASTN searches for possible homologues encoded in the cucumber genome. The conserved NBS domain of these predicted NBS-encoding proteins was determined by Pfam version 22.0 ( http://pfam.janelia.org). Third, based on the results above, the searches of candidate NBS-encoding genes in the cucumber genome were repeated using BLASTN searches. The e-value used was 1e^-5^. Finally, all BLAST hits in the cucumber genome, together with flank regions of 5,000–10,000 bp in the upstream and downstream of BLAST hits, were annotated using the FGENESH (http://www.softberry.com/) and GENSCAN (http://genes.mit.edu/genescan.html/) programs.

To classify these NBS-encoding genes, all candidate genes were evaluated to further verify whether they encoded TIR, CC, NBS, or LRR motifs using the Pfam database (http://pfam.janelia.org/), SMART protein motif analyses (http://smart.embl-heidelberg.de/), and COILS, with a threshold of 0.9, to specifically detect CC domains [53]**.**

### Prediction of conserved motif structures and gene duplication

To investigate the diversity and structure of NBS-encoding genes in cucumbers, their predicted amino acid sequences were subjected to domain and motif analyses. According to the methods of previous researchers [31,54], NBS-encoding genes from cucumbers were divided into three components, namely the N-terminal, NBS domain, and LRR-C-terminal regions. They were then analyzed individually using the Multiple Expectation Maximization for Motif Elicitation (MEME)/Motif Alignment and Search Tool (MAST) system (http://meme.sdsc.edu/meme/website/intro.html). Furthermore, MEME motif analyses were performed on members of TIR-NBS and CC-NBS families. Conservation of each motif among the NBS-encoding genes was performed with WebLogo version 2.8.2 (http://weblogo.berkeley.edu/) using the default settings.

Gene duplication events of NBS-encoding genes were defined based on the http://criterion used by previous researchers [55]. NBS-encoding genes in cucumber were aligned using BioEdit (http://www.mbio.ncsu.edu/bioedit/bioedit.html) and calculated by MEGA 5.0 [56] for homology gene calculation.

### Identification of NBS-encoding RGHs in other Cucurbitaceae crops

To understand the phylogenetic relationship among the NBS-encoding genes in Cucurbitaceae crops, NBS-encoding RGHs from melon, bottle gourd, luffa, watermelon and squash were also identified via degenerate PCR amplification and database mining. First, PCR was performed using genomic DNA for young leaves from melon, bottle gourd, luffa, and watermelon using 3 pairs of degenerate primers. The young leaves in the second true-leaf stage were harvested, frozen immediately in liquid nitrogen, and stored at −80°C. Genomic DNA was isolated using a plant DNA extraction kit (Tiangen, China). The primers were designed by the previous researchers based on the conserved regions of P-loop and GLPL of amino acid identity among the known NBS-LRR R genes from the other plant species (Additional file 13). The PCR amplifications were performed in 20 μL reaction mixtures with 1 U of LATaq DNA proof reading polymerase (TaKaRa, Kyoto, Japan), 1 × PCR buffer, 1.5 mM MgCl_2_, 0.5 μM each of forward/reverse primers, 0.4 mM dNTP, and 50 ng of template DNA. PCR was performed in a PTC-100 thermal cycler (MJ Research, Inc., Watertown, MA). The cycling conditions consisted of an initial denaturation performed for 3 min at 94°C, followed by 35 cycles at 94°C for 30 s, 55°C for 45 s, and 72°C for 1 min. These were followed by a 10 min extension step at 72°C and 10°C to terminate the reaction.

The DNA fragments from the PCR were separated using 1.0% agarose gels. Fragments with the expected size (~500 bp) were excised and reclaimed from the gel and purified with a PCR purification kit (Qiagen, Germany). Subsequently, these fragments were combined with vector DNA to generate recombinant DNA molecules, and then transformed into competent *Escherichia coli* JM10^9^ cells. Plasmid DNA was purified with a PCR purification kit (Qiagen, Germany). The DNA fragments were sequenced using an ABI 3730 sequencer (Applied Biosystems, Foster City, CA, USA). Then, each of the acquired DNA sequences was trimmed of vector sequence contamination using VecScreen at the National Center of Biotechnology Information (NCBI). Identity and similarity searches of nucleotide and amino acid sequences were performed using BLAST at the NCBI GenBank database (http://www.ncbi.nlm.nih.gov/BLAST/).

Second, other RGHs in melon, watermelon, and squash were obtained from the GenBank database searches. All sequences from these species were downloaded and searched with the NBS domain of NBS-encoding sequences from *A. thaliana* and rice [31-33] as the query. The RGHs in melon were sourced from a published paper [57]. In addition, *Arabidopsis* NBS-encoding proteins, which were obtained from http://niblrrs.ucdavis.edu/At_RGenes/, were selected for phylogenetic relationship analysis.

### Sequence and phylogenetic analysis

Amino acid sequences of all NBS-encoding genes in the cucumber genome and RGHs from the other five Cucurbitaceae crops were aligned using Clustal X version 1.8 [58], followed by manual adjustment. The conserved domains of P-loop to GLPL of these proteins and RGHs were applied to construct a phylogenetic tree using the NJ method [59] and an NJ algorithm implemented in the Molecular Evolutionary Genetics Analysis software version 5.0 (MEGA 5.0) [56]. Bootstrapping (1000 replicates) was used to evaluate the degree of support for a particular grouping pattern in the phylogenetic tree. Branch lengths were assigned by pairwise calculations of the genetic distances, and missing data were treated by pairwise deletions of the gaps.

## Competing interests

The authors declare that they have no competing interests.

## Authors’ contributions

HJW participated in conceiving the paper, primer design, sequence analysis, and drafting the final manuscript. WY participated in DNA extraction and PCR amplification. KLB and SJ participated in bioinformatics, and modified the final manuscript. XP participated in conceiving the study, and modified the final manuscript. JFC critically reviewed the manuscript and gave financial support to the study. All authors read and approved the final manuscript.

## Supplementary Material

Additional file 1 **Coding DNA and protein sequences of the NBS-encoding genes from cucumbers (*Cucumis sativus* L).** Click here for file

Additional file 2 Predicted domains of each NBS-encoding genes and numbers of LRR motifs in cucumber. Click here for file

Additional file 3 Sequence identity between 9930 and Gy14 NBS-encoding genes. Click here for file

Additional file 4 Distribution of the NBS-encoding genes with different numbers of introns in cucumber. Click here for file

Additional file 5 **Motif patterns of NBS-encoding genes in cucumbers (*Cucumis sativus*L.).** (**A**) CC-NBS family (**B**) TIR-NBS family Click here for file

Additional file 6 **(A) Motif sequence logos in the cucumber CC-NBS family of NBS-encoding genes.** (**B**) Motif sequence logos in the cucumber TIR-NBS family of NBS-encoding genes. Click here for file

Additional file 7 PCR amplification products generated by the three selected resistance gene degenerate primers in the four major Cucurbitaceae crops. Click here for file

Additional file 8 **Phylogenetic comparison of Cucurbitaceae NBS-encoding genes and RGHs.** The TIR- and CC-NBS families are distinct. The former is divided into subfamilies TIR1 to TIR 9 and the latter is separated into subfamilies CC1 to CC4. Click here for file

Additional file 9 **The detailed phylogenetic tree from Figure **4**.** The tree consists of 63 Cucurbitaceae and 52 *Arabidopsis* CC-NBS protein sequences. Parentheses indicate the ancient CC family (N1 to N4) as defined by Cannon et al. [41]. Click here for file

Additional file 10 **The detailed phylogenetic tree from Figure **5**.** The tree consists of 152 Cucurbitaceae and 106 *Arabidopsis* TIR-NBS sequences. *Arabidopsis* subfamilies are indicated by At-TIR-NBS-A to At-TIR-NBS-G corresponding to the seven TIR subfamilies identified by Richly et al. [52]. Click here for file

Additional file 11 **Total number of predicted NBS-encoding genes identified in the six sequenced angiosperm genomes.** The values for *Carica papaya*, *Arabidopsis thaliana*, *Vitis vinifera*, *Oryza sativa*, and *Populus trichocarpa* were previously summarized by Yang et al. [37] and Porter et al. [48]. The total number of predicted protein encoding genes and the genome size of each species is also shown. Click here for file

Additional file 12 **Amino acid sequence alignment of NBS-encoding genes.** The conserved domains are highlighted and indicated by an arrow. The alignment was constructed using Clustal X software. Click here for file

Additional file 13 Degenerate primers used for PCR amplification from the four major Cucurbitaceae crops (melon, bottle gourd, luffa and watermelon). Click here for file

## References

[B1] Wyszogrodzka AJ Williams PH Peterson CE Multiple-pathogen inoculation of cucumber (Cucumis sativus) seedlings Plant Dis 1987 71 275 280

[B2] Abul-Hayja ZM Multiple disease screening and genetics of resistance cucumber 1975 Madison: University of Wisconsin 149 Ph. D. thesis

[B3] Palmer MJ Williams PH A seedling evaluation method for Fusarium wilt of cucumber incited by Fusarium oxysporum f.sp. cucumerinum. (Abstr.) Phytopathol 1981 71 247

[B4] Chen JF Moriarty G Jahn M Lebeda A, Paris HS Some disease resistance tests in Cucumis hystrix and its progenies from interspecific hybridization with cucumber Progress in cucurbit genetics and breeding research. Proceedings of Cucurbitaceae 2004, the 8th EUCARPIA Meeting on Cucurbit Genetics and Breeding 2004 Olomouc (CZ): Palacky University in Olomouc 189 196

[B5] Chen JF Lewis S New source of nematode resistance was identified in Cucumis Cucurbit Genet Coop Rep 2000 23 32 35

[B6] Ellis J Jones D Structure and function of proteins controlling strain-specific pathogen resistance in plant Curr Opin Plant Biol 1998 1 288 293 10066601 10.1016/1369-5266(88)80048-7

[B7] Wan HJ Zhao G Ahmed AM Qiao CT Chen JF Identification and characterization of potential NBS-encoding resistance genes and induction kinetics of a putative candidate gene associated with downy mildew resistance in Cucumis BMC Plant Biol 2010 10 186 20731821 10.1186/1471-2229-10-186 PMC2956536

[B8] Wan HJ Chen JF Characterization of NBS-LRR resistance gene analogs from a high resistance to downy mildew introgression line from Cucumis hystrix x C. sativus Acta. horticul 2010 871 573 578

[B9] Wan HJ Qiao CT Ahmed AM Zhao ZG Chen JF Isolation, phylogeny and evolutionary analysis of Pto-type disease resistance gene analogues from a Cucumis hystrix introgression line of cucumber (C. sativus) Funct Plant Biol 2010 37 513 523

[B10] Huang S Li R Zhang Z Li L Gu X Fan W Lucas WJ Wang X Xie B Ni P Ren Y Zhu H Li J Lin K Jin W Fei Z Li G Staub J van der Kilian A Vossen EA Wu Y Guo J He J Jia Z Tian G Lu Y Ruan J Qian W Wang M Huang Q Li B Xuan Z Cao J Asan Wu Z Zhang J Cai Q Bai Y Zhao B Han Y Li Y Li X Wang S Shi Q Liu S Cho WK Kim JY Xu Y Heller-Uszynska K Miao H Cheng Z Zhang S Wu J Yang Y Kang H Li M Liang H Ren X Shi Z Wen M Jian M Yang H Zhang G Yang Z Chen R Ma L Liu H Zhou Y Zhao J Fang X Fang L Liu D Zheng H Zhang Y Qin N Li Z Yang G Yang S Bolund L Kristiansen K Li S Zhang X Wang J Sun R Zhang B Jiang S Du Y The genome of the cucumber, Cucumis sativus L Nat Genet 2009 41 1275 1281 19881527 10.1038/ng.475

[B11] Kang HX Weng YQ Yang YH Zhang ZH Zhang SP Mao ZC Cheng GH Gu XF Huang SW Xie BY Fine genetic mapping localizes cucumber scab resistance gene Ccu into an R gene cluster Theor Appl Genet 2011 122 795 803 21104067 10.1007/s00122-010-1487-2

[B12] Helm MA Hemleben V Characterization of a new prominent satellite DNA of Cucumis metuliferus and differential distribution of satellite DNA in cultivated and wild species of Cucumis and in related genera of Cucurbitaceae Euphytica 1997 94 219 226

[B13] Sanjur OI Piperno DR Andres TC Wessel-Beaver L Phylogenetic relationships among domesticated and wild species of Cucurbita (Cucurbitaceae) inferred from a mitochondrial gene: Implications for crop plant evolution and areas of origin Proc Natl Acad Sci USA 2002 99 535 540 11782554 10.1073/pnas.012577299 PMC117595

[B14] Levi A Thomas CE Simmons AM Thies JA Analysis based on RAPD and ISSR markers reveals closer similarities among Citrullus and Cucumis species than with Praecitrullus fistulosus (Stocks) Pangalo Genet Resour Crop Evol 2005 52 465 472

[B15] Sikdar B Bhattacharya M Mukherjee A Banerjee A Ghosh E Ghosh B Roy SC Genetic diversity in important members of Cucurbitaceae using isozyme, RAPD and ISSR markers Biol Plant 2010 54 135 140

[B16] Schaefer H Heibl C Renner SS Gourds afloat: a dated phylogeny reveals an Asian origin of the gourd family (Cucurbitaceae) and numerous oversea dispersal events Proc R Soc B 2009 276 843 851 10.1098/rspb.2008.1447 PMC2664369 19033142

[B17] Dangl JL Jones JDG Plant pathogens and integrated defence responses to infection Nature 2001 411 826 833 11459065 10.1038/35081161

[B18] McDowell JM Woffenden BJ Plant disease resistance genes: recent insights and potential applications Trends Biotech 2003 21 178 183 10.1016/S0167-7799(03)00053-2 12679066

[B19] Martin GB Bogdanove AJ Sessa G Understanding the function of plant disease resistance proteins Annu Rev Plant Biol 2003 54 23 61 14502984 10.1146/annurev.arplant.54.031902.135035

[B20] Keen NT Gene-for-gene complementarity in plant-pathogen interactions Annu Rev Genet 1990 24 447 473 2088175 10.1146/annurev.ge.24.120190.002311

[B21] Van der Hoorn RAL Kamoun S From guard to decoy: a new model for perception of plant pathogen effectors Plant Cell 2008 20 2009 2017 18723576 10.1105/tpc.108.060194 PMC2553620

[B22] Van der Biezen EA Jones IDG Plant disease resistance proteins and the gene-for-gene concept Trends Plant Sci 1998 23 454 456 10.1016/s0968-0004(98)01311-5 9868361

[B23] Meyers BC Dickerman AW Michelmore RW Sivaramakrishnan S Sobral BW Young ND Plant disease resistance genes encode members of an ancient and diverse protein family within the nucleotide-binding superfamily Plant J 1999 20 317 332 10571892 10.1046/j.1365-313x.1999.t01-1-00606.x

[B24] Pan QL Liu YS Budai-Hadrian O Sela M Carmel-Goren L Zamir D Fluhr R Comparative genetics of nucleotide binding site-leucine rich repeat resistance gene homologues in the genomes of two dicotyledons: tomato and Arabidopsis Genetics 2000 155 309 322 10790405 10.1093/genetics/155.1.309 PMC1461067

[B25] Kanazin V Mareck L Shoemaker P Resistance gene analogs are conserved and clustered in soybean Proc Natl Acad Sci USA 1996 93 11746 11750 8876208 10.1073/pnas.93.21.11746 PMC38129

[B26] Leister D Ballvora A Salamini F Gebhardt C A PCR-based approach for isolating pathogen resistance genes from potato with potential for wide application in plants Nat Genet 1996 14 421 429 8944022 10.1038/ng1296-421

[B27] Shen KA Meyers BC Islam-Faridi MN Chin DB Stelly DM Michelmore RW Resistance gene candidates identified by PCR with degenerate oligonucleotide primers map to clusters of resistance genes in lettuce Mol Plant Microbe Interact 1998 11 815 823 9675895 10.1094/MPMI.1998.11.8.815

[B28] Noir S Combes MC Anthony F Lashermes P Origin, diversity and evolution of NBS-type disease-resistance gene homologues in coffee trees (Coffea L.) Mol Genet Genomics 2001 265 654 662 11459185 10.1007/s004380100459

[B29] Martínez-Zamora MG Castagnaro AP Díaz-Ricci JC Isolation and diversity analysis of resistance gene analogues (RGHs) from cultivated and wild strawberries Mol Genet Genomic 2004 272 480 487 10.1007/s00438-004-1079-4 15565466

[B30] Nair RA Thomas G Isolation, characterization and expression studies of resistance gene candidates (RGCs) from zingiber spp Theor Appl Genet 2007 116 123 134 17928987 10.1007/s00122-007-0652-8

[B31] Meyers BC Kozik A Griego A Kuang H Michelmore RW Genome-wide analysis of NBS-LRR genes in Arabidopsis Plant Cell 2003 15 809 834 12671079 10.1105/tpc.009308 PMC152331

[B32] Zhou T Wang Y Chen JQ Araki H Jing Z Jiang K Shen J Tian D Genome-wide identification of NBS genes in rice reveals significant expansion of divergent non-TIR NBS genes Mol Genet Genomics 2004 271 402 415 15014983 10.1007/s00438-004-0990-z

[B33] Yang SH Feng ZM Zhang XY Jiang K Jin XQ Hang YY Chen LQ Tian DC Genome-wide investigation on the genetic variations of rice disease resistance genes Plant Mol Biol 2006 62 181 193 16915523 10.1007/s11103-006-9012-3

[B34] Xiao S Ellwood S Calis O Patrick E Li T Coleman M Turner JG Broad-spectrum mildew resistance in Arabidopsis thaliana mediated by RPW8 Science 2001 291 118 120 11141561 10.1126/science.291.5501.118

[B35] Garcia-Mas J Benjak A Sanseverino W Bourgeois M Mir G Gonzalez VM Henaff E Camara F Cozzuto L Lowy E Alioto T Capella-Gutierrez S Blanca J Canizares J Ziarsolo P Gonzalez-Ibeas D Rodriguez-Moreno L Droege M Du L Alvarez-Tejado M Lorente-Galdos B Mele M Yang L Weng Y Navarro A Marques-Bonet T Aranda MA Nuez F Pico B Gabaldon T Roma G Guigo R Casacuberta JM Arus P Puigdomenech P The genome of melon (Cucumis melo L.) Proc Natl Acad Sci USA 2012 109 11872 11877 22753475 10.1073/pnas.1205415109 PMC3406823

[B36] Guo AY Zhu QH Chen X Luo JC GSDS: a gene structure display server Yi Chuan 2007 29 1023 1026 17681935

[B37] Yang S Zhang X Yue JX Tian D Chen JQ Recent duplications dominate NBS-encoding gene expansion in two woody species Mol Genet Genomics 2008 280 187 198 18563445 10.1007/s00438-008-0355-0

[B38] Florian J Leighton P Graham JE Katrin M Peter JAC Frank W Sanjeev KS Dan B Glenn B Jonathan DGJ Ingo H Identification and localisation of the NB-LRR gene family within the potato genome BMC Genomics 2012 13 75 22336098 10.1186/1471-2164-13-75 PMC3297505

[B39] Tarek J Joseph JK Shelly JN Claude ET Ralph AD The Fusarium wilt resistance locus Fom-2 of melon contains a single resistance gene with complex features Plant J 2004 39 283 297 15255859 10.1111/j.1365-313X.2004.02134.x

[B40] Pan Q Wendel J Fluhr R Divergent evolution of plant NBS-LRR resistance gene homologues in dicot and cereal genomes J Mol Evol 2000 50 203 213 10754062 10.1007/s002399910023

[B41] Cannon SB Zhu HY Baumgarten AM Spangler R May G Cook DR Young ND Diversity, distribution, and ancient taxonomic relationships within the TIR and non-TIR NBS-LRR resistance gene families J Mol Evol 2002 54 548 562 11956693 10.1007/s0023901-0057-2

[B42] Ameline-Torregrosa C Wang BB OòBleness MS Identification and characterization of nucleotide-binding site-leucine-rich repeat genes in the model plant Medicago truncatula Plant Physiol 2008 146 5 21 17981990 10.1104/pp.107.104588 PMC2230567

[B43] Xu Q Wen XP Deng XX Isolation of TIR and nonTIR NBS-LRR resistance gene analogues and identification of molecular markers linked to a powdery mildew resistance locus in chestnut rose (Rosa roxburghii Tratt) Theor Appl Genet 2005 111 819 830 16075209 10.1007/s00122-005-0002-7

[B44] Peraza-Echeverria S Dale JL Harding RM Smith MK Collet C Characterization of disease resistance gene candidates of the nucleotide binding site (NBS) type from banana and correlation of a transcriptional polymorphism with resistance to Fusarium oxysporum f.sp. cubense race 4 Mol Breeding 2008 22 565 579

[B45] Reddy BU Cladistic analyses of a few members of Cucurbitaceae using rbcl nucleotide and amino acid sequences Int J Bioinf Res 2009 1 58 64

[B46] Sanseverino W Roma G De Simone M Faino L Melito S Stupka E Frusciante L Ercolano MR PRGdb: a bioinformatics platform for plant resistance gene analysis Nucleic Acids Res 2010 38 D814 821 19906694 10.1093/nar/gkp978 PMC2808903

[B47] Porter BW Paidi M Ming R Alam M Nishijima WT Zhu YJ Genome-wide analysis of Carica papaya reveals a small NBS resistance gene family Mol Genet Genomic 2009 281 609 626 10.1007/s00438-009-0434-x 19263082

[B48] Tarr DEK Alexander HM TIR-NBS-LRR genes are rare in monocots: evidence from diverse monocotorders BMC Res Notes 2009 2 197 19785756 10.1186/1756-0500-2-197 PMC2763876

[B49] Bai JF Pennill LA Ning JC Lee SW Ramalingam J Webb CA Zhao B Sun Q Nelson JC Leach JE Hulbert SH Diversity in nucleotide binding site-leucine-rich repeat Genes in cereals Genome Res 2002 12 1871 1884 12466291 10.1101/gr.454902 PMC187567

[B50] Tuskan GA Difazio S Jansson S Bohlmann J Grigoriev I Hellsten U Putnam N Ralph S Rombauts S Salamov A Schein J Sterck L Aerts A Bhalerao RR Bhalerao RP Blaudez D Boerjan W Brun A Brunner A Busov V Campbell M Carlson J Chalot M Chapman J Chen GL Cooper D Coutinho PM Couturier J Covert S Cronk Q Cunningham R Davis J Degroeve S Déjardin A Depamphilis C Detter J Dirks B Dubchak I Duplessis S Ehlting J Ellis B Gendler K Goodstein D Gribskov M Grimwood J Groover A Gunter L Hamberger B Heinze B Helariutta Y Henrissat B Holligan D Holt R Huang W Islam-Faridi N Jones S Jones-Rhoades M Jorgensen R Joshi C Kangasjärvi J Karlsson J Kelleher C Kirkpatrick R Kirst M Kohler A Kalluri U Larimer F Leebens-Mack J Leplé JC Locascio P Lou Y Lucas S Martin F Montanini B Napoli C Nelson DR Nelson C Nieminen K Nilsson O Pereda V Peter G Philippe R Pilate G Poliakov A Razumovskaya J Richardson P Rinaldi C Ritland K Rouzé P Ryaboy D Schmutz J Schrader J Segerman B Shin H Siddiqui A Sterky F Terry A Tsai CJ Uberbacher E Unneberg P Vahala J Wall K Wessler S Yang G Yin T Douglas C Marra M Sandberg G Van de Peer Y Rokhsar D The genome of black cottonwood, Populus trichocarpa (Torr. & Gray) Science 2006 313 1596 1604 16973872 10.1126/science.1128691

[B51] Plocik A Layden J Kesseli R Comparative analysis of NBS domain sequences of NBS-LRR disease resistance genes from sunflower, lettuce, and chicory Mol Phylogenet Evol 2004 31 153 163 15019616 10.1016/S1055-7903(03)00274-4

[B52] Richly E Kurth J Lesiter D Mode of amplification and reorganization of resistance genes during recent Arabidopsis thaliana evolution Mol Biol Evol 2002 19 76 84 11752192 10.1093/oxfordjournals.molbev.a003984

[B53] Lupas A Van Dyke M Stock J Predicting coiled coils from protein sequences Science 1991 252 1162 1164 2031185 10.1126/science.252.5009.1162

[B54] Kohler A Rinaldi C Duplessis S Baucher M Geelen D Duchaussoy F Meyers BC Boerjan W Martin F Genome-wide identification of NBS resistance genes in Populus trichocarpa Plant Mol Biol 2008 66 619 636 18247136 10.1007/s11103-008-9293-9

[B55] Cheng Y Li XY Jiang HY Ma W Miao WY Yamada T Zhang M Systematic analysis and comparison of nucleotide-binding site disease resistance genes in maize FEBS J 2012 279 2431 2443 22564701 10.1111/j.1742-4658.2012.08621.x

[B56] Tamura K Peterson D Peterson N Stecher G Nei M Kumar S MEGA5: molecular evolutionary genetics analysis using maximum likelihood, evolutionary distance, and maximum parsimony methods Mol Biol Evol 2011 28 2731 2739 21546353 10.1093/molbev/msr121 PMC3203626

[B57] Brotman Y Silberstein L Kovalski I Perin C Dogimont C Resistance gene homologues in melon are linked to genetic loci conferring disease and pest resistance Theor Appl Genet 2002 104 1055 1063 12582612 10.1007/s00122-001-0808-x

[B58] Thompson JD Gibson TJ Plewniak F Jeanmougin F Higgins DG The CLUSTAL_X windows interface: flexible strategies for multiple sequence alignment aided by quality analysis tools Nucleic Acids Res 1997 25 4876 4882 9396791 10.1093/nar/25.24.4876 PMC147148

[B59] Saitou N Nei M The neighbor-joining method: a new method for reconstructing phylogenetic trees Mol Biol Evol 1987 4 406 425 3447015 10.1093/oxfordjournals.molbev.a040454

