# Supplementary material for: Genome-wide analysis of NBS-encoding disease resistance genes in Cucumis sativus and phylogenetic study of NBS-encoding genes in Cucurbitaceae crops
Source: BMC Genomics. 2013 Feb 19;14:109. doi: 10.1186/1471-2164-14-109 (PMC3599390; doi:10.1186/1471-2164-14-109)
Supplement: Additional file 1 — Coding DNA and protein sequences of the NBS-encoding genes from cucumbers (Cucumis sativus L). [file 1471-2164-14-109-S1.doc]

Coding DNA sequences of the NBS-encoding genes from Gy14 cucumber (Cucumis sativus L.)

>Cucsa.017460

ATGGCTGAGTTCTTATGGACTTTTGCTGTGCAAGAAATTTTGAAGAAGGTCCTAACTCTTGTGGCTGAGCAGATCATTCTAGCAAGGGAGGTCAAGGATGTGCTACAACAACTACAAAAAGAGCTAGTTGAGTCTCAAAAAATTGTTAGTGCTATCACTACCCAAAGACAAAATCATTATTCACCAGATAGCTTAGTGACTCAGTGGGTGAATGATCTTCAACTTATTGTTCATGAGGCTGACGACTTGCTGGATTTTATATTGAATAAAAATCAACCCATCGAGAGGTTATGGTCTGTGATCTCTTTATCTTGCCTGCTATATTCTTCAAACCCTGAGACTAAAAAAATGAAGGAAATTATAGCGTTGTTAAACAAACATTGCACCAAATTACCTCACTTACTTCAACTTGAGCCTACACCCTCAAACATTGCAGAGACTGAAGTTGCGCAAATTCAAGAGACAGTCTCAAAGCCTGAAGATTATGTGGTGGGAAGGAACAGGGAAGTTGAAACCATAGTTGATCGAGTGATTGATGCCAGCAAACAGGAACTCAATTCTATTTTACCCGTTTTTGGAATGGGTGGATTAGGAAAAACCACTTTGGCAAAGTCGGTCTTCAACCATGATAGGATCAAAAATCATTTTGGTATCACTATTTGGATATATGTGTCACAACCTTTTGTCATCAACAACATTTTGCAAGCAATCTTACAAAAGGTGGAGGTTCATTCTAGTGATTGCTCCAACAATAGGGAGGCCTTACTTGAAAAGCTTACAGAAAACATGGGAGAGAAAACATATTTTCTTGTTCTTGACGATGTTTGGAATGAAAACAAAATGTTGTGGGAGAAGTTGAAGGAATGTTTGATGAGTATTACTCATATGTCAGGAAATAGTATTCTTGTCACTACAAGGAGCAGTGGAATTGCAAAAATGATGGAAGAAAATATTGGAAGTCATGAATTAAGAAAATTATCTGATGACCAATGTTGGTCAATATTTAGGAACTTTGCCAATGCCAAGGATGTACCAATGACTTCCAATTTGGAGTTTGTGCAAAAAGAGTTTGATAAAAGAATCGGTGGTCTTCCATTAATCGCTAAAGTTTTGGGAGCAGCAGTTCCATTTTCAGGAGACCATGACCAGTGGGTAGCAAATATAAAAAGCGTTCTAACAACTCCAATAAAAGAGGAAGAGTTTGTTAAATTCACATTGAAGTTAAGCGTTGATCGTCTACCAAATGCTTCAGTAAAGCAGTGTTTTGCTTATTGTTCAAATTTTTCCAAGGGTTGTGAGTTTGACAAAAAGCAAGTGATTCGAATGTGGATGGCACAAGGATTTACTCAACCAGATGAAAGAAACAATGAAACAATGGAAGATACAGGAGAAAGGTACTTTAACATCTTGTTGTCTTTCTGCTTATTTCAAGATGTTGTTAAGAATGAAAGAGGAATAATTGAGAAGGTTCGAATGCATGATCTTATACATGATATTGCTTGTCAAGTTTCAAATGATAAAAAGTTGCGAATAGATCACATCATTTCATCAAATTGGAAAGATTGGACGAAAGATGACAAAATACTTGTGAGCAAGTTACGAACAATAAATTTTTATGATCGTCATCATGTGGTGGTTCAGGATAAGATTGGGGACTTTACTGGTTTGCGTGTTTTGACAATTGAAAATTATATTGTTGAGGAGTTACCAAACTCAATATTCAAGTTGAAGCACTTGAGATATCTAGACATTTCGTATTGTTATTCAATAAAGAAGCTTCCTGAATCTATTGTTCTGCTTTATAATTTGCAAACACTGAGATTTCATCTCTTAAGCAAGGGATTTCTACCAAAAAACGTTGGACAAATGATTAGTTTGAGGCATTTGGAGTTCTCATCTATCGATAAACAAATGTCTCCCTATTTGAGTCAATTGATTCAACTTGAAACATTGCCTAAATTTGCAGTAGGTTTTGAGAAGGGTTGTAAGATTACAGAACTTGGGGTTCTAAGAAACCTGAAAGGGTTGTTGAAGCTTCAACGTTTAGAACATGTTGAAAGTAAAGAAGAAGCCGAAACTGCAAAATTAGTGGAAAAGGAGAATCTAGAAGAAGTACATTTTGTGTGGACAAAGGAAAGGAAGAGAAAAGTAGAGAATAAGAATGATTTGGAAGTGTTGGAAGGACTTCAACCACCCAAAAATGTAGAATATTTGAGAATCAAATACTTTTTAGGTGGGTGTTTACCAAACCAGACGTTTGTTGAGAATTTAGTGAAAATAGAGCTAAGAGATTGTGGAAATTGTGAGAAGCTTCCAAGGCTTGGGCAATTAGGAAATCTAGAGATACTTGATATTTCATGGTTTGAAAGAGTAAAGAGTATAGGGAATGAATTCTATGGAAACAGCTCCAACAACCAAAGGAGTTTATTCCCCAGGTTGAAGGAATTGTATGTTGATGAGATGAGGAGGATAGGAGAATGGGAAGAAGTGGGAAGTAATGTTAAAGCTTTCCCACGTCTTGAACGTTTGTATATTGGTTGTTGTAGAGATTTAGTGAAAATTCCAGATGTTTTTGGGTATTGTGATGAGTATGGTGAGAAGCATCTGGAAGTTGTGGAAATTATTGAACATTTGTG

>Cucsa.017490

ATGGCAGGTGTTGGAAAAACTTATTTTCTTAACGAAGTTAAGAAATTGGTGTTGAAAGGGGAAGACAGATTGTTTGATCGAGTTATTGATGTGCGTGTAGGTCGATTTAATGATGTAACAGACATACAAGAACAAATTGGTGATCAATTGAACGTAGAATTGCCAAAAAGTAAAGAGGGAAGAGCGTCTTTTCTACGGAATAATTTGGCGAAAATGGAAGGTAATATCCTCATTTTATTAGATGATTTGTGGAAGGAATATGATCTTTTAAAAGAGATTGGGATTCCATTAAGTAAAGATGGATGTAAGGTACTCATTACAAGTCGATCACAAGATATATTAACCAATAATATGAATACACAGGAGTGTTTTCAGGTGAGTTCGTTATCTGAAGAAGAGTCTTGGAAGTTTTTTATGGCAATCATTGGTGATAAGTTTGATACAATTTATAAGAAAAACATTGCAAAGAATGTTGCAAAAGAATGTGGAGGGTTACCGCTTGCACTTGATACCATTGCAAAAGCATTGAAGGGGAAAGATATGCACCATTGGGAGGATGCTTTAACCAAATTGAGAAATTCTATTGGAATGGATATTAAAGGGGTGAGTGACAAAGTTTATGCTTCACTTAGATTGAGTTATGATCATCTAGATGGAGAAGAAACAAAATTAATATTTCTTCTTTGCAGCGTATTTCCAGATGATTATAAGATTTCTATAAAAAATTTGCAAATGTATGCCATGTGTATGAGATTATTGAATAAAGTAAAAACTTGGGAGGATTCAAAAAATAGGGTCATGAAGTTGGTTAATGATCTAATATCGTCTTCTTTACTTCTCGAGGCTGAGAGCGATTCAAAAGACAAGTATGTTAAAATGCACGATGTGGTTCGTGATGTTGCGATACACATTGCATCCAAGGAAGGTAACATGTCTACATTGAACATTGGATATAATAAAGTTAATGAATGGGAAGATGAATGCAGAAGTGGTTCTCATCGTGCCATTTTTGCAAACTGTGATAACTTAAACAATCTTCCCCTAAAGATGAATTTTCCACAACTTGAGTTGTTGATATTAAGAGTTTCTTATTGGTTGGTGGAAGATAATCTTCAAATTCCATATGCATTTTTTGATGGAATGGTAAAGCTCAAGGTTTTGGACTTGACAGGAATGTGTTGCCTCAGACCATTGTGGACAACACCATCATTAAACAACCTTCAAGCATTGTGTATGTTGCGTTGCGAATTTAACGACATTGATACAATCGGAGAGCTAAAGAAACTGGAAGTTTTGAGAATCGTTAAGTGTAACATGCTAGATCACTTACCTCCAACTATGAGTCAATTGACACACCTTAAGGTACTAGAAGTTTTAAATTGCCCTAAATTGGAGGTGGTTCCTGCAAACATTTTTTCAAGTATGACAAAACTCGAAGAATTGAAATTACAAGACAGCTTTTGTAGATGGGGAGAAGAAGTATGGTACAAGGATCGATTGGTCAAGAATGTCACAGTTTCAGAATTGAATTGTCTGCCATGTCTATCTAATTTAAGTTTAGAAAGTTGGAATGTTAAGATTCTATCTGAAATAAGTTCACAAACTTGTAAGAAGTTAAAAGAATTTTGGATTTGTAGTAATGAATCAGATGATTTTATTCAACCCAAGGTTTCTAATGAATATGCAAGAACCTTGATGCTTAACATTGAATCCCAAGTTGGTTCAATTGATGAAGGACTTGAAATACTATTGCAAAGAAGTGAGAGATTGATTGTAAGTGATTCAAAGGGTAATTTTATAAATGCAATGTTCAAGCCAAATGGAAATGGCTATCCCTGTTTGAAGTATCTATGGATGATTGATGAAAATGGTAATTCAGAAATGGCACATTTAATTGGAAGTGACTTTACTTCTCTAAAGTATTTGATTATTTTTGGGATGAAGAGATTGGAGAACATTGTTCCTAGGCATATTTCACTAAGCCCTTTCAAGAAGGTTAAAACTATTGCAATTCAATTTTGTGGGCAGATAAGGAATCTTTTCTCATTCTCTATTTTTAAAGACCTTTTAGATCTTCAAGAGATTGAGGTGATTAATTGTGGTAAGATGGAAGGGATTATATTCATGGAAATTGGAGATCAACTCAACATTTGCTCTTGTCCTTTAACTTCTTTACAACTTGAAAATGTGGATAAACTTACAAGTTTTTGCACCAAAGACTTAATCCAAGAAAGTTCACAAAGTATCATTCCCTTTTTTGATGGTCAGGTTTCATTTCCTGAGTTGAATGATTTATCAATTGTTGGAGGTAACAATTTGGAGACGTTATGGCATAAAAATAATAACCCAACTACAGGTTCCTTTTGCAAACTCCAATCAATAAGAATTGAACAATGCACTCAATTAAGATGCATGTTTCCTTCAAACATGTTGACATCACTTGCTTCGTTACATACAATACAAATCATTTCTTGCGCGTCATTAAAAAGGATATTTGAAATTGAAAACCAAAGTTTTAATGACACAACAGTTTTGTGGTCATTGAATGAGTTGCATTTACTCAATCTACCAAATCTCAAACACGTATGGAGAAAAGACATCATCAAAATTTTGACATTTCCATCTCTAAAGAGAGTAAAAATCCATGGCTGTACTAAGCTAACACACGTCTGGAAGGACAACAATAAAGTAACCAGAAGCTTTGATAGCTTGGAGAGGATTGAAGTAGAAAAATGCAAGAATTTGAAGTATTTACTGCCATCATCAATTGCATTCTTAAACCTGAAGGAGCTTCACATCAAGAAATGTAATGGAATGATCAATTTGTTCAGCTCTACAGTGACAAAAAAGCTAGTGAATCTCAGCTCCATTAAAGTATCTTATTGTAAAGGAATGAGATGCATGGTTGAAGTAGATCAAGCAGAAAATGATGAAATTATTACTTTCAAGAAATTGAGTACGTTGGAATTAGATTATTTACCACGATTGGATAGCTTTTACTCTGGCAAATGCATGCTTGAGTTTCCCTGTTTGGAGAGTTTGGTTATAAAAAGATGTCCTGAAATGAAGACATTTTCGTATGGAGTAATAATCGCGCCAAGATTACAAACCTTGTGGATGAACGATAAAGAATTTGGAGTATCATCACCAGCATGTGGGATAAATGAAACCATACAAAATTTTCCGAGGCGAGTGGTATGTATGTTCAATTCTAATTAA

>Cucsa.088220

ATGGGGGGTGTTGGTAAAACAATGTTAGTGAAAGAAATTTTAAGAAAAATTGTTGAGAGTAAGTCTTTCGATGAGGTGGTAACATCCACGATCAGCCAAACACCAGATTTTAAAAGTATTCAAGGACAACTAGCTGACAAGCTAGGTTTGAAATTCGAACGAGAAACAATAGAAGGAAGGGCACCTAGTCTACGAAAGAGGTTGAAGATGGAGAGACGTATCCTAGTTGTGTTGGATGATATCTGGGAGTATATTGATTTGGAAACAATAGGAATTCCAAGTGTTGAAGATCATACAGGATGCAAGATATTGTTTACCTCTAGGAATAAACATTTGATCTCAAATCAAATGTGCGCCAATCAAATTTTTGAGATAAAAGTTTTAGGAGAAAATGAGTCATGGAATTTATTTAAGGCAATGGCTGGTAAAATTGTTGAAGCAAGTGATTTGAAGCCTATAGCCATTCAAGTTGTGAGAGAATGTGCAGGTTTGCCTATTGCTATTACTACTGTTGCTAAGGCATTACGAAATAAACCTTCCGACATTTGGAATGATGCCTTGGATCAACTTAAAAGTGTTGATGTGTTTATGACAAACATTGGAGAAATGGACAAGAAAGTGTATTTGTCACTAAAATTGAGTTACGATTGCTTGGGATATGAAGAGGTCAAGTTATTATTCTTGTTATGCAGCATGTTTCCAGAAGACTTTAGCATTGACATGGAAGAGTTGCATGTATATGCCATGGGCATGGGTTTCTTACATGGTGTTGATACTGTGGTAAAAGGACGACGTAGGATTAAAAAATTGGTTGACGATCTTATATCTTCTTCTTTGCTTCAACAATATTCTGAGTATGGGTACAATTATGTGAAAATGCATGATATGGTTCGTGATGTAGCTATATTTATTGCGTCTAAGAATGATCACATACGTACATTGAGCTATGTGAAAAGATTAGATGAAGAATGGAAAGAAGAGAGACTATTGGGTAATCATACCGTGGTGTCCATTCATGGTTTACATTATCCTCTCCCAAAGTTAATGTTACCCAAAGTTCAATTATTAAGGTTGGATGGACAATGGTTGAATAATACGTATGTGTCAGTGGTACAAACTTTTTTTGAAGAAATGAAAGAGCTCAAAGGTTTAGTATTAGAAAAAATGAATATATCCTTGTTGCAACGACCATTTGATCTTTACTTCTTAGCAAACATTAGAGTGTTACGTTTACGGGGATGTGAATTAGGGAGCATAGATATGATTGGTGAATTAAAAAGGCTTGAAATTCTCGATCTTAGTGGATCTAACATCATCCAGATTCCTACAACAATGGGTCAATTGACACAACTGAAAGTGTTAAATTTATCTAATTGTTTTAATAAGCTCGAGATAATTCCACCAAATATTCTTTCAAAGTTGACAAAACTGGAGGAATTACGTATGGGAACTTTTGGTAGTTGGGAAGGAGAAGAATGGTATGAAGGAAGGAAAAATGCTAGTCTTTCCGAGCTTAGGTTCTTGCCACACCTTTTTGATTTAGATTTAACCATTCAAGATGAAAAGATTATGCCAAAACACTTGTTTTCAGCAGAGGAGTTGAATCTTGAAAAATTCCACATTACTATTGGTTGTAAGAGAGAAAGAGTTAAAAATTATGATGGAATCATTAAGATGAACTACTCTAGAATATTGGAAGTCAAGATGGAATCAGAAATGTGCTTGGATGATTGGATAAAATTTTTGTTAAAGAGGTCAGAAGAAGTGCATTTAGAAGGATCAATTTGTTCAAAGGTTCTTAACTCTGAACTGTTAGATGCAAATGGCTTCTTACATTTGAAGAATCTCTGGATTTTTTATAATTCAGACATTCAACATTTCATCCACGAAAAGAACAAGCCTTTGCGAAAATGCTTGTCCAAATTGGAGTTCTTATATCTTAAGAACTTGGAGAATTTGGAAAGTGTAATTCATGGTTATAATCATGGTGAATCTCCTTTAAACAATTTGAAGAATGTAATCGTATGGAATTGCAATAAATTGAAAACCCTATTTTTGAATTGCATGTTGGATGACGTTTTGAATCTCGAGGAAATTGAGATCAATTATTGTAAAAAGATGGAAGTGATGATCACTGTGAAGGAAAATGAGGAGACAACCAACCACGTTGAGTTTACTCATTTGAAATCTTTATGTCTATGGACTTTACCACAACTTCATAAATTTTGCTCCAAAGTATCGCTTCCTAATTTGGAGAAGTTGAAAATTTGGTGTACAAAGGATTTGAAGAAGATATGGAGCAATAATGTACTCATTCCCAATTCCTTTTCCAAACTTAAGGAAATAGACATTTATTCATGCAACAATCTTCAAAAAGCATTGTTCTCTCCAAATATGATGAGCATTCTTACTTGCCTTAAAGTCCTAAGGATTGAAGATTGTAAATTGTTGGAAGGAATATTTGAAGTGCAAGAGCCAATTAGTGTTGTTGAAGCAAGTCCTATCGCCCTCCAAACTTTGAGTGAGTTGAAACTATATAAACTTCCAAACCTTGAGTACGTATGGAGCAAAGATTCCTGTGAGCTTCAGAGTTTGGTAAATATAAAACGTTTAACCATGGATGAATGTCCAAGACTTAGAAGAGAATATTCAGTCAAAATTCTCAAGCAACTTGAAGCACTAAGCATAGATATCAAACAATTGATGGAGGTTATTGGGAAGAAAAAGTCGACGGATTATAACAGAATCTTAATTAACAAATTAGTAATTGGTCAGGTTGAGGTTCTACAGTTGGGAGATGGTTCTGAGTTGTTTCCGAAGCTTAAAACTTTGAAGCTATATGGTTTTGTTGAGGATAACTCAACCCATCTGCCAATGGAAATTGTACAAAACTTATACCAATTTGAGAAGTTTGAATTAGAAGGAGCATTTATTGAAGAAATTCTCCCCAGCAACATATTGATTCCTATGAAAAAACAATACAATGCAAGACGATCTAAAACTTCACAGCGTAGTTGGGTTCTATCCAAGCTACCGAAACTTAGGCATTTGGGGAGTGAATGCTCACAAAAGAATAATGATTCAATTCTACAAGATCTGACCTCTCTGTCCATTTCAGAATGTGGTGGATTGAGTAGTTTAGTGTCATCATCGGTGTCTTTTACGAACTTGACGTTTCTTAAATTGAATAAATGTGATGGACTAACCCATTTGCTGGATCCTTCGATGGCTACAACGCTTGTGCAACTTAAACAGTTGAGAATAGGAGAATGCAAAAGGATGAGTCGTATAATTGAGGGAGGATCATCAGGTGAAGAAGATGGAAATGGTGAAATTATTGTATTCAACAACCTACAACTTTTAATCATTACTTCTTGTTCCAACCTAACAAGCTTTTATCGTGGAAGATGCATCATTCAATTTCCATGTTTGAAACATGTATCTCTTGAGAAGTGTCCTAAAATGAAGTCATTTTCATTTGGAATTGTAAGTACATCTCATTCAAAATATGAAAATGTTTCTTTAAAGAATGATGATGATGATACTCACTATCGTCCAAAAGAATCCAAAGAGAGATAG

>Cucsa.089350

ATGTCTGATAAAAAAATTGTTATGCTTGGATTATATGGAATTGGAGGTATCGGCAAGACAACTTTGGCCAAAGCATTGTACAATAGAATTGCTCATGACTTTGAAGGTTGTTGCTTTTTGGAAAAAATTAGAGAAGCTTCAAATCAATATGACGGCCTTGTTCAACTCCAAAAGAAAATACTTTGTGATATTCTAATGGATAATTCGATCAATGTTAGCAATCTTGATATAGGGGTTAACATCATAAGGAATCGACTATGCTCAAAAAAAATTCTTTTAATTCTTGATGATGTTGATACGAGAGAACAACTAGAAGCATTAGCGGGAGGGCATGATTGGTTTGGACATGGAAGTAAGATCATTGCGACAACAAGAAACATGCAATTACTTGCTAGTCATGGATTTAATAAATTGGAAAAAGTTAACGGATTGAATGCCATTGAAGGTCTTGAGCTTTTTAGTTGGCATGCATTCAACAATTGTCATCCCTCAAGTGATTATTTAGACCTTTCAAAACGTGCTGTACATTATTGTAAAGATCTTCCATTAGCTCTTGAAGTGTTAGGTTCCTTCCTTAATTCTATTCACGATCAATCCAAATTTGAACGTATATTGGACGAGTATAAGAACTTCTATCTAGACAAAGACATCCAAGATATTCTTCGAATAAGTTATGATGAACTTGAACAAGATGTAAAAGATATTTTCCTTTACATTTCTTGTTGCTTTGTAGGAGAAGATATCAACGAAGTTAAAATGAAGTTAGAAGCATGTGGTTGTTTATGTTTGGAAAAGGGAACAACAAAACTAATGAATTTATCACTTCTAACCATTGAATCCAATCGAATTAAAATGCATGACTTAATACAACAAATGGGTCGCTCAATTCATCTCTCGAAAACTTTTACATCTCATAAAAGAAAAAGATTGTTGATTAAAGATGATGCTATGGATGTCTTAAATGGGAATAAGTGTGATTTAACTACTGCAATAAACCTCGAAAAGTTGAATCTTGAAGGGTGTGAAAAATTAGTAAAGGTTCATGAATCAGTTGGATCTCTCAGTAAGCTTGTTGAGTTTTATCTTTCCAGCAGTGTTGAGGGTTTTGAGAAGTTTCCATCCTGCCTTAAGTTGAATTCTCTTGAAGCTTTGGTAGTGAGATATTGTAGAATAGAAGAATGTTGTCCTCAATTTAGTGAAGAAATGAATAGCCTAGAAATATTGGAGATCGATGATAGTATAATTAATCAATTATCTCCAACAATTGAATATCTTACTGGCCTAAAAGAACTCTGGATCACAGAGTGCACGAAGCTCGAAACTCTTCCAAGTACAATTTATCGTTTAAGTAATCTTACTTCTTTAGAAGTCAAAAAATCTGATCTTTCAATCTTTCCTTCCTTAAATGATCCTTCTTCATCTTCCTTATTAATTCCCTACCTAACATCAATAAAGCTTTTCAATTGTCAGATAACAAATTTGGATTTCTTAGAAACAATGGTTCACGTTACCCCTTCATTGGAAATGTTGGACTTATCTCAAAACAACTTTTGTGGACTACCCTCCTGTATTATTAATTTTAAATCCTTGAAATATCTTTATATAATCGAATGTAAGTCGCTTGAAGAAATTCTAAAGGTTCCAAAAGGAGTAGTTCGTATGGATACTAGAGGGTGTGTATCATTGGCCAAATTTCCTAACAACATTCCTGATTTCATATCTTGTGATGATAATGTGGAATATGATACAAAAGACGGAGTAATCAAACAACTCATATTAATGAATTGTGATATTCCAGATTGGTGCAATCGAATGTCAATCTCCAATTTTAACTTTTGGCTATCACGAGGTGAATGTCTATGGATGGCAGTACTTCATCCTTGCATGCATCGTCTAATCAACCCATATGGTGATGATATTATGGACATCTCACCGAATTTCTCAATAGGCATTTTGGACAATAAAATTACATTGTTATTTGAGGTTAATCCAGAGTGTAAAGACACA

>Cucsa.091460

ATGGGTTCTTCTGTTGTTGGAGATGAATCATTTTCTTCTTCTCCCAATTTCAATTACGATTATGATGTGTTTTTTAGTTTCAGAGGAGAAGATACTCGCTCCAATTTTATCAGTCATCTTCATATGGCCTTGCGTCTAAAGGAAGTCAACGTTTTCATAGACGACAAACTCAAAAGGGGTGAACAAATTTATGAGTCTCTTCTCAAATTTATAGAGCGATCTAGACTTTCCCTCGTTATTTTCTCTAAAGATTATGCATCTTCAACTTGGTGTTTGGATGAACTGGTGAAAATAATTGAGTGTAAGAAATCCAAAGGACAAGCAGTTTGGCCAGTGTTCTACAAGGTGGATCCATCCGAGGTTCGAAAACAAACCGGTGGGTTTGGGGAAGCATTGGCCAAACATGAAGCTAATAAGTTATTGGCCAACAAGATTCAACCATGGAGGGAAGCTTTGACTTTTGCTGCTGGTTTGTCTGGTTGGGATCTAGCAAATAGCAAGGATGAGGCTGAACTTATCCAAAAAATTGTTAAACGAGTGTTGTCTGCAGTAAATCCAATGCAATTACTACATGTAGCCAAGCACCAAGTAGGAGTTGATTCTCGACTAAGGAAAATTGAGGAGTTGGTCTCTCATATTGGGTCCGAGGGTGTTAATCTGGTGGGGTTGTATGGCATTGGAGGCATTGGTAAGACCACTTTGGCTAAGGCTTTGTACAACAAAATTGCTACCCAATTTGAAGGATGCTGCTTTCTACAAGATGTTAGACGAGAAGCTTCGAAGCATGGGCTCGTTCAACTACAGGAAACCTTACTCAATGAGATCTTAAAAGAGGATTTGAAGGTTATTGTCAGTCGTGATAGAGGAATTAACATCATAAGAAGTAGACTGTGTTCAAAGAAAGTTCTTATAGTTCTTGATGATGTGAATGATCTTGAGCAATTAGAAGCACTGGTTGGTGGGCGTGATTGGTTTGGTCAAGGTAGTAAAATCATTGTGACGACAAGGAATGAACATTTACTTTCTAGCCATGGATTTGATGAAAAGCATAAAATTCAAGAATTGAATCAAGACCATGCTCTTGAGCTTTTCAGTTGGCATGCTTTTAAGAAAAGTCATCCATCAAGTAATTATTTAGATTTTTCAAAGCGTGCTACAAGTTATTGTAAAGGTCTCTCTTTGGCCCTCGTTGTTTTGGGTTCTTTCCTTTGCGGCAGAGCTAAAGAAGAATGGAATGGTATACTAGATGAATTTGAAAACTCTTTGAGAAAAGATATTAAAGATGTACTTCAATTAAGTTTTGATGGACTTGAAGACAAAATAAAGGATATTTTCCTCGACATTTCTTGTTTATTCGTGGGAGAAGAATACAAGTGTGCTAAAAAAATGTTGAGTGCATGTCATTTGAACATAGATTTTGGAATTATGATACTCATGGATCTTTCACTTATTACGGTTGAAATGGATAGAGTGCAAATGCATGAGTTAATACAACAAATGGGTCGTAGCATAGTTCATAATGAATCATCTGAGCCTGGAAAGAGGAGTAGGCTGTGGTTGGAACACGACATTTGGGAGGTGTTTGTTAATAATTCTGGAACAGATGCAGTTAAAGCCATAAAGTTGGACTTGCCTAAATCCACAAGGCTAAATGTAGATCCACGGGCATTTGGAAGCATGAAAAATTTGAGATTGCTTATCATTCGAAATGCACGATTTTGTACAAAGATTAGGTACTTACCTAATAGCTTAAAGTGGATTGAATGGCATGGATTTGCTCATCGAACTTTGCCGTCCTGCTTCATTACCAAAAATCTTGTTGGACTTGATTTGCAACATAGCCTCATCAAAAGATTTGGGAAAAGACTTAAGGTCTCTTCAAGAAATTCCGAACCTTCCTCAAAACATACAAATTTTGAATGCGAATGGCTGTAA

>Cucsa.091470

ATGGATTCTTCCACTGTTGCAACAGAATCACCGACTTTCAAATGGACTTATGATGTGTTTTTGAGTTTCAGAGGAGAGGATACTCGCACAAATTTCACCAGTCATCTTGATATGGCCTTGCGTCAAAAGGGTGTCAATGTCTTCATAGACAACAAGCTCGAAAGGGGTGAGCAAATTTCTGAATCCCTTTTCAAATCTATACAGGAAGCTTCCATTTCTATTGTTATATTCTCTCAAAATTATGCATCTTCTTCCTGGTGTCTGGATGAATTGGTGAACATAATTGAGTGTAAGAAATCCAAGGGCCAGAATGTTTTCCCAGTTTTCTATAAGGTGGATCCGTCCGATATACGAAAACAAACTGGTAGCTTCGGAGAAGCACTGGCCAAACATCAGCCTAAGTTCCAAACAAAGACCCAAATTTGGAGGGAAGCTTTAACTACTGCTGCTAACTTGTCTGGTTGGAATCTAGGAACTAGGAAGGAGGCTGATCTTATTGGAGATCTTGTTAAAAAAGTGTTGTCTGTATTAAACCGCACTTGCACGCCCTTATATGTAGCTAAGTATCCGGTTGGAATTGATTCTAAACTAGAATATATGAAGCTTCGTTCACATAATCTTTTTGAGAAGAGCAACAAATTCCATTATCGAAAACAACATGAGTATGAGTCTGATACTGGTGTTTACATGGTGGGCTTATATGGCATTGGAGGCATTGGTAAGACAACTTTGGCTAAAGCTTTATACAACAAAATTGCTAGCCAATTTGAAGCTTGCTGCTTTCTATCAAATGTTAGAGAAGCTTCAAAGCAATTCAATGGCCTTGCTCAACTACAGGAAACCCTACTCTATGAGATCCTAACGGTTGATTTGAAGGTTATCAACCTTGATAGAGGAATTAACATCATAAGGAATAGATTGTGTTTGAAGAAAGTCCTTATAGTTCTTGATGATGTAGATAAGCTTGAGCAGTTAGAAGCATTGGTTGGCGGGCGTGATTGGTTTGGCCAAGGCAGTAGAATCATTGTGACGACAAGGAACAAACATTTACTTTCTAGCCATGGCTTTGATGAAATGGAAAATATTCTAGGATTGGATGAAGACGAAGCTATTGAGCTTTTTAGTTGGCATGCTTTCAAGAAAAATCATCCATCAAGTAATTATTTAGACCTTTCAAAACGTGCTACAAGTTATTGTAAAGGCCATTCTTTGGCTCTCGTTGTTTTGGGTTCTTTCCTCTGTACCAGAGATCAAGTAGAATGGTGTAGTATATTAGATGAATTTGAAAACTCTTTGAACAAAGATATCAAAGATATCCTTCAATTAAGTTTTGATGGTCTGGAAGACAAAGTAAAGGATATCTTTCTTGATATTTCTTGTTTACTTGTGGGAGAGAAAGTTAAGTACGTTAAGAATATGTTGAGTGCATGTCATGTAAATCTAGATTTTGGAATTATAGTACTCACGGATCTTTCATTTATTACGATTGAAAATGACATAATGCAAATGCATGATTTAATAAAACAGATGGGTCATAAAATAGTTTGTGGTGAATCTCTTGAGCTTGGAAAGAGGAGTAGGTTGTGGTTGGTACAGGATGTTTGGGAGGTGCTTGTTAATAATTCAGGAACAGACGCAGTTAAAGGCATAAAGTTGGACTTTCCTAATTCCACGAGGCTGGATGTGGATCCACAAGCTTTTAGAAAAATGAAAAATTTGAGATTGCTTATTGTTCAAAATGCAAGATTTTCTACAAAGATTGAGTACCTACCTGATAGCTTAAAGTGGATTAAGTGGCATGGATTTCGTCAACCAACTTTTCCTTCGTTCTTCACTATGAAAAATCTTGTTGGACTAGATTTGCAACATAGCTTCATCAAAACATTTGGGAAAAGACTTGAGGATTGTGAAAGGTTGAAGTATGTTGATCTTAGCTACTCTACTTTCTTAGAGAAAATTCCTAATTTCTCTGCAGCATCAAACCTTGAAGAGTTGTATCTCACCAATTGCACAAATTTAGGAATGATAGATAAGTCTGTTTTCTCTCTCGATAAGCTTACTGTCCTAAACCTTGATGGTTGTTCTAACCTTAAAAAGCTTCCAAGAGGCTACTTCATGTTAAGTTCTCTTAAAAAATTGAATCTCTCTTACTGCAAAAAACTTGAGAAAATTCCAGACTTATCTTCAGCATCAAACCTTACGAGCTTGCATATCTACGAATGCACAAATTTAAGAGTAATTCATGAATCTGTTGGATCTTTGGATAAGCTTGAAGGTTTGTACCTTAAACAATGCACTAACCTGGTAAAGCTTCCAAGCTATCTCAGCTTAAAGTCTCTTCTATGTTTATCACTTTCTGGGTGTTGTAAGCTTGAAAGCTTCCCAACAATTGCTAAAAACATGAAATCTTTAAGGACCTTGGATTTGGATTTTACTGCCATAAAGGAGTTACCTTCATCAATTAGATATCTCACTGAGCTTTGGACATTAAAACTTAATGGTTGCACAAACCTCATCTCCCTTCCCAATACAATTTATTTGTTAAGGAGTCTTGAGAATCTTCTTCTTAGTGGCTGTTCTATATTTGGAATTTTACCCTCATGTCTCCACAAGTTCATGTCGTTGTGGAATCTTGAATTAAGGAATTGTAAGTTTCTTCAAGAAATTCCAAGCCTTCCTGAGAGTATACAAAAAATGGATGCCTGTGGTTGTGAATCATTGTCTCGAATTCCAGATAACATTGTGGATATAATATCAAAAAAACAGGACCTCACAATGGGTGAGATTTCAAGAGAGTTTTTATTAACGGGGATTGAGATTCCAGAATGGTTCAGCTATAAGACTACATCCAATTTGGTGAGTGCTAGCTTTCGTCACTATCCAGACATGGAAAGAACTTTGGCTGCCTGTGTTAGTTTCAAAGTAAATGGAAATTCATCTGAAAGAGGTGCCCGGATTTCATGCAATATATTCGTCTGCAATAGACTCTATTTTTCATTGTCAAGACCATTTCTTCCCTCAAAATCAGAATATATGTGGTTAGTAACAACTTCTCTAGCGTTGGGTTCCATGGAGGTGAATGACTGGAATAAAGTTTTGGTCTGGTTTGAGGTTCATGAAGCACATAGTGAGGTTAATGCAACTATAACAAGGTATGGTGTCCATGTCACTGAAGAGCTCCATGCGATACAAACGGATGTCAAGTGGCCGATGGTAAATTATGCTGATTTTTATCAACTGGAGAAATTGCAAAGTCTGGATATTGAGGAACTTCTTCTCAAACGCCTTTTTGAAGAAATGTCGTGCTGGTCCAATTCCCAAGCAATGTTATATGCGGCAAATTATGATCCAGAAGCAATAATCGATTCGAATATACAACCTATGATATTTCCATTGCACGTAACATATAATGGTGAGACATTTATATGTGGAATGGAAGGCATGGGAGACACTACACTCGCCAACTCTTTATGCAATAAATTTAATAGGTCAAATGACAACGGTTGGCCAAGAGAAGCTTTAGATGATTCTACAAGCTTTTTGCATTTTCGAGGAGGAAAGTTTTATGGAGGTTCCTGGTCATTGTCCCACCACCGTAAGCGTGGAGATGGTGAAAGAGGAACCAATATCACAACCCGCACAATATCCTCCAAACGCTATTTGATACTCTTTCATAAAGCGGGGAGCTATAATCATTTATTTAACTTTGCTGGTAGCCACCGTTTGATTGCAGGTTCTGGCAGTTATGACAGTCTTAACGGAAGAGGTGATGTTCGGCTTCTGATTGAAAGGGTTGATACATCCTTGCTCTGA

>Cucsa.091680

ATGCAGTTGGATGTAGCTAAATATCCAGTTGGAATTGACATACAAGTTAGAAATTTACTCCCACATGTTATGTCTAATGGAATTACCATGTTTGGATTATATGGAGTTGGAGGTATGGGCAAGACAACTATAGCGAAAGCTTTATACAATAAAATTGCTGATGAGTTTGAAGGTTGTTGCTTTCTGTCAAATATTAGAGAAGCTTCGAATCAATATGGAGGCCTTGTTCAATTTCAAAAGGAGCTACTTTGTGAGATTCTAATGGATGATTCGATTAAAGTTAGCAATCTTCCTAGAGGAATTACTATCATAAGGAATCGACTATACTCAAAAAAGATTCTTTTGATTCTTGATGATGTTGATACGCGTGAACAACTACAGGCATTGGCGGGAGGACATGATTGGTTTGGACATGGAAGTAAGGTGATTGCGACAACAAGAAACAAGCAATTACTTGTTACTCATGGATTTGATAAAATGCAAAATGTTGGGGGATTGGATTACGATGAAGCTCTTGAGCTCTTTAGTTGGCATTGTTTTAGGAATAGTCATCCCTTAAATGTTTATTTAGAACTTTCAAAACGTGCCGTAGATTATTGTAAAGGCCTTCCCTTAGCTCTTGAAGTTTTAGGTTCCTTCCTTCATTCTATTGGTGATCCCTCCAATTTTAAACGTATATTGGATGAATATGAAAAACATTACCTCGATAAGGACATCCAAGACTCTCTTCGAATAAGTTACGATGGACTCGAAGATGAAGTAAAAGAAATATTTTGCTATATTTCTTGTTGCTTTGAAGCAAGAGCAGTGAAAGTCATAAAACTAAATTTTCCTAAACCTACGAAGTTGGACATCGATTCAAGAGCTTTTGATAAAGTGAAAAATTTGGTAGTACTGGAAGTTGGCAATGCCACATCTTCAGAAAGTTCTACTCTTGAGTATCTACCCAGTAGCTTAAGGTGGATGAATTGGCCTCAATTTCCTTTTTCATCTTTGCCTACAACCTACACAATGGAGAACCTTATTGAATTGAAATTGCCATATAGCTCCATCAAACATTTTGGTCAAGGATATATGAGTTGTGAAAGGTTGAAGGAAATTAATCTTAGTGACTCCAATCTTTTGGTGGAAATCCCAGATTTATCTACTGCAATAAACCTCAAATACTTGAATCTTGTAGGATGTGAAAATTTAGTAAAAGTTCATGAATCAATTGGATCTCTCAAGCTCACAACTCTTCCAAGTACAATTTATCGTTTAACCAATCTTACTTCTTTAACTGTGTTGGATTCTAATCTTTCAACATTTCCTTTCTTAAATCATCCGTCCTTACCTTCCTCACTTTTTTACCTAACCAAGTTACGTATTGTTGGTTGCAAGATAACAAATTTGGATTTCTTAGAAACAATTGTTTATGTCGCCCCTTCATTGAAAGAGTTGGACTTGTCCGAAAACAACTTTTGTAGATTACCGTCATGTATTATTAATTTTAAATCTCTAAAATATCTTTATACGATGGATTGTGAGTTGCTTGAAGAAATTTCAAAGGTTCCCGAAGGCGTAATTTGTATGAGTGCTGCTGGGAGCATATCATTGGCTAGATTTCCTAACAACTTGGCTGATTTCATGTCTTGTGATGATTCTGTGGAATATTGTAAGGGTGGAGAATTGAAACAACTGGTATTAATGAATTGTCATATTCCAGATTGGTATAGGTACAAGAGCATGAGCGATTCATTAACATTTTTTTTGCCAGCTGATTATCTAAGTTGGAAATGGAAGCCTTTGTTTGCTCCTTGTGTCAAATTTGAAGTTACGAATGATGATTGGTTCCAGAAGCTTGAATGTAAAGTTTTTATCAACGATATTCAAGTATGGAGTTCTGAAGAGGTGTATGCCAATCAGAAGGAACGGAGTGGGATGTTTGGAAAAGTATCACCAGGTGAGTATATGTGGCTGATAGTACTTGATCCTCATACACGTTTCCAATCATATTCGGATGATATCATGGACAGGAGGTCACTGAAGATTATTGATCTAAATCAACTAAGTTCTGAGATTAATTCCTCACAAAGTATTTTGGGTAAAATTACGGTGTCATTTGAGGTTACTCCATG

>Cucsa.091690

ATGTCTAATGAAATTACTATGGTTGGATTGTATGGAATTGGAGGTATGGGCAAGACAACTTTGGCCAAAGCTTTATACAATAAAATTTCTGATGACTTTGAAGGTTGTTGCTTTTTGGCAAATGTTAGAGAAGCTTCAAATCAATACTGGGGTCTTGTTGAACTCCAAAAGACGCTAATTCGTGAGATTCTGATGGATGATTCAATCAAAGTTAGCAATGTTGGTATAGGAATTAGCATCATAAGGGATCGATTATGCTCAAAAAAGATAATTTTGATTCTTGATGACATTGATACACATGAACAACTACAGGCATTGGCTGGAGGACATGATTGGTTTGGACATGGAAGTAAGGTCATTGCAACAACAAGAAACAAGCAATTACTTGCTAGTCATGGGTTTAATATATTGAAAAGAGTTAACGGATTAAATGCGATTGAAGGTCTTGAGCTTTTTAGTTGGCATGCATTTAAAAATAGTCATCCCTCAAGTGATTATTTAGATGTTTCAAAACGTGCTGTGCATTATTGTAAAGGTCTTCCCTTAGCACTTGAAGTGTTAGGTTCCTTCCTTAATTCTATCGATGATCAATCCAAGTTTGAACGTATATTGGATGAATATGAGAACTCATACCTGGACAAAGGCATCCAAGATATTCTTCGAATAAGTTATGATGAGCTTGAACAAGATGTAAAAGAAATTTTCCTTTACATTTCTTGTTGCTTTGTACATGAAGACAAAAACGAGGTTCAAATGATGTTAAAAGAATGTGACTCTCGTTTCAGATTGGAAATGGGTATTAAAAAACTCACTGATCTATCACTTCTTACAATTGATAAATTCAACCGGGTCGAAATGCATGACTTGATACAACAAATGGGTCACACAATTCATCTCTTGGAGACTTCTAATTCTCATAAAAGAAAAAGATTGTTGTTTGAAAAAGACGTCATGGATGTCTTAAATGGAGATATGAATTGCAAATGGTTGAAGCGTATAAATCTTAACTACTCAAAGTTTTTAGAAGAAATTTCTGATCTATCCAGTGCGATAAATCTTGAAGAGTTGAATCTTTCCGAGTGTAAAAAGCTGGTAAGAGTTCATGAATCAGTTGGATCACTAGGTAAACTTGCTAAATTGGAACTTTCTAGTCATCCTAATGGCTTTACGCAATTTCCATCCAACCTCAAGTTGAAGTCCCTACAAAAATTGGTAATGTACGAGTGCAGGATTGTTGAAAGTTATCCTCATTTCAGTGAAGAAATGAAGTCTAGTTTAAAAGAATTACGGATTCAGTCTTGTAGTGTGACAAAACTATCCCCAACGATTGGAAATCTTACTGGTCTTCAACATTTGTGGATCGATGTATGCAAAGAGCTCACTACTCTTCCAAGTACCATTTGTCATTTAAGCAATCTTATTTCTTTAAGTGTTTTCAGATCTGAAGTTTCAACTTTTTCGTTCTTATATTCTCGTTCCCTTTCCTTATTTCCCTACCTAACACTTTTAAAACTTTGTTACTGCAAGATAACAAATTTGAGTTTCTTAGAAACAATCACCCATGTCGCCCCTTCATTGACACAGTTGTACTTGACTGGAAACGACTTTTGTAGCCTACCCTCATGTATTGTTAATTTTAAATCTTTGAGATATTTTGATATAAGTTATTGTGGATCATTGGCTAGATTTCCAGACAATATAGCTGAATTCATATCTTGTGATTCGGAATATGTAGATGGAAAATACAAACAACTCATATTAATGAATAATTGTGATATTCCAGAATGGTTTCATTTCAAGAGTACGAACAATTCAATAACGTTTCCTACGACATTTAATTATCCGGGTTGGAAATTGAAAGTTCTTGCTGCTTGTGTTAAAGTTCAAGTTCATGATCCTGTTAATGGGTATCATAGAGGGGGGGATCTTGAATGTGAAGTGTTCTTTAAGGACATTCTAGTATGGAGTTCTGGAGACTGGACAAATTATCTTGGATACGATTCAAGATGGTTGCCCCTAGGAGCATCACCAAGTGAGTATACATGGTTTATTGTACTCAATCCTCATAGAGATTTCTCCCTAGATGATTGGGATGATATGATGGAGAGATCACCAGAGACTGATCTAAGTCAGCTATGTTTTGGAATTAATTCCATGGAAATGGACCGTAATAGATCAAATGATAAATGGAATTCTATTGGGGGAAGTATTTGGAAGAACTTTACGGTGTTGTTTGAGCCTCGTCCCCTGTCTCCAGACACTACAATAA

>Cucsa.091710

ATGGTTGGATTGCATGGAATTGGAGGTATGGGCAAGACAACTTTGGCCAAAACATTATACAATCGGATTGCTGATGACTTTGAAGGCTGTTGTTTTTTAGCAAATATTAGAGAAGCTTCAAAGCAACACGAGGGACTTGTTCGACTCCAAGAGAAACTACTTTATGAGATTTTAATGGATGACTTTATTAGAGTTAGTGATCTTTACAAAGGAATCAACATCATAAGGAATCGACTATGCTCCAAAAAGATTCTCTTAATTCTTGATGATATAGATACCAGTGAACAACTACAGGTATTAGCTGGAGGATACGATTGGTTTGGATATGGAAGTAAGGTCATTGTGACAACAAGAAACGAACACTTACTTGATATCCATGGATTTAATAAATTGCGAAGTGTTCCTGAATTGAATTATGGTGAAGCTCTTGAGCTTTTTAGCTGGCATGCTTTTCAGTGTAGTAGTCCACCAACCGAGTATTTACAACTTTCAAAAGATGCTGTAAATTATTGTAAAAATCTTCCCTTGGCGCTTGAAGTTTTAGGTTCATTCCTTTATTCAACTGATCAATCCAAATTTAAAGGTATATTGGAGGAATTTGCAATCTCCAACCTTGACAAAGACATCCAAAATCTTCTTCAAGTAAGTTACGATGAACTTGAAGGTGATGTACAAGAAATGTTCTTGTTTATTTCTTGTTTCTTTGTGGGAGAAGATAAAACCATGGTTGAAACGATGTTGAAGAGTTGTGGTTGTTTATGTTGGGAAAAGGGAATTCAAAAACTCATGAATCTATCACTTCTTACTATTAACCAATGGAACAAAGTTGAAATGCATGACTTAATACAACAATTGGGTCACACAATCGCACGTTCAAAGACTTCTATATCTCCTTCAGAAAAAAAATTATTGGTTGGAGATGATGCTATGCATGTGCTAGATGGCATTAAGCATTGTGAAAGGTTGAAGCAACTTGATCTTAGCAACTCCTTCTTTTTGGAGGAAATTCCTGATTTATCTGCGGCAATAAACCTCGAAAATTTGTCTCTTTCTGGATGTATAAGTTTAGTAAAGGTTCATAAATCAGTTGGATCTCTTCCTAAACTTATTGATTTGAGTCTTTCAAGCCATCTATCTTCAACAATTAGATATCTTACCAGCCTCAAAGATTTGACCATCGTGGATTGCAAAAAGCTCACTACTCTTCCAAGTACAATTTATGACTTGAGCAAACTTACATCCATAGAAGTCTCACAATCCGATCTTTCAACATTTCCTTCCTCATATTCCTGCCCTTCCTCACTTCCCCTCCTAACAAGATTACACCTTTATGAGAACAAGATAACAAATTTAGATTTTTTGGAAACTATCGCTCATGCTGCTCCATCACTGAGAGAGTTGAACTTGTCTAACAACAACTTTTCTATACTACCTTCATGTATTGTTAATTTTAAATCCTTGAGATTTCTTGAAACATTTGATTGTAAGTTTCTGGAAGAAATTCCAAAGATTCCAGAAGGCTTAATTTCTTTGGTTAATCATGTTGACCGGAATCGTTATATGGATCTTGAATGTAAAATGTTCATAAACGACATTCAAATATTGTCAAGAGATCACAGGAGTACTTTAATCTACATCAGCCAAGTTTTGGGATCAATTCCATGGGAAGGGACAATAATAATTGTAATTTTTGAACATCGAATATTATCAACTTATAAATTTTTTAGCTTCTTACCTATATCAAGCCCAAAA

>Cucsa.091780

ATGGCCAAACATCAGCCTAAGTTCCAAACAAAGACCCAAATTTGGAGGAAAGCTTTAACTACTGCTGCTAACTTGTCTGGTTGGGATCTAGGAGCTTATAGGAGGGAGGCTGATCTTATTCGGGATCTTGTTAAGGAAGTGTTATCTACAATAAATCGCACTCGCACACCCTTATATGTCGCCAAGTATCCAGTTGGAATTGATTCTCAACTAGAATACATGAAGTTTCACTCACATCATCTCAACAAGGGAAACAAATTCCAATATTGGACACAAAATGAGTATGAGTCTGATATTGGTGTTTACATGGTGGGGATATATGGCATTGGAGGCCTTGGTAAGACAACTTTGGCTAAAGCTCTATACAATAAAATAGCTAGCCAATTTGAAGGGTGCTGTTTTCTATCAAATGTTCGACAAGCTTCAAACCAATTCAATGGCCTTGTTCAACTACAGCAAAACCTACTCTATGAAATCTTAGAGGATGATTTGAAGTTTGTCAATCTTGATAAAGGAATTACCATCATAAGGAATAGACTGCGTTCAAAGAAAGTTTTGATAGTTCTTGATGATGTGGATAAGCTCGAACAACTAGAAGCATTGGTTGGTGGACGTGATTGGTTTGGTCAAGGTAGTAAAATCATAGTGACGACGAGGAATAGTCATTTACTTTCTAGCCATGGATTTGATGAAATGCACAATATTCAAGGATTGAATCAAGACAGAGCTATTGAGCTTTTTAGTTGGCATGCTTTTAAGGAAAGTCATCCATCAAGTAATTATTTAGACCTTGCCGAACGTGCTACAAGTTATTGTAAAGGTCATCCTTTGGCTCTTGTTGTTCTGGGTTCTTTCCTTTGTAATAGAGGTCAAACAGAATGGAGAAGTATATTGGATAAATTTGAAAACTCTTTGAACAATGATATTAAAGATATTCTTCAATTAAGTTTTGATGGGCTGGAAGGTGGAGTAAAGGATATTTTTCTTGATATTTCTTGTTTATTTGTAGGGGAAAAATACAATAATTGTGCTAAAAAAATGTTGAGTGCATGCCATTTGAACGTAGATTTTGGAATTATGATACTCATGGATCTTTCACTTGTTACGATTGAAAAGGATAGAGTGCAAATGCACGGATTAATACAACAGATGGGTCATAGCATAGTTCATAATGAATCATTTGAGTCAGGAAAGAGGAGTAGATTGTGGTCGGAGCGGGACATTTGGAACGTGTTTGTTAATAATTCGGATTGTGAAAGGTTGAAGCATGTTGATCTTAGCTACTCTACTTTATTAGAGAAAATTCCTGATTTATCCGCTGCATCAAACCTTGAAGAATTGTATCTCATCAATTGCACAAATTTAGGAATGATAGATAAGTCTGTTTTCTCTCTCAATAAGCTTACTGTCCTAAACTTTAAAGGTTGTTCTAACCTTAAAAAGCTTCCAAAAGGCTACTTCATGTTCAGTTCTCTTAAAATATTGAATCTCTCTTACTGCCAAGAACTTGAGAAAATTCCAGACTTATCTTCAGCATCAAACCTTCAGAGCTTGCTACTCAACGGATGCACAAATTTAAGAGTGATTCATGAATCTGTTGGATCTTTGAATGAGCTTGTATTGTTGGACCTTGGACAATGCACTAACCTTTCAAAGCTTCCGAGCTATCTCAGGTTAAAGTCTCTTGTCTATTTAGTACTTTTTGGGTGTGGTAAGCTTGAAAGCTTTCCAACAATTGCTGAAAACATGAAATCTTTAAGGTGCTTGGATTTGCATTCCACCGCCATAAAGGAGTTACCTTCATCACTTGGATATCTTACTCAACTCGATAAATTACACCTTACCGGTTGCACAAACCTCATCTCCCTTCCCAATACAATCTATTTGTTAAGGAATCTTAACGAACTTCATCTTGGTGGGTGTTCTAGATTTGAAATGTTTCCCCATAAATGGGTCCCAACCATCCAACCAGTATGCTCTCCTTCAAAAATGATGGAAGCAGCTTCGTGGAGCTTAGAGTTTCCCCATTTAGTAGTACCAAATGAAAGCATATGTTCCCATTTCACTTTGTTGGATCTTAAATCTTGCAACATATCAAATGCAAAATTTTTGGAAATTTTATGTGATGTTGCCCCTTTCTTATCTGATCTACGTTTGTCCGAAAACAAATTCTCTAGTTTACCCTCATGTCTCCACAAGTTCATGTCCTTATCGAATCTTGAATTAAGGAATTGTAAGTTTCTTCAAGAAATCCCAAACCTTCCCCAAAACATACGAAACTTGGATGCCAGTGGTTGCAAATCGTTGGCTCGAAGTCCAGATAACATTGTGGATATAATATCAATAAAACAGGACCTTGAATTGGGTGAGATTTTAAGAGAGTTCTTATTAACGGACATTGAGATTCCAGAATGGTTCAGCTATAAGACTGCATCCAATTTGGTGACTGCTAGCCTTCGTCACTATCCAGACATGGAAAGAACTTTGGCTGTCGCTGTTAGTTTTAAAGTGAATGGAGATTCATCTGAAAGTGAGGCCCAAATTTCATGCAATATATTCATCTACAATAAACTCCGTTGTTTGTTTTCAAGATCATTTCTTCCATCAAAATCAGAATATATGTGGTTAGTAACAATTTCTCTAGCGTGTTCCCTGGAGGTGAATGATTGGAATAAAGTTTTCGTCTGGTTTGAGGTTCATGAAGCACATGGTGTAACTGTAACAAGGTATGGGGTCCATGTCACTGAACAACTCCATGGGATACAAACGGATGTCAAGTGGCCGATGGTTAATTATGCTGATTTTTATCAACTGGAGAAATTGCGAAGGGATCTGTAA

>Cucsa.091820

ATGGGTTCTTCCATTGTTGGAGCTGAATCATCAACTTCTTCTTCTTCTAGTTTCAAGTGGAGTTTTGATGTGTTTTTGAGTTTTAGGGGAGATGATACTCGTTCTAATTTCACCGGTCATCTTGACATGGCCTTGCGTCAAAAGGGTGTCAATGTCTTCATAGACGACATGCTCAAAAGGGGTGAGCAAATTTCTGAAACCCTTTCCAAAGCTATACAGGAAGCTTTGATTTCTATTGTTATTTTCTCTCAAAATTATGCATCTTCTTCATGGTGTCTGGATGAATTGGTGAAAATAGTTGAGTGTAAGAAATCCAAGGGCCAGCTTGTTTTGCCAATTTTCTACAAGGTGGATCCTTCCGATGTACGAAAACAAACTGGTTGCTTTGGAGAAGCATTGGCCAAACATCAGGCTAATTTCATGGAGAAGACTCAAATATGGAGGGATGCTTTAACTACTGTTGCCAACTTCTCTGGTTGGGATCTAGGAACTAGGAAGGAGGCTGATTTTATTCAAGACCTTGTTAAAGAAGTATTGTCTAGATTAAATTGTGCCAACGGGCAGTTATATGTAGCTAAGTATCCAGTTGGAATTGATTCTCAACTAGAAGATATGAAGTTACTCTCGCATCAGATACGAGATGCGTTTGATGGCGTTTACATGATGGGGATATACGGCATTGGAGGCATTGGTAAGACTACTTTGGCTAAAGCTTTGTACAATAAAATTGCTAACCAATTTGAAGGTTTCTGCTTTCTATCAAATGTTAGAGAAACTTCAAAACAGTTCAATGGACTCGTTCAACTACAGGAAAAACTACTCTATGAAATCTTAAAGTTTGATTTGAAGATTGGCAATCTTGATGAAGGAATTAACATCATAAGAAGTAGATTGCGTTCAAAGAAAGTTCTTATAGTTCTTGATGATGTGGATAAGCTCAAGCAATTGGAAGCATTGGTTGGTGAACGTGATTGGTTTGGCCATGGTAGTAAAATCATTGTGACAACAAGAAATAGTCATTTACTTTCTAGCCATGAATTTGATGAAAAGTATGGTGTTCGGGAATTGAGTCATGGTCATTCCCTTGAACTTTTTAGTTGGCATGCTTTTAAGAAAAGTCATCCATCAAGTAATTACTTAGACCTTTCAAAACGTGCGACAAATTATTGTAAAGGTCATCCTTTGGCCCTTGTTGTTTTGGGTTCTTTCCTTTGTACCCGAGACCAAATAAAATGGAGAACTATATTAGATGAATTTGAGAACTCTTTGAGTGAAGACATTGAACATATTATTCAAATCAGTTTTGATGGGCTTGAAGAAAAAATAAAGGAGATCTTCCTTGATATTTCTTGTTTGTTTGTGGGAGAGAAAGATTGTAAAAGGTTGAAGCATGTTGATCTTAGTTACTCTTCTTTATTAGAGAAGATTCCCGACTTCCCTGCAACATCAAATCTTGAAGAATTATATCTTAACAACTGCACAAATTTAAGAACAATTCCTAAGTCAGTTGTTTCTCTTGGTAAGCTTCTTACTTTAGACCTTGATCATTGTTCAAACCTTATAAAGCTTCCAAGCTACCTCATGCTGAAGTCTCTTAAAGTTTTGAAGCTTGCTTACTGCAAAAAACTTGAGAAACTTCCAGACTTCTCTACAGCTTCAAACCTTGAAAAGTTGTACCTCAAAGAATGCACAAATTTAAGAATGATTCATGATTCTATTGGATCTCTGAGTAAGCTTGTTACCTTGGACCTTGGAAAATGCTCTAACCTTGAAAAGCTTCCAAGCTACCTCACATTAAAGTCTCTTGAATATTTGAATCTTGCTCATTGCAAAAAGCTTGAGGAAATTCCCGACTTCTCTTCTGCATTAAACCTTAAAAGCTTATATCTTGAACAATGCACAAATTTAAGAGTAATTCATGAGTCTATTGGATCTTTGAATAGTCTTGTTACCTTGGACCTTAGACAATGCACTAACCTTGAAAAGCTTCCAAGCTACCTCAAGTTGAAGTCTCTTAGACATTTCGAACTCTCTGGCTGCCACAAGCTCGAAATGTTTCCAAAAATTGCTGAAAACATGAAATCTTTAATTTCATTGCATTTGGATTCTACTGCCATAAGGGAGCTACCTTCATCAATTGGATACCTTACTGCGCTTTTGGTATTAAACCTTCACGGTTGCACAAATCTCATCTCCCTTCCTAGTACAATTTATTTGTTAAAGAGCCTTAAGCATCTTTATCTTGGTGGGTGTTCTAGATTTCAATTGTTTTCCCATTTTTTGGAAATTTTATGTAATGTCGCCCCTTTCTTATCTAGTATACTTTTGTCGGAAAACAAATTCTCTAGTCTACCACCATGTCTTCATAAGTTTATGTCCTTGTGGAATCTCCAATTAAGGAATTGCAAGTTCCTTCAAGAAATTCCTAACCTCCCTCATTGTATACAAAAAATGGATGCCACTGGTTGCACATTGTTGGGTAGAAGTCCAGACAACATCATGGACATAATATCGAGCAAGCAGGTTCCTCACTTCCATTTCCATTTTCCTTTGGTTGGAGATTCATATCAAGGAATGGCCTTAGTTTCATGTAAAATATTCATTGGCTACAGACTCCAAAGTTGTTTTATGAGAAAATTTCCATCATCAACATCAGAATATACATGGTTAGTAACAACTTCTTCTCCAACATTTAGCACTTCCTTGGAGATGAATGAGTGGAATCATGTCACAGTCTGGTTTGAGGTTGTGAAATGTTCTGAGGCCACCGTAACTATAAAATGCTGTGGTGTCCATCTCACTGAAGAGGTCCATGGAATACAAAATGATGTCAAGGGGCCAGGGGTAGTTTATACAGTTTTCGATCAACTGGACAAATTACCGAGCCGGATACGTGGTATGGAAGGCATGGCAGAGACAACACTTGCCAACTCTATATGTAACAAATATGAAAGAAGTCGGAATCTTTTCTCTGCAAAAAAAGCTTTGAATCATTCTACTGGCTTTCTTTGTGGAGATGGAAATGGGCTTTCTTGGGAAATGGTAGACAGACCGATATTAAGTGATAGATTGTCTTCCCAAAAATATCTTAGAATTTTCGACGATCGTGATCGATATGGAGACCTAAATGATGTGGCTCATGGGACTGGTAATAGGTTTCGTTCAAGATTTTTAAGGATGGATGATATAAAAGAAGATGATATCAGAGAAGAGCCTTATTGGAAGTACATGGAAAG

>Cucsa.091840

ATGGGTTCTAACGCTGCTGGAGCGGAATCGTCGTCTTCTTCTCCAATCAATTGGATTTATGATGTGTTTTTGAGTTTTAGAGGAGAGGATACTCGCTCCAATTTCACAAGTCATCTTCACATGTTCTTGCGTCACAAGGGTGTCAATGTTTTCATAGATGACAGGATCGAAAGGGGTGAGCAAATTTCTGAAGCCCTTTTAAAAACTATACAGTGTTCTTTGATTTCTATTGTTATATTCTCGGAAAATTATGCATCTTCTACATGGTGTCTGGATGAATTGGTGGAAATAATTGAGTGTAAGAAATCCAAGGGTCAGAAAGTATTGCCAATTTTCTACAAGGTGGATCCTTCGGATGTACGAAAACAAAATGGTTGCTATGGAGAAGGATTGGCCAAACATGAGGCTAATTTCATGGAGAAGATTCCAATATGGAGGAATGCTCTAACGACTGCTGCCAACTTGGCTGGTTGGGATCTCGGAACAATAAGAAATGAGGCTGACCTTATTCAAGTTATTGTTAAAGAAGTGTCGTCTACATTAAATGTCACCACGCCCTCAGATAAGCCTCTACTAGTTGGAATTGATTCCAAAATTGAATCCCTTTATTGGCCTACAGAAGAAATGTACAAGTCTGAATGTGTTGACATGTTGGGGATATATGGCATTCGAGGCATTGGTAAAACAACTTTGGCTAAAGCTTTATACTACAAAATGGCTAGCCAATTTGAATGTTGCTGCTTTCTATCAAATGTTAGAGAAGCTTCAAAGCAACTCAATGGCCTCGCTCAACTACAGAAAAAACTACTTTTTCAGATCTTAAAGTATGATTTGGAGGATGTCGATGATCTTGACAGGAGAAATAATATCATAAAGCATAGACTCCATTCGAAGAAAGTTCTTATACTTCTTGATGATGTGGATGAGATGAAGCAATTAAAAGCATTGGCTGGTGGGCATGATTGGTTTGGTCAGGGTAGTAAAATCATTGTGACGACTAGAGATAAACATTTACTTGATAGCCATGGATTTGGTCAAACATATGAAGTTGAAGGATTGTGGGAACACAATGCATTTGAGCTTTTTTGTTGGCATGCCTTCAAAAAAAGTCATCCATCTAGTAATTATTTAGACCTTTCAGAACGTGCTACAAGGCATTGTAAAGGTCATCCTTTGGCTCTTGTTGTTTTGGCTTCTTTCCTTTGTGGCAGAGATCAAGCAGAATGGAGTGGTCTATTAGATGGATTTGAAAACTCTTTGAGAAAAGGTATTAAAGATGTTCTTCAATTAAGTTTTGATGGGCTGGAAGACGAAGTAAAGAAATTTTTTCTTGATATTTCTTGTTTACTCGTGGGTGAGACAGTTACCTATGTTAAGAAAATGTTGAGTGAATTCCATTCGATTCTGGATTTCAAAATTAGCAATCTGAGGCATCTTTCACTTATTAGGATGGAAGAATATGATGATGATAGGGTGCAAATGCATGATTTAATAAAACAAATGGGTCATAAAATAGTTTATGATGAATGTGGTGATGAGCCTGGAAAAAGGAGTAGATCAGGGAAGCGATGCAGTGAAAGGCATAAAGTTGGTGTTTATCTGATCCCACCAAGGGTCATAAACGTGAATCCAGAGGCATTTAGAAGCATGAAGAATTTGAGAATACTGATTGTTGATGGAAATGTGAGGTTTTGCAAAAAAAAAAAGTATGTACCGAATGGGTTAAAGTGGATAAAATGGCATAGATTTCCTCATCAAACTTTACCCTCATGCTTTATTACAAAAGATCTGCTTCCAAGCTACCTCAAGTTAAAGTCTCTTACAGATTTAGATCTCTCTGGTTGTCGTAAGCTCGAAACGTTTCCAGAAATTGATGAAAACATGAAATCCTTAGAAAGGTTGAGGTTGTCTTATACGGCCATAAGGAAGCTACCTTCAGTCTTTGCGGGCTTAGTCTTTCTGAGTGTTCTATTGAAATGCGTTCCAAAATTTTTCCATTCACGAGTTCCAAAAGAAATCTTATTTTTCGAACATTTGGAGTTGCTGGATCTTAAAGGTTGCAATATATCAAATGTTGATTTTCTGGAAAATTTATGTAATGTAGCTCTATCCTTAACTAGTATAGTCTTGTCAGAAAACGAATTCTGTAGTCTACCTTCATGTCTCCATAAGTTTATGTCCTTGCGGAATCTCCAATTAAGGAATTGCATGTTTCTTCAAGAAATTCCAAACCTCCCTCAGAGTATACAAATAGTAGATGCCACTGGTTGCATATCGTTGAGAAGAAGTCCAAACATTATGTGGACATAA

>Cucsa.091880

ATGGAAAGTATTCCTATTTCAATAATTGCAAAAATTTGTGAATACACTGTTAAACCTGTTGGACGTCAACTTTGTTATGTATGTTTCATTCATTCCAACTTTCAAAAACTCAAGAGTCAAGTAGAAAAGCTGACAGATACAAAAGGATCTGTGGAAGACAAGGTTTTTATTGCAAGAAGAAATGCAGAAGACATAAAACCTGCAGTTGAGAAATGGTTGGAAAAGGTTGATCGCCTTGTTAGAAAATCTGAGAAGATACTAGCCCATGAAGGTAGGCATGGTAGATTGTGTTCCACCAATTTGGTCCAAAGACACAAGGCAAGTAGAAAAGCAAGCAAAATGGCAGATGAGGTTCTTGAGATGAAAAATCAGGGAGAAAGTTTTGATATGGTATCCTTTAAAGGTCGTATCTCATTGGTTGAGAGTCCACTGCCAAAAGCACCTGACTTTCTTGACTTTGGCTCTAGAAAGTCAACAGTGGAACAAATCATGGATGCACTCTCTGATGATAATGTCCATAAGATTGGAGTGTACGGGATGGGGGGTGTTGGCAAAACAATGCTAGTGAAAGAAATTGTAAGAAAAATTGAGGAGAGTAAGAAGTCTTTTGATAAGGTGGTAACATCCACGATTAGCCAAACACCAGATTTTAAAAGGATTCAAGGACAACTAGCTGACAAGATAGGTTTAAAATTCGAACAAGAAACAATAGAAGGAAGGGCTACTTTTCTACGAAGGTGGTTGAAGGCGGAGAGAAGAATTCCAAGTGTTGAAGATCATAAAGGAATATGCAAGATCTTGTTTACCTCTAGGAATAAACAATTGATCTCAAATGATATGGGCGCCAATAAAATTTTTGAGATAAAAGTTTTAGGAGAAGATGAGTCCTGGAATTTATTTAAGGCAATGGCGGGTGAAATTGTTGAAGCAACTGATTTGAAGCCTATAGCCATTCAAATTATGAGAGAATGTGCAGGTTTGCCTATTGCTATTACTACTGTTGCTAAGGCATTACTAAATAAACCTTCCGACATTTGGAATGATGCCTTAGATCAACTTAAAAGTGTTGATGTGGGTATGGCAAACATTGGAGAAATGGACAAGAAAGTGTATTTGTCACTAAAACTGAGTTACGATTACTTGGGATATGAAGAGGTGAAGTTACTATTCTTGTTATGTAGCATGTTTCCAGAAGATTTTAACATTGATGTGGAAAAGTTGCACGTATATGCTATGAGCATGGGTTTCTTACGTGGTGTTGATACTGTGGTAAAAGGACGACGAAGGATTAAAAAATTGGTTGACGATCTTATATCTTCTTCCTTGCTTCAACAATATTCTGAGTATGGGAACAATTATGTGAAAATACATGATATGGTTCGTGATGTAGCCATACTAATAGCATCTCAGAATGATCACATACGTACATTGAGCTATGTGAAAAGATCGAATGAAGAATGGAAAGAAGAGAAATTGTCGGGTAACCATACTGTAGTGTTCTTAATTATTCAAGAATTGGATTCACCTGATTTCTCAAAGTTAATGCTACCTAAAGTTCAATTGTTCGTGTTATTTGGACCATCACCATCTATATATAATAGACATGTTGTGTCAGTGGTAGAAACTTTCTATAAAGAAATGAAGGAGCTCGAAGGTTTGGTAATAGAAAGGGTGAAAATATCCTTATCGCCACAAGCTCTTTACTCATTTGCAAACCTTAGATTATTAAGATTACATGACTGTGAATTAGGGAGCATAGATATGATTGGTGAATTAAAAAAGCTTGAAATTCTTGATTTTAGTAAATCTAACATCGTTGAAATTCCTATGACCTTTAGCAAATTGACGCAGTTAAAAGTGTTAAATTTATCTTTTTGTGATGAGCTTGAGGTAATTCCACCCAATATTCTTTCAAAATTGACAAAACTGGAAGAATTACATCTAGAAACTTTCGATAGTTGGGAAGGGGAAGAATGGTACGAAGGAAGGAAAAATGCTAGTCTTTCTGAGCTAAGGTACTTGCCACACCTTTATGCTTTAAATTTAACCATTCAAGATGATGAGATTATGCCAAAACACTTGTTTTTAGCTGGGGAGTTGAATCTTGAAAATTTTCACATTACTATTGGTTGTCAGAGACAAAAAAGACATATTGATAATAAGACCAATTTCTTTAGAATCAAGATGGAATCAGAAAGGTGCTTGGATGATTGGATAAAAACTTTGTTAAAGAGGTCAGAAGAAGTCCATTTGAAAGGATCAATTTGTTCAAAGGTTCTCCACGATGCAAATGAATTCTTACATTTGAAAGAATCTCTTTTTAGCAAGTTGAAAAGTGTAGTCGTAACGAAGTGCAATAAATTAGAAAAGCTCTTTTTCAACTGCATATTGGATGACATTCTGAGTCTTGAGGAGATTGCTATTCATTATTGTGAGAAGATGGAAGTGATGATTGTGATGGAAAACGAGGAGGCAACCAACCACATTGAGTTTACTCATTTAAAGTATTTATTTCTAACGTATGTACCACAACTTCAAAAATTTTGCTCCAAAATTGAGAAATTTGGACAATTAAGTCAGGATAACTCAATCAGCAATACCGTTGACATTGGTATATTTGAAGTGCAAGAGTCAAGTATTACAGATACAAGTCTTATTGTGCTCAAAAACTTGAGAGAGTTGAAATTATATAATCTTCCCAACCTTGAGTACGTATGGAGCAAAAATCCTTGTGAGCTTCTGAGTTTTGTAAATATAAAAGGTTTGGCCATTGATGAATGTCCAAGACTTAGAAGAGAATATTCAGTCAAAATTCTCAAGCAACTTGAAAGACTAACAATGGATATTAAACAATTGATGGAGGTTATTGAGAATCAAAAGTCAACCGATCATAATATGGTGAAATCAAAGCAATTGGAGACTTCTTCTAAGGATAACTCAACTCATTTGCCAGTGGAAATTGTACAAATCTTATACCAACTTGAGCACTTTGAATTGGAAGGAGCGTATATTGAAGAAGTTTTCCCCAGCAATATATTGATTCCAATGAAAAAACAGTACTATGCAAGATCTAAGAATTCAGTGCGTAGTTGGTTTCTATCTAAACTACCCAAGCTTAGGCATTTGTGGAGTGAATGTTCACAAAAGAATGCTTTTCCAATTCTACAAGATTTGAATGTAATAAGAATTTCAGAATGTGGTGGGTTGAGTTCCTTAGTTTCGTCATCAGTATCCTTTACAAACTTGACAGTTCTTAAAGTGGATAAGTGTGATAGACTAACCTATTTGTTGAATCCTTTGGTGGCTACAACCCTTGTGCAACTTGAAGAATTGACTTTAAGAGAATGCAAAATGATGAGTAGTGTAATTGAGGGAGGATCAGCTGAAGAAGATGGAAATGAGGAGACAACCAACCAGATTGAGTTTACTCATTTGAAGTCTTTATTTCTAAAGGATTTACCACGACTACAAAAGTTTTACTCTAAAATTGAGACATTTGGTCAATTAAGCCGTGATAATTCCGAAAACCCTGAAACAACCACAATTCACAATCGCATTGGTGATTCATTTTTCAGTGAACAGGAATCACTTCCTAATTTGGAGACATTGAGAATTGATGGTGCAGAGAATTTGAGGATGATATGGAGTAATAATGTACTCATTCCTAATTCCTTTTCCAAACTCGAGGAAGTAGAGATTTATTCATGCAACAATCTTCAGGACGTATTATTTCATCCAAATATTATAAACATGCTTACATGCCTTAATACATTAAGGATTAAAAATTGTGAATTATTGGAGGGGATATTCGAAGTGCAAGAGCCGATTAGTGTTACAAAAACAAAAACAAATGCTATCGTGCTACCAAATAATTTGATAGAGTTGGAATTATATAATCTTCCAAACCTTGAGTACCTATGGAGTAAGAATCCAAATTTTGAACGGCTCGTGACTTTTGAAAGTATAAGAAGTTTGTCCATTGAAAAATGTTCAAAACTCAAAGGAGAATATTTTTTGTCAATCAAAACTTTCAAGCAACTTGTTGAGGTTCTACAGTTGAGAGATGGTTCTAAGTTGTTTTCCAATCTTAAAGAATTGAAGCTATATGGTTTTGTTGAGTATAACTCAACTCATTTGCCAATGGAAATTGTTCAAGTCTTAAACCAACTTGAGAAGTTTGAATTGAAAGGAATGTTCATTGAAGAAATTTTCCCCAGCAACATACTGATTCCAAGCTATATGGTTTTACGAAAATTGACTCTATCTAAACTATCCAAGCTTAGGCATTTGTGGGGTGAATGCTCACAAAAGAATAATGATTCACTTCTACGAGATCTGACCTTTTTATTCATTTCAAAATGTGGTGGATTGAGTAGTTTAGTGTCATCATCGGTGTCATCTTTTACAAACTTGAGAATTCTTGAAGTGGAAAAGTGTGATGGACTAAGCCATTTGTTGAGTCCATCGGTAGCTACAACGCTCGTGCATCTTGAAGAATTGAGAATAGAAGAATGCAAAAGGATGAGTAGTGTAATTGAGGGAGGATCATCTGAAGAAGATGGAAATGATGAAATTATTGTATTCAACAACCTACAACATTTAATCATTTCTTCTTGTTCCAACCTAACAAGTTTTCATTGTGGAAGATGCATCATTCAATTTCCATGTTTGAAACAAGTATACATTAACAAGTGTACTGAATTGAAGGTCTTTTCGCTTGGAATTGTAAGCACACCTCCTTTAAAATATGAAAATATTTATTTAAAGAATGATGATGATGATGATACGTGGCATCACCCAAAAGAATCCATAGAAATGGTGGTGGAAACTGATATGAATGTCATCATCAGAGAATATTGGGACGACAACATCGATACTAGAATTTCAAATTTGTTTGGAGAAGAG

>Cucsa.094560

ATGGGTGGAATCGGAAAAACAACTTTGGCAAAGACAATCTTCAATCATGAGGAGATCAAAGGACATTTTGATGAAACAATTTGGATATGTGTGTCCGAACCATTTCTTATCAACAAAATTTTGGGAGCAATTTTACAAATGATAAAGGGTGTTTCCAGTGGCTTGGATAATAGAGAGGCTTTACTTCGAGAGCTTCAAAAGGTGATGCGAGGTAAAAGATATTTTCTTGTGCTTGATGATGTTTGGAATGAAAATCTTGCTTTATGGACTGAATTGAAACATTGTTTACTGAGTTTCACTGAAAAATCTGGAAACGCTATTATTGTGACTACAAGAAGTTTCGAAGTAGGAAAGATTATGGAGAGTACTCTTTCTAGCCATCATTTGGGAAAATTATCTGATGAACAATGTTGGTCTTTGTTTAAAAAAAGTGCAAATGCAGATGAACTGCCAAAGAATCTAGAGTTGAAGGATCTTCAAGAAGAATTGGTGACAAGGTTTGGTGGTGCACCATTGGTTGCAAGAATGAAACATTTGAGATATCTCGACATTTCAAATTCAAAGATAGAGGAACTTCCAAATTCTATCTCTTTGCTTTATAACTTACAAACACTGAAGCTTGGAAGCTCAATGAAAGACCTTCCACAGAATTTGAGCAAGTTGGTTAGTTTAAGACATCTAAAGTTCTCAATGCCACAAACGCCTCCACATTTGGGTCGATTGACTCAACTTCAAACATTGTCTGGTTTTGCAGTTGGATTCGAGAAGGGTTTCAAAATAGGAGAACTTGGATTTTTGAAAAACCTCAAAGGTAGATTAGAACTTTCAAATCTTGATCGAATTAAACATAAAGAGGAAGCCATGAGTTCCAAATTGGTAGAAAAGAACTTGTGTGAGCTATTCTTGGAATGGGATATGCATATTTTAAGAGAAGGTAACAACTACAATGACTTTGAAGTGTTAGAAGGGCTTCAACCACACAAAAATCTTCAATTCCTGAGTATCATAAACTTTGCTGGCCAACTTCTGCCTCCTGCCATTTTTGTTGAAAATTTAGCTGTGATACATCTAAGACATTGTGTAAGATGTGAAATACTTCCAATGCTTGGACAATTACCTAATTTAGAGGAACTAAATATTTCCTACTTACTTTGTCTAAGAAGTATTGGCCATAAGGTTTTATTTCCCAAGTTGAAGAAATTTGTACTCTCTCAAATGCCCAATCTAGAGCAATGGGAAGAAGTAGTATTCATATCAAAGAAAGATGCAATTTTTCCTCTTCTTGAGGACTTAAATATTAGTTTTTGTCCTATATTAACAAGTATTCCAAATATTTTTAGACGTCCTCTTAAAAAGCTACATGTTTATGGATGTCATGAAGTGACAGGATTGCCAAAAGATCTACAACTTTGCACTTCCATTGAGGATCTAAAGATTGTTGGGTGCCGTAAAATGACACTAAATGTGCAAAATATGGATAGCTTGTCTCGTTTCTCTATGAATGGGTTGCAGAAGTTTCCCCAAGGGCTGGCTAATCTAAAAAACTTGAAAGAAATGACAATCATTGAATGCTCACAAGATTGTGACTTTAGTCCTCTCATGCAACTTTCTTCACTTGTAAAGCTTCATTTGGTTATTTTCCCAGGGAGCGTGACTGAGCAACTTCCTCAACAACTTGAGCATCTCATTGCCTTAAGATCTTTGTACATTAATGATTTTGATGGAATTGAGGTTTTACCAGAATGGTTGGGAAACCTTACCTCTTTGGAAGTTTTGGGACTTTATTATTGTATAAATTTGAAACAGTTTCCTTCAAAGAAAGCCATGCAATGTCTCACCCAATTAGTCCACGTGGATGTCCACAACTGCCCGAGTTCGCAGATTTTGTCCCATGATCTAAAGGCCAAAGCTCATGCCAAAGCAAACTTAGTTCAATGGTAA

>Cucsa.094580

ATGGCTATCGCTGAGTTCCTATGGACTTATGCTGTCCAACAAGTGTTGAAGAAGGTATTGGAACTTGCGGCTGACCAAATTGAAAAAAAACTACACCATAGTTCTGTGAGACTGTGGGTGGCAGATCTTCTACTTGTTGTTCATGAAGCCGACAATCTATTGGATGAGCTTGTTTATGAATATCTTCGTACAAAGGTGGAAAAAGGATCGATTAACAAGGTATGTTCTTCGGTGTCAAGTCTTTCTAATATTTTCATTATCTTTCGCTTCAAAATGGCCAAGAAAATCAAGAGTATTATTGAAAAGTTGCGTAAATGTTACTACGAGGCGACTCCTTTAGGACTTGTTGGTGAAGAATTCATAGAAACAGAGAATGATCTTAGTCAGATTCGAGAGACGATCTCAAAACTTGATGATTTTGAAGTTGTTGGAAGGGAGTTTGAAGTTTCAAGCATAGTGAAACAAGTAGTTGATGCTAGTAATCAATATGTTACATCTATCTTACCCATTATGGGTATGGGTGGAATCGGAAAAACAACTTTGGCAAAGACAATCTTCAATCATGAGGAGATCAAAAGACATTTTGATGAAACAATATGGATATGTGTGTCCGAACCATTTCTTATCAACAAGATTTTGGGAGCAATTTTACAAATGATAAAGGGTGTTTCTAGTGGCTTGGATAATAAAGAGGTTCTACTTCAAGAGCTTCAAAAAGTGATGCGAGGTAAAAGATATTTTCTTGTGCTTGATGATGTTTGGAATGAAAATATTGCTTTATGGACTGAATTGAAAAAATGTTTACTGTGTTTTACTGAAAAATCTGGAAACGGTATCATTGTAACTACGAGAAGTATTGAAGTTGGAAAGATTATGGAGAGTACTCTTCCTAGCCATCATTTGGGAAAATTATTTGATGAACAATGTCGGTCTTTGTTTAAAGAAAGTGCAAATGCAGATGAATTGCCAATGGATCCAGAGTTGAAGGATCTTCAAGAAGAATTGGTGACAAGGTTTGGTGGTGTACCATTTGTTGCAAGACTTGGAAGCTCAATGAAACACCTTCCATATAATTTGAGCAAGTTGGTTAGTTTAAGACATTTAAAGTTCTCAATACCACAAACGCCTCCACATTTGAGCCGGTTGACTCAACTACAAACGTTGTCTGGTTTTGCAGTTGGATTTGAGAAGGGTTGCAAAATAGAAGAACTTGGATTTTTGAAAAACTTCAAAGGTAGATTAGAACTTTCAAATCTCAATGGAATTAAACACAAAGAGGAAGCCATGAGTTCCAAATTGGTAGAAAAGAACTTATGTGAGCTATTCTTGGAATGGGATTTGCATATTTTAAGAGAAGGTAGCAACTACAATGACTTGGAAGTGTTAAAAGGGCTTCAACCACACAAAAATCTTCAATTCTTGAGTATCATAAACTATGCTGGCCAAATTTTGCCTCCTGCCATTTTTGTTGAAAATTTAGTTGTGATACATCTAAGACATTGTGTAAGATGCGAAACACTTCCAATGCTTGGAGAATTACCTAATTTGGAGGAACTAAATATTTCCAACTTACATTGTCTAAGATGTATTGGGAATGAATTCTACGGAAGTTATGATCATCCCAACAACCATAAGGTTTTATTTCGCAAGTTGAAGAAATTTGTACTCTCTGAAATGCACAATCTAGAGCAATGGGAAGAATTAGTATTCACATCAAGGAAAGATGCAATTTTTCCTCTTCTTGAAGACTTGAATATTCGTGATTGTCCTATATTAACAAATTGCCCAAAGATCGATCTACAACTCTGCACTTCCATTGAGGATCTAAAGATTGTTGGGTGCCTTGAAATGATACTAAATGTGCAAAATATGCATACCTTGTCTCGTTTCTCTATGAATGGGTTGCAAAAGTTTCCCCAAGGACTATCTCATCTCAAAAACTTGAAAGAAATGATAATCACTGAATGCTCACAAGATTGTGACTTTACTCCTCTTATGCAACTTTCTTCACTCGTAAATCTTGATTTGGTTCTTTTCGCGGGGAACGGGGCCGTGCAACTTCCTCAGCAACTCCAGCATCTCACAGCCTTAAGATCTTTGATCATTAATGATTTTGATGGAATTGAAGTTTTACCAGAATGGTTGGGAAATCTTGCATCTTTGGAAGTTTTGGGACTTTATTATTGTAGAAGTCTGAAACAGTTTCCTTCAAAGAAAGCCATTGCAATGTCTCACCCAATTAGTCCATGTGGATGTCTTTGGTTGTCCACAACTACCCAAGTTCGGAGATTTTGTGCCATGATGTTCTAA

>Cucsa.094650

ATGGCTGAATTCCTTTGGACATTTGCTGTGGAAGAGACGTTGAAGAGAACGGTGAACGTTGCAGCTCAGAAAATTTCTCTCGTTTGGGGTTTGGAAGATGAACTTTCAAATTTAAGCAAATGGCTACTCGATGCTGGAGCCCTTTTGCGCGATATCGATAGGGAAATACTTCGCAAGGAATCGGTGAAGAGATGGGCAGATGGGCTTGAAGATATCGTTAGTGAAGCTGAGGATCTTTTGGACGAGCTTGCTTATGAAGATCTTCGAAGAAAAGTGGAAACAAGTTCAAGGGTGTGTAATAATTTCAAATTTTCTTCTGTTCTTAACCCTCTTGTTCGTCATGATATGGCCTGTAAAATGAAGAAAATTACTAAAATGTTAAAACAACATTATCGCAACTCTGCTCCTTTAGGGCTTGTTGGGAAGGAATCCATGGAGAAAGAAGATGGAGGTAATAATCTTAGGCAGATTAGGGAAACAACTTCGATTCTGAATTTTGATGTTGTGGGAAGGGAAACTGAAGTTTTAGACATATTGAGATTGGTGATTGATTCTAGTAGTAATGAGTATGAGCTTCCTTTGTTGATTGTACCGATTGTAGGGATGGGTGGAGTTGGAAAAACAACTTTGGCGAAATTGGTTTTTCGTCATGAGTTGATCAAGAAACATTTTCATGAAACAATATGGATATGTGTGTCGGAACACTTCAACATCGACGAGATTTTGGTAGCAATTTTGGAAAGTTTGACGGATAAAGTTCCAACCAAAAGGGAAGCTGTACTTCGCAGGCTTCAAAAAGAGTTGCTAGACAAAAGATGTTTCCTTGTTTTGGATGATGTTTGGAATGAAAGTTCTAAGTTGTGGGAAGAGTTAGAAGACTGTTTAAAAGAGATAGTTGGGAAATTTGGAATCACCATTATAGTAACTACAAGGTTGGATGAAGTTGCTAATATTATGGGAACAGTTTCGGGTTATCGTTTGGAAAAGTTACCTGAAGACCATTGTTGGTCCTTATTTAAGAGAAGTGCAAATGCAAATGGAGTAAAAATGACTCCAAAGTTGGAGGCTATTCGAATAAAGTTGCTTCAAAAAATTGATGGCATACCGCTTGTTGCAAAAGTTTTGGGAGGAGCCGTGGAATTTGAAGGAGATCTTGATAGGTGGGAGACCACACTTGAAAGCATAGTAAGAGAAATTCCAATGAAACAAAAAAGTTATGTGTTGTCCATATTACAATTAAGTGTGGACCGTCTACCCTTTGTGGAAAAACAATGTTTTGCCTATTGTTCAATTTTTCCTAAAGATTGTGAAGTTGTTAAAGAAAATTTGATTAGAATGTGGATAGCACAAGGGTTTATTCAACCAACAGAAGGAGAGAACACGATGGAGGATCTGGGAGAAGGGCACTTCAACTTCCTCTTATCTCGCTCCTTATTTCAAGATGTCGTCAAGGATAAGTATGGGAGAATTACTCACTTTAAGATGCATGATCTAATACATGATGTTGCCCTTGCCATTTTGTCAACTCGTCAAAAGTCGGTATTAGATCCTACTCATTGGAATGGAAAAACGTCAAGAAAGTTGCGCACCTTACTTTACAATAACCAAGAGATCCACCATAAAGTTGCAGACTGTGTTTTCTTGCGTGTTTTAGAAGTGAATTCCTTACATATGATGAATAACTTACCAGACTTCATTGCTAAGTTGAAACACTTGAGATACCTTGACATTTCATCATGTTCTATGTGGGTTATGCCCCACTCTGTTACTACGCTTTTCAATTTACAGACACTGAAGCTTGGAAGTATAGAAAATCTTCCAATGAATTTGAGAAATTTGGTTAGACTACGTCACTTAGAATTCCACGTCTATTACAACACAAGGAAAATGCCTTCTCATATGGGTGAGTTGATTCATCTTCAAATATTGTCTTGGTTTGTTGCAGGGTTTGAGGAAGGCTGTAAAATTGAAGAACTCGGAAATTTGAAAAATTTGAAAGGTCAATTGCAACTTTCAAATCTTGAGCAAGTGAGGAGTAAAGAAGAAGCTCTAGCTGCAAAATTGGTCAATAAGAAAAACTTACGTGAGCTAACTTTTGAATGGAGTATAGATATTTTACGAGAATGTAGCAGCTACAATGACTTTGAAGTGTTGGAAGGACTTCAACCACCCAAAAATCTCAGTTCTTTGAAAATTACCAACTTTGGAGGGAAATTTTTGCCTGCTGCTACTTTTGTTGAAAATTTGGTGTTCCTATGTTTGTATGGTTGTACAAAATGTGAAAGGCTTCCAATGCTTGGACAATTAGCCAACTTGCAAGAACTTAGTATTTGTTTCATGGATAGTGTGAGAAGTATAGGGAGTGAGTTTTATGGCATTGACTCCAACCGAAGGGGTTATTTTCCCAAGTTGAAGAAATTTGACTTCTGTTGGATGTGCAACCTAGAGCAATGGGAATTAGAAGTGGCAAATCATGAGTCAAATCATTTTGGTTCTCTTCAAACTCTAAAGTTGGATAGATGTGGCAAATTGACAAAACTGCCAAATGGGTTAGAATGTTGCAAATCTGTTCATGAGGTGATAATATCAAATTGTCCTAACCTTACCTTAAATGTAGAGGAAATGCATAACCTGTCTGTTTTATTAATAGATGGGTTGAAGTTTTTGCCAAAAGGATTAGCTCTCCACCCTAACTTGAAGACCATAATGATTAAAGGATGCATAGAGGATTATGATTATAGCCCTTTCCTAAACTTGCCTTCTCTTACAAAACTTTACTTGAACGATGGCCTTGGAAATGCCACCCAGCTTCCTAAACAACTTCAGCATCTCACTGCCTTAAAGATTTTAGCCATTGAAAATTTTTATGGCATTGAAGTTCTTCCTGAATGGTTGAGAAAGCTTACATGTTTGGAGACTTTGGATCTTGTTCACCTGGTGAAATCTTTACCATCTCTTCAAGGAGAAGACAATCGTGTGGCGGAAATTGCGTATGAAACGGGTGAGATGGAAACATACGAATGTGACAACAAAAGATCATGGTTTCGACTACATTTAAATTTTTACGGTTGTACCTTAGTAGAGCTTGTTTAA

>Cucsa.094660

ATGGCGGATTTCCTATGGAGCTTTGCTGTAGATGAAGTGTTAAAGAAGACAGTGAAGCTTGTGGCAGAGCAAATTGGCATGTCATGGGGGTTTAAGAAGGATCTTTCAAAACTAAGGGACTCTTTACTAATGGTAGAAGCCATCCTACGTGATGTTAACAGAATCAAGGCAGAACATCAAGCCTTGAGGCTATGGGTGGAGAAGCTTGAACATATCGTTTTTGAAGCCGACGTTTTACTCGACGAGCTCTCTTACGAAGATCTTCGACGCAAGGTGGACGCCAGGCCGGTACGTAGTTTCGTTTCATCCTCAAAAAATCCCCTTGTTTTTCGCCTCAAAATGGCCAATAAAATTAAAGCTATTGCTAAAAGGTTAGACGAGCATTATTGTGCAGCGAGTATCATGGGGCTTGTTGCTATAACATCCAAAGAAGTCGAGTCCGAACCTAGCCAAATTCTAGAGACAGACTCGTTTCTTGATGAGATTGGAGTTATAGGGAGGGAAGCTGAAGTATTAGAGATAGTGAATAAACTACTTGAACTTAGCAAACAAGAAGCAGCTCTATCTGTTTTACCAATTGTTGGTATAGGTGGACTAGGAAAAACATCTTTGGCGAAGGCGATATTTCATCATGAAATGATAAGGGAGAATTTCGATAGAATGATATGGGTGTGTGTGTCTGAACCTTTTGTTATCAACAAGATTTTAAGAGCAATTTTGGAAACTCTTAATGCTAATTTTGGTGGATTAGACAATAAGGAAGCTTTACTTCAAGAGCTTCAAAAATTGTTGAGGAACAAAAAGTATTTTCTGGTGCTTGACGATGTCTGGAATGAAAATCCTGATCTGTGGAATGAGTTAAGGGCTTGTTTGCTAAAGGCCAATAAAAAATTTGGAAGTGTTATTGTTGTGACTACTAGGAGTGATGAAGTTGCAAATATTGTGGAGACAAATCATCAAAGACATCGTTTGAGAAAGTTATCAAATGATTATTGTTGGACTTTATTTGAAAAATGTGCATTTGGAAGTGATTTGCCAGTGACTCCAAGAGTTGATCATGTAATCAGAGAAGAGCTTGTTAAAAGATTTGGTGGCATACCTTTGGTTGTGAAAGTGTTTGGAGGAATGGTGAAATTAGACAAGAATAAATGTTGTCAAGGATTGCGATCAACTTTGGAAAATCTAATCATAAGTCCATTACAATATGAAAATAGTATTTTATCTACCATAAAATTAAGTGTGGACAGGCTGCCATCATCTTCATTGAAGCAATGTTTTGCCTATTGTTCAAACTTTCCACGAGGCTTCTTATTTATAAGAGAACCACTTGTTCAAATGTGGATAGCACAAGGGTTTATTCATCTACCTAGTGGGAGCAATGTAACGATGGAGGATATTGGAGCAAACTACTTTAATACTTTGTTGTCTCGCTCTTTGTTTCAAGATGTCGTCAAAGATGACAGAGAAAGAATTCTGTATTGCAAGATGCACGATGTTGTACATGATGTTGCATGTGCTATTTCAAATGCTCAAAAATTGAGACTGAGTGGCAAATCTAATGGAGACAAAGCTCTTTCGATCGGTCATGAAATTAGAACACTTCATTGCAGTGAAAATGTTGTTGAACGGTTTCACCTGCCAACCTTTGATAGTCATGTATTTCACAATGAGATCAGCAACTTCACCTACTTGTGCGTTTTAATTATTCATTCATGGTTTATACATCAACTGCCAGATTCAATTGCTAAGTTGAAGCATTTAAGGTACCTCGACATTTCACACTCTCTAATAAGAACGCTTCCAGACTCTATTGTTTCACTCTATAATCTGCAGACATTGAGGCTTGGAAGTAAAATTATGCATCTTCCTACAAAATTGAGAAAATTGGTCAATTTAAGGCATTTAGAATTCTCTCTCTCAACTCAAACTAAACAAATGCCTCAACATCTGAGTCGATTGCTTCAACTTCAAACGCTTTCGAGTTTTGTAGTCGGTTTCGACAAAGGATGTAAGATAGAGGAACTTGGACCACTGAATAACCTTAAAGGTGAACTAAGCCTTTTCCATCTTGAGCATGTCAAAAGTAAAACCGAGGCTATGGCTGCAAATTTGGCAATGAAGGAAAACATTTCTGATCTATATTTTCAATGGAGTTTGTTAAGTGAAAGAGAAGATTGTAGTAACAATGATTTGAATGTGTTGGAAGGGCTTCGACCACACAAAAACCTTCAAGCCTTGAAAATTGAAAACTTTGGAGGTGTTCTGCCTAATGGCCTCTTTGTTGAAAATTTGGTGGAGGTAATTCTATATGATTGCAAAAGATGTGAAACTTTGCCAATGTTGGGGCACTTATCTAAGCTTGAATTACTTCATATTCGTTGCTTAGATAGTGTAAAAAGTATTGGGGATGAATTTTATGGGAACAATAATAGTTACCACAATGAGTGGTCTTCATTGTTATTCCCTAAACTCAAGACCCTTCATATTTCCCAAATGAAAAGTTTAGAGCTTTGGCAAGAAATAGGGAGTTCATCAAACTATGGTGCGACCTTTCCTCATCTTGAAAGCTTGAGCATTGTTTGGTGTTCGAAATTGATGAATATTCCTAACCTTTTTCAAGTTCCTCCAAAGCTTCAATCTCTCAAGATTTTTTATTGTGAAAAATTGACAAAGTTACCACATTGGTTAAATCTCTGCAGCTCCATTGAAAATATGGTCATATGCAATTGTCCTAACGTTAACAATAATTCTCTTCCAAATTTGAAAAGTATGCCAAACTTGTCGTCCTTGAGCATCCAAGCTTTCGAGAAGTTGCCGGAGGGGCTTGCCACCATTCATAACTTGAAAAGATTGGATGTTTATGGGGAATTGCAAGGTTTGGATTGGAGTCCATTCATGTATCTCAATTCATCGATTGAAATTCTTCGTGACATTGATTCTTTGCCAGAATGGTTGGGAAACCTTACATCTTTAGAGACGTTAAATCTACGTTATTGCAAAAATTTGAAAAGTTTCCCTTCAATAGAAGCCAT

>Cucsa.094670

ATGGACTTATTGTATTCCAAAAATTATGAGGACAAAATACTGTCAAACCTCAGAGACTCTCTACTTATGGTGGAAGCCATTCTTCGTGATGTCGACAGAATTAAGGCAGAGCATCAAGCTGTGAAGCTATGGGTAGAGAAGCTTGAAGCTATTATTTTCGAAGTCGATGTTCTACTGGATGAGCTCGCTTACGAAGATCTTCGCCGCAAGGTTGAACCCCAAAAAGAGATGATGGTAAGTAATTTCATTTCTTTCTCCAAAACCCCTCTTGTTTTTCGTCTCAAAATGGCCAATAAAATCAAGAACATTGCTAAGATGTTGGAAAGACATTATTCTGCTGCTAGTACTGTGGGGCTTGTTGCTATATTATCTAAACAGACTGAACCTGATTTTAGCCAAATTCAGGAGACAGATTCGTTTCTTGATGAGTATGGAGTTATTGGGAGAGAAAGTGAAGTTTTGGAGATTGTGAATGTATCTGTCGATCTTAGCTATAGGGAGAATTTGTCTGTTTTGCCAATTGTTGGCATGGGTGGATTAGGAAAGACAGCTTTGGCTAAGGTAATATTCAATCATGAATTGATAAAGGGGAATTTTGATAGAGCTGTATGGGTGTGTGTTTCGGAACCTTTTCTTATCAAGAAGATTTTAAGAGCAATTTTGGAAACTCTTAATTCTCATTTTGGTGGCTTAGATAGTAAAGAAGCCTTACTTCAAGAGCTACAAAAGTTGTTGAATGATAAAAAGTATTTTCTAGTTCTTGATGATGTTTGGAATGAGAATCCTATCCTCTGGAATGAGTTGAAAGGTTGTTTGTTAAAGATTAGCCAAAGATCTGGAAATGTTGTTGTTGTGACTACTAGGAGTGACAGAGTTGCTGAAATCATGGAGACACATTCTAGATATCATTTGACAAAACTATCCGATGACCATTGCTGGTCTTTATTCAAGAAATATGCATTTGGAAATGAATTGCTACGAATTCCTGAATTGGATATTGTTCAGAAAGAGCTCGTTAAAAGATTTGGAGGCATACCATTGGCTGTAAAAGTGATGGGAGGAATCGTTAAATTTGACGAGAATCACGAGGGATTGCAGAAATCTTTGGAGAATCTAATGAGACTTCAATTGCAAGATGAAAACCATGTTGTATCCACAATAAAGTTAACTGTAGATCGCCTACCATTGCCATCGTTAAAACAATGTTTTGCCTACTGTTCAAATTTTCCAAAAGACTTTAAGTTCAGAAAAGAAGCCCTTATTCAGATGTGGATAGCACAAGGCTTTATTCAACCGTCTTTGGGAAGTGATGAAATGATGGAGGATATTGGTGAGAAGTACTTCAATGTTTTGTTGTCTCGCTTCTTGTTTCAAGATATTGTCAAGGATAATAGAGGGAGAATTATATTCTGTAAGATGCATGATCTTATACATGATGTTGCATGTGCTATTTCAAATTCTCCAGGATTGAAATGGGATCCTTCAGATTTGTTTGATGGAGAACCTTGGAGACGTCAAGCTTGCTTTGCTAGCCTTGAACTAAAAACGCCAGATTGTAATGAAAATCCTTCTAGAAAGTTGCACATGTTGACATTTGATAGTCATGTGTTTCACAATAAGGTCACAAACTTTCTCTACTTGCGGGTTTTAATTACACATTCGTGGTTTATATGTAAATTACCAAATTCAATTGCTAAGCTGAAGCATTTGAGGTATCTTGACATTTCATATTCTACCATAAGGGAGCTACCAGATTCCGCTGTTTTGCTTTATAATTTGCAAACACTGAAGCTTTCAAGATTTTTAAACGGCCTTCCAAAAAATTTGAGGAAGTTGGTTAGTTTAAGACATTTAGAATTTTTCTCTGATCCTTGTAATCCTAAACAAATGCCTCAACATTTGGGTAAATTGATTCAACTTCAAACGTTGTCTAGCTTTGTAGTTGGGTTTGATGATGGATGTAAGATAGAAGAACTCAGATCTTTGAGAAATCTTAAAGGTAAGTTAAGCCTTTTATGTCTTGAGCGAGTGAAAAGTAAAAAGGAAGCCATGGCTGCAAATTTGGTGGAGAAGAGGAATATTTCATATCTGTCTTTTTATTGGGCCTTGAGATGTGAAAGATCAGAGGGAAGCAACTACAATGATCTGAACGTGTTAGAAGGACTTCAACCACATAAAAATCTTCAAGCTTTGAGAATTCAAAACTTTTTAGGCAAACTTCTGCCCAATGTTATTTTTGTCGAAAATTTGGTCGAGATATATCTACACGAATGCGAAATGTGTGAAACTTTACCAACACTTGGGCAGTTATCAAAGCTTGAAGTACTCGAACTTCGTTGTCTATATAGTGTAAGAAGTATTGGAGAAGAATTTTATGGGAATTACCTTGAGAAGATGATTTTATTCCCAACATTGAAAGCATTTCATATCTGTGAAATGATCAATCTAGAGAATTGGGAAGAAATAATGGTTGTATCAAATGGTACAATCTTTTCCAACCTTGAAAGCTTCAACATTGTTTGTTGTCCGAGATTGACGAGCATTCCAAACCTTTTTGCATCTCAGCATGAGAGTTCATTTCCAAGCTTACAACATTCGGCAAAGCTTCGATCTCTAAAGATTTTGGGATGTGAAAGTTTGCAAAAACAACCAAATGGTTTAGAATTCTGCAGCTCCCTTGAAAACATGTGGATAAGCAACTGTTCTAACTTGAACTACCCTCCAAGCTTGCAGAATATGCAGAATTTAACTTCTTTAAGCATAACCGAGTTTCGAAAGCTGCCAGACGGGTTAGCTCAGGTTTGTAAGTTGAAAAGCTTGAGTGTTCATGGTTACTTGCAAGGTTACGATTGGAGTCCTCTTGTACATCTTGGTTCACTCGAAAATCTTGTGTTGGTTGACTTGGATGGAAGTGGTGCAATACAACTTCCTCAACAACTTGAGCAACTCACTTCTTTGAGATCACTGCATATTTCGCATTTTAGTGGCATTGAAGCGCTACCAGAATGGTTCGGAAACTTTACATGTTTGGAAACGTTGAAGCTTTACAATTGTGTAAACTTGAAAGACATGGCGTCGAAGGAAGCTATGTCAAAACTTACAAGATTAACGAGTCTACGAGTTTATGGATGTCCACAACTTAATACTTTTATGTTTAATGGTATGTCACCAAATTCCTCGACGAGCTCTTCCCGAACCTTATCGACATTGGAATGA

>Cucsa.102240

ATGGCGCTGGAATTGGTGGGTGGGGCTGTTTTGGGGGCTGTCGTTGGGGAGCTATTCAAAGCGATCTTGAATCTGGGTGAAAGGGCCATCAGTTTCAATCCTGTTCTTAAGGATATCCGTTCCAAGCTTAATGCTATAATGCCTTTGGTGAAGCAAATCGATGAGCTTAATGATTATCTCGATTACCCAAAAGAAGAAACAGAGAAATTGAGGGGTCTGATGGATGAAGGGAAGCAGTTGCTTCTCCAGTGCGGCGATGTGAAATTGGGGGATCTTAATTATTTGAAGAGACCATCTTACACCCAAAAGCTTCGGGAATTGGATACTGCACTTCGAAGCTTCATGGATGTTTTGATGTTGCAGATGGCTAGAGATCAGAAGAAGAACATGAAGATGATGAACCAAATGATGGAGATCATTTGTAGACTTGATAATAGAGGTGGGTCGAGTAAACCTATGGATTTGTTTGTTCCACCATGTCTGGTTCCTCAACTGCGAGAAGAAACCGTTGGGTTGGAGAAGCCAGTTAAGGAGTTGAAGGTGAAACTTCTCAAAAATGGGGTTCAAATGTTGGTGGTGACAGCTCCTGGTGGCTGCGGAAAAACCACACTGGCCTTAAAATTTTGCCACGACAAAGAAGTCAAAGATATATTCCAGGAGAAGATCTTTGTCCCAGTTTCAAGAAAACCAGATTTGAAGCTTATATTGAAAGATATAATTGAAAGCCTTAGAGGAATTCAATTGCCTGATTTGCAAAGTGATGAACGTGCATTCTGCTATTTAGAATTGTGGTTGAAGCAGACAAGTGTAAATCGTCCTGTTTTGATTGTGTTAGATGATGTGTGGAGTGGGCAAGAATCTGAAGTTCTTCTTGATAAGCTGTTTCAATTGCCTTGCTGCAAGATCTTGGTCACTTCTAGGTTTTATTTCCCAAGATTTAGTGAGTCTTATTATTTGGAACCTTTGAACCATGAGAATGCAGTACAACTTTTTCGTCGTGCAGCATCACTGGACAAAGGAATTTCTAAGCTCCCCGATGATGAAACTGTAGAAAAGATAATTGGGGGATGCAAGAGACTACCTCTTGCACTGAAGGTAATCGGGAGGTCTCTTTCCCACAAACCGACATCTGTTTGGAAAGTAACGGGGAGGAATTTGGCTAGAAGTGGCTCCATATTTGATTCTGACAATGAACTTCTTGAATGCCTTCAGAGCAGTTTGGATGTCTTGGATGATAACATGGTAACTAAGAAGAGTTTCATGGATTTAGGCTCTTTTCATGAAGATCAAAGAATTTCTGCTTCTACCTTCATTGACATGTGCACAGTTTTGTACACACTAGACGAAAGTGAAGCAATGGTTACCCTTGACGAACTATCCTCTCGAAGTCTAGTTAATTTTGTCACAGCGAGAAAATATGGATATGATGATGACTTTTATGAAGAGTACTCTTTTACTCAGCATGATATTCTCAGAGATTTGGCTATTCACTTGATGAATATGGAGCCCATAGAACAAAGGAAAAGATTGATCTTAGACATTAATGGAAATGATCTTCCCAAATGGTGGGTTGATCAAGAAAAGCATACTTCCTATGCTCGCCTTATATCCATAACCACAGATAAGAGATTCTCAGCAAGTTGGCCTGACATGGAAGCACCTGAAGTGGAGGTTCTGATTCTTAATCTTCAGTCAAGAACTTACAACTTGCCTGGGTTCATCAAAAGAATGAATAAGCTGAAAGTTTTGATAATCACATATTTTGGTTCTTTTCTAACTGAGGTGACAAGTGAAGATAATCAACTACTCGACAGCCTAACAAGTCTTGAACGAATCAGGTTTGAGCGGATTTCAGTTCCTATCTTTAGTAATCCAAACCCGAAACCACTGATAAATCTGCAGAAAATATCCTTCTTTATGTGCAAATTTGGTCAAACATTCATGGATCCTTCAACCCCAATCTCAGATTTGTTGCCAAACCTGCTGGAGATTTCCATAGACTTCTGCAACAATTTGAGTGAAGTCCCCAATAGGTTGTGTGAAATTGTCAGCTTGCAGAAGCTGAGCATTACAAATTGCCATGGACTATCTTCCTTGCCAGAAGATGTAGGGAAGTTGATTAATCTAAAAAATCTAAGGCTAAGATCTTGCATTCATTTAGAAGAGTTTCCAGAGTCGACAACGAAGCTTCGGGAATTAGTCCTGCTTGATATATCTAACTGTATTGGTCTTGCCAAGCTTCCCGAGAAGATTGGTGAATTTCATAATTTAGAAAAGCTTGACATGAGACACTGCTGGAGTTTGAGCAAGCTGCCACTGTCGATTGGAAAGCTGAAAAATGTGAAGTTTTTATGTGATAGAGAGGTTGGAGAGTGGTTGAGAAAGGTTGCACCTCGCCTTGCCAAACAGGTGAAAGTGCAAGAGGAAGAAGCCAACCTGGAGTGGCTTGGTTTTTGA

>Cucsa.123410

ATGGCGGTTACAGATTTCTTTGTTGGAGAGATAGCCACTGAGCTTCTCAGAATGATGGTACAACTTTCGACCAAATCCTGCCTTTGTAAAACGACGGCAGCTCAAATCGCCAATTCTATTCAACAAATTCTGCCGATTATTGAAGAGATCAAGTACTCGGGAGTTGAATTACCCGCTCATCGCCAATTTCAGTTAGATCGCTTCAGCGAAACTCTTAGAAGAGGCATCGAGATTTCCGAGAAGGCTCTTCAATGTGGCCGATTAAACATTTACAGAAACTTACGGCTCGCGAGGAAGATGGAGAAGCTTGAAAAGGATATATGTCGATTCATTAATGGCACCATGCAGGCGCATATACTGGCCGACGTGCATCATATGAGATTCCAGACCACCGAGCGGTTTGACCGGCTTGAAGGTGTTTTGTTGGAGCGGCGGCTTGAGTCGATGAAGATTAGAGCAGATGCTTCGGGAGAGGAAAGGTGGTGGGTTGAGGAGGCGTTTAAGAAGGCCGAGGAGGAGGAAAGGTATGAGAGTAATTTCGTGAATATAGGAACTGGATTGCGTGTGGGGAAGAGAAAATTGAAGGAGCTGGTGATTGGAAAGGAGGATTTAACGGCGGTTGGGATTAGTGGAATTGGGGGTTCGGGGAAGACTACTTTAGCTAGAGAATTCTGCAAAGATCCGGAAGTTCGAAGACACTTTAAAGAGAGAATTTTGTTCTTAACGGTGTCACAGTCCCCTGATGTGGAGCAGCTGAGGAGAACGATCTGGGAATTTGTGATGGGTAGTGATAGTGTCAATTCTAATAATTTGATTTTACATGGGAGGCCTTCAAATTCAGCGCTTTTGGTTCTGGATGATGTGTGGTCAATTTCAGTTCTTGAAAATGTTATTCCAAACGTAACTGGTTGCAAAACTCTTGTTGTTTCACGATTCAAATTCCCTGAAGTTCTTAGAGAAACTTATGAAGTAGAGTTGTTGAAAGAAAGTGAAGCAATTGCTCTGTTTTGCCACTCAGCTTTCGGACAACAGTCGATTCCTTTGTCTGCTAATCACAACTTGGTCAAACAGGTTGTGAATGAATGCAAATGTTTGCCTCTGGCTCTTAAAGTCATAGGAGCATCACTCAGAGGACAGAGCGAGATGTTCTGGAATAATGCCAAGTCTAGGTTGTCACGTGGCGAGCCTATTTGCGAGTCCCATGAGAACAAATTGCTTCAAAGAATGGCAATCAGTATTGAACGCCTCTCGAGTAAAGTGAGAGAATGTTTCCTCGACCTGGGATGCTTTCCTGAAGACAAAAGAATTCCTCTTGACATTCTCATCAATGTTTGGAAGGAGTTACATGATCTTGATGACGAAGAAGCTCTTGCTGTTCTTTTCGAGTTATCTCAGAAGAATCTTCTTACGTTGGTGAAAGATGCACGCGGTGGTGACATTTATAGCAGTTATTATGAGATGTATGTCACTCAACACGATGTATTAAGGGACCTTGCCCTTCATTTCAGTTGCCAGGAGAATGTGAACGACCGCAAGCGATTACTGATGCCAAAAAGCGACACAGAGCTTCCAAAAGAATGGTTAAGGAAATCGGAACAGCCATTTAATGCCCAACTTGTTTCAATTCACACAGGTGAAATGGAAGAAATGGATTGGGCGCCTATGATATTTCCTGAAGCTAAAGTGCTCATTTTAAACTTCTCCTCGAGTGGATACTTCTTGCCTTCTTTTCTTTGCAACATGCCGAAGATAAGAGCATTAATTGTGCTAAATAACAATGCAACACATGCAACTCTCACCAATTTCTCAGTTTTTTCTAGTTTGGTCAACTTGAGAGGCATCTGGCTGGAAAAAATTTCCATGACACAACTATTCGATGCTTGCACGCCATTGAAACATCTAAGGAAGCTATCTCTTGTTTTCTGCAAGATCAACAACAGCCTCGACGAGTGGGCGGTAGATGTATCCCAGATCTTCCCGTTTCTTTTCGAACTCAAAATTGATCACTGCAACGACTTGCGTAAGCTACCTTCAAGCATTTGTGAGATGCAAAGTCTCAAGTGTCTTAGTGTCACCAACTGTCATAATCTCAGTCAACTCCCTACCAACTTATGGAAGCTGAAAAATCTACAAATCTTGAGACTTTTTGCTTGCCCACTCCTCAAAACTCTATCCCCAAGCATTTGTGTACTTTCTTGTCTAAAGTACATTGACATCTCCCAATGTGTTTACTTAACCAGCCTTCCTGAAGAAATTGGCAAGCTGACAAGCCTAGAGAAAATTGACATGAGAGAATGCTCACTCATAAGGAGACTACCTAGATCAGTTGTGTCTTTGCAATCTCTCTGTCACGTAATCTGCGAAGAAGACGTCTCGTGGCTATGGGAGGATTTGAAGAGTCATATGCCTAATTTGTACATTCAAGTCGCCGAGAAATGCTTCAACTTAGATTGGCTCAAAGAGTGA

>Cucsa.128030

ATGGCCGAAGCAATTCTCTTCCAAGTTGCTGGGGAGATCTTGATGAAGCTAAGCTCTCAAGCTTTCCAGCGTCTTGGGATGCTATTTGGGCTGAAGGGTGATCTTAACAAACTCACAACAACTGTTTCCACCATTAAGGATGTGCTTCTTGATGCGGAGGGACGTCAAACTAAAAGTCACTTGCTGCAAAATTGGCTCCATAAGCTGGAAGAAGCTCTTTATGATGCAGAGGATGTGCTTGATGAACTCTCTACGGAGGCTCTCCGTCGAGAACTGATGACTAGAGATCATAAAAATGCAAAACAAGTAAGGATCTTCTTCTCCAAATCTAATCAAATTGCATTTAATTATAGGATGGCTCGTCAAATAAAGAATATTTGGGAGAGGCTAGATGCTATTGATGCTGAAAAAACACAATTTCACTTGCGTGAAAACTGTGAATCACGGACTCAATACGGTTCATTTGATCGAATAATGATGGGAAGGGAAACTTGGTCTTCTTCAAATGACGAGGAAGTGATTGGAAGGGATGATGATATAAAAGAAGTAAAAGAGCGTTTATTGGATATGAATATGAATGTCACGCATAATGTTTCGTTCATTGCTATAGCTGGAATGGGTGGGATAGGCAAGACGACCTTGGCTAAATCTCTCTACAATGACGAAGAGGTATCAGGATTTTTCGACTTAAAAATATGGGTTTGGGTTTCTGATCAATTTGAGGTACAAGTGGTAGCGGAAAAAATGATAGAATCAGCAACCAAAAACAATCCTAGTGTAAAAGGAATGGAAGCTTTACAAGCAAAGCTTCAGAAAGTGATTGGAGAAAGGAAGTATCTGTTAGTTATGGATGATGTATGGAATGAAAGTGAAGAGAAATGGCATGGGTTGAAATCATTGTTGATGGGTGGTGCAAGAGGGAGTAAGGTTTTGATCACAAAGCGTGACAGAAAAGTAGCCACAGAAATCAAAAGCATGACATCTTTGTTCACTTTAGAAGGCTTATCAGAGAGTAATTCCTGGTTATTGTTTAGTAAAGTGGCATTTAAAGAAGGCAAAGAGTCCACAGATCCAAGCACGATACATTTAGGAAAAGAAATTTTAGTGAGATGTGGAGGTGTTCCTCTTGTTATAAGACATGTTGGACGCATGTTATACTCTAAAACTTCACAAGAAGAGTGGATGTCCTTCAAGGATAATGAACTTTTAGAAGTCATTCAACAAGACAATGATATGACATCAATATTAAAATTGAGTTATAACCATCTCCCACCAAATTTGAAACGATGTTTTGCATATTCATCCCTGTTTCCCAAAGGATATAAAATAGAAATAAAAGACCTAATAAGGCAATGGGTGGCTCAAGGTTTTATTGAAGTGTCAAATGGAAGAAAATCCTTGGAAGATACAGGGAAGGACTATTTTAACGAATTATGTTGGAGGTTTTTTTATGCAAATTCTAGTGATGAGTGTAACATCAATGATATTGTTTGTATGCATGATGTGATGTGTGAGTTTGTAAGGAAGGTGGCAGGAAATAAATTATATGTACGTGGAAATCCCAATAATGATTATGTTGTCAGCGAACAAACACTTCACATTTCATTTGACTACGGAATACAATCATGGCAAGATGTTCTATCTAAATTATGCAAGGCTAAGGGATTAAGAACAATCCTTTTATTATTTCGTCCCTACGAGAAAATGAATAAAATTGATAAAGCTATTTTGGATGAATTATTTTCCAGTTTTCCACGTTTGCGAGTATTAGATCTTCATTTCTCGCAGATTTCTGTAGTGCCGAAGTCTATAAAAAAACTTAGACACCTTCGATATTTGGATCTCTCTGAAAATGATATGGAATTAATTCCACATTCTATCATTGAATTGCAAAATTTGCAAACACTAAATCTAACAGAATGCTATGAGCTAAAAGAATTGCCAAGGGACATCGACAATCTTGTAAATCTCAGGCATCTTACCTTTGAACCTTGTATGGAAGTAACTCCTACATCGGAGGGGATGGAGAAGTTGACTTGTCTACAAACAATCAGTTTATTTGTGTTTGACTGCAAAAAGACCAATAAGCTATGGGAATTGAATGATCTCAGTTATTTGACAGGAGAGTTAAAAATCATAGGTTTAGAGAAGTTGAGGTCTTCTCCATCTGAAATCACCTTAATAAACCTGAAAGACAAAAAAGGTTGGCAAGGTTTAAATTTGGAATGGAAACTGGGCAAGGATGAATACGAAGGTGAGGCTGATGAAACAATAATGGAAGGCTTGGAACCACATCCAAATGTTGAATCGTTGAGCATTAACGGGTACACTGGAGGAGCATTGCCCAATTGGGTGTTCAACTCGCTTATGAAGTTAACTGAAATTGAAATTGAAAATTGCCCTAGAGTGCAACATCTACCTCAGTTCAACCAGCTTCAGGATCTCAGAGCTCTACATTTAGTGGGCTTAAGATCTCTCGAGTTCATAGATAAGAGTGATCCATACTCATCATCAGTGTTTTTTCCATCTCTCAAGTTTCTACGTTTAGAAGATATGCCTAATTTGGAAGGATGGTGGGAATTAGGGGAATCAAAAGTAGTAGCAAGGGAGACATCTGGGAAAGCTAAATGGTTGCCTCCAACTTTTCCTCAATTAAGTTCCATGCCCAAGCTAGCTTCTATTGGAGCAGATGTTATTTTACATGATATTGGGGTTCAGATGGTGAGTACCATAGGTCCAGTATCGAGTTTTATGTTTCTATCAATGCATGGAATGACGAATCTCAAATATTTATGGGAGGAATTTCAGCAAGATCTAGTTTCTTCAAGTACCTCAACAATGTCCTCACCTATTTCCCTTCGTTATCTGACAATAAGTGGATGCCCCTATCTCATGAGTTTACCGGAATGGATTGGCGTTCTCACTTCCCTTGAAACATTGCATATTAAAGAATGTCCAAAATTAAAATCACTACCAGAAGGAATGCAGCAACTCAAATCTTTGAAAGAACTTCACATAGAAGACTGCCCTGAACTAGAGGACAGATGCAAGCAGGGAGGAGAGGATTGGCCAAACATTTCCCACGTTCCCAACTTTACTTACAAAAATGCCTCTGACATTGACACACCACAATCTTCTTCAGGTTTTTCACACCATCCCTTTTCAATCGTTCGTATCTCTGTTATATAG

>Cucsa.128100

ATGGCGGATTCAGTTCTGTTCAATGTTGCTGCAAGTGTTATTACTAAACTGGGATCTTCCGCACTTCGAGAACTTGGGTCTCTGTGGGGAGTCAACGATGAGCTCGACAAACTCCAAAACACTCTTTCGGCCATTAAAGCCGTCCTTCTCGATGCAGAGGAGCAACAGTCCAAGAGCCACACAGTCAAGGATTGGATTGCAAAGATCAAAGATGTTTTCTATGACATTGATGACTTGATTGACGAGTTCTCTTATGAAACCTTGAGAAGACAAGTTCTTACCAAGGATAGAACAATCACCAAACAAGTACGTATCTTCTTCTCCAAATCTAATCAGATTGCTTTTGGTTTCAAAATGGGTCAAACAATTAAAAAAGTTAGGGAGAAGCTAGATGCTATTGCGGCTATTAAAGCTCAACTTCACCTCTCTGTGTGTGCGAGGGAGGTACGAGATAATGAGCCAAGGAAGGTACGAGAGACGTCCTCATTCATACCCGAGGGGGAAATCATTGGTAGGGATGAGGATAGGAAATCTGTTATGGATTTTCTATTGAATACCAGCAACATCACAAAGGATAACGTTGAAGTTGTTTCCATTGTTGGAATGGGAGGATTAGGAAAGACAGCACTCGCTCAAACTGTCTATAATGATGAAAAAATAAACAATCGTTTTAAGTGGAAAATATGGGTGTGTATTTCTCAAGAATTTGATATCAAAGTAATTGTTGAAAAGATTTTAGAGTCTATTACGAAAACAAAACAAGAATCCCTTCAGTTGGATATATTACAAAGTATGCTTCAAGAGAAAATTTATGGAAAAAAATACTTGTTGGTCATGGATGATGTGTGGAATGTAGACCACGAGAAATGGATTGGTCTGAAAAGATTTCTGATGGGTGGTGCCAGCGGAAGTAAGATTTTGGTGACAACCCGTAATCTACAAACTGCCCAGGCTTCTGACACGGTTTGGTTCCATCACTTAAAAGAACTTGACAAGGATAACTCTTGGGCGTTGTTTAGGAAAATGGCATTCTTAAACAAAGAAGAAGAGCTTGAGAATTCAAATTTGGTTAGAATCGGTAAAGAGATTGTAGCAAAGTTGAAAGGTTATCCCCTTTCAATAAGAGTTGTTGGACGTCTGTTATATTTCAAAAACACAGAAATGGATTGGTCATCATTTAAGGACAACGAACTTGACTCAATTTTGCAAGAAGATGATCAAATTCAACCAATACTGAAGATAAGTTTTAACCACCTTCCACCTAAATTGAAGCAATGTTTTACGTACTGTGCTTTGTTTCCCAAGGATTATGAGTTTAAAAAGAATGGATTGGTAAAACAATGGATGGCACAAGGTTTCATTCAAGCACATAATAAAAAGGCAATTGAAGATGTGGGTGACGATTATTTTCAAGAGTTAGTGGGGAGGTCATTCTTTCAAGACATAAGAAAAAACAAATGGGGAGACTTAAAGTACTGTAAGATGCATGATTTGTTACATGATCTTGCGTGTTCGATAGGAGAAAATGAATGTGTGGTTGTAAGTGATGATGTCGGGTCCATTGACAAAAGGACTCGACATGCCTCATTTCTCTTGAGCAAGAGGCTAACAAGGGAAGTTGTATCAAAATCATCCATTGAGGTAACGAGTTTGAGAACATTGGATATTGATAGTCGTGCTTCTTTCCGTTCTTTCAAGAAAACTTGTCACATGAACCTTTTTCAATTACGAACATTGAATTTGGATAGATGCTGCTGTCATCCTCCTAAGTTTGTTGATAAGTTGAAACATTTGAGATATCTTAATCTTTCTGGTTTAAATGTAACTTTCCTTCCCAATTCTATTACCACATTGTATAATTTGGAAACACTTATCCTTCGTTACTGCCTTTGGCTAAGAAAATTGCCAAAAGATATTAACAATTTGATCAATCTCAGGCATCTTGATATTTATGATTGTTCCAGTTTGACTCACATGCCAAAAGGATTAGGTGGGATGACTAGCCTTCAGACAATGAGTATGTTTGTATTAGGAAAGAATAAAGGTGGTGATTTAAGTGCATTGAATGGACTTAAAAGCTTGAGAGGATTATTATGTATTAAAGGTTTACAATTTTGCACAACTGCTGATTTAAAAAATCTGGAATTACACTGGGATATAAAAATGGATCATGAAGATGCCTTAGATGATGGTGATAATGATGATGAGGGAGTTTTGGAGGGCTTAAAACCACATTCAAATATTCGCAAAATGATTATAAAAGGATACAGAGGAATGAAGTTATGTGATTGGTTTTCTTCTAATTTCCTGGGTGGTTTGGTTAGCATAGAGCTTTCACATTGTGAAAAATTGGAGCATCTCCCACAGTTTGATCAATTCCTATATCTCAAGCATCTTCTTCTTGGATACTTACCCAATATTGAATACATTGATAGCGGCAATTCTGTTTCTTCATCAACAACATTTTTTCCATCTCTCGAGAAGCTAAGGATTGAGAGCATGCCTAAGTTGAAAGGGTGGTGGAAGGGGGAAATTTCATTTCCAACAACAATATTACATCAACTCTCAGAATTATGTATTTTTTATTGTCCTCTGTTGGCTTCTATTCCACAACATCCATCTTTGGAATCATTGAGAATATGTGGTGTTAGTGTGCAACTTTTTCAAATGGTAATACGAATGGCTACAGACCTTTCTGAACATTCTTCTTCTTCTTCAACATTGTCTAAATTATCTTTCCTTGAGATTGGAACTATTGATCTTGAGTTCTTGCCAGTGGAGTTATTCTGCAATATGACACATCTTGAGTCTCTTATCATAGAACGCTGCAAAAGTTTACAAATGTCTTCTCCGCATCCTGTTGATGAGGATAATGATGTGGTATGGAAAAAACTCAGCAATCTCCGGACACTTCGGCTTGAGAGCATCCTCAAATTGGAGTATTTTCCCAAGAGTTTGAAATATATTACAAGTCTTGAAACTTTGAAGCTATCAAATTGTGAAAATTTAGTGAGTACGGAAGGGATTGGCGAACTCATTTCACTATCACATTTGGAAATTGATAGATGTCCTAATTTACCTATATTGTCGGAAGATGTCGGCGACCTCATTTCCCTATCACACTTGCTTATTTGGAATTGTCCCAAATTAACTTCCTTGTCAGAAGGAATCACTCGCCTCACTTCACTCTCAAGTTTGTGTCTTGAAGATTGTCCCAACTTAGTCTCCTTGCCCCAAGAATTTCTCCACCACCACAGCTCCTTACCAGGAGGACGGTTCTTGAGAATTTTGAACTGTCCCAAATTGCAGATTCAAGACAAGAAACAAAAGGAAGAAGAAGAAGAAGACCAGGAGGATTGGAATGAACTCATCCATGTATTGACCGGATGTAGGTAA

>Cucsa.128110

ATGGCGGATTCTATTTTGTTCAACGTTGCTGCAAATGTTATAACCAAATTGGGCTCTTCCGCGCTTCGAGAGCTTGGATCATTGTGGGGAGTTAATGATGAGCTTGGAAAACTACAAAACATTCTTTCAGCCATCAAAGCTGTGCTTCTCGATGCAGAGGAGCAACAATCAGTGAGCCACGCAGTCAAAGATTGGATTTCAAAGCTTAGAGATGTTTTCTACGACGTTGATGACTTGATTGATGAGTTCTCTTATGAAACCTTGAGAAGACAAGTTCTTACCAAAGATAGAACAATTACCAAACAAGTATGTATCTTCTTCTCCAAATCTAATCAGGTTTCATTTGGTCACAAAATGAGTCAAAAAATTAAACAAGTTAGGGAGAAACTAGATGCTATCGCTAATGATAAAACTCAACTCCACCTTTCTGTCCGTATGAGGGAGACACGAGATGATGAGTTGAGAAAGATGCGAGAGACTTGCTCTTTTATTCCTAAGGGAGAAGTGATTGGTAGGGATGATGACAAGAAAGCTATTATAGATTTTCTATTGGATACCAACACCATGGAGGATAATGTTGAAGTGGTTTCCATAGTTGGCATGGGAGGATTAGGAAAGACTGCAGTTGCTCAATCTGTTTATAATGATGAGAAGATAAATGAACATTTTAAGTTGAAATTATGGGTGTGCATCTCTCAAGAGTTTGATATCAAAGTAATTGTTGAAAAGATTATAGAGTTTATTGCGAAAAAGAAACCCGATTCTCTTCAATTGGATATACTACAAAGTATGCTTCAAGAAAAAATTGATGGAAAGAAATACTTGTTGGTCATGGATGATGTGTGGAATGAAAGCCATGAAACATGGGTTAGTCTAAAGAGATTTTTAATGGGTGGTGCCAAGGGAAGTCGGATTTTGATCACAACACGTAATCTGCAAGTGGCACAGGCTTCTGATACAGTTCAGTTTCATCACTTAAAAGAACTTGACAATGAGAGCTCTTGGGCGTTGTTTAGAAAAATGGCATTTTTGAATGAAGAAGAAGAGATTGAGAATTCAAATAAGGTTAGAATCGGTAAAGAGATTATAGCAAAGTTGAAAGGTTCTCCTCTTACAATAAGAATAGTTGGACGTTTGTTATATTTCAAAAACACAGAAATGGATTGGTTGTCATTCAAGGACAACGATCTTGGCACAATTTTGCAACAAGAAAATCAAATTCAACCAATATTGAAGATAAGTTTCAACCACCTTCCATCTAATTTGAAGCACTGTTTTACATATTGTGCATTGTTTCCGAAAGACTATGAGTTTCAAAAGGATGGATTGGTAAAGCAATGGATGGCACAAGGTTTCATTCAATCACATAGTAATAAAGAAATTGAAGATGTTGGTGATGATTATTTTAAAGAGTTGTTGGGCAGGTCATTCTTTCACAATGTAAAAGTAAATAAATGGGGAGACGTCAAGGAGTGCAAGATGCATGATTTGATACATGATCTTGCATGTTGGATAGTAGAAAATGAATGTGTGGATGCAAGTGATAAAACTAAGTCAATTGATAAAAGGACTCGACATGTGTCATTTCCCTCCAATTATTCAAGGAAAAGTTGGGAACTTGAAGCAAAATCATTGACTGAGGTAAAGAATTTGAGAACTTTGCATGGTCCTCCATTTCTTCTATCTGAAAATCATTTGCGATTACGGTCATTGAATTTGGGGTACAGCAAATTTCAGAAAATTCCCAAGTTCATTAGTCAGTTAAGACATTTAAGATATCTTGACATTTCTGATCATGATATGAAATTTCTCCCAAAGTTTATTACGAAATTGTATAATCTGGAAACACTTATCCTTCGTCACTGCAGTGATCTAAGAGAATTGCCAACCGATATTAACAATTTGATAAATCTTAAGCATCTTGATGTACATGGTTGCTACCGTTTGACTCACATGCCAAAAGGACTGGGTGGGTTGACTAGCCTTCAAACGATGAATTTGTTTGTATTAGGGAAGGATAAGGGTTGTGATTTAAGTGAGTTGAATGAACTTGCTAGGTTAAGAGGATCCTTGCTTATTAAAGGATTAGAACTTTGTACCACTACTGATTTGAAAAATGCTAAATATATGGAAGAGAAATTTGGAATTCAGAAGCTGAAACTACGTTGGAATAGGGATCTGTACGACGCTGAAACTGACTATGCATCAGAAAACGATGATGAGAGAGTTCTAGACTGCTTAAAACCACATTCAAATGTCCACAAAATGCAGATAAGAGGATATAGAGGAGTAAAGTTGTGTAATTGGCTATCTTTTGATTATTTAGGCGGTTTGGTCAACATAGAGCTTCAAAGTTGTGAAAAGTTGCAGCATCTCCCTCAATTTGATCAATTTCCTTTTCTCAAGCATCTTCTTCTTGAAAACTTACCCAGTATTGAGTACATTGATAATAACAATTCTCTTTCTTCATCAACTTTCTTTCCATCCCTTGAGAAGCTAACCATCATGACAATGCCTAACTTGAAAGGATGGTGGAAGGGGGAAACCCCCCCGGAATCTGCTCGTTACAGTGCCTTGTTTCCAACAATATTACATCACCTTTCTCGATTAGATATTTCTAATTGTCCTCAGTTGGCTTCTATTCCACAGCATCCACCTTTGCGATCATTAGCATTGAATGATGTTAGTGTGCAACTTTTTGATATGGTAATAAAAATGGCTACAACCCCTGCTGCTGATTCTTCTTCAGCTTTGTCTAAATTATCTATTCTTCACATTCAGAATATTGATCTTGAGTTTCTGCCAGAGGAGTTGTTTGGTAGTACGACAGATCTTGAGATTTTTACCGTAGAGTTGAAATATATGACAACTCTTGAACGTTTGGATCTATATAATTGTCCTAATATAGTGAGTCTTGAAGGGATTAGCCACCTCACTTCTCTGTCAAGCTTGAGAATTTGTAATTGTAGCAATTTAACTTCGTTGCCAGAAGGGATCAGCCATCTCACTTCACTATCATATTTGACGATTTATTGTGTCAATTTAACTTCGTTGCCAGAAGGAGTCAGCCATCTCACTTCATTATCAAGTTTCACTATTGAGGAATGTCCCTGTTTAACTTCATTGCCAGAAGGGGTCAGTCACCTCACTTCACTGTCAACTTTGATAATTAGGCGTTGTGTCAATTTAACTTCGTTACCCGAAGGGATCGGCCATCTCACTTCACTGTCAATTTTCACTATTGAGGAATGTCTCAATTTAACTTCGTTGCCAGAAGGACTTTTACACCTGTCCTCCTTGCGAGGATCATTGACAGTTTCAAAATGCCCCAAGTTATCGAAGACGTGGAAGAAGCTAAACAAGTAG

>Cucsa.128130

ATGGCAATTGGTGACCCTCAACTTCCTATCCTACATCGTGCACCTGTTGATAAAAGCCATGGCATAAAAAAGCTTAGCTCTCATGCTTTAGAATGCCTTGGAATGGTATGTGGTCTTAATGATGATCTTAACAAACTAAGGAGCAATGTTTCGTCCATTCAATCTGTACTTCGTGATGCAGAGCAACGTCAAATCAAAGGCAATGATCATTCTTTAACCGATTGGCTCGAAAAGTTGGGAGACGTTTTTTACGATGTTGAGGACGTTCTTGATGAAATCTCTACTGAGGCTCTCCGTCGAGAAGTGATGACAAGAGGAAAAAATGCAAAGCAGGTTAGAATCTTCTTCTCCAATTCTAACCAACTTGCATTTAACTATAGGATGGCATGTCAAGTCAAGAAAATTAATGAGAGGCTTGATGTTATTTCCCAAGAAAAAGATAAGTTTCAGCTCAATGGAATTGCTTATCTTGGGATACAAAATGTTTTATCTTATCCAATTGGGATGGAAAGGGATACTCACTCATCTTTAAGTGGGGATCAGAAAATAATTGGAAGGGATGATGAAATGAACAACCTTAAAAAAAATTTACTAGCAGAGGATGACAAGGTGAAAGCTAACGTTTCATTCATCGCTATTGTTGGAATGGGTGGAATTGGCAAGACAACTTTGGCCAAATCTCTCTACAATGACAAACAAGTCTCTGATGGTTTTAGTTCAAGAATTTGGATTTGGGTTTCTAATCAATTTGACACAAAAACAATATTGAAAAAGATAATTGAATCAGCAACCGAAAAGAAACCAAAGGTAGAAGAAATGGAACCTTTAAAGACAAAGCTTGAAGAAGTGATTGGAGGAAAGAAGTATTTGTTAGTTATGGATGATGTATGGAATGAAAATGAAAATGAATGGGAGAATTTGAAAAACCTGTTAATGCTTGGTGCAAGAGGGAGTAAGGTTTTGATCACAAAGCGTGACAGTAAAGCAGTTCCAGGAGTCGAAACAATTCCTCTAAAAGACTTAACTGAAGATTTTTCTTGGTTGTTGTTTAAAGAAGTGGCATTTAAAGAAAGTGACTTAGAGTCAATAAATCAAAACTTGATAAAAATGGGAAAAGAAATTTCAAAAAGATGTGGAGGTATTCCTCTTGTAATAAGACACATAGGACGTTTATTATATGGAAAAACTTCCGCAGAAGATTGGGAGTTCATCAAAGAAAATGAACTTTTAAATGTCACTCGTGAAAAAAATAATAATGATGGTCATGTGATATCAACATTAAAATTGAGCTATAACCATTTGTCACCAAATTTGAAGCAATGTTTTTCCTATTCATCCTTGTTTCCCAAAGGATACAAAATTAGAATGAATGAATTGATTAGACAATGGATAGCTCAAGGTTTTATCGAATCATCAAATGGAGGAAAATCTGTAGAGAATATTGGGAAGGAGTACTTGGATGAATTATGTTGGAGGTTTTTCTATGAAATTTCTATTGAGGATGTTCCTTTTGAAGAAGTTGGCATGCATGATTTGATGTGTGATCTTGCAAGAGAGGTAGCTGGACAGAAATTGTACATACGTGGATATCCAGAGAGTGGATATGTTGTGAGTGAACAAACTCGTCATATTTCATTTGAATATGAACCACGATCATGGATTGATGATGTGTCCAAATTGCAACAAGCTAAAGGATTAAGAACGTTCCTTTTGTTTACGAAAAATCCTTTCTTTACGAGAAATCCAATTGAAAAAGTTCTTTTGGACAGACTGTTTTCTCACTTTCCACGTTTGCGAGTATTACAAATCCCTAATGTGTCAAAGTCAATAAAAAAGCTTAGACATCTTCGATATCTAGAACTCGGTGAAGATGCGAAATCAGTTCCAAACTCCATCACGAAATTGCAAAATTTGCAAACACTAGATCTAACCAAGTGTTATGACCTAAAGGAGTTGCCAAGGGATATTAACAATTTTGTAAACCTCAGACATCTTCTTTGTGATTCAAGATTAATGAATATGCTGCAAGGGACGATGGAGAAGTTGACTAGTCTACAAACATTAAGTTCATTTTTGTTTGATTGTAAAAGGTTTGATAAGGTAAAGGAATTCAGTGAGCGGAGTTATTTTATAGAATTTGACTTAAAAATCAAAGGTTTGGAGCAGTTGAGGTTTTCTCCATCAGACGTCAAATCAGTAAATCTTAAAAACAAAAAAGTCCCACTTTTGAGACTGAAATGGAAATTTGAGAATGGTAATGAATATGAAGGTGATGCTGATGATATAGTATTGGAAGGCTTAGAACCACATCCATATGTTAATCTCTTGCAAATTGAAGGGTATTGTGGAGTAGGATTACCCAATTGGGTGTCCACCTCAATTTTGTTAAGGGGAATTCGAATTGGTAATTGTGATAGATTACATCTGAATCAACTCTCCCATCTTCATGCTCTTGAAATTCTAAATTTAGAGGGTTTAAAATCTGTCATGAGTATATCGGAATGGATTGGCACCCTTACTTCTCTTGTATCTTTGGAAATAGAAGAATGTCCAAAATTAAAATCACTTCCAAAGGAAATGCAACAGCTCAAATCTTTGGTGCAACTTAACATAATCAAGTGCCCACAACTTGGGGAGAGATGCAAGGAGGGAGGAGAGGATTGGCCTAACATTTCCCATATTCCTGACGTTCTTATTGATTGA

>Cucsa.128140

ATGGCTGAAGCTATTCTCTACAACGTTACTGCAGACATCATATTCAAATTGGGCTCTTCCGCACTACAGGAGCTTGGGTTGTTGTGGGGTGTCAATGATGAACTCGACAAACTCAAACACTCGCTTTCTGCCATTCAAGCTGTGCTTCTCGATGCGGAGGAGCAGCAGTCCAAGAGCCTTGCTGTCAAGGCTTGGGTTTCAAGGCTTAAGGATGCTTTGTACGAGATTGATGACCTGGTGGACGAGTCCTCCTACGAAACCTTAAGAAGGCAGGTTTTGGCCAAAGATCAGAGAAAAAGAAAACTAGTACGTATCCTCTTTTCCAAATTTAAATCTAATTGGAAAATAGATCACAAAATCAAGGATATTAGACAGAGGCTACAATCTATTAATGATGACAAAAATCAATTTAGCTTTTCTGAGCATGTGATCGAGAAAAGAGATGATGAAGAGTTGAGAAAGAGACGGGAGACTTACTCTTACATACTTGAAGAGGAAGTGATCGGTAGGAATGATGACAAGGAAGTAGTCATAGATCTTCTATTAAATTCCAACATCACAGAGGATATTGCAATTGTTTCCATTGTTGGAATGGGAGGACTGGGAAAGACTGCCCTTGCTCAGTCTATTTATACCCATCACAATATGACTAATAGTGGGTTTGAATTGAAGTTATGGGTGTGTGTTTCTGAAGAATTTGATCTAAAAGTTATTATCCAAAAGATGATAGAGTCTGCAACTGGGACGAAGCCTAAGCCGTACCTTCAAATAGATTCATTACAAAGTGAGCTTAGAAAGAAAATCGATGGAAAGAAATACTTATTCGTAATGGATGATGTGTGGAATGAGAAAAAAGAGGAATGGTTACGCCTTAAAAGATTATTGATGGGCGGTGCAAAGGGTAGTAGGATTTTGATCACAACACGGAGTGAACAAGTTGCTAAAACTTTTGACTCTACTTTCATCCATTTTTTACAAATTTTGGATGAGTACAATTCCTGGTTATTGTTTCAAAAAATTACTTGTTTGGAAGGACATCCAAGTAATCCAGAGAAGCTTGATCAAAGTTCAAGTTTGATACAAATTGGCAGGGAAATCGTTTCAAAGCTAAAAGGTGTTCCTCTCACGATAAGAACCATCGGAGGACTTTTAAAAGACAATAAATCAAAAAGAGTTTGGTTGTCTTTCAAAGATAATGAACTTCATCGAATTTTGGGGCAAGGACAAGATAATCTAAAAGAAGTGCGATTAATTCTTGAACTCAGCTATAAATACCTTCCAGCTAATTTGAAGCAATGTTTCCTATACTGTGCTTTGTTCCCGAAAGATTATGAAATTAAAACACATGAACTTATACTAATGTGGAGTGCGCAAGGTTTCATTCAACCAAATGGCAGCAAGGACAACAGCCTCATTGATATTGGCAATGATTATTTCATGGAGTTATTATCACGATCATTTTTTCAAGAGGTTACAAAAAATGAACGGGGAGACATAATAGCATGTAAGATGCATGATTTGATGCATGATCTTGCTTGTTGGATAGCAGATAATGAATGCAATGTCATCAACATAGGAACTCGTCACTTTGCATGGAAAGATCAATATTCTCATAAAGATCAACTTCTAAGATCATTATCAAAGGTGACAAATTTGAGAACATTTTTCATGCTAGATTCTGCAAATGATTTGAAATGGGAATTTACAAAAATACTTCATGATCATTTGCAATTACGAGCCTTGTATTTCAAAAATTTGAAGAATGCAATGATCGTTTTGGAGTTTACTGGTAAGTTGAAACATTTGAGATATTTGAGTATTATGGACTCATTTATTTTAAATCTTCCAGATTCCATTACAGAATTGTATAATTTAGAAACACTGATCCTTCGGAATTCCAGTTTTAAAATGTTGCCCGATAATATCGGCAATTTGATCAACCTCAAGCATTTAGATCTTTCTAATAATCGAAATTTAAAATTCCTGCCAGATTCTATTAGTGACTTGTGTAAATTGGAAGAACTCATCCTTCATGGTTGTTTGAGATTAGAAGAATTCCCAGAAGATACAAAAAAGTTGATCAACCTTAAGCATCTTAATGAGAAACACGCAGATGCTCTGTGGAGGGACAAAGAAAAGGGATTTAATGAAGGATTGTGTAGTTTAATCAAGGCTGTGGAAAAAGACTTCCATTCTGTACACAAGTGTAAGCTCTTCTTTTGTTCAGTCGTTTTCCTTATG

>Cucsa.132370

ATGGGTGACCTACTGACTTTTGGTGTGCAAGAAACTTTGAAGCAGGCTGTAACTCTTGTAGCCAAAAAAATTATTGCGTCAAGTGAATTTAAGGTGGTGCTAGAAGAGCTGAAAGATGATCTACTTCATGCTGAATGGATCCTCCATGCCATAAAAACAAAGCATGATCATTCACTCAATGACAAAATAACTCATTGGGTGAATGATCTTCAACTTATTGTTTATGAAGCTGAGGATATGTTAGACTTGTTTGCTTATGACGATGTTGAACGAAAAATAAGATCAAACAAGGTATTTCCTAATTCCTTATGCACCATAAAACCCATGCTTGATTGTTTTTCTTTGGTCGTCTTTGTACATCTTGACAATACAACCCGAAAAATAGAGAGTGAAGTTGAGCAGGTTGAAGAGACAACTTCACTGCTTGAAAATTATGTGGTGGGAAGGGAGATGGAAGTTGAAAGCATAGTTCAAGATGTGACTGAGGCTAGTCAACAACAACTCAATTCTATTTTACCCGTTTATGGAACGGGTGGATCAGGAAAAACCACTTTGGCCCAGTTGGTGTTTAATGACGAGAGGATTGGAAAACAATTTCATCATACTGTTTGGGTATGTGTGTCTCAACCTTTTGTCATCAACGAGATCTTGCAGTCAATCTTGAAAAAGGTAAGCAAAAGCAATGATAATCGTAGCAAGGATGATAAGGACACCTTAATTCGCAATCTTAAAGAAGTGATGGGTGGAAAAAGATATTTTCTTGTGCTTGACAATGTTTGGAATGAAAACAAAATATTCTGGGAGAAGTTGAAGGAATGCTTAATGAGTATTGTTGAAGAATTAGGAAGCAGTGTCCTTGTCACGACCAGGAGTCGTAAAATTGCAGAAATGATGAAAGAAACACTTGACACCTATCATTTAAACAAATTAACTGATGATCAATGTTGGTCAGTATTTAGCTACTTTGCCAAGGCGAATGCAGTACCAATAACTTCCAATTTGGAGCTTGTGCGAGAAGAGTTAAGCGTGGATCGTCTACCAAAAGCTTCAATAAAGCAATGCTTTGCTTACTGTTCAAATTTTCCTAAAGGTTATTGGTTTGACAAAAAACAAGTGATCAAAATGTGGATGGCACACGGGTTTACTCGACCAGATGAAGGAAATAATGAAACAATGGAGGATACAGGAGAGAGGTACTTCAATATCTTATTGTCTTATTGCTTATTTCAAGATGCTGATGATGACAAATGGCATATTGGTAGGAAGTTTCGTATGCATGATCTTATACACGATATTGCTTGTGATGTTTCAAGCGATAAAAGGTTGCAATTAGATCATAGCAGTTCATCAAAGTGGAAAGGTTTGACAGAAGAGAAAAAAAAAATTGAGAGCAAGTTGCGTACGGTAATAGATTTCGGGAGGAATGGTAAGATTAAAGATTTTGTGTGTTTGCGTGTTTTGACAATTGCAGAAAATGTTCGTGAGTTACCAAACTCAATTTCTAAGTTGAAACATCTTAGATATCTAGACATTTCACGTTGTTATTCAATAAAGAAGCTTCCAGAATCTATTGTTGGGCATTTGGAAATTCTATTAATGGGTATTGATTTACCCCCTAAATTTGAAATGCCTCCATATTTGAGCGAATTGGTTCAACTTCAAACGTTGTTTGCTTTTGCAGTAGGATTTGAGACGGGTCGTAAGATTTCTGAACTTAGGGGTCTTAGAAACTTGAAAGGTTTGTTGAAGCTTCATCGTTTAGAACATGTTGAAAGTAAAGAGGAAGCCAAGGCTGCAAAATTGGTGGAAAAAGAGAAGGTAGAAGGACTAAACTTGTCGTGGCGTGGAAAGTGGAAGAATAGACTTGAACCACACAAAAATCTTAAAGATTTGAAAATCCAATCCTTTTTAGGTGGGTGTTTTCCGAAGGAGACTTTTGTTGAGAATTTAGTAACAATAACTCTACATAAATGTGGAAATTGTGAAAAGCTTCCAATGCTTGGGCAATTAAGCAAGCTAGAGGCACTTATAATTATATCAAACTTTCCAAAAGTAAAGAGTATAGGCAATGAATTCTATGGAAATTATAATGACGGCCAAAGCAAGAGTAGTGTAGTATTCCCCAAGTTGAAGGAATTTTATGTTATTGCGATGTACAGCCTAGTTGAATGGGAAGAAGTGGTAAATAATGTTAAAGCTTTTCCTCGTCTTGAATGTCTGCATATTGTTAAATGTACAAAATTAACATCAGCATTAAAAATTGTCTTTTCTTCTTTAATTAATTTTGATCATTCTATTAGTTACTTAAATTTGCTTCCAAATTCACTCAAAATCTTATTGTCTTGTTGCTTATTTCAAGATGTTGAAGATGAAAGTGAGATAGGTCAGAAGTTTCTTATGCATGATCTTATACATGATATTGCTTGTCATGTTTCAAATGATGAAAAATTGCCATCGGATCATAGCCTTTTATCAATGAGGAAACATTGGACGAACGATGATAAAATAGTTGCGAGCAAGCTACGTACGAATATAGTGAAGAATGAGAATGATTTTGAAGTGTTGGAAGGACTTGAACAACACAATAATCTGAAATATTTGGAAATCGAATCCTTTTCAGGTGGGCAGTTTCCCAACCAGATTTTTGTTGAAAATTTAGTAAAAATAACTCTAATTGAATGTGGAAACTGTGAAAAGCTTCCAATGCTTGGGCAATTAACCAAGTACTTAGAAATACTTGTTATTTTTCGATTACGAAAAGTAGAGAGTATAGGCAATGAGTTCTATGGAAACCAAAGGCGTAGTAGTAGTAGTGTATTCCCCAAGTTGAAGGAATTTTATGTTGATGAGATGGACAGCCTAGTTGAATGGGAAGAAGCTGTGTCAAATTATAATGTTAAAGCTTTTCCACGACTTGAATGTTTGCATATTATTTCATGCAAGAAATTATTGAAAATTCCAGATACAAAGGTTCAAATTTGTGGTCTTTTGTTACTACAACTACAAGAGGAGGCCCCTTGGCTCATTTGCTATTCCAACGTCCGATCATTTTTTTTGAAAGAAGACACGGGTCCTTCATGA

>Cucsa.133510

ATGGCGGGAGCTTTAATTGGTGGCGCGGCATTGGGTGTTCCGTTTAACGAGCTAGCGACCCTCTTGAAGAATTTTGGCGAGAGGGCGTGGAGTTTCAATTCTGTTCTTAACGAGACCGAATCCAAGGTAAATGATATAATTCCTCTGGTTAAAGAAATAGATGGTCTTAATGAATCCCTGGATTATCCAAGAGAAGAAACGGAGAAGTTGAAAAACTTATTAGAATATGCTGGAAAGCTACTTAGACGGTGTTTAAGAGTGGGGAAGGCTGATTTGATAAGGAAATCAAGTCATACAGAGAAGCTTCGTGAACTGAATGCCAGAATCAAAAGTTTCAGTGACGTTGTGTTGTTCCAAACGTCTAGAGACGGGAAGAAGACATTGAGTTTAGTGACTGAGATCAAGGAAGTCGTTCGCAGGCTTGATAGCAAATCTGGATTAAGCAATCCGGTGGATTTAGTTGTGACGGTTCCTGTGATTTCAGAAGAAAGTGTTGGGTTGGAAAAGCCTGTTGAGAAATTGAAGGCCAAACTATTTAGAGATGGGGTTCGATTGTTGGTAGTGACAGCTCCCGGAGGTTGTGGAAAAAGCACTCTGGCCGAAATTTTTTGTCACGACAAGCAAGTTAAAAATAAATTTCAGAGAAACATCTTGTTCCTCGTTGTCTCAAGCAAACCAGAAACGAAACGCATCTTAATATCTATAATTCAAAGACTCGGGGGGCCTATAGAATCTGGTTCTGTAAGTGATGATGAGGCATTCCGGTTGTTAGAAGTTCGGGTGGGGGAATTGAGTCCAAATCCTGTATTGATTGTGTTGGACGATGTCTGGGACGGTTCTGAATCAAACAAGCTTCTTGAAAAGTTCTCCCGATTACCCAACTGCAAAGTTTTGGTCACTTCTAGATTTAAGTTTCCTGCATTTGGTGAGTCGTATGATTTGGAACCTCTGGACCATAAGGATGCAATGGAGTTGTTTCGTCGCTGGGCATCGAGGGGTAACAGAGTGCTACAGTTCCCAGATGAAAGAATTGTAGAAAAGATAGTGAGGGGTTGTAAGAGATTCCCACTTGCTCTGAAAGTGATTGCAGGATCACTTTCGGGTAGAGCCACTTCGGTTTGGGAAGTTACGGGGAGGAAATTATCTAGAGGAGATTCTATTCTGGGTTCTGAGAAAGAGCTTCAGAAGTGCCTCAAAGACACCTTAGATGCAATCCCAGATGACAAGATAGTTCTCAAGGAGTGTTTCATGGACTTAGGTTCATTTCCTGAAGATCAAAGAATTCGTGCGGCTACCTTCATTGACATTTGTGCAGTGTTGTATGAACAAGATGAATGTGAAACAATGTCAAACCTTGATGAGCTCTTCACCCGGACTTTAGTTAACACTGTCTCTTTGAGAAATAAAGCGCATGAAGATGATTACTACAGCGAGTCCTATATTACACAGCATGACGTACTTAGAGAATTGGCTGTCCTTTTGACTAATGAGCAGCCAGTAGACCAAAGAACAAGATTGCTTGTGGATATTAACAAAAATGAATTTCCCAAATGGTGGTCTGTAAGACAGATGCAACCTGTGAAAGCCCGCCTTTTGTCCATAACAACAGATGAGAAGTTCTCATCATGTTGGCCTGATATGGAAGCACCTGAAGTTGAGGTGTTAATTCTAAATCCTGGGTCAGAAACTTACAAGTTACCTGATTTTGCAAAGAAAATGAACAGATTGAAAGCGCTGATAGTCAGGAATTACAGGTCCTTTCCAACTGAATTGACAAGTGATTATCAATTAATCAATTGTTTGTCAAGGCTAGAAAGAATCAGTCTTGAGCGGATTTCAATATCTTCTTTCATTGACCAGAACCTGAAGCCCCTGTGGCATCTTAAGAAGCTATCGTTCTTTATGTGCAAAATTGACAAAGCTTTCACAGACTGCTCAACTCAGATCTCATACATGTTGCCTAACTTACTTGAGATCTCCATAGATTTTTGCAACGATTTGGTGGCTTTCCCTGTCGGACTATGTGAAGTTGTCACATTGGAGAAACTGAGCATTACAAACTGTCATGCATTATCTTCGTTACCCGAGGAAATTGGGCAGTTGATTAATCTAAAAATTCTAAGGCTTAGATCTTGTATTCATTTGGAGAAGTTGCCAGAATCAATCTCAAGGCTCCGGGAATTAGTTTATCTTGACATATCTCATTGTGTTGGCCTTACCAAACTTCCAGATAAGATTGGCAACTTGCAGAAGTTGGAAAAGCTTAATATGTGGAGTTGCCCGAACATGCGCAAGCTTCCAAAATCAGTAGGAAATCTAAAAAATTTGAAGGAAGTAGTTTGTGAAAGCGAGATGAAAATATGGGTGAATTTTGTCGCACCTCGGCTTGGCAATGTGGTAAAAGAACACAAGGAAGAAATCAACTTGGATTTTCTAAATTGA

>Cucsa.155730

ATGGCCATTTCTACAAACCACTCCACTCTCGTCCTCGGCATCTATGGCATGAGCGGCATTGGCAAAACCACTCTCTCTAAAGCACTCTTCAACCACTTCTTCCACTTCTTCAATTCTAGATCTTTTCTCCCCAACATCAACTCCCTCTCTACCTCCTCTCCCGACGGTCTCCTTCGACTCCAACAAACTCTCCTCTCCGATCTCCTCATCGCCACAAACCTCCGCTCTCGTTCCTCAACCACCACCGACTCCACCGTCGTTCGGATGCAGGAAAGACTCCAAAACAAAAAGGTCTTGGTAGTCCTCGACGACCTGGATCGTATCGAACAAGCAAATGCACTAGCAATACGGGACCGAAGATGGTTTGGAGACGGAAGCCGAATCATAATCACAACACGAAACAAACAAATTTTGGACACTCTAAAAGTCGACGAAGTTTACAACATGGAATCCAATCTACTGAACGACGAGGAATCGTTGGAGCTTTTTAGCTACCACGCATTCCGGGAGCAAAATCCACCAGAGGAGCTTTTGGAATGTTCGAAATCCATCGTTTCGTACTGCGGAAGCCTTCCTCTAGCTCTGGAAATCCTGGGTGGGTCATTCTTCGGAGGGAGACCGATGGAGGAATGGAGATCAGCGATGGAGAGATTGAAGAGGATTCCGGCGTGGGATTTGCAAGAGAAGCTTCGAATAGGGTTTGAAGGATTGAGAGATGAGATGGAGAGGGAGATATTTCTTGATGTGTGTTGCTATTTTGTGGGAATGAAAGAGGAATTGGTAGTGAAGATTATGGATGGATGTGGAATGTATGGAGAAAGTGGATTGAGAGGGTTGAAATGGAGGTGTTTGGTTGGTGTTGAGTTTTGGAGTGGGAGGTTGAAGATGCATGATTTGGTTAGGGACATGGGGAGGGAGATTGTGAGGCAAACATGTGTGAAGGAACCTGCTAGACGGTCCAGGGTTTGGCTTTATCATGAGGCTCTCAAAATCTTACTCCATCAGAACGGAAGTGAAAACATTGAAGGACTTGCAATAGACATGGGTAAAGGAAATAACAAGGAGAAATTCAGATTGGAAGCATTTGGGAAAATGAGAAATCTAAGGTTACTCAAACTCAACTATGTGCATCTCATTGGAAGTAATTTTGAGCATATAATAAGCAAAGAATTAAGGTGGATTTGTTGGCATGGATTCCCTTTGAAGTCTATTCCAAGCTCATTTTATCAAGGAAACCTTGTTGCCATTGACATGAGATATAGCAGCTTGATACACCCTTGGACTTGGAGGGATTCACAGATTCTTGAGAATCTAAAAGTTCTAAACCTAAGCCACTCTGAAAAGCTAAAGAAGTCCCCAAACTTCACAAAGCTCCCAAACCTAGAGCAGCTAAAACTCAAGAATTGCACAGCCTTATCAAGCCTCCACCCCTCCATTGGTCAACTTTGTAAGCTTCATCTCATCAACCTCCAAAACTGTACAAATCTCTCGTCTTTACCAACCTCCATCTACAACCTCCACTCCCTCCAAACTTTCATCATCTCTGGCTGCTCCAAGATCCACTGCCTCCACGACGACCTCGGTCACCTTGAGTCCCTCACCACCCTTCTCGCTGACCGAACCGCCATATCCCACATTCCTTTCTCCATTGTCAAGTTGAAGAAACTCACTGACTTGTCTCTATGTGGTTGTAACTGCAGATCAGGATCGGGAAGCTCGGCATCGCTGCCATGGAGGCTGGTTTCATGGGCATTGCCAAGACCAAACCAAACATGCACAGCCCTAACTCTTCCATCTTCATTACAAGGTTTGAGCTCTCTAACAGAGTTGAGTCTACAAAATTGCAATCTCGAGTCACTTCCAATTGACATTGGGAGCTTGAGTGAACTAAAGAAGTTGAATCTTGGAGGCAACAAAAATTTGAGGGTTTTGGGGACTGAACTTTGTGGACTTTTGAAACTGAATGAGCTGAATGTGGAGAATTGTGGGAGGCTTGAGTTCATCCAAGAATTTCCAAAGAATATGAGAAGTTTTTGTGCTACCAATTGTAAGTCATTGGTGAGAACTCCTGATGTTTCCATGTTTGAAAGAGCACCTAATATGATTCTCACCAATTGTTGTGCATTGCTTGAGGTTTGTGGATTGGACAAATTGGAGTGCTCTACTAATATTCGTATGGCCGGTTGTTCGAATCTCTCTACTGACTTTAGGATGAGCCTTCTTGAGGTTTTTTCTTCCTCTTATCTGTTGTTAAATTACAAATTTGGTTTTGCAGTTTTTCACTTATATATAGTATCAATTTAG

>Cucsa.163670

ATGATACCACACGAAATCTTATCACTTTTCATAACCTCTGTCTATGAGTATTTGACAAATATTGCAACCAAATTGGGTTCTTTAGCACTCCAAGACCTTGGATTGCTGTGGACCGGTATCCATGAGGAGATTGACAAACTCAGAGACACTCTTTCCGCCATCCAAGCAGTACTTCACGACGCAGAACAGAAGCAGTACAAGAGTTCTGCTGTGAAGGAATGGGTTTCAAGGCTAAAAGATGCTTTCTATGATATGGATGATTTGATGGATGAGTTCTCCTATGAATCCTTTCAAAGACAGGTTATGACCAAACATAGAACCAACAACTGTACCAAACAAGTATGTATTTTCTTCTCAAAATCTAATCAAATTAGATTTCGTTTGAAAATGGTTCATAAAATAAAAAAGATCAGGGAGAAACTCGATACTATTGATAAGGATAAAACTCAATTCAATCTTTTTGATAATACAAGGGAGATACGAAATGATGAAATGACAAAACGATCAGAGACTTGCTCTTTTATACTTGAAGGAGAAGTAATTGGTCGAGATGATGACAAGAAATGTATTGTACATTTTCTATTGGATACCAACATTATTGCAAAGGAAAATATTGTTGTGGTTGCCATTATTGGAATGGGAGGATTAGGAAAGACTGCCCTTGCTCAATCTATCTACGGCGATATGAAGGAAAATAAACATTTTGAATTGACAATGTGGGTGTGTATTTCTGAAGAATTTGATGTCAAAGTAATTGTTGAAAAGATCATAGAATCTCTCACAAAAAAGAGACCTAAGCCCAACCTTACACTCGATACCTTACAAAGTATGCTACGAGAGAAAATTGATGGAAAAAAATACTTGCTTGTCATGGATGATGTGTGGAACGATGAACGGACGAAATGGATTAATCTAAAAAAATTTCTTATGGGTGGAGCTAAGGGAAGTAGGATTTTGATCACAACTCGTACCCATCAAGTTGCACATATTTTTGACACAGATTTGTTCCATGATTTAAGTGAACTAGACAAGGACAACTCTTGGGAGTTGTTTAGAAAAATGGCATTTTCCAACGAATCAGAGATGCTTGAGAATTCAAAGTTGGTCGGGATCGGTAAGGAGATTGTGACAAAGTTGAAAGGTTCTCCTCTTGCAATAAGAGTAATTGGAAGCTATCTGTATTCTAAAAAGTCAGAAAAGGATTGGTTGTCATTCAAGGAGAACGAACTTGACACAATCATGCAACAGGAAAATGAGATTCAATCCATACTAAAGATTAGTTTTAACCACCTCTCATCCAGTTTGAAGCAATGTATCACATATTGTGCTTTGTTCCCTAAAGATTTTGAGATTGATAAAGATGATTTGATAAAACAATGGATGGGAGAAGGCTTCATTCAACCACATAATAAGAAGGCAATGGAAGATGTTGGTGATGAATATTTCAAAGAACTCTTGGGAAGATCATTTTTTCAAGACATAAGTAAAAACCAACTGGGAGAGATCATGAAGTTCAAGATGCACGACTTCATGCATGATCTTGCATGTTTTGTTGGAGAAAATGATTATGTGTTTGCTACTGATGACACTAAGTTCATTGACAAAAGGACTCGACATTTGTCAATTTCGCCCTTCATCTCAAAGACAAGATGGGAAGTCATTAAAGAATCATTAATAGCGGCAAAGAATTTGAGAACATTGAACTATGCTTGTCACAATTATGATGGTGATGAAATCGAAATCGACTTCTCTAATCATTTGCGGTTACGAACATTGAATTTAATATTTTCTACTCATGTTCCCAAGTGTATTGGTAAGATGAAACATTTGAGATATATTAATTTTACTCGGTGTTATTTTGATTTCCTTCCCAAGGTAGTTACAAAATTGTACCATTTGGAAACACTTATCTTTCGTGAATGTTTCAAGCTAAGAGAACTGCCAAGTGATATTACGAATTTGATCAATCTCAGGCATCTTGGTATTAACTCTTTAATTGAAGGTTTAAGTTATATGCCAAAAGGAATGGGTTCAATGACTACCCTTCAAACAATGAATTTGTTTATATTGGGAGAGAATGAAGGTGGTGAGTTAAGTGAACTTAATGGATTGATTAACTTGAGAGGATCATTAAGTATTCAACAATTGCAGTTCTGCAAACCCATTGGTATAGAAAATGCTAAACACCTTGAAGAAAAGTCTGGAATTCAAAAGTTAAAATTATATTGGTATCTCTTGGAAAGGAAATATGAAATTGATGATGAAGATGAGAAAGTTTTAGAGTGCTTGAAACCACATCCAAATCTTCAGAAAATAGTCATAAATGGATACGGTGGAGTGAAGCTATGTAATTGGTTCTCATTTGATTATATTGTCAATTTGGTCATTATAGACCTTTTCAACTGTAATAAATTGCAACAGCTCCCTCGATTTGATCAATTTCCTTTTCTCAAACATCTTAAGCTCCAATATTTACCAAATGTTGAGTTTATTGATAATAACGATTCTGTTTCTTCTTCGTTAACAACTTTCTTTCCCTCCCTTGAGAAACTGAGAATCTTTAGGTTACCTAAGTTGAAAGAATGGTGGAAGAGGAAACTCATCGATCAAACTATTCCACAACATAGACGTTTGGAATCATTGAACATAAGTGGTGTTAGTTTGCAAGTTTTTGAGTTGGTAATGGAAATGGCTACTACAAACATTATTGTTGGATCACAGGATTCTTCTTCTTCAACTACATCTATATCATTATCTTTTCTAAGTATTGAAGACATTGATTTTGAGTTTTTACAATTCCATGACTTATTCTCCAATATGACACATCTTAAGTCTCTTTGGATAATAAATTGCAAGAATATAAAAATGTCTTCTTCTCTTGATGCTGTGACATGGAAAGGACTTGGAAGTCTTCGTGAACTTATGTTGTCCAGCATCCCTGATTTGGAATATTTGCCGAAGAGTTTGCAATGTGTGACAACTCTTCAAAGTTTGCAAATATATAATTGTCCAAATTTGGTATCTATTGAAAGTATTAGGCATCTCACCACTTCACTATCAGTATTGGAAATTCATGGTTGTCCTAATATAACTTTCTACCCTCACGAAATGAGTCAACTCGCTTCACTAGCTATCACATTTCAGAATCGTGGTTGGTCGGATAATTATGATCCAGGTGAAGGAAGGAAAGAAGATGACGATCAGAAACAATTTGGAAGAGATGAACAACATGAAGGAACGACTCATTGA

>Cucsa.178360

ATGGAGTTGTGTGCCGGTGCCATTGTTAATCCAATCGCAGAAAAAATCGCCAACTGCACGGTGGATCCGGTTTTCCGGCAACTAGATTATTTGCTCCACTTTAAAACCAATGTGAATGATCTCAAAGATCAAGGCAAGAAGCTGGTGGAAACCAGAGATTTTGTTCAACATTCTGTCGACTCCGCCAAAACCAATGGGTACGAGATCGAAGTTATGGTCACTGAATGGTTGGGGATAGCTGATCAATTTAGTGAAGATGTCGATAGGTTTTTCAACGAAGCCGACGGCCGAAGTCTTCGATGGTGGAATATGCTATCACGCCATCGATTTAGTAGAAGAGCTACCAAATTGGCTGTGGCAGTTGATAAAGCCATTCAAGGTGGGAGTTTCGAGAGAGTTGGGTTCCGTGTAACTCCACAAGAAATTATGACGCTAAGGAACAATAAGAAGTTCGAAGCCTTTGAATCTAGGGTTTTGATTCTGAAGGAGATAATTGAAGCGGTTGGCGATGCTAATGCGAGGGTGATTGTGGTACATGGGATGGCGGGAGTTGGGAAAACCACCCTAGTTGAAGAAATTGCAAGATTGGCCAAGGAGGGGAAGCTTTTTGATGCTATAGCAATGGTGACTGTAAAGCACATTCCAAACATTAAGAAAATACAGGGGGAGATTGCTGATCAATTGGGGTTGAAATTTGAAGAGGAAAAGGAACGAATTAGGGCCGATCGACTACGTCGAAGGTTAGAGATGGAGAAGAAGGTGTTAGTGGTTTTGGATGATGTTTGGAGTAGGCTTGATTTGGAAGCTGTTGGAATTTCTAGCCATCACAAGGGATGTAAGATACTTGTAACTTCTAGAAAGGATGATTTGTTTTTCAATGATTTTGGTACTCAGAAAAATATATATATCAATATTCTGTCAAAAAAAGAAGCTAGGGATTTTTTCAACAAGGTGGCATGTGATTCTGTTGAATCTTCTGATGATACTGATCCTGAAATGGAAGCTGTTGCTACTGAATTGGCAGATGAATGTGGAGGATTGCCACTTTCTCTTGCAACTGTTGGACAAGCCTTGAAAGGTAAAGGGCTTCCAAGTTGGAATGATGCCTTGCAAGGAATGAAGTTTCCTGGCGAACCCAGTAACTATGGGGTGAATAAAGTGGCATATTTGTCTCTGAAAGTGAGTTATAGATCTCTAAACAGAGAAGAAGCCAGATCACTATTCTTACTATGTAGCTTGTTTCCAGAAGATTATCAAATTAACATCAAATACTTGTTGATGTATGCCATGGGTTTGGGGTTATTAAACGCCATGAGTTCTCTAGCAATGGCAAAATGGAGAATACTTTCTTTGGTTGATGAGCTCAAAACTTCTCACTTGTTGCTTGATGGGGTTGATAACGATTTTGTGAAAATGCACGATATAGTTCGAGATACAGCAATTTTGATTGCGTCGAAAATGAAGTCCAAGTATTTGGTTAGACATGGTGCTGGAGAGAGTTTGTGGCCCCCAATGGATGAGTTCAAAGATTACACTGCAATCTCATTAGGTTGCAGTGATCACTCGGAACTCCCAGAATTTATATGTCCACAGCTTAGATTCTTATTACTGGTAGGAAAAAGAACATCTTTGCGATTACCTGAAAAGTTCTTTGCAGGTATGCAGGAACTACGAGTTTTAGATCTCACTGGCTTATGTATTCAGCGGCTTCCACCATCAATCGACCAACTGGTAAATCTTCAAACATTGTGTTTAGATGACTGTGTTTTGCCAGACATGTCTGTAGTTGGTGAACTGAAAAAGCTTGAAATTCTTAGCTTGAGAGCATCTGATATTATTGCACTTCCTAGAGTAATTGGGGAACTTACCAATTTGAAAATGTTGAATTTGTCTGATTGTTCTAAACTCAAGGTGATCCCTGCTAACCTTTTATCTAGGTTGATAGGGTTGTCTGAGCTATACATGGACAATAGTTTTAAACATTGGAATGTAGGACAGATGGAAGGTTATGTTAATGCAAGGATTTCTGAACTAGACAACCTGCCACGGTTGACCACTCTACATGTCCATATTCCAAATCCCACCATTCTACCACATGCCTTTGTCTTTAGAAAATTGAGTGGTTACAGAATACTAATTGGAGATAGATGGGATTGGTCTGGCAATTATGAAACTTCAAGGACCTTGAAACTCAAGCTTGATAGTAGCATTCAGAGAGAGGATGCAATTCAAGCACTTCTAGAGAATATTGAAGATCTGTATTTAGATGAATTAGAAAGTGTCAAGAATATTCTATTCAGTCTAGACTATAAAGGCTTTCCGAAATTGAAAGGTTTGCGTGTCAAAAACAATGGTGAAATTGTGACTGTTGTCAACTCGGATAACATGCATCATCCACACAGTGCCTTTCCATTGTTGGAGTCCTTATTTCTGAAAAATCTAGCTGAACTTGGAAGCATTTGTCGTGGAAAGCTTCCACAAATGTCCTTCCGTAACTTGAAAAGAGTAAAAGTTGAAAGTTGTGACAGATTAAAATTTGTTTTCCCATCTTCTATGGTCAGAGGCCTTATACATCTTCAAAGCCTGGAGATTAGTGAATGTGGCATCATAGAAACTATAGTTTCGAAAAACAAAGAAACAGAAATGCAAATCAATGGTGATAAGTGGGATGAGAACATGATTGAGTTTCCTGAATTGCGTTCTCTGATACTTCAACATCTACCAGCCCTTATGGGTTTCTATTGTCATGATTGCATAACTGTGCCTTCAACCAAAGTGGATTCACGTCAAACAGTTTTTACTATTGAACCTAGTTTTCATCCACTTCTCAGTCAACAGGTTTCCTTCCCCAAATTGGAGACATTAAAATTACACGCTTTGAACTCAGGAAAGATATGGCAGGATCAACTTCCTTCTAGCTTTTATGGCTTTAAAAATCTAACTTCTTTGAGTGTGGAGGGTTGTGCTTCAATAAAATATTTAATGACAATCACTGTGGCTAGAAGCCTTGTGAATCTTGAACGCCTTGAACTAAACGACTGTAAGTTGATGAAAGCTATAATCATTTCAGAAGATCAAGATCTGGACAACAATTACCCTTCCAAATCTATCTTGCAGAACAAGGATGTTTTTGCGAACCTGGAGTCCCTCTTAATCTCTCGCATGGATGCTTTGGAGACATTATGGGTCAATGAAGCTGCTTCAGGATCCTTTACAAAGCTGAAAAAAGTGGACATCAGAAACTGCAAAAAACTTGAGACAATCTTTCCAAATTACATGCTTAACAGAGTGACAAATCTCGAGAGATTAAACGTTACAGATTGCAGTTCCCTAGTGGAGATCTTTCAAGTGAAAGTCCCAGTTAACAATGGCAACCAAGTAAGAGACATTGGAGCTAACCATTTGAAAGAGTTGAAGCTGCTTCGTCTACCTAAACTAAAGCACATATGGAGCTCAGATCCACACAATTTTTTACGCTATCCATCTCTCCAACTTGTTCATACAATTCATTGTCAAAGCCTTTTGAATCTCTTCCCTGTATCCATAGCTAAGGATCTCATACAACTTGAAGTGCTTAAAATACAGTTCTGTGGAGTTGAGGAAATTGTTGCGAAACGAGGAGACGATGGAGATGGAGATGATGCTGCGTCGTTTTTGTTGAGTGGTTTGACATCATTGACTCTTTGGAATTTGTTCGAGTTCAAGAGGTTTTATCCTGGGAAATATACTTTGGATTGTCCATCATTGACAGCGCTAGATGTACGCCATTGCAAATCATTTAAGTTGATGGAAGGAACTTTGGAAAATTCGTCATCAATCTCATCCGCTGTTGAAAAGGTATTACATTCTCTCTAA

>Cucsa.178450

ATGAATCAAGCAAGTGGGTCATCTTCTTCCTCACGTTTTAGATGGCATTTCGATGTATTTTTAAGCTTTCGAGGGGAAGACACTCGATCCAACTTCACTAGTCATCTTAATATGGCTTTGCGTCAAAGAGGAATCAACGTTTTTATAGATAACAAACTTTCAAGGGGTGAAGAAATTTCTGCATCTCTTTTGGAAGCTATTGAAGGATCCAAGATCTCCATTGTCATAATCTCTGAAAATTATGCTTCTTCCAGGTGGTGTTTGAATGAGCTGGTGAAAATCATTATGTGTAACAAATTGAGAGGACAAGTGGTTTTACCAATTTTCTACAAAGTGGATCCATCTGAAGTAAGAAAACAAAGTGGAAAATTTGGAGAAGAATTTGCCAAACTTGAAGTTAGATTCTCGTCGGAGAAGATGCAAGCATGGAGGGAGGCCATGATTTCTGTTTCTCATATGTCTGGATGGCCGGTTCCTAAGAAAGATGACGAGGCCAATTTGATTCAAAGAATTGTTCAAGAAGTCTGGAAGAAATTAAATCGTGGAACAAGAGAGATGCGTGTACCTAAATATCCAGTTGGAATAGATAGACAAGTTAATAATATACTCTCCCAAGTTATGTCTGATGAAATAATTACTATGGTTGGATTATATGGAATTGGAGGTATTGGCAAGACAACTTTGGCCAAAGCTTTATACAATAAAATTGCTGATGACTTTGAAGGTTGTTGCTTTTTGATAAATGTTAGAGAAGCTTCAAATCAATATCGGGGTCTTGTTGAACTCCAAAAGGAGCTACTTCGTGAGATTCTAATGGATGATTCAATCAAAGTTAGCAATCTCGATATAGGAATTAGCATCATAAGGGATCGACTATGCTCAAGAAAGATTCTTTTGATTCTTGATGATGTTGATACGAGTGAACAACTAGAAGCATTAGCAGGAGGACATGATTGGTTTGGACCGGGAAGTGTGGTCATTGCGACAACAAGAAACAAACACTTACTTGCTATTAATGAATTTGATATATTGCAAAGTGTTCAGGGATTGAATGATGATGAAGCCTTCGAGCTTTTTAGCTGGCATGCTTTTAAGATGAGTTGTCCATCAAGTCATTATTTATACCTAATTTCAAAACGTGCCGTAAGTTATTGTAAAGGTCTTCCCTTGGCTTTGGAAGTTGTAGGTTCATTCCTTTATTCTATTGAGCCATCCAAGCTTAAACTTATATTGGATGAATATGAAAACCAATACCTTGACAAGGGCATCCAAGATCCTCTTCGAATAAGTTATGATGGACTTGAAGATGAAGTAAAAGAAATTTTTCTTTATATTTCTTGTTGCTTTGTAGGAGAAGACATCAACAAAGTTAAAATGAAGTTAGAAGCATGTGGTTGTTTATGTTTGGAAAAAGGAACAACAAAACTCATGAATCTATCACTTCTGACCATTGATAAATCCAATCGGGTTGAAATGCATAATTTAATACAACATATGGGTCGCACAATTCATCTTTCGAAGACTTCTACATCTCATAAAAGAAAAAGATTGTTGATTAAAGATGATGCTATGGATGTCTTAAATGGGAATAAGGAAGCAAAAGGAGTTAAAGCCATAAAATTAAGTTTTCCTAAAGCTACGGAGTTGGACATTGATTCAAGAGCTTTTGAAAAAGTGAAAAATGTGGTAGTACTCGAAGTTGGCAATGTCACATCTTCAAAAGGTACTGATCTTGAGTATCTACCTAGTAGCTTAAGGTGGATGAATTGGCCTCATTTTCCTTTTCCATCTTTGCCTACAACCTACACAATGGAGAACCTTATGGAATTGAAATTGCCATATAGCTCCATCAAACATTTTGGAAGAGGATTCATGAGTGGTGAACGGTTGAAGGAAATTGATCTTAGTGGCTCTGAGTTTTTAGTGGAAATTGCTGATTTATCTACTGCAACAAACCTTGAAAAGTTGAATCTTTTAGGGTGTGTAAATTTAGTAAAAGTTCATGATTCTGTGGGATCTCTCACTAAGCTTGTTACATTTTCTCTTTCTAGCAATGTTAAGGGCTTTGAGCAGTTTCCACCCCACCTCAAGTTGAAATCCCTCAAACTTTTGTCAATGGAAAATTGTAGAATAGATGAATGGTGTCCTCAATTTAGCGAAGAAATGAAGTCTAGCCTAGAAGAATTGTTGATTCAATATAGTACTGTAATTAATCAGCTATCTCCAACAATTGGATATCTTACTAGCCTAAAACGTTTGTTTATCATAGAGTGCATGAAGCTCAAAACTCTTCCAAGTACAATTTATCGTTTAAGGAATCTTACTTTTTTAAGTATAACAAATTTGGATTTCTTAGAAACAATGGTTCATGTTGCCCCTGCATTGAAACTGTTGGACTTATCTGGAAACAATTTTTGTAGACTACCCTCATGTATTATTAATTTTAAATCCTTGAAATCTCTTGTTACAATGGAATGCAAGTTGCTTGAAGAAATTCCAAAGGTTCCAAAAGGAGTAGTTCGTATGAATGCTACAGGGTGTATATCATTAACCAGATTTCCTGACAACATACCTGACTTCATATGCTGCGATGATAATGTGGTGCGTATCATTGTTCTTTCTCATGACCTCATGATCTCTCGTGTATTTCGTTCATATAAGAATTAA

>Cucsa.178620

ATGGCTGAAGCTATTCTCTTCAACCTTACTGCAGACATCATATTCAAACTGGGTTCTTCAGCACTCCGACAGTTTGGATCTCTACGGGGCGGTGTCAAGGATGATTTTGACAAACTCTGGCACTCTCTTTCTGCCATCCAAGCTGTTCTTCACGACGCGGAGGAGAAGCAGTTCAAGGACCATGCGGTCGAAGTTTGGGTTTCAAGGCTTAAGGATGTTTTGTACGAGATTGATGACTTGATCGACGAGTTCTCTTACCAAATCTTGAGAAGGCAAGTTCTGCGAAGTAACAGAAAACAAGTACGTACCCTCTTCTCCAAATTTATAACTAATTGGAAAATAGGCCACAAAATCAAGGAAATCAGTCAGAGGCTACAAAATATTAATGAAGATAAAATTCAATTTAGCTTTTGTAAGCATGTGATAGAGAGAAGAGATGATGATGATGAAGGGTTGAGAAAGAGACGGGAGACTCACTCTTTTATACTTGAAGATGAAGTGATTGGTAGGAATGATGACAAGGAAGCAGTCATAGATCTTCTGCTAAATTCCAACACCAAAGAGGATATTGCAATTGTTTCCATTGTTGGAATGCCAGGATTTGGAAAAACTGCCCTTGCCCAATCTATTTATAACCATAAGAGGATAATGACTCAATTTCAGTTGAAAATATGGGTGTGTGTTTCTGACGAATTTGATCTGAAAATTACTATCCAAAAGATAATAGAGTCTGCAACCGGGAAGAAGCCTAAATCATTCCTTCAAATGGATCCATTACAATGTGAGCTTAGAAAGCAAATTGATGGAAAGAAATATTTGATCGTCATGGATGATGTGTGGAATGAGAAAAAAGAGAAATGGTTACATCTGAAAAGATTGTTGATGGGCGGTGCAAAGGGTAGTAGGATTTTGATCACAACACGCAGTGAACAAGTTGCAAAAACTTTTGACTCTACTTTCGTTCATCTATTACAAATTTTGGATGCATCCAATTCTTGGTTATTGTTTCAAAAGATGATTGGTTTAGAAGAACATTCAGATAATCAAGAGGTCGAGCTTGATCAAAAGAATTCAAATTTGATCCAAATCGGCATGGAGATTGTGTCAACGTTAAGAGGTGTTCCGCTTTTAATAAGAACCATTGGAGGACTTTTAAAAGATAATAAATCAGAAAGATTTTGGTTGTCTTTTAAGGATAAGGAACTTTATCAAGTCTTGGGACGAGGACAAGATGCTCTGAAAGAAATACAATTGTTTCTTGAGCTTAGTTATAAATATCTCCCATCGTCTAACTTGAAACAATGTTTCCTATATTGTGCTTTGTTCCCCAAAGATTATCGAATTAAAAAGGATGAACTTATATTACTATGGAGAGCACAAGGTTTCATTCAACAAAATGGCAACAACGACGACAATAGTTCCCTCGTTGATATTGGTGAAGATTATTTCATGGAGTTATTATCAAGGTCGTTTTTTCAAGAGGTTGAAAAAAATGATTTTGGAGATATAATAACATGTAAGATGCATGATTTGATGCACGATCTTGCTTGTTCGATAACAAATAATGAATGTGTGCGTGGACTGAAGGGAAATGTCATCGACAAAAGAACTCATCACCTTTCTTTTGAAAAAGTTAGTCATGAAGATCAACTTATGGGATCATTATCTAAGGCAACTCATTTGAGGACACTTTTTAGTCAAGATGTTCATTCACGATGTAACTTGGAAGAAACCTTCCACAATATTTTCCAATTGCGAACATTGCACTTAAACTCGTATGGTCCACCCAAATGTGCAAAGACTTTGGAGTTTATTAAATTCCTTCCCGATTCTATTACAAAATTGTATAAGTTGGAAGCACTTATACTTGACGGTTGTTCCAATTTGAAAGAATTGCCAAAATATACTAAAAGGTTGATCAACCTTAAGCGTCTTGTTTTGTACGGATGTTCGGCTCTCACTCATATGCCAAAAGGATTAAGTGAGATGACTAATCTTCAAACATTGACTACATTTGTATTGGGAAAGAATATTGGTGGTGAGTTAAAGGAGTTGGAAGGACTTACTAAATTAAGGGGAGGATTAAGCATTAAACATTTGGAATCTTGTACCAGCATTGTTGATCAACAAATGAAGAGTAAGAACAGTAAGTTCTTGCAACTAAAGTCTGGTCTTCAAAACTTGGAGTTACAATGGAAGAAACTGAAAATTGGTGATGATCAGTTGGAGGATGTGATGTACGAAAGTGTTTTAGATTGCTTACAACCACATTCAAATCTTAAAGAGATACGTATTGATGGATATGGTGGAGTAAATTTATGTAATTGGGTATCCTCTAATAAGTCCCTTGGTTGTCTTGTCACTACATATCTTTATCGTTGTAAAAGATTACGACATCTCTTCAGATTAGATCAATTTCCTAATCTCAAGTATCTTACGCTTCAAAACTTACCCAACATCGAGTACATGATTGTAGACAATGATGATTCAGTTTCTTCATCAACAATTTTTCCATACCTAAAGAAATTTACTATTTCAAAAATGCCTAAGTTGGTGAGCTGGTGCAAGGATTCAACCTCAACCAAATCTCCTACATATTGGCATGCACCTAAGTTGAAACTCTTGCAAATTAGTGATTCAGAGGATGAGTTGAATGTTGTACCGTTGAAAATTTATGAAAACCTCACCTTTCTATTTCTTCACAATTTGAGTAGAGTGGAGTACTTGCCCGAGTGTTGGCAACATTATATGACATCTCTACAACTTCTTTGTTTAAGCAAATGCAACAATTTAAAGAGCTTGCCGGGTTGGATTCGCAACCTTACCTCTCTTACAAATTTGAATATTTCTTATTGCGAAAAGCTAGCTTTTCTACCAGAAGGGATTCAACACGTCCATAATTTACAATCGATAGCAGTTGTTGATTGTCCTATATTGAAAGAATGGTGCAAGAAAAACAGACGAGAAGATTGGCCTAAGATCAAGTACTATATTTCCGAACATATTTGGGAGAATATTTGTAGCCTCACAGGTTCTTGGTCATCTAGAAACAAGATTTTTTCTGATCATTTCCGCTCTGTAATTGCTCTAAACTGTAGTGCATGCTTTTACTTGATTTATATTTGTCGCTGCTACTATGAGCTTATCTCTAGTCCTTTTTTTATCATCTTGTATCATGTGCTTTTCAATGAATGA

>Cucsa.189390

ATGGATATAATTTCTCCTGTCGTTGGACCAATTGTGGAGTACACTTTAAAGCCTATTGGTCGTCAATTGAGTTATCTATTCTTCATTCGCCAACATATTCAAAACCTTGAGAGTCAAGTTGAATTGTTGAAGAACACTAAAGAATCGGTGGTTAACAAGGTTAATGAAGCGATAAGAAATGCTGAAAAGATAGAATCCGGTGTTCAAAGTTGGTTGACTAAGGTGGATTCCATCATTGAAAGATCTGAAACGTTACTAAAGAATCTTTCTGAGCAAGGTGGATTGTGCTTGAATTTGGTCCAGAGACACCAATTAAGTAGGAAAGCTGTAAAGTTGGCTGAGGAGGTTGTTGTGATAAAAATTGAGGGGAATTTCGATAAAGTCTCCTCTCCTGTAGCTCTTTCAGAGGTTGAGAGTTCAAAGGCAAAGAATTCTGATTTTGTCGACTTTGAATCAAGAAAGCCAACTATTGACAAAATCATTGCTGCACTTATGGATGATAATGTCCACACAATTGGAGTGTACGGGATGGGAGGTGTTGGCAAAACAATGCTAGTCCAAGAGATTTCAAAATTAGCTATGGAGCAAAAGCTATTTGATGAAGTAATCACATCAACTGTTAGTCAAACGCCAGACTTAAGAAGAATTCAAGGACAACTTGGTGATAAGCTTGGACTCCGATTTGAACAAGAAACAGAAGAAGGAAGGGCTCTTAAGTTACTAAATAGGTTGAAGATGGAACGTCAAAAGATCCTCATTGTACTTGATGATGTTTGGAAGCAAATTGACTTGGAAAAAATAGGAATTCCAAGCATTGAAGATCACAGTGGATGCAAGATCCTATTTACCTCTAGAGATAATGATGTTCTCTTTAATGATTGGCGCACATATAAAAATTTTGAGATAAAATTTTTACAAGAGGACGAGACGTGGAATTTATTCAGGAAAATGGCTGGTGAGATTGTTGAAACATCTGATTTTAAGAGTATAGCTGTTGAAATAGTAAGGGAATGCGCACATTTGCCCATTGCTATTACTACAATCGCTAGGGCATTGAGAAATAAACCTGCATCCATTTGGAAAGATGCCTTAATCCAACTAAGAAATCCTGTCTTTGTGAATATTAGAGAAATAAATAAGAAAGTGTATTCTTCCCTAAAGTTAAGTTACGATTACTTAGATTCTGAAGAGGCCAAATCACTATTTTTGCTCTGTAGTATGTTCCCAGAAGATTATATCATTGATTGTCAGGTCTTGCATGTATACGCTATGGGCATGGGTTTATTGCATGGTGTTGAGAGTGTAGCACAAGCACGAAATAGGATAACGAAATTAGTTGATGATCTCATATCTTCTTCTTTGCTTTTAAAAGAATCAAATGTCGATTTGGTTATGTATGTTAAAATGCATGATATAGTTCGTGATGTGGCTATAATAATTGCATCTAAAGATGATCGTATTTTTACACTAAGCTATTCCAAAGGATTATTGGATGAATCATGGGATGAAAAGAAACTAGTAGGTAAGCATACTGCAGTGTGCTTAAATGTTAAAGGTTTGCATAACCTTCCCCAAAAGTTAATGCTACCCAAAGTTCAGTTATTGGTGTTTTGTGGAACTTTATTAGGTGAACATGAGTTGCCAGGAACATTTTTTGAAGAAATGAAAGGGATGCGAGTTTTGGAAATAAGAAGCATGAAAATGCCCTTATTGTCACCATCACTTTACTCTTTGACAAACCTTCAATCGTTGCATTTGTTTGATTGTGAATTGGAAAACATAGATGTGATTTGTGAGTTGAACAAACTTGAAAATCTCAGCCTAAAAGGATCACATATCATCCAGATCCCTGCAACTATAAGTCAATTGACACAACTAAAAGTATTAGACTTATCAGAATGTTATGCACTAAAGGTAATTCCGCCTAATATTCTTGTAAATTTGACAAAGTTGGAAGAATTATATTTGCTAAATTTTGATGGTTGGGAAAGCGAAGAATTGAACCAAGGAAGAAGAAATGCTAGTATATCTGAGCTTAGTTACCTTTCTCAGCTTTGTGCTTTAGCATTACATATTCCAAGTGAAAAAGTTATGCCAAAAGAGTTGTTTTCAAGGGTTTTCCCGTTTGAGTTGAATGAAAACGAATCTTCATATTTGAAGTATCTCTACATCAACTATAATTCAAATTTTCAACATTTTATCCATGGACAGAACAAGACTAATTTGCAAAAAGTCTTGTCCAATATGGAGCGTCTGGAATTGAGCTATTTGGAGAATTTGGAGAGTTTTTTTCATGGTGATATTAAAGATATTTCTTTCAACAACTTGAAGGTCATAAAGTTGTTAAGTTGTAATAAATTAGGAAGTCTTTTTTTGGATTCCAACATGAATGGCATGTTATTGCATCTTGAGAGGATTAACATTACTGATTGTGAGAAGGTGAAAACAGTTATTTTAATGGAAAGTGGAAACCCATCTGACCCTGTTGAATTTACAAATTTGAAGCGTTTAAGGCTAAATGGGTTACCACAACTTCAAAGTTTTTACTCCAAAATTGAACAATTGAGTCCTGATCAAGAAGCAGAAAAAGATGAGAGAAGCAGAAATTTCAATGATGGTTTACTTTTTAATGAACAG

>Cucsa.237070

ATGGAAACTGTAATCGCAATTTTAGGGACAGTTTGTGAGTACGCAGTTGCACCCATTGGACGTCAAGTAGGATATGTTTCTTCCTACAAAAAGAACATCAATGATCTTAAAGACCAACTTCAAAATCTTGTGGATACTAAAACAAGGCTACAACACATGGTCAATGAGGCAAGAAGTAGTGCGTACAATATCCAAAGTGATGTTTCATCATGGTTGAACCAAGTAGATAAAATCATTGAACAATCCAACGACATATTGTACAAGAATGAAAATGAATCAAATAGCAAGTATTGTTCCAATAAGCTTAACTTCATTCATCAATATCAAATGAGTAAGAAAGCTAAGAAGATGGTGAAAGTAATTTCACAAATTATAGAGAAAAGAAAGTTGATGTTTCACCAAGTTGGTTATCCTACACCTCTTTCAAGGATTCATGGAAGTTCTACTAGTAGTTCTCATGGCTATGATCAGATTTTGGAATCAAGAACATCAATCGCCAAGCAAATTAGAGATGCACTCGTTGATTGTAACGTGAATAAGGTTGGAGTATATGGTATGGGAGGTGTTGAAAAAACTACGCTGCTGAAACAAGTCACACCATTAGTGATGGAAGAAAAATTGTTTGATCACGTGATTATAGTGAATGTAGGTCAAACATTGGGGGTAGAAGGCATACAAGCTCAAATTGGAGATAAGTTAAGGTTGGAATTAAATAAGAAGGTAGAGAGTAAGGAGGGAAGAGCATCTTTACTACAAAATAAGTTGGAGATGGAAAGTAACGTCCTCTTGGTGTTAGATGATTTATGGAAGGGACTCGATCTAGAAGAGGTTGGAATTCCTTGTAGATCAGAGTCATGTGAAAAGGGATGTAAGATACTCATAACAAGTCGAGATAGAGATGTCTTAACTAATGAAATGGACACACAAGTTTATTTCGAGGTGAAGCCTTTAAGTGAAAAGGAATCATGGGAGTTTTTCAAGAACATGATTGGTGAGTTTGATAATAAATGCATAGAACTAATAGGGAAAGAGATGGTGAAGAAGTGTGGGGGATTGCCAATAGCACTTGCTACAATTGTAAAAACTTTGAAGGGGAAGGAAGTGCCTATTTGGAAGGATGCTTTGAAGCAATTGAAAAATCCTATTGCAGTGGATGTTAAAGGGGTGACTGATGTATTCCCAGATGATTATGAGATTTCTGTGGAAGACTTGCAAATATATGCCATGAGTTTGAGATTGTTAAACCAAGTGAATACCTGGGACGAAGCTAGAAACAGGGTAATTAAATTGGTTGATGATCTTAAAGCTTCTTCTTTACTTCTAGAATCCAATTCAAGGGATAATCATGTTAAAATGCATGATATCGTTCGTGATGTGGCAATATACATTGCATCAAAGGAAGCTAATATGTCTACATTGAGCTATGGTTTCGGGCTGAGTGAATGGCAAGAAAAGGATAGACATGGATTCTATAGAGCAATCTTTGGAAACTGTCACAACTTCTACAACTTTCCTCAAAACTTAGAGTTTCCAAAACTTGAATTGTTGATATTAGATGGACATGATTGGAGGGGAGAAAAACTTCAAATTTGTTACTCTTTTTTTGAAGGAATGAAAGAACTTAAAGTTTTGAACTTGTCAAGGATGTGTTTTCAACTACTAAGGAGGCCATCAATCCACTCTCTAGAAAATCTTCAAACATTATGCATGTCACATTGTACATTCAATGACATTGATGCAATAAGTCACCTAAAGAAGCTACAAATTTTGAGGATTGATAAATGTCCAATCACATTGTTACCTAAAAGCATGAGTCAATTGACACAACTTAAGGTACTACAAGTGTCAAATTGCCCTTTGAAAGTGATTCCTCCAAACACCCTTTCAAGCCTTTTGAAGTTACAAGCACTAGATATATGGACAAGCTTCAATGGATGGGGAGAAGAAGTATCACACAATAACAAATTGATCAATAATGCAAGGCTATCAGAGTTGAAGTGTCTACCACATCTAACAAATTTAAAGATACATATCTTGGACATCAAAATTCTTTCAGATCTAATATTTCTGAAAAATTTGAAGCTAGAAAGATTTGTTATCCATGTTGGTGAATTGAAAATGTCCCAAAGGTTGCAAGGATGTGAACAATATGCAACAACTTTGATGCTTAAAATCATAACATCATCATCCCAAATTGTTTCAATCGATCATCATGAGTTCTTGTCCCTTGAAAAAATGGAGAGCTTGGAGAATATAGTTCATGCAGATGTGTTTACAAGTCCTTTCAGAAAGTTGAGATCCATAAAAGTAATAAGTTGTAAGAGATTAAGGTATCTCTTCTCTTTCTCTATCTTTAAAGGCCTTGTGGATCTACAAAGGGTTTTTATATTTGATTGCAACATGATGGATGAGATACTTTGCATGGATAGTGAAGACTCAACAATTGCAGTTGAAGGCAATTCTATTGAGTGTCCTCAATTGAAGGATTTGACAATCATTGGAGCACATAATTTGAAGATGTTATGGCATAAAAATGGGTTGGCTCCAAATTTCTTCAGCAAACTCCAAAGGATAAGTATCAATAGTTGCAACACTTTAAGATACCGTGGG

>Cucsa.237390

ATGGTCCCTTCTTCTCCTTCTTCTTCTGCTGCTTCTTCTTCTTCTCCTTCTTCTTCTCCTTCAATTGGTAAATGGAAATTTGATGTGTTCTTGAGCTTTCGAGGCGAAGATACACGTGGTGGCTTCACGGATCATCTCTACAAAGCCTTAACACGAAAGGGAATTTCAACATTTAGAGATGAAAATGAGATCGAAGAAGGTGAACACATTCCTTCAAATCTGCTGGCTTCCATTGATGCCTCGAGATTTGCCATTGTTGTGGTTTCGGAAGATTATGCATCTTCAAGATGGTGCCTCGAGGAATTGGCTAGGATGTTTGAATGTAAAAAAGAAGTTTTACCAATTTTTTATAAAGTGGATCCCTCTCATGTGAAAAACCAAAGTGGAACGTTTGAAGAAGCTTTTGTTAAACATGAAAAGAGATTTGGAAGAGGTGATGGGAAGGTTCAAAGTTGGAGGACGTTTCTCACCGAGCTTGCTAACACCAAAGCTTGGCTTTCTCAATCTTGGTCACATGAATCAAATATCATTGAAGAAATCACCACAAAAATATGGAAAAGATTGAAACCCAATTTGACAGTCATTAAGGAAGACCAACTAGTTGGAATTAATTCTAAAATAAACAAACTTTCTTCACTTTTGATTCCAAACTCAGATGATGATGATGCTGATGATGATGTGATCTTTGTGGGAATACATGGAATGGGTGGCATTGGTAAGACCACAATAGCTAGGGTCTGTTATGAGCGAATTCGTGACGAATTTGAAGCTCATTGCTTCCTCTCCAACGTTCGAGAGAATTATATCAGAACCCTTGGGAACCTTTCATGTTTACAAACCAAACTCCTTTCAAGCATGTTTTCGCTTAAAAACAATCACATAATGGATGTTGAAGAAGGTACCGCTATGATCAATAAAGCCATTTTTCGAAAAAAGACACTTCTCGTCCTTGACGACGTGGATTCTTCGGATCAAATCAAAGGATTGATTCCAGACAACAACTCTTTTGGCAATGGAAGTAGAGTCATCATCACAACACGGAATGCGGATTTTCTTTCGAATGAATTTGGGGTGAAAAGAATTTTTGAAATGGATGAACTTAAATATGAGGAAGCTCTTCAACTTCTTAGTTTGAGTGCTTTTATGAAAACATGTCCAAAAGAAGGTTACTTGGAACACTCCAAGAAGATTGTAAAGGTTGTGGGAGGCCACCCTCTTGCACTCAAATTGTTAGGGTCGTCTCTAAGAAACAAAAATTTGAGTGTGTGGAATGAGGTGATAGAAGAGGTTGGAGGAGGTGGGAATATTCATGAAAAAATTTTCAAGTGTCTTAAAGTGAGTTATGATGGGTTGGATGAAAGGGAGAGAGAGATATTTCTTGACGTTGCTTGCTTCTTCAATGGGAAGAGAAGAGAAGTTGTAGAAGAGATATTAAATGGATGTGGTTTCTATGCCAAAACAAGGATTGAACTTCTTATTCAAAAGTCTCTCTTAACTCTTTCTTATGACAATAAGTTACATATGCATAATTTATTGCAAGAAATGGGTCGAAAGATTGTTCGGGATAAGCATGTTCGAGATCGATTAATGTGCCACAAAGATATAAAAAGTGTGGTGACAGAGGCATTGATCCAAAGCATATTTTTCAAATCAAGTTCAAAGAATATGGTGGAATTTCCAATTTTGTTTTCAAGAATGCACCAACTTAGGCTGCTTAATTTTCGCAATGTGAGACTGAAAAACAAGTTGGAATATAGCATTCCAAGTGAGTTAAGGTATTTGAAGTGGAAAGGATATCCGTTGGAGTTTCTGCCAATCGATAGCTCTGAAGAATGTAAGCTTATTGAGCTTCACATGTGCCATAGCAATCTCAAACAATTTTGGCAACAAGAAAAGAATTTGGTGGAGCTGAAGTATATCAAACTCAATAGTTCTCAAAAGTTGTCCAAAACTCCAAACTTTGCAAACATTCCAAATCTCAAAAGATTAGAGCTTGAAGATTGCACAAGTTTAGTCAACATTCATCCATCAATTTTCACTGCAGAAAAACTCATATTCTTGAGTTTGAAAGATTGCATCAATCTCACCAATCTTCCTTCTCACATTAACATCAAGGTTCTTGAAGTCTTGATTCTCTCTGGTTGTTCAAAAGTAAAAAAAGTCCCTGAATTTTCAGGTAACACTAATAGATTACTCCAACTCCATTTGGATGGTACCTCCATATCAAACCTACCTTCATCAATTGCAAGCTTGAGTCATCTAACAATATTGAGTTTAGCCAACTGCAAAATGTTAATCGACATTTCGAACGCGATTGAGATGACATCTCTCCAAAGCTTAGATGTTTCTGGATGTTCGAAGCTTGGAAGTAGAAAAGGAAAGGGGGACAATGTCGAATTGGGGGAGGTCAACGTGAGAGAAACCACACGAAGAAGAAGAAACGACGACTGTAACAATATTTTCAAAGAAATCTTCCTTTGGTTATGCAACACTCCAGCTACTGGCATTTTTGGGATCCCATCATTAGCTGGTTTGTACTCTCTTACAAAACTAAACTTGAAGGATTGCAACCTTGAAGTAATCCCACAAGGGATTGAGTGTATGGTGTCATTGGTAGAGCTCGACTTGAGTGGCAATAATTTCTCTCATCTTCCAACAAGCATATCAAGACTTCATAACTTGAAAAGATTGAGGATAAACCAATGCAAAAAGCTTGTACATTTCCCAAAGTTACCTCCAAGGATCTTGTTTTTGACGTCAAAGGATTGCATTTCATTGAAAGATTTTATAGATATTTCAAAAGTTGATAATTTATACATAATGAAAGAAGTGAACCTTTTGAACTGCTACCAGATGGCTAACAACAAAGACTTCCATAGATTGATCATTTCTTCGATGCAGAAGATGTTCTTTCGAAAAGGAACATTCAACATCATGATTCCGGGGAGTGAGATTCCCGATTGGTTTACAACAAGGAAAATGGGATCTTCGGGATGA

>Cucsa.237410

ATGGAGAGAAGAGCTTCAATTAAATCCTTATCTCCTCCTCCCTATTCTATCTCTCTTCCTCTTCCTCCCTTACGAAACTATGACGTTTTCCTCAGCCACAGAGTTAAGGATACCGGGAGTAGTTTCGCAGCTGATCTTCATGAAGCTTTGACAAACCAAGGAATTGTAGTTTTCAGAGACGGCATAGACGACGAAGACGCAGAGCAACCATATGTAGAGGAGAAGATGAAGGCCGTGGAAGAATCGAGGTCTTCGATCGTGGTTTTTTCAGAGAACTACGGGAGTTTTGTTTGCATGAAGGAAGTAGGGAAGATTGTAACGTGTAAGGAGTTGATGGATCAACTGGTTCTTCCTATATTTTACAAAATAGATCCAGGCAATGTGAGGAAGCAAGAGGGGAACTTTAAGAAGTACTTTAATGACCATGAAGCCAATCCTAAGATTGATATTGAAGAAGTTGAGAACTGGAGATATTCTATGAATCAAGTTGGCCATCTCTCTGGATGGCATGTCCAAGATTCCCAGTCTGAAGAAGGGAGCATAATCAATGAAGTTGTGAAGCATATATTCAACAAATTGCGTCCTGATTTGTTTCGATATGATGATAAATTAGTTGGAATTTCCCCAAGATTACACCAAATAAATATGCTTTTGGGAATAGGTTTAGATGATGTACGCTTTGTTGGAATATGGGGAATGGGTGGAATTGGCAAAACTACAATTGCTAGAATCATTTACAAAAGTGTTTCTCATTTATTTGATGGATGTTATTTCTTGGACAATGTCAAAGAAGCTTTGAAGAAAGAAGACATAGCTTCATTACAACAAAAGCTTCTAACAGGAACTCTAATGAAAAGAAACATTGACATCCCTAATGCTGATGGAGCTACATTAATTAAGAGAAGAATAAGTAATATTAAAGCTCTTATAATTCTTGACGATGTCAACCATCTAAGCCAACTTCAAAAATTAGCCGGCGGTTTAGATTGGTTTGGCTCAGGAAGTCGAGTCATCGTTACAACGAGAGACGAACATCTCCTAATTTCACATGGAATCGAAAGACGATACAATGTTGAAGTGCTGAAAATTGAAGAAGGTCTTCAGCTTTTTTCACAAAAGGCATTTGGAGAAGAGCATACAAAGGAAGAGTATTTTGATGTTTGTAGCCAAGTTGTAGACTATGCTGGAGGACTTCCATTGGCAATTGAGGTTCTTGGATCTTCTTTACGTAATAAACCAATGGAGGATTGGATAAATGCAGTGGAAAAGTTGTGGGAAGTTCGTGATAAGGAAATTATAGAAAAGTTGAAAATTAGTTATTATATGTTGGAGAAATCTGAACAGAAAATTTTTCTAGATATTGCATGTTTTTTTAAGAGAAAGAGTAAGAAACAAGCAATAGAAATTCTTGAAAGTTTTGGATTTCCTGCTGTTCTTGGACTAGAAATATTGGAGGAGAAATGTCTTATTACTACACCACATGATAAGCTACATATGCATGATTTAATACAAGAAATGGGCCAAGAAATTGTTCGCCAAAACTTTCTGAATGAGCCCGAAAAGCGAACTAGGTTGTGGCTTCGTGAGGATGTCAATCTCGCACTAAGTCGAGATCAGGGAACAGAAGCAATTGAAGGGATAATGATGGATTTGGATGAGGAAGGAGAATCACATTTGAATGCCAAAGCCTTTTCAGAAATGACAAATCTAAGAGTATTGAAATTGAACAATGTTCATCTTAGTAAAGAAATTGAATATCTGTCTGATCAACTAAGGTTTCTCAATTGGCATGGTTACCCTTTAAAGACCTTACCATCAAATTTCAATCCCACAAATCTATTGGAGCTTGAGTTGCCAAATAGCTCTATTCACCATCTTTGGACTGCTTCAAAG

>Cucsa.237440

ATGACATCCTTATCTTTTCCTCCTCCTCCTCCTCCTCCTTATTCTATCTCTCTTCCTCTTCCTCCCTTACGAAGATATGACGTTTTCCTCAGCCACAGAGCTAAGGACACTGGATGTAGTTTCACTTCCAACCTCCACGAAGCTCTAACAAGTCAAGGAATTGTAGTTTTCATAGACAAGGAAGACGGAGGGAAACCGTTAACGGAGAAGATGAAAGCGGTGGATGAATCGAGGTCTTCGATCGTGGTTTTTACCAAGAATTATGGGAGTTTGGTTTGCATGAAGGAAATAAGGAAGATTAGAATGTGTCAGAAGTTAAGGGATCAATTGGTCCTTCCAGTATTTTACAAAATAGATCCAGGCGATGTGAGGAAGCAAGAGGGGAGCTTTGAGAAGTACTTTAATGAACATGAAGTCAATCCTAATATTAGTATTGAAGAAGTTAAAAAATGGAGAAAATCTATGAACAAAGTTGGCAATCTCTCCGGATGGTCTGAAGAAGGGACCATCAATGAAGTTGTGAATCATATTTTCAACAAATTACGTCCAGATTTATTTCGATATGATGATAAATTAGTTGGAATTAGCCGAAGATTACATGAAATAAATAAGCTAATGGGAATAGGCTTAGATGACGTACGGTTGATTGGAATATGGGGAATGGGTGGAATTGGCAAAACAACCATCGCTAGAATCATTTACAAAAGTGTTTCCCATTTGTTTGATGGATGTTATTTTTTGGACAATGTCAAAGAAACTTTAAAGAAAGAAGGCATAGCTTCTTTACAACAAAAGCTTCTAACAGGAGCTCTAATGAAAAGAAACATTGACATCCCTAATGCTGAAGGAGCTACATTAATCAAGAGAAGAATGAGTAATATTAAAGCTCTTATAATTCTCGATGATGTCGACCATCTAAGCCAACTTCAGCAGTTAGCTGGCGGTTCGGATTGGTTCGGTTCAGGAAGTCGAGTCATCGTTACGACGAGAGAAGAACATCTCCTAATTTCACATGGAATCAAAAGACGATACAATGTTGAAGTGCTGAAAATTGAAGAAGGTATTCAGCTTTTCTCACAAAAGGCATTTGGAGAGGACCATCCAAAGAAAGGGTATTTTGATCTTTGTAGCCAAGTTGTAGATTATGCTGGAGGGCTTCCATTAGCAATTGAGGTTCTTGGATCTTCTCTACGTAATAAACCAATGGAGGATTGGATAGATGCTGTGAAAAAGTTGTGGGAAGTTCGTGATAAGGAAATTATTGAAAAGTTGAAAATTAGTTATTATATGTTAGAGAAAGATGATAGGGAAATTTTTCTAGATATTGCATGTTTTTTCAAGAGGAAGAGTAAGAGACAAGCAATAGAAATTCTTGAAAGTTTTGGATTTCCTGCTGTTTTTGGACTAGACATATTGAAGGAAAAGTCTCTTATTACTACACCACACGAGAAGATACAAATGCATGATTTGATACAAGAAATGGGTCAAAAAATCGTTAACGAAAAGTTTCCAGATGAACCCGAAAAACGAAGCAGGTTGTGGCTTCGTGAGGATATAACTCGTGCTCTAAGTCATGATCAGGGAACAGAAGCAATTAAAGGGATAATGATGGATTTGGATGAGGAGGGAGAATCACATTTAAATGCCAAAGCCTTTTTTTCAATGACAAATCTAAGAATATTGAAATTGAACAATGTTCATCTTAGTGAAGAAATTGAATATCTGTCTGATCAACTAAGGTTTCTCAATTGGCATGGTTACCCTTTAAAGACCTTACCATCAAATTTTAATCCCACAAATCTATTGGAGCTTGAGTTGCCAAATAGCTCTATTCACCATCTTTGGACTGCTTCAAAGGTACATCAAAACAACAGTAGTAATTAA

>Cucsa.237520

ATGTGTCGACCTCCATACTGGTGTTGGGATGATTCATGGATGTTCTTTGATGTTGGCTATACAAAGTTCAAAATCCAATCCATCGCAAATTCAATAGGTGATCACTTGCTTCGTCTTAAGCTCCAAGCCAAAGAAGAAAATTTATTTGAAATGCCACTTCGATTAAGAACAATGAAAATGCTCCTTGGCTTAGGCTCAAATGACGTACGTTTTATAGGGATAGTGGGGATGAGTGGTATTGGTAAAACAACCCTTGCGGAAATGACATATTTACGTATTTTTAAGCCTTTTGTATCTGCCTTACGAAAACCTTACTTTCTTCACTTTGTTGGACGCAGTATAGTCTCCTTGCAGCAACAACTACTTGATCAACTTGCTTTCCTAAAACCTATCGATATCCAAGTTTTGGATGAGAATCATGGAGTAGAATTGATTATGCAGCATTTGAGTTCACTTAAAAATGTGCTTATTGTTTTTGATGGAATAACCGAAAGAAGTCAATTAGAAATGTTAGCTGGCAGCCCCGATTGGTTTGGCGCAGGGAGTCGAATCATTATTACAACCACAAATAAAAATATTTTTCATCACCCTAATTTCAAAGACAAAGTGCAAGAATATAATGTAGAATTACTTTCTCATGAGGCTGCCTTCTCCCTCTTTTGCAAGCTTGCATTTGGAGATCACCCTCATACCCAGAATATGGATGATCTTTGTAATGAGATGATCGAAAAGGTTGGAAGACTCCCATTAGCTTTGGAAAAAATAGCTTTTTCATTGTATGGTCAGAACATAGATGTATGGGAACATACATTGAAGAATTTTCATCAAGTAGTTTATGATAATATTTTCTCTGATGTATTAAAGTCAAGTTATGAAGGATTAGAAGCAGAGAGCCAACAAATTTTCCTAGATTTGGCATGTTTCCTCAATGGAGAGAAGGTGGATAGAGTGATTCAAATACTTCAAGGCTTTGGTTATACCTCACCTCAAACTAATTTGCAATTGTTGGTTGATAGATGTCTTATTGATATTTTAGACGGACATATACAAATGCACATCTTGATTCTTTGTATGGGCCAAGAAATTGTGCACCGCGAGCTGGGAAATTGTCAACAAACAAGGATTTGGCTTCGAGATGATGCTCGTCGTCTATTTCATGAAAACAATGAATTAAAATATATTCGTGGAATAGTGATGGACTTAGAGGAGGAAGAAGAATTGGTATTGAAGGCTAAGGCATTTGCAGATATGTCTGAGCTAAGAATTTTACGAATCAACAATGTGCAACTTTCGGAAGATATTGAATGTCTGTCAAATAAATTGACGTTGCTCAACTGGCCTGGCTATCCTTCAAAGTATTTGCCATCAACTTTTCAACCACCATCTCTGCTTGAGTTACACTTGCCTGGTAGTAATGTTGAACGACTCTGGAATGGAACACAGAATTTTAAGAACTTAAAGGAGATTGATGCAAGTGATTCGAAGTTTTTGGTTGAAACTCCTAATTTTTCAGAGGCTCCAAAGCTTCGACGATTGATTTTACGAAATTGTGGAAGACTAAACAAGGTTCATTCTTCAATAAATAGTCTCCATCGTCTAATTTTATTGGACATGGAAGGTTGTGTCAGTTTCAGAAGCTTCTCATTTCCTGTCACTTGCAAAAGTCTCAAAACTTTAGTTCTTTCGAACTGTGGTCTAGAGTTTTTTCCAGAGTTTGGATGTGTGATGGGATATTTGACTGAACTACACATTGATGGGACTTCCATAAATAAACTTTCTCCCTCAATTACAAATCTACTTGGCTTGGTTTTATTGAACCTGAGGAATTGTATTAGACTTTCTAGTCTTCCAACTGAAATTTGTAGGTTGAGTTCACTTAAAACTCTCATTCTGAATGGTTGCAAAAACTTGGACAAAATTCCACCATGTTTGAGGTATGTAAAGCATCTTGAGGAGCTTGACATTGGCGGAACATCCATAAGCACAATTCCTTTCTTGGAAAATCTAAGAATTTTGAACTGCGAAAGGCTGAAAAGCAATATTTGGCATTCTTTGGCTGGTTTGGCAGCACAGTATTTAAGGTCACTCAACGATTTAAATTTAAGTGATTGTAATCTTGTGGATGAAGACATTCCAAATGATCTTGAACTCTTTTCCTCATTGGAAATTCTAGATCTGAGCAGCAATCATTTTGAAAGACTGTCAGAAAGTATTAAACAACTTATTAACCTTAAAGTATTGTACTTGAATGATTGCAACAAGCTAAAGCAAGTACCCAAGCTTCCAAAAAGTATAAAGTATGTGGGAGGAGAGAAGTCCTTGGGCAT

>Cucsa.237530

ATGGGGAAGCAGACTGATAATAAGCTAGTACTTTCTCACAAAACTAGTTTAGTTGGAATGGAGAATCAAGTGAAGAAAGTTTGTAATCTCCTAGATTTAGAACGATCCAAGGACATACTTTTTGTGGGGATATTTGGATCAAGTGGCATTGGTAAAACAACCATTGCTGAAGTTGTTTACAACACAATTATAGATGAATTCCAAAGTGGTTGTTTTCTTTACCTTTCTTCAAAGCAAAACAGTTTGGTCCCACTTCAGCATCAAATTCTTTCTCATCTTCTATCAAAAGAAACTAAAATTTGGGACGAAGATCATGGAGCACAACTGATTAAGCATCACATGAGTAATAGAAAAGTTGTTATTGTTCTTGACGGAGTTGATGAAAGAAACCAAATTGAAAAGTTAGTTGGAAGTCCGAATTGGTTTGCACCCGGAAGCAGAGTTATTATTACGGCTACAAATAGAGATGTTCTGCATCAACTCAATTATAGAGATCAAGTGCAAGAATACAAGGTGGAGTTACTTTCCCGCGAGAGTGCTTACTCACTGTTTTGCAAGAATGCATTTGGAGATGGCCCATCTGATAAAAATGATCTCTGCAGTGAAATTGTGGAAAAGGTTGGAAGACTTCCATTAGCTTTGAGAACCATTGGTTCCTATTTGCATAATAAGGACTTGGATGTATGGAATGAAACACTGAAGAGACTAGATGAAGAGGAACAAAATTACTTTGATACAATATTGAAGAGAAAT

>Cucsa.237540

ATGCAGAGTTCATCATCGTCTTCTTTGGATCGTCCTAAGATGAACTATGATGTGTTCATAAGCTTTAGAGGTAGAGATGTTCGTCACACTTTTGCAGGATATTTGTACGATGCTTTGAATCGTTTGGGGATAAAAGCTTTCCTGGACAACAAGAGGTTTCTAATTGGAGATGATCTTCATGACTTATTCAAAATAATCGATGAATCAAGATCAGCAATTGTTGTTCTTTCAGAAGACTATGCTTCTGCTAAATGGTGTTTGAGAGAGTTGACTAAGATAATGGATTCCATGGGAACCTCAATGGAGCGTGTCCTTCCTGTGTTTTATCATATTGATCCATCAATTGTTAAAGATCAATCTGGAACTTTTAAGACAAGTTTTGATGAACATGAAGCCAATGTTTTAAAGGAAATTGATAATCAAGAGAAGGAGAAGCGCTTGAAGGAACTCCAGAATTGGAAAAGTGCACTGAAGAAAATTGGCAATCACACTGGAGTTGTCATCACTAAGAACAGTTCTGAGGTAGATATAGTAAATAAAATTGCAAGTCAAATATTCGATGCATGGCGTCCTAAGTTGGAAGCATTGAATAAGAATTTAGTTGGAATGACATCCCGATTGCTCCATATGAACATGCATCTTGGTTTAGGATTAGACGATGTACGCTTCGTTGCGATAGTAGGAATGGGTGGTATTGGTAAAACAACTATTGCTCAAGTCGTTTTTGATTGCATTCTTTCAAAGTTTGAAGATTGCTGCTTTCTAACATTACCTGGAGGTGATTCAAAGCAAAGTTTAGTGTCATTACAACGGGAAATGCTTTCTCAAATTTTTCATAAAGAAGATTTTAGAATATGGCATGAGAATCATGGAGTAGAGATGATTAAAAATCGACTGAGTGGTAGAAAGGTTCTTATTGTTCTTGATGGCATCGAAGAGAGAAGGCAGTTAGAAATGTTGGCTGGAAGCATCGAGTGGTTTGGTCCTGGAAGCAGAATCATCATTACAACTAGAAATAAAGGATTATTGTGCCATCCTAATTATGATGAAATGAAAGTATACAATGTTGAAGAACTAGATCATGATAGTGCCCTTCAACTCTTTTTGAAGCATGCATTTGGTAGTAATCATCAAAACAATGACAGTTTCATGGATCTTAGTAATGAGATAGTTGAGAAGGCTAAAAGACTTCCATTAGCTTTAAGAGTGATTGGATCTTCTTTGTATGGTAAAGATATTACAGTATGGAGAGAAACGTTGAAGAGGCTGATCAAAGTGGATGAAAGAAATTTTTTTGATGTATTGAAAATAAGTTATGATGGATTAGGAGTTGAAAGCCAACAAGTTTTTCTTGACATTACATGTTTCTTCAATGGAAAAAATGAAGATAGAGTAATTGAAATATTAGAGAGTTTTGGTTATAGTCCTAATAGTGAAGTACAATTACTGATGCAAAGATGTTTAATTGAAGTTTCACACAAGAAAATATTGGTGCATGATTTAATTCTTGAAATGGGTCGAGAAATTGTGCGTAAGGAGTCCCTCACTCAAGCAGAAAAACAGAGTAGGATTTGGCTTCATGAAGATCTTTACTGCAGGTTTGCTGAAAAACATGACTTGATGCATATTCAAGGGATAGTTTTAAGTTTGGCAAAAGAAATGGAAGAATCAATAGAATTGGATGCTGAATCCTTTTCAGAGATGACCAAACTAAGAATACTGGAAATCAGTAATGTGGAGCTCGATGAAGACATTGAATATCTCTCTCCACTCTTACGGATAATTAATTGGCTTGGCTATCCTTCGAAGAGTTTGCCCCCAACGTTTCAATCCCGCTATTTGTTTGAACTACTCTTGCCTCATAGTCACCTTTTACGAATTTGGGATGGAAAAAAGAGATTTCCAAAGCTGAAATTAATTGATGTTAGTAACTCAGAACACTTGAGGGTGACACCTGATTTTTCTGGGGTTCCAAATCTTGAGAGATTGGTTCTATGTAACTGTGTTAGACTGTGTGAGATTCATCCCTCCATCAATTCCCTCAACAAACTCATTTTACTGGATTTAGAGGGTTGTGGTGATCTTAAACATTTTCCAGCAAATATAAGATGTAAAAATCTCCAAACACTCAAACTTTCTGGTACAGGTCTTGAAATTTTTCCAGAGATAGGCCATATGGAACATTTGACTCATCTTCATCTTGATGGATCCAATATAACCCATTTTCATCCTTCAATTGGGTATCTAACTGGCTTAGTTTTCTTGGACCTATCCTCCTGTTTAGGTCTTTCTAGTCTTCCTTGTGAAATTGGTAACTTGAAGTCTTTGAAAACCCTCCTTTTGAAATATTGTAAAAAACTTGATAAAATCCCTCCAAGCTTAGCAAATGCAGAATCCTTGGAGACACTTTCTATTAGTGAAACCTCAATAACCCATGTTCCACCAAGCATTATTCATTGTTTAAAGAACCTAAAAACGTTAGATTGTGAAGGACTATCACATGGAATTTGGAAGTCATTGCTCCCCCAATTCAACATTAATCAAACAATAACCACTGGTTTGGGGTGCCTCAAAGCTCTAAATTTAATGGGTTGCAAACTTATGGATGAGGACATTCCTGAAGATCTCCATTGCTTTTCTTCATTAGAAACACTAGATCTCAGCTATAATAACTTCACAACACTCCCTGATAGTCTTAGCCACCTCAAGAAGTTAAAGACATTGAACCTGAATTGTTGCACTGAGCTTAAAGACTTACCAAAGCTTCCAGAAAGTTTGCAATATGAACGATTTAGAAGTAAGTTTGATCTGCTTCTACATGGAGATAAGATCCCAAAATTTTTCAGCAATCAAAGCAAAGGAAACATGACAGAAATAAAGTTACCACAATATTTGGAAAAATTTCGAGAGAGTATAGGTGTTGCTGTGTGTGCTCTTGTGGTTGTGGACAAGAAAAGAAGAAAACTAAATGAGATTATTCCAGAACGAGAGAGGTATACAAAAGTTGTGGATCTTATATGTAAATTCAAAGTTGACTCGTATCAAATTATGCCAGAGCACTGTCACTTCACATCCCAACAAAAACTGTTGAGTGAATATGCTTCACAATTCCTTTGGCTCTCTTACATTCCCCTTCATGGATTTAATATCAATTGGCATTATTGCACCCAGTTTGAAATTGCACTTGAAACTAGCTGTGATGAGCTATTTGGAGTGAAGAATTGTGGTCTTCATCTCATACATAAGCATGAAAGGATGATGATTGATAAGATGGTAATGGAGTCAACTGTTCCATCATCCACTAGCCACAAAGGAAAGGAACCTCAAATTCATTGA

>Cucsa.237560

ATGGCTTCCTCAACCCCCAAGGAATTATCTTCTTTTTCTTCTTCTCCTAGATTCATATTTGACGTCTTTCTCAGCTTCAGAGGCGTCGACACTCGCAAGAATGTCACAAATCGTCTTTACGAAGCTCTGAGGCGACAAGGCATCATTGTTTTCAGAGATGACGATGAGCTCGAGAGAGGGAAGACTATTGCTAACACTCTAACCAACTCGATTAACCAATCCAGGTGTACCATTGTTATTCTCTCTAAAAGATATGCAGATTCAAAATGGTGCTTGAGGGAGTTGGTTGAGATTGTCAAATGCAAGAATACCTTCAAGCAATTAGTTCTTGTGGTTTTCTACAAAATTAAGCCCTCCGATGTCAACAGCCCTACTGGGATTTTTGAGAAATTTTTTGTTGATTTCGAAAATGATGTTAAGGAGAATTTTGAAGAGGTTCAGGACTGGAGGAAGGCCATGGAAGTGGTTGGAGGTCTCCCTCCATGGCCTGTAAATGAACAGACCGAAACAGAGAAAGTCCAAAAGATTGTTAAGCATGCTTGCGATCTTCTGCGTCCTGATTTGCTTAGCCATGATGAGAATTTGGTTGGCATGAACTTGAGATTAAAAAAAATGAATATGCTTATGGGCATAGGACTGGATGATAAGCGCTTTATTGGGATATGGGGGATGGGTGGAATAGGCAAGACAACTATTGCTAAAGCTGTTTTCAAAAGTGTCGCTCGTGAATTCCATGGAAGTTGCATTCTGGAAAATGTTAAGAAAACTTTAAAGAATGTTGGAGGCTTGGTGTCCTTGCAGGAGAAACTTCTTTCCGATACTCTAATGAGAGGAAAAGTTCAAATTAAAGATGGCGATGGAGTTGAAATGATAAAGAAAAACTTAGGAAATCAAAAAGTTTTTGTTGTTCTTGATGGTGTTGATCATTTTAGCCAGGTGAAAGATCTGGCAGGAGGAGAAGAATGGTTTGGTTGTGGAAGTAGAATCATCATTACAACAAGAGATGAAGGTTTGCTTCTTTCTCTTGGAGTTGATATAAGATACAATGTTGAGAGTTTCGATGATGAAGAGGCTCTTCAGCTCTTTTGCCATGAAGCATTTGGAGTAAAGTTCCCTAAGAAAGGTTATTTGGATCTTTGTATGCCATTTATAGAATATGCTGAGGGCCTTCCATTAGCAATCAAGGCTCTTGGGCATTCTTTGCACAATAGATTGTTTAAGTCATGGGAAGGTGCTATTAGAAAGTTAAATAATTCTTTAAACAGGCAAGTATATGAAAACTTGAAAATTAGTTACGATGCACTTGGAAAGGAAGAGAGGAGAATTTTTTTGTATATTGCCTGTTTTCTTAAAGGACAGAACAAAGACCAAGTCATTGACACATTCGTGAGTTTTGAAATTGATGCTGCTGATGGGCTTCTTACCAGAAAAAATGCTGCCGATGTACTTTGTATAAAAGAAACTGCTGCTGATGCTCTAAAAAAATTGCAGGAGAAATCCCTTATAACTATGTTATATGACAAAATAGAGATGCATAATTTACACCAAAAACTAGGTCAAGAAATTTTTCATGAGGAGTCATCGAGGAAAGGTAGTAGGCTATGGCATCGAGAGGATATGAACCACGCTTTAAGGCATAAACAGGGAGTTGAAGCTATTGAAACCATTGTCTTGGACTCAAAAGAGCATGGAGAGTCACACTTAAATGCCAAGTTCTTTTCAGCAATGACCGGTCTAAAAGTGTTGCGTGTTCATAATGTATTCCTTTCTGGAGTTCTTGAATATCTCTCAAACAAGTTGAGACTTCTCAGTTGGCATGGATATCCCTTCAGAAATTTACCATCGGATTTCAAGCCGAGTGAACTATTGGAACTCAATTTACAGAATAGCTGCATTGAAAATATTTGGAGAGAAACAGAGAAGTTGGATAAATTGAAGGTAATTAACCTTAGTAATTCCAAGTTCTTATTGAAGACCCCTGACCTGTCAACGGTGCCAAATCTTGAGAGGTTGGTCTTGAATGGTTGTACAAGACTACAAGAGCTTCACCAATCTGTCGGCACTCTAAAGCATCTAATCTTTTTGGATCTTAAGGACTGCAAATCTCTCAAAAGCATTTGTTCTAATATTTCTCTTGAATCACTCAAGATTCTCATTCTTTCTGGTTGTTCAAGACTTGAAAATTTTCCAGAGATTGTGGGAAACATGAAACTTGTGAAAGAGCTTCATTTAGATGGCACTGCTATTCGAAAATTGCATGTCTCGATTGGAAAACTTACAAGCCTTGTTTTGTTGGATCTTAGATACTGCAAAAATCTTCGTACACTTCCAAATGCAATCGGTTGCTTAACATCCATTGAACATCTCGCATTGGGTGGCTGCTCAAAGCTTGATAAAATTCCTGACAGCTTGGGGAACATTTCTTGTTTAAAGAAACTTGATGTGAGTGGTACTTCTATTAGTCATATCCCATTTACTCTAAGACTTTTGAAGAACCTTGAAGTATTGAATTGCGAAGGCCTATCCCGAAAATTATGTTATTCGTTGTTCCTATTATGGAGTACGCCGAGGAATAACAATTCACATTCATTTGGTTTGTGGTTGATAACTTGCTTAACGAATTTTAGTTCGGTAAAGGTTTTGAATTTTAGTGATTGCAAGCTGGTAGATGGAGACATACCCGACGACCTCAGCTGTTTGTCTTCATTGCACTTTCTGGATCTAAGCAGGAACCTCTTCACCAATCTGCCTCATAGTTTGAGTCAACTTATCAATCTCAGATGCCTTGTTTTGGACAACTGCAGTAGACTCAGGTCATTACCGAAGTTCCCAGTCAGTTTACTTTATGTACTCGCAAGGGATTGTGTGTCACTGAAAGAACACTATAACTATAACAAAGAAGATCGCGGGCCTATGAGCCAAGCAGAAGTAAGGGTCCTTAGTTACCCCTCATCAGCTAAAGACCAAAACTCTAAAATCTCTCAGTTAATGATATCAAGTATGTGCACAGCTTGCGAGAATGGGGGTTGA

>Cucsa.239860

ATGGGAGGTGTTGGAAAAACATATTTGCTCAAGGAAGTTAAGAAATTGGTGTTGGAAGAAAAATTGTTTGATCTAGTGATTGATGTGACTGTAGGTCAATCTAATGATGTAATGAATATGCAACAACAAATTGGAGACTTCCTCAATAAAGAATTGCCAAAGAGTAAGGAGGGAAGAACATCCTTTCTACGAAATGCATTGGTGGAAATGAAAGGTAATATCCTGATCACATTCGATGATTTATGGAATGAATTTGATATCATAAACGATGTTGGAATTCCGTTAAGTAAAGAAGGATGTAAGACACTTGTCACAAGTCGTTTTCAAAATGTTCTAGCCAATAAAATGAATATAAAAGAGTGTTTTAAGGTGACTTGTCTAGACGATGAAGAGTCTTGGAAGTTTTTTAAGAAAATTATTGGTGATGAGTTTGATGCAAAAATGGAAAACATTGCAAAGGAAGTGGCCAAACAATGTGGAGGATTACCACTTGCACTTGATATCATTGCAAAAACATTAAAGAGATCAAGACATATAAATTATTATTGGGAGGGAGTGTTAAGTAAGCTGAAAAATTCAATTCCGGTGAATATTGACGTGGGTGAAAAAGTTTATGCTTCACTTAAACTAAGCTATGAACATTTGGATGGAGAAGAAGTCAAATCACTATTTCTTCTTTGTAGCGTATTTCCAGATGATCATGGGATTTCAGTAAACGATCTGCAAATGTATGTGATGGGTATGGGACTATTGAAAATGGTAAATACTTGGAAGGAAGCAAGAGCTGAAGCACATTACTTGGTCGAGGATCTTACATCATCTTCTTTACTTCAACGACTTAAGAATAGAGATGTTAAAATGCATGATATAGTTCGTGATGTTGCAATATACATTGGACCAGACTTTAACATGTCTACACTTTACTATGGATATAGTACAAGTAGCAAAGGGCTAGATGAGGATAAATGTAGATCTTATCGTGCAATCTTTGTAGACTGTAAGAAGTTTTGCAACCTTCTTCCAAACTTGAAGCTTCCAAAACTAGAATTGTTAATATTAAGTTTTCCTTTTTGGGGGAAAGATAGAAATATTGACATTATGGATGCATATTTTGAAGGAATGGAAAATCTTAAGGTTTTGGACATTGAAGGAACAAGTTTCCTTCAACCATTTTGGACACCGTTAAAGAACCTTCGAACGTTATGTATGTCATATTGTTGGTGTGAGGATATTGATACAATTGGGCACTTAAAGCAATTGGAAATTTTGAGGATTAGTAATTGTAGAGGCATCACAGAATTACCAACGTCTATGAGTGAATTGAAACAACTTAAGGTATTAGTTGTGTCGCATTGCTTCAAGTTGGTGGTGATTCACACAAACATTATTTCAAGCATGACCAAATTAGAAGAGTTGGATATACAAGACTGCTTTAAGGAATGGGGAGAAGAAGTAAGGTACAAGAACACATGGATTCCAAATGCACAACTTTCAGAATTGAATTGTCTGTCACATCTTTCTATTTTAAGAATTGTTTCAGTCAACGGGACGAAACTTAGCATATTATTAGAAGGAACTAAAAGGTTGATGATTCTAAATGACTCCAAAGGTTTTGCAAATGATATTTTCAAAGCAATTGGAAATGGTTATCCCCTGTTGAAGTGTCTTGAAATTCACGATAATTCAGAGACACCACATTTGAGAGGAAATGATTTCACATCTTTGAAGAGGTTGGTTCTTGATAGAATGGTGATGTTGGAGAGTATTATTCCGAGGCATTCTCCAATAAATCCTTTCAACAAACTTAAATTCATAAAAATAGGAAGGTGCGAGCAGCTAAGGAATTTTTTTCCACTCTCTGTTTTTAAAGGGCTTTCAAATCTTCGACAGATTGAGATCTATGAATGTAATATGATGGAGGAGATTGTATCAATAGAAATTGAAGATCATATCACTATTTACACTTCTCCTTTGACATCTTTACGCATCGAGCGTGTGAATAAACTTACAAGTTTTTGCAGTACCAAATCATCCATCCAACAAACAATTGTTCCCTTATTTGATGAACGACGGGTTTCATTTCCTGAATTGAAGTATTTATCAATTGGTAGAGCAAACAATTTGGAGATGCTATGGCATAAGAATGGAAGTTCCTTTTCCAAACTTCAAACAATAGAGATTAGTGATTGCAAGGAGTTGAGATGCGTGTTTCCTTCAAATATAGCGACGTCACTTGTCTTTTTAGATACATTGAAAATCTATGGTTGTGAGTTATTGGAAATGATATTTGAAATTGAAAAGCAGAAGACTTCGGGAGATACAAAAGTAGTGCCATTGAGATACTTATCTTTAGGATTTCTAAAAAATTTAAAGTACGTGTGGGACAAAGATGTTGACGATGTTGTGGCATTTCCAAACCTAAAGAAAGTTAAGGTTGGTAGATGCCCTAAGTTGAAAATTATTTTTCCAGCTTCCTTCACCAAATATATGAAAGAAATTGAAGAGTTAGAAATGGTTGAGCCGTTTAATTATGAAATATTTCCAGTGGATGAAGCATCAAAGTTAAAAGAGGTTGCATTGTTCCAAAGCTTGGAAACATTGAGAATGAGTTGTAAGCAGGCTGTAAAAGAGAGGTTTTGGGTTATGTCAAAGTTCTTCAAACTCAAAAGTCTTGAATTGTTTGGTTGTGAAGATGGTAAAATGATTAGCTTGCCGATGGAAATGAATGAAGTATTATACAGCATTGAAGAATTGACAATTAGAGGATGCCTCCAGCTGGTAGATGTAATTGGAAATGACTATTATATCCAAAGATGTGCAAATTTGAAGAAGTTAAAATTGTATAATCTTCCGAAGCTTATGTACGTGTTGAAGAACATGAATCAAATGACTGCAACCACATTCTCCAAGTTGGTTTATCTTCAAGTAGGTGGTTGCAATGGAATGATAAATTTATTTAGTCCTTCAGTGGCAAAGAATCTAGCGAATCTCAATTCCATTGAAATATATGATTGTGGAGAAATGAGAACCGTAGTTGCAGCAAAAGCAGAGGAAGAAGAGGAAAATGTTGAAATTGTGTTCAGCAAGCTAACTGGTATGGAATTCCATAATTTAGCAGGATTGGAATGTTTTTACCCTGGAAAATGCACACTTGAATTCCCCTTATTAGATACGTTGAGGATAAGCAAATGCGATGACATGAAAATCTTTTCATACGGAATAACAAACACTCCCACTTTGAAAAACATCGAGATTGGAGAACATAACTCATTGCCATTTACTAGTATATGTGGTGTACATACTATACTTAGTGAAAAAGAAGGTATCATTAACTTGATGGCTTAA

>Cucsa.248810

ATGGCTGATTTCCTATGGACTTTTGCTGTCGAAGAAATGTTGAAGAATGTGTTGAAGGTTGCAGGGGAGCAAACTGGCCTTGCATGGGGCTTCCAGGAGCATCTCTCCAACCTCCAAAAATGGCTACTCAACGCTCAAGCTTTCTTACGCGATATCAACACCAGAAAACTACATCTTCATTCTGTGAGCATCTGGGTGGACCATCTTCAGTTTCTTGTTTATCAAGCCGAGGATCTATTAGACGAAATTGTTTATGAACATCTTCGACAAAAGGTCCAAACAACAGAAATGAAGGTGTGTGATTTCTTCTCTCTTTCTACCGATAATGTTTTGATCTTTCGTCTTGACATGGCAAAAAAAATGATGACCCTTGTACAACTGTTAGAAAAGCATTACAATGAGGCTGCTCCTTTAGGACTAGTTGGGATTGAAACTGTAAGACCCGAGATCGATGTTATTAGTCAATATCGAGAGACAATTTCAGAACTTGAAGATCATAAGATTGCGGGGAGGGATGTTGAAGTTGAAAGTATAGTGAAACAAGTGATTGATGCTAGCAATAATCAACGTACATCTATCCTGCCCATTGTTGGTATGGGTGGATTAGGAAAAACAACTTTGGCAAAGTTAGTTTTTAACCATGAGTTGGTTAGACAACGTTTTGATAAAACTGTATGGGTTTGTGTGTCTGAACCATTTATTGTCAACAAGATTTTGCTTGATATTTTAAAAAATGTAAAAGGTGCCTATATTTCTGATGGAAGGGATAGCAAGGAGGTTTTACTTCGTGAACTCCAAAAAGAGATGCTTGGGCAAAGCTATTTTCTTGTGCTTGACGATGTTTGGAACGAAACTTTTTTTCTATGGGATGACTTGAAATATTGTTTGCTCAAGATCACTGGAAACTCTAACAATAGTATCCTTGTGACTACAAGGAGTGCTGAAGTTGCAAAAATCATGGGAACATGTCCTAGTCATCTTTTAAGTAAATTATCTGATGATCAATGTTGGTCTTTGTTTAAAGAAAGTGCAAATGCATATGGACTATCAATGACTTCAAACTTGGGGATCATTCAAAAAGAGTTGGTCAAAAAAATTGGTGGCGTACCATTGGCTGCACGAGTTTTGGGTAGGGCAGTAAAATTTGAAGGAGATGTTGAGAGATGGGAGGAAATGTTGAAAAATGTGCTAACAACTCCACTGCAAGAGGAAAATTTTGTTTTATCTATATTAAAATTAAGTGTGGATCGTTTACCATCATCTTCAGTAAAGCAGTGTTTTGCATATTGTTCAATTTTTCCCAAAGACTTTGTGTTTGAAAAACAAGAATTGATTCAAATGTGGATGGCCCAAGGTTTTCTTCAACCACAACAAGGAAGATACAATAACACAGCAATGGAAAATGTAGGAGATATATACTTCAACATCTTGTTGTCACGTTGCTTATTTGAATTCGAAGATGCCAATAAAACAAGGATAAGAGATATGATAGGTGATTATGAAACAAGAGAAGAATATAAGATGCATGATCTTGTACATGATATTGCAATGGAAACTTCAAGGTCGTATAAAGATTTGCATCTAAATCCTAGCAATATATCGAAGAAGGAACTTCAAAAGGAGATGATAAATGTTGCAGGCAAGTTACGCACAATTGATTTCATTCAAAAGATTCCTCACAATATAGATCAAACACTTTTTGATGTTGAGATAAGAAACTTTGTTTGTTTGCGTGTTTTGAAGATATCGGGTGATAAATTACCAAAGTCAATTGGTCAATTGAAACACTTGAGATATCTAGAAATTTTAAGTTATTCAATAGAATTAAAATTACCAGAGTCTATTGTTTCACTTCATAATTTGCAAACGCTAAAGTTCGTATACTCAGTGATTGAAGAATTTCCAATGAACTTTACAAATTTGGTAAGTTTAAGGCACTTGGAATTAGGGGAAAATGCTGACAAAACACCTCCACATTTAAGTCAATTGACTCAACTTCAAACATTGTCTCATTTTGTAATCGGATTTGAAGAAGGTTTTAAGATTACTGAATTGGGTCCATTGAAAAACTTGAAAAGATGTCTGTGTGTTTTGTGTTTGGAGAAAGTTGAAAGTAAAGAGGAAGCAAAGGGAGCAGATTTGGCAGGAAAGGAGAATTTAATGGCGCTACACTTAGGGTGGTCCATGAATAGAAAAGATAATGATTTGGAAGTGTTGGAAGGACTTCAACCAAACATAAATCTCCAATCATTGAGAATCACCAACTTTGCTGGAAGACATTTGCCTAACAATATTTTTGTTGAGAATTTAAGAGAGATACATTTGTCTCATTGTAATAGTTGTGAAAAGCTTCCAATGCTTGGACAACTAAACAACCTAAAGGAACTTCAGATTTGCAGCTTTGAAGGCCTCCAAGTTATAGACAACGAGTTCTACGGCAATGATCCAAACCAAAGAAGGTTCTTCCCAAAGCTTGAGAAATTTGAAATCAGTTATATGATCAACTTAGAGCAATGGAAAGAAGTAATAACAAATGATGAATCATCAAATGTCACAATTTTCCCCAATCTCAAGTGCTTGAAAATATGGGGATGTCCCAAATTATTAAACATTCCAAAAGCTTTTGATGAGAATAATATGCAACACCTTGAATCATTGATCCTTTCATGTTGTAACAAATTGACAAAACTCCCAGATGGATTACAATTTTGTAGCTCTATTGAAGGGTTGACAATAGACAAATGTTCAAATTTGAGCATAAATATGAGAAATAAGCCGAAATTATGGTATTTAATCATTGGTTGTGTAACGCAAATTCCTGAACAACTTCAACACCTCACTGCCTTACAATTTCTGTCTATTCAACATTTTAGATGCATTGAAGCTTTGCCAGAATGGTTAGGAAACTATGTATGTTTGCAAACACTCAATCTTTGGAATTGCAAAAAATTGAAAAAACTGCCTTCTACAGAAGCAATGCTACGTCTCACCAAATTAAATAAATTGCATGATCTTAATATTTGTTTATCACCTTCATTCTCATCTCTCTTGTGCTTTTTAATTAATTAA

>Cucsa.249360

TAAATCTAAAGCGATTACTACGAGTAGCGAAACATCTAGTCGAAATTGATTCTCAACTAAGAGAAATTGAGGAGTCGGTCTCTCATATTGGGTCCGAGGGAATTGGTAAGACCACTTTGGCTAAGGATTTGTACAACAAAATTGCTACCCAATTTGAAAGATGCTGCTTTCTACAAGATGTTAGACGAGAAGCTTCAAAGCAATATGGGCTCGTTCAACTACATGAAACCTTACTCTGTGAGATTTTAAAGGAGGATTTGAAGGTTGTCAATTGTGATAAAGGAATTAACATCATAAGAAGTAGACTGTGTTTAAAGAAAGTTCTTATAGTTTTTGATGATGTGGATCATCACAGGCAATTAGAAGCACTAGTTGGTGAGCTCGATTGGTTTGGTCGAGGTAGTAAAATCATTATGTTGACAAGGAATGGACATTTACTTTCTAGCCATGGATTCGATGAAAAGCATAAAATTCATGAATTGGATCAAGACCATGCTCTTGTTCTTTTTAGCCTTTCAGAAAGTGCTACAAATTATTGTAAAGGTCTCTCTGTGGCACTCGTTGTTTTGGGTTCTTTCCTTCGTGGTAGAGATCAAACAAGATGGAGTTGTATATTAGATGAATTTGAAAACTCTCTACCAAAAGATATTAAAGATGTTCTTCAATTAAGTTTTGATGGACTAGAAGACAAAGCAAATGATATTTTTCTTGATATTTCTTTGCATGTCATTCGACCGTTAGATTTTGGAATTATGATTCTCATGGATCTTTCACTTATTATGATTGAAAGTGATAGAGTGCAAATGCATGGATTAATACAACAAATGGGTTGTAGCATAGTTCGTAATGAATCATCTCAACCTGAAAAGAGGAGTAGGTTGTGGTTGGTTCAGGATATTGGGGAGGTATTCGTTAATAAGTCTGTGAGAAACTCTTTACCTAAATTGAAGCATGTTGATCTTAGCTACTCTACTTTATTAGAGAAAATTCTGGATTTCTCTGCTGCATCAAACCTTGAAGAATTGTATCTCACCAATTGCACAAATTTAGGAATGCTAGATAAGTCTATTTTATCTCTCAATAAGCTTACTGTCCTAAACTTTGAAGGTTGTTCTAACCTTAAAATGCTTTCAAGAGGCTACTTCATGTTAAGTTCTCTTAAAGAATTGAGGCCCTCTTACTATAAGAAGCTTGAGAAAATTCCAGACTTATCTGCAGCATCAAACCTTAAGAGATTGTATCTCCAAGAATGCACAAATTTAAGAGTGATTCATAAATCTGTTGGATCTTTGGATAAGCTTGGATTGTTGGACCTTAGTCAATGTACTAACCTAGTAAAGCTTCCAAGCTATCTCAGGTTAAAGTCTCTATACACTTTATATCTTTCTGGGTGTTGTAAGCTTGAAAGCTTCCCAACAATTGCTGAAAACATGAAATATTTAGAGGAATTGTATTTGAATTTTACTGCCATTTAG

>Cucsa.251930

ATGGCTGATTTTATATGGACATTTGCACTGCAAGAGATTCTCAAGAAGACATTGCACCTTGCAACCCAACAAATCCGTCTGGCCTCCGGTTTCAACCACGACCTCTCTAAACTCCTCCACTCATTGCTCTTCTTCGAAGCCATTCTTCGCGATGTCGATCGAACAAAATCCGACCTACAGTCGGTCAAGATTTGGGTCACTAAGCTTCAGGATTTAGTGCTCGATGCTGAAGTTGTGCTGGACGAGCTCTCCTACGAGGACCTTAGGCGAGAAGTGGACGTCAATGGAAATTCGAAGAAAAGAGTACGCGATTTCTTTTCGTTCTCGAATCCCTTGATGTTTAGGTTGAAAATGGCGCGTAAAATTAGAACCATCACCCAAGTTTTGAATGAGATTAAAGGGGAGGCTAGTGCTGTTGGGGCTATTCCTACAGGGGGCAGTGATGAAATAGTGGCTGATAATGGCCATATTCCGGAGACTGACTCATTTCTTGATGAATTCGAAGTTGTAGGAAGAAGGGCTGATATATCTAGAATAGTGAACGTTGTTGTTGATAATGCCACTCATGAAAGGATCACTGTGATTCCTATTGTGGGAATGGGTGGTCTTGGAAAGACAACTTTGGCAAAAGCAGTTTTCAACCATGAGCTTGTGATAGCACATTTTGATGAAACTATTTGGGTGTGTGTGACTGCAACTTTTGATGAAAAGAAGATTTTAAGAGCAATTTTGGAATCTCTAACGAATTTTCCAAGTGGTTTGGATAGTAAGGATGCTATACTTAGAAGGCTACAAAAGGAGCTGGAAGGGAAAAGGTATTTTCTTGTGCTGGATGACGTGTGGAATGAAAATGTTAAACTGTGGAACAATTTCAAGAGTCTTCTGCTAAAGATTACAAATAGTATTGGGAACAGAGTTCTTGTGACAACTAGAAGTGAGGAAGCTGGAAAAATCATGGAAACATTTCCCAGTCATCATGTAGAAAAGTTATCGGATGATGAATGCTGGTCAATATTCAAGGAAAGAGCATCGGCAAATGGATTACCACTGACTCCAGAATTGGAAGTTATTAAGAATGTGCTTGCAGAGCAGTTTGGAGGCATTCCATTGGTTGCAAAAGTTTTGGGAGGGGCTGTACAATTTAAGAAAAGAACAGAGACTTGGTTGATGTCAACATTGGAAACCCTTATAATGAATCCACTTCAAAATGAAAATGACGTTTCATCTATTTTGAGATTAAGCGTGGATCATCTGCCAAACTCATCATTGAAACAATGCTTTGCCTACTTTTCTAATTTTCCCAAGGGTTTTAACTTTGAAAAGGAACAACTAATCCAATTTTGGATGGCAGAAGGGTTCATTCAACCTTCTGATAAAGTAAACCCCGAAACCATGGAAGATATAGGAGATAAATACTTCAATATCTTGCTGGCTCGTTCCTTATTTCAAGATATTGTTAAAGATGAGAATGGTAAAATTACACACTGTAAGATGCATCATCTTCTACATGATCTTGCTTATTCTGTCTCAAAATGTGAAGCACTGGGTTCGAATCTTAATGGTCTGGTTGATGATGTTCCTCAAATTCGACGATTATCCCTGATTGGCTGCGAGCAAAATGTAACGTTGCCTCCTAGAAGGAGCATGGTGAAGTTGCGTTCTCTATTTTTGGATAGAGATGTGTTTGGCCACAAGATTTTAGATTTCAAGCGTTTGCGTGTTCTGAACATGTCCCTATGTGAAATCCAAAACTTACCAACTTCAATCGGAAGGTTAAAGCATCTAAGGTATCTTGATGTCTCAAATAATATGATAAAGAAACTTCCAAAATCTATTGTTAAGCTTTATAAATTGCAGACCCTGAGGCTGGGTTGTTTCCGTGGAGAAGCCCCCAAAAAATTCATAAAATTGATCAGCTTGAGACATTTCTATATGAATGTTAAAAGACCAACAACTAGGCACATGCCTTCGTATTTAGGCAGGTTGGTTGATCTTCAATCCTTGCCTTTTTTTGTTGTTGGGACAAAGAAGGGTTTCCATATAGAAGAGCTTGGATACTTGAGGAATCTCAGAGGTAAATTAAAGCTTTACAATCTTGAATTAGTAAGAAATAAGGAGGAAGCCATGAGGGCAGATTTGGTGAAAAAGGATAAGGTGTACAAATTGAAACTGGTATGGAGTGAAAAAAGAGAAAATAATAATAACCATGACATTTCTGTTTTAGAAGGACTTCAACCACACATCAATCTTCAGTACTTGACAGTTGAAGCCTTTATGGGAGAACTTTTTCCAAATCTTACTTTTGTTGAAAATTTGGTACAAATTTCTCTAAAAAATTGTAGCAGATGTCGAAGAATTCCAACATTTGGACATCTACCCAATCTTAAGGTTCTTGAGATTTCTGGATTACACAACCTAAAATGTATAGGAACAGAGTTCTATGGGAATGAATATGGAGAAGGAAGTTTGTTTCCAAAATTGAAAAGATTTCATCTTTCAGACATGAATAATCTTGGACGTTGGGAAGAAGCAGCAGTGCCAACAGAAGTTGCAGTTTTTCCTTGTCTTGAAGAGTTGAAAATTCTCGACTGTCCTAGACTAGAAATTGCACCTGATTACTTCTCAACTCTTAGGACATTAGAAATTGATGATGTCAACAACCCAATTTCACAGATCACTCTTCAGACATTCAAACTACTTGGTATTATACACTCTGGCAACCTGAGTGGTTTGCCTGAGGAGTTACGTGGTAATCTGTCATCTCTTGAGGAGTTTAAGGTTTGGTATTATCTTCACTTGAAATCCTTTCCAACTATTCAGTGGCTCACTGATATTTTGAAAGGCAAGACCGGATATGACACAAAGTGGACAAATATTCAATCTCATGGGCTAGAATCGTACACTTCTGTGAATGAATTGTCCATTGTTGGGCACTCTGATCTCACATCAACCCCAGATATAAAAGCTTTATATAATCTTTCGTCTTTAACAATTAGTGGCTTGAAGAAATTGCCAAAAGGATTTCACTGCCTCACTTGCTTGAAAAGTTTGTCAATTGGTGGATTCATGGAGGGGTTTGATTTTAGGCCTCTTTTGCATCTCAAGTCTCTTGAAAATCTTGCAATGATAGACTTTATCCTTGCAGAAAGCACACTTCCTGATGAGCTTCAACACCTAACTGGCTTAAAGCACTTGAAAATTGTTGGATTTCAGGGCATTGAATCTCTGCCAGAGTGGTTAGGAAATCTTAACTCATTGGAAAGTTTGCACATTGAGAGTTGCAGAAAATTGAGAGAGCTTCCAGAAGCCATGGGTTGCCTTGCCAAATTGGAGGAAGTGCGGAGTTTCAATTGCCCAGAGTTGAGGGTTTACCAAGACGAATCAGAATGGGCGAAGATTTCTTACATTCCAAGATTCATATCATTCAATTATTGGGTTGATGAGGACCAACAAAGGATTCAGTTCAAAGTTTGCCACAAACCTAGCTAA

>Cucsa.275630

CACAATCATCATCGTCTTGTTCTTCAAATTTGAAATGGAGTTATGATGTGTTTTTGAGTTTCAGAGGTGAGGATACTCGAAACAACTTCACTAGTCATCTTGACAGGGCCTTGCGTGAAAAGGGTGTCAATTTCTTCATAGATGACAAGCTAGAGAGGGGTGGTCAAATTTCTGAATCCCTTCTCAAATCTATTGATGGTTCTAAAATTTCCATCATTATTTTCTCCAAAAATTATGCATCTTCCACCTGGTGTTTGGATGAACTGGTGAAAATAGTTCAGTGCATGAAATCCATGGGACATATAGTTTTTCCTGTCTTCTACAAGGTGGATCCATCTGAGGTTCGAAAACAAACTGGTGGGTTTGGTGAAGCATTGGCCAAACATGAAGCTAATGAGTTAATGACCAACAAGGTTCAACCATGGAAGGAGGCTTTGACCACTGCTGCTTCTTTGTCTGGTTGGGATTTAGCAACTAGGAAGAATGAGGCTGATCTTATTCATGACCTTGTTAAGGAGGTGTTGTCTATATTAAATCAAACACAACTACTACATGTAGCCAAGCATCCAGTTGGAATTGATTCTCAACTTAGAGCTGTTGAGGAATTGGCCTCCCATGATGTGCCCGATGGTGTTAACATGGTGGGGATACATGGGATGGGAGGCATTGGTAAGACCACTCTGGCCAAAGCTTTATACAACAAAATCGCTTATCAATTTGAAGCTTGTTGCTTTCTTTCGAATGTTAGAGAAACCTTAGAGCAATTCAAAGACCTGGTTCAACTACAAGAAAAACTACTCAGTGAGATCTTAAAAGATAATGCTTGGAAGGTGGGCAACGTTCATAAAGGAAAGAATATCATTAGGGATCGGTTATGCTCAAAGAAAGTTCTTATCATTCTTGATGATGTGGATAAGGATGAACAATTAGACGCACTAGTTGGTGAACGTGATTGGTTCGGTCGAGGAAGTAAAATCATAGCAACAACAAGAGATCGACATTTACTAGAAAACCATTCATTTGATATAGTATATCCTATTCAGTTGTTGGATCCTAAGAAATCCCTTGAGCTTTTTAGCCTGCATGCTTTTAAGCAAAATCATCCCTCAAGTAATTATGTAGACCTTTCAAAATTTGCTGTAAGTTATTGCAAAGGTCTTCCATTGGCTCTTGTTATTTTGGGTTCTCTTCTCCATAAGAGAGAGCGAAAAATATGGAAAAGTAAATTACATGAACTTGAAAATTCCCTCGAACCAAGTGTTGAAGCTGTTTTTCAAATAGGTTTTAAGGAGCTTCACGAAAGAGTGAAGGAGATTTTTCTTGATATTTCTTGCTTTTTCGTGGGAGAGGATATTAACTACAGTAAGGATGTGTTAAAGGCATGTGATCTCAATCCAGACTATGGAATTATAATTCTTATGGATCTTTCCCTTGTTACTGTTGAAGATGGAAAGATACAAATGCATGATTTAATACAACAAATGGGTCAAACAATTGTTCGCCATGAATCTTTTGAGCCTGCAAAAAGGAGTAGGTTGTGGGAGGCAGAAGGAGCTATCAAGATATTGAAAGAGAAATCTGTGAGTGACTTTAGACAATGTTTTTATTTACTTATTGCCAAAGATATATACTCAGAAGCATTTAGAAACATGAAAAATCTTAGATTGCTTATCCTTCAAAGAGTAGCATACTTCCCTAGAAATATATTTGAGTATTTACCTAATTCGTTGAAGTGGATTGAGTGGTCTACATTTTATGTTAACCAGTCTTCGTCCATAAGTTTTTCTGAAACTCCCAACTTCTTTGCGACATTAAACCTTGAGAAATTATATCTTAGGGGATGCACGAGTTTGAAAGGAATGCGACCCTCTTTTAGAAAGTTTCCAAGCCACCTGAAGTTCAAATCTCTTAAAGTTCTGAATCTACGGGATTGTCTAAATCTTGAAGAAATTACTGACTTTTCAATGGCATCAAACCTTGAGATATTAGATCTCAATACTTGCTTCTCTTTAAGAATAATTCACGAGTCTATTGGGTCTCTTGATAAACTTATCACCTTACAACTCGATTTATGCCATAACCTAGAAAAGCTTCCTAGCAGCCTGAAGTTGAAGTCTCTTGATTCTTTGAGTTTCACTAATTGTTACAAGCTTGAACAACTTCCAGAATTTGATGAAAACATGAAATCTTTAAGGGTGATGAATTTGAACGGTACAGCCATAAGGGTGTTACCTTCATCAATTGGATATCTTATTGGGCTCGAGAATTTAAACCTTAATGATTGTGCAAACCTGACTGCCCTTCCAAATGAAATTCATTGGCTAAAAAGTCTCGAGGAACTTCATCTTCGCGGGTGTTCTAAACTCGACATGTTTCCCCCGAGATCAAGCTTAAATTTTTCCCAAGAAAGCTCATATTTCAAGCTGACGGTATTGGATCTCAAAAATTGTAATATATCAAATTCTGATTTCCTCGAAACATTATCTAATGTCTGCACTTCCTTGGAGAAGCTAAATTTGTCAGGAAACAAATTCTCTTGTCTACCCTCTCTCCAAAATTTTAAGTCATTAAGGTTTCTTGAATTAAGGAATTGCAAGTTTCTTCAAAATATAATAAAGCTTCCCCATCATTTAGCTCGGGTGAATGCCAGTGGTAGCGAATTGTTGGCTATACGTCCTGATTGCATTGCTGATATGATATACTTGCACGCCAATGATCGACACCACATCAAAGTCTTGTTTCCCAACACAACATCAAAATTTGTATCGAAAAGATTTAAAAGTGCAAATGTTATTATGAGAACCCAAGGAGTCTATATGGTTGATAGACGGTTTTCATGCTATACATAA

>Cucsa.277260

ATGGCTGAATTTTTATGGACTTTTGCTGTTCAGGAAGTTTTGAAGAAGATTGTGAACTTTGGAGCAGAGCAAATTAGTTTGGCATGGGGTTTGGAGAAGGAGTTGTCCCACTTGAAAAAGTGGTTACTCAAAGCGCAAACAATCTTAGCAGACATTAACACAAAGAAATCACACCATCATTCTGTTGGGTTATGGGTGGAAGAACTTCATGATATTATCTATGAAGCTGATGATTTGTTAGATGAGATTGTTTATGAACAAATTCGACAAACTGTGGAGCAAACTGGTAAACTTAGAAAGGTACGTGATTCTATCTCACCATCCAAAAATTCCTTTTTGTTTGGTCTCAAGATGGCCAAGAAAATGAAGAAGATTACCAAAACTTTATACGAACATTACTGTGAGGCAAGTCCTTTAGGACTAGTTGGTGATGAATCCCCCACAGAATCAGAGGCTGCACTTAATCAGATTCGGGAGACAACCTCAATTCTTGACTTTGAAGTTGAAGGAAGGGAAGCTGAAGTCTTGGAGATACTAAAATTGGTGATTGACTCTACCGATGAAGATCATATCTCTGTGATATCCATTGTTGGAATGGGTGGTCTTGGAAAAACAACTTTGGCCAAGATGGTTTTCAATCATGATGCCATTAAAGGACATTTTGATAAAACTGTATGGGTTTGTGTGTCTAAACCATTTATTGTGATGAAAATTTTGGAAGCAATCTTTCAAGGTTTAACGAATACTAGTAGTGGTTTGAACTCCAGGGAGGCCTTGCTTAATCGACTCCGAGAGGAGATGCAAGGAAAAAAGTATTTTCTTGTGCTTGACGATGTTTGGGATAAAGAGAATTGCTTGTGGGACGAGCTTATTGGCAATTTGAAATATATTGCTGGAAAATCTGGAAATAGTATTATGGTGACCACAAGGAGTGTAGAAGTAGCGACCATGGTGAAGACAGTTCCCATTTATCATCTAAAAAAATTATCGGATGATCATTGTTGGGCGTTGTTAAAAAAAAGTGCAAATGCAAATCAGCTGCAGATGAATTCAAAGTTGGAGAATACGAAAAATATTTTGGTTAGAAAAATTGGTGGTGTACCACTCATTGCAAAAGTTTTAGGTGGGGCAGTAAAGTTTGAAGAAGGTGGGTCTGAGAGTTGGATGGCAAAAATTGAAAGCTTTGCGAGAAATATTTCAATAGAGGACAAAGATTTTGTTTTGTCCATATTAAAATTAAGTGTAGAGTCTCTCCCTCATTCTGCATTGAAGCAATGTTTTGCTTACTGCTCAAATTTTCCTCAAGATTATGAATTTGATAAAGATGAAGCAATCCAAATGTGGATAGCCGAAGGATTTATTCAACCCGAACAAGAAAGAGAAAACTTGACAATGGAGAACATAGGAGAAGAGTATCTTAACTTTTTATTGTCTCGCTCCTTATTTGAAGATGCCATTAAATATGATGGAAGAATTGTCACCTTTAAGATTCATGATCTAATGCATGATATTGCTTGTGCAATTTCAAATCATCATAAGATGGACTCAAATCCTATTAGTTGGAATGGAAAAAGTACAAGAAAGTTGCGCACATTAATTTGCGAGAATGAAGAAGCTTTTCATAAAATTCAGACTGACATTATTTGTTTGCGTGTGTTAGTCTTAAAATGGTTTGACACTAATACCTTGTCGACTATTATGGCGAAATTGATACATTTGAGATATCTTGATATTTCAAACTGTAATATAAACAAGCTTCTTCGAGATTCTATTTGTGCACTTTATAATTTACAAACGCTAAAACTTGGATATATTGAATGTGATCTGCCGAAGAATTTGAGGAACTTGGTTAATTTGAGACATTTAGAATTTAAGAAATTTTTTGATATGGGACAAATGCCTTCACATATGGGCAACATGATTCATCTTCAAACACTATCTGAGTTTGTAGTTGGACTTGAGAAGGGTTGTAAAATTGATGAGCTTGGACCGTTAAAAGACCTCAAAGGTACACTAACTCTTAAAAATCTACAAAATGTGCAAAATAAAGACGAGGCTATGGCTGCAAAATTGGTGGAAAAGAAGTATTTACGTCATCTAATCTTTCAATGGTTTCTAAATCTTTATGATAGAGGAGAATATGATGAAGATGATAACAAACAAGTGTTGGAAGGACTTCAGCCACACAAAAACGTACAGTCATTGGACATTAGAGGCTTCCAAGGAAGAGTTTTGAATAATAATATTTTTGTTGAAAATTTAGTTGAGATACGTTTGGTTGATTGTGGAAGATGTGAAGTGCTTCCTATGCTTGGACAGTTGCCCAACTTGAAGAAACTTGAGATTATTTCAATGAACAGTGTGAGAAGTATAGGCAGTGAGTTCTATGGAGTTGACTGTAACGACAGAAATTCTTCTGCTTTTCCTCAGCTGAACAAATTTCATATTTGTGGGTTGAAGAAGCTACAACAATGGGATGAAGCAACGGTTTTTGCATCAAATCGCTTTGGATGTCTAAAAGAACTTATTCTTTCTGGATGTCATCAATTGGCAAAATTGCCAAGTGGGTTAGAAGGGTGCTACTCCATTGAATATTTGGCCATCGATGGGTGTCCTAATTTAATGCTAAATGTGCAAAATTTGTACAACTTGTATCATTTAGACATTCGTGGGTTGAAAAGATTGCCAGATGAATTTGGTAAGCTCACTAACTTGAAAAAATTGAGAATTGGTGGATGTATGCAAAACTATGAATTTAGTCCCTTCATACATTTATCTTCTCAGCTTGTTGAACTTGAGTTGACTGATGATGGGTCAAGTGGTAGTGAAACAACCCAACTTCCCCAACAACTTCAGCATCTGACCAACTTGAAGGTTTTGAAGATTGCAGATTTTGATGACATTGAAGTTCTACCAGAATGGTTGGGAAACCTTACATGTTTGGCAACATTGGTTTTCCTCGAATGCAAAAATTTGAAAGAGTTACCTTCGAGAGAGGCCATACAACGATTAACCAAATTAGATGATTTGGTGATCGATGGATGTCCCAAACTACTACTAGGGGAAGGCGATCAGGAGAGGGCTAAACTTTCTCATCTCCCATCAAAATGTGGTTTGGGATTGAAGAGTGTTATGATAAACTTGTTGCCAAAAAAATCATTTTAA

>Cucsa.292710

ATGGTGGGGATACATGGGATGGGAGGCATTGGTAAGACAACTTTGGCCAAAGCTTTATACAACAAAATCACTTATCAATTTGAAGCTTGTTGCTTTCTTTCAAATGTTAGAGAAACTTCAGAGCAATTCAACGGCCTAGTTCAACTGCAAGAAAAATTACTCAATGAGATCTTCAAGGATAATAACTTGAAGGTTGACAATGTGGACAAAGGAATGAATATCATAAAGGATCGATTGTGCTCAAGGAAAGTTCTTATGGTTTTGGATGACGTGGATAAGGACGATCAACTAGATGCATTGGTAGGTGGACGTGATTGGTTCGGTCGAGGTAGCAAAATCATTGTGACAACAAGAGATAGACATTTACTCGAAACATATTCATTTGATAAAATACATCCTATTCAATTGTTGGATTGTGATAAATCTCTTGAGCTTTTTTGTTGGCATGCTTTTAAGCAAAGCCATCCATCAAGGAATTATTCGGAACTTCCAGAACTAGTACGTTATTGCAACGGTCTTCCTCTAGCTCTTGTTATTTTGGGCTCTCTCCTTTGTAAGAGAGATCAAATAATATGGAAAAGCAAATTAGATGAACTTAAAAACTTCCCCGAACCAGGTATTGAAGCTGTTTTCCAGATAAGTTTTAAGAGGCTTCCAGAAAACCCCCCAGTAAAGGAAATTTTCCTTGATATTTGTTGTTTTTTTGTGGGAGAGGATGTTAGCTATAGTAAGAATGTGTTAAAGGCATGTGATCCTTATCTAGAATCAAGAATTATAATTCTCATGGATCTTTCTCTTGTTACGGTTGAAGACGGCAAGATACAAATGCATGATTTAATTCGACAAATGGGTCAGATGATTGTACGTCGTAAATCTTTTAAGCCAGAAAAAAGGAGTAGGCTGTGGGTGGCAAAAGAAGCTGTCAAGATGTTGATAGAAAAATCAGGAACTCATAAAGTTAAAGCCATAAAGCTAGACTTGCGCAACAACGGTTCACTGATTGTTGAAGCAGAAGCATTTAGAAACATGGAAAATCTTAGATTGCTTATTCTTCAAAATGCAGCAAAATTGCCTACAAATATATTCAAGTATTTACCTAATATTAAGTGGATTGAGTACTCATCATCTAGTGTTCGATGGTATTTCCCTATAAGCTTTGTTGTGAATGGCGGGCTAGTTGGACTAGTCATAAATGGTGTATCCAACAAACATCCAGGGATTATATTTGAGGATTGCAAAATGTTGAAGCATGTTGATCTGAGTTATTGGCGGTTATTAGAGGAAACCCCTGACTTCTCTGCAGCATTAAACCTTGAAAAATTATATCTTTTAAGTTGCAAACGTTTAAAAATGATTCATGGATCTGTTGCTTCTCTTAGTAAGCTTGTTACCTTGGACCTCGAAGGCTGTGAAAATCTAGAAAAGCTTCCAAGTAGCTTCCTCATGTTAAAGTCTCTTGAAGTTTTGAATCTAAGTGGATGCATAAAGCTAAAAGAAATTCCTGACTTATCGGCATCGTCAAACCTTAAAGAACTACATCTTAGAGAATGCTATCACTTGAGAATAATTCACGACTCTGCAGTTGGACGCTTTCTTGATAAACTTGTTATCCTGGACCTTGAAGGATGTAAAATTCTTGAAAGGCTTCCAAGATACATCAGCAACTCAAAGTCTATTGAAGTTATGAATCTTGATTCATGCCGAAAGATCGAACAACTTTTTGACAACTATTTTGAAAAGTTTCCAAGCCACCTCAAGTTCGAATCTCTTAAAGTTTTGAATCTTAGTTATTGTCAAAATCTTAAGGAAATTACTGACTTTTCAATTGCATCAAACCTTGAGATATTTGATCTTAGGGGCTGCTTCTCTTTAAGAACGATTCACAAGTCTGTTGGATCTCTCGATCAACTTATTGCCTTAAAACTTGATTTTTGCCATCAACTTGAAGAGCTTCCTAGTTGCCTCAGATTGAAGTCTCTTGATTCTTTGAGTCTCACTAACTGTTATAAGATTGAACAACTTCCAGAATTTGATGAAAATATGAAGTCTTTGAGGGAGATGAATTTGAAAGGTACAGCCATAAGGAAGTTACCCACATCAATTAGATATCTTATTGGGCTTGAGAATTTGATCCTTAGTTATTGCACAAACCTGATTTCTCTTCCAAGTGAAATTCATTTGTTAAAGAGTCTTAAGGAACTTGATCTTCGAGAGTGTTCTAGACTCGACATGCTTCCCTCGGGATCAAGCTTAAACTTTCCCCAACGAAGCTTATGTTCAAACTTGACTATATTGGATCTACAAAATTGCAACATATCAAATTCAGATTTTTTGGAAAATTTATCTAATTTCTGCACTACCTTGAAGGAGCTAAATTTGTCCGGAAACAAATTCTGTTGTCTACCTTCTCTCAAAAATTTTACATCATTGAGGCTTCTTGAACTAAGAAATTGTAAGTTTCTTCGAAACATTGTGAAGATTCCACATTGTTTAAAACGAATGGATGCTAGTGGTTGCGAATTGTTGGTAATAAGCCCCGACTACATTGCCGATATGATGTTCAGAAATCAGCGAGATCCCGAAATTCTGCAACAATCAAACCACAGAAAGTTCAATAAGTTTTAG

>Cucsa.318890

TTAGTTTTCAACCATGAGTTGGTTAGACAACATTTTGATAAAACTGTTTGGGTTTGTGTCTCTGAACCATTTATTGTCAACAAGATTTTGCTAGATATTTTACAAAATCTAAAAGGCACCATTTCTAATGGAGGGGATAGTAAGGAAGTTTTACTTCGTGAACTCCAAAAGAAGATGCATGGCCAAAGATATTTTCTTGTGCTTGACGATGTTTGGAACGAAAATTCTTTTCTATGGGATGAGTTGAAATACTGTTTGCTCAAGATCACTGGAAACTCTAAAAATAGTATTGTTGTGACTACAAGGAGTGCTGAAGTTGCAAAAATCATGGGAACATGTTCTGGTCATCTTTTAAGTAAATTATCTGATGATCATTGTTGGTCCTTGTTTAAAGAAAGTGCAAATGCATATGGATTATCAATGACTTCAAACTTGGAGATCATTCAAAAAGAGTTAGTCAAAAAAATTGGTGGTATACCATTGGCTGCACGAGTTTTGGGAAGGGCAGTAAAATTTGAAGGAGATGTTGAGAGATGGGAGGAAATGTTGAAAAATGTGTTAAGCACTCCACTCAAAGAGGAAAATTTTATTTTGTCTATATTAAAATTAAGTGTGGATCGTCTACCGTCATCTGCATTAAAGCAATGTTTTTCATATTGTTCAATTTTTCCCAAGGATTTTGTGTTTGAAAAACAAGAACTAATTCACATGTGGATGGCACAAGGTTTTCTTCAACCACAAGAAGGAAGGAACATGACAATGGAAACTGTAGGAGACATATACTTCAAGATCTTGTTGTCACACTGCTTATTTGAAGATGCCCATGAAACAAAGACAGAGGAATATGAGATACCTGATCTGCTTGAATTTGAAACAAGGCCAGAAGAATATAAGATGCATGATCTTGTACATGATATTGCGATAGAAATTTCAAGAGATCAAAATTTGCAACTAAATCCTAGCAATATATCAAAGAAGGAACTTCAAAAGGAGATTAAAAAGGTTGCATGCAAGTTACGCATGGTTGATTTCATTCGACGGATTCCTTGCAATATAGGCCAACTAACATTTTTTGATGTTGAGATAAGGAACTTTGTTTGTTTGCGAGTTTTAAAGCTATCAACGCTGCCTAGTGATAAGTTACCGAAGTCAATTGGTCAATTGAAACACTTGAGATATCTAGAAATTGCATGTTATTTAGGTAGATTAAAATTTCCAGAGTCTATTGTTTCTCTTCATAATTTGCAAACACTAAAGTTTCTATACTCATACGTTGAAAAATTTCCGATGAACTTTACAAATTTGACATTGTCTCATTTTGTGATCGGGTTTGAAGAAGGTTGTAAAATTACTGAATTGGGTCCATTGAAAAACTTGCAAGGTTGTTTGAGTCTTTTGTGTTTGGAGAAAGTTGAAAGCAAAGAGGAAGCCAATGGAACAAACTTGGCAGAAAAGGAGAAGTTAAAAGATCTACACTTAAGTTGGTCCAATGAAAGAAAAGATAACAACAATTACAATGATTTGGAAGTGTTGGAAGGACTTCAACCAAACCAAAATCTACAATCATTAGGAATCTACAACTTTGCAGAAAGACGTTTGCCTAACAAGATTTTTGTTGAAAATTTAAGCGTGATAGGTTTGTATGGTTGTAATAATTGTGAAAAGCTTCCAATGCTTGGACAATTAAACAACCTAAAGAAACTTGAGATTTACAGCTTCCATGGCGTCCAAATTATAGACAACGAGTTCTATGGTAATGATCTAAACCAAAGAAGGTTCTTCCCAAAGCTTGAGATATTTGTAATGTGTGATATGATCAACTTAGAGCAATGGAAAGAAGTAATGACAAATGATGCATCATCAAATGTTACAATCTTTTCCAATCTTAAATGCTTGGAAATACGTGGATGTCCCAAATTAACAAAACTTCCAAATGGACTACACTTTTGTAGCTCCATTCGACGTCACCTTCTTTCCCTTAAAAAAATTACTTTAGTCGAGGATGAGTTGAGCAACAATAGTGTAACACAAATTTCTGAACAACTTCAACACCTCACTGCCTTGGAATTTCTGTCCATTGAAAATTTTGGAGGCATTGAAGCTTTGCCAGAATGGTTAGGAAACTTTGTATGTTTGCAAACACTCAGTCTTTATAACTGCAAAAATTTGAAAAAACTGCCTTCTACAAAAGCAATGCTACGTCTCACTAAATTAAATCAATTGTATGCTTGCAAATGTCCGATGCTACTACTCGAAGAAGGTGATCCAGAGCGAGCAAAACTTTCCCACTTTCCAAACATGTTGGTTCAGCGCAACGGTTATCTGAAGTGTATTTAG

>Cucsa.326910

CAAAATACCCATTTTACACCAACTTGATTACCTGTTCCACTACAAGCAAAATATTAAGGAAGTTGAAAAGAAAGTTGAAGCTCTTGGGACTGCAAAAGGGAATGCAGTTTTTGATGGAGTTTCTAAATGGTTGACAATTGTAAAGGATGTATTGGAGATAGCACAACAAAATGAAAACCCCTCATGCTTTAATTTTGTTGAACGATATCAATTATCCAGAAAAGCAAAAAAGAGGGTGGAAAATATTATTGAACTCATAAATGAAGGAAATGGATTTAACAAAGATAATGTTGGTTATCCCGTACCTTCTCCCGATACTAATTCTCCCACTCTCCCTACCGATTATCAAATTATCGCGTCAAGAACTTCAATAGTAGAAGAAATAAAAGAGGCACTTGCAAACCCTAATGTCGACACAGTTGGAGTATGTGGTATGGGTGGTGTTGGAAAAACTGCTTTATTGAATGAAGTTAAGAAGTTAGTGTTGGAAAAGAATTTGTTTGATCGAGTGATTCAAGTGGAAGTTGGTGAATCCAAAAGTGTATTCAATATTCAAGAACAAATTAAAGATGAGTTAAATATGGAATTGAATATAGAATGTGAGGAGGTAAGAGCATGTCGCCTACGAACTCATATTGCCGAGAGGAAAGAGAATATGTTGTTTATGTTGGATGATATATGGAAGGAACACGATGTTGAAAAAGAGTTTGGAATTCCTTTTAAACATGTATTAAACAATGAAATGAACACAGAAAAGACATTTGAGGTGAATTCTCTTACCAACGAAGAGTCTAGGAACTTTTTCGTGACAATAGTTGGTGAGTCTTCGTGTGTTGAAGATGGACATAATATACAACAAATAGCAGAGGATGTGGTAAAAGAATGTGGAGGGTTACCACTTGCACTTAAAATTCTAGGAAAAGCATTGAAGGGAAAAAGAGTACAGATATGGAAGGATGCGTTAAAGTCATTGAAAAATCCTGTTACAGTGACTATTTCAGGAGTGAGTGAGCAATTGTATTCTTGTCTCCAATTCAGTTACGATTCAACAGAAGATGAAGCAGAGCAAGTATTACTTCTATGTAGTGTATTTCCAGATGATTACAAGATTGAAGTGAAGGATTTGCAGATGTATGCAATGGGTATGGGATTGGTAAAGCACATAAATACTTGGGAAGATGCAGGGAATAGAGTAATCAAGTTGGTTGATGATCTTAAATCTTGCTATTTGCTTCAAGATGAGCAGTCCAAGAAAGGATCAGATGATTGTGTTCAAATGCATGATGTGGTCCACGATTTTGCGAAATACGTTGCATCAAAGAAAGATAAGATGACGTCCTTGACGTATAGGAGTGGGCAAAGGCTGGAATACTGGCAAGAAGAAGATGATGATATGCATGAATCTTATAAAGCAATTTATGCAGATTGTGCGAAATATTGTGTATACCTTCCCCCAAAAGTTGGAGTTTCCGAACCTTCAATTGGTAATAAAATTCGAATTCCTACTGCATTTTTTGAAAGAATGAAAGCGCTTCGAGTTTTGTCTGTGGAAACTATGAGTATAAGTTTTGAACCATCAAGTTGGGCATCAATTAACAACCTTGAAGCATTATATGAATTGATAAAACTAAAGGTGCTACACGTGTTGAAATGCGACGACTTTAATCCGTCGGAGTTTCCTCCAAACATCATTGAAAGTATGACACAACTGGAAGAGTTGAAATTTGATGGCTTCAAAATGAATGAACTTTCCGAATTGAACCGTCTGACACGCCTTTTCAGTTTAGAGTTAAGGATTCAGAATGTTGAGATCTTGTTAAACGAACTGAGTGTAGAAAAAGCAGAAAAGTTGGAAGAATTTAGTTTTTGTGTTGATTCGGTGGGTTTTACAAACCTTCTTTGCAATAATAACCATACTGTTCCATATGGAAATTATAATTGGTATCCCCGTTTGAAGGAACTTCAAATCTACATTCACAATAATCAAAATCAATACTTAGACATGCCACGTGGGATTGAAAATAACCCATGCATCTTGATCTTCAGTTGCAACAAATTAACATACGTATTTCCATCACATATGCTTACGTTACTTGTTTTCTTAAATACATTAGAAGTACATCATTGTAAGTTAGTAGAAAGGATATTTGAAATTGAAGAATGGAGTGGTAGTGGTGGTGCAGGTGATGTAAACCAAGTACTAGTCCCCTTCACGATCTTACACCTATCTTTTCTACCAAACTTAAAGCATGTTTGGAATACCGATCCAAATCCCACAACTTCAGACCTTCTTCCCCAATGTAAAGAAAGTGGAGATTACAAAGTGTCCGACGCTTAA

>Cucsa.328080

ATGGCAGAATCCATTCTGTGCAGCCTTGCAGGAAGCATTATTACCAAATTGGGTTCTTTCGCACTTCAAGACCTTGGATTGTTGTGGGGTTTCCATGATGAACTTGACAAACTGAAAGGCACTGTTTCCGCCCTCGAAGCCGTACTTCTCGACGCAGAAGAGAAGCAGTCCAAAAGTCGTGCAGTGAAGGACTGGATTTTAAAGCTTAAAGATACTTTCTACGACATCGACGATTTGTTGGACGTGTTCTCCTATGAATCTTTGAAAAGACAAGTTATGACCAAACATAGAACTAACAACACCAAAAAAGTACGCATTTTCTTCTCAAAATCTAATCAAATTGCATTTCGTTTGAAAATGAGTCAAAAAATCAAAAGGGTCCGAGAGAAATTAGATGCAATTGCTATGGATAAAACTCAATTCAATCTTTATGAGAATACTAGGGAAATACAAGATGATGAATCGACGAAACGACTGGAGACTACCTCTTTCATACGTGAAGGAGAAATAATTGGTCGGGATGATGACAAAAAAAGTATTATACATTATCTATTGGATACCAACATCCACGAAGATAGTGTTGCAGTGATTGCGATTATTGGAATGGGAGGATTAGGAAAGACTGCTCTTGTTCAATCTATTTATGGTGACGAGAAGGTAAAGAAACATTTTGAGTTGACAATGTGGGTGTGTATTTCTGAAGAATTTGATGTCAAAGTAATTATTGAAAAAATTATAGAGTCTCTCACAAAAAAGAAACGTGAGCCTGACCTTCAGCTTGATACGTTGCAAAGTATGGTCCGAGAGAAAATTGATGGAAAAAGATACTTGCTTGTCATGGATGATGTGTGGAATGTTAATCGTGCAAAATGGATAAGTCTAAAAAGGTATCTCATGGGTGGAGCTAAGGGAAGTAGGATTTTGATCACAACCCGTACTCATCAAGTTGCACAGACTTTTGAAACAATTTTATCCCATCATTTAAAAGAACTAGATGAAGAAAAATCTTGGAAATTGTTTAGAAAAATGGCATTTTCCAACGAATCAGAGGTGCTTGAGAATTCAAAGTTGGTTGTAATTGGTAAGGAGATTGTTACAAAGTTGAAAGGTTCTCCTCTTGCAATAAGAGTAATTGGGAGTTATCTCTATTCAAAAAAGTCAGAAAAGGATTGGTTGTCATTCAAGGACCATGAACTCGACACAATCATGCAACAAGAAAATGAGATACAATCGATACTAAAGATCAGTTTTAACCACCTCTCATCTAGTTTGAAGCATTGTTTCACATATTGTGCATTATTCTCCAAAGATTATCACTATGAAATTCGAAAAAATGATTTGATAAAACAATGGATGGCACAAGGCTTCATTCAACCACATAATAAGAAGGCAATGGAAGATGTTGGTGATGATTATTTCGAAGAACTACTGGGGAGATCATTTTTTCAAGACATAAGAAAAAACAAATGGGGAGAGATCAAGAAGTTCAAGATGCACGACATCATACACGATCTTGCATGTTCTGTTGTAGAAAATGACTGTGTACTTGCTAATGATGACACTAAGTCCATTGACAAAAGGACTCGACATGTGTCAATTTCGGCCTTCAACTCAATGACAAGATGGAAACTCATTACAAAATCATTAATAGAGGCAAAGAATTTGAGAACATTGAATTATGCTCGTCGTCATCACATCGATCTCTCTAATCATTTGCGGTTACGAACATTGAATTTGGAGTTTCATTTTGTTCCCAAGTGTATTGGTAAGATGAAACATTTGAGATATATTAATATTACTTACTGTTATATTGATTTCCTTCCCAAGGCAGTTACAAAACTGTACCATTTGGAAACACTCATCATTCGTGGATGTCTCGAGCTAAGAGAATTGTCAAGTGATATTAAGAATCTTATCAATCTTAGACATCTAGATATTAAGGATTTTAAACATGTTTGGAGTTATATGCCAAAAGGAATGGGTTCAATGACTACCCTTCAAACAATGAATTTGTTTATATTGGGAGAGAATAAAGGTGGTGAGTTAAGTGAACTCAATGGATTGGTCAACTTGAGAGGATCATTAAGTATTCAACAATTGCAGTTCTGCAAACCCATTGGCTTAGAAAATGTTAAATACCTTGAAGAAAAATCCAGAATTCAAAAGTTGGAATTACATTGGAAGACCTATCAAAGGGAATCTAAAATTGATGATGAAGATGAGAGAGTTTTGGAGAGCTTGAAACCACATTCAAATCTTCAAAAAATACGCATAGAAGGATATAGAGGATTGAAGTTATGTAACTGGTTCTCATTTGATTCTATTGTGAATTTGGTCTTTATAAAGCTTTTCAACTGTGAAAAATTGCAACAGCTTCCTCGATTTGATCGATTTCCTTTTCTCAAACATCTTCATCTGGAAGATTTACCGAGTATCGAGTATATTGCTATTAACAATTATGTTTCTTCATCAATGACTACTTTCTTTCCATCCCTTGAGAATCTAAGCATCATTAAGTTGCCTAACTTGAAAGAATGGTGGAAGGGGGAAAGCATTGATCAAAATACTTCATTTCCAACAATTTTACGTCATCTTTCTCAACTAAAGATTCATTATTGTAGACAATTGGCTTCTATTCCACAACATGGACCTTTGCAATCATTGGACATACGTGATATCAGTTTGCAACTTTTTGAGTTGGTAATCAAAATGACCGCTACGAACATTATTTTTCTACCAAATGACTTATTCTCCAATGTGACACATCTCCAGTCTCTTGTCATAGGACGTTGCTTCAATTTAAAAATGTCTTTTGATGATGATAATGTAAGATGGAAAGAACTCGGAAGTCTTCGCACACTTCGACTTTGTTTCATCCCCAAATTAGAGTATTTGCCTAAGGGTTTCCAATATTTGAAAGCACTCGAACATTTGGAACTACTTTGGTGTGAAAATTTGGCATGTATTTTAGGGATTGAGCATCTCACTTCACTATCACGATTGGAAATTTCAAATTGTCCTAATTTAACTTCTTTGCCGGAAGGGATGACTCAACTTATTTCATTAACATGTTTGATAATCGATGATTGTCCCAATTTAAGTACCTTGCCAGAAGGGCTTCATCACCTCCTTAATACCCCGAGGTACGCACCACTAATTTTTTCCCACTAA

>Cucsa.337180

ATGGCTTGTTGCATCTATGAGCAAGCAGAAAATATCTTGATAGAGCTAAAAAAATTCCCAATGTACCTGAGACGAATGCAGTATACCATGTTGAGCCTTAAAACAATTCTTAAGGATGCTGAAAAGGAAGAATATCGTCATTGTCTAAATGATTGGCTACAGAAGCTTCAAAGTGTATTTTTACAAATTGAGGAATTGCTATATGAATCCAATAGGGAAGTCAAAAAACAAGAGGCTACTGGAAAATGGGTATTTCTTCCTTCCTTTAACTTCAGTCAAATTGATCAAACTAAAAAAATGATGAAACTATGCGACGATTTGGATGAAATTGCATCCCATATGTATGGCTTCAATCTAACAAACATGGAGACAACACACTCCTTTCTTAGTGCTACTGAAGTTTCGACAAGACTCATGAAACCAAGTTGGCAATTGCTTTACTCGTTGACTAATGCTCCCAAGGTTTTCCAAGACAAACGATATCATAACTTTCTGGATCATTTCAAAAAATCTACTCACGGGCTCTTCCACATAGTTGGAGAACCAGGTATAGGTAAGACCACACTTGCCAAATTCTTTTACAACAATCTGGTGAACACGTTTCCATCAAGATTGTGGATTTGTGTGAAAGAGGAATTTGATCCACAGAGATTGATAAAAGAGATGCTCAGTTTTTCACATTGCCAAGTAACATGTGATAACTTGACTGAGAAACAATTGTGCTTTGCAGTTCAACAATTTCTGAGGGATAAAAAATTTCTGATTGTTTTTCAAGATATTTCAATCAAAAACCTTGGTAATTGCTCCATATTTAAAAGTTTATTGGGGATGGGAAACCGTGGCAGCAAAATCATAGTGACCACTCAGAATGAGAAAATAGCTGATGCTGTCGGACTAAAAAAACTCTACAAGAACGAGAGCCAGGTAGTTCCATCCCCAGAAGCCACAAAACCTTCAGATGTTAACAAGGATAATATGAAACATCAAACAATTTTCAAAGTTGAGAGGTTGTCAAAGGAAAATTCATTGTCTTTGTTCAAAGTTCATGCTTTCACAGAAACACAGGAAGCACAAATCCCAAATCTCACAAAAATACAAGAAGTAATTGAGCAGAAATGTCATGGGGTTCCTTTGGCAATAAAGTGCCTGGGGGGTCTGCTATCAAAAACTAGTATAGCTGAGTGGAACGGTGTCATCGATAAGTTATGGGAACATGAGGAAGAGGAGGATGGGAATAAGAGTATTTTACCTACACTTAGATTATGCTATGATCAAATGCCTTCACACCTACAACGTTGTTTTCTTTATTGTTCCCAATTAAAAAAAGATCGCATATTGTCTTCCAATGATGTGATTCAATTATGGATTGCAAGCGACCTCCTACCCAAAGAGAATTACTTATCTTTGGAAAAAATAGGTGAGAATTATTTCAAGGAACTATGCTCAAGATGTTTCCTACAAGAACTAGAGGAATATGGTTTTGGCTATTGGTTTAAATTGCACCCTCTTATTGAAAAACTTGCACGTCTACTCACACAAAAACAGGTATTCGAAGTCACAAAAACCCAATCTATAGCCTTCACAATAAGAGATAAGGTGCCCCCTAGTGCATTCCTAGCAAATGCATGCATCGACAAGTTCAAATACTTAAGACTATTGCATTTAGGCAATGCAAATCTACAGGGAATTCCAAGTGCTGTAGAAAATCTGGTACAGCTCAGATACCTAGACTTGCAAGGGAATAAGAAAATCAAGCGGCTACCAAATTCAATCTTCAAGCTAAAAAATTTACAAACCTTGATTCTTGCATCCTGTTCCGCACTTAAAGAACTGCCCAATGATATTAGGCAATTGACCAACCTGAGATACCTCTGGGTAACAGCAAACAACCTTCGTCTGCACAAAAATGGAGTTGGAACCATGACTTCTCTTCGATTTCTCGCAATTGGAGGTTGCAAAAAACTTACACTGACGTTGAAAGGAGTGGAGTTCAGGCTTCAGAGGTTCACAATCAGAGAGCTTCCAATAGTGAAAAAATTGCCGGAATGGACTCAAAGATTCACCGAAACCCTAAGAGTTTTGGAAATCATCGATTGTCCCATCGAATGGAATGATGATGTGTTAAAATCATACAAATCACTTGAACGGTTTTCAATTCATGGAGCTGTGAGGACCAAAAACCAGATCGGGGGGTACAACATCGATTATCGTAATTTCGTTAGGAGTAGGAAAGTCAAGAAGGAAGTGAAGACATGTGTCTACTACTAA

>Cucsa.337190

ATGGCTTATTGCATCTATTACCGAGCTGAAAACATCTTGAGTGAACTGAAAAACCTCCCAAACTACCCAAGAAGAATTGAGTATACCATGTTGAGTCTTAAATCAATTCTTATGGATGCGGAAGAGAAGCAAGAACAGAGTCGTGGTCTACAGAATTGGCTAGAGGAGCTTCAAAATGTATTTTCCCAAATTGAAGGCTTCATAGATGAACACAAAGAGGAAGCCTACGAGGGTATTGGTAAACAGGTACTTGCTCCTTTCTCGTGCTCCAGTAATCAAATAGCACGTACTTGGAAAATGGAGAAACTATTCGACCATTTGAATGAAGTTGCGGCAAAAATGTATGAATTTAATCTTACAGAAAGGCACACTGGTGCCATAAAAACGGAGACAACAAACTCTTTCCTTACTGCTACTGAAGTTTCAACAAGACTCATGAAACCAAGCTGGAAAGTACTTTACCCCTTAACTAATGCTCCGAAGTTTTATCAGGATGAGCGGTACCGTAAGATTCTGAATGATTTCAAAAACCCTACTCTAGGGTTCTTCCACATAGTTGGAGAAGCAGGTATAGGTAAGAGCACACTTGCCAAATTCATTTACAATGATCCAGAAGTAGAAGGAATGTTTCCATCAAGATTGTGGGTTTGTGTGAAAGAGGAATTTGATACACAGAGATTGATGAAAGAGATACTCAACTTTTCATATTCTCCAGCAACTTGTGACAATTTGACTACAAAATTGTGCCCCACAGATCAATATCTGAGAGAGAGAACTTTTCTGCTTGTTTTTCAAGACCTTTCAATCAAGAACCTAGATAATTGTTCCCTGTTTACAAGTTTATTGATGATGGGAAAGCCTGGTAGCAAAATCATAGTGACCACTCAGAATGAGGAAATTGCAAATGCTATAGAACTAACAATGATTTACAAGGTTGGGCAACAATCAGAGCAAAATCGGAGCCAGACAGCCCTAGACACGGTTACTAAAGAGACTGCAAATGTTAACAACGCCGACCAGTTCGTTCAAGCTAACCCTTTGGGTAAGATAGATCAATCTATCCCTTCTCAAACAATATTCAAAGTTAAGAGGCTGTCAGAAAAAGATTCCCTTTCTTTATTCAAAGATTATGCTTCTACATATGAAGGTAATGAAAAAGATATAATGAAAACTCTGAAGAAATGTAATGGAATACCATTGGCAATAAAGTGTCTGGGGAGCATGTTATCTCTAGGTCCTCCAGCAACTAAATGGATGGAGGATAATGAGCGACAAAAGGGAGATAATGAGTCTTCTAGTACATTTAGTATACTTAAACTATGCTACAATGAGATGCCCTCACACCTGAAGCGTTGTTTTCTTTATTGTTCTCAATTACCAAACGATAGCATACTGTCCTCAAATGATGTCATTCAGTTATGGATGGCAAATGGACTTCTCCGTTCACGCCAAGAGAATTACTTATCCTTGGAAGACATAGGTGAGATTTATTTCAAAGAACTATGCTCAAGATGTTTCCTTCAAGATGTTGAGGAATATGGTCTTGGCTATTGGTTTAAAATGCACCCTCTCATTCGGGAACTTGCACGCCTCGTGCAAAAACGAACTAAGGACTTGATAAGCATTAAACCAGTCACCAATGTCACATCTATAGCCTTCCCAGTAAGAGATGAGGTGCCATCTAGTTCATTTCTAGCTGAAAAATGCATCTCAAAGTTCCAACACTTAAGATTATTGTATTTAGGCCACACAGATCTACAGGAAATTCCAAATACTATAGAAACACTGAATCACCTAACATACCTCGACTTGCAGGGGAATAAGAACATCAAGCGGTTACCAAATGCAATCTGTAATCTACAACATTTGCAGACCTTGATTCTTGCATCTTGTTCTGCACTTGAAGAATTGCCAAAAGATATATGCAAGTTGAGCAACCTCAGATACCTGTGGGTAACATCAAACAAGCTTCGTTTGCACAAAAATGGAGTAGGAACCATGACTTCTCTAAGATTCCTCGCAATTGGAGGATGTGACAAACTTCAAGATCTATTCGAACGGCCATCATGCCTTGTACGCCTTGAAACCCTAATGATTTACGATTGTAACTCTTTGCAATTGTTGCCAAACGAGATGGGGTCTCTAATATCGTTACAGAATTTGGTGATATGGAGTTGCAAACAACTTACACTGAAGGGCTTAGAGAAAGTCGATTTCAGCCTCCAAAGATTCACAATCAGAGAGCTTCCAGAAGTGAATAAATTGCCTGAATGGCTTCAAAGGTCGACAGAAACCCTAAGAGTCCTGGAAATCATCGATTGTCCCATCAAAGTGGAGGAAGAGGGAATCAAAATACATCACTGGGAATTTGGTACGGCGTAG

>Cucsa.338110

ATGGCCAAGAAAATCAAGAACATTACCGATACTTTAAATCAACATTATTGTGCGGCAAGTGCTTTTGGGCTAGTTGGTGTGGAAACTGTCACAGAAATAGAGCTTGCGCTCAATCAGATTCGAGAGACAACCTCAATTCTTGACTTCCAAGTCGAAGGAAGGGAGGCTGAAGTTTTGGAGCTACTTAAATTGGCGATTGACTCTACCAATGAACATCATATGTCTGTGATATCCATCGTTGGAATGGGTGGTCTTGGCAAAACAACTTTGGCCAAGATGATCTTCAATCATCGTGAAATTGAAGGACATTTTGATAAAACTATATGGGTTTGTGTGTCAAAACCATTTATTGTCACAAAAATTTTGGAAAAAATCTTTCAGGGTTTAACAAAAACTTGTAGTGGGTTGGAATCCAATAAGGAGGCCTTGCTTGGGAGGCTGCGAAAGGAGATGCAAGACAAGAATTATTTTCTTGTGCTTGATGATGTTTGGGATAATGAGAAACACTTGTGGGACGAGCTTAGAGGCTGTTTGAAACATATTGCTGGAAAACCTGGAAATACTATTGTGATGACCACAAGGAATGAAGAAGTAGCGACGATGGTGGAGCCAATTTCTATTTATCGTCTAAAAAAGTTATCCAATGATCAATGTTGGGCGTTGTTTAAAGAAAGTGCAAATGCAAATCAGTTGCCAATGAATTCGAAGTTGGAGATTATGAAAAAGGAGCTGGTTAAAAAAATGGGTGGTGTACCACTCGTGGCAAAAGTTTTAGGAGGTGCAGTCAAGTTTGAAGAAACTGAACTTGAAGAGGAAGATCATGAGATCAGTTGGATGACAAAAGTTGAAAGCATTGTAAGGAACATTTCATTAGAGGACAAAGATTTTGTTTTGTCCATATTAAAATTAAGTGTGGATTCTTTACCAAATCCCGTGTTAAAGCAATGTGTTGCCTATTGCTCAAATTTTTCCCAAGATTATGACTTTCAGAAAGATGACCTAATTAAAATGTGGATAGCACAAGGATTTATCCAACCCGGACAAGGAAGAGATAAGAACTTGCTAATGGAGGATATTGGAGAACAATACTTCAACTTCTTATTGTCTCGTTCCATATTTCAAGATGTCACTAGGGATGCGAATAAGAGAATTGTTGGGTTTAAGATGCATGATCTAATGCATGATATTGCTTGTGCAATTTCGAGTCATCAAAATGTAGAATCAAATCCAAATAATTTGAGTGGAAAAAGTGTAAGAAAGTTACGCACGTTGATTTGCAATGATGAAGTGATTAATTATTTGAATCAGAAAGACATTGTTTGTTTACGTGTTTTAAAGGTTATTTTTCAATCGCATACGGATTTGTGGATTCCAATAGACAAGTTGATTCATTTGAGATATCTTGATATTTCAGAATGTTCTATAAACAAGCTTCTTCTTGAATCCCTTTCTCTTCTTTATAATCTACAAACGCTAAAGCTTGGACAAAGTGGTCTACCGAAGAATTTGAGAAAATTGGTTAACTTAAGACATTTAGAATTTAAAATGTTTGGTGATACAGCAATGCCTTCAGATATGGGCAACTTGATTCATCTTCAATCATTGTCTGGATTTTTAGTTGGGTTCGAGAAGGGTTGTAAAATAGAAGAGCTTGGACCGTTGAAAAACCTGAAAGGTAAACTAACTCTTACAAATCTCTGGAGAGTGCAAAATAAAGATGAAGCTATGGCTGCAAAATTGGTGGAAAAGAAGAACTTACGTCATCTAAACCTATGGTTTTTCGAAACCGATAAGAGAGGAGAAGATGATGAAGATGGTATAGTACAAGTGTTGGAAGGACTTCAACCACACAAAAACCTACAATCATTGGAAATCCTTGGTTTTCGAGGAAAAGTTTTGCCTACTGGTATTTTTGTTGAAAATTTAGTAAAGATACGTTTGGGTCATTTTGAAAGATGTGAAGTGCTTCCCATGCTTGGACAGTTGCCCAATTTAAAGGAACTTGAGATTATGTACATGGAAAGTGTGAGAAGTATAGGGAATGAGTTCTATGGAGTTGACTCCAGCCACCAAAATTCTGTTGCTTTTCCACAGTTAAAGAAAGTCAGCATTTATGAGATGATGAACCTAGAGCAATGGGATGAAGCAACGGTGGTTCTTGCATCAAATCTCTTTGGATGTCTAAAAGAAGTTAGGATTAGGAGATGTAATCCATTGGCAAAGTTGCCAAGTGGGTTGGAAGGTTGCCATTCCCTTGAATATTTGAGCATCCGTGGTTGTTTTAATTTGATGCTAAATGTGCAAAATTTGCACAAATTATACCATTTAGAGATTGATGGGTTGAAAAGATTGCCAAAGGGAATGGACGGACTCACTCGCTTGAAAGAGTTGAAAATTGGAGGATGCATGCAAAATTATGAGTTTAGTTCCGTCATACACTTGGCTTCTCAGCTTGTTGAACTTGAGTTGTCTGGCCGTTATGGGTCAGTTGACACCCAACTTCCCCAACAACTTCAACACCTCACTAACTTGCAAGTATTAAAGATTACACAGTTTGATTGCATTGAAGCTCTGCCAGAATGGATTGGAAACCTCATCTCTTTGAAAACATTGAAATGCTCCTATTGCTTTAAGTTGAAAGAATTACCTTCGAGAGAGGCCATATTACGCCTAACCAAATTAGAAAATTTGGACATTTTTGAATGTCCAAAGCTACTAGTTGGGGAAGGTGACCAGGAGAGGGCTAAGCTTTCCCATCTTCCATCAAAATGTGTTCATAAATCTGAGTAA

>Cucsa.338190

ATGGTTGGACTTCTCGACAGTGTGGCCGGAAATCTGCTCGGAAGGATAATCGAAGCCGCCGACCGACTAGAGTTTCGTGCTATCCAAAGCGAATTGAAAAACCTCGAAACAGATGTGTTGAATCTTAAGGCCAGACTCCGAGACGCCGAGGAGAAGCAGGCTAGTAATTGTGAACTCAATGAACTGCTTAAAAACCTCAAAAATGTGTTTTCAAGGGCAGACATTGCAATTGAGGAATTGGAATGCGATTATTTGAAGTGGAGAGTGCAGAATCGAAAGAACGACGTTGACGATAAGGGATGCCAGTTCTCTTCTTGTTTCTCCTCCAATTTCCTCATTTCTCCATTTAATACCGGCAGTAAATTCCAGGAAGATCTTAAAATAATTACCTCCGAATTACGTTCGATTGAGAAAGCCATGTCTAAATTCTCTCTGGTTGAAGATGAAGATGAATATATAAAAAAATTGAAGGGTGAAATGACTTTGCGGACCTCCATTACTGGTTCGCATGCTTTCGCTAGGCTTCTGCGCTTGAGGAGAGAGGCGATTCTCTCTAATGTAGATTCCATTTTTGGTAGAGATAAAATACAAGAGAGTATCATTAAGGAACTTGTGAATGATGAACAAAAATCTCCCCGTATTCTTTCAATCCAAGGAGATGGAGGGATGGGAAAGACGGCTCTGGCCAAGTTAGTCTACAATGCAGACGAAGTGTTTGATCATTTTGACAAGAGAATGTGGGTATGCGTTTCTGAAGATTTTGATATCCGGAGAATCTTAAGGGAGGTTCTGATGTCTGCAACTGGAGAAAATGTTACCACTGTTGCCTTAACCGAAAGTCGTTTACGAATCCGGCTCCAGCGGTACTTTTTTGGCAAAAAAATCTTGCTTGTTTTGGATGATTTTGGGAATTTGGATCCCGAAAGAGTATCAGAACTGAAAAAAATCGTGAAGATGGGTGTTGGTGGCAGCAAGATAATGATAACCACTCGCAGCGATGAAACTCTAAATGTTGCTACGACACACAAGATTGACAAACTCGACGAGACGATATCTATGCAAATATTCGAAGATACATATGGAAGCGAAGGGCTTAGCGAAGGGCTTAGAGACGATTTGTATCTCAAAAACCTTGTGGCAGAATGTGGAGGAGCTCCTTTGGCAATCAAATGTTTGGCTGGACTGCTCTCTTCAAAACCGAGCGATGGTGCTAAGAGTCCAAATGTCAAGGACTTGAGTGAGAAATGGAAACAGGAAGAGGCAAACAACGGTGGTGGCGTTTTATGTGCACTAAGACTGAGTTATGATCTAATGCCATCTTATTTGAAACCTTGTTTTCTTTGCTTTTCAGTGTTGCCGAAAGATAATGTGTTCTTCTCATTTGAGCTAATCCAGTTATGGATGGCACAAGGAATCCTTCCTTCGGGTACCAAAGATAATCCTGAAGAAGTTGGGGAGAAATATTTCAAGGAATTTCGGGATCGCCGTTTACTCGTTGATGTTGAGGAGCACACTCTTGGATATTGGTTCAAAATCCATAGCCTTGTACATGATCTTGCAGTCCAAAAGGCTACGGAACAAAAGAACCTCGGAAATTTTCATATGCTTTCATTTGTCGATTGCGACAGCATCCCTTCGTCGACAAACTATGATAACACTCGTTTTATTTCCATTCCCGTGGTAGGAGGTGCGGGACCAAATATCAATAGTGACCTTTTCAAATGCATCACCCAGTTCAGGCAGCTAAGGTTTTTGTACTTGTGCAACTCTTCTCTGGAAGAAATTCCAACCTCCATCGACACGCTGAAACATTTGAGGTGTTTAGATTTGCGAGGGAGTCAACGTCTGAAGAGGTTGCCAGAATCAATTTGCAAACTACAGAGCCTACAGACTTTGGTTCTTGCATTCTGCTCAGAGCTTGAAGAGCTTCCCAGAAACATAAAGAACTTGATCAGCCTCAGATTCTTATGGATACAAACAAAGCAAGCCCGCTTGGAAAAAGATGAAATAGGAAGCTTAACATCCCTTCGTTTTCTCGCCATTGGAAGGAGTGAAAACTTGACTCACTTGTTTGAAGATATCAACAAACTCAATTCCCTCAAAACACTGATCATTTATGAGTGCAAATCGCTGCTAACACTGCCAAAAGGCTTGGAAAACATGAAATCTATATGTAATATGGGAATATGGGAATGTGATCGGCTGAGATTTACATTCTCACTGGCTTCACTTCACCTCAAGAAACTGATACTCAGAGAACTTACAGCAGTGTCCACTTTGCCTAATTGGCTGTCCAATTTGGATGGTACTTTAGAAGTGCTAGAAATTGGAGAGTTCCCCACGCTAAGAAAATTGCCAATCTGGCTTTTAAACTTTTGGGAACTCCGAATTCTTGGGATCTCCAACTGTCCTAAGTTGAAGCATGATTCCTTCCCTCCTGAGCTAAATTATTTTTGTGATAAGATTGAGGAGTTGAGGATCACATTTTGTGGGTCTTTGAGCAAGTCTTTGTTGAAAAAAAGCATGAAGGAAATTGAACCTGAAAGCCGGGTAATCTTTTACATCCATACCATTTATGTGGACTCCAAAAGAATGACGCCACCAGTAGAATCAACAGACGAACCTAAGGAAGCAGAGACAAAACAGGATGATGCTTATAACAATGCAAGTCCTCCTGGGACTGAACAACCTTCAAAGACTAAACATGATGATGCGAATAACAATATGAGTCATCCTGGGATTGGACTACTTTCAGAGTCAAAACAGGAGCATACAAATAACAATATAAACGAGATTGAGACTGTTAAGGTTTGTTTGGGTGATAATGACCATGCTGAAGCTCACCAAGCTATG

>Cucsa.338650

ATGGCCGACGAGCTCCGACCTCAACACGGGAATTGGACTTACGATGTTTTCTTGAGTTTTAGAGGTGAAGATACTCGCAAGAACTTCACTGATCATCTCTACTACGCATTCAAAGATGCAGGCATCAATGTGTTTCGAGACGATCCAGAGCTCGAACGGGGTGAAGACATAAGTTCGGAGCTCGAGCGAGCGATCGAAGGGTCGAAGGTGGCAGTTGTCGTATTCTCGGAAAGGTATGCGGAGTCGGGATGGTGTTTGGAGGAGTTGGTAAAGATCATGGAGTGCAGGAGGACTTTGAGACAACTGGTTTTCCCAATATTTTATAATGTGGATCCTTCATGTGTGAGGAAGCAAAAGGGTGAATTTGAAGAGGCTTTTGTTAAACATGAAGTGCGTTATTTTAGGGATATTGATAGAGTTCTTAAGTGGAGAATGGCTCTCACTGAAGCTGCTAATTTATCTGGTTGGGATTTGAGAAACATTGCAAATGGACATGAAGCGAAGTTCATAAGGTTGATTGTTGAAAAGGTATCAAAGGAGGTGAACAGTAAATACTTATTCATAGCTCTTTATCCAGTGGGAATTGAATCAAGACTCAAACTTCTTTTATCACATCTTCATATTGGTTCAAATGATGTTAGGTTTGTAGGAATTTTGGGGATGGGAGGACTGGGTAAAACCACCGTTGCAAAAGCACTTTACAACCAGCTTTATCACAACTTTGAAGCCAAATGTTTCCTTTCCAATATCAAAGCTGAAACCTCCAATCTAATTCACTTACAAAAACAACTCCTCTCTTCCATCACAAATTCTACCAACATCAATCTTGGAAACATCGACCAAGGAATCGCAGTGTTGCAAGAAAGACTTCGTTGCAAAAGGCTTCTTTTGATATTAGACGATGTAGACGACTTAAGCCAGTTAACTGCATTAGCAACAAGTCGTGATTTGTTTGCTTCAGGTAGTAGAATTATCATAACAACTCGAGATCGACATCTGCTAAATCAGCTTGAAGTAGACGAAATTTGTTCCATCGATGAAATGGATGACGATGAAGCACTTGAACTCTTTAGTTGGCATGCTTTTCGCAATAGTTATCCATCAGAAACCTTTCATCAACTTTCGAAACAAGTGGTCACTTATTGTGGAGGATTGCCATTAGCTCTCGAAGTGTTGGGTTCTTTCCTTTTTGGTAGAAGTAGAGAAGAATGGGAAGATACACTGAAGAAATTGAAGAAAATCCCAAACGATCAAATTCAAAAAAAGCTTAAAATAAGCTTTGATGGGCTAAACGATCATACTTACAAAGATATATTTCTCGACGTGTCATGTTTCTTTATTGGAATGGAAAGAAACTACGTTGAACAAATATTAGATGGGTGTGGATTTTTTCCAAGAATCGGAATTAGTGTTCTTCTTCAAAGATGTCTATTAACAATTGGAGACAAAAACAGATTAATGATGCATGATTTGTTAAGAGATATGGGGAGAGAAATTGTTCGTGAAAATTTTCCAAAATACCCTGAGAGACATTCAAGACTTTTTCTTCATGAGGAAGTGCTTTCTGTTCTTACAAGACAAAAGGGAACTGATGCAACTGAAGGCCTAAGTTTGAAGTTGCCAAGATTTAGCAAGCAGAAGTTGAGCACAAAAGCATTTAATGAAATGCAAAAATTGAGGTTACTTCAACTTAATTTTGTTGATGTAAATGGAGATTTCAAGCATATTTCTGAAGAGATAAGATGGGTTTGTTGGCACGGATTTCCTTTGAAGTTTTTGCCTAAAGAATTTCATATGGACAAATTGGTTGCTATGGACTTGAGATATAGCCAAATCAGATTCTTTTGGAAGGAGTCTAAGTTTCTCAAGAATTTGAAGTTTCTTAATCTAGGCCATTCTCATTACTTAACCCACACTCCAAATTTCTCCAAACTCCCCAATCTAGAGATACTCAGCCTCAAAGACTGCAAGAATTTGATTGAATTGCACCCTACAATTGGAGAATTAAAAGCCCTCATTTCCCTAAACTTAAAAGATTGCAAATCCCTCAATTCACTTCCAAATAGTTTCTCCAACTTAAAATCCTTACAAACTCTCATTATTTCAGGTTGTTCAAAGCTCAATAGTTTGCCAGAAGATTTAGGCGAAATTACATCATTAATAACTCTAATAGCTGATAACACACCAATCCAAAAAATCCCTAACACAATTATAAACTTAAAAAACCTCAAATATTTATCTTTATGTGGGTGCAAAGGGTCACCATCAAAATCATCATTCTCTTCAATGATTTGGTCTTGGATTTCACCAAAGAAATTATCTCAAAACTACACATCAATTCTTCTCCCTTCTTCATTACAAGGCTTAAACTCCTTAAGAAAATTATGCCTTAAAAATTGTAACTTGTCAAATAACACAATTCCAAAAGATATCGGGAGTTTGAGTTCTTTGAGAGAATTGGATTTGAGTGAGAATTTATTCCACAGTTTGCCATCAACTATCAGTGGCCTTTTGAAACTTGAGACACTTTTGTTGGATAATTGCCCTGAACTTCAACTTATACCAAATTTGCCACCACATTTGAGTTCATTGTATGCATCAAACTGTACTTCATTGGAAAGGACTTCAGATTTGTCTAATGTGAAGAAAATGGGATCTTTGTCTATGAGTAATTGTCCTAAACTTATGGAGATTCCTGGCTTGGACAAATTATTGGATTCTATTAGAGTTATTCACATGGAAGGATGTAGCAACATGTCCAATTCCTTCAAGGATACCATTCTACAGGGATGGACAGTTAGTGGATTTGGAGGAGTATGTCTTCCAGGCAAAGAAGTTCCAGATTGGTTTGCATACAAAGATGAAGGTCACTCAATATTTTTAGAATTGCCTCAGTATAATAATTCCAATTTAGAAGGCTTCATTGTTTGCATAGTTTACTGTTCTTGTTTTAACAACACAGTCTCAACTGACCTTCCAAGTTTATCAGTCATTAATTACACAAAATCTTCCATTACAACCAACAAACCTCTTACCAATGATGTAATAATGTCAACTCAAGATCACTTGTGGCAAGGCCATTTATCTAACAAAGCCTTCAAGATGGAACCTGGCGATGAAGTCGAGATCATCGTTGATTTCGGTGCTGAAATCACCGTGAAGAAAATTGGCATCTCGCTTGTGTTTGACAAGTATGTCGATCAAACAATGTTAGAGTTTGCATCCACCTGTAATGATGATGATGTCGTCGTGGATAACCAAGATGAAACTGTAAGTGAAAAGGATGGAGAAGTTGGGAGCAAGAGAGGTTTTGACGAGAATGATGATGAAGGATTGAAAAATTCATACCAAATTCCCAAAAGGTTGAAGTGTGAGATTGATTCTAACATGAAAATTGATGAGGAGTAG

>Cucsa.338660

ATGGCCAACGAGTTCCAAGCTCAACATGGAGACTGGACGTACGATGTTTTCTTAAGTTTTAGAGGCGAAGATACTCGAAAAAACTTCACAGATCATCTCTACTACGCATTGAAAGATGCAGGCATCAATGTCTTTCGAGACGACCCAGAGCTCCAACGAGGCGAAGACATAAGTTCGGGGCTGGAGCGAGCAATCGAAGGATCGAAGGTGGCAGTTATCGTATTCTCGGAAAGGTATGCGGAGTCGGGATGGTGTTTGGAGGAGTTGGTAAAGATCATGGAGTGTAGAAGGACTTTGAGACAAATGGTTTTGCCAGTATTTTATAATGTGGATCCTTCATGTGTGAGGAAGCAAAAGGGTGAATTTGAAGAGGCTTTTGTTAAGCATGAAAAGGGTAAGGATATTGATAAAGTTCGTAGGTGGAGAATGGCTCTCACTGAAGCTGCTAATGTAGCTGGTTTGGGTTTGACACAAAATGCAAATGGGCATGAGGCAGAATTCATAAGGTCAATTGTTAAAATGATATCAAAGGAGGTGAAGAGCAATTACTTATTCATAGCTCTCTATCCAGTGGGAATTGAATCAAGAATCAAACTTGTTTTACCACATCTTCATATTGGTTCAAATGATGATGTTAAGTTTGTAGGAATTTTGGGGATTGGAGGTTTGGGAAAAACCACCATTGCAAAAGCACTTTACAATCAACTTCATCACAACTTTGAAGCTGCATGCTTCCTTGCTAATATCAAACAAACCCCCAACCAACCCAATGGTCTAGTTCACTTACAAAAACAACTCCTCTCTTCGATTACAAATTCCAGTAACATCAATTTCGAAAACATGGATCGAGGAATCGTTGTGTTGCAAGAAAGCCTTCGTCGCAAAAAGCTTCTTTTGATATTAGACGATGTAGACAAAATAAGCCAATTAACTGCATTAGCAACAAGACGTGAATGTTTCGGTTCAGGTAGTAGAATTGTCATAACAACTCGACATCGACGTTTACTAAACCAGATTGAAGTAGATGGAATTTGTTCCATTGATGTAATGGATGACGCTGAAGCGCTCCAACTCTTTAGTTGGCATGCCTTTCACAATAGTTATCCTTCAGAAACTTTTCATCAACTTTCAAAACGTGTTGTTAATTATTGTGGAGGATTGCCATTAGCCCTTCAAGTGTTGGGCTGTTTCCTTTTTGGCAGAAGTAGAGAAGAATGGCAAGATACATTGAAGAATTTGAAGAAAATTCTAGATGATCAAATTCAAATAAAGCCTAAAATAACCTTTGATACCCACAATGATCACACTTGTAAAGATATATATCTTGTGAACCAAATGTTAGATGGGTGGGGATCTTTTCCAAGAATTGGTGACATAAACAGATTAGTGACAAGTGATTTGTTAAGAGATCATACCCAACTTTTTCTTCCGAAGGAAGTGCGCCTTTCTGTACTTGGACCAAAGGTAAGAAAATAA

Protein sequences of the NBS-encoding genes from cucumber (Cucumis sativus L.).

>Cucsa.017460

MAEFLWTFAVQEILKKVLTLVAEQIILAREVKDVLQQLQKELVESQKIVSAITTQRQNHYSPDSLVTQWVNDLQLIVHEADDLLDFILNKNQPIERLWSVISLSCLLYSSNPETKKMKEIIALLNKHCTKLPHLLQLEPTPSNIAETEVAQIQETVSKPEDYVVGRNREVETIVDRVIDASKQELNSILPVFGMGGLGKTTLAKSVFNHDRIKNHFGITIWIYVSQPFVINNILQAILQKVEVHSSDCSNNREALLEKLTENMGEKTYFLVLDDVWNENKMLWEKLKECLMSITHMSGNSILVTTRSSGIAKMMEENIGSHELRKLSDDQCWSIFRNFANAKDVPMTSNLEFVQKEFDKRIGGLPLIAKVLGAAVPFSGDHDQWVANIKSVLTTPIKEEEFVKFTLKLSVDRLPNASVKQCFAYCSNFSKGCEFDKKQVIRMWMAQGFTQPDERNNETMEDTGERYFNILLSFCLFQDVVKNERGIIEKVRMHDLIHDIACQVSNDKKLRIDHIISSNWKDWTKDDKILVSKLRTINFYDRHHVVVQDKIGDFTGLRVLTIENYIVEELPNSIFKLKHLRYLDISYCYSIKKLPESIVLLYNLQTLRFHLLSKGFLPKNVGQMISLRHLEFSSIDKQMSPYLSQLIQLETLPKFAVGFEKGCKITELGVLRNLKGLLKLQRLEHVESKEEAETAKLVEKENLEEVHFVWTKERKRKVENKNDLEVLEGLQPPKNVEYLRIKYFLGGCLPNQTFVENLVKIELRDCGNCEKLPRLGQLGNLEILDISWFERVKSIGNEFYGNSSNNQRSLFPRLKELYVDEMRRIGEWEEVGSNVKAFPRLERLYIGCCRDLVKIPDVFGYCDEYGEKHLEVVEIIEHL

>Cucsa.017490

MAGVGKTYFLNEVKKLVLKGEDRLFDRVIDVRVGRFNDVTDIQEQIGDQLNVELPKSKEGRASFLRNNLAKMEGNILILLDDLWKEYDLLKEIGIPLSKDGCKVLITSRSQDILTNNMNTQECFQVSSLSEEESWKFFMAIIGDKFDTIYKKNIAKNVAKECGGLPLALDTIAKALKGKDMHHWEDALTKLRNSIGMDIKGVSDKVYASLRLSYDHLDGEETKLIFLLCSVFPDDYKISIKNLQMYAMCMRLLNKVKTWEDSKNRVMKLVNDLISSSLLLEAESDSKDKYVKMHDVVRDVAIHIASKEGNMSTLNIGYNKVNEWEDECRSGSHRAIFANCDNLNNLPLKMNFPQLELLILRVSYWLVEDNLQIPYAFFDGMVKLKVLDLTGMCCLRPLWTTPSLNNLQALCMLRCEFNDIDTIGELKKLEVLRIVKCNMLDHLPPTMSQLTHLKVLEVLNCPKLEVVPANIFSSMTKLEELKLQDSFCRWGEEVWYKDRLVKNVTVSELNCLPCLSNLSLESWNVKILSEISSQTCKKLKEFWICSNESDDFIQPKVSNEYARTLMLNIESQVGSIDEGLEILLQRSERLIVSDSKGNFINAMFKPNGNGYPCLKYLWMIDENGNSEMAHLIGSDFTSLKYLIIFGMKRLENIVPRHISLSPFKKVKTIAIQFCGQIRNLFSFSIFKDLLDLQEIEVINCGKMEGIIFMEIGDQLNICSCPLTSLQLENVDKLTSFCTKDLIQESSQSIIPFFDGQVSFPELNDLSIVGGNNLETLWHKNNNPTTGSFCKLQSIRIEQCTQLRCMFPSNMLTSLASLHTIQIISCASLKRIFEIENQSFNDTTVLWSLNELHLLNLPNLKHVWRKDIIKILTFPSLKRVKIHGCTKLTHVWKDNNKVTRSFDSLERIEVEKCKNLKYLLPSSIAFLNLKELHIKKCNGMINLFSSTVTKKLVNLSSIKVSYCKGMRCMVEVDQAENDEIITFKKLSTLELDYLPRLDSFYSGKCMLEFPCLESLVIKRCPEMKTFSYGVIIAPRLQTLWMNDKEFGVSSPACGINETIQNFPRRVVCMFNSN*

>Cucsa.088220

MGGVGKTMLVKEILRKIVESKSFDEVVTSTISQTPDFKSIQGQLADKLGLKFERETIEGRAPSLRKRLKMERRILVVLDDIWEYIDLETIGIPSVEDHTGCKILFTSRNKHLISNQMCANQIFEIKVLGENESWNLFKAMAGKIVEASDLKPIAIQVVRECAGLPIAITTVAKALRNKPSDIWNDALDQLKSVDVFMTNIGEMDKKVYLSLKLSYDCLGYEEVKLLFLLCSMFPEDFSIDMEELHVYAMGMGFLHGVDTVVKGRRRIKKLVDDLISSSLLQQYSEYGYNYVKMHDMVRDVAIFIASKNDHIRTLSYVKRLDEEWKEERLLGNHTVVSIHGLHYPLPKLMLPKVQLLRLDGQWLNNTYVSVVQTFFEEMKELKGLVLEKMNISLLQRPFDLYFLANIRVLRLRGCELGSIDMIGELKRLEILDLSGSNIIQIPTTMGQLTQLKVLNLSNCFNKLEIIPPNILSKLTKLEELRMGTFGSWEGEEWYEGRKNASLSELRFLPHLFDLDLTIQDEKIMPKHLFSAEELNLEKFHITIGCKRERVKNYDGIIKMNYSRILEVKMESEMCLDDWIKFLLKRSEEVHLEGSICSKVLNSELLDANGFLHLKNLWIFYNSDIQHFIHEKNKPLRKCLSKLEFLYLKNLENLESVIHGYNHGESPLNNLKNVIVWNCNKLKTLFLNCMLDDVLNLEEIEINYCKKMEVMITVKENEETTNHVEFTHLKSLCLWTLPQLHKFCSKVSLPNLEKLKIWCTKDLKKIWSNNVLIPNSFSKLKEIDIYSCNNLQKALFSPNMMSILTCLKVLRIEDCKLLEGIFEVQEPISVVEASPIALQTLSELKLYKLPNLEYVWSKDSCELQSLVNIKRLTMDECPRLRREYSVKILKQLEALSIDIKQLMEVIGKKKSTDYNRILINKLVIGQVEVLQLGDGSELFPKLKTLKLYGFVEDNSTHLPMEIVQNLYQFEKFELEGAFIEEILPSNILIPMKKQYNARRSKTSQRSWVLSKLPKLRHLGSECSQKNNDSILQDLTSLSISECGGLSSLVSSSVSFTNLTFLKLNKCDGLTHLLDPSMATTLVQLKQLRIGECKRMSRIIEGGSSGEEDGNGEIIVFNNLQLLIITSCSNLTSFYRGRCIIQFPCLKHVSLEKCPKMKSFSFGIVSTSHSKYENVSLKNDDDDTHYRPKESKER*

>Cucsa.089350

MSDKKIVMLGLYGIGGIGKTTLAKALYNRIAHDFEGCCFLEKIREASNQYDGLVQLQKKILCDILMDNSINVSNLDIGVNIIRNRLCSKKILLILDDVDTREQLEALAGGHDWFGHGSKIIATTRNMQLLASHGFNKLEKVNGLNAIEGLELFSWHAFNNCHPSSDYLDLSKRAVHYCKDLPLALEVLGSFLNSIHDQSKFERILDEYKNFYLDKDIQDILRISYDELEQDVKDIFLYISCCFVGEDINEVKMKLEACGCLCLEKGTTKLMNLSLLTIESNRIKMHDLIQQMGRSIHLSKTFTSHKRKRLLIKDDAMDVLNGNKCDLTTAINLEKLNLEGCEKLVKVHESVGSLSKLVEFYLSSSVEGFEKFPSCLKLNSLEALVVRYCRIEECCPQFSEEMNSLEILEIDDSIINQLSPTIEYLTGLKELWITECTKLETLPSTIYRLSNLTSLEVKKSDLSIFPSLNDPSSSSLLIPYLTSIKLFNCQITNLDFLETMVHVTPSLEMLDLSQNNFCGLPSCIINFKSLKYLYIIECKSLEEILKVPKGVVRMDTRGCVSLAKFPNNIPDFISCDDNVEYDTKDGVIKQLILMNCDIPDWCNRMSISNFNFWLSRGECLWMAVLHPCMHRLINPYGDDIMDISPNFSIGILDNKITLLFEVNPECKDT

>Cucsa.091460

MGSSVVGDESFSSSPNFNYDYDVFFSFRGEDTRSNFISHLHMALRLKEVNVFIDDKLKRGEQIYESLLKFIERSRLSLVIFSKDYASSTWCLDELVKIIECKKSKGQAVWPVFYKVDPSEVRKQTGGFGEALAKHEANKLLANKIQPWREALTFAAGLSGWDLANSKDEAELIQKIVKRVLSAVNPMQLLHVAKHQVGVDSRLRKIEELVSHIGSEGVNLVGLYGIGGIGKTTLAKALYNKIATQFEGCCFLQDVRREASKHGLVQLQETLLNEILKEDLKVIVSRDRGINIIRSRLCSKKVLIVLDDVNDLEQLEALVGGRDWFGQGSKIIVTTRNEHLLSSHGFDEKHKIQELNQDHALELFSWHAFKKSHPSSNYLDFSKRATSYCKGLSLALVVLGSFLCGRAKEEWNGILDEFENSLRKDIKDVLQLSFDGLEDKIKDIFLDISCLFVGEEYKCAKKMLSACHLNIDFGIMILMDLSLITVEMDRVQMHELIQQMGRSIVHNESSEPGKRSRLWLEHDIWEVFVNNSGTDAVKAIKLDLPKSTRLNVDPRAFGSMKNLRLLIIRNARFCTKIRYLPNSLKWIEWHGFAHRTLPSCFITKNLVGLDLQHSLIKRFGKRLKVSSRNSEPSSKHTNFECEWL*

>Cucsa.091470

MDSSTVATESPTFKWTYDVFLSFRGEDTRTNFTSHLDMALRQKGVNVFIDNKLERGEQISESLFKSIQEASISIVIFSQNYASSSWCLDELVNIIECKKSKGQNVFPVFYKVDPSDIRKQTGSFGEALAKHQPKFQTKTQIWREALTTAANLSGWNLGTRKEADLIGDLVKKVLSVLNRTCTPLYVAKYPVGIDSKLEYMKLRSHNLFEKSNKFHYRKQHEYESDTGVYMVGLYGIGGIGKTTLAKALYNKIASQFEACCFLSNVREASKQFNGLAQLQETLLYEILTVDLKVINLDRGINIIRNRLCLKKVLIVLDDVDKLEQLEALVGGRDWFGQGSRIIVTTRNKHLLSSHGFDEMENILGLDEDEAIELFSWHAFKKNHPSSNYLDLSKRATSYCKGHSLALVVLGSFLCTRDQVEWCSILDEFENSLNKDIKDILQLSFDGLEDKVKDIFLDISCLLVGEKVKYVKNMLSACHVNLDFGIIVLTDLSFITIENDIMQMHDLIKQMGHKIVCGESLELGKRSRLWLVQDVWEVLVNNSGTDAVKGIKLDFPNSTRLDVDPQAFRKMKNLRLLIVQNARFSTKIEYLPDSLKWIKWHGFRQPTFPSFFTMKNLVGLDLQHSFIKTFGKRLEDCERLKYVDLSYSTFLEKIPNFSAASNLEELYLTNCTNLGMIDKSVFSLDKLTVLNLDGCSNLKKLPRGYFMLSSLKKLNLSYCKKLEKIPDLSSASNLTSLHIYECTNLRVIHESVGSLDKLEGLYLKQCTNLVKLPSYLSLKSLLCLSLSGCCKLESFPTIAKNMKSLRTLDLDFTAIKELPSSIRYLTELWTLKLNGCTNLISLPNTIYLLRSLENLLLSGCSIFGILPSCLHKFMSLWNLELRNCKFLQEIPSLPESIQKMDACGCESLSRIPDNIVDIISKKQDLTMGEISREFLLTGIEIPEWFSYKTTSNLVSASFRHYPDMERTLAACVSFKVNGNSSERGARISCNIFVCNRLYFSLSRPFLPSKSEYMWLVTTSLALGSMEVNDWNKVLVWFEVHEAHSEVNATITRYGVHVTEELHAIQTDVKWPMVNYADFYQLEKLQSLDIEELLLKRLFEEMSCWSNSQAMLYAANYDPEAIIDSNIQPMIFPLHVTYNGETFICGMEGMGDTTLANSLCNKFNRSNDNGWPREALDDSTSFLHFRGGKFYGGSWSLSHHRKRGDGERGTNITTRTISSKRYLILFHKAGSYNHLFNFAGSHRLIAGSGSYDSLNGRGDVRLLIERVDTSLL*

>Cucsa.091680

MQLDVAKYPVGIDIQVRNLLPHVMSNGITMFGLYGVGGMGKTTIAKALYNKIADEFEGCCFLSNIREASNQYGGLVQFQKELLCEILMDDSIKVSNLPRGITIIRNRLYSKKILLILDDVDTREQLQALAGGHDWFGHGSKVIATTRNKQLLVTHGFDKMQNVGGLDYDEALELFSWHCFRNSHPLNVYLELSKRAVDYCKGLPLALEVLGSFLHSIGDPSNFKRILDEYEKHYLDKDIQDSLRISYDGLEDEVKEIFCYISCCFEARAVKVIKLNFPKPTKLDIDSRAFDKVKNLVVLEVGNATSSESSTLEYLPSSLRWMNWPQFPFSSLPTTYTMENLIELKLPYSSIKHFGQGYMSCERLKEINLSDSNLLVEIPDLSTAINLKYLNLVGCENLVKVHESIGSLKLTTLPSTIYRLTNLTSLTVLDSNLSTFPFLNHPSLPSSLFYLTKLRIVGCKITNLDFLETIVYVAPSLKELDLSENNFCRLPSCIINFKSLKYLYTMDCELLEEISKVPEGVICMSAAGSISLARFPNNLADFMSCDDSVEYCKGGELKQLVLMNCHIPDWYRYKSMSDSLTFFLPADYLSWKWKPLFAPCVKFEVTNDDWFQKLECKVFINDIQVWSSEEVYANQKERSGMFGKVSPGEYMWLIVLDPHTRFQSYSDDIMDRRSLKIIDLNQLSSEINSSQSILGKITVSFEVTP

>Cucsa.091690

MSNEITMVGLYGIGGMGKTTLAKALYNKISDDFEGCCFLANVREASNQYWGLVELQKTLIREILMDDSIKVSNVGIGISIIRDRLCSKKIILILDDIDTHEQLQALAGGHDWFGHGSKVIATTRNKQLLASHGFNILKRVNGLNAIEGLELFSWHAFKNSHPSSDYLDVSKRAVHYCKGLPLALEVLGSFLNSIDDQSKFERILDEYENSYLDKGIQDILRISYDELEQDVKEIFLYISCCFVHEDKNEVQMMLKECDSRFRLEMGIKKLTDLSLLTIDKFNRVEMHDLIQQMGHTIHLLETSNSHKRKRLLFEKDVMDVLNGDMNCKWLKRINLNYSKFLEEISDLSSAINLEELNLSECKKLVRVHESVGSLGKLAKLELSSHPNGFTQFPSNLKLKSLQKLVMYECRIVESYPHFSEEMKSSLKELRIQSCSVTKLSPTIGNLTGLQHLWIDVCKELTTLPSTICHLSNLISLSVFRSEVSTFSFLYSRSLSLFPYLTLLKLCYCKITNLSFLETITHVAPSLTQLYLTGNDFCSLPSCIVNFKSLRYFDISYCGSLARFPDNIAEFISCDSEYVDGKYKQLILMNNCDIPEWFHFKSTNNSITFPTTFNYPGWKLKVLAACVKVQVHDPVNGYHRGGDLECEVFFKDILVWSSGDWTNYLGYDSRWLPLGASPSEYTWFIVLNPHRDFSLDDWDDMMERSPETDLSQLCFGINSMEMDRNRSNDKWNSIGGSIWKNFTVLFEPRPLSPDTTI

>Cucsa.091710

MVGLHGIGGMGKTTLAKTLYNRIADDFEGCCFLANIREASKQHEGLVRLQEKLLYEILMDDFIRVSDLYKGINIIRNRLCSKKILLILDDIDTSEQLQVLAGGYDWFGYGSKVIVTTRNEHLLDIHGFNKLRSVPELNYGEALELFSWHAFQCSSPPTEYLQLSKDAVNYCKNLPLALEVLGSFLYSTDQSKFKGILEEFAISNLDKDIQNLLQVSYDELEGDVQEMFLFISCFFVGEDKTMVETMLKSCGCLCWEKGIQKLMNLSLLTINQWNKVEMHDLIQQLGHTIARSKTSISPSEKKLLVGDDAMHVLDGIKHCERLKQLDLSNSFFLEEIPDLSAAINLENLSLSGCISLVKVHKSVGSLPKLIDLSLSSHLSSTIRYLTSLKDLTIVDCKKLTTLPSTIYDLSKLTSIEVSQSDLSTFPSSYSCPSSLPLLTRLHLYENKITNLDFLETIAHAAPSLRELNLSNNNFSILPSCIVNFKSLRFLETFDCKFLEEIPKIPEGLISLVNHVDRNRYMDLECKMFINDIQILSRDHRSTLIYISQVLGSIPWEGTIIIVIFEHRILSTYKFFSFLPISSPK

>Cucsa.091780

MAKHQPKFQTKTQIWRKALTTAANLSGWDLGAYRREADLIRDLVKEVLSTINRTRTPLYVAKYPVGIDSQLEYMKFHSHHLNKGNKFQYWTQNEYESDIGVYMVGIYGIGGLGKTTLAKALYNKIASQFEGCCFLSNVRQASNQFNGLVQLQQNLLYEILEDDLKFVNLDKGITIIRNRLRSKKVLIVLDDVDKLEQLEALVGGRDWFGQGSKIIVTTRNSHLLSSHGFDEMHNIQGLNQDRAIELFSWHAFKESHPSSNYLDLAERATSYCKGHPLALVVLGSFLCNRGQTEWRSILDKFENSLNNDIKDILQLSFDGLEGGVKDIFLDISCLFVGEKYNNCAKKMLSACHLNVDFGIMILMDLSLVTIEKDRVQMHGLIQQMGHSIVHNESFESGKRSRLWSERDIWNVFVNNSDCERLKHVDLSYSTLLEKIPDLSAASNLEELYLINCTNLGMIDKSVFSLNKLTVLNFKGCSNLKKLPKGYFMFSSLKILNLSYCQELEKIPDLSSASNLQSLLLNGCTNLRVIHESVGSLNELVLLDLGQCTNLSKLPSYLRLKSLVYLVLFGCGKLESFPTIAENMKSLRCLDLHSTAIKELPSSLGYLTQLDKLHLTGCTNLISLPNTIYLLRNLNELHLGGCSRFEMFPHKWVPTIQPVCSPSKMMEAASWSLEFPHLVVPNESICSHFTLLDLKSCNISNAKFLEILCDVAPFLSDLRLSENKFSSLPSCLHKFMSLSNLELRNCKFLQEIPNLPQNIRNLDASGCKSLARSPDNIVDIISIKQDLELGEILREFLLTDIEIPEWFSYKTASNLVTASLRHYPDMERTLAVAVSFKVNGDSSESEAQISCNIFIYNKLRCLFSRSFLPSKSEYMWLVTISLACSLEVNDWNKVFVWFEVHEAHGVTVTRYGVHVTEQLHGIQTDVKWPMVNYADFYQLEKLRRDL*

>Cucsa.091820

MGSSIVGAESSTSSSSSFKWSFDVFLSFRGDDTRSNFTGHLDMALRQKGVNVFIDDMLKRGEQISETLSKAIQEALISIVIFSQNYASSSWCLDELVKIVECKKSKGQLVLPIFYKVDPSDVRKQTGCFGEALAKHQANFMEKTQIWRDALTTVANFSGWDLGTRKEADFIQDLVKEVLSRLNCANGQLYVAKYPVGIDSQLEDMKLLSHQIRDAFDGVYMMGIYGIGGIGKTTLAKALYNKIANQFEGFCFLSNVRETSKQFNGLVQLQEKLLYEILKFDLKIGNLDEGINIIRSRLRSKKVLIVLDDVDKLKQLEALVGERDWFGHGSKIIVTTRNSHLLSSHEFDEKYGVRELSHGHSLELFSWHAFKKSHPSSNYLDLSKRATNYCKGHPLALVVLGSFLCTRDQIKWRTILDEFENSLSEDIEHIIQISFDGLEEKIKEIFLDISCLFVGEKDCKRLKHVDLSYSSLLEKIPDFPATSNLEELYLNNCTNLRTIPKSVVSLGKLLTLDLDHCSNLIKLPSYLMLKSLKVLKLAYCKKLEKLPDFSTASNLEKLYLKECTNLRMIHDSIGSLSKLVTLDLGKCSNLEKLPSYLTLKSLEYLNLAHCKKLEEIPDFSSALNLKSLYLEQCTNLRVIHESIGSLNSLVTLDLRQCTNLEKLPSYLKLKSLRHFELSGCHKLEMFPKIAENMKSLISLHLDSTAIRELPSSIGYLTALLVLNLHGCTNLISLPSTIYLLKSLKHLYLGGCSRFQLFSHFLEILCNVAPFLSSILLSENKFSSLPPCLHKFMSLWNLQLRNCKFLQEIPNLPHCIQKMDATGCTLLGRSPDNIMDIISSKQVPHFHFHFPLVGDSYQGMALVSCKIFIGYRLQSCFMRKFPSSTSEYTWLVTTSSPTFSTSLEMNEWNHVTVWFEVVKCSEATVTIKCCGVHLTEEVHGIQNDVKGPGVVYTVFDQLDKLPSRIRGMEGMAETTLANSICNKYERSRNLFSAKKALNHSTGFLCGDGNGLSWEMVDRPILSDRLSSQKYLRIFDDRDRYGDLNDVAHGTGNRFRSRFLRMDDIKEDDIREEPYWKYME

>Cucsa.091840

MGSNAAGAESSSSSPINWIYDVFLSFRGEDTRSNFTSHLHMFLRHKGVNVFIDDRIERGEQISEALLKTIQCSLISIVIFSENYASSTWCLDELVEIIECKKSKGQKVLPIFYKVDPSDVRKQNGCYGEGLAKHEANFMEKIPIWRNALTTAANLAGWDLGTIRNEADLIQVIVKEVSSTLNVTTPSDKPLLVGIDSKIESLYWPTEEMYKSECVDMLGIYGIRGIGKTTLAKALYYKMASQFECCCFLSNVREASKQLNGLAQLQKKLLFQILKYDLEDVDDLDRRNNIIKHRLHSKKVLILLDDVDEMKQLKALAGGHDWFGQGSKIIVTTRDKHLLDSHGFGQTYEVEGLWEHNAFELFCWHAFKKSHPSSNYLDLSERATRHCKGHPLALVVLASFLCGRDQAEWSGLLDGFENSLRKGIKDVLQLSFDGLEDEVKKFFLDISCLLVGETVTYVKKMLSEFHSILDFKISNLRHLSLIRMEEYDDDRVQMHDLIKQMGHKIVYDECGDEPGKRSRSGKRCSERHKVGVYLIPPRVINVNPEAFRSMKNLRILIVDGNVRFCKKKKYVPNGLKWIKWHRFPHQTLPSCFITKDLLPSYLKLKSLTDLDLSGCRKLETFPEIDENMKSLERLRLSYTAIRKLPSVFAGLVFLSVLLKCVPKFFHSRVPKEILFFEHLELLDLKGCNISNVDFLENLCNVALSLTSIVLSENEFCSLPSCLHKFMSLRNLQLRNCMFLQEIPNLPQSIQIVDATGCISLRRSPNIMWT*

>Cucsa.091880

MESIPISIIAKICEYTVKPVGRQLCYVCFIHSNFQKLKSQVEKLTDTKGSVEDKVFIARRNAEDIKPAVEKWLEKVDRLVRKSEKILAHEGRHGRLCSTNLVQRHKASRKASKMADEVLEMKNQGESFDMVSFKGRISLVESPLPKAPDFLDFGSRKSTVEQIMDALSDDNVHKIGVYGMGGVGKTMLVKEIVRKIEESKKSFDKVVTSTISQTPDFKRIQGQLADKIGLKFEQETIEGRATFLRRWLKAERRIPSVEDHKGICKILFTSRNKQLISNDMGANKIFEIKVLGEDESWNLFKAMAGEIVEATDLKPIAIQIMRECAGLPIAITTVAKALLNKPSDIWNDALDQLKSVDVGMANIGEMDKKVYLSLKLSYDYLGYEEVKLLFLLCSMFPEDFNIDVEKLHVYAMSMGFLRGVDTVVKGRRRIKKLVDDLISSSLLQQYSEYGNNYVKIHDMVRDVAILIASQNDHIRTLSYVKRSNEEWKEEKLSGNHTVVFLIIQELDSPDFSKLMLPKVQLFVLFGPSPSIYNRHVVSVVETFYKEMKELEGLVIERVKISLSPQALYSFANLRLLRLHDCELGSIDMIGELKKLEILDFSKSNIVEIPMTFSKLTQLKVLNLSFCDELEVIPPNILSKLTKLEELHLETFDSWEGEEWYEGRKNASLSELRYLPHLYALNLTIQDDEIMPKHLFLAGELNLENFHITIGCQRQKRHIDNKTNFFRIKMESERCLDDWIKTLLKRSEEVHLKGSICSKVLHDANEFLHLKESLFSKLKSVVVTKCNKLEKLFFNCILDDILSLEEIAIHYCEKMEVMIVMENEEATNHIEFTHLKYLFLTYVPQLQKFCSKIEKFGQLSQDNSISNTVDIGIFEVQESSITDTSLIVLKNLRELKLYNLPNLEYVWSKNPCELLSFVNIKGLAIDECPRLRREYSVKILKQLERLTMDIKQLMEVIENQKSTDHNMVKSKQLETSSKDNSTHLPVEIVQILYQLEHFELEGAYIEEVFPSNILIPMKKQYYARSKNSVRSWFLSKLPKLRHLWSECSQKNAFPILQDLNVIRISECGGLSSLVSSSVSFTNLTVLKVDKCDRLTYLLNPLVATTLVQLEELTLRECKMMSSVIEGGSAEEDGNEETTNQIEFTHLKSLFLKDLPRLQKFYSKIETFGQLSRDNSENPETTTIHNRIGDSFFSEQESLPNLETLRIDGAENLRMIWSNNVLIPNSFSKLEEVEIYSCNNLQDVLFHPNIINMLTCLNTLRIKNCELLEGIFEVQEPISVTKTKTNAIVLPNNLIELELYNLPNLEYLWSKNPNFERLVTFESIRSLSIEKCSKLKGEYFLSIKTFKQLVEVLQLRDGSKLFSNLKELKLYGFVEYNSTHLPMEIVQVLNQLEKFELKGMFIEEIFPSNILIPSYMVLRKLTLSKLSKLRHLWGECSQKNNDSLLRDLTFLFISKCGGLSSLVSSSVSSFTNLRILEVEKCDGLSHLLSPSVATTLVHLEELRIEECKRMSSVIEGGSSEEDGNDEIIVFNNLQHLIISSCSNLTSFHCGRCIIQFPCLKQVYINKCTELKVFSLGIVSTPPLKYENIYLKNDDDDDTWHHPKESIEMVVETDMNVIIREYWDDNIDTRISNLFGEE

>Cucsa.094560

MGGIGKTTLAKTIFNHEEIKGHFDETIWICVSEPFLINKILGAILQMIKGVSSGLDNREALLRELQKVMRGKRYFLVLDDVWNENLALWTELKHCLLSFTEKSGNAIIVTTRSFEVGKIMESTLSSHHLGKLSDEQCWSLFKKSANADELPKNLELKDLQEELVTRFGGAPLVARMKHLRYLDISNSKIEELPNSISLLYNLQTLKLGSSMKDLPQNLSKLVSLRHLKFSMPQTPPHLGRLTQLQTLSGFAVGFEKGFKIGELGFLKNLKGRLELSNLDRIKHKEEAMSSKLVEKNLCELFLEWDMHILREGNNYNDFEVLEGLQPHKNLQFLSIINFAGQLLPPAIFVENLAVIHLRHCVRCEILPMLGQLPNLEELNISYLLCLRSIGHKVLFPKLKKFVLSQMPNLEQWEEVVFISKKDAIFPLLEDLNISFCPILTSIPNIFRRPLKKLHVYGCHEVTGLPKDLQLCTSIEDLKIVGCRKMTLNVQNMDSLSRFSMNGLQKFPQGLANLKNLKEMTIIECSQDCDFSPLMQLSSLVKLHLVIFPGSVTEQLPQQLEHLIALRSLYINDFDGIEVLPEWLGNLTSLEVLGLYYCINLKQFPSKKAMQCLTQLVHVDVHNCPSSQILSHDLKAKAHAKANLVQW*

>Cucsa.094580

MAIAEFLWTYAVQQVLKKVLELAADQIEKKLHHSSVRLWVADLLLVVHEADNLLDELVYEYLRTKVEKGSINKVCSSVSSLSNIFIIFRFKMAKKIKSIIEKLRKCYYEATPLGLVGEEFIETENDLSQIRETISKLDDFEVVGREFEVSSIVKQVVDASNQYVTSILPIMGMGGIGKTTLAKTIFNHEEIKRHFDETIWICVSEPFLINKILGAILQMIKGVSSGLDNKEVLLQELQKVMRGKRYFLVLDDVWNENIALWTELKKCLLCFTEKSGNGIIVTTRSIEVGKIMESTLPSHHLGKLFDEQCRSLFKESANADELPMDPELKDLQEELVTRFGGVPFVARLGSSMKHLPYNLSKLVSLRHLKFSIPQTPPHLSRLTQLQTLSGFAVGFEKGCKIEELGFLKNFKGRLELSNLNGIKHKEEAMSSKLVEKNLCELFLEWDLHILREGSNYNDLEVLKGLQPHKNLQFLSIINYAGQILPPAIFVENLVVIHLRHCVRCETLPMLGELPNLEELNISNLHCLRCIGNEFYGSYDHPNNHKVLFRKLKKFVLSEMHNLEQWEELVFTSRKDAIFPLLEDLNIRDCPILTNCPKIDLQLCTSIEDLKIVGCLEMILNVQNMHTLSRFSMNGLQKFPQGLSHLKNLKEMIITECSQDCDFTPLMQLSSLVNLDLVLFAGNGAVQLPQQLQHLTALRSLIINDFDGIEVLPEWLGNLASLEVLGLYYCRSLKQFPSKKAIAMSHPISPCGCLWLSTTTQVRRFCAMMF*

>Cucsa.094650

MAEFLWTFAVEETLKRTVNVAAQKISLVWGLEDELSNLSKWLLDAGALLRDIDREILRKESVKRWADGLEDIVSEAEDLLDELAYEDLRRKVETSSRVCNNFKFSSVLNPLVRHDMACKMKKITKMLKQHYRNSAPLGLVGKESMEKEDGGNNLRQIRETTSILNFDVVGRETEVLDILRLVIDSSSNEYELPLLIVPIVGMGGVGKTTLAKLVFRHELIKKHFHETIWICVSEHFNIDEILVAILESLTDKVPTKREAVLRRLQKELLDKRCFLVLDDVWNESSKLWEELEDCLKEIVGKFGITIIVTTRLDEVANIMGTVSGYRLEKLPEDHCWSLFKRSANANGVKMTPKLEAIRIKLLQKIDGIPLVAKVLGGAVEFEGDLDRWETTLESIVREIPMKQKSYVLSILQLSVDRLPFVEKQCFAYCSIFPKDCEVVKENLIRMWIAQGFIQPTEGENTMEDLGEGHFNFLLSRSLFQDVVKDKYGRITHFKMHDLIHDVALAILSTRQKSVLDPTHWNGKTSRKLRTLLYNNQEIHHKVADCVFLRVLEVNSLHMMNNLPDFIAKLKHLRYLDISSCSMWVMPHSVTTLFNLQTLKLGSIENLPMNLRNLVRLRHLEFHVYYNTRKMPSHMGELIHLQILSWFVAGFEEGCKIEELGNLKNLKGQLQLSNLEQVRSKEEALAAKLVNKKNLRELTFEWSIDILRECSSYNDFEVLEGLQPPKNLSSLKITNFGGKFLPAATFVENLVFLCLYGCTKCERLPMLGQLANLQELSICFMDSVRSIGSEFYGIDSNRRGYFPKLKKFDFCWMCNLEQWELEVANHESNHFGSLQTLKLDRCGKLTKLPNGLECCKSVHEVIISNCPNLTLNVEEMHNLSVLLIDGLKFLPKGLALHPNLKTIMIKGCIEDYDYSPFLNLPSLTKLYLNDGLGNATQLPKQLQHLTALKILAIENFYGIEVLPEWLRKLTCLETLDLVHLVKSLPSLQGEDNRVAEIAYETGEMETYECDNKRSWFRLHLNFYGCTLVELV*

>Cucsa.094660

MADFLWSFAVDEVLKKTVKLVAEQIGMSWGFKKDLSKLRDSLLMVEAILRDVNRIKAEHQALRLWVEKLEHIVFEADVLLDELSYEDLRRKVDARPVRSFVSSSKNPLVFRLKMANKIKAIAKRLDEHYCAASIMGLVAITSKEVESEPSQILETDSFLDEIGVIGREAEVLEIVNKLLELSKQEAALSVLPIVGIGGLGKTSLAKAIFHHEMIRENFDRMIWVCVSEPFVINKILRAILETLNANFGGLDNKEALLQELQKLLRNKKYFLVLDDVWNENPDLWNELRACLLKANKKFGSVIVVTTRSDEVANIVETNHQRHRLRKLSNDYCWTLFEKCAFGSDLPVTPRVDHVIREELVKRFGGIPLVVKVFGGMVKLDKNKCCQGLRSTLENLIISPLQYENSILSTIKLSVDRLPSSSLKQCFAYCSNFPRGFLFIREPLVQMWIAQGFIHLPSGSNVTMEDIGANYFNTLLSRSLFQDVVKDDRERILYCKMHDVVHDVACAISNAQKLRLSGKSNGDKALSIGHEIRTLHCSENVVERFHLPTFDSHVFHNEISNFTYLCVLIIHSWFIHQLPDSIAKLKHLRYLDISHSLIRTLPDSIVSLYNLQTLRLGSKIMHLPTKLRKLVNLRHLEFSLSTQTKQMPQHLSRLLQLQTLSSFVVGFDKGCKIEELGPLNNLKGELSLFHLEHVKSKTEAMAANLAMKENISDLYFQWSLLSEREDCSNNDLNVLEGLRPHKNLQALKIENFGGVLPNGLFVENLVEVILYDCKRCETLPMLGHLSKLELLHIRCLDSVKSIGDEFYGNNNSYHNEWSSLLFPKLKTLHISQMKSLELWQEIGSSSNYGATFPHLESLSIVWCSKLMNIPNLFQVPPKLQSLKIFYCEKLTKLPHWLNLCSSIENMVICNCPNVNNNSLPNLKSMPNLSSLSIQAFEKLPEGLATIHNLKRLDVYGELQGLDWSPFMYLNSSIEILRDIDSLPEWLGNLTSLETLNLRYCKNLKSFPSIEA

>Cucsa.094670

MDLLYSKNYEDKILSNLRDSLLMVEAILRDVDRIKAEHQAVKLWVEKLEAIIFEVDVLLDELAYEDLRRKVEPQKEMMVSNFISFSKTPLVFRLKMANKIKNIAKMLERHYSAASTVGLVAILSKQTEPDFSQIQETDSFLDEYGVIGRESEVLEIVNVSVDLSYRENLSVLPIVGMGGLGKTALAKVIFNHELIKGNFDRAVWVCVSEPFLIKKILRAILETLNSHFGGLDSKEALLQELQKLLNDKKYFLVLDDVWNENPILWNELKGCLLKISQRSGNVVVVTTRSDRVAEIMETHSRYHLTKLSDDHCWSLFKKYAFGNELLRIPELDIVQKELVKRFGGIPLAVKVMGGIVKFDENHEGLQKSLENLMRLQLQDENHVVSTIKLTVDRLPLPSLKQCFAYCSNFPKDFKFRKEALIQMWIAQGFIQPSLGSDEMMEDIGEKYFNVLLSRFLFQDIVKDNRGRIIFCKMHDLIHDVACAISNSPGLKWDPSDLFDGEPWRRQACFASLELKTPDCNENPSRKLHMLTFDSHVFHNKVTNFLYLRVLITHSWFICKLPNSIAKLKHLRYLDISYSTIRELPDSAVLLYNLQTLKLSRFLNGLPKNLRKLVSLRHLEFFSDPCNPKQMPQHLGKLIQLQTLSSFVVGFDDGCKIEELRSLRNLKGKLSLLCLERVKSKKEAMAANLVEKRNISYLSFYWALRCERSEGSNYNDLNVLEGLQPHKNLQALRIQNFLGKLLPNVIFVENLVEIYLHECEMCETLPTLGQLSKLEVLELRCLYSVRSIGEEFYGNYLEKMILFPTLKAFHICEMINLENWEEIMVVSNGTIFSNLESFNIVCCPRLTSIPNLFASQHESSFPSLQHSAKLRSLKILGCESLQKQPNGLEFCSSLENMWISNCSNLNYPPSLQNMQNLTSLSITEFRKLPDGLAQVCKLKSLSVHGYLQGYDWSPLVHLGSLENLVLVDLDGSGAIQLPQQLEQLTSLRSLHISHFSGIEALPEWFGNFTCLETLKLYNCVNLKDMASKEAMSKLTRLTSLRVYGCPQLNTFMFNGMSPNSSTSSSRTLSTLE*

>Cucsa.102240

MALELVGGAVLGAVVGELFKAILNLGERAISFNPVLKDIRSKLNAIMPLVKQIDELNDYLDYPKEETEKLRGLMDEGKQLLLQCGDVKLGDLNYLKRPSYTQKLRELDTALRSFMDVLMLQMARDQKKNMKMMNQMMEIICRLDNRGGSSKPMDLFVPPCLVPQLREETVGLEKPVKELKVKLLKNGVQMLVVTAPGGCGKTTLALKFCHDKEVKDIFQEKIFVPVSRKPDLKLILKDIIESLRGIQLPDLQSDERAFCYLELWLKQTSVNRPVLIVLDDVWSGQESEVLLDKLFQLPCCKILVTSRFYFPRFSESYYLEPLNHENAVQLFRRAASLDKGISKLPDDETVEKIIGGCKRLPLALKVIGRSLSHKPTSVWKVTGRNLARSGSIFDSDNELLECLQSSLDVLDDNMVTKKSFMDLGSFHEDQRISASTFIDMCTVLYTLDESEAMVTLDELSSRSLVNFVTARKYGYDDDFYEEYSFTQHDILRDLAIHLMNMEPIEQRKRLILDINGNDLPKWWVDQEKHTSYARLISITTDKRFSASWPDMEAPEVEVLILNLQSRTYNLPGFIKRMNKLKVLIITYFGSFLTEVTSEDNQLLDSLTSLERIRFERISVPIFSNPNPKPLINLQKISFFMCKFGQTFMDPSTPISDLLPNLLEISIDFCNNLSEVPNRLCEIVSLQKLSITNCHGLSSLPEDVGKLINLKNLRLRSCIHLEEFPESTTKLRELVLLDISNCIGLAKLPEKIGEFHNLEKLDMRHCWSLSKLPLSIGKLKNVKFLCDREVGEWLRKVAPRLAKQVKVQEEEANLEWLGF*

>Cucsa.123410

MAVTDFFVGEIATELLRMMVQLSTKSCLCKTTAAQIANSIQQILPIIEEIKYSGVELPAHRQFQLDRFSETLRRGIEISEKALQCGRLNIYRNLRLARKMEKLEKDICRFINGTMQAHILADVHHMRFQTTERFDRLEGVLLERRLESMKIRADASGEERWWVEEAFKKAEEEERYESNFVNIGTGLRVGKRKLKELVIGKEDLTAVGISGIGGSGKTTLAREFCKDPEVRRHFKERILFLTVSQSPDVEQLRRTIWEFVMGSDSVNSNNLILHGRPSNSALLVLDDVWSISVLENVIPNVTGCKTLVVSRFKFPEVLRETYEVELLKESEAIALFCHSAFGQQSIPLSANHNLVKQVVNECKCLPLALKVIGASLRGQSEMFWNNAKSRLSRGEPICESHENKLLQRMAISIERLSSKVRECFLDLGCFPEDKRIPLDILINVWKELHDLDDEEALAVLFELSQKNLLTLVKDARGGDIYSSYYEMYVTQHDVLRDLALHFSCQENVNDRKRLLMPKSDTELPKEWLRKSEQPFNAQLVSIHTGEMEEMDWAPMIFPEAKVLILNFSSSGYFLPSFLCNMPKIRALIVLNNNATHATLTNFSVFSSLVNLRGIWLEKISMTQLFDACTPLKHLRKLSLVFCKINNSLDEWAVDVSQIFPFLFELKIDHCNDLRKLPSSICEMQSLKCLSVTNCHNLSQLPTNLWKLKNLQILRLFACPLLKTLSPSICVLSCLKYIDISQCVYLTSLPEEIGKLTSLEKIDMRECSLIRRLPRSVVSLQSLCHVICEEDVSWLWEDLKSHMPNLYIQVAEKCFNLDWLKE*

>Cucsa.128030

MAEAILFQVAGEILMKLSSQAFQRLGMLFGLKGDLNKLTTTVSTIKDVLLDAEGRQTKSHLLQNWLHKLEEALYDAEDVLDELSTEALRRELMTRDHKNAKQVRIFFSKSNQIAFNYRMARQIKNIWERLDAIDAEKTQFHLRENCESRTQYGSFDRIMMGRETWSSSNDEEVIGRDDDIKEVKERLLDMNMNVTHNVSFIAIAGMGGIGKTTLAKSLYNDEEVSGFFDLKIWVWVSDQFEVQVVAEKMIESATKNNPSVKGMEALQAKLQKVIGERKYLLVMDDVWNESEEKWHGLKSLLMGGARGSKVLITKRDRKVATEIKSMTSLFTLEGLSESNSWLLFSKVAFKEGKESTDPSTIHLGKEILVRCGGVPLVIRHVGRMLYSKTSQEEWMSFKDNELLEVIQQDNDMTSILKLSYNHLPPNLKRCFAYSSLFPKGYKIEIKDLIRQWVAQGFIEVSNGRKSLEDTGKDYFNELCWRFFYANSSDECNINDIVCMHDVMCEFVRKVAGNKLYVRGNPNNDYVVSEQTLHISFDYGIQSWQDVLSKLCKAKGLRTILLLFRPYEKMNKIDKAILDELFSSFPRLRVLDLHFSQISVVPKSIKKLRHLRYLDLSENDMELIPHSIIELQNLQTLNLTECYELKELPRDIDNLVNLRHLTFEPCMEVTPTSEGMEKLTCLQTISLFVFDCKKTNKLWELNDLSYLTGELKIIGLEKLRSSPSEITLINLKDKKGWQGLNLEWKLGKDEYEGEADETIMEGLEPHPNVESLSINGYTGGALPNWVFNSLMKLTEIEIENCPRVQHLPQFNQLQDLRALHLVGLRSLEFIDKSDPYSSSVFFPSLKFLRLEDMPNLEGWWELGESKVVARETSGKAKWLPPTFPQLSSMPKLASIGADVILHDIGVQMVSTIGPVSSFMFLSMHGMTNLKYLWEEFQQDLVSSSTSTMSSPISLRYLTISGCPYLMSLPEWIGVLTSLETLHIKECPKLKSLPEGMQQLKSLKELHIEDCPELEDRCKQGGEDWPNISHVPNFTYKNASDIDTPQSSSGFSHHPFSIVRISVI*

>Cucsa.128100

MADSVLFNVAASVITKLGSSALRELGSLWGVNDELDKLQNTLSAIKAVLLDAEEQQSKSHTVKDWIAKIKDVFYDIDDLIDEFSYETLRRQVLTKDRTITKQVRIFFSKSNQIAFGFKMGQTIKKVREKLDAIAAIKAQLHLSVCAREVRDNEPRKVRETSSFIPEGEIIGRDEDRKSVMDFLLNTSNITKDNVEVVSIVGMGGLGKTALAQTVYNDEKINNRFKWKIWVCISQEFDIKVIVEKILESITKTKQESLQLDILQSMLQEKIYGKKYLLVMDDVWNVDHEKWIGLKRFLMGGASGSKILVTTRNLQTAQASDTVWFHHLKELDKDNSWALFRKMAFLNKEEELENSNLVRIGKEIVAKLKGYPLSIRVVGRLLYFKNTEMDWSSFKDNELDSILQEDDQIQPILKISFNHLPPKLKQCFTYCALFPKDYEFKKNGLVKQWMAQGFIQAHNKKAIEDVGDDYFQELVGRSFFQDIRKNKWGDLKYCKMHDLLHDLACSIGENECVVVSDDVGSIDKRTRHASFLLSKRLTREVVSKSSIEVTSLRTLDIDSRASFRSFKKTCHMNLFQLRTLNLDRCCCHPPKFVDKLKHLRYLNLSGLNVTFLPNSITTLYNLETLILRYCLWLRKLPKDINNLINLRHLDIYDCSSLTHMPKGLGGMTSLQTMSMFVLGKNKGGDLSALNGLKSLRGLLCIKGLQFCTTADLKNLELHWDIKMDHEDALDDGDNDDEGVLEGLKPHSNIRKMIIKGYRGMKLCDWFSSNFLGGLVSIELSHCEKLEHLPQFDQFLYLKHLLLGYLPNIEYIDSGNSVSSSTTFFPSLEKLRIESMPKLKGWWKGEISFPTTILHQLSELCIFYCPLLASIPQHPSLESLRICGVSVQLFQMVIRMATDLSEHSSSSSTLSKLSFLEIGTIDLEFLPVELFCNMTHLESLIIERCKSLQMSSPHPVDEDNDVVWKKLSNLRTLRLESILKLEYFPKSLKYITSLETLKLSNCENLVSTEGIGELISLSHLEIDRCPNLPILSEDVGDLISLSHLLIWNCPKLTSLSEGITRLTSLSSLCLEDCPNLVSLPQEFLHHHSSLPGGRFLRILNCPKLQIQDKKQKEEEEEDQEDWNELIHVLTGCR*

>Cucsa.128110

MADSILFNVAANVITKLGSSALRELGSLWGVNDELGKLQNILSAIKAVLLDAEEQQSVSHAVKDWISKLRDVFYDVDDLIDEFSYETLRRQVLTKDRTITKQVCIFFSKSNQVSFGHKMSQKIKQVREKLDAIANDKTQLHLSVRMRETRDDELRKMRETCSFIPKGEVIGRDDDKKAIIDFLLDTNTMEDNVEVVSIVGMGGLGKTAVAQSVYNDEKINEHFKLKLWVCISQEFDIKVIVEKIIEFIAKKKPDSLQLDILQSMLQEKIDGKKYLLVMDDVWNESHETWVSLKRFLMGGAKGSRILITTRNLQVAQASDTVQFHHLKELDNESSWALFRKMAFLNEEEEIENSNKVRIGKEIIAKLKGSPLTIRIVGRLLYFKNTEMDWLSFKDNDLGTILQQENQIQPILKISFNHLPSNLKHCFTYCALFPKDYEFQKDGLVKQWMAQGFIQSHSNKEIEDVGDDYFKELLGRSFFHNVKVNKWGDVKECKMHDLIHDLACWIVENECVDASDKTKSIDKRTRHVSFPSNYSRKSWELEAKSLTEVKNLRTLHGPPFLLSENHLRLRSLNLGYSKFQKIPKFISQLRHLRYLDISDHDMKFLPKFITKLYNLETLILRHCSDLRELPTDINNLINLKHLDVHGCYRLTHMPKGLGGLTSLQTMNLFVLGKDKGCDLSELNELARLRGSLLIKGLELCTTTDLKNAKYMEEKFGIQKLKLRWNRDLYDAETDYASENDDERVLDCLKPHSNVHKMQIRGYRGVKLCNWLSFDYLGGLVNIELQSCEKLQHLPQFDQFPFLKHLLLENLPSIEYIDNNNSLSSSTFFPSLEKLTIMTMPNLKGWWKGETPPESARYSALFPTILHHLSRLDISNCPQLASIPQHPPLRSLALNDVSVQLFDMVIKMATTPAADSSSALSKLSILHIQNIDLEFLPEELFGSTTDLEIFTVELKYMTTLERLDLYNCPNIVSLEGISHLTSLSSLRICNCSNLTSLPEGISHLTSLSYLTIYCVNLTSLPEGVSHLTSLSSFTIEECPCLTSLPEGVSHLTSLSTLIIRRCVNLTSLPEGIGHLTSLSIFTIEECLNLTSLPEGLLHLSSLRGSLTVSKCPKLSKTWKKLNK*

>Cucsa.128130

MAIGDPQLPILHRAPVDKSHGIKKLSSHALECLGMVCGLNDDLNKLRSNVSSIQSVLRDAEQRQIKGNDHSLTDWLEKLGDVFYDVEDVLDEISTEALRREVMTRGKNAKQVRIFFSNSNQLAFNYRMACQVKKINERLDVISQEKDKFQLNGIAYLGIQNVLSYPIGMERDTHSSLSGDQKIIGRDDEMNNLKKNLLAEDDKVKANVSFIAIVGMGGIGKTTLAKSLYNDKQVSDGFSSRIWIWVSNQFDTKTILKKIIESATEKKPKVEEMEPLKTKLEEVIGGKKYLLVMDDVWNENENEWENLKNLLMLGARGSKVLITKRDSKAVPGVETIPLKDLTEDFSWLLFKEVAFKESDLESINQNLIKMGKEISKRCGGIPLVIRHIGRLLYGKTSAEDWEFIKENELLNVTREKNNNDGHVISTLKLSYNHLSPNLKQCFSYSSLFPKGYKIRMNELIRQWIAQGFIESSNGGKSVENIGKEYLDELCWRFFYEISIEDVPFEEVGMHDLMCDLAREVAGQKLYIRGYPESGYVVSEQTRHISFEYEPRSWIDDVSKLQQAKGLRTFLLFTKNPFFTRNPIEKVLLDRLFSHFPRLRVLQIPNVSKSIKKLRHLRYLELGEDAKSVPNSITKLQNLQTLDLTKCYDLKELPRDINNFVNLRHLLCDSRLMNMLQGTMEKLTSLQTLSSFLFDCKRFDKVKEFSERSYFIEFDLKIKGLEQLRFSPSDVKSVNLKNKKVPLLRLKWKFENGNEYEGDADDIVLEGLEPHPYVNLLQIEGYCGVGLPNWVSTSILLRGIRIGNCDRLHLNQLSHLHALEILNLEGLKSVMSISEWIGTLTSLVSLEIEECPKLKSLPKEMQQLKSLVQLNIIKCPQLGERCKEGGEDWPNISHIPDVLID*

>Cucsa.128140

MAEAILYNVTADIIFKLGSSALQELGLLWGVNDELDKLKHSLSAIQAVLLDAEEQQSKSLAVKAWVSRLKDALYEIDDLVDESSYETLRRQVLAKDQRKRKLVRILFSKFKSNWKIDHKIKDIRQRLQSINDDKNQFSFSEHVIEKRDDEELRKRRETYSYILEEEVIGRNDDKEVVIDLLLNSNITEDIAIVSIVGMGGLGKTALAQSIYTHHNMTNSGFELKLWVCVSEEFDLKVIIQKMIESATGTKPKPYLQIDSLQSELRKKIDGKKYLFVMDDVWNEKKEEWLRLKRLLMGGAKGSRILITTRSEQVAKTFDSTFIHFLQILDEYNSWLLFQKITCLEGHPSNPEKLDQSSSLIQIGREIVSKLKGVPLTIRTIGGLLKDNKSKRVWLSFKDNELHRILGQGQDNLKEVRLILELSYKYLPANLKQCFLYCALFPKDYEIKTHELILMWSAQGFIQPNGSKDNSLIDIGNDYFMELLSRSFFQEVTKNERGDIIACKMHDLMHDLACWIADNECNVINIGTRHFAWKDQYSHKDQLLRSLSKVTNLRTFFMLDSANDLKWEFTKILHDHLQLRALYFKNLKNAMIVLEFTGKLKHLRYLSIMDSFILNLPDSITELYNLETLILRNSSFKMLPDNIGNLINLKHLDLSNNRNLKFLPDSISDLCKLEELILHGCLRLEEFPEDTKKLINLKHLNEKHADALWRDKEKGFNEGLCSLIKAVEKDFHSVHKCKLFFCSVVFLM

>Cucsa.132370

MGDLLTFGVQETLKQAVTLVAKKIIASSEFKVVLEELKDDLLHAEWILHAIKTKHDHSLNDKITHWVNDLQLIVYEAEDMLDLFAYDDVERKIRSNKVFPNSLCTIKPMLDCFSLVVFVHLDNTTRKIESEVEQVEETTSLLENYVVGREMEVESIVQDVTEASQQQLNSILPVYGTGGSGKTTLAQLVFNDERIGKQFHHTVWVCVSQPFVINEILQSILKKVSKSNDNRSKDDKDTLIRNLKEVMGGKRYFLVLDNVWNENKIFWEKLKECLMSIVEELGSSVLVTTRSRKIAEMMKETLDTYHLNKLTDDQCWSVFSYFAKANAVPITSNLELVREELSVDRLPKASIKQCFAYCSNFPKGYWFDKKQVIKMWMAHGFTRPDEGNNETMEDTGERYFNILLSYCLFQDADDDKWHIGRKFRMHDLIHDIACDVSSDKRLQLDHSSSSKWKGLTEEKKKIESKLRTVIDFGRNGKIKDFVCLRVLTIAENVRELPNSISKLKHLRYLDISRCYSIKKLPESIVGHLEILLMGIDLPPKFEMPPYLSELVQLQTLFAFAVGFETGRKISELRGLRNLKGLLKLHRLEHVESKEEAKAAKLVEKEKVEGLNLSWRGKWKNRLEPHKNLKDLKIQSFLGGCFPKETFVENLVTITLHKCGNCEKLPMLGQLSKLEALIIISNFPKVKSIGNEFYGNYNDGQSKSSVVFPKLKEFYVIAMYSLVEWEEVVNNVKAFPRLECLHIVKCTKLTSALKIVFSSLINFDHSISYLNLLPNSLKILLSCCLFQDVEDESEIGQKFLMHDLIHDIACHVSNDEKLPSDHSLLSMRKHWTNDDKIVASKLRTNIVKNENDFEVLEGLEQHNNLKYLEIESFSGGQFPNQIFVENLVKITLIECGNCEKLPMLGQLTKYLEILVIFRLRKVESIGNEFYGNQRRSSSSVFPKLKEFYVDEMDSLVEWEEAVSNYNVKAFPRLECLHIISCKKLLKIPDTKVQICGLLLLQLQEEAPWLICYSNVRSFFLKEDTGPS*

>Cucsa.133510

MAGALIGGAALGVPFNELATLLKNFGERAWSFNSVLNETESKVNDIIPLVKEIDGLNESLDYPREETEKLKNLLEYAGKLLRRCLRVGKADLIRKSSHTEKLRELNARIKSFSDVVLFQTSRDGKKTLSLVTEIKEVVRRLDSKSGLSNPVDLVVTVPVISEESVGLEKPVEKLKAKLFRDGVRLLVVTAPGGCGKSTLAEIFCHDKQVKNKFQRNILFLVVSSKPETKRILISIIQRLGGPIESGSVSDDEAFRLLEVRVGELSPNPVLIVLDDVWDGSESNKLLEKFSRLPNCKVLVTSRFKFPAFGESYDLEPLDHKDAMELFRRWASRGNRVLQFPDERIVEKIVRGCKRFPLALKVIAGSLSGRATSVWEVTGRKLSRGDSILGSEKELQKCLKDTLDAIPDDKIVLKECFMDLGSFPEDQRIRAATFIDICAVLYEQDECETMSNLDELFTRTLVNTVSLRNKAHEDDYYSESYITQHDVLRELAVLLTNEQPVDQRTRLLVDINKNEFPKWWSVRQMQPVKARLLSITTDEKFSSCWPDMEAPEVEVLILNPGSETYKLPDFAKKMNRLKALIVRNYRSFPTELTSDYQLINCLSRLERISLERISISSFIDQNLKPLWHLKKLSFFMCKIDKAFTDCSTQISYMLPNLLEISIDFCNDLVAFPVGLCEVVTLEKLSITNCHALSSLPEEIGQLINLKILRLRSCIHLEKLPESISRLRELVYLDISHCVGLTKLPDKIGNLQKLEKLNMWSCPNMRKLPKSVGNLKNLKEVVCESEMKIWVNFVAPRLGNVVKEHKEEINLDFLN*

>Cucsa.155730

MAISTNHSTLVLGIYGMSGIGKTTLSKALFNHFFHFFNSRSFLPNINSLSTSSPDGLLRLQQTLLSDLLIATNLRSRSSTTTDSTVVRMQERLQNKKVLVVLDDLDRIEQANALAIRDRRWFGDGSRIIITTRNKQILDTLKVDEVYNMESNLLNDEESLELFSYHAFREQNPPEELLECSKSIVSYCGSLPLALEILGGSFFGGRPMEEWRSAMERLKRIPAWDLQEKLRIGFEGLRDEMEREIFLDVCCYFVGMKEELVVKIMDGCGMYGESGLRGLKWRCLVGVEFWSGRLKMHDLVRDMGREIVRQTCVKEPARRSRVWLYHEALKILLHQNGSENIEGLAIDMGKGNNKEKFRLEAFGKMRNLRLLKLNYVHLIGSNFEHIISKELRWICWHGFPLKSIPSSFYQGNLVAIDMRYSSLIHPWTWRDSQILENLKVLNLSHSEKLKKSPNFTKLPNLEQLKLKNCTALSSLHPSIGQLCKLHLINLQNCTNLSSLPTSIYNLHSLQTFIISGCSKIHCLHDDLGHLESLTTLLADRTAISHIPFSIVKLKKLTDLSLCGCNCRSGSGSSASLPWRLVSWALPRPNQTCTALTLPSSLQGLSSLTELSLQNCNLESLPIDIGSLSELKKLNLGGNKNLRVLGTELCGLLKLNELNVENCGRLEFIQEFPKNMRSFCATNCKSLVRTPDVSMFERAPNMILTNCCALLEVCGLDKLECSTNIRMAGCSNLSTDFRMSLLEVFSSSYLLLNYKFGFAVFHLYIVSI*

>Cucsa.163670

MIPHEILSLFITSVYEYLTNIATKLGSLALQDLGLLWTGIHEEIDKLRDTLSAIQAVLHDAEQKQYKSSAVKEWVSRLKDAFYDMDDLMDEFSYESFQRQVMTKHRTNNCTKQVCIFFSKSNQIRFRLKMVHKIKKIREKLDTIDKDKTQFNLFDNTREIRNDEMTKRSETCSFILEGEVIGRDDDKKCIVHFLLDTNIIAKENIVVVAIIGMGGLGKTALAQSIYGDMKENKHFELTMWVCISEEFDVKVIVEKIIESLTKKRPKPNLTLDTLQSMLREKIDGKKYLLVMDDVWNDERTKWINLKKFLMGGAKGSRILITTRTHQVAHIFDTDLFHDLSELDKDNSWELFRKMAFSNESEMLENSKLVGIGKEIVTKLKGSPLAIRVIGSYLYSKKSEKDWLSFKENELDTIMQQENEIQSILKISFNHLSSSLKQCITYCALFPKDFEIDKDDLIKQWMGEGFIQPHNKKAMEDVGDEYFKELLGRSFFQDISKNQLGEIMKFKMHDFMHDLACFVGENDYVFATDDTKFIDKRTRHLSISPFISKTRWEVIKESLIAAKNLRTLNYACHNYDGDEIEIDFSNHLRLRTLNLIFSTHVPKCIGKMKHLRYINFTRCYFDFLPKVVTKLYHLETLIFRECFKLRELPSDITNLINLRHLGINSLIEGLSYMPKGMGSMTTLQTMNLFILGENEGGELSELNGLINLRGSLSIQQLQFCKPIGIENAKHLEEKSGIQKLKLYWYLLERKYEIDDEDEKVLECLKPHPNLQKIVINGYGGVKLCNWFSFDYIVNLVIIDLFNCNKLQQLPRFDQFPFLKHLKLQYLPNVEFIDNNDSVSSSLTTFFPSLEKLRIFRLPKLKEWWKRKLIDQTIPQHRRLESLNISGVSLQVFELVMEMATTNIIVGSQDSSSSTTSISLSFLSIEDIDFEFLQFHDLFSNMTHLKSLWIINCKNIKMSSSLDAVTWKGLGSLRELMLSSIPDLEYLPKSLQCVTTLQSLQIYNCPNLVSIESIRHLTTSLSVLEIHGCPNITFYPHEMSQLASLAITFQNRGWSDNYDPGEGRKEDDDQKQFGRDEQHEGTTH*

>Cucsa.178360

MELCAGAIVNPIAEKIANCTVDPVFRQLDYLLHFKTNVNDLKDQGKKLVETRDFVQHSVDSAKTNGYEIEVMVTEWLGIADQFSEDVDRFFNEADGRSLRWWNMLSRHRFSRRATKLAVAVDKAIQGGSFERVGFRVTPQEIMTLRNNKKFEAFESRVLILKEIIEAVGDANARVIVVHGMAGVGKTTLVEEIARLAKEGKLFDAIAMVTVKHIPNIKKIQGEIADQLGLKFEEEKERIRADRLRRRLEMEKKVLVVLDDVWSRLDLEAVGISSHHKGCKILVTSRKDDLFFNDFGTQKNIYINILSKKEARDFFNKVACDSVESSDDTDPEMEAVATELADECGGLPLSLATVGQALKGKGLPSWNDALQGMKFPGEPSNYGVNKVAYLSLKVSYRSLNREEARSLFLLCSLFPEDYQINIKYLLMYAMGLGLLNAMSSLAMAKWRILSLVDELKTSHLLLDGVDNDFVKMHDIVRDTAILIASKMKSKYLVRHGAGESLWPPMDEFKDYTAISLGCSDHSELPEFICPQLRFLLLVGKRTSLRLPEKFFAGMQELRVLDLTGLCIQRLPPSIDQLVNLQTLCLDDCVLPDMSVVGELKKLEILSLRASDIIALPRVIGELTNLKMLNLSDCSKLKVIPANLLSRLIGLSELYMDNSFKHWNVGQMEGYVNARISELDNLPRLTTLHVHIPNPTILPHAFVFRKLSGYRILIGDRWDWSGNYETSRTLKLKLDSSIQREDAIQALLENIEDLYLDELESVKNILFSLDYKGFPKLKGLRVKNNGEIVTVVNSDNMHHPHSAFPLLESLFLKNLAELGSICRGKLPQMSFRNLKRVKVESCDRLKFVFPSSMVRGLIHLQSLEISECGIIETIVSKNKETEMQINGDKWDENMIEFPELRSLILQHLPALMGFYCHDCITVPSTKVDSRQTVFTIEPSFHPLLSQQVSFPKLETLKLHALNSGKIWQDQLPSSFYGFKNLTSLSVEGCASIKYLMTITVARSLVNLERLELNDCKLMKAIIISEDQDLDNNYPSKSILQNKDVFANLESLLISRMDALETLWVNEAASGSFTKLKKVDIRNCKKLETIFPNYMLNRVTNLERLNVTDCSSLVEIFQVKVPVNNGNQVRDIGANHLKELKLLRLPKLKHIWSSDPHNFLRYPSLQLVHTIHCQSLLNLFPVSIAKDLIQLEVLKIQFCGVEEIVAKRGDDGDGDDAASFLLSGLTSLTLWNLFEFKRFYPGKYTLDCPSLTALDVRHCKSFKLMEGTLENSSSISSAVEKVLHSL*

>Cucsa.178450

MNQASGSSSSSRFRWHFDVFLSFRGEDTRSNFTSHLNMALRQRGINVFIDNKLSRGEEISASLLEAIEGSKISIVIISENYASSRWCLNELVKIIMCNKLRGQVVLPIFYKVDPSEVRKQSGKFGEEFAKLEVRFSSEKMQAWREAMISVSHMSGWPVPKKDDEANLIQRIVQEVWKKLNRGTREMRVPKYPVGIDRQVNNILSQVMSDEIITMVGLYGIGGIGKTTLAKALYNKIADDFEGCCFLINVREASNQYRGLVELQKELLREILMDDSIKVSNLDIGISIIRDRLCSRKILLILDDVDTSEQLEALAGGHDWFGPGSVVIATTRNKHLLAINEFDILQSVQGLNDDEAFELFSWHAFKMSCPSSHYLYLISKRAVSYCKGLPLALEVVGSFLYSIEPSKLKLILDEYENQYLDKGIQDPLRISYDGLEDEVKEIFLYISCCFVGEDINKVKMKLEACGCLCLEKGTTKLMNLSLLTIDKSNRVEMHNLIQHMGRTIHLSKTSTSHKRKRLLIKDDAMDVLNGNKEAKGVKAIKLSFPKATELDIDSRAFEKVKNVVVLEVGNVTSSKGTDLEYLPSSLRWMNWPHFPFPSLPTTYTMENLMELKLPYSSIKHFGRGFMSGERLKEIDLSGSEFLVEIADLSTATNLEKLNLLGCVNLVKVHDSVGSLTKLVTFSLSSNVKGFEQFPPHLKLKSLKLLSMENCRIDEWCPQFSEEMKSSLEELLIQYSTVINQLSPTIGYLTSLKRLFIIECMKLKTLPSTIYRLRNLTFLSITNLDFLETMVHVAPALKLLDLSGNNFCRLPSCIINFKSLKSLVTMECKLLEEIPKVPKGVVRMNATGCISLTRFPDNIPDFICCDDNVVRIIVLSHDLMISRVFRSYKN*

>Cucsa.178620

MAEAILFNLTADIIFKLGSSALRQFGSLRGGVKDDFDKLWHSLSAIQAVLHDAEEKQFKDHAVEVWVSRLKDVLYEIDDLIDEFSYQILRRQVLRSNRKQVRTLFSKFITNWKIGHKIKEISQRLQNINEDKIQFSFCKHVIERRDDDDEGLRKRRETHSFILEDEVIGRNDDKEAVIDLLLNSNTKEDIAIVSIVGMPGFGKTALAQSIYNHKRIMTQFQLKIWVCVSDEFDLKITIQKIIESATGKKPKSFLQMDPLQCELRKQIDGKKYLIVMDDVWNEKKEKWLHLKRLLMGGAKGSRILITTRSEQVAKTFDSTFVHLLQILDASNSWLLFQKMIGLEEHSDNQEVELDQKNSNLIQIGMEIVSTLRGVPLLIRTIGGLLKDNKSERFWLSFKDKELYQVLGRGQDALKEIQLFLELSYKYLPSSNLKQCFLYCALFPKDYRIKKDELILLWRAQGFIQQNGNNDDNSSLVDIGEDYFMELLSRSFFQEVEKNDFGDIITCKMHDLMHDLACSITNNECVRGLKGNVIDKRTHHLSFEKVSHEDQLMGSLSKATHLRTLFSQDVHSRCNLEETFHNIFQLRTLHLNSYGPPKCAKTLEFIKFLPDSITKLYKLEALILDGCSNLKELPKYTKRLINLKRLVLYGCSALTHMPKGLSEMTNLQTLTTFVLGKNIGGELKELEGLTKLRGGLSIKHLESCTSIVDQQMKSKNSKFLQLKSGLQNLELQWKKLKIGDDQLEDVMYESVLDCLQPHSNLKEIRIDGYGGVNLCNWVSSNKSLGCLVTTYLYRCKRLRHLFRLDQFPNLKYLTLQNLPNIEYMIVDNDDSVSSSTIFPYLKKFTISKMPKLVSWCKDSTSTKSPTYWHAPKLKLLQISDSEDELNVVPLKIYENLTFLFLHNLSRVEYLPECWQHYMTSLQLLCLSKCNNLKSLPGWIRNLTSLTNLNISYCEKLAFLPEGIQHVHNLQSIAVVDCPILKEWCKKNRREDWPKIKYYISEHIWENICSLTGSWSSRNKIFSDHFRSVIALNCSACFYLIYICRCYYELISSPFFIILYHVLFNE*

>Cucsa.189390

MDIISPVVGPIVEYTLKPIGRQLSYLFFIRQHIQNLESQVELLKNTKESVVNKVNEAIRNAEKIESGVQSWLTKVDSIIERSETLLKNLSEQGGLCLNLVQRHQLSRKAVKLAEEVVVIKIEGNFDKVSSPVALSEVESSKAKNSDFVDFESRKPTIDKIIAALMDDNVHTIGVYGMGGVGKTMLVQEISKLAMEQKLFDEVITSTVSQTPDLRRIQGQLGDKLGLRFEQETEEGRALKLLNRLKMERQKILIVLDDVWKQIDLEKIGIPSIEDHSGCKILFTSRDNDVLFNDWRTYKNFEIKFLQEDETWNLFRKMAGEIVETSDFKSIAVEIVRECAHLPIAITTIARALRNKPASIWKDALIQLRNPVFVNIREINKKVYSSLKLSYDYLDSEEAKSLFLLCSMFPEDYIIDCQVLHVYAMGMGLLHGVESVAQARNRITKLVDDLISSSLLLKESNVDLVMYVKMHDIVRDVAIIIASKDDRIFTLSYSKGLLDESWDEKKLVGKHTAVCLNVKGLHNLPQKLMLPKVQLLVFCGTLLGEHELPGTFFEEMKGMRVLEIRSMKMPLLSPSLYSLTNLQSLHLFDCELENIDVICELNKLENLSLKGSHIIQIPATISQLTQLKVLDLSECYALKVIPPNILVNLTKLEELYLLNFDGWESEELNQGRRNASISELSYLSQLCALALHIPSEKVMPKELFSRVFPFELNENESSYLKYLYINYNSNFQHFIHGQNKTNLQKVLSNMERLELSYLENLESFFHGDIKDISFNNLKVIKLLSCNKLGSLFLDSNMNGMLLHLERINITDCEKVKTVILMESGNPSDPVEFTNLKRLRLNGLPQLQSFYSKIEQLSPDQEAEKDERSRNFNDGLLFNEQ

>Cucsa.237070

METVIAILGTVCEYAVAPIGRQVGYVSSYKKNINDLKDQLQNLVDTKTRLQHMVNEARSSAYNIQSDVSSWLNQVDKIIEQSNDILYKNENESNSKYCSNKLNFIHQYQMSKKAKKMVKVISQIIEKRKLMFHQVGYPTPLSRIHGSSTSSSHGYDQILESRTSIAKQIRDALVDCNVNKVGVYGMGGVEKTTLLKQVTPLVMEEKLFDHVIIVNVGQTLGVEGIQAQIGDKLRLELNKKVESKEGRASLLQNKLEMESNVLLVLDDLWKGLDLEEVGIPCRSESCEKGCKILITSRDRDVLTNEMDTQVYFEVKPLSEKESWEFFKNMIGEFDNKCIELIGKEMVKKCGGLPIALATIVKTLKGKEVPIWKDALKQLKNPIAVDVKGVTDVFPDDYEISVEDLQIYAMSLRLLNQVNTWDEARNRVIKLVDDLKASSLLLESNSRDNHVKMHDIVRDVAIYIASKEANMSTLSYGFGLSEWQEKDRHGFYRAIFGNCHNFYNFPQNLEFPKLELLILDGHDWRGEKLQICYSFFEGMKELKVLNLSRMCFQLLRRPSIHSLENLQTLCMSHCTFNDIDAISHLKKLQILRIDKCPITLLPKSMSQLTQLKVLQVSNCPLKVIPPNTLSSLLKLQALDIWTSFNGWGEEVSHNNKLINNARLSELKCLPHLTNLKIHILDIKILSDLIFLKNLKLERFVIHVGELKMSQRLQGCEQYATTLMLKIITSSSQIVSIDHHEFLSLEKMESLENIVHADVFTSPFRKLRSIKVISCKRLRYLFSFSIFKGLVDLQRVFIFDCNMMDEILCMDSEDSTIAVEGNSIECPQLKDLTIIGAHNLKMLWHKNGLAPNFFSKLQRISINSCNTLRYRG

>Cucsa.237390

MVPSSPSSSAASSSSPSSSPSIGKWKFDVFLSFRGEDTRGGFTDHLYKALTRKGISTFRDENEIEEGEHIPSNLLASIDASRFAIVVVSEDYASSRWCLEELARMFECKKEVLPIFYKVDPSHVKNQSGTFEEAFVKHEKRFGRGDGKVQSWRTFLTELANTKAWLSQSWSHESNIIEEITTKIWKRLKPNLTVIKEDQLVGINSKINKLSSLLIPNSDDDDADDDVIFVGIHGMGGIGKTTIARVCYERIRDEFEAHCFLSNVRENYIRTLGNLSCLQTKLLSSMFSLKNNHIMDVEEGTAMINKAIFRKKTLLVLDDVDSSDQIKGLIPDNNSFGNGSRVIITTRNADFLSNEFGVKRIFEMDELKYEEALQLLSLSAFMKTCPKEGYLEHSKKIVKVVGGHPLALKLLGSSLRNKNLSVWNEVIEEVGGGGNIHEKIFKCLKVSYDGLDEREREIFLDVACFFNGKRREVVEEILNGCGFYAKTRIELLIQKSLLTLSYDNKLHMHNLLQEMGRKIVRDKHVRDRLMCHKDIKSVVTEALIQSIFFKSSSKNMVEFPILFSRMHQLRLLNFRNVRLKNKLEYSIPSELRYLKWKGYPLEFLPIDSSEECKLIELHMCHSNLKQFWQQEKNLVELKYIKLNSSQKLSKTPNFANIPNLKRLELEDCTSLVNIHPSIFTAEKLIFLSLKDCINLTNLPSHINIKVLEVLILSGCSKVKKVPEFSGNTNRLLQLHLDGTSISNLPSSIASLSHLTILSLANCKMLIDISNAIEMTSLQSLDVSGCSKLGSRKGKGDNVELGEVNVRETTRRRRNDDCNNIFKEIFLWLCNTPATGIFGIPSLAGLYSLTKLNLKDCNLEVIPQGIECMVSLVELDLSGNNFSHLPTSISRLHNLKRLRINQCKKLVHFPKLPPRILFLTSKDCISLKDFIDISKVDNLYIMKEVNLLNCYQMANNKDFHRLIISSMQKMFFRKGTFNIMIPGSEIPDWFTTRKMGSSG*

>Cucsa.237410

MERRASIKSLSPPPYSISLPLPPLRNYDVFLSHRVKDTGSSFAADLHEALTNQGIVVFRDGIDDEDAEQPYVEEKMKAVEESRSSIVVFSENYGSFVCMKEVGKIVTCKELMDQLVLPIFYKIDPGNVRKQEGNFKKYFNDHEANPKIDIEEVENWRYSMNQVGHLSGWHVQDSQSEEGSIINEVVKHIFNKLRPDLFRYDDKLVGISPRLHQINMLLGIGLDDVRFVGIWGMGGIGKTTIARIIYKSVSHLFDGCYFLDNVKEALKKEDIASLQQKLLTGTLMKRNIDIPNADGATLIKRRISNIKALIILDDVNHLSQLQKLAGGLDWFGSGSRVIVTTRDEHLLISHGIERRYNVEVLKIEEGLQLFSQKAFGEEHTKEEYFDVCSQVVDYAGGLPLAIEVLGSSLRNKPMEDWINAVEKLWEVRDKEIIEKLKISYYMLEKSEQKIFLDIACFFKRKSKKQAIEILESFGFPAVLGLEILEEKCLITTPHDKLHMHDLIQEMGQEIVRQNFLNEPEKRTRLWLREDVNLALSRDQGTEAIEGIMMDLDEEGESHLNAKAFSEMTNLRVLKLNNVHLSKEIEYLSDQLRFLNWHGYPLKTLPSNFNPTNLLELELPNSSIHHLWTASK

>Cucsa.237440

MTSLSFPPPPPPPYSISLPLPPLRRYDVFLSHRAKDTGCSFTSNLHEALTSQGIVVFIDKEDGGKPLTEKMKAVDESRSSIVVFTKNYGSLVCMKEIRKIRMCQKLRDQLVLPVFYKIDPGDVRKQEGSFEKYFNEHEVNPNISIEEVKKWRKSMNKVGNLSGWSEEGTINEVVNHIFNKLRPDLFRYDDKLVGISRRLHEINKLMGIGLDDVRLIGIWGMGGIGKTTIARIIYKSVSHLFDGCYFLDNVKETLKKEGIASLQQKLLTGALMKRNIDIPNAEGATLIKRRMSNIKALIILDDVDHLSQLQQLAGGSDWFGSGSRVIVTTREEHLLISHGIKRRYNVEVLKIEEGIQLFSQKAFGEDHPKKGYFDLCSQVVDYAGGLPLAIEVLGSSLRNKPMEDWIDAVKKLWEVRDKEIIEKLKISYYMLEKDDREIFLDIACFFKRKSKRQAIEILESFGFPAVFGLDILKEKSLITTPHEKIQMHDLIQEMGQKIVNEKFPDEPEKRSRLWLREDITRALSHDQGTEAIKGIMMDLDEEGESHLNAKAFFSMTNLRILKLNNVHLSEEIEYLSDQLRFLNWHGYPLKTLPSNFNPTNLLELELPNSSIHHLWTASKVHQNNSSN*

>Cucsa.237520

MCRPPYWCWDDSWMFFDVGYTKFKIQSIANSIGDHLLRLKLQAKEENLFEMPLRLRTMKMLLGLGSNDVRFIGIVGMSGIGKTTLAEMTYLRIFKPFVSALRKPYFLHFVGRSIVSLQQQLLDQLAFLKPIDIQVLDENHGVELIMQHLSSLKNVLIVFDGITERSQLEMLAGSPDWFGAGSRIIITTTNKNIFHHPNFKDKVQEYNVELLSHEAAFSLFCKLAFGDHPHTQNMDDLCNEMIEKVGRLPLALEKIAFSLYGQNIDVWEHTLKNFHQVVYDNIFSDVLKSSYEGLEAESQQIFLDLACFLNGEKVDRVIQILQGFGYTSPQTNLQLLVDRCLIDILDGHIQMHILILCMGQEIVHRELGNCQQTRIWLRDDARRLFHENNELKYIRGIVMDLEEEEELVLKAKAFADMSELRILRINNVQLSEDIECLSNKLTLLNWPGYPSKYLPSTFQPPSLLELHLPGSNVERLWNGTQNFKNLKEIDASDSKFLVETPNFSEAPKLRRLILRNCGRLNKVHSSINSLHRLILLDMEGCVSFRSFSFPVTCKSLKTLVLSNCGLEFFPEFGCVMGYLTELHIDGTSINKLSPSITNLLGLVLLNLRNCIRLSSLPTEICRLSSLKTLILNGCKNLDKIPPCLRYVKHLEELDIGGTSISTIPFLENLRILNCERLKSNIWHSLAGLAAQYLRSLNDLNLSDCNLVDEDIPNDLELFSSLEILDLSSNHFERLSESIKQLINLKVLYLNDCNKLKQVPKLPKSIKYVGGEKSLG

>Cucsa.237530

MGKQTDNKLVLSHKTSLVGMENQVKKVCNLLDLERSKDILFVGIFGSSGIGKTTIAEVVYNTIIDEFQSGCFLYLSSKQNSLVPLQHQILSHLLSKETKIWDEDHGAQLIKHHMSNRKVVIVLDGVDERNQIEKLVGSPNWFAPGSRVIITATNRDVLHQLNYRDQVQEYKVELLSRESAYSLFCKNAFGDGPSDKNDLCSEIVEKVGRLPLALRTIGSYLHNKDLDVWNETLKRLDEEEQNYFDTILKRN

>Cucsa.237540

MQSSSSSSLDRPKMNYDVFISFRGRDVRHTFAGYLYDALNRLGIKAFLDNKRFLIGDDLHDLFKIIDESRSAIVVLSEDYASAKWCLRELTKIMDSMGTSMERVLPVFYHIDPSIVKDQSGTFKTSFDEHEANVLKEIDNQEKEKRLKELQNWKSALKKIGNHTGVVITKNSSEVDIVNKIASQIFDAWRPKLEALNKNLVGMTSRLLHMNMHLGLGLDDVRFVAIVGMGGIGKTTIAQVVFDCILSKFEDCCFLTLPGGDSKQSLVSLQREMLSQIFHKEDFRIWHENHGVEMIKNRLSGRKVLIVLDGIEERRQLEMLAGSIEWFGPGSRIIITTRNKGLLCHPNYDEMKVYNVEELDHDSALQLFLKHAFGSNHQNNDSFMDLSNEIVEKAKRLPLALRVIGSSLYGKDITVWRETLKRLIKVDERNFFDVLKISYDGLGVESQQVFLDITCFFNGKNEDRVIEILESFGYSPNSEVQLLMQRCLIEVSHKKILVHDLILEMGREIVRKESLTQAEKQSRIWLHEDLYCRFAEKHDLMHIQGIVLSLAKEMEESIELDAESFSEMTKLRILEISNVELDEDIEYLSPLLRIINWLGYPSKSLPPTFQSRYLFELLLPHSHLLRIWDGKKRFPKLKLIDVSNSEHLRVTPDFSGVPNLERLVLCNCVRLCEIHPSINSLNKLILLDLEGCGDLKHFPANIRCKNLQTLKLSGTGLEIFPEIGHMEHLTHLHLDGSNITHFHPSIGYLTGLVFLDLSSCLGLSSLPCEIGNLKSLKTLLLKYCKKLDKIPPSLANAESLETLSISETSITHVPPSIIHCLKNLKTLDCEGLSHGIWKSLLPQFNINQTITTGLGCLKALNLMGCKLMDEDIPEDLHCFSSLETLDLSYNNFTTLPDSLSHLKKLKTLNLNCCTELKDLPKLPESLQYERFRSKFDLLLHGDKIPKFFSNQSKGNMTEIKLPQYLEKFRESIGVAVCALVVVDKKRRKLNEIIPERERYTKVVDLICKFKVDSYQIMPEHCHFTSQQKLLSEYASQFLWLSYIPLHGFNINWHYCTQFEIALETSCDELFGVKNCGLHLIHKHERMMIDKMVMESTVPSSTSHKGKEPQIH*

>Cucsa.237560

MASSTPKELSSFSSSPRFIFDVFLSFRGVDTRKNVTNRLYEALRRQGIIVFRDDDELERGKTIANTLTNSINQSRCTIVILSKRYADSKWCLRELVEIVKCKNTFKQLVLVVFYKIKPSDVNSPTGIFEKFFVDFENDVKENFEEVQDWRKAMEVVGGLPPWPVNEQTETEKVQKIVKHACDLLRPDLLSHDENLVGMNLRLKKMNMLMGIGLDDKRFIGIWGMGGIGKTTIAKAVFKSVAREFHGSCILENVKKTLKNVGGLVSLQEKLLSDTLMRGKVQIKDGDGVEMIKKNLGNQKVFVVLDGVDHFSQVKDLAGGEEWFGCGSRIIITTRDEGLLLSLGVDIRYNVESFDDEEALQLFCHEAFGVKFPKKGYLDLCMPFIEYAEGLPLAIKALGHSLHNRLFKSWEGAIRKLNNSLNRQVYENLKISYDALGKEERRIFLYIACFLKGQNKDQVIDTFVSFEIDAADGLLTRKNAADVLCIKETAADALKKLQEKSLITMLYDKIEMHNLHQKLGQEIFHEESSRKGSRLWHREDMNHALRHKQGVEAIETIVLDSKEHGESHLNAKFFSAMTGLKVLRVHNVFLSGVLEYLSNKLRLLSWHGYPFRNLPSDFKPSELLELNLQNSCIENIWRETEKLDKLKVINLSNSKFLLKTPDLSTVPNLERLVLNGCTRLQELHQSVGTLKHLIFLDLKDCKSLKSICSNISLESLKILILSGCSRLENFPEIVGNMKLVKELHLDGTAIRKLHVSIGKLTSLVLLDLRYCKNLRTLPNAIGCLTSIEHLALGGCSKLDKIPDSLGNISCLKKLDVSGTSISHIPFTLRLLKNLEVLNCEGLSRKLCYSLFLLWSTPRNNNSHSFGLWLITCLTNFSSVKVLNFSDCKLVDGDIPDDLSCLSSLHFLDLSRNLFTNLPHSLSQLINLRCLVLDNCSRLRSLPKFPVSLLYVLARDCVSLKEHYNYNKEDRGPMSQAEVRVLSYPSSAKDQNSKISQLMISSMCTACENGG*

>Cucsa.239860

MGGVGKTYLLKEVKKLVLEEKLFDLVIDVTVGQSNDVMNMQQQIGDFLNKELPKSKEGRTSFLRNALVEMKGNILITFDDLWNEFDIINDVGIPLSKEGCKTLVTSRFQNVLANKMNIKECFKVTCLDDEESWKFFKKIIGDEFDAKMENIAKEVAKQCGGLPLALDIIAKTLKRSRHINYYWEGVLSKLKNSIPVNIDVGEKVYASLKLSYEHLDGEEVKSLFLLCSVFPDDHGISVNDLQMYVMGMGLLKMVNTWKEARAEAHYLVEDLTSSSLLQRLKNRDVKMHDIVRDVAIYIGPDFNMSTLYYGYSTSSKGLDEDKCRSYRAIFVDCKKFCNLLPNLKLPKLELLILSFPFWGKDRNIDIMDAYFEGMENLKVLDIEGTSFLQPFWTPLKNLRTLCMSYCWCEDIDTIGHLKQLEILRISNCRGITELPTSMSELKQLKVLVVSHCFKLVVIHTNIISSMTKLEELDIQDCFKEWGEEVRYKNTWIPNAQLSELNCLSHLSILRIVSVNGTKLSILLEGTKRLMILNDSKGFANDIFKAIGNGYPLLKCLEIHDNSETPHLRGNDFTSLKRLVLDRMVMLESIIPRHSPINPFNKLKFIKIGRCEQLRNFFPLSVFKGLSNLRQIEIYECNMMEEIVSIEIEDHITIYTSPLTSLRIERVNKLTSFCSTKSSIQQTIVPLFDERRVSFPELKYLSIGRANNLEMLWHKNGSSFSKLQTIEISDCKELRCVFPSNIATSLVFLDTLKIYGCELLEMIFEIEKQKTSGDTKVVPLRYLSLGFLKNLKYVWDKDVDDVVAFPNLKKVKVGRCPKLKIIFPASFTKYMKEIEELEMVEPFNYEIFPVDEASKLKEVALFQSLETLRMSCKQAVKERFWVMSKFFKLKSLELFGCEDGKMISLPMEMNEVLYSIEELTIRGCLQLVDVIGNDYYIQRCANLKKLKLYNLPKLMYVLKNMNQMTATTFSKLVYLQVGGCNGMINLFSPSVAKNLANLNSIEIYDCGEMRTVVAAKAEEEEENVEIVFSKLTGMEFHNLAGLECFYPGKCTLEFPLLDTLRISKCDDMKIFSYGITNTPTLKNIEIGEHNSLPFTSICGVHTILSEKEGIINLMA*

>Cucsa.248810

MADFLWTFAVEEMLKNVLKVAGEQTGLAWGFQEHLSNLQKWLLNAQAFLRDINTRKLHLHSVSIWVDHLQFLVYQAEDLLDEIVYEHLRQKVQTTEMKVCDFFSLSTDNVLIFRLDMAKKMMTLVQLLEKHYNEAAPLGLVGIETVRPEIDVISQYRETISELEDHKIAGRDVEVESIVKQVIDASNNQRTSILPIVGMGGLGKTTLAKLVFNHELVRQRFDKTVWVCVSEPFIVNKILLDILKNVKGAYISDGRDSKEVLLRELQKEMLGQSYFLVLDDVWNETFFLWDDLKYCLLKITGNSNNSILVTTRSAEVAKIMGTCPSHLLSKLSDDQCWSLFKESANAYGLSMTSNLGIIQKELVKKIGGVPLAARVLGRAVKFEGDVERWEEMLKNVLTTPLQEENFVLSILKLSVDRLPSSSVKQCFAYCSIFPKDFVFEKQELIQMWMAQGFLQPQQGRYNNTAMENVGDIYFNILLSRCLFEFEDANKTRIRDMIGDYETREEYKMHDLVHDIAMETSRSYKDLHLNPSNISKKELQKEMINVAGKLRTIDFIQKIPHNIDQTLFDVEIRNFVCLRVLKISGDKLPKSIGQLKHLRYLEILSYSIELKLPESIVSLHNLQTLKFVYSVIEEFPMNFTNLVSLRHLELGENADKTPPHLSQLTQLQTLSHFVIGFEEGFKITELGPLKNLKRCLCVLCLEKVESKEEAKGADLAGKENLMALHLGWSMNRKDNDLEVLEGLQPNINLQSLRITNFAGRHLPNNIFVENLREIHLSHCNSCEKLPMLGQLNNLKELQICSFEGLQVIDNEFYGNDPNQRRFFPKLEKFEISYMINLEQWKEVITNDESSNVTIFPNLKCLKIWGCPKLLNIPKAFDENNMQHLESLILSCCNKLTKLPDGLQFCSSIEGLTIDKCSNLSINMRNKPKLWYLIIGCVTQIPEQLQHLTALQFLSIQHFRCIEALPEWLGNYVCLQTLNLWNCKKLKKLPSTEAMLRLTKLNKLHDLNICLSPSFSSLLCFLIN*

>Cucsa.249360

NLKRLLRVAKHLVEIDSQLREIEESVSHIGSEGIGKTTLAKDLYNKIATQFERCCFLQDVRREASKQYGLVQLHETLLCEILKEDLKVVNCDKGINIIRSRLCLKKVLIVFDDVDHHRQLEALVGELDWFGRGSKIIMLTRNGHLLSSHGFDEKHKIHELDQDHALVLFSLSESATNYCKGLSVALVVLGSFLRGRDQTRWSCILDEFENSLPKDIKDVLQLSFDGLEDKANDIFLDISLHVIRPLDFGIMILMDLSLIMIESDRVQMHGLIQQMGCSIVRNESSQPEKRSRLWLVQDIGEVFVNKSVRNSLPKLKHVDLSYSTLLEKILDFSAASNLEELYLTNCTNLGMLDKSILSLNKLTVLNFEGCSNLKMLSRGYFMLSSLKELRPSYYKKLEKIPDLSAASNLKRLYLQECTNLRVIHKSVGSLDKLGLLDLSQCTNLVKLPSYLRLKSLYTLYLSGCCKLESFPTIAENMKYLEELYLNFTAI*

>Cucsa.251930

MADFIWTFALQEILKKTLHLATQQIRLASGFNHDLSKLLHSLLFFEAILRDVDRTKSDLQSVKIWVTKLQDLVLDAEVVLDELSYEDLRREVDVNGNSKKRVRDFFSFSNPLMFRLKMARKIRTITQVLNEIKGEASAVGAIPTGGSDEIVADNGHIPETDSFLDEFEVVGRRADISRIVNVVVDNATHERITVIPIVGMGGLGKTTLAKAVFNHELVIAHFDETIWVCVTATFDEKKILRAILESLTNFPSGLDSKDAILRRLQKELEGKRYFLVLDDVWNENVKLWNNFKSLLLKITNSIGNRVLVTTRSEEAGKIMETFPSHHVEKLSDDECWSIFKERASANGLPLTPELEVIKNVLAEQFGGIPLVAKVLGGAVQFKKRTETWLMSTLETLIMNPLQNENDVSSILRLSVDHLPNSSLKQCFAYFSNFPKGFNFEKEQLIQFWMAEGFIQPSDKVNPETMEDIGDKYFNILLARSLFQDIVKDENGKITHCKMHHLLHDLAYSVSKCEALGSNLNGLVDDVPQIRRLSLIGCEQNVTLPPRRSMVKLRSLFLDRDVFGHKILDFKRLRVLNMSLCEIQNLPTSIGRLKHLRYLDVSNNMIKKLPKSIVKLYKLQTLRLGCFRGEAPKKFIKLISLRHFYMNVKRPTTRHMPSYLGRLVDLQSLPFFVVGTKKGFHIEELGYLRNLRGKLKLYNLELVRNKEEAMRADLVKKDKVYKLKLVWSEKRENNNNHDISVLEGLQPHINLQYLTVEAFMGELFPNLTFVENLVQISLKNCSRCRRIPTFGHLPNLKVLEISGLHNLKCIGTEFYGNEYGEGSLFPKLKRFHLSDMNNLGRWEEAAVPTEVAVFPCLEELKILDCPRLEIAPDYFSTLRTLEIDDVNNPISQITLQTFKLLGIIHSGNLSGLPEELRGNLSSLEEFKVWYYLHLKSFPTIQWLTDILKGKTGYDTKWTNIQSHGLESYTSVNELSIVGHSDLTSTPDIKALYNLSSLTISGLKKLPKGFHCLTCLKSLSIGGFMEGFDFRPLLHLKSLENLAMIDFILAESTLPDELQHLTGLKHLKIVGFQGIESLPEWLGNLNSLESLHIESCRKLRELPEAMGCLAKLEEVRSFNCPELRVYQDESEWAKISYIPRFISFNYWVDEDQQRIQFKVCHKPS*

>Cucsa.275630

QSSSSCSSNLKWSYDVFLSFRGEDTRNNFTSHLDRALREKGVNFFIDDKLERGGQISESLLKSIDGSKISIIIFSKNYASSTWCLDELVKIVQCMKSMGHIVFPVFYKVDPSEVRKQTGGFGEALAKHEANELMTNKVQPWKEALTTAASLSGWDLATRKNEADLIHDLVKEVLSILNQTQLLHVAKHPVGIDSQLRAVEELASHDVPDGVNMVGIHGMGGIGKTTLAKALYNKIAYQFEACCFLSNVRETLEQFKDLVQLQEKLLSEILKDNAWKVGNVHKGKNIIRDRLCSKKVLIILDDVDKDEQLDALVGERDWFGRGSKIIATTRDRHLLENHSFDIVYPIQLLDPKKSLELFSLHAFKQNHPSSNYVDLSKFAVSYCKGLPLALVILGSLLHKRERKIWKSKLHELENSLEPSVEAVFQIGFKELHERVKEIFLDISCFFVGEDINYSKDVLKACDLNPDYGIIILMDLSLVTVEDGKIQMHDLIQQMGQTIVRHESFEPAKRSRLWEAEGAIKILKEKSVSDFRQCFYLLIAKDIYSEAFRNMKNLRLLILQRVAYFPRNIFEYLPNSLKWIEWSTFYVNQSSSISFSETPNFFATLNLEKLYLRGCTSLKGMRPSFRKFPSHLKFKSLKVLNLRDCLNLEEITDFSMASNLEILDLNTCFSLRIIHESIGSLDKLITLQLDLCHNLEKLPSSLKLKSLDSLSFTNCYKLEQLPEFDENMKSLRVMNLNGTAIRVLPSSIGYLIGLENLNLNDCANLTALPNEIHWLKSLEELHLRGCSKLDMFPPRSSLNFSQESSYFKLTVLDLKNCNISNSDFLETLSNVCTSLEKLNLSGNKFSCLPSLQNFKSLRFLELRNCKFLQNIIKLPHHLARVNASGSELLAIRPDCIADMIYLHANDRHHIKVLFPNTTSKFVSKRFKSANVIMRTQGVYMVDRRFSCYT*

>Cucsa.277260

MAEFLWTFAVQEVLKKIVNFGAEQISLAWGLEKELSHLKKWLLKAQTILADINTKKSHHHSVGLWVEELHDIIYEADDLLDEIVYEQIRQTVEQTGKLRKVRDSISPSKNSFLFGLKMAKKMKKITKTLYEHYCEASPLGLVGDESPTESEAALNQIRETTSILDFEVEGREAEVLEILKLVIDSTDEDHISVISIVGMGGLGKTTLAKMVFNHDAIKGHFDKTVWVCVSKPFIVMKILEAIFQGLTNTSSGLNSREALLNRLREEMQGKKYFLVLDDVWDKENCLWDELIGNLKYIAGKSGNSIMVTTRSVEVATMVKTVPIYHLKKLSDDHCWALLKKSANANQLQMNSKLENTKNILVRKIGGVPLIAKVLGGAVKFEEGGSESWMAKIESFARNISIEDKDFVLSILKLSVESLPHSALKQCFAYCSNFPQDYEFDKDEAIQMWIAEGFIQPEQERENLTMENIGEEYLNFLLSRSLFEDAIKYDGRIVTFKIHDLMHDIACAISNHHKMDSNPISWNGKSTRKLRTLICENEEAFHKIQTDIICLRVLVLKWFDTNTLSTIMAKLIHLRYLDISNCNINKLLRDSICALYNLQTLKLGYIECDLPKNLRNLVNLRHLEFKKFFDMGQMPSHMGNMIHLQTLSEFVVGLEKGCKIDELGPLKDLKGTLTLKNLQNVQNKDEAMAAKLVEKKYLRHLIFQWFLNLYDRGEYDEDDNKQVLEGLQPHKNVQSLDIRGFQGRVLNNNIFVENLVEIRLVDCGRCEVLPMLGQLPNLKKLEIISMNSVRSIGSEFYGVDCNDRNSSAFPQLNKFHICGLKKLQQWDEATVFASNRFGCLKELILSGCHQLAKLPSGLEGCYSIEYLAIDGCPNLMLNVQNLYNLYHLDIRGLKRLPDEFGKLTNLKKLRIGGCMQNYEFSPFIHLSSQLVELELTDDGSSGSETTQLPQQLQHLTNLKVLKIADFDDIEVLPEWLGNLTCLATLVFLECKNLKELPSREAIQRLTKLDDLVIDGCPKLLLGEGDQERAKLSHLPSKCGLGLKSVMINLLPKKSF*

>Cucsa.292710

MVGIHGMGGIGKTTLAKALYNKITYQFEACCFLSNVRETSEQFNGLVQLQEKLLNEIFKDNNLKVDNVDKGMNIIKDRLCSRKVLMVLDDVDKDDQLDALVGGRDWFGRGSKIIVTTRDRHLLETYSFDKIHPIQLLDCDKSLELFCWHAFKQSHPSRNYSELPELVRYCNGLPLALVILGSLLCKRDQIIWKSKLDELKNFPEPGIEAVFQISFKRLPENPPVKEIFLDICCFFVGEDVSYSKNVLKACDPYLESRIIILMDLSLVTVEDGKIQMHDLIRQMGQMIVRRKSFKPEKRSRLWVAKEAVKMLIEKSGTHKVKAIKLDLRNNGSLIVEAEAFRNMENLRLLILQNAAKLPTNIFKYLPNIKWIEYSSSSVRWYFPISFVVNGGLVGLVINGVSNKHPGIIFEDCKMLKHVDLSYWRLLEETPDFSAALNLEKLYLLSCKRLKMIHGSVASLSKLVTLDLEGCENLEKLPSSFLMLKSLEVLNLSGCIKLKEIPDLSASSNLKELHLRECYHLRIIHDSAVGRFLDKLVILDLEGCKILERLPRYISNSKSIEVMNLDSCRKIEQLFDNYFEKFPSHLKFESLKVLNLSYCQNLKEITDFSIASNLEIFDLRGCFSLRTIHKSVGSLDQLIALKLDFCHQLEELPSCLRLKSLDSLSLTNCYKIEQLPEFDENMKSLREMNLKGTAIRKLPTSIRYLIGLENLILSYCTNLISLPSEIHLLKSLKELDLRECSRLDMLPSGSSLNFPQRSLCSNLTILDLQNCNISNSDFLENLSNFCTTLKELNLSGNKFCCLPSLKNFTSLRLLELRNCKFLRNIVKIPHCLKRMDASGCELLVISPDYIADMMFRNQRDPEILQQSNHRKFNKF*

>Cucsa.318890

LVFNHELVRQHFDKTVWVCVSEPFIVNKILLDILQNLKGTISNGGDSKEVLLRELQKKMHGQRYFLVLDDVWNENSFLWDELKYCLLKITGNSKNSIVVTTRSAEVAKIMGTCSGHLLSKLSDDHCWSLFKESANAYGLSMTSNLEIIQKELVKKIGGIPLAARVLGRAVKFEGDVERWEEMLKNVLSTPLKEENFILSILKLSVDRLPSSALKQCFSYCSIFPKDFVFEKQELIHMWMAQGFLQPQEGRNMTMETVGDIYFKILLSHCLFEDAHETKTEEYEIPDLLEFETRPEEYKMHDLVHDIAIEISRDQNLQLNPSNISKKELQKEIKKVACKLRMVDFIRRIPCNIGQLTFFDVEIRNFVCLRVLKLSTLPSDKLPKSIGQLKHLRYLEIACYLGRLKFPESIVSLHNLQTLKFLYSYVEKFPMNFTNLTLSHFVIGFEEGCKITELGPLKNLQGCLSLLCLEKVESKEEANGTNLAEKEKLKDLHLSWSNERKDNNNYNDLEVLEGLQPNQNLQSLGIYNFAERRLPNKIFVENLSVIGLYGCNNCEKLPMLGQLNNLKKLEIYSFHGVQIIDNEFYGNDLNQRRFFPKLEIFVMCDMINLEQWKEVMTNDASSNVTIFSNLKCLEIRGCPKLTKLPNGLHFCSSIRRHLLSLKKITLVEDELSNNSVTQISEQLQHLTALEFLSIENFGGIEALPEWLGNFVCLQTLSLYNCKNLKKLPSTKAMLRLTKLNQLYACKCPMLLLEEGDPERAKLSHFPNMLVQRNGYLKCI*

>Cucsa.326910

KIPILHQLDYLFHYKQNIKEVEKKVEALGTAKGNAVFDGVSKWLTIVKDVLEIAQQNENPSCFNFVERYQLSRKAKKRVENIIELINEGNGFNKDNVGYPVPSPDTNSPTLPTDYQIIASRTSIVEEIKEALANPNVDTVGVCGMGGVGKTALLNEVKKLVLEKNLFDRVIQVEVGESKSVFNIQEQIKDELNMELNIECEEVRACRLRTHIAERKENMLFMLDDIWKEHDVEKEFGIPFKHVLNNEMNTEKTFEVNSLTNEESRNFFVTIVGESSCVEDGHNIQQIAEDVVKECGGLPLALKILGKALKGKRVQIWKDALKSLKNPVTVTISGVSEQLYSCLQFSYDSTEDEAEQVLLLCSVFPDDYKIEVKDLQMYAMGMGLVKHINTWEDAGNRVIKLVDDLKSCYLLQDEQSKKGSDDCVQMHDVVHDFAKYVASKKDKMTSLTYRSGQRLEYWQEEDDDMHESYKAIYADCAKYCVYLPPKVGVSEPSIGNKIRIPTAFFERMKALRVLSVETMSISFEPSSWASINNLEALYELIKLKVLHVLKCDDFNPSEFPPNIIESMTQLEELKFDGFKMNELSELNRLTRLFSLELRIQNVEILLNELSVEKAEKLEEFSFCVDSVGFTNLLCNNNHTVPYGNYNWYPRLKELQIYIHNNQNQYLDMPRGIENNPCILIFSCNKLTYVFPSHMLTLLVFLNTLEVHHCKLVERIFEIEEWSGSGGAGDVNQVLVPFTILHLSFLPNLKHVWNTDPNPTTSDLLPQCKESGDYKVSDA*

>Cucsa.328080

MAESILCSLAGSIITKLGSFALQDLGLLWGFHDELDKLKGTVSALEAVLLDAEEKQSKSRAVKDWILKLKDTFYDIDDLLDVFSYESLKRQVMTKHRTNNTKKVRIFFSKSNQIAFRLKMSQKIKRVREKLDAIAMDKTQFNLYENTREIQDDESTKRLETTSFIREGEIIGRDDDKKSIIHYLLDTNIHEDSVAVIAIIGMGGLGKTALVQSIYGDEKVKKHFELTMWVCISEEFDVKVIIEKIIESLTKKKREPDLQLDTLQSMVREKIDGKRYLLVMDDVWNVNRAKWISLKRYLMGGAKGSRILITTRTHQVAQTFETILSHHLKELDEEKSWKLFRKMAFSNESEVLENSKLVVIGKEIVTKLKGSPLAIRVIGSYLYSKKSEKDWLSFKDHELDTIMQQENEIQSILKISFNHLSSSLKHCFTYCALFSKDYHYEIRKNDLIKQWMAQGFIQPHNKKAMEDVGDDYFEELLGRSFFQDIRKNKWGEIKKFKMHDIIHDLACSVVENDCVLANDDTKSIDKRTRHVSISAFNSMTRWKLITKSLIEAKNLRTLNYARRHHIDLSNHLRLRTLNLEFHFVPKCIGKMKHLRYINITYCYIDFLPKAVTKLYHLETLIIRGCLELRELSSDIKNLINLRHLDIKDFKHVWSYMPKGMGSMTTLQTMNLFILGENKGGELSELNGLVNLRGSLSIQQLQFCKPIGLENVKYLEEKSRIQKLELHWKTYQRESKIDDEDERVLESLKPHSNLQKIRIEGYRGLKLCNWFSFDSIVNLVFIKLFNCEKLQQLPRFDRFPFLKHLHLEDLPSIEYIAINNYVSSSMTTFFPSLENLSIIKLPNLKEWWKGESIDQNTSFPTILRHLSQLKIHYCRQLASIPQHGPLQSLDIRDISLQLFELVIKMTATNIIFLPNDLFSNVTHLQSLVIGRCFNLKMSFDDDNVRWKELGSLRTLRLCFIPKLEYLPKGFQYLKALEHLELLWCENLACILGIEHLTSLSRLEISNCPNLTSLPEGMTQLISLTCLIIDDCPNLSTLPEGLHHLLNTPRYAPLIFSH*

>Cucsa.337180

MACCIYEQAENILIELKKFPMYLRRMQYTMLSLKTILKDAEKEEYRHCLNDWLQKLQSVFLQIEELLYESNREVKKQEATGKWVFLPSFNFSQIDQTKKMMKLCDDLDEIASHMYGFNLTNMETTHSFLSATEVSTRLMKPSWQLLYSLTNAPKVFQDKRYHNFLDHFKKSTHGLFHIVGEPGIGKTTLAKFFYNNLVNTFPSRLWICVKEEFDPQRLIKEMLSFSHCQVTCDNLTEKQLCFAVQQFLRDKKFLIVFQDISIKNLGNCSIFKSLLGMGNRGSKIIVTTQNEKIADAVGLKKLYKNESQVVPSPEATKPSDVNKDNMKHQTIFKVERLSKENSLSLFKVHAFTETQEAQIPNLTKIQEVIEQKCHGVPLAIKCLGGLLSKTSIAEWNGVIDKLWEHEEEEDGNKSILPTLRLCYDQMPSHLQRCFLYCSQLKKDRILSSNDVIQLWIASDLLPKENYLSLEKIGENYFKELCSRCFLQELEEYGFGYWFKLHPLIEKLARLLTQKQVFEVTKTQSIAFTIRDKVPPSAFLANACIDKFKYLRLLHLGNANLQGIPSAVENLVQLRYLDLQGNKKIKRLPNSIFKLKNLQTLILASCSALKELPNDIRQLTNLRYLWVTANNLRLHKNGVGTMTSLRFLAIGGCKKLTLTLKGVEFRLQRFTIRELPIVKKLPEWTQRFTETLRVLEIIDCPIEWNDDVLKSYKSLERFSIHGAVRTKNQIGGYNIDYRNFVRSRKVKKEVKTCVYY*

>Cucsa.337190

MAYCIYYRAENILSELKNLPNYPRRIEYTMLSLKSILMDAEEKQEQSRGLQNWLEELQNVFSQIEGFIDEHKEEAYEGIGKQVLAPFSCSSNQIARTWKMEKLFDHLNEVAAKMYEFNLTERHTGAIKTETTNSFLTATEVSTRLMKPSWKVLYPLTNAPKFYQDERYRKILNDFKNPTLGFFHIVGEAGIGKSTLAKFIYNDPEVEGMFPSRLWVCVKEEFDTQRLMKEILNFSYSPATCDNLTTKLCPTDQYLRERTFLLVFQDLSIKNLDNCSLFTSLLMMGKPGSKIIVTTQNEEIANAIELTMIYKVGQQSEQNRSQTALDTVTKETANVNNADQFVQANPLGKIDQSIPSQTIFKVKRLSEKDSLSLFKDYASTYEGNEKDIMKTLKKCNGIPLAIKCLGSMLSLGPPATKWMEDNERQKGDNESSSTFSILKLCYNEMPSHLKRCFLYCSQLPNDSILSSNDVIQLWMANGLLRSRQENYLSLEDIGEIYFKELCSRCFLQDVEEYGLGYWFKMHPLIRELARLVQKRTKDLISIKPVTNVTSIAFPVRDEVPSSSFLAEKCISKFQHLRLLYLGHTDLQEIPNTIETLNHLTYLDLQGNKNIKRLPNAICNLQHLQTLILASCSALEELPKDICKLSNLRYLWVTSNKLRLHKNGVGTMTSLRFLAIGGCDKLQDLFERPSCLVRLETLMIYDCNSLQLLPNEMGSLISLQNLVIWSCKQLTLKGLEKVDFSLQRFTIRELPEVNKLPEWLQRSTETLRVLEIIDCPIKVEEEGIKIHHWEFGTA*

>Cucsa.338110

MAKKIKNITDTLNQHYCAASAFGLVGVETVTEIELALNQIRETTSILDFQVEGREAEVLELLKLAIDSTNEHHMSVISIVGMGGLGKTTLAKMIFNHREIEGHFDKTIWVCVSKPFIVTKILEKIFQGLTKTCSGLESNKEALLGRLRKEMQDKNYFLVLDDVWDNEKHLWDELRGCLKHIAGKPGNTIVMTTRNEEVATMVEPISIYRLKKLSNDQCWALFKESANANQLPMNSKLEIMKKELVKKMGGVPLVAKVLGGAVKFEETELEEEDHEISWMTKVESIVRNISLEDKDFVLSILKLSVDSLPNPVLKQCVAYCSNFSQDYDFQKDDLIKMWIAQGFIQPGQGRDKNLLMEDIGEQYFNFLLSRSIFQDVTRDANKRIVGFKMHDLMHDIACAISSHQNVESNPNNLSGKSVRKLRTLICNDEVINYLNQKDIVCLRVLKVIFQSHTDLWIPIDKLIHLRYLDISECSINKLLLESLSLLYNLQTLKLGQSGLPKNLRKLVNLRHLEFKMFGDTAMPSDMGNLIHLQSLSGFLVGFEKGCKIEELGPLKNLKGKLTLTNLWRVQNKDEAMAAKLVEKKNLRHLNLWFFETDKRGEDDEDGIVQVLEGLQPHKNLQSLEILGFRGKVLPTGIFVENLVKIRLGHFERCEVLPMLGQLPNLKELEIMYMESVRSIGNEFYGVDSSHQNSVAFPQLKKVSIYEMMNLEQWDEATVVLASNLFGCLKEVRIRRCNPLAKLPSGLEGCHSLEYLSIRGCFNLMLNVQNLHKLYHLEIDGLKRLPKGMDGLTRLKELKIGGCMQNYEFSSVIHLASQLVELELSGRYGSVDTQLPQQLQHLTNLQVLKITQFDCIEALPEWIGNLISLKTLKCSYCFKLKELPSREAILRLTKLENLDIFECPKLLVGEGDQERAKLSHLPSKCVHKSE*

>Cucsa.338190

MVGLLDSVAGNLLGRIIEAADRLEFRAIQSELKNLETDVLNLKARLRDAEEKQASNCELNELLKNLKNVFSRADIAIEELECDYLKWRVQNRKNDVDDKGCQFSSCFSSNFLISPFNTGSKFQEDLKIITSELRSIEKAMSKFSLVEDEDEYIKKLKGEMTLRTSITGSHAFARLLRLRREAILSNVDSIFGRDKIQESIIKELVNDEQKSPRILSIQGDGGMGKTALAKLVYNADEVFDHFDKRMWVCVSEDFDIRRILREVLMSATGENVTTVALTESRLRIRLQRYFFGKKILLVLDDFGNLDPERVSELKKIVKMGVGGSKIMITTRSDETLNVATTHKIDKLDETISMQIFEDTYGSEGLSEGLRDDLYLKNLVAECGGAPLAIKCLAGLLSSKPSDGAKSPNVKDLSEKWKQEEANNGGGVLCALRLSYDLMPSYLKPCFLCFSVLPKDNVFFSFELIQLWMAQGILPSGTKDNPEEVGEKYFKEFRDRRLLVDVEEHTLGYWFKIHSLVHDLAVQKATEQKNLGNFHMLSFVDCDSIPSSTNYDNTRFISIPVVGGAGPNINSDLFKCITQFRQLRFLYLCNSSLEEIPTSIDTLKHLRCLDLRGSQRLKRLPESICKLQSLQTLVLAFCSELEELPRNIKNLISLRFLWIQTKQARLEKDEIGSLTSLRFLAIGRSENLTHLFEDINKLNSLKTLIIYECKSLLTLPKGLENMKSICNMGIWECDRLRFTFSLASLHLKKLILRELTAVSTLPNWLSNLDGTLEVLEIGEFPTLRKLPIWLLNFWELRILGISNCPKLKHDSFPPELNYFCDKIEELRITFCGSLSKSLLKKSMKEIEPESRVIFYIHTIYVDSKRMTPPVESTDEPKEAETKQDDAYNNASPPGTEQPSKTKHDDANNNMSHPGIGLLSESKQEHTNNNINEIETVKVCLGDNDHAEAHQAM

>Cucsa.338650

MADELRPQHGNWTYDVFLSFRGEDTRKNFTDHLYYAFKDAGINVFRDDPELERGEDISSELERAIEGSKVAVVVFSERYAESGWCLEELVKIMECRRTLRQLVFPIFYNVDPSCVRKQKGEFEEAFVKHEVRYFRDIDRVLKWRMALTEAANLSGWDLRNIANGHEAKFIRLIVEKVSKEVNSKYLFIALYPVGIESRLKLLLSHLHIGSNDVRFVGILGMGGLGKTTVAKALYNQLYHNFEAKCFLSNIKAETSNLIHLQKQLLSSITNSTNINLGNIDQGIAVLQERLRCKRLLLILDDVDDLSQLTALATSRDLFASGSRIIITTRDRHLLNQLEVDEICSIDEMDDDEALELFSWHAFRNSYPSETFHQLSKQVVTYCGGLPLALEVLGSFLFGRSREEWEDTLKKLKKIPNDQIQKKLKISFDGLNDHTYKDIFLDVSCFFIGMERNYVEQILDGCGFFPRIGISVLLQRCLLTIGDKNRLMMHDLLRDMGREIVRENFPKYPERHSRLFLHEEVLSVLTRQKGTDATEGLSLKLPRFSKQKLSTKAFNEMQKLRLLQLNFVDVNGDFKHISEEIRWVCWHGFPLKFLPKEFHMDKLVAMDLRYSQIRFFWKESKFLKNLKFLNLGHSHYLTHTPNFSKLPNLEILSLKDCKNLIELHPTIGELKALISLNLKDCKSLNSLPNSFSNLKSLQTLIISGCSKLNSLPEDLGEITSLITLIADNTPIQKIPNTIINLKNLKYLSLCGCKGSPSKSSFSSMIWSWISPKKLSQNYTSILLPSSLQGLNSLRKLCLKNCNLSNNTIPKDIGSLSSLRELDLSENLFHSLPSTISGLLKLETLLLDNCPELQLIPNLPPHLSSLYASNCTSLERTSDLSNVKKMGSLSMSNCPKLMEIPGLDKLLDSIRVIHMEGCSNMSNSFKDTILQGWTVSGFGGVCLPGKEVPDWFAYKDEGHSIFLELPQYNNSNLEGFIVCIVYCSCFNNTVSTDLPSLSVINYTKSSITTNKPLTNDVIMSTQDHLWQGHLSNKAFKMEPGDEVEIIVDFGAEITVKKIGISLVFDKYVDQTMLEFASTCNDDDVVVDNQDETVSEKDGEVGSKRGFDENDDEGLKNSYQIPKRLKCEIDSNMKIDEE*

>Cucsa.338660

MANEFQAQHGDWTYDVFLSFRGEDTRKNFTDHLYYALKDAGINVFRDDPELQRGEDISSGLERAIEGSKVAVIVFSERYAESGWCLEELVKIMECRRTLRQMVLPVFYNVDPSCVRKQKGEFEEAFVKHEKGKDIDKVRRWRMALTEAANVAGLGLTQNANGHEAEFIRSIVKMISKEVKSNYLFIALYPVGIESRIKLVLPHLHIGSNDDVKFVGILGIGGLGKTTIAKALYNQLHHNFEAACFLANIKQTPNQPNGLVHLQKQLLSSITNSSNINFENMDRGIVVLQESLRRKKLLLILDDVDKISQLTALATRRECFGSGSRIVITTRHRRLLNQIEVDGICSIDVMDDAEALQLFSWHAFHNSYPSETFHQLSKRVVNYCGGLPLALQVLGCFLFGRSREEWQDTLKNLKKILDDQIQIKPKITFDTHNDHTCKDIYLVNQMLDGWGSFPRIGDINRLVTSDLLRDHTQLFLPKEVRLSVLGPKVRK*

Coding DNA sequences of the NBS-encoding genes from the 9930 cucumber (Cucumis sativus L.)

>Csa001236

ATGGCTGAGTTCTTATGGACTTTTGCTGTGCAAGAAATTTTGAAGAAGGTCCTAACTCTTGTGGCTGAGCAGATCATTCTAGCAAGGGAGGTCAAGGATGTGCTACAACAACTACAAAAAGAGCTAGTTGAGTCTCAAAAAATTGTTAGTGCTATCACTACCCAAAGACAAAATCATTATTCACCAGATAGCTTAGTGACTCAGTGGGTGAATGATCTTCAACTTATTGTTCATGAGGCTGACGACTTGCTGGATTTGTTTGTTTATGAACATCTTCAGCAAAGAGTGAACCCATCAGCTCATGGAAAAATAATAAAAAAGGTACCTCATTTCTTATGCGCTTCGGCAAGGACTAAAAAAATGAAGGAAATTATAGCGTTGTTAAACAAACATTGCACCAAATTACCTCACTTACTTCAACTTGAGCCTACACCCTCAAACATTGCAGAGACTGAAGTTGCGCAAATTCAAGAGACAGTCTCAAAGCCTGAAGATTATGTGGTGGGAAGGAACAGGGAAGTTGAAACCATAGTTGATCGAGTGATTGATGCCAGCAAACAGGAACTCAATTCTATTTTACCCGTTTTTGGAATGGGTGGATTAGGAAAAACCACTTTGGCAAAGTCGGTCTTCAACCATGATAGGATCAAAAATCATTTTGGTATCACTATTTGGATATATGTGTCACAACCTTTTGTCATCAACAACATTTTGCAAGCAATCTTACAAAAGGTGGAGGTTCATTCTAGTGATTGCTCCAACAATAGGGAGGCCTTACTTGAAAAGCTTACAGAAAACATGGGAGAGAAAACATATTTTCTTGTTCTTGACGATGTTTGGAATGAAAACAAAATGTTGTGGGAGAAGTTGAAGGAATGTTTGATGAGTATTACTCATATGTCAGGAAATAGTATTCTTGTCACTACAAGGAGCAGTGGAATTGCAAAAATGATGGAAGAAAATATTGGAAGTCATGAATTAAGAAAATTATCTGATGACCAATGTTGGTCAATATTTAGGAACTTTGCCAATGCCAAGGATGTACCAATGACTTCCAATTTGGAGTTTGTGCAAAAAGAGTTTGATAAAAGAATCGGTGGTCTTCCATTAATCGCTAAAGTTTTGGGAGCAGCAGTTCCATTTTCAGGAGACCATGACCAGTGGGTAGCAAATATAAAAAGCGTTCTAACAACTCCAATAAAAGAGGAAGAGTTTGTTAAATTCACATTGAAGTTAAGCGTTGATCGTCTACCAAATGCTTCAGTAAAGCAGTGTTTTGCTTATTGTTCAAATTTTTCCAAGGGTTGTGAGTTTGACAAAAAGCAAGTGATTCGAATGTGGATGGCACAAGGATTTACTCAACCAGATGAAAGAAACAATGAAACAATGGAAGATACAGGAGAAAGGTACTTTAACATCTTGTTGTCTTTCTGCTTATTTCAAGATGTTGTTAAGAATGAAAGAGGAATAATTGAGAAGGTTCGAATGCATGATCTTATACATGATATTGCTTGTCAAGTTTCAAATGATAAAAAGTTGCGAATAGATCACATCATTTCATCAAATTGGAAAGATTGGACGAAAGATGACAAAATACTTGTGAGCAAGTTACGAACAATAAATTTTTATGATCGTCATCATGTGGTGGTTCAGGATAAGATTGGGGACTTTACTGGTTTGCGTGTTTTGACAATTGAAAATTATATTGTTGAGGAGTTACCAAACTCAATATTCAAGTTGAAGCACTTGAGATATCTAGACATTTCGTATTGTTATTCAATAAAGAAGCTTCCTGAATCTATTGTTCTGCTTTATAATTTGCAAACACTGAGATTTCATCTCTTAAGCAAGGGATTTCTACCAAAAAACGTTGGACAAATGATTAGTTTGAGGCATTTGGAGTTCTCATCTATCGATAAACAAATGTCTCCCTATTTGAGTCAATTGATTCAACTTGAAACATTGCCTAAATTTGCAGTAGGTTTTGAGAAGGGTTGTAAGATTACAGAACTTGGGGTTCTAAGAAACCTGAAAGGGTTGTTGAAGCTTCAACGTTTAGAACATGTTGAAAGTAAAGAAGAAGCCGAAACTGCAAAATTAGTGGAAAAGGAGAATCTAGAAGAAGTACATTTTGTGTGGACAAAGGAAAGGAAGAGAAAAGTAGAGAATAAGAATGATTTGGAAGTGTTGGAAGGACTTCAACCACCCAAAAATGTAGAATATTTGAGAATCAAATACTTTTTAGGTGGGTGTTTACCAAACCAGACGTTTGTTGAGAATTTAGTGAAAATAGAGCTAAGAGATTGTGGAAATTGTGAGAAGCTTCCAAGGCTTGGGCAATTAGGAAATCTAGAGATACTTGATATTTCATGGTTTGAAAGAGTAAAGAGTATAGGGAATGAATTCTATGGAAACAGCTCCAACAACCAAAGGAGTTTATTCCCCAGGTTGAAGGAATTGTATGTTGATGAGATGAGGAGGATAGGAGAATGGGAAGAAGTGGGAAGTAATGTTAAAGCTTTCCCACGTCTTGAACGTTTGTATATTGGTTGTTGTAGAGATTTAGTGAAAATTCCAGATGTTTTTGGGTATTGTGATGAGTATGGTGAGAAGCATCTGGAAGTTGTGGAAATTATTGAACATTTGTGGTTAGACCGACCTTCAAATTTGTGGTCTTTTGTTACAACGCAAGGAGGAGCATTGGCCAATTTGCTATCCAGAAGAACTCGCTCTTTCTTCTATTACCGAAAAGAAAGAAGACAAATGAAAGAAAAAATAAAAGAAAAATTGCGGTTTGCTCTTTATGTGCAGTCTGCTGCGACCCAATTCATCGTCAAAGTCCCAACACCAACACCAACACCAAAGCCTAAAATTCGTTCTTTTTCTCCTTGCTTCTTCTAA

>Csa001480

ATGGCAGAATCAATTCTGTTCAGCCTTGCAGCAAATATTGCAACCAAATTGGGTTCTTTAGCACTCCAAGACCTTGGATTGCTGTGGACCGGTATCCATGAGGAGATTGACAAACTCAGAGACACTCTTTCCGCCATCCAAGCAGTACTTCACGACGCAGAACAGAAGCAGTACAAGAGTTCTGCTGTGAAGGAATGGGTTTCAAGGCTAAAAGATGCTTTCTATGATATGGATGATTTGATGGATGAGTTCTCCTATGAATCCTTTCAAAGACAGGTTATGACCAAACATAGAACCAACAACTGTACCAAACAAGTATGTATTTTCTTCTCAAAATCTAATCAAATTAGATTTCGTTTGAAAATGGTTCATAAAATAAAAAAGATCAGGGAGAAACTCGATACTATTGATAAGGATAAAACTCAATTCAATCTTTTTGATAATACAAGGGAGATACGAAATGATGAAATGACACAACGATCAGAGACTTGCTCTTTTATACTTGAAGGAGAAGTAATTGGTCGAGATGATGACAAGAAATGTATTGTACATTTTCTATTGGATACCAACATTATTGCAAAGGAAAATATTGTTGTGGTTGCCATTATTGGAATGGGAGGATTAGGAAAGACTGCCCTTGCTCAATCTATCTACGGCCATATGAAGGAAAATAAACATTTTGAATTGACAATGTGGGTGTGTATTTCTGAAGAATTTGATGTCAAAGTAATTGTTGAAAAGATCATAGAATCTCTCACAAAAAAGAGACCTGAGCCCAACCTTACACTCGATACCTTACAAAGTATGCTACGAGAGAAAATTGATGGAAAAAAATACTTGCTTGTCATGGATGATGTGTGGAACGATGAACGGACGAAATGGATTAATCTAAAAAAATTTCTTATGGGTGGAGCTAAGGGAAGTAGGATTTTGATCACAACTCGTACCCATCAAGTTGCACATATTTTTGACACAGATTTGTTCCATGATTTAAGTGAACTAGACAAGGACAAATCAGAGATGCTTGAGAATTCAAAGTTGGTCGGGATCGGTAAGGAGATTGTGGCAAAGTTGAAAGGTTCTCCTCTTGCAATAAGAGTAATTGGAAGCTATCTGTATTCTAAAAAGTCAGAAAAGGATTGGTTGTCATTCAAGGAGAACGAACTTGACACAATCATGCAACAGAAAAATGAGATTCAATCCATACTAAAGATCAGTTTTAAAAAGGTATTCTTGAAGGTAGCACCTATGAAGGGTGTTATGCGTTTTGAGAAGAAAGGAAAGTTAAGTCCTCGATTTGTTGGACCATTTGAGATCTTAGAGAGGGTTGGTGTTGTGGCGTATCGCTTGGCGTTACCACCACCACTCTCTGCAGTCCATAATGTTTTCCATGTTTCGATGTTGAGGAAATATGTGGTCGATACATCTCATGTAGTAGACTATGAACCCTTGGAGATTGATGAGCATTTGAGCTATGTAGAACAACCTGTGGAGATTCTGGCTAGAGAGGTGAAGATGCTTCGTAATAGAAGCATTCCATTAGTAAAGGTTTTGTGGCGGAATCATCGAATTGAAGAGGCGACATGGGAGCGAGAGGAAGAGATGAGGACTCGATATCCAAAGTTATTTCAGGATTAA

>Csa001907

ATGGCCGAAGCAATTCTCTTCCAAGTTGCTGGGGAGATCTTGATGAAGCTAAGCTCTCAAGCTTTCCAGCGTCTTGGGATGCTATTTGGGCTGAAGGGTGATCTTAACAAACTCACAACAACTGTTTCCACCATTAAGGATGTGCTTCTTGATGCGGAGGGACGTCAAACTAAAAGTCACTTGCTGCAAAATTGGCTCCATAAGCTGGAAGAAGCTCTTTATGATGCAGAGGATGTGCTTGATGAACTCTCTACGGAGGCTCTCCGTCGAGAACTGATGACTAGAGATCATAAAAATGCAAAACAAGTAAGGATCTTCTTCTCCAAATCTAATCAAATTGCATTTAATTACAGGATGGCTCGTCAAATAAAGAATATTTGGGAGAGGCTAGATGCTATTGATGCTGAAAAAACACAATTTCACTTGCGTGAAAACTGTGAATCACGGACTCAATACGGTTCATTTGATCGAATAATGATGGGAAGGGAAACTTGGTCTTCTTCAAATGACGAGGAAGTGATTGGAAGGGATGATGATATAAAAGAAGTAAAAGAGCGTTTATTGGATATGAATATGAATGTCACGCATAATGTTTCGTTCATTGCTATAGCTGGAATGGGTGGGATAGGCAAGACGACCTTGGCTAAATCTCTCTACAATGACGAAGAGGTATCAGGATTTTTCGACTTAAAAATATGGGTTTGGGTTTCTGATCAATTTGAGGTACAAGTGGTAGCGGAAAAAATGATAGAATCAGCAACCAAAAACAATCCTAGTGTAAAAGGAATGGAAGCTTTACAAGCAAAGCTTCAGAAAGTGATTGGAGAAAGGAAGTATCTGTTAGTTATGGATGATGTATGGAATGAAAGTGAAGAGAAATGGCATGGGTTGAAATCATTGTTGATGGGTGGTGCAAGAGGGAGTAAGGTTTTGATCACAAAGCGTGACAGAAAAGTAGCCACAGAAATCAAAAGCATGACATCTTTGTTCACTTTAGAAGGCTTATCAGAGAGTAATTCCTGGTTATTGTTTAGTAAAGTGGCATTTAAAGAAGGCAAAGAGTCCACAGATCCAAGCACGATACATTTAGGAAAAGAAATTTTAGTGAGATGTGGAGGTGTTCCTCTTGTTATAAGACATGTTGGACGCATGTTATACTCTAAAACTTCACAAGAAGAGTGGATGTCCTTCAAGGATAATGAACTTTTAGAAGTCATTCAACAAGACAATGATATGACATCAATATTAAAATTGAGTTATAACCATCTCCCACCAAATTTGAAACGATGTTTTGCATATTCATCCCTGTTTCCCAAAGGATATAAAATAGAAATAAAAGACCTAATAAGGCAATGGGTGGCTCAAGGTTTTATTGAAGTGTCAAATGGAAGAAAATCCTTGGAAGATACAGGGAAGGACTATTTTAACGAATTATGTTGGAGGTTTTTTTATGCAAATTCTAGTGATGAGTGTAACATCAATGATATTGTTTGTATGCATGATGTGATGTGTGAGTTTGTAAGGAAGGTGGCAGGAAATAAATTATATGTACGTGGAAATCCCAATAATGATTATGTTGTCAGCGAACAAACACTTCACATTTCATTTGACTACGGAATACAATCATGGCAAGATGTTCTATCTAAATTATGCAAGGCTAAGGGATTAAGAACAATCCTTTTATTATTTCGTCCCTACGAGAAAATGAATAAAATTGATAAAGCTATTTTGGATGAATTATTTTCCAGTTTTCCACGTTTGCGAGTATTAGATCTTCATTTCTCGCAGATTTCTGTAGTGCCGAAGTCTATAAAAAAACTTAGACACCTTCGATATTTGGATCTCTCTGAAAATGATATGGAATTAATTCCACATTCTATCATTGAATTGCAAAATTTGCAAACACTAAATCTAACAGAATGCTATGAGCTAAAAGAATTGCCAAGGGACATCGACAATCTTGTAAATCTCAGGCATCTTACCTTTGAACCTTGTATGGAAGTAACTCCTACATCGGAGGGGATGGAGAAGTTGACTTGTCTACAAACAATCAGTTTATTTGTGTTTGACTGCAAAAAGACCAATAAGCTATGGGAATTGAATGATCTCAGTTATTTGACAGGAGAGTTAAAAATCATAGGTTTAGAGAAGTTGAGGTCTTCTCCATCTGAAATCACCTTAATAAACCTGAAAGACAAAAAAGGTTGGCAAGGTTTAAATTTGGAATGGAAACTGGGCAAGGATGAATACGAAGGTGAGGCTGATGAAACAATAATGGAAGGCTTGGAACCACATCCAAATGTTGAATCGTTGAGCATTAACGGGTACACTGGAGGAGCATTGCCCAATTGGGTGTTCAACTCGCTTATGAAGTTAACTGAAATTGAAATTGAAAATTGCCCTAGAGTGCAACATCTACCTCAGTTCAACCAGCTTCAGGATCTCAGAGCTCTACATTTAGTGGGCTTAAGATCTCTCGAGTTCATAGATAAGAGTGATCCATACTCATCATCAGTGTTTTTTCCATCTCTCAAGTTTCTACGTTTAGAAGATATGCCTAATTTGGAAGGATGGTGGGAATTAGGGGAATCAAAAGTAGTAGCAAGGGAGACATCTGGGAAAGCTAAATGGTTGCCTCCAACTTTTCCTCAAGTTAATTTCCTACGTATCTACGGATGTCCGAAGTTAAGTTCCATGCCCAAGCTAGCTTCTATTGGAGCAGATGTTATTTTACATGATATTGGGGTTCAGATGGTGAGTACCATAGGTCCAGTATCGAGTTTTATGTTTCTATCAATGCATGGAATGACGAATCTCAAATATTTATGGGAGGAATTTCAGCAAGATCTAGTTTCTTCAAGTACCTCAACAATGTCCTCACCTATTTCCCTTCGTTATCTGACAATAAGTGGATGCCCCTATCTCATGAGTTTACCGGAATGGATTGGCGTTCTCACTTCCCTTGAAACATTGCATATTAAAGAATGTCCAAAATTAAAATCACTACCAGAAGGAATGCAGCAACTCAAATCTTTGAAAGAACTTCACATAGAAGACTGCCCTGAACTAGAGGACAGATGCAAGCAGGGAGGAGAGGATTGGCCAAACATTTCCCACGTTCCCAACTTTACTTACAAAAATGCCTCTGACATTGACACACCACAATCTTCTTCAGGTTTTTCACACCATCCCTTTTCAATCGTTCGTATCTCTGTTATATAG

>Csa002354

ATGGCCAACGAGTTCCAAGCTCAACATGGAGACTGGACGTACGATGTTTTCTTAAGTTTTAGAGGCGAAGATACTCGAAAAAACTTCACAGATCATCTCTACTACGCATTGAAAGATGCAGGCATCAATGTCTTTCGAGACGACCCAGAGCTCCAACGAGGCGAAGACATAAGTTCGGGGCTGGAGCGAGCAATCGAAGGATCGAAGGTGGCAGTTATCGTATTCTCGGAAAGGTATGCGGAGTCGGGATGGTGTTTGGAGGAGTTGGTAAAGATCATGGAGTGTAGAAGGACTTTGAGACAAATGGTTTTGCCAGTATTTTATAATGTGGATCCTTCATGTGTGAGGAAGCAAAAGGGTGAATTTGAAGAGGCTTTTGTTAAGCATGAAAAGGGTAAGGATATTGATAAAGTTCGTAGGTGGAGAATGGCTCTCACTGAAGCTGCTAATGTAGCTGGTTTGGGTTTGACACAAAATGCAAATGGGTATGAGGCAGAATTCATAAGGTCAATTGTTAAAATGATATCAAAGGAGGTGAAGAGCAATTACTTATTCATAGCTCTCTATCCAGTGGGAATTGAATCAAGAATCAAACTTGTTTTACCACATCTTCATATTGGTTCAAATGATGATGTTAAGTTTGTAGGAATTTTGGGGATTGGAGGTTTGGGAAAAACCACCATTGCAAAAGCACTTTACAATCAACTTCATCACAACTTTGAAGCTGCATGCTTCCTTGCTAATATCAAACAAACCCCCAACCAACCCAATGGTCTAGTTCACTTACAAAAACAACTCCTCTCTTCGATTACAAATTCCAGTAACATCAATTTCGAAAACATGGATCGAGGAATCGTTGTGTTGCAAGAAAGCCTTCGTCGCAAAAAGCTTCTTTTGATATTAGACGATGTAGACAAAATAAGCCAATTAACTGCATTAGCAACAAGACGTGAATGTTTCGGTTCAGGTAGTAGAATTGTCATAACAACTCGACATCGACGTTTACTAAACCAGATTGAAGTAGATGGAATTTGTTCCATTGATGTAATGGATGACGCTGAAGCGCTCCAACTCTTTAGTTGGCATGCCTTTCACAATAGTTATCCTTCAGAAACTTTTCATCAACTTTCAAAACGTGTTGTTAATTATTGTGGAGGATTGCCATTAGCCCTTCAAGTGTTGGGCTGTTTCCTTTTTGGCAGAAGTAGAGAAGAATGGCAAGATACATTGAAGAATTTGAAGAAAATTCTAGATGATCAAATTCAAATAAAGCCTAAAATAACCTTTGATACCCACAATGATCACACTTGTAAAGATATATATCTTGTGAACCAAATGTTAGATGGGTGGGGATCTTTTCCAAGAATTGGTGACATAAACAGATTAGTGACAAGTGATTTGTTAAGAGATCATACCCAACTTTTTCTTCCGAAGGAAGTGCGCCTTTCTGTACTTGGACCAAAGGTCTCAGGTGATCAAAGAACCATGCCCCAAACTGAATACCTTGGTATCTCATTCCCAGAAGACTCGCATTCACAGACACAATTGCTTTTGGGTGCTCCCCATGACAAGAGACACAGCAATAACAGTGACACACAAAAAGCTGGGCTTATTGAATTGACACTTTAG

>Csa002355

ATGGCCGACGAGCTCCGACCTCAACACGGGAATTGGACTTACGATGTTTTCTTGAGTTTTAGAGGTGAAGATACTCGCAAGAACTTCACTGATCATCTCTACTACGCATTCAAAGATGCAGGCATCAATGTGTTTCGAGACGATCCAGAGCTCGAACGGGGTGAAGACATAAGTTCGGAGCTCGAGCGAGCGATCGAAGGGTCGAAGGTGGCAGTTGTCGTATTCTCGGAAAGGTATGCGGAGTCGGGATGGTGTTTGGAGGAGTTGGTAAAGATCATGGAGTGCAGGAGGACTTTGAGACAACTGGTTTTCCCAATATTTTATAATGTGGATCCTTCATGTGTGAGGAAGCAAAAGGGTGAATTTGAAGAGGCTTTTGTTAAACATGAAGTGCGTTATTTTAGGGATATTGATAGAGTTCTTAAGTGGAGAATGGCTCTCACTGAAGCTGCTAATTTATCTGGTTGGGATTTGAGAAACATTGCAAATGGACATGAAGCGAAGTTCATAAGGTTGATTGTTGAAAAGGTATCAAAGGAGGTGAACAGTAAATACTTATTCATAGCTCTTTATCCAGTGGGAATTGAATCAAGACTCAAACTTCTTTTATCACATCTTCATATTGGTTCAAATGATGTTAGATTTGTAGGAATTTTGGGGATGGGAGGACTGGGTAAAACCACCGTTGCAAAAGCACTTTACAACCAGCTTTATCACAACTTTGAAGCCAAATGTTTCCTTTCCAATATCAAAGCTGAAACCTCCAATCTAATTCACTTACAAAAACAACTCCTCTCTTCCATCACAAATTCTACCAACATCAATCTTGGAAACATCGACCAAGGAATCGCAGTGTTGCAAGAAAGACTTCGTTGCAAAAGGCTTCTTCTGATATTAGACGATGTAGACGACTTAAGCCAGTTAACTGCATTAGCAACAACTCGTGATTTGTTTGCTTCAGGTAGTAGAATTATCATAACAACTCGAGATCGACATCTGCTAAATCAGCTTGAAGTAGACGAAATTTGTTCCATCGATGAAATGGATGACGATGAAGCACTTGAACTCTTTAGTTGGCATGCTTTTCGCAATAGTTATCCATCAGAAACCTTTCATCAACTTTCGAAACAAGTGATCACTTATTGTGGAGGATTGCCATTAGCTCTCGAAGTGTTGGGTTCTTTCCTTTTTGGTAGAAGTAGAGAAGAATGGGAAGATACACTGAAGAAATTGAAGAAAATCCCAAACGATCAAATTCAAAAAAAGCTTAAAATAAGCTTTGATGGGCTAAACGATCATACTTACAAAGATATATTTCTCGACGTGTCATGTTTCTTTATTGGAATGGAAAGAAACTACGTTGAACAAATATTAGATGGGTGTGGATTTTTTCCAAGAATCGGAATTAGTGTTCTTCTTCAAAGATGTCTATTAACAATTGGAGACAAAAACAGATTAATGATGCATGATTTGTTAAGAGATATGGGGAGAGAAATTGTTCGTGAAAATTTTCCAAAATACCCTGAGAGACATTCAAGACTTTTTCTTCATGAGGAAGTGCTTTCTGTTCTTACAAGACAAAAGGGAACTGATGCAACTGAAGGCCTAAGTTTGAAGTTGCCAAGATTTAGCAAGCAGAAGTTGAGCACAAAAGCATTTAATGAAATGCAAAAATTGAGGTTACTTCAACTTAATTTTGTTGATGTAAATGGAGATTTCAAGCATATTTCTGAAGAGATAAGATGGGTTTGTTGGCACGGATTTCCTTTGAAGTTTTTGCCTAAAGAATTTCATATGGACAAATTGGTTGCTATGGACTTGAGATATAGCCAAATCAGATTCTTTTGGAAGGAGTCTAAGTTTCTCAAGAATTTGAAGTTTCTTAATCTAGGCCATTCTCATTACTTAACCCACACTCCAAACTTCTCCAAACTCCCCAATCTAGAGATACTCAGCCTCAAAGACTGCAAGAATTTGATTGAATTGCACCCTACAATTGGAGAATTAAAAGCCCTCATTTCCCTAAACTTAAAAGATTGCAAATCCCTCAATTCACTTCCAAATAGTTTCTCAAACTTAAAATCCTTACAAACTCTCATTATTTCAGGTTGTTCAAAGCTCAATAGTTTGCCAGAAGATTTAGGCGAAATTACATCATTAATAACTCTAATAGCTGATAACACACCAATCCAAAAAATCCCTAACACAATTATAAACTTAAAAAACCTCAAATATTTATCTTTATGTGGGTGCAAAGGGTCACCATCAAAATCATCATTCTCTTCAATGATTTGGTCTTGGATTTCACCAAAGAAATTATCTCAAAACTACACATCAATTCTTCTCCCTTCTTCATTACAAGGCTTAAACTCCTTAAGAAAATTATGCCTTAAAAATTGTAACTTGTCAAATAACACAATTCCAAAAGATATCGGGAGTTTGAGTTCTTTGAGAGAATTGGATTTGAGTGAGAATTTATTCCACAGTTTGCCATCAACTATCAGTGGCCTTTTGAAACTTGAGACACTTTTGTTGGATAATTGCCCTGAACTTCAATTTATACCAAATTTGCCACCACATTTGAGTTCATTGTATGCATCAAACTGTACTTCATTGGAAAGGACTTCAGATTTGTCTAATGTGAAGAAAATGGGATCTTTGTCTATGAGTAATTGTCCTAAACTTATGGAGATTCCTGGCTTGGACAAATTATTGGATTCTATTAGAGTTATTCACATGGAAGGATGTAGCAACATGTCCAATTCCTTCAAGGATACCATTCTACAGGGATGGACAGTTAGTGGATTTGGAGGAGTATGTCTTCCAGGCAAAGAAGTTCCAGATTGGTTTGCATACAAAGATGAAGTCTCAACTGACCTTCCAAGTTTATCAGTCATTAATTACACAAAATCTTCCATTACAACCAACAAACCTCTTACCAATGATGTAATAATGTCAACTCAAGATCACTTGTGGCAAGGCCATTTATCTAACAAAGCCTTCAAGATGGAACCTGGCGATGAAGTCGAGATCATCGTTGATTTCGGTGCTGAAATCACCGTGAAGAAAATTGGCATCTCGCTTGTGTTTGACAAGTATGTCGATCAAACAATGTTAGAGTTTGCATCCACCTCTAATGATGATGATGTCGTCGTGGATAACCAAGATGAAAATGTAAGTGAAAAGGATGGAGAAGTTGGGAGCAAGAGAGGTTTTGACGAGAATGATGATGAAGGATTGAAAAATTCATACCAAATTCCCAAAAGGTTGAAGTGTGAGATTGATTCTAACATGAAAATTGATGAGGAGTAG

>Csa002384

ATGGCTGAAATTGGAACTTTTGTTGTTCAGGAAGTTTTGAAGAGGATTGTAAAATATGGAGCAGAGCAAATTGTTGTGGCATGGGAGTTGGAGAATGAGGTGTCCCTGTTGAAAGATAAGTTACACGATGCTGATACAATCTTAGAAGACATCAACAGAAAGAAATCACACCCTGGTAATTCTGTGAAAAGATGGGTGGAAAAACTTGAAGATATTGTCCATGAAGCCGATGATCTACTGGATGAGCTTGTTTATGAACATCTTCGACGAACAGTGGAGCATACTGAGAAATTTAGCAAGGTAAGTGATTCAATCTCATCATCCATAAATTCTTTTTTGTTTCGTCGCAAGATGGCCAAGAAAATCAAGAACATTACCGATACTCTAAATCAACATTATTGTGCGGCAAGTGCTTTTGGGCTAGTTGGTGTGGAAACTGTCACAGAAATAGAGCTTGCGCTCAATCAGATTCGAGAGACAACCTCAATTCTTGACTTCCAAGTCGAAGGAAGGGAGGCTGAAGTTTTGGAGCTACTTAAATTGGCGATTGACTCTACCAATGAACATCATATGTCTGTGATATCCATCGTTGGAATGGGTGGTCTTGGCAAAACAACTTTGGCCAAGATGATCTTCAATCATCGTGAAATTGAAGGACATTTTGATAAAACTATATGGGGTTTAACAAAAACTTGTAGTGGGTTGGAATCCAATAAGGAGGCCTTGCTTGGGAGGCTGCGAAAGGAGATGCAAGACAAGAATTATTTTCTTGTGCTTGATGATGTTTGGGATAATGAGAAACACTTGTGGGACGAGCTTAGAGGCTGTTTGAAACATATTGCTGGAAAACCTGGAAATACTATTATGGTGACCACAAGGAATGAAGAAGTAGCGACGATGGTGGAGCCAATTTCTATTTATCGTCTAAAAAAGTTATCCAATGATCAATGTTGGGCGTTGTTTAAAGAAAGTGCAAATGCAAATCAGTTGCCAATGAATTCGAAGTTGGAGATTATGAAAAAGGAGCTGGTTAGAAAAATGGGTGGTGTACCACTCGTGGCAAAAGTTTTAGGAGGTGCAGTCAAATTTGAAGAAACTGAACTTGAAGAGGAAGATCATGAGATCAGTTGGATGACAAAAGTTGAAAGCATTGTAAGGAACATTTCATTAGAGGACAAAGATTTTGTTTTGTCCATATTAAAATTAAGTGTGGATTCTTTACCAAATCCCGTGTTAAAGCAATGTGTTGCCTATTGCTCAAATTTTTCCCAAGATTATGACTTTCAGAAAGATGACCTAATTAAAATGTGGATAGCACAAGGATTTATCCAACCCGGACAAGGAAGAGATAAGAACTTGCTAATGGAGGATATTGGAGAACAATACTTCAACTTCTTATTGTCTCGTTCCATATTTCAAGATGTCACTAGGGATGCGAATAAGAGAATTGTTGGGTTTAAGATGCATGATCTAATGCATGATATTGCTTGTGCAATTTCGAGTCATCAAAATGTGGAATCAAATCCAAATAATTTGAGTGGAAAAAGTGTAAGAAAGTTACGCACGTTGATTTGCAATGATGAAGTGATTAATTATTTGAATCAGAACGACATTGTTTGTTTACGTGTTTTAAAGGTTATTTTTCAATCGCATACGGATTTGTGGATTCCAATAGACAAGTTGATTCATTTGAGATATCTTGATATTTCAGAATGTTCTATAAACAAGCTTCTTCTTGAATCCCTTTCTCTTCTTTATAATCTACAAACGCTAAAGCTTGGACAAAGTGGTCTACCGAAGAATTTGAGAAAATTGGTTAACTTAAGACATTTAGAATTTAAAATGTTTGGTGATACAGCAATGCCTTCAGATATGGGCAACTTGATTCATCTTCAATCATTGTCTGGGTTTTTAGTTGGGTTCGAGAAGGGTTGTAAAATAGAAGAGCTTGGACCGTTGAAAAACCTGAAAGGTAAACTAACTCTTACAAATCTCTGGAGAGTGCAAAATAAAGATGAAGCTATGGCTGCAAAATTGGTGGAAAAGAAGAACTTACGTCATCTAAACCTATGGTTTTTCGAAACTGATAAGAGAGGAGAAGATGATGAAGATGGTATAGTACAAGTGTTGGAAGGACTTCAACCACACAAAAACCTACAATCATTGGAAATCCTTGGTTTTCGAGGAAAAGTTTTGCCTACTGGTATTTTTGTTGAAAATTTAGTAAAGATACGTTTGGGTCATTTTGAAAGATGTGAAGTGCTTCCCATGCTTGGACAGTTGCCCAATTTAAAGGAACTTGAGATTATGTACATGGAAAGTGTGAGAAGTATAGGGAATGAGTTCTATGGAGTTGACTCCAGCCACCAAAATTCTGTTGCTTTTCCACAGTTAAAGAAACTCAGCATTTATGAGATGATGAACCTAGAGCAATGGGATGAAGCAACGGTGGTTCTTGAATCAAATCTCTTTGGATGTCTAAAAGAAGTTAGGATTAGGAGATGTAATCCATTGGCAAAGTTGCCAAGTGGGTTAGAAGGTTGCCATTCCCTTGAATATTTGAGCATCCGTGGTTGTTTTAATTTGATGCTAAATGTGCAAAATTTGCACAAATTATACCATTTAGAGATTGATGGGTTGAAAAGATTGCCAAAGGGAATGGACGGACTCACTCGCTTGAAAGAGTTGAAAATTGGAGGATGCATGCAAAATTATGAGTTTAGTTCCGTCATACACTTGGCTTCTCAGCTTGTTGAACTTGAGTTGTCTGGCCGTTATGGGTCAGTTGACACCCAACTTCCCCAACAACTTCAACACCTCACTAACTTGCAAGTATTAAAGATTACACAGTTTGATTGCATTGAAGCTCTGCCAGAATGGATTGGAAACCTCATCTCTTTGAAAACATTGAAATGCTCCTATTGCTTTAAGTTGAAAGAATTACCTTCGAGAGAGGCCATATTACGCCTAACCAAATTAGAAAATTTGGACATTTTTGAATGTCCAAAGCTACTAGTTGGGGAAGGTGACCAGGAGAGGGCTAAGCTTTCCCATCTTCCATCAAAATGTGTTCATAAATCTGAGTAA

>Csa002436

ATGGCTCATTGCATCTACACCCAAGCTGAAAATATCTTGACTAAGCTAAAAGATTCCCCACCCTACCAAAAAAGAATAGAGTATGCGATGTCGAGCCTTAAAGCGGTTCTTTTGGATGCTGAAGAGAAGCAAGAACAAAATCAACGTCTACAGAACTGGCTAAAGGAACTTCAAAATGTCTTTTACCAAGTTGAGGACTCCATAGATGAATTCAAATGGGAAATCTTCAAACAAAAGGACATTGGAAAACAGGTACTTGCCCCTTTCTCGTGCTCTAATCAAATTTCTGCAAATAAATTGAAACAGAAATGTAAAAGGAAGCAAGTATGCGACGAACTCAATAACATTGCAACCAGAATGTATGAATTTCATCTCAAAGTAAAGCACATTGATTCCATAAGTATGGAGACAACACACACTTTCCCTAGTGCTTCAGAAATTTCAATAAGACACCTGAAACCAAGTTGGCAATTGCTTTACCCTTTAATAGATGCTTCCAGAACATATGACGAAATATATGATGGAATTTTGAATGTTTTCAATGAATGTACTCATGTATTCCACATAGTTGGGGAAGCCGGTATAGGTAAGAGCACAGTGGCCAGATTCTTGTACAATCATAACAATGTAGTTGGTAAGTATACTTCAAGATACTGGGTTTGTGTGGAGGAAGGCTTTAATAAACATAGATTGGTGAAGGAGGTTTACAGTCATGCAGACAATAAAGAAATTTGCGAGGACTTGACGACAGAACAATTGCTTTCTAAGGTTAAACGACTTCTGAAAGTGGAAACTTTTTTGCTCGTCTTTCAAGACCTTTCAATCACCAACTTGAAAGATCGTTCCCCTTTAGTGTTAAATGAATTATTGGAGATGGGACAACATGGCAGCAAGATCATAGTGACCACACAAACTGAGGAAATTGCAAATTATTTACAAGACCGTGGCTACAAGACTGAGAGACGATCAAAGGAAAATCTGGCGAATGGTGACAGAGTATCTGCACACAATCAGTCGTTAACAAAAAATACCCAGAACCAAGCAGTTCCAGGTTCACAAATTGAGGAAACTGCAGACGCTATAGCAAACAAAATCTATGAACACAGCCTATCATTAACAGAAGATGCTCAGAATCTAGAAGTTCCAATTCCACAAGTTACAGAAGATACTAATATTGGCTTGGGGATCAATCCAGATATCCCACCGATAAAGCAAGATGATACGGAATATCAAACAATTTTCAAACTTGAGAAACTGTCAAAGCAAAGTTCATCTCGTTTATTCAAAGAATATGCTTTCAGAAACAGACAAGAAGTAGAAAATCCAGAACTCACTAAAATAGTTGATCAACTTTTGGAGAAATGCATGGGAGTTCCTTTGGCAATAAAGTGTCTAGGAAGCTTGCTATCTTCAGAAACTAGCATAGCCAAGTGGAAAAACATCGAGGAAAAGTTGTCGCTCCAAGAGAAAAAGGAAAACGGTATTTTACATGTACTCAGAGTTTGCTATGATCAAATGCCCTCACAACTGAAGCCTTGTTTTCTGCATTGTTCTCAATTACCTAACGATCGCATAATTTCTTCAAATGATATGATTCAGTTATGGATGGCAAATGGGCTCCTCCATTCACCTGAAGAGAAGAACTCAACTATGGAAAATATAGGTGAGAAGTACTTCATGGAGCTATGGTCAAGATATTTCATTCAAGAAATTGAAGAACATGGGCTTGGCTACTGGATTAAATTGCACCCTCTCATCCAAAAACTTGCACACAAAATCACACAAGAACAATCTGAGGGCTCGGGGGGCAACAATCATCCCAAAGAAGTCACTGAAATAAGATCCATAGCCTTTCAAGAAAGAAATATGGTGCTACCTAATGCATCCCTAACTGAAAAGTGCATCTGGAAGTACAAAGGGTTAAGATTGTTGTATTTAAGCAATGCAGACCTACAGGAAATTCCAAATTCCATAGGAACACTCAAGTACCTGAGATACCTCGACTTGCATGGCAATACGAAAATCAAGCATCTACCAAATTCAATATGTAATCTACAAAGTTTGCAAACCTTAATTCTTGGATCTTGTTCCGCACTTGAAGACCTGCCCAAGGATATAAGGAATTTGATCAGCCTGAGATACTTGTGGGTAACAACAAACAAGCTTCGTCTGGACAAAAACGGAGTTGGAACCATGAATTCTCTGCGGTTTCTTGCCATTGGAGGGTGCAATAACCTAGAAAATCTATTTGAACGCCCAGATTGCCTTGCAGGCCTCGAAACCCTAATGATATATAATTGCACTACCTTGAAATTGTTGCCAGACGAGATGAGATATCTAAAATCACTACAAAATTTGATGATTTGGAGTTGCAAGCAACTTACACTAAACTTAAAAGAAGTGGAATTCAAGCTTCAAAGATTCACGATCAAAGAGCTTCCAAGAGTGGAAAGATTACCCCAATGGCTTGAAAACTCGGCAGAAACTTTGAGAACCTTGCAGATCATCAATTGTCCCATAAGAATAATGGAACGACAGGGAATTGAAAAGTACGAAGCAGTTGAAAATACCATAATCTATGGTGCTGTAAGGTTTGAAATGGCACCACCAGGTTACGACTTCGAACACCGGAATTTGGCAGTGCGTAATGGGAATGAGGAGATGCATATATATCCTTAG

>Csa002437

ATGGCTTATTGCATCTATTACCGAGCTGAAAACATCTTGAGTGAACTGAAAAACCTCCCAAACTACCCAAGAAGAATTGAGTATACCATGTTGAGTCTTAAATCAATTCTTATGGATGCGGAAGAGAAGCAAGAACAGAGTCGTGGTCTACAGAATTGGCTAGAGGAGCTTCAAAATGTATTTTCCCAAATTGAAGGCTTCATAGATGAACACAAAGAGGAAGCCTACGAGGGTATTGGTAAACAGGTACTTGCTCCTTTCTCGTGCTCCAGTAATCAAATAGCACGTACTTGGAAAATGGAGAAACTATTCGACCATTTGAATGAAGTTGCGGCAAAAATGTATGAATTTAATCTTACAGAAAGGCACACTGGTGCCATAAAAACGGAGACAACAAACTCTTTCCTTACTGCTACTGAAGTTTCAACAAGACTCATGAAACCAAGCTGGAAAGTACTTTACCCCTTAACTAATGCTCCGAAGTTTTATCAGGATGAGCGGTACCGTAAGATTCTGAATGATTTCAAAAACCCTACTCTAGGGTTCTTCCACATAGTTGGAGAAGCAGGTATAGGTAAGAGCACACTTGCCAAATTCATTTACAATGATCCAGAAGTAGAAGGAATGTTTCCATCAAGATTGTGGGTTTGTGTGAAAGAGGAATTTGATACACAGAGATTGATGAAAGAGATACTCAACTTTTCATATTCTCCAGCAACTTGTGACAATTTGACTACAAAATTGTGCCCCACAGATCAATATCTGAGAGAGAGAACTTTTCTGCTTGTTTTTCAAGACCTTTCAATCAAGAACCTAGATAATTGTTCCCTGTTTACAAGTTTATTGATGATGGGAAAGCCTGGTAGCAAAATCATAGTGACCACTCAGAATGAGGAAATTGCAAATGCTATAGAACTAACAATGATTTACAAGGTTGGGCAACAATCAGAGCAAAATCGGAGCCAGACAGCCCTAGACACGGTTACTAAAGAGACTGCAAATGTTAACAACGCCGACCAGTTCGTTCAAGCTAACCCTTTGGGTAAGATAGATCAATCTATCCCTTCTCAAACAATATTCAAAGTTAAGAGGCTGTCAGAAAAAGATTCCCTTTCTTTATTCAAAGATTATGCTTCTACATATGAAGGTAATGAAAAAGATATAATGAAAACTCTGAAGAAATGTAATGGAATACCATTGGCAATAAAGTGTCTGGGGAGCATGTTATCTCTAGGTCCTCCAGCAACTAAATGGATGGAGGATAATGAGCGACAAAAGGGAGATAATGAGTCTTCTAGTACATTTAGTATACTTAAACTATGCTACAATGAGATGCCCTCACACCTGAAGCGTTGTTTTCTTTATTGTTCTCAATTACCAAACGATAGCATACTGTCCTCAAATGATGTCATTCAGTTATGGATGGCAAATGGACTTCTCCGTTCACGCCAAGAGAATTACTTATCCTTGGAAGACATAGGTGAGATTTATTTCAAAGAACTATGCTCAAGATGTTTCCTTCAAGATGTTGAGGAATATGGTCTTGGCTATTGGTTTAAAATGCACCCTCTCATTCGGGAACTTGCACGCCTCGTGCAAAAACGAACTAAGGACTTGATAAGCATTAAACCAGTCACCAATGTCACATCTATAGCCTTCCCAGTAAGAGATGAGGTGCCATCTAGTTCATTTCTAGCTGAAAAATGCATCTCAAAGTTCCAACACTTAAGATTATTGTATTTAGGCCACACAGATCTACAGGAAATTCCAAATACTATAGAAACACTGAATCACCTAACATACCTCGACTTGCAGGGGAATAAGAACATCAAGCGGTTACCAAATGCAATCTGTAATCTACAACATTTGCAGACCTTGATTCTTGCATCTTGTTCTGCACTTGAAGAATTGCCAAAAGATATATGCAAGTTGAGCAACCTCAGATACCTGTGGGTAACATCAAACAAGCTTCGTTTGCACAAAAATGGAGTAGGAACCATGACTTCTCTAAGATTCCTCGCAATTGGAGGATGTGACAAACTTCAAGATCTATTCGAACGGCCATCATGCCTTGTACGCCTTGAAACCCTAATGATTTACGATTGTAACTCTTTGCAATTGTTGCCAAACGAGATGGGGTCTCTAATATCGTTACAGAATTTGGTGATATGGAGTTGCAAACAACTTACACTGAAGGGCTTAGAGAAAGTCGATTTCAGCCTCCAAAGATTCACAATCAGAGAGCTTCCAGAAGTGAATAAATTGCCTGAATGGCTTCAAAGGTCGACAGAAACCCTAAGAGTCCTGGAAATCATCGATTGTCCCATCAAAGTGGAGGAAGAGGGAATCAAAATGTACAAAGCAGTTGAAAGTAAGATAATTCAAGGAGCTGTAGACATCACTGGGAATTTGGTACGGCGTAGTCCAATGGTAACGAAGAAAGTACAGATGACAGGTAACTTCTATTAA

>Csa002438

ATGGCTTGTTGCATCTATGAGCAAGCAGAAAATATCTTGATAGAGCTAAAAAAATTCCCAATGTACCTGAGACGAATGCAGTATACCATGTTGAGCCTTAAAACAATTCTTAAGGATGCTGAAAAGGAAGAATATCGTCATTGTCTAAATGATTGGCTACAGAAGCTTCAAAGTGTATTTTTACAAATTGAGGAATTGCTATATGAATCCAATAGGGAAGTCAAAAAACAAGAGGCTACTGGAAAATGGGTATTTCTTCCTTCCTTTAACTTCAGTCAAATTGATCAAACTAAAAAAATGATGAAACTATGCGACGATTTGGATGAAATTGCATCCCATATGTATGGCTTCAATCTAACAAACATGGAGACAACACACTCCTTTCTTAGTGCTACTGAAGTTTCGACAAGACTCATGAAACCAAGTTGGCAATTGCTTTACTCGTTGACTAATGCTCCCAAGGTTTTCCAAGACAAACGATATCATAACTTTCTGGATCATTTCAAAAAATCTACTCACGGGCTCTTCCACATAGTTGGAGAACCAGGTATAGGTAAGACCACACTTGCCAAATTCTTTTACAACAATCTGGTGAACACGTTTCCATCAAGATTGTGGATTTGTGTGAAAGAGGAATTTGATCCACAGAGATTGATAAAAGAGATGCTCAGTTTTTCACATTGCCAAGTAACATGTGATAACTTGACTGAGAAACAATTGTGCTTTGCAGTTCAACAATTTCTGAGGGATAAAAAATTTCTGATTGTTTTTCAAGATATTTCAATCAAAAACCTTGGTAATTGCTCCATATTTAAAAGTTTATTGGGGATGGGAAACCGTGGCAGCAAAATCATAGTGACCACTCAGAATGAGAAAATAGCTGATGCTGTCGGACTAAAAAAACTCTACAAGAACGAGAGCCAGGTAGTTCCATCCCCAGAAGCCACAAAACCTTCAGATGTTAACAAGGATAATATGAAACATCAAACAATTTTCAAAGTTGAGAGGTTGTCAAAGGAAAATTCATTGTCTTTGTTCAAAGTTCATGCTTTCACAGAAACACAGGAAGCACAAATCCCAAATCTCACAAAAATACAAGAAGTAATTGAGCAGAAATGTCATGGGGTTCCTTTGGCAATAAAGTGCCTGGGGGGTCTGCTATCAAAAACTAGTATAGCTGAGTGGAACGGTGTCATCGATAAGTTATGGGAACATGAGGAAGAGGAGGATGGGAATAAGAGTATTTTACCTACACTTAGATTATGCTATGATCAAATGCCTTCACACCTACAACGTTGTTTTCTTTATTGTTCCCAATTAAAAAAAGATCGCATATTGTCTTCCAATGATGTGATTCAATTATGGATTGCAAGCGACCTCCTACCCAAAGAGAATTACTTATCTTTGGAAAAAATAGGTGAGAATTATTTCAAGGAACTATGCTCAAGATGTTTCCTACAAGAACTAGAGGAATATGGTTTTGGCTATTGGTTTAAATTGCACCCTCTTATTGAAAAACTTGCACGTCTACTCACACAAAAACAGGTATTCGAAGTCACAAAAACCCAATCTATAGCCTTCACAATAAGAGATAAGGTGCCCCCTAGTGCATTCCTAGCAAATGCATGCATCGACAAGTTCAAATACTTAAGACTATTGCATTTAGGCAATGCAAATCTACAGGGAATTCCAAGTGCTGTAGAAAATCTGGTACAGCTCAGATACCTAGACTTGCAAGGGAATAAGAAAATCAAGCGGCTACCAAATTCAATCTTCAAGCTAAAAAATTTACAAACCTTGATTCTTGCATCCTGTTCCGCACTTAAAGAACTGCCCAATGATATTAGGCAATTGACCAACCTGAGATACCTCTGGGTAACAGCAAACAACCTTCGTCTGCACAAAAATGGAGTTGGAACCATGACTTCTCTTCGATTTCTCGCAATTGGAGGGTGCCAAAACCTACAAGATCTATTCAAAAAGCCGTCATGCCTCGTACGCCTAGAAACCCTAATGATTTACGATTGTAAGACATTGAAATCGTTGCCAAACGAGATAGGATCGCTAATATCACTAAAGAATTTGGTGATTTGGAGTTGCAAAAAACTTACACTGACGTTGAAAGGAGTGGAGTTCAGGCTTCAGAGGTTCACAATCAGAGAGCTTCCAATAGTGAAAAAATTGCCGGAATGGACTCAAAGATTCACCGAAACCCTAAGAGTTTTGGAAATCATCGATTGTCCCATCGAATGGAATGATGATGTGTTAAAATCATACAAATCACTTGAACGGTTTTCAATTCATGGAGCTGTGAGGACCAAAAACCAGATCGGGGGGTACAACATCGATTATCGTAATTTCGTTAGGAGTAGGAAAGTCAAGAAGGAAGTGAAGACATGTGTCTACTACTAA

>Csa002698

ATGGTTGGACTTCTCGACAGTGTGGCCGGAAATCTGCTCGGAAGGATAATCGAAGCCGCCGACCGACTAGAGTTTCGTGCTATCCAAAGCGAATTGAAAAACCTCGAAACAGATGTGTTGAATCTTAAGGCCAGACTCCGAGACGCCGAGGAGAAGCAGGCTAGTAATTGTGAACTCAATGAACTGCTTAAAAACCTCAAAAATGTGTTTTCAAGGGCAGACATTGCAATTGAGGAATTGGAATGCGATTATTTGAAGTGGAGAGTGCAGAATCGAAAGAACGACGTTGACGATAAGGGATGCCAGTTCTCTTCTTGTTTCTCCTCCAATTTCCTCATTTCTCCATTTAATACCGGCAGTAAATTCCAGGAAGATCTTAAAATAATTACCTCCGAATTACGTTCGATTGAGAAAGCCATGTCTAAATTCTCTCTGGTTGAAGATGAAGATGAATATATAAAAAAATTGAAGGGTGAAATGACTTTGCGGACCTCCATTACTGGTTCGCATGCTTTCGCTAGGCTTCTGCGCTTGAGGAGAGAGGCGATTCTCTCTAATGTAGATTCCATTTTTGGTAGAGATAAAATACAAGAGAGTATCATTAAGGAACTTGTGAATGATGAACAAAAATCTCCCCGTATTCTTTCAATCCAAGGAGATGGAGGGATGGGAAAGACGGCTCTGGCCAAGTTAGTCTACAATGCAGACGAAGTGTTTGATCATTTTGACAAGAGAATGTGGGTATGCGTTTCTGAAGATTTTGATATCCGGAGAATCTTAAGGGAGGTTCTGATGTCTGCAACTGGAGAAAATGTTACCACTGTTGCCTTAACCGAAAGTCGTTTACGAATCCGGCTCCAGCGGTACTTTTTTGGCAAAAAAATCTTGCTTGTTTTGGATGATTTTGGGAATTTGGATCCCGAAAGAGTATCAGAACTGAAAAAAATCGTGAAGATGGGTGTTGGTGGCAGCAAGATAATGATAACCACTCGCAGCGATGAAACTCTAAATGTTGCTACGACACACAAGATTGACAAACTCGACGAGACGATATCTATGCAAATATTCGAAGATACATATGGAAGCGAAGGGCTTAGCGAAGGGCTTAGAGACGATTTGTATCTCAAAAACCTTGTGGCAGAATGTGGAGGAGCTCCTTTGGCAATCAAATGTTTGGCTGGACTGCTCTCTTCAAAACCGAGCGATGGTGCTAAGAGTCCAAATGTCAAGGACTTGAGTGAGAAATGGAAACAGGAAGAGGCAAACAACGGTGGTGGCGTTTTATGTGCACTAAGACTGAGTTATGATCTAATGCCATCTTATTTGAAACCTTGTTTTCTTTGCTTTTCAGTGTTGCCGAAAGATAATGTGTTCTTCTCATTTGAGCTAATCCAGTTATGGATGGCACAAGGAATCCTTCCTTCGGGTACCAAAGATAATCCTGAAGAAGTTGGGGAGAAATATTTCAAGGAATTTCGGGATCGCCGTTTACTCGTTGATGTTGAGGAGCACACTCTTGGATATTGGTTCAAAATCCATAGCCTTGTACATGATCTTGCAGTCCAAAAGGCTACGGAACAAAAGAACCTCGGAAATTTTCATATGCTTTCATTTGTCGATTGCGACAGCATCCCTTCGTCGACAAACTATGATAACACTCGTTTTATTTCCATTCCCGTGGTAGGAGGTGCGGGACCAAATATCAATAGTGACCTTTTCAAATGCATCACCCAGTTCAGGCAGCTAAGGTTTTTGTACTTGTGCAACTCTTCTCTGGAAGAAATTCCAACCTCCATCGACACGCTGAAACATTTGAGGTGTTTAGATTTGCGAGGGAGTCAACGTCTGAAGAGGTTGCCAGAATCAATTTGCAAACTACAGAGCCTACAGACTTTGGTTCTTGCATTCTGCTCAGAGCTTGAAGAGCTTCCCAGAAACATAAAGAACTTGATCAGCCTCAGATTCTTATGGATACAAACAAAGCAAGCCCGCTTGGAAAAAGATGAAATAGGAAGCTTAACATCCCTTCGTTTTCTCGCCATTGGAAGGAGTGAAAACTTGACTCACTTGTTTGAAGATATCAACAAACTCAATTCCCTCAAAACACTGATCATTTATGAGTGCAAATCGCTGCTAACACTGCCAAAAGGCTTGGAAAACATGAAATCTATATGTAATATGGGAATATGGGAATGTGATCGGCTGAGATTTACATTCTCACTGGCTTCACTTCACCTCAAGAAACTGATACTCAGAGAACTTACAGCAGTGTCCACTTTGCCTAATTGGCTGTCCAATTTGGATGGTACTTTAGAAGTGCTAGAAATTGGAGAGTTCCCCACGCTAAGAAAATTGCCAATCTGGCTTTTAAACTTTTGGGAACTCCGAATTCTTGGGATCTCCAACTGTCCTAAGTTGAAGCATGATTCCTTCCCTCCTGAGCTAAATTATTTTTGTGATAAGATTGAGGAGTTGAGGATCACATTTTGTGGGTCTTTGAGCAAGTCTTTGTTGAAAAAAAGCATGAAGGAAATTGAACCTGAAAGCCGGGTAATCTTTTACATCCATACCATTTATGTGGACTCCAAAAGAATGACGCCACCAGTAGAATCAACAGACGAACCTAAGGAAGCAGAGACAAAACAGGATGATGCTTATAACAATGCAAGTCCTCCTGGGACTGAACAACCTTCAAAGACTAAACATGATGATGCGAATAACAATATGAGTCATCCTGGGATTGGACTACTTTCAGAGTCAAAACAGGAGCATACAAATAACAATATAAACGAGATTGAGACTGTTAAGGTTTGTTTGGGTGATAATGACCATGCTGAAGCTCACCAAGCTATGGTCACTACATATGAGGGTTTCTGA

>Csa002745

ATGGCTTCAACGGAAATCTCAACTATTCCCCAAATGATCGAATCGCTATCGGAGCTCCACAAAAATCTCTCCACCGCCCTCGGGAATCACACCGCCGGGGATCAGATCGAGGAGGAGCATCAGACCCCCAAATTCGAGAAATTATTGAACAGCATCGATCACTTAAAGGAGGCGTTGGAGACGACAAGGGAGCTCGATAAGAAGCTCAATGACCCAATTCAGAGCATAAATACCTGTCTCGAGGAGGTTATCAACAGCGTCGAAGCAGCTCAGAGAATTGAAGGGAATTTTCTTGATGCCATAAGTAAGGATCTGAAAACGTTGAAGTTCCGGATCCCTTCTTACCACAAATTTTCGGTTCCAGCCCGTTTGATTGATAGGGGGAGTGACACGCCAGGGCAGAGTGAATTCAAGTTGCCAAATTTGCATGACGACGAGGTGTTTGATGAAAGTCCTGCTTTTATAGAAATTCAGGAAATCTATAATGGCTTTACTGATGATCTTTTTAAAAAGTGTTTTTTGTATTTTGCTGTGTTTCCTGACAATGTTGTGTTAAAGAAGCGGTTTCTTACACATTGGTGGATTGGGGAAGGCCTACTGGACTCTTTAGATAATGGGGATGAAACGCCTGAAGTTCTTGCTGGTAAGATTCTTAAGGAATTTGCAGAGAAGGGTTTGATTGTGCCAGTGATAGAAAAAGAAAAGAAGGTCAAAAGGAGATTTAGAATTCCCCCTCTTGTGCGTTCTGCTGCAATTAAACTGGCCAAACAAAAGGAGTTTTTAGATTATGATATTGGGGACAACCCAACTGGGAAATCTTCCGACTGTGATAGGATTTTTCTAGTGAAGGGGGGAGGCTCTCACCCACCGAAAGCCCCAACGAAGGACCGGAATTTGGAAAAAACAATGGAAGTAATCTTCAATGTTAGCCAACCTTTCCCTGATTCTGCATTGGAGTGGTTAGCGAAGGAAGGGGAAGTAGACATGAGAACTGCCAAAGTTGTGGAATGGTTGCGAAGGCTGAGAAACCTAAAGGTTCTTTACTTGGGGAGATGGCAGAGTGCAGTTGATGAGCAGCATATCGAAGTTGAAAGCCTTGAGTTCTTAAAAGGTTTGAAGAAAATGAAAAAACTAAGGCTTTTGAGCCTCCAAGGGATATCCTGGATCAATAAGCTTCCAAAGTCCATAAGAACATTGAGTGATCTCAGGGTTTTGGACTTGAAATCTTGTTTCAATCTTGAGAAAATTCCTCATAGCATAGGATCTCTCAAAATGCTTACACATCTAGATGTCTCTGGGTGCTATATGCTCAATGGGATGCCCAAGAGTATATCTGCACTAACTGAATTGAGAGTCTTGAAGGGGTTTGTCACAGGAAAGTCAAATCTTAATGATCTAAAAGGCTTAAAGAAGCTGAGAAAGTTAAGCATCAACACAAGCAGACAAGATTTTCCTAATGAAACCGATCTATGTGTTCTCCAAGGACTTGGGGAGCACGGTAAGCTTCGAAATCTGACAATCTCATGGGGGGCAGAAGACGTGAAGCAACAATCTTCGAGCGAACGGAACATTATACGACAGGTATCTAAGAAGTTAAGCAAACAATTATCCAAGACATTGACCAAACAAAGGAGTCAGTTTGGCTATGAGATCGTAGAATTGCCAAAAGAGCTAGAGAAGCTTGAGATGGAATGTCTTCCAAAGGAAGAGCTACCCCCATGGCTAAATCCTTCAAAATTGACAAACCTGAAGAGACTCTACATTAGAGGAGGGAAACTAGCAGGGCTCGGGAACGAGACGTGGAATGCGGAGGTTGTTCGTCTGAAATACATGGCAGATCTGAAGATAGATTGGAGAGAACTTCAGAAAATTCTTCCAAATTTGAGTTACTTCCAGAGGGTAAAATGTCCAAGAGTTACTTTTTGTCCCTGTGATGCGAATGGAGTTTGGATGAAGCCATAA

>Csa002747

ATGGCTTCCCCTTCTCTGATCCTCCATCCTCAAAACTCAACCCCAATTCCAACAATTCCTCAAATGATCGAAACCCTTTCAGATTTATACAAAAAACTCTCCACAGCTCTCCAAAAACATACCACCCAGGCCGAACCCAACAAACCAACACAACAAATACCGGGAATTAGTGGCACCATCGACAACGATAAAGACCCCAAAGTTTTGAAATTGCAGAGCAACATCATGCTTTTGATGGAGGCGTTGGAGACGATGATCAATAATCGCGACAAAAAGCTCAATGTGCCGATACAGAGCATAGAAACCAATCTTGATATAGTTATCAAAAGGGTCAATGATGCCCCTCCTGGTTCTCCACTGACCCAGAAAATCGGAGAGGACTATCTCGACGCCATTATCCAGGATGTAAGAACGTTGAAGTTCCGCATTCCTTCGTACCGCAAGTTGTCTTTGGCCAAAACCGTCGCCCATTCAGGTGGCAGGGGAAGTCATGCACTGACGCCGATTGAGTTTGTGTTGCCTAATTTGCAAGGTGATGAGGTGTTCGACGAGAGTCCTGCTTTCAAAGAAGTTCAGAAAATTTACTATGAGTTTAATGATGATATTTTTAGGAAATGCTTTTTGTATTTTGCTGTGTTTCCTGAAAATGTAGTGTTAAAGAAGCAGTTTCTTACCTATTGGTGGATTGGAGAAGGAATATTAGACGTTAAAGGTACTGGAGATTCAAATCTAGAGGATGAGGCTGGTGGAATTCTTCTGAAATTTGTAGAGAAGGGTTTGATCGTGCCAGTGAAGGAAGAACAGAAGAAGGTCAAAAAGAAATTTAGAATGCCCCCACTTGTGCGTTTTGCTTCCATTAAACTGGCCATTGAAAACAAGTTTTTGAATTTTGATGATTGGGGTAACCCAACTTATAGATCATTCGGCTGTGACAGAATTTTACTGGTGAAGGGAGGAGGCTTTCATCCCCCGGAAGCACCGACGAAGTATCAGAATTTGGAAGAAAAGATGGTAACAATCTTCAATGTTAGCCAGCCTTTCCCCGATTCTGCATTGGAGTGGTTGGCTAAGAAAGGGGATGTAGACATGAGAACTACCAAAGTTGTGGAATGGTTGCTAAAGCTGGAACACCTTAAAGTTCTTTACTTGGGGAGGTGGCAAAGTGAAGTTGATGATGAGGAGCATGTGATTGAGGTTTTAAGCCTTGAGTTTTTAGAAGGATTGAGGAAAATGAAAAAACTGAGGCTTTTGAGCCTTCAGGGGATCTTTTGGATCAATGAGCTTCCAAATTCCATAAATATGCTCTGTGATCTCAGGGTTTTAGACTTGAAATGTTGCTACAATTTGGAGAAACTGCCTGGCGGTATAGGATCTCTCAAGAGCCTTACACATTTAGATGTCACTGGCTGTTATATGCTCAATGGAATGCCAAAGAGCATATCTAGACTCACTCAACTTAGAGTCTTGAAGGGGTTTGTTACAGGGAAGTCAAGTCTTAATGATCTAAAAGGCTTAAAGAAACTGAGGAAGTTGAGCATCAACACAAACAGCCCAGGTTTTCCGGATGCCAAAGATCTACGTGTTCTTCGAGAACTTGGGGAGCACGGTGAACTTCGAAATCTCTCGATTATGTGGGCAGCTGAAGGGTTGAAGTTCGATCAACCACCTTCCAAGACAGAAAAAGGAACGTTTATACGAGAACTAACCAAACAAATTAGCAAACTAACCGCACAACCTAACGATGAAACCTCAGAATTGCCAAAGAACTTAGAGAAGCTGGAACTCGAATGTCTACGGGAGAAAAATCTACCCAACTGGCTAAATCCTGATAACTTGACAAGCTTGAAGAAACTCTACATTAGAGGGGGGAGCCTAGAAACGCTCGGGAATAAAAAGTGGGAGGCGGCCGAGGTTGTTCGGCTCAAATACATGACGGAATTAAAGATAAAATGGAGAGAACTTCAAAACAATTTTCCAAAGCTGAGTTACTTGCAGAAAGTAAAATGTCCAAGAGTTACTCTCTTCCCCTGTGATGCCAGTGGAGTTTGGATAAAGCCATAA

>Csa002916

ATGAAGGACACTACTGAAACTATGTGTAAACGTTACCATGAGTCAAGTCCTTTAGGCTTGATTGCTATGGTTAGGGAGGAATGCATTGAAACACATGGGAATGTTGATATAATTAAGCAGATTCGAGAGACAACATCAAATCTTAATTTTGATGAAGTTATGATAGGAAGAGAAGTGGAAGTTTCAAACATAGTGAAGTTGGTGATTGAATTTAGCAAAGAACATCAAATATCCATCATACCCATTGTGGGTATGCGTGGGTTGGGGAATACAACTTTGGCCTTCAATCATGAGCCGGTTAAAGGTCATTTTGATGAAACTATATGGCTATGTGTGTGGCTGAACATGAAAACACCTCGCAATCTTGTTACTTGA

>Csa002921

ATGGCGGAATTTCTTTGGACTTTCGCAGCTCAAGAGCTGTTGAAGAAGACAGTGAAGCTCGCAGCAGAACAGATCGGCCTGGCATGGGGTTTCAACAATGAGCTGTCAAACCTCAGAGACTCTCTACTTATGGTGGAAGCCATTCTTCGTGATGTCGACAGAATTAAGGCAGAGCATCAAGCTGTGAAGCTATGGGTAGAGAAGCTTGAAGCTATTATTTTCGAAGTCGATGTTCTACTGGATGAGCTCGCTTACGAAGATCTTCGCCGCAAGGTTGAACCCCAAAAAGAGATGATGGTAAGTAATTTCATTTCTTTCTCCAAAACCCCTCTTGTTTTTCGTCTCAAAATGGCCAATAAAATCAAGAACATTGCTAAGATGTTGGAAAGACATTATTCTGCTGCTAGTACTGTGGGGCTTGTTGCTATATTATCTAAACAGACTGAACCTGATTTTAGCCAAATTCAGGAGACAGATTCGTTTCTTGATGAGTATGGAGTTATTGGGAGAGAAAGTGAAGTTTTGGAGATTGTGAATGTATCTGTCGATCTTAGCTATAGGGAGAATTTGTCTGTTTTGCCAATTGTTGGCATGGGTGGATTAGGAAAGACAGCTTTGGCTAAGGTAATATTCAATCATGAATTGATAAAGGGGAATTTTGATAGAGCTGTATGGGTGTGTGTTTCGGAACCTTTTCTTATCAAGAAGATTTTAAGAGCAATTTTGGAAACTCTTAATTCTCATTTTGGTGGCTTAGATAGTAAAGAAGCCTTACTTCAAGAGCTACAAAAGTTGTTGAATGATAAAAAGTATTTTCTAGTTCTTGATGATGTTTGGAATGAGAATCCTATCCTCTGGAATGAGTTGAAAGGTTGTTTGTTAAAGATTAGCCAAAGATCTGGAAATGTTGTTGTTGTGACTACTAGGAGTGACAGAGTTGCTGAAATCATGGAGACACATTCTAGATATCATTTGACAAAACTATCCGATGACCATTGCTGGTCTTTATTCAAGAAATATGCATTTGGAAATGAATTGCTACGAATTCCTGAATTGGATATTGTTCAGAAAGAGCTCGTTAAAAGATTTGGAGGCATACCATTGGCTGTAAAAGTGATGGGAGGAATCGTTAAATTTGACGAGAATCACGAGGGATTGCAGAAATCTTTGGAGAATCTAATGAGACTTCAATTGCAAGATGAAAACCATGTTGTATCCACAATAAAGTTAACTGTAGATCGCCTACCATTGCCATCGTTAAAACAATGTTTTGCCTACTGTTCAAATTTTCCAAAAGACTTTAAGTTCAGAAAAGAAGCCCTTATTCAGATGTGGATAGCACAAGGCTTTATTCAACCGTCTTTGGGAAGTGATGAAATGATGGAGGATATTGGTGAGAAGTACTTCAATGTTTTGTTGTCTCGCTTCTTGTTTCAAGATATTGTCAAGGATAATAGAGGGAGAATTATATTCTGTAAGATGCATGATCTTATACATGATGTTGCATGTGCTATTTCAAATTCTCCAGGATTGAAATGGGATCCTTCAGATTTGTTTGATGGAGAACCTTGGAGACGTCAAGCTTGCTTTGCTAGCCTTGAACTAAAAACGCCAGATTGTAATGAAAATCCTTCTAGAAAGTTGCACATGTTGACATTTGATAGTCATGTGTTTCACAATAAGGTCACAAACTTTCTCTACTTGCGGGTTTTAATTACACATTCGTGGTTTATATGTAAATTACCAAATTCAATTGCTAAGCTGAAGCATTTGAGGTATCTTGACATTTCATATTCTACCATAAGGGAGCTACCAGATTCCGCTGTTTTGCTTTATAATTTGCAAACACTGAAGCTTTCAAGATTTTTAAACGGCCTTCCAAAAAATTTGAGGAAGTTGGTTAGTTTAAGACATTTAGAATTTTTCTCTGATCCTTGTAATCCTAAACAAATGCCTCAACATTTGGGTAAATTGATTCAACTTCAAACGTTGTCTAGCTTTGTAGTTGGGTTTGATGATGGATGTAAGATAGAAGAACTCAGATCTTTGAGAAATCTTAAAGGTAAGTTAAGCCTTTTATGTCTTGAGCGAGTGAAAAGTAAAAAGGAAGCCATGGCTGCAAATTTGGTGGAGAAGAGGAATATTTCATATCTGTCTTTTTATTGGGCCTTGAGATGTGAAAGATCAGAGGGAAGCAACTACAATGATCTGAACGTGTTAGAAGGACTTCAACCACATAAAAATCTTCAAGCTTTGAGAATTCAAAACTTTTTAGGCAAACTTCTGCCCAATGTTATTTTTGTCGAAAATTTGGTCGAGATATATCTACACGAATGCGAAATGTGTGAAACTTTACCAACACTTGGGCAGTTATCAAAGCTTGAAGTACTCGAACTTCGTTGTCTATATAGTGTAAGAAGTATTGGAGAAGAATTTTATGGGAATTACCTTGAGAAGATGATTTTATTCCCAACATTGAAAGCATTTCATATCTGTGAAATGATCAATCTAGAGAATTGGGAAGAAATAATGGTTGTATCAAATGGTACAATCTTTTCCAACCTTGAAAGCTTCAACATTGTTTGTTGTCCGAGATTGACGAGCATTCCAAACCTTTTTGCATCTCAGCATGAGAGTTCATTTCCAAGCTTACAACATTCGGCAAAGCTTCGATCTCTAAAGATTTTGGGATGTGAAAGTTTGCAAAAACAACCAAATGGTTTAGAATTCTGCAGCTCCCTTGAAAACATGTGGATAAGCAACTGTTCTAACTTGAACTACCCTCCAAGCTTGCAGAATATGCAGAATTTAACTTCTTTAAGCATAACCGAGTTTCGAAAGCTGCCAGACGGGTTAGCTCAGGTTTGTAAGTTGAAAAGCTTGAGTGTTCATGGTTACTTGCAAGGTTACGATTGGAGTCCTCTTGTACATCTTGGTTCACTCGAAAATCTTGTGTTGGTTGACTTGGATGGAAGTGGTGCAATACAACTTCCTCAACAACTTGAGCAACTCACTTCTTTGAGATCACTGCATATTTCGCATTTTAGTGGCATTGAAGCGCTACCAGAATGGTTCGGAAACTTTACATGTTTGGAAACGTTGAAGCTTTACAATTGTGTAAACTTGAAAGACATGGCGTCGAAGGAAGCTATGTCAAAACTTACAAGATTAACGAGTCTACGAGTTTATGGATGTCCACAACTTAAGCTTAATATAGGAGACTTTGAGCGGGTAAACATTTCCCTTGTACCTACCATCAGTTGA

>Csa002922

ATGGCTGAATTCCTTTGGACATTTGCTGTGGAAGAGACGTTGAAGAGAACGGTGAACGTTGCAGCTCAGAAAATTTCTCTCGTTTGGGGTTTGGAAGATGAACTTTCAAATTTAAGCAAATGGCTACTCGATGCTGGAGCCCTTTTGCGCGATATCGATAGGGAAATACTTCGCAAGGAATCGGTGAAGAGATGGGCAGATGGGCTTGAAGATATCGTTAGTGAAGCTGAGGATCTTTTGGACGAGCTTGCTTATGAAGATCTTCGAAGAAAAGTGGAAACAAGTTCAAGGGTGTGTAATAATTTCAAATTTTCTTCTGTTCTTAACCCTCTTGTTCGTCATGATATGGCCTGTAAAATGAAGAAAATTACTAAAATGTTAAAACAACATTATCGCAACTCTGCTCCTTTAGGGCTTGTTGGGAAGGAATCCATGGAGAAAGAAGATGGAGGTAATAATCTTAGGCAGATTAGGGAAACAACTTCGATTCTGAATTTTGATGTTGTGGGAAGGGAAACTGAAGTTTTAGACATATTGAGATTGGTGATTGATTCTAGTAGTAATGAGTATGAGCTTCCTTTGTTGATTGTACCGATTGTAGGGATGGGTGGAGTTGGAAAAACAACTTTGGCGAAATTGGTTTTTCGTCATGAGTTGATCAAGAAACATTTTCATGAAACAATATGGATATGTGTGTCGGAACACTTCAACATCGACGAGATTTTGGTAGCAATTTTGGAAAGTTTGACGGATAAAGTTCCAACCAAAAGGGAAGCTGTACTTCGCAGGCTTCAAAAAGAGTTGCTAGACAAAAGATGTTTCCTTGTTTTGGATGATGTTTGGAATGAAAGTTCTAAGTTGTGGGAAGAGTTAGAAGACTGTTTAAAAGAGATAGTTGGGAAATTTGGAATCACCATTATAGTAACTACAAGGTTGGATGAAGTTGCTAATATTATGGGAACAGTTTCGGGTTATCGTTTGGAAAAGTTACCTGAAGACCATTGTTGGTCCTTATTTAAGAGAAGTGCAAATGCAAATGGAGTAAAAATGACTCCAAAGTTGGAGGCTATTCGAATAAAGTTGCTTCAAAAAATTGATGGCATACCGCTTGTTGCAAAAGTTTTGGGAGGAGCCGTGGAATTTGAAGGAGATCTTGATAGGTGGGAGACCACACTTGAAAGCATAGTAAGAGAAATTCCAATGAAACAAAAAAGTTATGTGTTGTCCATATTACAATTAAGTGTGGACCGTCTACCCTTTGTGGAAAAACAATGTTTTGCCTATTGTTCAATTTTTCCTAAAGATTGTGAAGTTGTTAAAGAAAATTTGATTAGAATGTGGATAGCACAAGGGTTTATTCAACCAACAGAAGGAGAGAACACGATGGAGGATCTGGGAGAAGGGCACTTCAACTTCCTCTTATCTCGCTCCTTATTTCAAGATGTCGTCAAGGATAAGTATGGGAGAATTACTCACTTTAAGATGCATGATCTAATACATGATGTTGCCCTTGCCATTTTGTCAACTCGTCAAAAGTCGGTATTAGATCCTACTCATTGGAATGGAAAAACGTCAAGAAAGTTGCGCACCTTACTTTACAATAACCAAGAGATCCACCATAAAGTTGCAGACTGTGTTTTCTTGCGTGTTTTAGAAGTGAATTCCTTACATATGATGAATAACTTACCAGACTTCATTGCTAAGTTGAAACACTTGAGATACCTTGACATTTCATCATGTTCTATGTGGGTTATGCCCCACTCTGTTACTACGCTTTTCAATTTACAGACACTGAAGCTTGGAAGTATAGAAAATCTTCCAATGAATTTGAGAAATTTGGTTAGACTACGTCACTTAGAATTCCACGTCTATTACAACACAAGGAAAATGCCTTCTCATATGGGTGAGTTGATTCATCTTCAAATATTGTCTTGGTTTGTTGCAGGGTTTGAGGAAGGCTGTAAAATTGAAGAACTCGGAAATTTGAAAAATTTGAAAGGTCAATTGCAACTTTCAAATCTTGAGCAAGTGAGGAGTAAAGAAGAAGCTCTAGCTGCAAAATTGGTCAATAAGAAAAACTTACGTGAGCTAACTTTTGAATGGAGTATAGATATTTTACGAGAATGTAGCAGCTACAATGACTTTGAAGTGTTGGAAGGACTTCAACCACCCAAAAATCTCAGTTCTTTGAAAATTACCAACTTTGGAGGGAAATTTTTGCCTGCTGCTACTTTTGTTGAAAATTTGGTGTTCCTATGTTTGTATGGTTGTACAAAATGTGAAAGGCTTCCAATGCTTGGACAATTAGCCAACTTGCAAGAACTTAGTATTTGTTTCATGGATAGTGTGAGAAGTATAGGGAGTGAGTTTTATGGCATTGACTCCAACCGAAGGGGTTATTTTCCCAAGTTGAAGAAATTTGACTTCTGTTGGATGTGCAACCTAGAGCAATGGGAATTAGAAGTGGCAAATCATGAGTCAAATCATTTTGGTTCTCTTCAAACTCTAAAGTTGGATAGATGTGGCAAATTGACAAAACTGCCAAATGGGTTAGAATGTTGCAAATCTGTTCATGAGGTGATAATATCAAATTGTCCTAACCTTACCTTAAATGTAGAGGAAATGCATAACCTGTCTGTTTTATTAATAGATGGGTTGAAGTTTTTGCCAAAAGGATTAGCTCTCCACCCTAACTTGAAGACCATAATGATTAAAGGATGCATAGAGGATTATGATTATAGCCCTTTCCTAAACTTGCCTTCTCTTACAAAACTTTACTTGAACGATGGCCTTGGAAATGCCACCCAGCTTCCTAAACAACTTCAGCATCTCACTGCCTTAAAGATTTTAGCCATTGAAAATTTTTATGGCATTGAAGTTCTTCCTGAATGGTTGAGAAAGCTTACATGTTTGGAGACTTTGGATCTTGTTCGTTGCAAAAACTTGAAACGGTTGCCTTCAAGAGGAGCCATGCGATGCCTCACCAAATTAAAGGATTTCAAAGTTATAGCATGTCCATTGTTGCTACTTGGGGGCCAAGCTGACCAAGAAGGTGCCAAGTATCTTCATATTCCAGCCTATCTTTGTCATGTGTATCAATCTAGAGGAAGCCCTCTTTCCAAAACATCTTCCATCTAA

>Csa002925

ATGGCTATCGCTGAGTTCCTATGGACTTATGCTGTCCAACAAGTGTTGAAGAAGGTATTGGAACTTGCGGCTGACCAAATTGGTTTGGCATGGGGCTTGGACAAGGAGCTTTCAAACCTCTCCCAATGGCTACTCAAAGCAGAAGCTATTTTAGCCGACAATCTATTGGATGAGCTTGTTTATGAATATCTTCGTACAAAGGTGGAAAAAGGATCGATTAACAAGGTATGTTCTTCGGTGTCAAGTCTTTCTAATATTTTCATTATCTTTCGCTTCAAAATGGCCAAGAAAATCAAGAGTATTATTGAAAAGTTGCGTAAATGTTACTACGAGGCGACTCCTTTAGGACTTGTTGGTGAAGAATTCATAGAAACAGAGAATGATCTTAGTCAGATTCGAGAGACGATCTCAAAACTTGATGATTTTGAAGTTGTTGGAAGGGAGTTTGAAGTTTCAAGCATAGTGAAACAAGTAGTTGATGCTAGTAATCAATATGTTACATCTATCTTACCCATTATGGGTATGGGTGGAATCGGAAAAACAACTTTGGCAAAGACAATCTTCAATCATGAGGAGATCAAAGGACATTTTGATGAAACAATTTGGATATGTGTGTCCGAACCATTTCTTATCAACAAGATTTTGGGAGCAATTTTACAAATGATAAAGGGTGTTTCTAGTGGCTTGGATAATAAAGAGGTTCTACTTCAAGAGCTTCAAAAAGTGATGCGAGGTAAAAGATATTTTCTTGTGCTTGATGATGTTTGGAATGAAAATATTGCTTTATGGACTGAATTGAAAAAATGTTTACTGTGTTTTACTGAAAAATCTGGAAACGGTATCATTGTAACTACGAGAAGTATTGAAGTTGGAAAGATTATGGAGAGTACTCTTCCTAGCCATCATTTGGGAAAATTATTTGATGAACAATGTCGGTCTTTGTTTAAAGAAAGTGCAAATGCAGATGAATTGCCAATGGATCCAGAGTTGAAGGATCTTCAAGAAGAATTGGTGACAAGGTTTGGTGGTGTACCATTTGTTGCAAGAGTTTTGGGAGGGGCACCGAAATTTGAAGGGGTCTACGAGAAATGGGTGATGTCTCTTAGAACCACAACAAGTATACCGTTACAAGATGAAGATTTAGTTTTATCTACATTAAAATTAAGTGTAGATCGTCTACCATCCTTCTTGTTGAAGCAATGCTTTGCATATTGTTCAAATTTTCCTAAAGGTTTTAAATTTAAAAAAGAAGAGCTAATTGAAATGTGGATGGCACAAGGGTTCATTCAACTACACGAAGGAAGAAACGATATAACGATGGAGGAAAATGGAGAAAAGTACTTCAACATCTTGTTGTCTCGCTCTCTCTTTCAAGATATCATTAAGGATGATAGAGGAAGAATTACTCATTGTAAGATGCATGATCTTATCTATGAAATTGCGTGTACAATTTTAAATTCTCAAAAGTTGCAAGAGGAACATATTGATTTGTTGGATAAAGGAAGTCACACCAATCATAGGATAAACAACGCCCAAAATTTACGCACACTCATTTGCAATAGACAGGTGCTTCACAAGACTATTTTTGACAAGATTGCAAATTGTACTCGCTTGCGAGTTTTAGTAGTGGATTCATCTATTACAAAACTACCTGAGTCAATTGGTAAGATGAAACATTTGAGATATCTCGACATTTCAAGTTCAAACATAGAAGAACTTCCAAATTCTATCTCTTTGCTTTATAACTTACAAACATTGAAGCTTGGAAGCTCAATGAAACACCTTCCATATAATTTGAGCAAGTTGGTTAGTTTAAGACATTTAAAGTTCTCAATACCACAAACGCCTCCACATTTGAGCCGGTTGACTCAACTACAAACGTTGTCTGGTTTTGCAGTTGGATTTGAGAAGGGTTGCAAAATAGAAGAACTTGGATTTTTGAAAAACTTCAAAGGTAGATTAGAACTTTCAAATCTCAATGGAATTAAACACAAAGAGGAAGCCATGAGTTCCAAATTGGTAGAAAAGAACTTATGTGAGCTATTCTTGGAATGGGATTTGCATATTTTAAGAGAAGGTAGCAACTACAATGACTTGGAAGTGTTAAAAGGGCTTCAACCACACAAAAATCTTCAATTCTTGAGTATCATAAACTATGCTGGCCAAATTTTGCCTCCTGCCATTTTTGTTGAAAATTTAGTTGTGATACATCTAAGACATTGTGTAAGATGCGAAACACTTCCAATGCTTGGAGAATTACCTAATTTGGAGGAACTAAATATTTCCAACTTACATTGTCTAAGATGTATTGGGAATGAATTCTACGGAAGTTATGATCATCCCAACAACCATAAGGTTTTATTTCGCAAGTTGAAGAAATTTGTACTCTCTGAAATGCACAATCTAGAGCAATGGGAAGAATTAGTATTCACATCAAGGAAAGATGCAATTTTTCCTCTTCTTGAAGACTTGAATATTCGTGATTGTCCTATATTAACAAGTATTCCAAATATTTTTGGATGTCCTCTTAAAAAGCTACATGTTTGTGGATGTGATGAAGTGACAAGATTGCCCAAAGATCGATCTACAACTCTGCACTTCCATTGA

>Csa002928

ATGGCTGAGTTCCTATGGACTTTTGCTGTTCAAGAAGTGTTGAAGAAGGTATTGAAACTTGCAGCTGACCAAATTGGTTTGGCATGGGGCTTGGACAAGGAGCTTTCAAACCTCTCCCAATGGCTACTCAAAGCAGAAGCTATTTTAGGTGAGATTAACAGGAAAAAACTACACCCTAGTTCTGTGAGACTGTGGGTGGAAGATCTTCAACTTGTTGTTCATGAAGCAGACGATCTATTGGATGAGCTTGTTTATGAAGATCTTCGTACGAAGGTGGAAAAAGGACCGATTAACAAGGTACGTTCTTCTATATCAAGTCTCTCGAATATTTTCATTATCTTTCGCTTCAAAATGGCCAAGAAAATCAAGGCTATTATTCAAAAGTTGCGTAAATGTTACTCTGAGGCCACTCCTTTAGGACTTGTTGGTGAAGAATTCATAGAAACAGAGAATGATCTTAGTCAGATTCGAGAGACGATCTCAAAACTTGATGATTTTGAAGTTGTTGGAAGGGAGTTTGAAGTTTCAAGCATAGTGAAACAAGTGGTTGATGCTAGTATTGACAATGTTACATCTATCTTGCCCATTGTGGGTATGGGTGGAATCGGAAAAACAACTTTGGCAAAGACAATCTTCAATCATGAGGAGATCAAAGGACATTTTGATGAAACAATTTGGATATGTGTGTCCGAACCATTTCTTATCAACAAAATTTTGGGAGCAATTTTACAAATGATAAAGGGTGTTTCCAGTGGCTTGGATAATAGAGAGGCTTTACTTCGAGAGCTTCAAAAGGTGATGCGAGGTAAAAGATATTTTCTTGTGCTTGATGATGTTTGGAATGAAAATCTTGCTTTATGGACTGAATTGAAACATTGTTTACTGAGTTTCACTGAAAAATCTGGAAACGCTATTATTGTGACTACAAGAAGTTTCGAAGTAGGAAAGATTATGGAGAGTACTCTTTCTAGCCATCATTTGGGAAAATTATCTGATGAACAATGTTGGTCTTTGTTTAAAAAAAGTGCAAATGCAGATGAACTGCCAAAGAATCTAGAGTTGAAGGATCTTCAAGAAGAATTGGTGACAAGGTTTGGTGGTGCACCATTGGTTGCAAGAGTTTTGGGAGGGGCACTGAAATTTGAAGGGGTCTACGAGAAATGGGTGATGTCTCTTAGAACCACAACAAGTATACCGTTACAAGATGAAGATTTAGTTTTATCTACATTAAAATTAAGTGTAGATCGTCTACCATCCTTCTTGTTGAAGCAATGCTTTGCATATTGTTCAAATTTTCCTAAAGGTTTTAAATTTAAAAAAGAAGAGCTAATTGAAATGTGGATGGCACAAGGGTTCATTCAACTACATGAAGGAAGAAACGAGATAACGATGGAGGAAAATGGAGAAAAGTACTTCAACATCTTGTTGTCTCGCTCTCTATTTCAAGATATCATTAAGGATGATAGAGGAAGAATTACTCATTGTAAGATGCATGATCTTATCTATGAAATTGCGTGTACAATTTTAAATTCTCAAAAGTTGCAAGAGGAACATATTGATTTGTTGGATAAAGGAAGTCACACCAATCATAGGATAAACAACGCCCAAAATTTACGCACACTCATTTGCAATAGACAGGTGCTTCACAAGACTATTTTTGACAAGATTGCAAATTGTACATGCCTGCGAGTTTTAGTAGTGGATTCATCTATTACAAAACTACCTGAGTCAATTGGTAAGATAAAACATTTGAGATATCTCGACATTTCAAATTCAAAGATAGAGGAACTTCCAAATTCTATCTCTTTGCTTTATAACTTACAAACACTGAAGCTTGGAAGCTCAATGAAAGACCTTCCACAGAATTTGAGCAAGTTGGTTAGTTTAAGACATCTAAAGTTCTCAATGCCACAAACGCCTCCACATTTGGGTCGATTGACTCAACTTCAAACATTGTCTGGTTTTGCAGTTGGATTCGAGAAGGGTTTCAAAATAGGAGAACTTGGATTTTTGAAAAACCTCAAAGGGAGCGTGACTGAGCAACTTCCTCAACAACTTGAGCATCTCATTGCCTTAAGATCTTTGTACATTAATGATTTTGATGGAATTGAGTTTCCTTCAAAGAAAGCCATGCAATGTCTCACCCAATTAATCCACGTGGATGTCCACAACTGTCCGAGTTCGCAGATTTTGTCCCATGATCTAAAGGCCAAAGCTCATGCCAAAGCAAACTTAGTTCAATGGTAA

>Csa002955

ATGGCGGATTTCCTATGGAGCTTTGCTGTAGATGAAGTGTTAAAGAAGACAGTGAAGCTTGTGGCAGAGCAAATTGGCATGTCATGGGGGTTTAAGAAGGATCTTTCAAAACTAAGGGACTCTTTACTAATGGTAGAAGCCATCCTACGTGATGTTAACAGAATCAAGGCAGAACATCAAGCCTTGAGGCTATGGGTGGAGAAGCTTGAACATATCGTTTTTGAAGCCGACGTTTTACTCGACGAGCTCTCTTACGAAGATCTTCGACGCAAGGTGGACGCCAGGCCGGTACGTAGTTTCGTTTCATCCTCCAAAAATCCCCTTGTTTTTCGCCTAAAAATGGCCAATAAAATTAAAGCTATTGCTAAAAGGTTAGACGAGCATTATTGTGCAGCGAGTATCATGGGGCTTGTTGCTATAACATCCAAAGAAGTCGAGTCCGAACCTAGCCAAATTCTAGAGACAGACTCGTTTCTTGATGAGATTGGAGTTATAGGGAGGGAAGCTGAAGTATTAGAGATAGTGAATAAACTACTTGAACTTAGCAAACAAGAAGCAGCTCTATCTGTTTTACCAATTGTTGGTATAGGTGGACTAGGAAAAACATCTTTGGCGAAGGCGATATTTCATCATGAAATGATAAGGGAGAATTTCGATAGAATGATATGGGTGTGTGTGTCTGAACCTTTTGTTATCAACAAGATTTTAAGAGCAATTTTGGAAACTCTTAATGCTAATTTTGGTGGATTAGACAATAAGGAAGCTTTACTTCAAGAGCTTCAAAAATTGTTGAGGAACAAAAAGTATTTTCTGGTGCTTGACGATGTCTGGAATGAAAATCCTGATCTGTGGAATGAGTTAAGGGCTTGTTTGCTAAAGGCCAATAAAAAATTTGGAAGTGTTATTGTTGTGACTACTAGGAGTGATGAAGTTGCAAATATTGTGGAGACAAATCATCAAAGACATCGTTTGAGAAAGTTATCAAATGATTATTGTTGGACTTTATTTGAAAAATGTGCATTTGGAAGTGATTTGCCAGTGACTCCAAGAGTTGATCATGTAATCAGAGAAGAGCTTGTTAAAAGATTTGGTGGCATACCTTTGGTTGTGAAAGTGTTTGGAGGAATGGTGAAATTAGACAAGAATAAATGTTGTCAAGGATTGCGATCAACTTTGGAAAATCTAATCATAAGTCCATTACAATATGAAAATAGTATTTTATCTACCATAAAATTAAGTGTGGACAGGCTGCCATCATCTTCATTGAAGCAATGTTTTGCCTATTGTTCAAACTTTCCACGAGGCTTCTTATTTATAAGAGAACCACTTGTTCAAATGTGGATAGCACAAGGGTTTATTCATCTACCTAGTGGGAGCAATGTAACGATGGAGGATATTGGAGCAAACTACTTTAATACTTTGTTGTCTCGCTCTTTGTTTCAAGATGTCGTCAAAGATGACAGAGAAAGAATTCTGTATTGCAAGATGCACGATGTTGTACATGATGTTGCATGTGCTATTTCAAATGCTCAAAAATTGAGACTGAGTGGCAAATCTAATGGAGACAAAGCTCTTTCGATCGGTCATGAAATTAGAACACTTCATTGCAGTGAAAATGTTGTTGAACGGTTTCACCTGCCAACCTTTGATAGTCATGTATTTCACAATGAGATCAGCAACTTCACCTACTTGTGCGTTTTAATTATTCATTCATGGTTTATACATCAACTGCCAGATTCAATTGCTAAGTTGAAGCATTTAAGGTACCTCGACATTTCACACTCTCTAATAAGAACGCTTCCAGACTCTATTGTTTCACTCTATAATCTGCAGACATTGAGGCTTGGAAGTAAAATTATGCATCTTCCTACAAAATTGAGAAAATTGGTCAATTTAAGGCATTTAGAATTCTCTCTCTCAACTCAAACTAAACAAATGCCTCAACATCTGAGTCGATTGCTTCAACTTCAAACGCTTTCGAGTTTTGTAGTCGGTTTCGACAAAGGATGTAAGATAGAGGAACTTGGACCACTGAATAACCTTAAAGGTGAACTAAGCCTTTTCCATCTTGAGCATGTCAAAAGTAAAACCGAGGCTATGGCTGCAAATTTGGCAATGAAGGAAAACATTTCTGATCTATATTTTCAATGGAGTTTGTTAAGTGAAAGAGAAGATTGTAGTAACAATGATTTGAATGTGTTGGAAGGGCTTCGACCACACAAAAACCTTCAAGCCTTGAAAATTGAAAACTTTGGAGGTGTTCTGCCTAATGGCCTCTTTGTTGAAAATTTGGTGGAGGTAATTCTATATGATTGCAAAAGATGTGAAACTTTGCCAATGTTGGGGCACTTATCTAAGCTTGAATTACTTCATATTCGTTGCTTAGATAGTGTAAAAAGTATTGGGGATGAATTTTATGGGAACAATAATAGTTACCACAATGAGTGGTCTTCATTGTTATTCCCTAAACTCAAGACCCTTCATATTTCCCAAATGAAAAGTTTAGAGCTTTGGCAAGAAATAGGGAGTTCATCAAACTATGGTGCGACCTTTCCTCATCTTGAAAGCTTGAGCATTGTTTGGTGTTCGAAATTGATGAATATTCCTAACCTTTTTCAAGTTCCTCCAAAGCTTCAATCTCTCAAGATTTTTTATTGTGAAAAATTGACAAAGTTACCACATTGGTTAAATCTCTGCAGCTCCATTGAAAATATGGTCATATGCAATTGTCCTAACGTTAACAATAATTCTCTTCCAAATTTGAAAAGTATGCCAAACTTGTCGTCCTTGAGCATCCAAGCTTTCGAGAAGTTGCCGGAGGGGCTTGCCACCATTCATAACTTGAAAAGATTGGATGTTTATGGGGAATTGCAAGGTTTGGATTGGAGTCCATTCATGTATCTCAATTCATCGATTGAAATTCTTCGGTTGGTTAACACAGGAGTAAGTAATTTACTTCTGCAACTTCCTCGACAACTTGAGTATCTCACCGCTTTAAGATCATTGGATATCGAACGTTTTAGTGACATTGATTCTTTGCCAGAATGGTTGGGAAACCTTACATCTTTAGAGACGTTAAATCTACGTTATTGCAAAAATTTGAAAAGTTTCCCTTCAATAGAAGCCATGTCTAATCTCACCAAATTAAGTCGTTTGGAAACTTATGAATGTTTCCAACTTAAACTCGACGAAGGTAGCTATGAGCGGGCGAAAATTGCGCATGTACATGATATTAGCTGCTAG

>Csa003127

ATGAACAGCCTACTCCAAAATTCCGAGACCCAACCCACCGCGCCCGCCGAAACAAGAACACGAATCTCCGAATGGGTTACACAAACCGTCGACGGATCCACCGTCCACGGCGTCGAAAACGAGCTTCTAGTTCTACAGAAAATGCTCGACATACCACCTATCAGCGGCGGCGGAAACGGCTTCAGAGCAATCGGAATCATCGGAGTACGAGGTATCGGAAAGTCAACAATTTCTCGAGCCTTTCTCCAAAACCCAGAAGTAAAATCCAAGTTCCTCCCCAGAATTTGGATCTCAATGTCGGAAAATTTCACAGAAGACGCCGATCCCAAAATCGCTCTTCTGAAGAGAATCCTAATCTCTCTCGGAGTTGACACGAAGTTTCCCGGTGGCGAAACACTCGGCAGCCTCCTCTACGCTCTCCGACTTCAACTGAGAGGGAAACGGTATCTGATTGTACTGGATGATGTTCAAGAGTTTAAAACAGAGGAAGAACAGAATGATTGGTACTGGGATTTGAATTCTTGTGAGAAAAATGGGGAGAAATTAAGAGATGGGTTTCCGAAAGGGAATGGAGGAGCTGTGATTTTGACGAGTAGAAGTGAGAAAGCTGCAAAAGCTATGGTAGGAGAGGGAAATTTGAGGTGTTTGGTTCCTCATAAAGACCCAGAAAGTTTCTGGGAAATTTTCCGGCAAGAAGTTGTGAAAGATGGAATTTCGATCCCTGATGAAATCCTGAACTTCAAGGAATTGAAAGTGAAATTGCTGAAGAAATGTGGTGGACTTCCGTTGATCGCTAAGATGATGGGAGAGATTCAATTCAAAAAAGAACTAGAAAAGAAGAAAAATACAGAACAACAACGACGAGAAGAAGAAGATCGATGA

>Csa006282

ATGGATATAATTTCTCCTGTCGTTGGACCAATTGTGGAGTACACTTTAAAGCCTATTGGTCGTCAATTGAGTTATCTATTCTTCATTCGCCAACATATTCAAAACCTTGAGAGTCAAGTTGAATTGTTGAAGAACACTAAAGAATCGGTGGTTAACAAGGTTAATGAAGCGATAAGAAATGCTGAAAAGATAGAATCCGGTGTTCAAAGTTGGTTGACTAAGGTGGATTCCATCATTGAAAGATCTGAAACGTTACTAAAGAATCTTTCTGAGCAAGGTGGATTGTGCTTGAATTTGGTCCAGAGACACCAATTAAGTAGGAAAGCTGTAAAGTTGGCTGAGGAGGTTGTTGTGATAAAAATTGAGGGGAATTTCGATAAAGTCTCCTCTCCTGTAGCTCTTTCAGAGGTTGAGAGTTCAAAGGCAAAGAATTCTGATTTTGTCGACTTTGAATCAAGAAAGCCAACTATTGACAAAATCATTGCTGCACTTATGGATGATAATGTCCACACAATTGGAGTGTACGGGATGGGAGGTGTTGGCAAAACAATGCTAGTCCAAGAGATTTCAAAATTAGCTATGGAGCAAAAGCTATTTGATGAAGTAATCACATCAACTGTTAGTCAAACGCCAGACTTAAGAAGAATTCAAGGACAACTTGGTGATAAGCTTGGACTCCGATTTGAACAAGAAACAGAAGAAGGAAGGGCTCTTAAGTTACTAAATAGGTTGAAGATGGAACGTCAAAAGATCCTCATTGTACTTGATGATGTTTGGAAGCAAATTGACTTGGAAAAAATAGGAATTCCAAGCATTGAAGATCACAGTGGATGCAAGATCCTATTTACCTCTAGAGATAATGATGTTCTCTTTAATGATTGGCGCACATATAAAAATTTTGAGATAAAATTTTTACAAGAGGACGAGACGTGGAATTTATTCAGGAAAATGGCTGGTGAGATTGTTGAAACATCTGATTTTAAGAGTATAGCTGTTGAAATAGTAAGGGAATGCGCACATTTGCCCATTGCTATTACTACAATCGCTAGGGCATTGAGAAATAAACCTGCATCCATTTGGAAAGATGCCTTAATCCAACTAAGAAATCCTGTCTTTGTGAATATTAGAGAAATAAATAAGAAAGTGTATTCTTCCCTAAAGTTAAGTTACGATTACTTAGATTCTGAAGAGGCCAAATCACTATTTTTGCTCTGTAGTATGTTCCCAGAAGATTATATCATTGATTGTCAGGTCTTGCATGTATACGCTATGGGCATGGGTTTATTGCATGGTGTTGAGAGTGTAGCACAAGCACGAAATAGGATAACGAAATTAGTTGATGATCTCATATCTTCTTCTTTGCTTTTAAAAGAATCAAATGTCGATTTGGTTATGTATGTTAAAATGCATGATATAGTTCGTGATGTGGCTATAATAATTGCATCTAAAGATGATCGTATTTTTACACTAAGCTATTCCAAAGGATTATTGGATGAATCATGGGATGAAAAGAAACTAGTAGGTAAGCATACTGCAGTGTGCTTAAATGTTAAAGGTTTGCATAACCTTCCCCAAAAGTTAATGCTACCCAAAGTTCAGTTATTGGTGTTTTGTGGAACTTTATTAGGTGAACATGAGTTGCCAGGAACATTTTTTGAAGAAATGAAAGGGATGCGAGTTTTGGAAATAAGAAGCATGAAAATGCCCTTATTGTCACCATCACTTTACTCTTTGACAAACCTTCAATCGTTGCATTTGTTTGATTGTGAATTGGAAAACATAGATGTGATTTGTGAGTTGAACAAACTTGAAAATCTCAGCCTAAAAGGATCACATATCATCCAGATCCCTGCAACTATAAGTCAATTGACACAACTAAAAGTATTAGACTTATCAGAATGTTATGCACTAAAGGTAATTCCGCCTAATATTCTTGTAAATTTGACAAAGTTGGAAGAATTATATTTGCTAAATTTTGATGGTTGGGAAAGCGAAGAATTGAACCAAGGAAGAAGAAATGCTAGTATATCTGAGCTTAGTTACCTTTCTCAGCTTTGTGCTTTAGCATTACATATTCCAAGTGAAAAAGTTATGCCAAAAGAGTTGTTTTCAAGGTTTTTTAATTTGGAAAAGTTTGAAATTTTTATTGGTCGCAAACCTGTTGGACTTCACAAAAGGAAATTCTCAAGAGTGTTGTGTTTGAAGATGGAAACAACAAATAGTATGGATAAAGGAATAAACATGTTGTTAAAGAGGTCAGAAAGATTACATTTAGTAGGATCAATTGGTGCAAGGGTTTTCCCGTTTGAGTTGAATGAAAACGAATCTTCATATTTGAAGTATCTCTACATCAACTATAATTCAAATTTTCAACATTTTATCCATGGACAGAACAAGACTAATTTGCAAAAAGTCTTGTCCAATATGGAGCGTCTGGAATTGAGCTATTTGGAGAATTTGGAGAGTTTTTTTCATGGTGATATTAAAGATATTTCTTTCAACAACTTGAAGGTCATAAAGTTGTTAAGTTGTAATAAATTAGGAAGTCTTTTTTTGGATTCCAACATGAATGGCATGTTATTGCATCTTGAGAGGATTAACATTACTGATTGTGAGAAGGTGAAAACAGTTATTTTAATGGAAAGTGGAAACCCATCTGACCCTGTTGAATTTACAAATTTGAAGCGTTTAAGGCTAAATGGGTTACCACAACTTCAAAGTTTTTACTCCAAAATTGAACAATTGAGTCCTGATCAAGAAGCAGAAAAAGATGAGAGAAGCAGAAATTTCAATGATGGTTTACTTTTTAATGAACAGGTATCACTTCCTAATCTGGAGGATTTGAATATTGAAGAGACTCACAATCTGAAGATGATATGGTGCAATGTACTCATTCCCAATTCCTTTTCTAAGCTCACATCCGTCAAGATTATTAATTGTGAGAGTCTTGAAAAACTTTTTTCTTCAAGTATGATGAGCAGACTTACATGTCTTCAATCCTTATACATTGGAAGTTGCAAGTTATTAGAAGAGGTATTCGAAGGTCAAGAGTCGGGCGTTACGAACAAGGATATTGATTTGCTCCCAAATTTGAGGCGTTTGGACTTAATTGGACTGCCAAAGCTGCAGTTTATATGCGGGAAGAATGATTGTGAATTTCTGAATTTTAAAAGTATACCTAATCTGACCATTGGTGGTTGTCCAAAACTTGAAGCAAAGTATTTAATCCAAGTTCTTGACAACATGAAAGATCTGACAATAGATTTAAGAAGATTGGAGGAGATTTTAAACAAGGAAAAGTCAGTGGTAGAACTTGATCTTTCATTGGAGACGTCCAAGGATGGTGGAGAGTTATTTGGAAAACTTGAATTTTTGGACTTGTGCGGTTCGCTTAGTCCTGACTACAAAACGATTACTCATTTGCCAATGGAAATTGTTCCAATATTACACAACCTTAAAAGTTTGATTGTGAAAAGGACATTCTTGGAAGAAATCTTTCCAATGACAAGATTGGGTAATGTTGAAGAATGGCAAAATAAAAGATTCAAGCTTTCCTCTTTGGCATTAAGGGAATTGCCCAAACTTAAGCATTTGTGCAACGAAGACTTACAAAAGAATTCTTCGATGCTTCAAAATTTGAAATATTTCTCCATAAAGGGATGTGGGAAATTGAATATGTTTGTTCCATCGTCAATGTCGTTTAGAAACCTGGTCGATTTGAAAGTGATGGAATGTCATAAATTGATCTATTTGATAAATCCTTCTGTAGCTAGAACCATGGGTCAACTTAGACAGTTGGAAATAAGACGATGCAAAAGAATGACAAGTGTGATTGCTAAAGAAGAAAATGATGAGATTTTATTCAACAAATTAATCTACTTGGTTGTTGTGGATTTGCCCAAATTGCTGAACTTTCATTCCGGAAAATGTACCATTAGATTTCCGGTCTTGAGACGAATAAGTGTTCAAAATTGTCCTGAAATGAAGGATTTTTGTACTGGAATTGTTAGCACGCCTCATTTGCTAACTGAAAGTATTATACACTACGATGATGCAACTAATAAATATATTCCAATACTTAAAGATTATTCAAAAGAGGCCATTGTAAAAGATATGAATGTTGCTATTCGACAAGTTTGGGAGAACCATTACGACTTCAACCTTCACTGTTTGTTTGAAGTAGAGAATCTGAAGGAGGAAAACCAATGTGAATCTTCTTCACATGTTGAGCAGTTAGAGACACAGTGCCTTACGTTCCACGCTTTCATCTCTGTTGAATGTTTCACGCTTGCGATACCCACCCACGTCCCGTCTCACACGACTGCCCTCCGTTCAACCCACGACGACGCCGTTGTTGTCTCTGTCCAATCGCCAATCGCTAATGCTTTCAAATCAAACTCGTTTGAATTCTAA

>Csa006679

ATGCAGTTGCGTGTACCTAAATATCCGGTTGGAATAGATATACAAGTTGATAATTTACTCTTCCATGTTGTGTCTGATGAATTAATTACTATGGTTGGATTATATGGAATTGGAGGTATTGGCAAAACAACTTTGGCCAGAGCTTTGTACAATAAAATTGTGGATGACTTTGAAAGTTGTTGCTTTTTGGCAAATGTTAGAGAAGCTTCAAATCAATATCGGGGTCTTGTTGGACTCCAAAATGAGCTACTTCGTGAGATTTTAGTAGATGATTCAATCAAAGTTAGCAATCTTGATATTGGAATTAGCATTATAAGGGATCGACTATGCTCAAAAAAGATTCTTTTGATTCTTGATGATGTTGATACGAGTGAACAACTAGAAGCATTAGCGGGAGGACGTGATTGGTTTGGACCTAGAAGTATGGTCATTGCGACAACAAGAAACAAACATTTACTTGCTATTCATGAATTTGATATATTGCAAAGTGTTAAGGGATTGAATGATGATGAAGCCCTCGAGCTTTTTAGTTGGCATGCTTTTAAGACGAGTTGTCCATCAAGTGATTATTTAGACCTTTCAAAACGTGTCGTACGTTATTGTAAAGGTCTTCCTTTGGCTCTTGAAGTTGTAGGAGAGGATATCAACAAAGTTAAACTGATGTTAGAAGCATGTGGTTGTTTATGTTTGGAAAAGAGAACCACAAAACTCATGAATTTATCACTTCTTACCATTGATGAATCCAATCAGGTTGAAAAGGTTGTCTACAAATTTGGCTTTCTTAGAAACAATGGTTCATGCCACCCCTTCATTGAAAAGGTTGGACTTGTCCAAAAACAACTTTTGTAG

>Csa006693

ATGGCGGGAGCTTTAATTGGTGGCGCGGCATTGGGTGTTCCGTTTAACGAGCTAGCGACCCTCTTGAAGAATTTTGGCGAGAGGGCGTGGAGTTTCAATTCTGTTCTTAACGAGACCGAATCCAAGGTAAATGATATAATTCCTCTGGTTAAAGAAATAGATGGTCTTAATGAATCCCTGGATTATCCAAGAGAAGAAACGGAGAAGTTGAAAAACTTATTAGAATATGCTGGAAAGCTACTTAGACGGTGTTTAAGAGTGGGGAAGGCTGATTTGATAAGGAAATCAAGTCATACAGAGAAGCTTCGTGAACTGAATGCCAGAATCAAAAGTTTCAGTGACGTTGTGTTGTTCCAAACGTCTAGAGACGGGAAGAAGACATTGAGTTTAGTGACTGAGATCAAGGAAGTCGTTCGCAGGCTTGATAGCAAATCTGGATTAAGCAATCCGGTGGATTTAGTTGTGACGGTTCCTGTGATTTCAGAAGAAAGTGTTGGGTTGGAAAAGCCTGTTGAGAAATTGAAGGCCAAACTATTTAGAGATGGGGTTCGATTGTTGGTAGTGACAGCTCCCGGAGGTTGTGGAAAAAGCACTCTGGCCGAAATTTTTTGTCACGACAAGCAAGTTAAAAATAAATTTCAGAGAAACATCTTGTTCCTCGTTGTCTCAAGCAAACCAGAAACGAAACGCATCTTAATATCTATAATTCAAAGACTCGGGGGGCCTATAGAATCTGGTTCTGTAAGTGATGATGAGGCATTCCGGTTGTTAGAAGTTCGGGTGGGGGAATTGAGTCCAAATCCTGTATTGATTGTGTTGGACGATGTCTGGGACGGTTCTGAATCAAACAAGCTTCTTGAAAAGTTCTCCCGATTACCCAACTGCAAAGTTTTGGTCACTTCTAGATTTAAGTTTCCTGCATTTGGTGAGTCGTATGATTTGGAACCTCTGGACCATAAGGATGCAATGGAGTTGTTTCGTCGCTGGGCATCGAGGGGTAACAGAGTGCTACAGTTCCCAGATGAAAGAATTGTAGAAAAGATAGTGAGGGGTTGTAAGAGATTCCCACTTGCTCTGAAAGTGATTGCAGGATCACTTTCGGGTAGAGCCACTTCGGTTTGGGAAGTTACGGGGAGGAAATTATCTAGAGGAGATTCTATTCTGGGTTCTGAGAAAGAGCTTCAGAAGTGCCTCAAAGACACCTTAGATGCAATCCCAGATGACAAGATAGTTCTCAAGGAGTGTTTCATGGACTTAGGTTCATTTCCTGAAGATCAAAGAATTCGTGCGGCTACCTTCATTGACATTTGTGCAGTGTTGTATGAACAAGATGAATGTGAAACAATGTCAAACCTTGATGAGCTCTTCACCCGGACTTTAGTTAACACTGTCTCTTTGAGAAATAAAGCGCATGAAGATGATTACTACAGCGAGTCCTATATTACACAGCATGACGTACTTAGAGAATTGGCTGTCCTTTTGACTAATGAGCAGCCAGTAGACCAAAGAACAAGATTGCTTGTGGATATTAACAAAAATGAATTTCCCAAATGGTGGTCTGTAAGACAGATGCAACCTGTGAAAGCCCGCCTTTTGTCCATAACAACAGATGAGAAGTTCTCATCATGTTGGCCTGATATGGAAGCACCTGAAGTTGAGGTGTTAATTCTAAATCCTGGGTCAGAAACTTACAAGTTACCTGATTTTGCAAAGAAAATGAACAGATTGAAAGCGCTGATAGTCAGGAATTACAGGTCCTTTCCAACTGAATTGACAAGTGATTATCAATTAATCAATTGTTTGTCAAGGCTAGAAAGAATCAGTCTTGAGCGGATTTCAATATCTTCTTTCATTGACCAGAACCTGAAGCCCCTGTGGCATCTTAAGAAGCTATCGTTCTTTATGTGCAAAATTGACAAAGCTTTCACAGACTGCTCAACTCAGATCTCATACATGTTGCCTAACTTACTTGAGATCTCCATAGATTTTTGCAACGATTTGGTGGCTTTCCCTGTCGGACTATGTGAAGTTGTCACATTGGAGAAACTGAGCATTACAAACTGTCATATATTATCTTCGTTACCCGAGGAAATTGGGCAGTTGATTAATCTAAAAATTCTAAGGCTTAGATCTTGTATTCATTTGGAGAAGTTGCCAGAATCAATCTCAAGGCTCCGGGAATTAGTTTATCTTGACATATCTCATTGTGTTGGCCTTACCAAACTTCCAGATAAGATTGGCAACTTGCAGAAGTTGGAAAAGCTTAATATGTGGAGTTGCCCGAACATGCGCAAGCTTCCAAAATCAGTAGGAAATCTAAAAAATTTGAAGGAAGTAGTTTGTGAAAGCGAGATGAAAATATGGGTGAATTTTGTCGCACCTCGGCTTGGCAATGTGGTAAAAGAACACAAGGAAGAAATCAACTTGGATTTTCTAAATTGA

>Csa006711

ATGGGTTCTTCCACTGCTGCAACAGAATCCATGGCTTTTGAATGGAGTTATGATGTTTTTTTGAGTTTCAGAGGAGAGGATACTCGTACCAATTTCACCAGTCATCTTGATATGGCCTTGCGTCAAAAGGGGGTCAACGTCTTCATAGACGACAAGCTCGAAAGGGGTGAGCAAATTTCTGAATCCCTTTTCAAATCTATACAGGAAGCTTCCATTTCTATTGTTATATTCTCTCAAAATTATGCATCTTCTTCCTGGTGTCTGGATGAATTGGTGAACATAATTGAGTGTAAGAAATCCAAGGGCCAGAATGTTTTCCCAGTTTTCTATAAGGTGGATCCGTCGGATATACGAAAACAAACTGGTAGCTTCGGAGAAGCACTGGCCAAACATCAGCCTAAGTTCCAAACAAAGACCCAAATTTGGAGGGAAGCTTTAACTACTGCTGCTAACTTGTCTGGTTGGGATCTAGGAGCTTATAGGAGGGAGGCTGATCTTATTCGGGATCTTGTTAAGGAAGTGTTATCTACAATAAATCGCACTCGCACACCCTTATATGTCGCCAAGTATCCAGTTGGAATTGATTCTCAACTAGAATACATGAAGTTTCACTCACATCATCTCAACAAGGGAAACAAATTCCAATATTGGACACAAAATGAGTATGAGTCTGATATTGGTGTTTACATGGTGGGGATATATGGCATTGGAGGCCTTGGTAAGACAACTTTGGCTAAAGCTCTATACAATAAAATAGCTAGCCAATTTGAAGGGTGCTGTTTTCTATCAAATGTTCGACAAGCTTCAAACCAATTCAATGGCCTTGTTCAACTACAGCAAAACCTACTCTATGAAATCTTAGAGGATGATTTGAAGTTTGTCAATCTTGATAAAGGAATTACCATCATAAGGAATAGACTGCGTTCAAAGAAAGTTTTGATAGTTCTTGATGATGTGGATAAGCTCGAACAACTAGAAGCATTGGTTGGTGGACGTGATTGGTTTGGTCAAGGTAGTAAAATCATAGTGACGACGAGGAATAGTCATTTACTTTCTAGCCATGGATTTGATGAAATGCACAATATTCAAGGATTGAATCAAGACAGAGCTATTGAGCTTTTTAGTTGGCATGCTTTTAAGGAAAGTCATCCATCAAGTAATTATTTAGACCTTGCCGAACGTGCTACAAGTTATTGTAAAGGTCATCCTTTGGCTCTTGTTGTTCTGGGTTCTTTCCTTTGTAATAGAGGTCAAACAGAATGGAGAAGTATATTGGATAAATTTGAAAACTCTTTGAACAATGATATTAAAGATATTCTTCAATTAAGTTTTGATGGGCTGGAAGGTGGAGTAAAGGATATTTTTCTTGATATTTCTTGTTTATTTGTAGGGGAAAAATACAATAATTGTGCTAAAAAAATGTTGAGTGCATGCCATTTGAACGTAGATTTTGGAATTATGATACTCATGGATCTTTCACTTGTTACGATTGAAAAGGATAGAGTGCAAATGCACGGATTAATACAACAGATGGGTCATAGCATAGTTCATAATGAATCATTTGAGTCAGGAAAGAGGAGTAGATTGTGGTCGGAGCGGGACATTTGGAACGTGTTTGTTAATAATTCGGGAACAGATGCAATTAAAGCCATAAAGTTAGACTTGCCTAATCCCATAAACGTAAATGTAGATCCAAAAGCATTCTTTAGAAGCATGAAAAATTTGAGATTGCTTATCATTCGAAATGCACAAGTTTGTACAAAGATTAAGTACCTACCTAATAGCTTAAAGTGGATTGAGTGGCAAGGATTTGCTCATCGAACTTTCCCGTCGTGCTTCATTACCAAAAATCTTGTTGGACTTGATTTGCGACGTAGCTTCATCAAAAGATTTGGGAAAAGACTTGAGGATTGTGAAAGGTTGAAGCATGTTGATCTTAGCTACTCTACTTTATTAGAGAAAATTCCTGATTTATCCGCTGCATCAAACCTTGAAGAATTGTATCTCATCAATTGCACAAATTTAGGAATGATAGATAAGTCTGTTTTCTCTCTCAATAAGCTTACTGTCCTAAACTTTAAAGGTTGTTCTAACCTTAAAAAGCTTCCAAAAGGCTACTTCATGTTCAGTTCTCTTAAAATATTGAATCTCTCTTACTGCCAAGAACTTGAGAAAATTCCAGACTTATCTTCAGCATCAAACCTTCAGAGCTTGCTACTCAACGGATGCACAAATTTAAGAGTGATTCATGAATCTGTTGGATCTTTGAATGAGCTTGTATTGTTGGACCTTGGACAATGCACTAACCTTTCAAAGCTTCCGAGCTATCTCAGGTTAAAGTCTCTTGTCTATTTGGTACTTTTTGGGTGTGGTAAGCTTGAAAGCTTTCCAACAATTGCTGAAAACATGAAATCTTTAAGGTGCTTGGATTTGCATTCCACCGCCATAAAGGAGTTACCTTCATCACTTGGATATCTTACTCAACTCGATAAATTACACCTTACCGGTTGCACAAATCTCATCTCCCTTCCCAATACAATCTATTTGTTAAGGAATCTTAACGAACTTCATCTTGGTGGGTGTTCTAGATTTGAAATGTTTCCCCATAAATGGGTCCCAACCATCCAACCAGTATGCTCTCCTTCAAAAATGATGGAAGCAGCTTCGTGGAGCTTAGAGTTTCCCCATTTAGTAGTACCAAATGAAAGCATATGTTCCCATTTCACTTTGTTGGATCTTAAATCTTGCAACATATCAAGTGCAAAATTTTTGGAAATTTTATGTGATGTTGCCCCTTTCTTATCTGATCTACGTTTGTCCGAAAACAAATTCTCTAGTTTACCCTCATGTCTCCACAAGTTCATGTCCTTATCGAATCTTGAATTAAGGAATTGTAAGTTTCTTCAAGAAATCCCAAACCTTCCCCAAAACATACGAAACTTGGATGCCAGTGGTTGCAAATCGTTGGCTCGAAGTCCAGATAACATTGTGGATATAATATCAATAAAACAGGACCTTGAATTGGGTGAGATTTTAAGAGAGTTCTTATTAACGGACATTGAGATTCCAGAATGGTTCAGCTATAAGACTACATCCAATTTGGTGAGTGCTAGCTTTCGTCACTATCCAGACATGGAAAGAACTTTGGCTGTCGCTGTTAGTTTTAAAGTGAATGGAGATTCATCTGAAACGTGTTCCCTGGAGGTGAATGATTGGAATAAAGTTTTCGTCTGGTTTGAGGTTCATGAAGCACATGGTGTAACTGTAACAAGGTATGGGGTCCATGTCACTGAACAACTCCATGGGATACAAACGGATGTCAAGTGGCCGATGGTAAATTATGCTGATTTTTATCAACTGGAGAAATTGCGAAGGGATCTGGATTTTGAGGATCTCAAAGCTAGTTTAAAGAAGTCTGCTGTTCAAATTCCAAAGCAACATTGCATGCACTTTAGTATGATCCAGAGGCAATAA

>Csa006724

ATGGATTCTTCCACTGTTGCAGCAGAATCATCTACTTTCAAATGGAGCTATGATGTGTGTTTGAGTTTCAGAGGAGAGGATACTCGAGACAATTTCACCAGTCATCTTGACATGGCCTTGCGTCAAAAGGGTGTCAACGTCTTCATTGACGACCAGCTCGAAAGGGGTGAGCAAATTTCTGAAACCCTTTTCAAATCTATACACAAAACTTCCATTTCTATTGTTATATTCTCTGAAAATTATGCATCTTCGACATGGTGTCTGGATGAATTGGTGGAAATAATTGAATGTAAGAAATCCAAGGGTCAGGAAGTTTTGCCGATTTTCTACAAGGTGGATCCTTCGGATGTACGAAAACAAACTGGTTGGTTTGGAGGAGCATTGGCCAAACATGAGGCTAATTTCATGGAGAAGATTCCAATATGGAGGGATGCTTTAACTACTGCTGCCAACTTAGCTGGTTGGGATCTCGGAACCATAAGAAAGGAGGCTGATCTTATTCAAGTTATTGTTGAACGAGTGTTGTCTATATTAAATCAAACCCACACGCCCTTAAAGGTAGCTGAGTATCCAGTTGGAATTGATTACAAAATAGAATCCCTTTACTGGACACAAGAAATGTACAAGTCTGAATGTGTTGACATGGTGGGGATATATGGCATTCGAGGCATTGGTAAAACAACTTTGGCTAAAGCTTTATACAACAAAATTGCTAGCCAATTTGAAGGTTGCTGCTTTCTATCAAATGTTAGAGAAGCTTCAAAGCAATTCAATGGCCTTGCTCAATTACAGAAAAAGCTCCTTTTTCAAATCTTAAAGTATGATTTGGAGGTTGTCGATCTTGACAGGGGACATAATATCAAGCAAGCAGACAATTTTTTAATTGCGACTAATTCCAAAATGCAAGCATACGATCATGTACATGACGATATTCGTTTTACACCAAAACGTGGCATGGAAGGCCTGACAGAGATAACACTCTCCAAATCTATATGGGACAAATTTGTAAAAGATCATAATATTACCTCTGAAATACTAGCTTCAAATGATTCTAATGCATTGGTTCGTGGATACATAGATGGAGATAAACTCTATCTGGTCACACACGATAGACAACACTTCCAAGAATATTTGGGATAG

>Csa006742

ATGGGTTCTTCTGTTGTTGGAGATGAATCATTTTCTTCTTCTCCCAATTTCAATTACGATTATGATGTGTTTTTTAGTTTCAGAGGAGAAGATACTCGCTCCAATTTTATCAGTCATCTTCATATGGCCTTGCGTCTAAAGGAAGTCAACGTTTTCATAGACGACAAACTCAAAAGGGGTGAACAAATTTATGAGTCTCTTCTCAAATTTATAGAGCGATCTAGACTTTCCCTCGTTATTTTCTCTAAAGATTATGCATCTTCAACTTGGTGTTTGGATGAACTGGTGAAAATAATTGAGTGTAAGAAATCCAAAGGACAAGCAGTTTGGCCAGTGTTCTACAAGGTGGATCCATCCGAGGTTCGAAAACAAACCGGTGGGTTTGGGGAAGCATTGGCCAAACATGAAGCTAATAAGTTATTGACCAACAAGATTCAACCATGGAGGGAAGCTTTGACTTTTGCTGCTGGTTTGTCTGGTTGGGATCTAGCAAATAGCAAGGATGAGGCTGAACTTATCCAAAAAATTGTTAAACGAGTGTTGTCTGCAGTAAATCCAATGCAATTACTACATGTAGCCAAGCACCAAGTAGGAGTTGATTCTCGACTAAGGAAAATTGAGGAGTTGGTCTCTCATATTGGGTCCGAGGGTGTTAATCTGGTGGGGTTGTATGGCATTGGAGGCATTGGTAAGACCACTTTGGCTAAGGCTTTGTACAACAAAATTGCTACCCAATTTGAAGGATGCTGCTTTCTACAAGATGTTAGACGAGAAGCTTCGAAGCATGGGCTCGTTCAACTACAGGAAACCTTACTCAATGAGATCTTAAAAGAGGATTTGAAGGTTATTGTCAGTCGTGATAGAGGAATTAACATCATAAGAAGTAGACTGTGTTCAAAGAAAGTTCTTATAGTTCTTGATGATGTGAATGATCTTGAGCAATTAGAAGCACTGGTTGGTGGGCGTGATTGGTTTGGTCAAGGTAGTAAAATCATTGTGACGACAAGGAATGAACATTTACTTTCTAGCCATGGATTTGATGCTTTTCAGTTGGCATGCTTTTAA

>Csa006744

ATGGATTCTTCCACTGTTGCAACAGAATCACCGACTTTCAAATGGACTTATGATGTGTTTTTGAGTTTCAGAGGAGAGGATACTCGCACCAATTTCACCAGTCATCTTGATATGGCCTTGCGTCAAAAGGGTGTCAATGTCTTCATAAACGACAAGCTCGAAAGGGGTGAGCAAATTTCTGAATCCCTTTTCAAATCTATACAGGAAGCTTCCATTTCTATTGTTATATTCTCTCAAAATTATGCATCTTCTTCCTGGTGTCTGGATGAGTTGGTGAACATAATTGAGTGTAAGAAATCCAAGGGCCAGAATGTTTTCCCAGTTTTCTATAAGGTGGATCCGTCGGATATACGAAAACAAACTGGTAGCTTCGGAGAAGCACTGGCCAAACATCAGCCTAAGTTCCAAACAAAGACCCAAATTTGGAGGGAAGCTTTAACTACTGCTGCTAACTTGTCTGGTTGGAATCTAGGAACTAGGAAGGAGGCTGATCTTATTGGAGATCTTGTTAAAAAAGTGTTGTCTGTATTAAACCGCACTTGCACGCCCTTATATGTAGCTAAGTATCCGGTTGGAATTGATTCTAAACTAGAATATATGAAGCTTCGTTCACATAGTCTTTTTGAGAAGAGCAACAAATTCCATTATCGAAAACAACATGAGTATGAGTCTGATACTGGTGTTTACATGGTGGGCTTATATGGCATTGGAGGCATTGGTAAGACAACTTTGGCTAAAGCTTTATACAACAAAATTGCTAGCCAATTTGAAGGTTGCTGCTTTCTATCAAATGTTAGAGAAGCTTCAAAGCAATTCAATGGCCTTGCTCAACTACAGGAAACCCTACTCTATGAGATCCTAACGGTTGATTTGAAGGTTGTCAACCTTGATAGAGGAATTAACATCATAAGGAATAGATTGTGTTTGAAGAAAGTCCTTATAGTTCTTGATGATGTAGATAAGCTTGAGCAGTTAGAAGCATTGGTTGGCGGGCGTGATTGGTTTGGCCAAGGCAGTAGAATCATTGTGACGACAAGGAACAAACATTTACTTTCTAGCCATGGCTTTGATGAAATGAAAAATATTCTAGGATTGGATGAAGACAAAGCTATTGAGCTTTTTAGTTGGCATGCTTTCAAGAAAAATCATCCATCAAGTAATTATTTAGACCTTTCAAAACGTGCTACAAGTTATTGTAAAGGCCATCCTTTGGCTCTCGTTGTTTTGGGTTCTTTCCTCTGTACCAGAGATCAAGGAACAGACGCAGTTAAAGGCATAAAGTTGGACTTTCCTAATTCCACGAGGCTGGATGTGGATCCACAAGCTTTTAGAAAAATGAAAAATTTGAGATTGCTTATTGTTCAAAATGCAAGATTTTCTACAAAGATTGAGTACCTACCTGATAGCTTAAAGTGGATTAAGTGGCATGGATTTCGTCAACCAACTTTTCCTTCGTTCTTCACTATGAAAAATCTTGTTGGACTAGATTTGCAACATAGCTTCATCAAAACATTTGGGAAAAGACTTGAGGATTGTGAAAGGTTGAAGCTTGTTGATCTTAGCTACTCTACTTTCTTAGAGAAAATTCCTAATTTCTCTGCAGCATCAAACCTTGAAGAGTTGTATCTCTCCAATTGCACAAATTTAGGAATGATAGATAAGTCTGTTTTCTCTCTCGATAAGCTTACTGTCCTAAACCTTGATGGTTGTTCTAACCTTAAAAAGCTTCCAAGAGGCTACTTCATGTTAAGTTCTCTTAAAAAATTGAATCTCTCTTACTGCAAAAAACTTGAGAAAATTCCAGACTTATCTTCAGCATCAAACCTTACGAGCTTGCATATCTATGAATGCACAAATTTAAGAGTAATTCATGAATCTGTTGGATCTTTGGATAAGCTTGAAGGTTTGTACCTTAAACAATGCACTAACCTGGACCTCACAATGGGTGAGATTTCAAGAGAGTTTTTATTAACGGGGATTGAGATTCCAGAATGGTTCAGCTATAAGACTACATCCAATTTGGTGAGTGCTAGCTTTCGTCACTATCCAGACATGGAAAGAACTTTGGCTGCCTGTGTTAGTTTCAAAGTAAATGGAAATTCATCTGAAAGAGAATATATGTGGTTAGTAACAACTTCTCTAGCGTTGGGTTCCATGGAGGTGAATGACTGGAATAAAGTTTTGGTCTGGTTTGAGGTTCATGAAGCACATAGTGAGGTTAATGCAACTATAACAAGGTATGGTGTCCATGTCACTGAAGAGCTCCATGCGATACAAACGGATGTCAAGTGGCCGATGGTAAATTATGCTGATTTTTATCAACTGGAGAAATTGCAAAGTCTGGATATTGAGGAACTTCTTCTCAAACGCTTTTTTGAAGAAATGTCGTGCTGGTCCAATTCCCAAGCAATGTTATATGCGGCAAATTATGATCCAGAAGCAATAATCGATTCGAATATACAACCTATGATATTTCCATTGCACGTAACATATAATGGTGAGACATTTATATGTGGAATGGAAGGCATGGGAGACACTACACTCGCCAACTCTTTATGCAATAAATTTAATTGGCCAAATGACAACGTTCGGGCAAGAGAAGCTTTAGATAATTCTACAAGCTTTTTGCATTTTCGAGGAGGAAAGTTTAATGGAGGTTCCTGGTCATCGTCCCACCACCGTAAGCGTGGAGATGGTGAAAGAGGAACCAATATCACAACCCGCACAATATCCTCCAAACGCTATTTGATACTCTTTCATAAAGCGGGGAGCTATGATGATTTATTTAACTTTGCTGGTAGCCACCGTTTGATTGCAGGTTCTCGCAGTTATGACAGTCTTAACGGAAGAGGTGATGTTCGGCTTCTGATTGAAAGGGTTGATACATCCTTGCTCTGA

>Csa006756

ATGCAGTTGGATGTAGCTAAATATCCAGTTGGAATTGACATACAAGTTAGTAATTTACTCCCACATGTTATGTCTAATGGAATTACCATGTTTGGATTATATGGAGTTGGAGGTATGGGCAAGACAACTATAGCGAAAGCTTTATACAATAAAATTGCTGATGAGTTTGAAGGTTGTTGCTTTCTGTCAAATATTAGAGAAGCTTCGAATCAATATGGAGGCCTTGTTCAATTTCAAAAGGAGCTACTTTGTGAGATTCTAATGGATGATTCGATTAAAGTTAGCAATCTTCCTAGAGGAATTACTATCATAAGGAATCGACTATACTCAAAAAAGATTCTTTTGATTCTTGATGATGTTGATACGCGTGAACAACTACAGGCATTGGCGGGAGGACATGATTGGTTTGGACATGGAAGTAAGGTGATTGCGACAACAAGAAACAAGCAATTACTTGTTACTCATGGATTTGATAAAATGCAAAATGTTGGGGGATTGGATTACGATGAAGCTCTTGAGCTCTTTAGTTGGCATTGTTTTAGGAATAGTCATCCCTTAAATGTTTATTTAGAACTTTCAAAACGTGCCGTAGATTATTGTAAAGGCCTTCCCTTAGCTCTTGAAGTTTTAGGTTCCTTCCTTCATTCTATTGGTGATCCCTCCAATTTTAAACGTATATTGGATGAATATGAAAAACATTACCTCGATAAGGACATCCAAGACTCTCTTCGAATAAGTTACGATGGACTCGAAGATGAAGGAATAACTAAACTTATGAATCTATCACTTCTTACCATTGGTAGATTCAACAGAGTTGAAATGCATAACATAATACAACAAATGGGTCGGACAATTCATCTTTCAGAAACTTCTAAATCTCACAAAAGAAAAAGATTGTTGATTAAAGATGATGCTATGGATGTTTTAAATGGGAATAAGGAAGCAAGAGCAGTGAAAGTCATAAAACTAAATTTTCCTAAACCTACGAAGTTGGACATCGATTCAAGAGCTTTTGATAAAGTGAAAAATTTGGTAGTACTGGAAGTTGGCAATGCCACATCTTCAGAAAGTTCTACTCTTGAGTATCTACCCAGTAGCTTAAGGTGGATGAATTGGCCTCAATTTCCTTTTTCATCTTTGCCTACAACCTACACAATGGAGAACCTTATTGAATTGAAATTGCCATATAGCTCCATCAAACATTTTGGTCAAGGATATATGAGTTGTGAAAGGTTGAAGGAAATTAATCTTAGTGACTCCAATCTTTTGGTGGAAATCCCAGATTTATCTACTGCAATAAACCTCAAATACTTGAATCTTGTAGGATGTGAAAATTTAGTAAAAGTTCATGAATCAATTGGATCTCTCAGTAAGCTTGTCGCCCTTCATTTTTCTAGTAGTGTTAAGGGCTTTGAGCAGTTTCCATCCTGCCTCAAGTTGAAATCTCTTAAGTTTTTGTCAATGAAAAATTGTAGAATAGATGAATGGTGTCCCCAATTCAGTGAAGAAATGAAGTCTATAGAATATTTGTCGATTGGGTACAGTACTGTAACATATCAGCTATCTCCAACAATTGGATATCTTACTAGCCTAAAACATTTGAGCCTCTATTATTGCAAAGAGCTCACAACTCTTCCAAGTACAATTTATCGTTTAACCAATCTTACTTCTTTAACTGTGTTGGATTCTAATCTTTCAACATTTCCTTTCTTAAATCATCCGTCCTTACCTTCCTCACTTTTTTACCTAACCAAGTTACGTATTGTTGGTTGCAAGATAACAAATTTGGATTTCTTAGAAACAATTGTTTATGTCGCCCCTTCATTGAAAGAGTTGGACTTGTCCGAAAACAACTTTTGTAGATTACCGTCATGTATTATTAATTTTAAATCTCTAAAATATCTTTATACGATGGATTGTGAGTTGCTTGAAGAAATTTCAAAGGTTCCCGAAGGCGTAATTTGTATGAGTGCTGCTGGGAGCATATCATTGGCTAGATTTCCTAACAACTTGGCTGATTTCATGTCTTGTGATGATTCTGTGGAATATTGTAAGGGTGGAGAATTGAAACAACTGGTATTAATGAATTGTCATATTCCAGATTGGTATAGGTACAAGAGCATGAGCGATTCATTAACATTTTTTTTGCCAGCTGATTATCTAAGTTGGAAATGGAAGCCTTTGTTTGCTCCTTGTGTCAAATTTGAAGTTACGAATGATGATTGGTTCCAGAAGCTTGAATGTAAAGTTTTTATCAACGATATTCAAGTATGGAGTTCTGAAGAGGTGTATGCCAATCAGAAGGAACGGAGTGGGATGTTTGGAAAAGTATCACCAGGTGAGTATATGTGGCTGATAGTACTTGATCCTCATACACGTTTCCAATCATATTCGGATGATATCATGGACAGGAGGTCACTGAAGATTATTGATCTAAATCAACTAAGTTCTGAGATTAATTCCTCACAAAGTATTTTGGGTAAAATTACGGTGTCATTTGAGGTTACTCCATGGTATAAAGACGTAGTTATAAAAATGTGTGGTGTTCATGTCATCATGGGGGAATGA

>Csa006757

ATGAATCGAGCAACTGGATCGTCTTCCTCGCATCTTAGGTTGCCTTTTGATGTATTCTTAAGTTTTAGAGGGGAAGATACTCGTTCCAACTTCACGAGTCATCTTCATATGGCTTTGTGTCAAAAGGGTATCAATGTTTTCATAGATGATGACAAGCTTCCAAGGGGTGAAGAAATTTGTACATCTCTTTTGAAAGCCATCGAAGAATCGAAAATCTCGATTGTTATAATTTCAGAAAATTATGCATCTTCCCATTGGTGTTTGGATGAACTGATGAAAATCATTATGTGTAACAAATCCAATAATCGACAAGTCGTTTTTCCTGTTTTTTACAAAGTGAATCCATCTCATGTACGACGACAAAGGGGAGTATTTGGAGAAGAATTTGCCAAACTCCAAGTTAGGTTCTCCAACAAGATGCAAGCATGGAGTGAGGCTCTAACTTTCATCTCCACTATGTCTGGATGGGATCTAAAAAACTATGAAAATGAAGCAAGTTTGATTCAAATAATTGTTCAAGAAGTCAGAAAGAAATTAAGGAATAGTGCAACAACCGAGTTAGATGTAGCTAAATATCCAGTTGGAATTGACATACAAGTTAGTAATTTACTGCCACATGTTATGTCTAATGAAATTACTATGGTTGGATTGTATGGAATTGGAGGTATGGGCAAGACAACTTTGGCCAAAGCTTTATACAATAAAATTGCTGATGAGTTTGAAGGTTGTTGCTTTCTGTCAAATGTTAGAGAAGCTTCAAATCAATACTGGGGTCTTGTTGAACTCCAAAAGACGCTACTTCGTGAGATTCTGATGGATGATTCAATCAAAGTTAGCAATGTTGGTATAGGAATTAGCATCATAAGGGATCGATTATGCTCAAAAAAGATAATTTTGATTCTTGATGATGTTGATACACATGAACAACTACAGGCATTGGCTGGAGGACATCATTGGTTTGGACATGGAAGTAAGGTCATTGCAACAACAAGAAACAAGCAATTACTTGCTAGTCATGGGTTTAATATATTGAAAAGAGTTAACGGATTAAATGCGATTGAAGGTCTTGAGCTTTTTAGTTGGCATGCATTCAACAATTGTCATCCCTCAAGTGATTATTTAGATGTTTCAAAACGTGCTGTGCATTATTGTAAAGGTCTTCCCTTAGCACTTGAAGTGTTAGGTTCCTTCCTTAATTCTATCGATGATCAATCCAAGTTTGAACGTATATTGGATGAATATGAGAACTCATACCTGGACAAAGGCATCCAAGATATTCTTCGAATAAGTTATGATGAACTTGAACAAGATGTAAAAGATATTTTCCTTTACATTTCTTGTTGCTTTGTACATGAAGACAAAAACGAGGTTCAAATGATGTTAAAAGAATGTGACTCTCGTTTCAGATTGGAAATGGGTATTAAAAAACTCACTGATCTATCACTTCTTACAATTGATAAATTCAACCGGGTCGAAATGCATGACTTGATACAACAAATGGGTCACACAATTCATCTCTTGGAGACTTCTAATTCTCATAAAAGAAAAAGATTGTTGTTTGAAAAAGACGTCATGGATGTCTTAAATGGAGATATGGAAGCAAGGGCTGTGAAAGTCATAAAGCTAAATTTTCATCAGCCCACTGAGCTAGACATTGATTCAAGAGGTTTTGAAAAAGTGAAAAACTTGGTAGTGCTCAAAGTTCACAACGTCACATCTTCAAAAAGTCTCGAGTATCTACCGAGTAGCTTAAGGTGGATGATTTGGCCTAAATTTCCGTTTTCATCTTTGCCTTCAACCTACTCACTGGAGAAACTTACTGAACTCAGCATGCCAAGTAGCTTCATCAAACATTTTGGAAATGGATATCTGAATTGCAAATGGTTGAAGCGTATAAATCTTAACTACTCAAAGTTTTTAGAAGAAATTTCTGATCTATCCAGTGCGATAAATCTTGAAGAGTTGAATCTTTCCGAGTGTAAAAAGCTGGAATATGCAGATGGAAAATACAAACAACTCATATTAATGAATAATTGTGATATTCCAGAATGGTTTCATTTCAAGAGTACGAACAATTCAATAACGTTTCCTACCACATTTAATTATCCGGGTTGGAAATTGAAAGTTCTTGCTGCTTGTGTTAAAGTTCAAGTTCATGATCCTGTTAATGGGTATCATAGAGGGGGGGATCTTGAATGTGAAGTGTTCTTTAAGGACATTCTAGTATGGAGTTCTGGAGACTGGACAAATTATCTTGGATACGATTCAAGATGGTTGCCCCTAGGAGCATCACCAAGTGAGTATACATGGTTTATTGTACTCAATCCTCATAGAGATTTCTCCCTAGATGATTGGGATGATACGATGGAGAGATCACCAGAGACTGATCTAAGTCAGCTATGTTTTGGAATTAATTCCATGGAAATGGACCGTAATAGATCAAATGATAAATGGAATTCTATTGGGGGAAGTATTTGGAAGAACTTTACGGTGTTGTTTGAGCCTCGACCCCTGTCTCGAGACACTATAATAAGTATAAAAGGTTGTGGTGTTCATGTGATCATGGAGTAA

>Csa006758

ATGTGTAAGGAATTGAGAGGACAAGTTGTTTTACCAATTTTTTACAAAGTGAATCCATCTCAAGTACGGAAACAAAATGGAGCATTTGGAGAAGCATTTGCCGAACTTGAAGTTAGATTCTTTGACAAGATGCAAGCATGGGGAGAGGCTTTGACTGCTGTTTCCCATATGTCTGGATGGGTGGTTCTTGAGAAAGATGATGAGGCTAATTTGATACAAAAAATTGTTCAACAAGTCTGGAAGAAATTAACTTGTTCAACAATGCAGTTGCCTGTAACTAAATATCCAGTTGGAATTGACAGACAATTTGAGAATCTGCTCTCTCATGTTATGATTGATGGAACTAGAATGGTTGGATTGCATGGAATTGGAGGTATGGGCAAGACAACTTTGGCCAAAACATTATACAATCGGATTGCTGATGACTTTGAAGGCTGTTGTTTTTTAGCAAATATTAGAGAAGCTTCAAAGCAACACGAGGGACTTGTTCGACTCCAAGAGAAACTACTTTATGAGATTTTAATGGATGACTTTATTAGAGTTAGTGATCTTTACAAAGGAATCAACATCATAAGGAATCGACTATGCTCCAAAAAGATTCTCTTAATTCTTGATGATATAGATACCAGTGAACAACTACAGGTATTAGCTGGAGGATACGATTGGTTTGGATATGGAAGTAAGGTCATTGTGACAACAAGAAACGAACACTTACTTGATATCCATGGATTTAATAAATTGCGAAGTGTTCCTGAATTGAATTATGGTGAAGCTCTTGAGCTTTTTAGCTGGCATGCTTTTCAGTGTAGTAGTCCACCAACCGAGTATTTACAACTTTCAAAAGATGCTGTAAATTATTGTAAAAATCTTCCCTTGGCGCTTGAAGTTTTAGGTTCATTCCTTTATTCAACTGATCAATCCAAATTTAAAGGTATATTGGAGGAATTTGCAATCTCCAACCTTGACAAAGACATCCAAAATCTTCTTCAAGTAAGTTACGATGAACTTGAAGGTGATGTACAAGAAATGTTCTTGTTTATTTCTTGTTTCTTTGTGGGAGAAGATAAAACCATGGTTGAAACGATGTTGAAGAGTTGTGGTTGTTTATGTTGGGAAAAGGGAATTCAAAAACTCATGAATCTATCACTTCTTACTATTAACCAATGGAACAAAGTTGAAATGCATGACTTAATACAACAATTGGGTCACACAATCGCACGTTCAAAGACTTCTATATCTCCTTCAGAAAAAAAATTATTGGTTGGAGATGATGCTATGCATGTGCTAGATGGCATTAAGGATGCAAGAGCAGTTAAAGCCATAAAGTTAGAATTTCCTAAACCGACAAAGTTGGACATTATTGATTCAACGGCTTTTAGAAAAGTAAAGAACCTTGTAGTACTCAAAGTGAAGAATGTCATATCTCCAAAAATTAGTACTCTTGATTTTCTACCTAATAGCTTAAGGTGGATGAGTTGGTCTGAATTTCCTTTTTCATCATTTCCTTCAAGCTACTCAATGGAGAACCTTATTCAACTCAAATTGCCACATAGCGCCATCCAACATTTTGGAAGAGCATTTATGCATTGTGAAAGGTTGAAGCAACTTGATCTTAGCAACTCCTTCTTTTTGGAGGAAATTCCTGATTTATCTGCGGCAATAAACCTCGAAAATTTGTCTCTTTCTGGATGTATAAGTTTAGTAAAGGTTCATAAATCAGTTGGATCTCTTCCTAAACTTATTGATTTGAGTCTTTCAAGCCATGTTTATGGCTTTAAGCAGTTTCCTTCACCACTCAGGTTGAAATCCCTTAAAAGATTTTCAACTGATCATTGTACAATACTTCAAGGCTATCCTCAATTCAGCCAAGAAATGAAGTCTAGTCTAGAAGATTTGTGGTTTCAAAGTAGTTCTATAACAAAGCTATCTTCAACAATTAGATATCTTACCAGCCTCAAAGATTTGACCATCGTGGATTGCAAAAAGCTCACTACTCTTCCAAGTACAATTTATGACTTGAGCAAACTTACATCCATAGAAGTCTCACAATCCGATCTTTCAACATTTCCTTCCTCATATTCCTGCCCTTCCTCACTTCCCCTCCTAACAAGATTACACCTTTATGAGAACAAGATAACAAATTTAGATTTTTTGGAAACTATCGCTCATGCTGCTCCATCACTGAGAGAGTTGAACTTGTCTAACAACAACTTTTCTATACTACCTTCATGTATTGTTAATTTTAAATCCTTGAGATTTCTTGAAACATTTGATTGTAAGTTTCTGGAAGAAATTCCAAAGATTCCAGAAGGCTTAATTTCTTTGGGTGCATATCATTGGCCAAATCTCCCGACAACTTAG

>Csa006768

ATGGGTTCTTCCATTGTTGGAGCTGAATCATCAACTTCTTCTTCTTCTAGTTTCAAGTGGAGTTTTGATGTGTTTTTGAGTTTTAGGGGAGATGATACTCGTTCTAATTTCACCGGTCATCTTGATATGGCCTTGCGTCAAAAGGGTGTCAATGTCTTCATAGACGACATGCTCAAAAGGGGTGAGCAAATTTCTGAAACCCTTTCCAAAGCTATACAGGAAGCTTTGATTTCTATTGTTATTTTCTCTCAAAATTATGCATCTTCTTCATGGTGTCTGGATGAATTGGTGAAAATAGTTGAGTGTAAGAAATCCAAGGGCCAGCTTGTTTTGCCAATTTTCTACAAGGTGGATCCTTCCGATGTACGAAAACAAACTGGTTGCTTTGGAGAAGCATTGGCCAAACATCAGGCTAATTTCATGGAGAAGACTCAAATATGGAGGGATGCTTTAACTACTGTTGCCAACTTCTCTGGTTGGGATCTAGGAACTAGGAAGGAGGCTGATTTTATTCAAGACCTTGTTAAAGAAGTATTGTCTAGATTAAATTGTGCCAACGGGCAGTTATATGTAGCTAAGTATCCAGTTGGAATTGATTCTCAACTAGAAGATATGAAGTTACTCTCGCATCAGATACGAGATGCGTTTGATGGCGTTTACATGATGGGGATATACGGCATTGGAGGCATTGGTAAGACTACTTTGGCTAAAGCTTTGTACAATAAAATTGCTAACCAATTTGAAGTTCTTATAGTTCTTGATGATGTGGATAAGCTCAAGCAATTGGAAGCATTGGTTGGTGAACGTGATTGGTTTGGCCATGGTAGTAAAATCATTGTGACAACAAGAAATAGTCATTTACTTTCTAGCCATGAATTTGATGAAAAGTATGGTGTTCGGGAATTGAGTCATGGTCATTCCCTTGAACTTTTTAGTTGGCATGCTTTTAAGAAAAGTCATCCATCAAGTAATTACTTAGACCTTTCAAAACGTGCGACAAATTATTGTAAAGGTCATCCTTTGGCCCTTGTTGTTTTGGGTTCTTTCCTTTGTACCCGAGACCAAATAAAATGGAGAACTATATTAGATGAATTTGAGAACTCTTTGAGTGAAGACATTGAACATATTATTCAAATCAGTTTTGATGGGCTTGAAGAAAAAATAAAGGAGATCTTCCTTGATATTTCTTGTTTGTTTGTGGGAGAGAAAGTTAATTATGTTAAGAGTGTGTTAAATACGTGTCATTTCAGCCTAGATTTTGGAATCATAGTTCTCATGGATCTTTCACTTATTACGGTTGAAAATGAAGAGGTTCAAATGCATGATTTAATTCGACAAATGGGCCAGAAAATAGTTAATGGTGAATCTTTTGAGCCCGGGAAAAGGAGTAGGTTGTGGTTGGTACATGATGTTTTGAAGGTGTTTGCTGATAATTCGGGAACGATTGCAGTTAAAGCCATAAAGTTAGACTTGTCTAATCCCACGAGGCTAGACGTGGATTCACGAGCTTTTAGGAACATGAAGAATCTGAGGTTGCTTATTGTTCGAAATGCAAGATTTTCGACAAATGTTGAGTATCTACCTGATAACTTGAAGTGGATTAAGTGGCATGGCTTTTCTCATCGATTTTTGCCACTGTCCTTCCTTAAGAAAAATCTTGTAGGACTAGATTTGCGTCATAGCTTGATCAGAAATTTGGGCAAAGGATTTAAGGATTGTAAAAGGTTGAAGCATGTTGATCTTAGTTACTCTTCTTTATTAGAGAAGATTCCCGACTTCCCTGCAACATCAAATCTTGAAGAATTATATCTTAACAACTGCACAAATTTAAGAACAATTCCTAAGTCAGTTGTTTCTCTTGGTAAGCTTCTTACTTTAGACCTTGATCATTGTTCAAACCTTATAAAGCTTCCAAGCTACCTCATGCTGAAGTCTCTTAAAGTTTTGAAGCTTGCTTACTGCAAAAAACTTGAGAAACTTCCAGACTTCTCTACAGCTTCAAACCTTGAAAAGTTGTACCTCAAAGAATGCACAAATTTAAGAATGATTCATGATTCTATTGGATCTCTGAGTAAGCTTGTTACCTTGGACCTTGGAAAATGCTCTAACCTTGAAAAGCTTCCAAGCTACCTCACATTAAAGTCTCTTGAATATTTGAATCTTGCTCATTGCAAAAAGCTTGAGGAAATTCCCGACTTCTCTTCTGCATTAAACCTTAAAAGCTTATATCTTGAACAATGCACAAATTTAAGAGTAATTCATGAGTCTATTGGATCTTTGAATAGTCTTGTTACCTTGGACCTTAGACAATGCACTAACCTTGAAAAGCTTCCAAGCTACCTCAAGTTGAAGTCTCTTAGACATTTCGAACTCTCTGGCTGCCACAAGCTCGAAATGAATTGCAAGTTCCTTCAAGAAATTCCTAACCTCCCTCATTGTATACAAAAAATGGATGCCACTGGTTGCACATTGTTGGGTAGAAGTCCAGACAACATCATGGACATAATATCGAGCAAGCAGGACGTTGCACTCGGTGACTTTACAAGAGAGTTTATTCTAATGAATACTGGGATTCCAGAATGGTTCAGCTATCAGTCAATATCAAATTCAATAAGGGTTAGCTTTCGACACGATCTCAATATGGAACGAATTTTGGCTACATATGCTACTTTGCAAGTGGTTGGAGATTCATATCAAGGAATGGCCTTAGTTTCATGTAAAATATTCATTGGCTACAGACTCCAAAGTTGTTTTATGAGAAAATTTCCATCATCAACATCAGAATATACATGGTTAGTAACAACTTCTTCTCCAACATTTAGCACTTCCTTGGAGATGAATGAGTGGAATCATGTCACAGTCTGGTTTGAGGTTGTGAAATGTTCTGAGGCCACCGTAACTATAAAATGCTGTGGTGTCCATCTCACTGAAGAGGTCCATGGAATACAAAATGATGTCAAGGGGCCAGGGGTAGTTTATACAGTTTTCGATCAACTGGACAAATTACCGAGCCGGGATGTTATAAAATCCTTTGGTCAAGAAGTATCTGCCAAATCAGATTGCAATGCAATGTTGCATGCAGAAAATTTTCCAGTTTGGAACGATTCGAAAATGCAACAACATATGAATTTTCCCTTACATGTAACTTCTCAAGGTGTTACAAGGATACGTGGTATGGAAGGCATGGCAGAGACAACACTTGCCAACTCTATATGTAACAAATATGAAAGAAGTCGGAATCTTTTCTCTGCAAAAAAAGCTTTGAATCATTCTACTGGCTTTCTTTGTGGAGATGGAAATGGGCTTTCTTGGGAAATGGTAGACAGACCGATATTAAGTGATAGATTGTCTTCCCAAAAATATCTTAGAATTTTCGACGATCGTGATCGATATGGAGACCTAAATGATGTGGCTCATGGGACTGGTAATAGGTTTCGTTCAAGATTTTTAAGGATGGATGATATAAAAGAAGATGATATCAGAGAAGAGCCTTATTGGAAGTACATGGAAAGGTTATTCCAAACAGATCCTATATCATGA

>Csa006769

ATGAGGAAGTCTTTGGGTAGATTTGGGATGGGTTCTAACGCTGCTGGAGCGGAATCGTCGTCTTCTTCTCCAATCAATTGGATTTATGATGTGTTTTTGAGTTTTAGAGGAGAGGATACTCGCTCCAATTTCACAAGTCATCTTCACATGTTCTTGCGTCACAAGGGTGTCAATGTTTTCATAGATGACAGGATCGAAAGGGGTGAGCAAATTTCTGAAGCCCTTTTAAAAACTATACAGTGTTCTTTGATTTCTATTGTTATATTCTCGGAAAATTATGCATCTTCTACATGGTGTCTGGATGAATTGGTGGAAATAATTGAGTGTAAGAAATCCAAGGGTCAGAAAGTATTGCCAATTTTCTACAAGGTGGATCCTTCGGATGTACGAAAACAAAATGGTTGCTATGGAGAAGGATTGGCCAAACATGAGGCTAATTTCATGGAGAAGATTCCAATATGGAGGAATGCTCTAACGACTGCTGCCAACTTGGCTGGTTGGGATCTCGGAACAATAAGAAATGAGGCTGACCTTATTCAAGTTATTGTTAAAGAAGTGTCGTCTACATTAAATGTCACCACGCCCTCAGATAAGCCTCTACTAGTTGGAATTGATTCCAAAATTGAATCCCTTTATTGGCCTACAGAAGAAATGTACAAGTCTGAATGTGTTGACATGTTGGGGATATATGGCATTCGAGGCATTGGTAAAACAACTTTGGCTAAAGCTTTATACAACAAAATTGCTAGCCAATTTGAAGGTTGCTGCTTTCTATCAAATGTTAGAGAAGCTTCAAAGCAACTCAATGGCCTCGCTCAACTACAGAAAAAACTACTTTTTCAGATCTTAAAGTATGATTTGGAGGATGTCGATGATCTTGACAGGAGAAATAATATCATAAAGCATAGACTCCATTCGAAGAAAGTTCTTATACTTCTTGATGATGTGGATGAGATGAAGCAATTAAAAGCATTGGCTGGTGGGCATGATTGGTTTGGTCAGGGTAGTAAAATCATTGTGACGACTAGAGATAAACATTTACTTGATAGCCATGGATTTGGTCAAACATATGAAGTTGAAGGATTGTGGGAACACAATGCATTTGAGCTTTTTTGTTGGCATGCCTTCAAAAAAAGTCATCCATCTAGTAATTATTTAGACCTTTCAGAACGTGCTACAAGGCATTGTAAAGGTCATCCTTTGGCTCTTGTTGTTTTGGCTTCTTTCCTTTGTGGCAGAGATCAAGCAGAATGGAGTGGTCTATTAGATGGATTTGAAAACTCTTTGAGAAAAGGTATTAAAGATGTTCTTCAATTAAGTTTTGATGGGCTGGAAGACGAAGTAAAGAAATTTTTTCTTGATATTTCTTGTTTACTCGTGGGTGAGACAGTTACCTATGTTAAGAAAATGTTGAGTGAATTCCATTCGATTCTGGATTTCAAAATTAGCAATCTGAGGCATCTTTCACTTATTAGGATGGAAGAATATGATGATGATAGGGTGCAAATGCATGATTTAATAAAACAAATGGGTCATAAAATAGTTTATGATGAATGTGGTGATGAGCCTGGAAAAAGGAGTAGATCAGGTTGGAGGAGGACATTCTGGAGGTGTTTAGTAACAATTCAGTAA

>Csa006843

ATGGAAGTTGAAAGCATAGTTCAAGATGTGATTGAGGCTAGTCAACAACAACTCAATTCTATTTTACCCGTTTATGGAACGGGTGGATCAGGAAAAACCACTTTGGCCCAGTTGGTGTTTAATGACGAGAGGATTGGAAAACAATTTCATCATACTGTTTGGGTATGTGTGTCTCAACCTTTTGTCATCAACGAGATCTTGCAGTCAATCTTGAAAAAGGTAAGCAAAAGCAACGATAATCGTAGCAAGGATGATAAGGACACCTTAATTCGCAATCTTAAAGAAGTGATGGGTGGAAAAAGATATTTTCTTGTGCTTGACAATGTTTGGAATGAAAACAAAATGTTATGGGAGAAGTTGAAGGAATGCTTAATGAGTATTGTTGAAGAATTAGGAAGCAGTGTCCTTGTCACGACCAGGAGTCGTAAAATTGCAGAAATGATGAAAGAAACACTTGACACCTATCATTTAAACAAATTAACTGATGATCAATGTTGGTCAGTATTTAGCTACTTTGCCAAGGCGAATGCAGTACCAATAACTTCCAATTTGGAGCTTGTGCGAGAAGAGGTCGTTAAAAAAATTGGTGGCCTTCCATTACTTGCTAGAGTTTTGGGAGAAGCCGCTCAATTTTCTGGAGACTACGAAAAATGGGTGGAAATTTTAAAATCTATTCCAACAACTCCATTAAAATATGAAGAGTCGTCATATGTTAAATACATATTGAAGTTAAGCGTGGATCGTCTACCAAAAGCTTCAATAAAGCAATGTTTTGCTTACTGTTCAAATTTTCCTAAAGGTTATTGGTTTGACAAAAAACAAGTGATTAAAATGTGGATGGCACACGGGTTTACTCGACCAGATGAAGGAAATAATGAAACAATGGAGGATACAGGAAAGAGGTACTTCAATATCTTATTGTCTTATTGCTTATTTCAAGATGCTGATGATGACAAATGGCATATTGGTAGGAAGTTTCGTATGCATGATCTTATACACGATATTGCTTGTGATGTTTCAAGCGATAAAAGGTTGCAATTAGATCATAGCAGTTCATCAAAGTGGAAAGTAGGATTTGAGACGGGTCGTAAGATTTCTGAACTTGGGGGTCTTAGAAACTTGAAAGGTTTGTTGAAGCTTCATCGTTTAGAACATGTTGAAAGTAAAGAGGAAGCCAAGGCTGCAAAATTGGTGGAAAAAGAGAAGGTAGAAGGACTAAACTTGTCGTGGCGTGGAAAGTGGAAGAATAGTGGAGAATCAGAATGA

>Csa007358

ATGGCCGAATTTATAATAAATGTTGCGTCAGTAATTGTAACACCAATAGGAAAGTATGTGATTAAACCAATTGGAAATCAACTTGGTTACATTGTTTTCTACAACAGAAACAAGAATGAGATTAAAGAGCAACTTGAAAGTCTTGAGACTACTAAAAAGGATTTGGATCTAAGGGTTGAAGATGCAAAAAGCAAGGCATATACCATCTTTACGAAAGTTTCAGAGTGGTTGGTCGCTGCGGATGACGAAATAAAGAAATCTGATGAGCTATTCAATTCCAACCCACCTTGCCTTAACTTTCTCCAACGACACCAACTAAGTAGAAAGGCAAGGAAGAGGGCGACGGATATCCGCCGACTCAAAGACGGAGGAAACAACTTTCTGGAAGTTGGTTGTCCTGCCCCTTTACCGGATACTATGAATACTATTGTTCCTGAAGCTTATCAAACTTTAGGATCAAAAACCTCAATGGCCAAGCAAATTAAGGACGCCCTTGCAAAACCTGAGGTAAGAAAGGTTGGAATCTATGGTATGGGAGGTGTTGGAAAAACATATTTGCTCAAGGAAGTTAAGAAATTGGTGTTGGAAGAAAAATTGTTTGATCTAGTGATTGATGTGACTGTAGGTCAATCTAATGATGTAATGAATATGCAACAACAAATTGGAGACTTCCTCAATAAAGAATTGCCAAAGAGTAAGGAGGGAAGAACATCCTTTCTACGAAATGCATTGGTGGAAATGAAAGGTAATATCCTGATCACATTCGATGATTTATGGAATGAATTTGATATCATAAACGATGTTGGAATTCCGTTAAGTAAAGAAGGATGTAAGACACTTGTCACAAGTCGTTTTCAAAATGTTCTAGCCAATAAAATGAATATAAAAGAGTGTTTTAAGGTGACTTGTCTAGACGATGAAGAGTCTTGGAAGTTTTTTAAGAAAATTATTGGTGATGAGTTTGATGCAAAAATGGAAAACATTGCAAAGGAAGTGGCCAAACAATGTGGAGGATTACCACTTGCACTTGATATCATTGCAAAAACATTAAAGAGATCAAGACATATAAATTATTATTGGGAGGGAGTGTTAAGTAAGCTGAAAAATTCAATTCCGGTGAATATTGACGTGGGTGAAAAAGTTTATGCTTCACTTAAACTAAGCTATGAACATTTGGATGGAGAAGAAGTCAAATCACTATTTCTTCTTTGTAGCGTATTTCCAGATGATCATGGGATTTCAGTAAACGATCTGCAAATGTATGTGATGGGTATGGGACTATTGAAAATGGTAAATACTTGGAAGGAAGCAAGAGCTGAAGCACATTACTTGGTCGAGGATCTTACATCATCTTCTTTACTTCAACGACTTAAGAATAGAGATGTTAAAATGCATGATATAGTTCGTGATGTTGCAATATACATTGGACCAGACTTTAACATGTCTACACTTTACTATGGATATAGTACAAGTAGCAAAGGGCTAGATGAGGATAAATGTAGATCTTATCGTGCAATCTTTGTAGACTGTAAGAAGTTTTGCAACCTTCTTCCAAACTTGAAGCTTCCAAAACTAGAATTGTTAATATTAAGTTTTCCTTTTTGGGGGAAAGATAGAAATATTGACATTATGGATGCATATTTTGAAGGAATGGAAAATCTTAAGGTTTTGGACATTGAAGGAACAAGTTTCCTTCAACCATTTTGGACACCGTTAAAGAACCTTCGAACGTTATGTATGTCATATTGTTGGTGTGAGGATATTGATACAATTGGGCACTTAAAGCAATTGGAAATTTTGAGGATTAGTAATTGTAGAGGCATCACAGAATTACCAACGTCTATGAGTGAATTGAAACAACTTAAGGTATTAGTTGTGTCGCATTGCTTCAAGTTGGTGGTGATTCACACAAACATTATTTCAAGCATGACCAAATTAGAAGAGTTGGATATACAAGACTGCTTTAAGGAATGGGGAGAAGAAGTAAGGTACAAGAACACATGGATTCCAAATGCACAACTTTCAGAATTGAATTGTCTGTCACATCTTTCTATTTTAAGAGTACGTGTTTTGAAGCTTACCATTCTCTCCGAGGCTTTGAGTTCACAAATGTTGAAAAACCTAAGAGAATTCTTTATTTATGTTGGTACCCATGAGCCTAAGTTTCATCCTTTTAAATCATGGTCGAGTTTTGATAAATATGAAAAAAATATGTCCTTTAATATGAAATCGCAGATTGTTTCAGTCAACCCGACGAAACTTAGCATATTATTAGAAGGAACTAAAAGGTTGATGATTCTAAATGACTCCAAAGGTTTTGCAAATGATATTTTCAAAGCAATTGGAAATGGTTATCCCCTGTTGAAGTGTCTTGAAATTCACGATAATTCAGAGACACCACATTTGAGAGGAAATGATTTCACATCTTTGAAGAGGTTGGTTCTTGATAGAATGGTGATGTTGGAGAGTATTATTCCGAGGCATTCTCCAATAAATCCTTTCAACAAACTTAAATTCATAAAAATAGGAAGGTGCGAGCAGCTAAGGAATTTTTTTCCACTCTCTGTTTTTAAAGGGCTTTCAAATCTTCGACAGATTGAGATCTATGAATGTAATATGATGGAGGAGATTGTATCAATAGAAATTGAAGATCATATCACTATTTACACTTCTCCTTTGACATCTTTACGCATCGAGCGTGTGAATAAACTTACAAGTTTTTGCAGTACCAAATCATCCATCCAACAAACAATTGTTCCCTTATTTGATGAACGACGGGTTTCATTTCCTGAATTGAAGTATTTATCAATTGGTAGAGCAAACAATTTGGAGATGCTATGGCATAAGAATGGAAGTTCCTTTTCCAAACTTCAAACAATAGAGATTAGTGATTGCAAGGAGTTGAGATGCGTGTTTCCTTCAAATATAGCGACGTCACTTGTCTTTTTAGATACATTGAAAATCTATGGTTGTGAGTTATTGGAAATGATATTTGAAATTGAAAAGCAGAAGACTTCGGGAGATACAAAAGTAGTGCCATTGAGATACTTATCTTTAGGATTTCTAAAAAATTTAAAGTACGTGTGGGACAAAGATGTTGACGATGTTGTGGCATTTCCAAACCTAAAGAAAGTTAAGGTTGGTAGATGCCCTAAGTTGAAAATTATTTTTCCAGCTTCCTTCACCAAATATATGAAAGAAATTGAAGAGTTAGAAATGGTTGAGCCGTTTAATTATGAAATATTTCCAGTGGATGAAGCATCAAAGTTAAAAGAGGTTGCATTGTTCCAAAGCTTGGAAACATTGAGAATGAGTTGTAAGCAGGCTGTAAAAGAGAGGTTTTGGGTTATGTCAAAGTTCTTCAAACTCAAAAGTCTTGAATTGTTTGGTTGTGAAGATGGTAAAATGATTAGCTTGCCGATGGAAATGAATGAAGTATTATACAGCATTGAAGAATTGACAATTAGAGGATGCCTCCAGCTGGTAGATGTAATTGGAAATGACTATTATATCCAAAGATGTGCAAATTTGAAGAAGTTAAAATTGTATAATCTTCCGAAGCTTATGTACGTGTTGAAGAACATGAATCAAATGACTGCAACCACATTCTCCAAGTTGGTTTATCTTCAAGTAGGTGGTTGCAATGGAATGATAAATTTATTTAGTCCTTCAGTGGCAAAGAATCTAGCGAATCTCAATTCCATTGAAATATATGATTGTGGAGAAATGAGAACCGTAGTTGCAGCAAAAGCAGAGGAAGAAGAGGAAAATGTTGAAATTGTGTTCAGCAAGCTAACTGGTATGGAATTCCATAATTTAGCAGGATTGGAATGTTTTTACCCTGGAAAATGCACACTTGAATTCCCCTTATTAGATACGTTGAGGATAAGCAAATGCGATGACATGAAAATCTTTTCATACGGAATAACAAACACTCCCACTTTGAAAAACATCGAGATTGGAGAACATAACTCATTGCCAGTATTACCAACACAAGGGATAAATGACATTATCCATGCTTTTTTCACAATTGAGGTATTCTTCACTAAGGTCTTCTCATGTACATTAGAGTCGTGCTCCCCTTGA

>Csa008307

ATGGCTGAATTTTTATGGACTTTTGCTGTTCAGGAAGTTTTGAAGAAGATTGTGAACTTTGGAGCAGAGCAAATTAGTTTGGCATGGGGTTTGGAGAAGGAGTTGTCCCACTTGAAAAAGTGGTTACTCAAAGCGCAAACAATCTTAGCAGACATTAACACAAAGAAATCACACCATCATTCTGTTGGGTTATGGGTGGAAGAACTTCATGATATTATCTATGAAGCTGATGATTTGTTAGATGAGATTGTTTATGAACAAATTCGACAAACTGTGGAGCAAACTGGTAAACTTAGAAAGGTACGTGATTCTATCTCACCATCCAAAAATTCCTTTTTGTTTGGTCTCAAGATGGCCAAGAAAATGAAGAAGATTACCAAAACTTTATACGAACATTACTGTGAGGCAAGTCCTTTAGGACTAGTTGGTGATGAATCCACCACAGAATCAGAGGCTGCACTTAATCAGATTCGGGAGACAACCTCAATTCTTGACTTTGAAGTTGAAGGAAGGGAAGCTGAAGTCTTGGAGATACTAAAATTGGTGATTGACTCTACCGATGAAGATCATATCTCTGTGATATCCATTGTTGGAATGGGTGGTCTTGGAAAAACAACTTTGGCCAAGATGGTTTTCAATCATGATGCCATTAAAGGACATTTTGATAAAACTGTATGGGTTTGTGTGTCTAAACCATTTATTGTGATGAAAATTTTGGAAGCAATCTTTCAAGGTTTAACGAATACTAGTAGTGGTTTGAACTCCAGGGAGGCCTTGCTTAATCGACTCCGAGAGGAGATGCAAGGAAAAAAGTATTTTCTTGTGCTTGACGATGTTTGGGATAAAGAGAATTGCTTGTGGGACGAGCTTATTGGCAATTTGAAATATATTGCTGGAAAATCTGGAAATAGTATTATGGTGACCACAAGGAGTGTAGAAGTAGCGACCATGGTGAAGACAGTTCCCATTTATCATCTAAAAAAATTATCGGATGATCATTGTTGGGCGTTGTTAAAAAAAAGTGCAAATGCAAATCAGCTGCAGATGAATTCAAAGTTGGAGAATACGAAAAATATTTTGGTTAGAAAAATTGGTGGTGTACCACTCATTGCAAAAGTTTTAGGTGGGGCAGTAAAGTTTGAAGAAGGTGGGTCTGAGAGTTGGATGGCAAAAATTGAAAGCTTTGCGAGAAATATTTCAATAGAGGACAAAGATTTTGTTTTGTCCATATTAAAATTAAGTGTAGAGTCTCTCCCTCATTCTGCATTGAAGCAATGTTTTGCTTACTGCTCAAATTTTCCTCAAGATTATGAATTTGATAAAGATGAAGCAATCCAAATGTGGATAGCCGAAGGATTTATTCAACCCGAACAAGAAAGAGAAAACTTGACAATGGAGAACATAGGAGAAGAGTATCTTAACTTTTTATTGTCTCGCTCCTTATTTGAAGATGCCATTAAATATGATGGAAGAATTGTCACCTTTAAGATTCATGATCTAATGCATGATATTGCTTGTGCAATTTCAAATCATCATAAGATGGACTCAAATCCTATTAGTTGGAATGGAAAAAGTACAAGAAAGTTGCGCACATTAATTTGCGAGAATGAAGAAGCTTTTCATAAAATTCAGACTGACATTATTTGTTTGCGTGTGTTAGTCTTAAAATGGTTTGACACTAATACCTTGTCGACTATTATGGACAAATTGATACATTTGAGATATCTTGATATTTCAAACTGTAATATAAACAAGCTTCTTCGAGATTCTATTTGTGCACTTTATAATTTACAAACGCTAAAACTTGGATATATTGAATGTGATCTGCCGAAGAATTTGAGGAACTTGGTTAATTTGAGACATTTAGAATTTAAGAAATTTTTTGATATGGGACAAATGCCTTCACATATGGGCAACATGATTCATCTTCAAACACTATCTGAGTTTGTAGTTGGACTTGAGAAGGGTTGTAAAATTGATGAGCTTGGACCGTTAAAAGACCTCAAAGGTACACTAACTCTTAAAAATCTACAAAATGTGCAAAATAAAGACGAGGCTATGGCTGCAAAATTGGTGGAAAAGAAGTATTTACGTCATCTAATCTTTCAATGGTTTCTAAATCTTTATGATAGAGGAGAATATGATGAAGATGATAACAAACAAGTGTTGGAAGGACTTCAGCCACACAAAAACGTACAGTCATTGGACATTAGAGGCTTCCAAGGAAGAGTTTTGAATAATAATATTTTTGTTGAAAATTTAGTTGAGATACGTTTGGTTGATTGTGGAAGATGTGAAGTGCTTCCTATGCTTGGACAGTTGCCCAACTTGAAGAAACTTGAGATTATTTCAATGAACAGTGTGAGAAGTATAGGCAGTGAGTTCTATGGAGTTGACTGTAACGACAGAAATTCTTCTGCTTTTCCTCAGCTGAACAAATTTCATATTTGTGGGTTGAAGAAGCTACAACAATGGGATGAAGCAACGGTTTTTGCATCAAATCGCTTTGGATGTCTAAAAGAACTTATTCTTTCTGGATGTCATCAATTGGCAAAATTGCCAAGTGGGTTAGAAGGGTGCTACTCCATTGAATATTTGGCCATCGATGGGTGTCCTAATTTAATGCTAAATGTGCAAAATTTGTACAACTTGTATCATTTAGACATTCGTGGGTTGAAAAGATTGCCAGATGAATTTGGTAAGCTCACTAACTTGAAAAAATTGAGAATTGGTGGATGTATGCAAAACTATGAATTTAGTCCCTTCATACATTTATCTTCTCAGCTTGTTGAACTTGAGTTGACTGATGATGGGTCAAGTGGTAGTGAAACAACCCAACTTCCCCAACAACTTCAGCATCTGACCAACTTGAAGGTTTTGAAGATTGCAGATTTTGATGACATTGAAGTTCTACCAGAATGGTTGGGAAACCTTACATGTTTGGCAACATTGGTTTTCCTCGAATGCAAAAATTTGAAAGAGTTACCTTCGAGAGAGGCCATACAACGATTAACCAAATTAGATGATTTGGTGATCGATGGATGTCCCAAACTACTACTAGGGGAAGGCGATCAGGAGAGGGCTAAACTTTCTCATCTCCCATCAAAATGTGTTCGTTACAATAATTTTGGTTTTAGATGTTAG

>Csa008754

ATGGCTGAGGCCATTCTCTACATCGTTACTGCACACATCATTTTCAAATTGGGCTCTTTCGCGCTCCAAGAGCTTGGATCGCTATGGCGTGTCAATTGTGAACTTCACAAACTCAAAGACTCTCTTTCTGCCATTCAAGTCGTGCTTCACGACGCAGAGGAGCAACAATCGAAGAACAATCAAGTCAAAGATTGGGTTTTAAAGCTTGAGGATGTTTTGTATGAGATTGATGACTTGATCGATAAGTTCTCTTACCAAACCTTGAGAAGGCAAGTTATGGCCAAACACCAAAGGTACCGAAAACGAGTACGTATCCTCTTCTCCAAATTTAAATCTAATTGGGAAATAGGTTTCAAAATCAAGGAAATTAGGCCGGGGCTACTAGCTATTAATGAAGATAAAAATCAATTTAGCTTTACTAAGCATGTGATAGAGAGAAGAGATGATGATGAAGGGTTGAGAAAGAGTTGGGAGACTCACTCTTTTGAAGTGATTGGTAGGAATGATGACAAGGAAGCAGTCATAAATCTTCTACTAAATTCCAACACCAAAGAGGATATTGCAATAGCTTCCATCGTTGGAATGGCAGGATTGGGAAAGGCTGCCCTTGTCCAATCTATTTATAACTCTAAGAGGATTATGACTCGATTTCAGTTGAAACTATGGGTGTGTGTTTATGATGAATTTGATCCGAAAGTTATTATCCAAAAGAAACCCAAATCATTCCTTCAAATAGATTCATTACAACGTGGAAAAAAAAATCAATAA

>Csa009413

ATGGCGGTTACAGATTTCTTTGTTGGAGAGATAGCCACTGAGCTTCTCAGAATGATGGTACAACTTTCGACCAAATCCTGCCTTTGTAAAACGACGGCAGCTCAAATCGCCAATTCTATTCAACAAATTCTGCCGATTATCGAAGAGATCAAGTACTCGGGAGTTGAATTACCCGCTCATCGCCAATTTCAGTTAGATCGCTTCAGCGAAACTCTTAGAAGAGGCATCGAGATTTCCGAGAAGGCTCTTCAATGTGGCCGATTAAACATTTACAGAAACTTACGGCTCGCGAGGAAGATGGAGAAGCTTGAAAAGGATATATGTCGATTCATTAATGGCACCATGCAGGCGCATATACTGGCCGACGTGCATCATATGAGATTCCAGACCACCGAGCGGTTTGACCGGCTTGAAGGTGTTTTGTTGGAGCGGCGGCTTGAGTCGATGAAGATTAGAGCAGATGCTTCGGGAGAGGAAAGGTGGTGGGTTGAGGAGGCGTTTAAGAAGGCCGAGGAGGAGGAAAGGTATGAGAGTAATTTCGTGAATATAGGAACTGGATTGCGTGTGGGGAAGAGAAAATTGAAGGAGCTGGTGATTGGAAAGGAGGATTTAACGGCGGTTGGGATTAGTGGAATTGGGGGTTCGGGGAAGACTACTTTAGCTAGAGAATTCTGCAAAGATCCGGAAGTTCGAAGACACTTTAAAGAGAGAATTTTGTTCTTAACGGTGTCACAGTCCCCTGATGTGGAGCAGCTGAGGAGAACGATCTGGGAATTTGTGATGGGTAGTGATAGTGTCAATTCTAATAATTTGATTTTACATGGGAGGCCTTCAAATTCAGCGCTTTTGGTTCTGGATGATGTGTGGTCAATTTCAGTTCTTGAAAATGTTATTCCAAACGTAACTGGTTGCAAAACTCTTGTTGTTTCACGATTCAAATTCCCTGAAGTTCTTAGAGAAACTTATGAAGTAGAGTTGTTGAAAGAAAGTGAAGCAATTGCTCTGTTTTGCCACTCAGCTTTCGGACAACAGTCGATTCCTTTGTCTGCTAATCACAACTTGGTCAAACAGGTTGTGAATGAATGCAAATGTTTGCCTCTGGCTCTTAAAGTCATAGGAGCATCACTCAGAGGACAGAGCGAGATGTTCTGGAATAATGCCAAGTCTAGGTTGTCACGTGGCGAGCCTATTTGCGAGTCCCATGAGAACAAATTGCTTCAAAGAATGGCAATCAGTATTGAACGCCTCTCGAGTAAAGTGAGAGAATGTTTCCTCGACCTGGGATGCTTTCCTGAAGACAAAAGAATTCCTCTTGACATTCTCATCAATGTTTGGAAGGAGTTACATGATCTTGATGACGAAGAAGCTCTTGCTGTTCTTTTCGAGTTATCTCAGAAGAATCTTCTTACGTTGGTGAAAGATGCACGCGGTGGTGACATTTATAGCAGTTATTATGAGATGTATGTCACTCAACACGATGTATTAAGGGACCTTGCCCTTCATTTCAGTTGCCAGGAGAATGTGAACGACCGCAAGCGATTACTGATGCCAAAAAGCGACACAGAGCTTCCAAAAGAATGGTTAAGGAAATCGGAACAGCCATTTAATGCCCAACTTGTTTCAATTCACACAGGTGAAATGGAAGAAATGGATTGGGCGCCTATGATATTTCCTGAAGCTAAAGTGCTCATTTTAAACTTCTCCTCGAGTGGATACTTCTTGCCTTCTTTTCTTTGCAACATGCCGAAGATAAGAGCATTAATTGTGCTAAATAACAATGCAACACATGCAACTCTCACCAATTTCTCAGTTTTTTCTAGTTTGGTCAACTTGAGAGGCATCTGGCTGGAAAAAATTTCCATGACACAACTATTCGATGCTTGCACGCCATTGAAACATCTAAGGAAGCTATCTCTTGTTTTCTGCAAGATCAACAACAGCCTCGACGAGTGGGCGGTAGATGTATCCCAGATCTTCCCGTTTCTTTTCGAACTCAAAATTGATCACTGCAACGACTTGCGTAAGCTACCTTCAAGCATTTGTGAGATGCAAAGTCTCAAGTGTCTTAGTGTCACCAACTGTCATAATCTCAGTCAACTCCCTACCAACTTATGGAAGCTGAAAAATCTACAAATCTTGAGACTTTTTGCTTGCCCACTCCTCAAAACTCTATCCCCAAGCATTTGTGTACTTTCTTGTCTAAAGTACATTGACATCTCCCAATGTGTTTACTTAACCAGCCTTCCTGAAGAAATTGGCAAGCTGACAAGCCTAGAGAAAATTGACATGAGAGAATGCTCACTCATAAGGAGACTACCTAGATCAGTTGTGTCTTTGCAATCTCTCTGTCACGTAATCTGCGAAGAAGACGTCTCGTGGCTATGGGAGGATTTGAAGAGTCATATGCCTAATTTGTACATTCAAGTCGCCGAGAAATGCTTCAACTTAGATTGGCTCAAAGAGTGA

>Csa009587

ATGGAAACTGTAATCGCAATTTTAGGGACAGTTTGTGAGTACGCAGTTGCACCCATTGGACGTCAAGTAGGATATGTTTCTTCCTACAAAAAGAACATCAATGATCTTAAAGACCAACTTCAAAATCTTGTGGATACTAAAACAAGGCTACAACACATGGTCAATGAGGCAAGAAGTAGTGCGTACAATATCCAAAGTGATGTTTCATCATGGTTGAACCAAGTAGATAAAATCATTGAACAATCCAACGACATATTGTACAAGAATGAAAATGAATCAAATAGCAAGTATTGTTCCAATAAGCTTAACTTCATTCATCAATATCAAATGAGTAAGAAAGCTAAGAAGATGGTGAAAGTAATTTCACAAATTATAGAGAAAAGAAAGTTGATGTTTCACCAAGTTGGTTATCCTACACCTCTTTCAAGGATTCATGGAAGTTCTACTAGTAGTTCTCATGGCTATGATCAGATTTTGGAATCAAGAACATCAATCGCCAAGCAAATTAGAGATGCACTCGTTGATTGTAACGTGAATAAGGTTGGAGTATATGGTATGGGAGGTGTTGAAAAAACTACGCTGCTGAAACAAGTCACACCATTAGTGATGGAAGAAAAATTGTTTGATCACGTGATTATAGTGAATGTAGGTCAAACATTGGGGGTAGAAGGCATACAAGCTCAAATTGGAGATAAGTTAAGGTTGGAATTAAATAAGAAGGTAGAGAGTAAGGAGGGAAGAGCATCTTTACTACAAAATAAGTTGGAGATGGAAAGTAACGTCCTCTTGGTGTTAGATGATTTATGGAAGGGACTCGATCTAGAAGAGGTTGGAATTCCTTGTAGATCAGAGTCATGTGAAAAGGGATGTAAGATACTCATAACAAGTCGAGATAGAGATGTCTTAACTAATGAAATGGACACACAAGTTTATTTCGAGGTGAAGCCTTTAAGTGAAAAGGAATCATGGGAGTTTTTCAAGAACATGATTGGTGAGTTTGATAATAAATGCATAGAACTAATAGGGAAAGAGATGGTGAAGAAGTGTGGGGGATTGCCAATAGCACTTGCTACAATTGTAAAAACTTTGAAGGGGAAGGAAGTGCCTATTTGGAAGGATGCTTTGAAGCAATTGAAAAATCCTATTGCAGTGGATGTTAAAGGGGTGACTGAGTTATGA

>Csa009602

ATGCTTTTGGGAATAGGTTTAGATGATGTACGCTTTGTTGGAATATGGGGAATGGGTGGAATTGGCAAAACTACAATTGCTAGAATCATTTACAAAAGTGTTTCTCATTTATTTGATGGATGTTATTTCTTGGACAATGTCAAAGAAGCTTTGAAGAAAGAAGACATAGCTTCATTACAACAAAAGCTTCTAACAGGAACTCTAATGAAAAGAAACATTGACATCCCTAATGCTGATGGAGCTACATTAATTAAGAGAAGAATAAGTAATATTAAAGCTCTTATAATTCTTGACGATGTCAACCATCTAAGCCAACTTCAAAAATTAGCCGGCGGTTTAGATTGGTTTGGCTCAGGAAGTCGAGTCATCGTTACAACGAGAGACGAACATCTCCTAATTTCACATGGAATCGAAAGACGATACAATGTTGAAGTGCTGAAAATTGAAGAAGGTCTTCAGCTTTTTTCACAAAAGGCATTTGGAGAAGAGCATACAAAGGAAGAGTATTTTGATGTTTGTAGCCAAGTTGTAGACTATGCTGGAGGACTTCCATTGGCAATTGAGGTTCTTGGATCTTCTTTACGTAATAAACCAATGGAGGATTGGATAAATGCAGTGGAAAAGTTGTGGGAAGTTCGTGATAAGGAAATTATAGAAAAGTTGAAAATTAGTTATTATATGTTGGAGAAATCTGAACAGAAAATTTTTCTAGATATTGCATGTTTTTTTAAGAGAAAGAGTAAGAAACAAGCAATAGAAATTCTTGAAAGTTTTGGATTTCCTGCTGTTCTTGGACTAGAAATATTGGAGGAGAAATGTCTTATTACTACACCACATGATAAGCTACATATGCATGATTTAATACAAGAAATGGGCCAAGAAATTGTTCGCCAAAACTTTCTGAATGAGCCCGAAAAGCGAACTAGGTTGTGGCTTCGTGAGGATGTCAATCTCGCACTAAGTCGAGATCAGGAAGGAGAATCACATTTGAATGCCAAAGCCTTTTCAGAAATGACAAATCTAAGAGTATTGAAATTGAACAATGTTCATCTTAGTAAAGAAATTGAATATCTGTCTGATCAACTAAGGTTTCTCAATTGGCATGGTTACCCTTTAAAGACCTTACCATCAAATTTCAATCCCACAAATCTATTGGAGCTTGAGTTGCCAAATAGCTCTATTCACCATCTTTGGACTGCTTCAAAGAGCATGGAAACATTGAAAGTGATAAACCTAAGTGATTCTCAGTTCCTATCAAAGACACCTGATTTTTCAGGTGTTCCAAATCTTGAAAGATTGGTTTTAAGTGGCTGTGTAGAACTTCACCAACTTCACCACTCTTTGGGTAATCTAAACCATCTAATTCAATTGGACCTCAGAAATTGCAAGAAATTAACAAACATTCCTTTCAATATTTCCTTAGAATCTCTCAAAATTTTGGTTCTTTCAGGCTGTTCAAATCTCACCCATTTCCCAAAAATCTCATCAAACATGAACCATCTACTAGAGCTTCATTTAGACGAAACATCCATAAAAGTTTTGCATTCATCAATAGGACATTTAACATCACTTGTTTTATTAAATCTCAAAAATTGCACAGATCTTCTAAAACTTCCTTCCACTATTGGCTCTCTAACATCTCTAAAAACCCTCAATTTAAATGGCTGCTCAAAACTTGATAGTCTTCCAGAGAGTTTAGGAGATATTTCTTCCTTAGAGAAGCTTGATATTACAAGCACTTGTGTAAATCAAGCTCCAATGTCATTTCAGCTTTTGACCAAACTAGAAATACTAAACTGTCAAGGACTATCTCGCAAATTTCTTCATTCATTATTCCCTACTTGGAAGTTCACTAGAAAATTCTCCAATTATTCTCAAGGGTTGAAAGTGACAAATTGGTTTACATTTGGTTGCTCTTTGAGGATTTTGAATCTGAGTGATTGTAATTTGTGGGATGGAGATTTACCTAATGACCTTCATAGCTTAGCTTCATTGCAAATTCTTCATCTAAGTAAAAACCATTTTACCAAATTGCCTGAAAGCATCTGTCATCTTGTGAATTTGAGGGATCTATTTTTGGTGGAATGTTTTCATCTTCTGAGTTTACCAAAACTTCCGCTAAGTGTTAGAGAAGTAGAAGCAAGAGATTGTGTTTCACTAAAAGAATATTACAATAAAGAGAAACAAATTCCTTCAAGTGAATTGGGAATAACATTTATCCGATGTCCTATTTCCAATGAACCATCTGAAAGTTATAATATTGATCAGCCACACTTTTCTGCCATTCACGTAAGGACAACGACTCAACGATACATTGAGGTAATTAACTTCTTTACTTTTCTTTTCTCATCATAA

>Csa009604

ATGCTACTAATTAACTATGCATCCAACGCTTGCACATACTTGTTATCAATTGGAAGTCTTATTACAACTAAAAAGAGAGATGTACTTCATCAACTAAATTATAGAGATAAAGTGCTAGAATACAAGGTGGAGTTACTTTCTCGTGAAAGTGCTTACTCACTGTTTAGCAAGAATGCATTTGGAGGTGGCCCTTCCGATAAAGATGAACTTTGTAATGAAATTGTGGAAAAGGTTGGAAGACTTCCATTAGCTTTGAAAACCATTGGCTCCTATTTGCATAATAAGGAGTTGGATGTGTGGAATGAAACATTGAAGAGACTAGATGGAGTGGAGCAAGACTTCTGTGATACAGTATTGCAGAAAAGTCAGAAGAATTTACACTAA

>Csa009605

ATGGAGGGTTGTGTCAGTTTCAGAAGCTTCTCATTTGCTATCACTTGCGAAAGTCTTGAAACTTTAGTTCTTTCTAACTGCGGTCTAGAGTTTTTTCAAGAGTTTGGATGTCTGATGGGATATTTGACTGAACTACACATTGATGGAACTTTCATAAATGAACTCTCTATCTCAATTACAAATCTATTTAGCTTGATTTTATTGAACCTGAGGAATTGTATTAGACTTCCTTGTCTTCCAACTGAAATTGGTAGCTTGAGTTCACTTAAAACTCTCATTCTTAATGGTTGCAAAAACTTGGACAAAATTCCATCAAGTTTGGGGAATGTAAAGCCTCTCGAGGAGCTTGACATTGGGGGAACATCCATAAGCATTATTCCTTTCTTGGAAAATCTAAGAATTTTGAACTGTGAAAGGCTGAAAAGCAATATTTGGCATTCTTTAGCTAGTTTGCCAGCAAATTATTTTAGTTCACGCAGAGATTTAAATTTAAGTGATTGTAATCTGGTGGACGAAGACATTCCTAATGATCTTAAACTCTTTTCCTCATTGGAAATTCTAGATCTTAGCAGCAATCATTTTGAAAAACTGTCAGAAAGCATTGAACAACTTATTAACCTTAAAGCATTTTACTTGAATGATTGCCCCGAGCTAAAGCGAGTACCGAAGCTTCCAAAAAGTACAAAATATGTGGAAGGAGAGAAGTCCTTGGGCACCAGGTCAGAGATGTCACCTTCACAATCTGATGCTCGTCTGTTGAAAAGTACAAAATATGTGGAAGGAGAAAAGTCCAAGGGGAGCACTAAAGAAGTGCATATTACGAAGGATATGGGGAAGCAGACTAATCATAAGCTAGTACTTGCTCACAAAACTAGTTTAGTTGGAATGGAGAATCAAGTGGAGAAAGCTTGTAATCTCCTAGATTTAGAACGATCCAAGAACATACTTTTTGTGGGGATTTTTGGGTCAAGTGGCATTGGTAAAACAACCATTGCTGAAGTTGTTTACAACACAATTGTAGATGAATTCCAAAGTGGTTATTTTCTCTACCTTTCTTCAAAGCAAAACAGTTCAGTCCCACTTCAGCATCAAATGCTTTCTCATCTTCAATCAAAAGAAACTAAAATCTGGGATGAAGATCATGGAGCACAACTGATTAAGCATCACATGAGTAATAGAAAAGTTGTTATATATTGTTCTTGA

>Csa009607

ATGTTAGCTGGCAGCCCCGATTGGTTTGGCGCAGGGAGTCGAATCATTATTACAACCACAAATAAAAATATTTTTCATCACCCTAATTTCAAAGACAAAGTGCAAGAATATAATGTAGAATTACTTTCTCATGAGGCTGCCTTCTCCCTCTTTTGCAAGCTTGCATTTGGAGATCACCCTCATACCCAGAATATGGATGATCTTTGTAATGAGATGATCGAAAAGGTTGGAAGACTCCCATTAGCTTTGGAAAAAATAGCTTTTTCATTGTATGGTCAGAACATAGATGTATGGGAACATACATTGAAGAATTTTCATCAAGTAGTTTATGATAATATTTTCTCTGATGTATTAAAGTCAAGTTATGAAGGATTAGAAGCAGAGAGCCAACAAATTTTCCTAGATTTGGCATGTTTCCTCAATGGAGAGAAGGTGGATAGAGTGATTCAAATACTTCAAGGCTTTGGTTATACCTCACCTCAAACTAATTTGCAATTGTTGGTTGATAGATGTCTTATTGATATTTTAGACGGACATATACAAATGCACATCTTGATTCTTTGTATGGGCCAAGAAATTGTGCACCGCGAGCTGGGAAATTGTCAACAAACAAGGATTTGGCTTCGAGATGATGCTCGTCGTCTATTTCATGAAAACAATGAATTAAAATATATTCGTGGAATAGTGATGGACTTAGAGGAGGAAGAAGAATTGGTATTGAAGGCTAAGGCATTTGCAGATATGTCTGAGCTAAGAATTTTACGAATCAACAATGTGCAACTTTCGGAAGATATTGAATGTCTGTCAAATAAATTGACGTTGCTCAACTGGCCTGGCTATCCTTCAAAGTATTTGCCATCAACTTTTCAACCACCATCTCTGCTTGAGTTACACTTGCCTGGTAGTAATGTTGAACGACTCTGGAATGGAACACAGAATTTTAAGAACTTAAAGGAGATTGATGCAAGTGATTCGAAGTTTTTGGTTGAAACTCCTAATTTTTCAGAGGCTCCAAAGCTTCGACGATTGATTTTACGAAATTGTGGAAGACTAAACAAGGTTCATTCTTCAATAAATAGTCTCCATCGTCTAATTTTATTGGACATGGAAGGTTGTGTCAGTTTCAGAAGCTTCTCATTTCCTGTCACTTGCAAAAGTCTCAAAACTTTAGTTCTTTCGAACTGTGGTCTAGAGTTTTTTCCAGAGTTTGGATGTGTGATGGGATATTTGACTGAACTACACATTGATGGGACTTCCATAAATAAACTTTCTCCCTCAATTACAAATCTACTTGGCTTGGTTTTATTGAACCTGAGGAATTGTATTAGACTTTCTAGTCTTCCAACTGAAATTTGTAGGTTGAGTTCACTTAAAACTCTCATTCTGAATGGTTGCAAAAACTTGGACAAAATTCCACCATGTTTGAGGTATGTAAAGCATCTTGAGGAGCTTGACATTGGCGGAACATCCATAAGCACAATTCCTTTCTTGGAAAATCTAAGAATTTTGAACTGCGAAAGGCTGAAAAGCAATATTTGGCATTCTTTGGCTGGTTTGGCAGCACAGTATTTAAGGTCACTCAACGATTTAAATTTAAGTGATTGTAATCTTGTGGATGAAGACATTCCAAATGATCTTGAACTCTTTTCCTCATTGGAAATTCTAGATCTGAGCAGCAATCATTTTGAAAGACTGTCAGAAAGTATTAAACAACTTATTAACCTTAAAGTATTGTACTTGAATGATTGCAACAAGCTAAAGCAAGTACCCAAGCTTCCAAAAAGTATAAAGTATGTGGGAGGAGAGAAGTCCTTGGGCATGTTAAGAACTTCACAAGGCAAAGTTCCCCTGTATGCACCAGGTCAGAGATGTCACCTTCACCATCTCGTGATCATTCGTTCACATGTACTGAATATGCAGTACCCAAGCTTCCAAGAAGTATAA

>Csa009608

ATGGTTTTGGCTGGTTCATCATCTCATGCATCTCATTTGCCTTTTAATGTTTTCATAGGTTTCAACAGACATGAGGAAGACAACAAATACAAAAGTTCTACGAGGGATTTATATAAGATTTTGTGTATCCAGGGAATGAAGGTGTTTATGGATGATGATGGTGGTGGTGGTGGTGGTAGTAAGAAGAAGGTGTTAATGGATGAAGATAATGTGAGCGATAAGATTGTGAAAGCAATTGAAACTTACAGTGATAGTAATTCTTCGACCTCATCAATGGCTTCCTCAACCCCCAAGGAATTATCTTCTTTTTCTTCTTCTCCTAGATTCATATTTGACGTCTTTCTCAGCTTCAGAGGCGTCGACACTCGCAAGAATGTCACAAATCGTCTTTACGAAGCTCTGAGGCGACAAGGCATCATTGTTTTCAGAGATGACGATGAGCTCGAGAGAGGGAAGACTATTGCTAACACTCTAACCAACTCGATTAACCAATCCAGGTGTACCATTGTTATTCTCTCTAAAAGATATGCAGATTCAAAATGGTGCTTGAGGGAGTTGGTTGAGATTGTCAAATGCAAGAATACCTTCAAGCAATTAGTTCTTGTGGTTTTCTACAAAATTAAGCCCTCCGATGTCAACAGCCCTACTGGGATTTTTGAGAAATTTTTTGTTGATTTCGAAAATGATGTTAAGGAGAATTTTGAAGAGGTTCAGGACTGGAGGAAGGCCATGGAAGTGGTTGGAGGTCTCCCTCCATGGCCTGTAAATGAACAGACCGAAACAGAGAAAGTCCAAAAGATTGTTAAGCATGCTTGCGATCTTCTGCGTCCTGATTTGCTTAGCCATGATGAGAATTTGGTTGGCATGAACTTGAGATTAAAAAAAATGAATATGCTTATGGGCATAGGACTGGATGATAAGCGCTTTATTGGGATATGGGGGATGGGTGGAATAGGCAAGACAACTATTGCTAAAGCTGTTTTCAAAAGTGTCGCTCGTGAATTCCATGGAAGTTGCATTCTGGAAAATGTTAAGAAAACTTTAAAGAATGTTGGAGGCTTGGTGTCCTTGCAGGAGAAACTTCTTTCCGATACTCTAATGAGAGGAAAAGTTCAAATTAAAGATGGCGATGGAGTTGAAATGATAAAGAAAAACTTAGGAAATCAAAAAGTTTTTGTTGTTCTTGATGGTGTTGATCATTTTAGCCAGGTGAAAGATCTGGCAGGAGGAGAAGAATGGTTTGGTTGTGGAAGTAGAATCATCATTACAACAAGAGATGAAGGTTTGCTTCTTTCTCTTGGAGTTGATATAAGATACAATGTTGAGAGTTTCGATGATGAAGAGGCTCTTCAGCTCTTTTGCCATGAAGCATTTGGAGTAAAGTTCCCTAAGAAAGGTTATTTGGATCTTTGTATGCCATTTATAGAATATGCTGAGGGCCTTCCATTAGCAATCAAGGCTCTTGGGCATTCTTTGCACAATAGATTGTTTAAGTCATGGGAAGGTGCTATTAGAAAGTTAAATAATTCTTTAAACAGGCAAGTATATGAAAACTTGAAAATTAGTTACGATGCACTTGGAAAGGAAGAGAGGAGAATTTTTTTGTATATTGCCTGTTTTCTTAAAGGACAGAACAAAGACCAAGTCATTGACACATTCGTGAGTTTTGAAATTGATGCTGCTGATGGGCTTCTTACCAGAAAAAATGCTGCCGATGTACTTTGTATAAAAGAAACTGCTGCTGATGCTCTAAAAAAATTGCAGGAGAAATCCCTTATAACTATGTTATATGACAAAATAGAGATGCATAATTTACACCAAAAACTAGGTCAAGAAATTTTTCATGAGGAGTCATCGAGGAAAGGTAGTAGGCTATGGCATCGAGAGGATATGAACCACGCTTTAAGGCATAAACAGGGAGTTGAAGCTATTGAAACCATTGTCTTGGACTCAAAAGAGCATGGAGAGTCACACTTAAATGCCAAGTTCTTTTCAGCAATGACCGGTCTAAAAGTGTTGCGTGTTCATAATGTATTCCTTTCTGGAGTTCTTGAATATCTCTCAAACAAGTTGAGACTTCTCAGTTGGCATGGATATCCCTTCAGAAATTTACCATCGGATTTCAAGCCGAGTGAACTATTGGAACTCAATTTACAGAATAGCTGCATTGAAAATATTTGGAGAGAAACAGAGAAGTTGGATAAATTGAAGGTAATTAACCTTAGTAATTCCAAGTTCTTATTGAAGACCCCTGACCTGTCAACGGTGCCAAATCTTGAGAGGTTGGTCTTGAATGGTTGTACAAGACTACAAGAGCTTCACCAATCTGTCGGCACTCTAAAGCATCTAATCTTTTTGGATCTTAAGGACTGCAAATCTCTCAAAAGCATTTGTTCTAATATTTCTCTTGAATCACTCAAGATTCTCATTCTTTCTGGTTGTTCAAGACTTGAAAATTTTCCAGAGATTGTGGGAAACATGAAACTTGTGAAAGAGCTTCATTTAGATGGCACTGCTATTCGAAAATTGCATGTCTCGATTGGAAAACTTACAAGCCTTGTTTTGTTGGATCTTAGATACTGCAAAAATCTTCGTACACTTCCAAATGCAATCGGTTGCTTAACATCCATTGAACATCTCGCATTGGGTGGCTGCTCAAAGCTTGATAAAATTCCTGACAGCTTGGGGAACATTTCTTGTTTAAAGAAACTTGATGTGAGTGGTACTTCTATTAGTCATATCCCATTTACTCTAAGACTTTTGAAGAACCTTGAAGTATTGAATTGCGAAGGCCTATCCCGAAAATTATGTTATTCGTTGTTCCTATTATGGAGTACGCCGAGGAATAACAATTCACATTCATTTGGTTTGTGGTTGATAACTTGCTTAACGAATTTTAGTTCGGTAAAGGTTTTGAATTTTAGTGATTGCAAGCTGGTAGATGGAGACATACCCGACGACCTCAGCTGTTTGTCTTCATTGCACTTTCTGGATCTAAGCAGGAACCTCTTCACCAATCTGCCTCATAGTTTGAGTCAACTTATCAATCTCAGATGCCTTGTTTTGGACAACTGCAGTAGACTCAGGTCATTACCGAAGTTCCCAGTCAGTTTACTTTATGTACTCGCAAGGGATTGTGTGTCACTGAAAGAACACTATAACTATAACAAAGAAGATCGCGGGCCTATGAGCCAAGCAGAAGTAAGGGTCCTTAGTTACCCCTCATCAGCTAAAGACCAAAACTCTAAAATCTCTCAGTTAATGATATCAAGTATGTGCACAGCTTGCGAGAATGGGGGTTGA

>Csa009775

ATGCAGAGTTCATCATCGTCTTCTTTGGATCGTCCTAAGATGAACTATGATGTGTTCATAAGCTTTAGAGGTAGAGATGTTCGTCACACTTTTGCAGGATATTTGTACGATGCTTTGAATCGTTTGGGGATAAAAGCTTTCCTGGACAACAAGAGGTTTCTAATTGGAGATGATCTTCATGACTTATTCAAAATAATCGATGAATCAAGATCAGCAATTGTTGTTCTTTCAGAAGACTATGCTTCTGCTAAATGGTGTTTGAGAGAGTTGACTAAGATAATGGATTCCATGGGAACCTCAATGGAGCGTGTCCTTCCTGTGTTTTATCATATTGATCCATCAATTGTTAAAGATCAATCTGGAACTTTTAAGACAAGTTTTGATGAACATGAAGCCAATGTTTTAAAGGAAATTGATAATCAAGAGAAGGAGAAGCGCTTGAAGGAACTCCAGAATTGGAAAAGTGCACTGAAGAAAATTGGCAATCACACTGGAGTTGTCATCACTAAGAACAGTTCTGAGGTAGATATAGTAAATAAAATTGCAAGTCAAATATTCGATGCATGGCGTCCTAAGTTGGAAGCATTGAATAAGAATTTAGTTGGAATGACATCCCGATTGCTCCATATGAACATGCATCTTGGTTTAGGATTAGACGATGTACGCTTCGTTGCGATAGTAGGAATGGGTGGTATTGGTAAAACAACTATTGCTCAAGTCGTTTTTGATTGCATTCTTTCAAAGTTTGAAGATTGCTGCTTTCTAACATTACCTGGAGGTGATTCAAAGCAAAGTTTAGTGTCATTACAACGGGAAATGCTTTCTCAAATTTTTCATAAAGAAGATTTTAGAATATGGCATGAGAATCATGGAGTAGAGATGATTAAAAATCGACTGAGTGGTAGAAAGGTTCTTATTGTTCTTGATGGCATCGAAGAGAGAAGGCAGTTAGAAATGTTGGCTGGAAGCATCGAGTGGTTTGGTCCTGGAAGCAGAATCATCATTACAACTAGAAATAAAGGATTATTGTGCCATCCTAATTATGATGAAATGAAAGTATACAATGTTGAAGAACTAGATCATGATAGTGCCCTTCAACTCTTTTTGAAGCATGCATTTGGTAGTAATCATCAAAACAATGACAGTTTCATGGATCTTAGTAATGAGATAGTTGAGAAGGCTAAAAGACTTCCATTAGCTTTAAGAGTGATTGGATCTTCTTTGTATGGTAAAGATATTACAGTATGGAGAGAAACGTTGAAGAGGCTGATCAAAGTGGATGAAAGAAATTTTTTTGATGTATTGAAAATAAGTTATGATGGATTAGGAGTTGAAAGCCAACAAGTTTTTCTTGACATTACATGTTTCTTCAATGGAAAAAATGAAGATAGAGTAATTGAAATATTAGAGAGTTTTGGTTATAGTCCTAATAGTGAAGTACAATTACTGATGCAAAGATGTTTAATTGAAGTTTCACACAAGAAAATATTGGTGCATGATTTAATTCTTGAAATGGGTCGAGAAATTGTGCGTAAGGAGTCCCTCACTCAAGCAGAAAAACAGAGTAGGATTTGGCTTCATGAAGATCTTTACTGCAGGTTTGCTGAAAAACATGACTTGATGCATATTCAAGGGATAGTTTTAAGTTTGGCAAAAGAAATGGAAGAATCAATAGAATTGGATGCTGAATCCTTTTCAGAGATGACCAAACTAAGAATACTGGAAATCAGTAATGTGGAGCTCGATGAAGACATTGAATATCTCTCTCCACTCTTACGGATAATTAATTGGCTTGGCTATCCTTCGAAGAGTTTGCCCCCAACGTTTCAATCCCGCTATTTGTTTGAACTACTCTTGCCTCATAGTCACCTTTTACGAATTTGGGATGGAAAAAAGAGATTTCCAAAGCTGAAATTAATTGATGTTAGTAACTCAGAACACTTGAGGGTGACACCTGATTTTTCTGGGGTTCCAAATCTTGAGAGATTGGTTCTATGTAACTGTGTTAGACTGTGTGAGATTCATCCCTCCATCAATTCCCTCAACAAACTCATTTTACTGGATTTAGAGGGTTGTGGTGATCTTAAACATTTTCCAGCAAATATAAGATGTAAAAATCTCCAAACACTCAAACTTTCTGGTACAGGTCTTGAAATTTTTCCAGAGATAGGCCATATGGAACATTTGACTCATCTTCATCTTGATGGATCCAATATAACCCATTTTCATCCTTCAATTGGGTATCTAACTGGCTTAGTTTTCTTGGACCTATCCTCCTGTTTAGGTCTTTCTAGTCTTCCTTGTGAAATTGGTAACTTGAAGTCTTTGAAAACCCTCCTTTTGAAATATTGTAAAAAACTTGATAAAATCCCTCCAAGCTTAGCAAATGCAGAATCCTTGGAGACACTTTCTATTAGTGAAACCTCAATAACCCATGTTCCACCAAGCATTATTCATTGTTTAAAGAACCTAAAAACGTTAGATTGTGAAGGACTATCACATGGAATTTGGAAGTCATTGCTCCCCCAATTCAACATTAATCAAACAATAACCACTGGTTTGGGGTGCCTCAAAGCTCTAAATTTAATGGGTTGCAAACTTATGGATGAGGACATTCCTGAAGATCTCCATTGCTTTTCTTCATTAGAAACACTAGATCTCAGCTATAATAACTTCACAACACTCCCTGATAGTCTTAGCCACCTCAAGAAGTTAAAGACATTGAACCTGAATTGTTGCACTGAGCTTAAAGACTTACCAAAGCTTCCAGAAAGTTTGCAATATGTAGGAGGAATAGACTGCAGATCGATGTCAGAACGATATTATAACAAAATTTTGCTTATCCCTTCTAGTTCTGGGCACCAACTTTACCTTACTTTTATCATTCCTTCCAAGGATGTGGATGTAGAATGTGACATGAATGAGTTCCAACATTCGATATTTACTCGAAGATCATTTGAGCTAAACATTATAGAAGAGAAACCATCCATGATCGTCCATGACGCTGTTGACATGTTCCACTGGTTTGGCCAGATAAATGAAGGGAATTGGACAAACATTCAGTATGAGCAAGAATTCTCGATCAGTAAACCACTCAATATTATGTATGAAGATGTTGATCTAAGCAATGTTTGTGGAGTTTTCCTTTCAACAAACATTGAATTTCCAGAAAATTTAAATCATCTTGCAATTGGAAGATTCTTAGTTAGCTTTGAAATTGATGGGAAATGTTCTGGTGGAACAATGAATTATGAGATGTCCCAATTTAAAGCTGCAAGATTCTTTTGGGCAGCATACATACCAATTTGGATGTTTAAAGATCATAGTGTGATGGTTCAAAGATGTTGCTCTATGAAGGTTACAATTAGTTATTGTTGTGATCATATAGATGCAAGCAAGGTCAAAATTAAAGCATGTGGTGTTTCTTCTATGCTCTCCTGGCCAAATGTAGCAGAGTATTTGGCAAAATTGTTCACTAAGCGTTTTTGCTCTAAACGCAATTTCTACACCATGATTCGACAACATAATGATCATCAGAATGAATGCAGGTGTGATGAACTTGAAGTTCGAAAGGATGACTTTTCATCCTCTACATTTGAATCTAACGATTCAACATTTTTGCTCAGGAAGAACCTCAGAGCAATACTTGGGGTAATGTTCGAGGAAAAAAAGCGTTACTACATGAAATACTTCTTCCCACATACAAATATTTTTGGATGGTTCAAAAATCAGAATAAGAAAGACAAAGTAGCAGTGAAGATTCCCGTAAATATAGAAAAAGACAGGAAGTGGATGGGGTTGGCAATGTTTGTTGTCTTCTCGATTTCTGAGAAAGCTTCTTGTTATTGTTTTGAGTATGAAATACAAACAAAGGAGAAAATTATATCCACCCAAAGGCACTCTATCTCTACAGATCAGGTTTTAGAGTATTCAAATCAAATACTGTTTGTGGCTTTTGAACCGCGGTATAATTGGTATCCTTATGATGAACTGAAGTCTTCTTCATCCAACCATGTTTTCATTAATTTCAATACTAATGGTGCAAGAATGCGAGTAGAGTTTTGTGGAGCTCGTTTAGTTTACCAGCAAAATGTTGAGGGACTTATTCACACAATTATGAATTGCATAGAGGAGAGTGGTGATGAGCTTTATGAATATTATAACCAACAAATTGTGGAATCTCATTTAAACTTGATAAATGCACATTGGTATACTATCTCATTCCGTCGAAATAACTCTGTGAAGAATCAACCATCAACTGCTGCTTCAACTTGTACTGCTTCAAGCCTTTCAGTTGAGCACCTTTTATATGGAAGTTTCCCACACCCATTCTTCCATAAGTCCTTACAGGAACGATTTAGAAGTAAGTTTGATCTGCTTCTACATGGAGATAAGATCCCAAAATTTTTCAGCAATCAAAGCAAAGGAAACATGACAGAAATAAAGTTACCACAATATTTGGAAAAATTTCGAGAGAGTATAGGTGTTGCTGTGTGTGCTCTTGTGGTTGTGGACAAGAAAAGAAGAAAACTAAATGAGATTATTCCAGAACGAGAGAGGTATACAAAAGTTGTGGATCTTATATGTAAATTCAAAGTTGACTCGTATCAAATTATGCCAGAGCACTGTCACTTCACATCCCAACAAAAACTGTTGAGTGAATATGCTTCACAATTCCTTTGGCTCTCTTACATTCCCCTTCATGGATTTAATATCAATTGGCATTATTGCACCCAGTTTGAAATTGCACTTGAAACTAGCTGTGATGAGCTATTTGGAGTGAAGAATTGTGGTCTTCATCTCATACATAAGCATGAAAGGATGATGATTGATAAGATGGTAATGGAGTCAACTGTTCCATCATCCACTAGCCACAAAGGAAAGGAACCTCAAATTCATTGA

>Csa009776

ATGAGTACTTTTGATACTTTCATAAGTTTTAGAGGCGAAGATACTCGTAACACGTTCACCGGACATTTGTACAAGGCGCTGGTTGATTTTGGAATATCGACTTTTATGGACGACAAGAAACTCTTGATTGGAGATAGTCTTAGTGAAGATCTTATTGGAGCTATAGAAAAATCAGGGTCTTTCATTGTTGTTTTATCAGAGAACTATGCTTCTTCAAAGTGGTGCTTGAGAGAATTGGTGAAGATAATTGGTTGTATGGTGGAACAAAAGCGTCGAGTTCTTCCTGTATTTTACCACGTGAGTCCTCATGATGTTCGACATCAATCAGGGTGTTTCAAGAAAAGCTTTTGTGAATATGAAGAAATTCTTCAAGAGCTCAACGATAGGGAAGGAGATAAATATACGAAGGAGGTTCAAGAATGGAGGAGTGCGTTGACAAAAGTTGGCGAGCTCACTGGAGTAGTTGTAACAAAAGATAGTCTTGAAGCCGCTAGCATCGACAAAATCACAGAACAACTAAGTTCTACGTTGCATCAACAAAAGTTAGTAAATTTGGACGAGCTCACTGAGTTAGTTGATATTGAACGTCAGTTATGCAAGATGGATAAGCTAAATGATTTGGAGCCAAATGTGTTAGAACAGTTAGTTGGAAATCCTAATTGGTTTGGTCAAGGGTCCAAAATCATCATTACAACCAGAAATAGGGATGTTCTTCGTCAGCCAAATTATAAAGATAAAATGGTTGAATACAAAGTGGAGTTTCTTGATAATAAAAGTGCCATGACACTCTTTTGCAAACAAGCATTTGGATCATGTGATCAGTTTCCCAGTAAGAATTTTGAGGACTTTTCTAAGGAGATTGTAGAAAGGGTTAAAGGACATCCACAGGTTTTGAGACAAATTGGGTCGTCTTTATATGATAAAGGTATAGAGATATGGAAAGAACAATTGAAGAGTCTTGAGGAAGATTACAACAATCGTATATTTAAGACATTAAAGATAAGTTTTGATGATTTAGGAAAGACAAGCCAAGAAGTTTTTCTTGATTTTGCATGCTTCTTCAATGAGAAGAAGAAAGAGAGTGTGATTGAAATACTTAAGAGTCTTGATTATAGACCTCATAGCGAAATACAATTGTTGGAAGATAGATGTCTCATTGAAGTAAGACGTGACAACACAATATTTATGCCTAAGTGCATTCAAGCTATGGGTCAACAGATTGAACGTGAAGCTGATAAACGGAGTAGGATTTGGCTTCCGAAAGATGCCCATGATGTATTTGATGAACCACATAGAGTAAAGGACATAAAAGGTGTAGTCTTGAAATTGGAAGAGAAGCAAGAAGAAGTAAAGTTAGAGGGTAAGGTTTTTGAGGATATGAGAAGTTTAAAAATATTGGAAATTGGGAATGTAGAGGTTCCAGCTCGCATCCGCCCATCTCCCCCAATGTCCCTTCTGCCAGCCGCAGTGACTCCAACGCACAACCACCGTCGTCCGCCTCTGCCTCATCTTCCTTCTCACCACCACAATGACTCCAACACACAACTTCCATCGTTCATCTATCTCTCCATCGCCACTCCGTCTGCCACACACATTTCAACCATCGCCTATCGTCGTTGTTTAGCCTGCCGCACCCAGTCCACGGTCTCTCTTTCTCTTCACTTCGTGAAGTCCTCTTTATTGGAACAAAATTCCAACGAAAGACGGTTGGGACAACATTCTAATCATCTGCGAGAGTAA

>Csa009778

ATGGGAATAGGCTTAGATGACGTACGGTTGATTGGAATATGGGGAATGGGTGGAATTGGCAAAACAACCATCGCTAGAATCATTTACAAAAGTGTTTCCCATTTGTTTGATGGATGTTATTTTTTGGACAATGTCAAAGAAACTTTAAAGAAAGAAGGCATAGCTTCTTTACAACAAAAGCTTCTAACAGGAGCTCTAATGAAAAGAAACATTGACATCCCTAATGCTGAAGGAGCTACATTAATCAAGAGAAGAATGAGTAATATTAAAGCTCTTATAATTCTCGATGATGTCGACCATCTAAGCCAACTTCAGCAGTTAGCTGGCGGTTCGGATTGGTTCGGTTCAGGAAGTCGAGTCATCGTTACGACGAGAGAAGAACATCTCCTAATTTCACATGGAATCAAAAGACGATACAATGTTGAAGTGCTGAAAATTGAAGAAGGTATTCAGCTTTTCTCACAAAAGGCATTTGGAGAGGACCATCCAAAGAAAGGGTATTTTGATCTTTGTAGCCAAGTTGTAGATTATGCTGGAGGGCTTCCATTAGCAATTGAGGTTCTTGGATCTTCTCTACGTAATAAACCAATGGAGGATTGGATAGATGCTGTGAAAAAGTTGTGGGAAGTTCGTGATAAGGAAATTATTGAAAAGTTGAAAATTAGTTATTATATGTTAGAGAAAGATGATAGGGAAATTTTTCTAGATATTGCATGTTTTTTTAAGAGAAAGAGTAAGAAACAAGCAATAGAAATTCTTGAAAGTTTTGGATTTCCTGCTGTTTTTGGACTAGACATATTGAAGGAAAAGTCTCTTATTACTACACCACACGAGAAGATACAAATGCATGATTTGATACAAGAAATGGGTCAAAAAATCGTTAACGAAAAGTTTCCAGATGAACCCGAAAAACGAAGCAGGTTGTGGCTTCGTGAGGATATAACTCGTGCTCTAAGTCATGATCAGGGAACAGAAGCAATTAAAGGGATAATGATGGATTTGGATGAGGAGGGAGAATCACATTTAAATGCCAAAGCCTTTTTTTCAATGACAAATCTAAGAATATTGAAATTGAACAATGTTCATCTTAGTGAAGAAATTGAATATCTGTCTGATCAACTAAGGTTTCTCAATTGGCATGAGAAACAAATTCCTTCAAGTGAAATGGGAATGACATTTATTCGATGTCCAATCTCTAAAGAACCATCGGAAAGCTACAATATTGATCAGCCTCGCCTTTCTGCTATTCACTTGAGAACTATGGTTCAACGATACATTGAGGTTCTGACATGGCAACAAGAAAAATACTTTTTTGTGATTCCATGTCCTAACTGCATAGGATGCTTTGATAAAAAAAAATATGGATTCTCAATAACAGCCTGTTGCGAACCAGATTACATAAGTGAAGAAAATCCAAGGATTGGAATTGCTTTAGGTGCTGCATTTGAAGTTCAAAAACATGAAATGAGAAACAACAGTAACGATGCAAAAATTTGTTGTGAGTTCATAGTGAAAATGGAAACAGATGAGTGCCCTCCAAAATCAGCCATTGTTTTTGATGGGCAAAGAGATGAATTGGGAAGCCCAGTTGGGCTATCAGTATTTTACATTCCAATGAAAAGGATATCAAGTTGGTTGAACCAATCTTGTTGCATTGATGTTTCAATAGTCACTGACAACCCATTTGTGAAGATCAAATGGTGTGGAGCTTCAATATTGTATGAACAAAATGCAGGGAGTTTTATCGGGAACATTATCAAGGACTTGTTTGGATCTCCAGGAAAATATCATACATCAATTGTTGATCATATTTTGAACCGACAAAATCGTGTCGATGTTTCTACTTTGTTGGATGGTGGAGCTCGTTACAAGACTTCTTGGTCTAATGCATTCCAAAGGACGATTGGGTCATTTCCAAGACTTCAACCAAGTAGACAACCACGTAAGGTTATAGAGGATTCTTCCACCATGAATACAACGTTTGAAGTTGAAGAAAATGAAAGTGATGACAACTCTATCATTTTAAAACGAAAAAATCTAAAGGCAACACTTCTAAGGACTTTTGAGGAACTGAAGTTGTATGGTGAATACTACATATTTCCTAAAAAAGAAATGCCAAGAAGTTTCTTCAATTTTCAACTAGAGGAGCCTGAAATCACAATCAAGATACCTCCAAATTTGCATAAAGATAAGAAGTGGATGGGGTGTGCCTTTTTTGTAGTATTTTCAGTTGATGAGAATTCACCAAAATCTCATTCCTTCTCTTACCAAGTGGACAATGATGAATATACAATGGAAAGAGAATCAGTTATTCGCTTGAATACGGAGTTGTTCGACGATTCCCATCAACTTTGGATGTTTTTCGAGCCTCGTGGTGTTTATCCATATAGATTAAATCAATGGAGGCATCTTTGTTTCACATTCGTATGCAATAACCCAGACTTTAAGGCTGTTCTTTGTGGTGCACGTCTTGTTTATAAGCAAGATGTTGAAGGATTTGTGAACACAATTGTGAGTAATGTGTTGAGTTTACCAGTTGAATTGCTCGAATTTTATGATCAAATGTATGTTGAAGGCATGTTAAGGAATATACTTTATCATAAGTATGATCCAAAGCATAAGCAGTGGGTTGAGGAACAAAATTCAAATCCTCATAATTCTCAAGAAGATTCAAGTTCTTGTAGTTCAAATATGGAAAGAAGCCTCATTTTGCAACTCAAAGAAAGCATTCCTTCTTTCCTTCAAAAGGATTCAAAGGATCGGTTTGGAAACACATTTGATTTCGTTATTCCAAAGAGAAACTTTCCCCCGGCACTGCTTAATCAACTATCTCCAGAGAATCCTACAGGAGTCCAATTACCTCCAAGTTTATATACTAATAATGATTGGTTGGGATTTGTAGTTTGCACTCTCTTCCAAATCAACAAACATCCCACAGCAATACTCAACAATGTTTGCTCAATTTCAAGGCATGAACTCATTTGTCAATTTGCGATTGAGATTGGATTAATTGAACCATTACACACTCATGGTATCACTGAGGACAGATCCATTTGGCTTCAGGAACGCCAATTTGTTTGGCTTTATTACACCCCAAGACACACATATGGTGAAATCTTTCGTCAATGGTCTTCTGTTTGGGCTGTTATTGAAGCTGATACCCCTGATTTGATGGTGAGCTGTTGTGGAATGAGTTTAGTATACAAGAAAGATGCGGCAGTGATCGACAAGATATTGATGAGAGCCATTCAATGA

>Csa009781

ATGGGTGGCATTGGTAAGACCACAATAGCTAGGGTCTGTTATGAGCGAATTCGTGACGAATTTGAAGCTCATTGCTTCCTCTCCAACGTTCGAGAGAATTATATCAGAACCCTTGGGAACCTTTCATGTTTACAAACCAAACTCCTTTCAAGCATGTTTTCGCTTAAAAACAATCACATAATGGATGTTGAAGAAGGTACCGCTATGATCAATAAAGCCATTTTTCGAAAAAAGACACTTCTCGTCCTTGACGACGTGGATTCTTCGGATCAAATCAAAGGATTGATTCCAGACAACAACTCTTTTGGCAATGGAAGTAGAGTCATCATCACAACACGGAATGCGGATTTTCTTTCGAATGAATTTGGGGTGAAAAGAATTTTTGAAATGGATGAACTTAAATATGAGGAAGCTCTTCAACTTCTTAGTTTGAGTGCTTTTATGAAAACATGTCCAAAAGAAGGTTACTTGGAACACTCCAAGAAGATTGTAAAGGTTGTGGGAGGCCACCCTCTTGCACTCAAATTGTTAGGGTCGTCTCTAAGAAACAAAAATTTGAGTGTGTGGAATGAGGTGATAGAAGAGGTTGGAGGAGGTGGGAATATTCATGAAAAAATTTTCAAGTGTCTTAAAGTGAGTTATGATGGGTTGGATGAAAGGGAGAGAGAGATATTTCTTGACGTTGCTTGCTTCTTCAATGGGAAGAGAAGAGAAGTTGTAGAAGAGATATTAAATGGATGTGGTTTCTATGCCAAAACAAGGATTGAACTTCTTATTCAAAAGTCTCTCTTAACTCTTTCTTATGACAATAAGTTACATATGCATAATTTATTGCAAGAAATGGGTCGAAAGATTGTTCGGGATAAGCATGTTCGAGATCGATTAATGTGCCACAAAGATATAAAAAGTGTGGTGACAGAGGCATTGATCCAAAGCATATTTTTCAAATCAAGTTCAAAGAATATGGTGGAATTTCCAATTTTGTTTTCAAGAATGCACCAACTTAGGCTGCTTAATTTTCGCAATGTGAGACTGAAAAACAAGTTGGAATATAGCATTCCAAGTGAGTTAAGGTATTTGAAGTGGAAAGGATATCCGTTGGAGTTTCTGCCAATCGATAGCTCTGAAGAATGTAAGCTTATTGAGCTTCACATGTGCCATAGCAATCTCAAACAATTTTGGCAACAAGAAAAGAATTTGGTGGAGCTGAAGTATATCAAACTCAATAGTTCTCAAAAGTTGTCCAAAACTCCAAACTTTGCAAACATTCCAAATCTCAAAAGATTAGAGCTTGAAGATTGCACAAGTTTAGTCAACATTCATCCATCAATTTTCACTGCAGAAAAACTCATATTCTTGAGTTTGAAAGATTGCATCAATCTCACCAATCTTCCTTCTCACATTAACATCAAGGTTCTTGAAGTCTTGATTCTCTCTGGTTGTTCAAAAGTAAAAAAAGTCCCTGAATTTTCAGGTAACACTAATAGATTACTCCAACTCCATTTGGATGGTACCTCCATATCAAACCTACCTTCATCAATTGCAAGCTTGAGTCATCTAACAATATTGAGTTTAGCCAACTGCAAAATGTTAATCGACATTTCGAACGCGATTGAGATGACATCTCTCCAAAGCTTAGATGTTTCTGGATGTTCGAAGCTTGGAAGTAGAAAAGGAAAGGGGGACAATGTCGAATTGGGGGAGGTCAACGTGAGAGAAACCACACGAAGAAGAAGAAACGACGACTGTAACAATATTTTCAAAGAAATCTTCCTTTGGTTATGCAACACTCCAGCTACTGGCATTTTTGGGATCCCATCATTAGCTGGTTTGTACTCTCTTACAAAACTAAACTTGAAGGATTGCAACCTTGAAGTAATCCCACAAGGGATTGAGTGTATGGTGTCATTGGTAGAGCTCGACTTGAGTGGCAATAATTTCTCTCATCTTCCAACAAGCATATCAAGACTTCATAACTTGAAAAGATTGAGGATAAACCAATGCAAAAAGCTTGTACATTTCCCAAAGTTACCTCCAAGGATCTTGTTTTTGACGTCAAAGGATTGCATTTCATTGAAAGATTTTATAGATATTTCAAAAGTTGATAATTTATACATAATGAAAGAAGTGAACCTTTTGAACTGCTACCAGATGGCTAACAACAAAGACTTCCATAGATTGATCATTTCTTCGATGCAGAAGATGTTCTTTCGAAAAGGAACATTCAACATCATGATTCCGGGGAGTGAGATTCCCGATTGGTTTACAACAAGGAAAATGGGATCTTCGGTATGCATGGAGTGGGATCCAGATGCCCCAAACACCAACATGATTCGATTTGCGCTCTGCGTCGTTATTGGTCTGAGTGACAAAAGCGACGTTTGCAATGTTTCGTCCTTCACCATTATCGCATCAGTGACTGGAAAAGACCGTAACGACACGAATTTGAAGAATGGAGATGATCTTCTGGTTGATGGATTTCTTGTTTCAGGGATGAAGAAGTTAGACCATATATGGATGTTTGTTTTGCCACGAACTGGGACTCTGCTAAGAAAGATTAGCAACTATAAAGAGATTAAGTTTAGATTCTTACTTCAAGCTATTAACAATAAAAGTAGTTA

>Csa010121

ATGGCGCTGGAATTGGTGGGTGGGGCTGTTTTGGGGGCTGTCGTTGGGGAGCTATTCAAAGCGATCTTGAATCTGGGTGAAAGGGCCATCAGTTTCAATCCTGTTCTTAAGGATATCCGTTCCAAGCTTAATGCTATAATGCCTTTGGTGAAGCAAATCGATGAGCTTAATGATTATCTCGATTACCCAAAAGAAGAAACAGAGAAATTGAGGGGTCTGATGGATGAAGGGAAGCAGTTGCTTCTCCAGTGCGGCGATGTGAAATTGGGGGATCTTAATTATTTGAAGAGACCATCTTACACCCAAAAGCTTCGGGAATTGGATACTGCACTTCGAAGCTTCATGGATGTTTTGATGTTGCAGATGGCTAGAGATCAGAAGAAGAACATGAAGATGATGAACCAAATGATGGAGATCATTTGTAGACTTGATAATAGAGGTGGGTCGAGTAAACCTATGGATTTGTTTGTTCCACCATGTCTGGTTCCTCAACTGCGAGAAGAAACCGTTGGGTTGGAGAAGCCAGTTAAGGAGTTGAAGGTGAAACTTCTCAAAAATGGGGTTCAAATGTTGGTGGTGACAGCTCCTGGTGGCTGCGGAAAAACCACACTGGCCTTAAAATTTTGCCACGACAAAGAAGTCAAAGATATATTCCAGGAGAAGATCTTTGTCCCAGTTTCAAGAAAACCAGATTTGAAGCTTATATTGAAAGATATAATTGAAAGCCTTAGAGGAATTCAATTGCCTGATTTGCAAAGTGATGAACGTGCATTCTGCTATTTAGAATTGTGGTTGAAGCAGACAAGTGTAAATCGTCCTGTTTTGATTGTGTTAGATGATGTGTGGAGTGGGCAAGAATCTGAAGTTCTTCTTGATAAGCTGTTTCAATTGCCTTGCTGCAAGATCTTGGTCACTTCTAGGTTTTATTTCCCAAGATTTAGTGAGTCTTATTATTTGGAACCTTTGAACCATGAGAATGCAGTACAACTTTTTCGTCGTGCAGCATCACTGGACAAAGGAATTTCTAAGCTCCCCGATGATGAAACTATAATTGGGGGATGCAAGAGACTACCTCTTGCACTGAAGGTAATCGGGAGGTCTCTTTCCCACAAACCGACATCTGTTTGGAAAGTAACGGGGAGGAATTTGGCTAGAAGTGGCTCCATATTTGATTCTGACAATGAACTTCTTGAATGCCTTCAGAGCAGTTTGGATGTCTTGGATGATAACATGGTAACTAAGAAGAGTTTCATGGATTTAGGCTCTTTTCATGAAGATCAAAGAATTTCTGCTTCTACCTTCATTGACATGTGCACAGTTTTGTACACACTAGACGAAAGTGAAGCAATGGTTACCCTTGACGAACTATCCTCTCGAAGTCTAGTTAATTTTGTCACAGCGAGAAAATATGGATATGATGATGACTTTTATGAAGAGTACTCTTTTACTCAGCATGATATTCTCAGAGATTTGGCTATTCACTTGATGAATATGGAGCCCATAGAACAAAGGAAAAGATTGATCTTAGACATTAATGGAAATGATCTTCCCAAATGGTGGGTTGATCAAGAAAAGCATACTTCCTATGCTCGCCTTATATCCATAACCACAGATAAGAGATTCTCAGCAAGTTGGCCTGACATGGAAGCACCTGAAGTGGAGGTTCTGATTCTTAATCTTCAGTCAAGAACTTACAACTTGCCTGGGTTCATCAAAAGAATGAATAAGCTGAAAGTTTTGATAATCACATATTTTGGTTCTTTTCTAACTGAGGTGACAAGTGAAGATAATCAACTACTCGACAGCCTAACAAGTCTTGAACGAATCAGGTTTGAGCGGATTTCAGTTCCTATCTTTAGTAATCCAAACCCGAAACCACTGATAAATCTGCAGAAAATATCCTTCTTTATGTGCAAATTTGGTCAAACATTCATGGATCCTTCAACCCCAATCTCAGATTTGTTGCCAAACCTGCTGGAGATTTCCATAGACTTCTGCAACAATTTGAGTGAAGTCCCCAATAGGTTGTGTGAAATTGTCAGCTTGCAGAAGCTGAGCATTACAAATTGCCATGGACTATCTTCCTTGCCAGAAGATGTAGGGAAGTTGATTAATCTAAAAAATCTAAGGCTAAGATCTTGCATTCATTTAGAAGAGTTTCCAGAGTCGACAACGAAGCTTCGGGAATTAGTCCTGCTTGATATATCTAACTGTATTGGTCTTGCCAAGCTTCCCGAGAAGATTGGTGAATTTCATAATTTAGAAAAGCTTGACATGAGACACTGCTGGAGTTTGAGCAAGCTGCCACTGTCGATTGGAAAGCTGAAAAATGTGAAGTTTTTATGTGATAGAGAGGTTGGAGAGTGGTTGAGAAAGGTTGCACCTCGCCTTGCCAAACAGGTGAAAGTGCAAGAGGAAGAAGCCAACCTGGAGTGGCTTGGTTTTTGA

>Csa012256

ATGGCTGAAGCTATTCTCTTCAACCTTACTGCAGACATCATATTCAAACTGGGTTCTTCAGCACTCCGACAGTTTGGATCTCTACGGGGCGGTGTCAAGGATGATTTTGACAAACTCTGGCACTCTCTTTCTGCCATTCAAGCTGTTCTTCACGACGCGGAGGAGAAGCAGTTCAAGGACCATGCGGTCGAAGTTTGGGTTTCAAGGCTTAAGGATGTTTTGTACGAGATTGATGACTTGATCGACGAGTTCTCTTACCAAATCTTGAGAAGGCAAGTTCTGCAAAGTAACAGAAAACAAGTACGTACCCTCTTCTCCAAATTTATAACTAATTGGAAAATAGGCCACAAAATCAAGGAAATCAGTCAGAGGCTACAAAATATTAATGAAGATAAAATTCAATTTAGCTTTTGTAAGCATGTGATAGAGAGAAGAGATGATGATGATGAAGGGTTGAGAAAGAGACGGGAGACTCACTCTTTTATACTTGAAGATGAAGTGATTGGTAGGAATGATGACAAGGAAGCAGTCATAAATCTTCTACTAAATTCCAACACCAAAGAGGATATTGCAATTGTTTCCATTGTTGGAATGCCAGGATTTGGAAAAACTGCCCTTGCCCAATTTATTTATAACCATAAGAGGATAATGACTCAATTTCAGTTGAAAATATGGGTGTGTGTTTCTGACGAATTTGATCTGAAAATTACTATCCAAAAGATAATAGAGTCTGCAACCGGGAAGAAGCCTAAATCATTACTTCAAATGGATCCATTACAATGTGAGCTTAGAAAGCAAATTGATGGAAAGAAATATTTGATCGTCATGGATGATGTGTGGAATGAGAAAAAAGAGAAATGGTTACATCTGAAAAGATTGTTGATGGGCGGTGCAAAGGGTAGTAGGATTTTGATCACAACACGCAGTGAACAAGTTGCAAAAACTTTTGACTCTACTTTCGTTCATCTATTACAAATTTTGGATGCATCCAATTCTTGGTTATTGTTTCAAAAGATGATTGGTTTAGAAGAACATTCAAATAATCAAGAGATCGAGCTTGATCAAAAGAATTCAAATTTGATCCAAATCGGCATGGAGATTGTGTCAACGTTAAGAGGTGTTCCGCTTTTAATAAGAACCATTGGAGGACTTTTAAAAGATAATAAATCAGAAAGATTTTGGTTGTCTTTTAAGAATAAGGAACTTTATCAAGTTTTGGGACGAGGACAAGATGCTCTGAAAGAAATACAATTGTTTCTTGAGCTTAGTTATAAATATCTCCCATCGTCTAACTTGAAACAATGTTTCCTATATTGTGCTTTGTTCCCCAAAGATTATCGAATTAAAAAGGATGAACTTATATTACTATGGAGAGCACAAGGTTTCATTCAACAAAATGGCAACAACGACGACAATAGTTCCCTCGTTGATATTGGTGAAGATTATTTCATGGAGTTATTATCAAGGTCGTTTTTTCAAGAGGTTGAAAAAAATGATTTTGGAGATATAATAACATGTAAGATGCATGATTTGATGCACGATCTTGCTTGTTCGATAACAAATAATGAATGTGTGCGTGGACTGAAGGGAAATGTCATCGACAAAAGAACTCATCACCTTTCTTTTGAAAAAGTTAGTCATGAAGATCAACTTATGGGATCATTATCTAAGGCAACTCATTTGAGGACACTTTTTATTCAAGATGTTTGTTCACGATGTAACTTGGAAGAAACCTTCCACAATATTTTCCAATTGCGAACATTGCACTTAAATTTGTATAGTCCAACCAAATTTGCAAAGACTTGGAAGTTTATTAGTAAGTTGAAACATTTGAGATATTTGCATCTTAAAAATTCGTTTTGTGTTACATATCTTCCAGATTCCATTTTAGAGTTGTATAATTTAGAAACATTTATCTTTCAAAGCTCTTTGTTAAAAAAGTTGCCTAGTAATGTAGGAAACTTGATCAACCTTAAGCATTTGGATCTTTCTTCTCATTTAAATTTAGAATTCCTTCCCGATTCTATTACAAAATTGTATAAGTTGGAAGCACTTATACTTCACGGTTGTTCCAATTTAAAAGAATTGCCCAAATATACTAAAAGGTTGATCAACCTTAAGAGTCTTGTTTTGTACGGATGTTCGGCTCTCACTCATATGCCAAAAGGATTAAGTGAGATGACTAATCTTCAAACATTGACTACATTTGTATTGGGAAAGAATATTGGTGGTGAGTTAAAGGAGTTGGAAGGACTTACTAAATTAAGGGGAGGATTAAGCATTAAACATTTGGAATCTTGTACCAGCATTGTTGATCAACAAATGAAGAGTAAGTTGTTGCAACTAAAGTCTGGTCTTCAAAAGTTGGAGTTACAATGGAAGAAACCGAAAATTGGTGATGATCAGTTGGAGGATGTGATGTACGAAAGTGTTTTAGATTGCTTACAACCACATTCAAATCTTAAAGAGATACGTATTGATGGATATGGTGGAGTAAATTTATGTAATTGGGTATCCTCTAATAAGTCCCTTGGTTGTCTTGTCACTATATATCTTTATCGTTGTAAAAGATTACGACATCTCTTCAGATTAGATCAATTTCCTAATCTCAAGTATCTTACGCTTCAAAACTTACCCAACATCGAGTACATGATTGTAGACAATGATGATTCAGTTTCTTCATCAACAATTTTTCCATGCCTAAAGAAATTTACTATTTCAAAAATGCCTAAGTTGGTGAGCTGGTGCAAGGATTCAACCTCAACCAAATCTCCTACAGTAATATTTCCTCACCTTTCTAGTTTAATGATTCGCGGTCCTTGCCGACTACATATGTTGAAGTATTGGCATGCACCTAAGTTGAAACTCTTGCAAATTAGTGATTCAGAGGATGAGTTGAATGTTGTACCATTGAAAATTTATGAAAACCTCACCTCTCTATTTCTTCACAATTTGAGTAGAGTGGAGTACTTGCCCGAGTGTTGGCAACATTATATGACATCTCTACAACTTCTTTATTTAAGCAAATGTGAAAATTTAAAGAGCTTACCGGGATGGATTGGCAACCTCACCTCACTCACAGGATTGAAAATTTCAACATGTGACAAGTTAACTATGCTACCTGAAGAAATTGACAATCTTACCTCACTTACAAATTTGGATATTTCTTATTGCAAAAACTTAGCTTTTCTACCAGAAGGGATTAAACACATCCATAATTTACGATCGATAGCAGTTATTGGTTGTCCTATATTAGAAGAATGGTGCAAGAAAAACAGACGAGAAGATTGGCCTAAGATCGAGTACTATATTTCCAGACTTAGTCATTTAATCAAGAATTCATTCATTACTTCATGA

>Csa012262

ATGGTGGACGCCGCATCATCTCCGACCACCAGACGCTGGACGTACGACGTTTTCTTAAGCTTTAGAGGTGAGGACACTCGTCCGAAGTTCACCAAACACCTTTACCAAGCCCTCGATGCTGCCGGAGTCAACACGTTTCGTGACGACGTCGAACTCCGGCAAGGAGACGCCGTACGCTCAGAGCTCGTGGTAGCGATTAAAAAATCGAGGATAGCGGTCGTGGTGTTCTCCAGTGGCTATGCCGACTCACAGTGGTGCCTAGGGGAGATCGCCGAGATCATGGATTGCCGGACCGCGGATGGCCAACTGGTCCTTCCGATCTTCTATGAGGTTGATCCGTCGGACGTTCGGAAGCAGATGGGGAGGTTTGCGGCCGCGTTTGAGAAGGTCCTGCGGTGGAGGGCGGCGCTCACGGAGGCCGCTAGCTTGTCCGGCTGGGACTTGAGGCAGCTAGCCGATGGGCATGAAGGAAAATTCATACAGAAAATAGTGGAAAGGGTTCAAAGCGAACTGCGAGTGACATATTTGGAAGTCGCCATCTACCCTGTTGGCATTGATCTTCGCCTCAAACACTTGATCTCATTAATGGCCATTTCTACAAACCACTCCACTCTCGTCCTCGGCATCTATGGCATGAGCGGCATTGGGAAAACCACTCTCTCTAAAGCACTCTTCAACCACTTCTTCCACTTCTTCAATTCTAGATCTTTTCTCCCCAACATCAACTCCCTCTCTACCTCCTCTCCCGACGGTCTCCTTCGACTCCAACAAACTCTCCTCTCCGATCTCCTCATCGCCACAAACCTCCGCTCTCGTTCCTCAACCACCACCGACTCCACCGTCGTTCGGATGCAGGAAAGACTCCAAAACAAAAAGGTCTTGGTAGTCCTCGACGACCTGGATCGTATCGAACAAGCAAATGCACTAGCAATACGGGACCGAAGATGGTTTGGAGACGGAAGCCGAATCATAATCACAACACGAAACAAACAAATTTTGGACACTCTAAAAGTCGACGAAGTTTACAACATGGAATCCAATCTACTGAACGACGAGGAATCGTTGGAGCTTTTTAGCTACCACGCATTCCGGGAGCAAAATCCACCAGAGGAGCTTTTGGAATGTTCGAAATCCATCGTTTCGTACTGCGGAAGCCTTCCTCTAGCTCTGGAAATCCTGGGTGGGTCATTCTTCGGAGGGAGACCGATGGAGGAATGGAGATCAGCGATGGAGAGATTGAAGAGGATTCCGGCGTGGGATTTGCAAGAGAAGCTTCGAATAGGGTTTGAAGGATTGAGAGATGAGATGGAGAGGGAGATATTTCTTGATGTATGTTGCTATTTTGTGGGAATGAAAGAGGAATTGGTAGTGAAGATTATGGATGGATGTGGAATGTATGGAGAAAGTGGATTGAGAGGGTTGAAATGGAGGTGTTTGGTTGGTGTTGAGTTTTGGAGTGGGAGGTTGAAGATGCATGATTTGGTTAGGGACATGGGGAGGGAGATTGTGAGGCAAACATGTGTGAAGGAACCTGCTAGACGGTCCAGGGTTTGGCTTTATCATGAGGCTCTCAAAATCTTACTCCATCAGAACGGAAGTGAAAACATTGAAGGACTTGCAATAGACATGGGTAAAGGAAATAACAAGGAGAAATTCAGATTGGAAGCATTTGGGAAAATGAGAAATCTAAGGTTACTCAAACTCAACTATGTGCATCTCATTGGAAGTAATTTTGAGCATATAATAAGCAAAGAATTAAGGTGGATTTGTTGGCATGGATTCCCTTTGAAGTCTATTCCAAGCTCATTTTATCAAGGAAACCTTGTTGCCATTGACATGAGATATAGCAGCTTGATACACCCTTGGACTTGGAGGGATTCACAGATTCTTGAGAATCTAAAAGTTCTAAACCTAAGCCACTCTGAAAAGCTAAAGAAGTCCCCAAACTTCACAAAGCTCCCAAACCTAGAGCAGCTAAAACTCAAGAATTGCACAGCCTTATCAAGCCTCCACCCCTCCATTGGTCAACTTTGTAAGCTTCATCTCATCAACCTCCAAAACTGTACAAATCTCTCGTCTTTACCAACCTCCATCTACAACCTCCACTCCCTCCAAACTTTCATCATCTCTGGCTGCTCCAAGATCGACTGCCTCCACGACGACCTCGGTCACCTTGAGTCCCTCACCACCCTTCTCGCTGACCGAACCGCCATATCCCACATTCCTTTCTCCATTGTCAAGTTGAAGAAACTCACTGACTTGTCTCTATGTGGTTGTAACTGCAGATCAGGATCGGGAAGCTCGGCATCGCTGCCATGGAGGCTGGTTTCATGGGCATTGCCAAGACCAAACCAAACATGCACAGCCCTAACTCTTCCATCTTCATTACAAGGTTTGAGCTCTCTAACAGAGTTGAGTCTACAAAATTGCAATCTCGAGTCACTTCCAATTGACATTGGGAGCTTGAGTGAACTAAAGAAGTTGAATCTTGGAGGCAACAAAAATTTGAGGGTTTTGGGGACTGAACTTTGTGGACTTTTGAAACTGAATGAGCTGAATGTGGAGAATTGTGGGAGGCTTGAGTTCATCCAAGAATTTCCAAAGAATATGAGAAGTTTTTGTGCTACCAGTTGTAAGTCATTGGTGAGAACTCCTGATGTTTCCATGTTTGAAAGAGCACCTAATATGATTCTCACCAATTGTTGTGCATTGCTTGAGGTTTGTGGATTGGACAAATTGGAGTGCTCTACTAATATTCGTATGGCCGGTTGTTCGAATCTCTCTACTGACTTTAGGATGAGCCTTCTTGAGAAATGGAGTGGGGATGGCTTAGGCAGCTTGTGTGTAGCAGGCAACCAACTCCCCAAATGCTTACACTTCTTCACCACACACCCACCCTTAACCTTCCAAGTTCCCAACATCAACAATAACATTCTTCTTGGCCTCACAATCTTTGCCATTTTCACTCACTTGATCACTGACATAAACCACTCCCCATCTCTCCGTATCATCAACAGAACCTCATCCCGAACGCATATCTACCGAATGCTTGGCCTCCACTATGACTCCCTCAACATCCATGCTCACCACATTTGGGCCATCCACCTTCCCTTCTCTTATGGTTATTGTCTCAACCCTGGGGACGACATAGAGCTTCACATTCCTAATGCCAATGCCTATGGAGTTCGGTTGGTGTATCATTTAGATGAACCTCAGCCAATCGTGAGTTTTGCCCCATCGATGGTGGTGGAAGAACAAGGTGGTGCTAGCAACGACCATGACAATGACGATTCTACATATCATGTTGTTGATCAACAAATGCAAGAGAGTGGCTCTAATACTACTTCTCCATGGTTGCTTAGGGTGGTAGATTAA

>Csa012269

ATGGCTTTGCGTCAAAGAGGAATCAATGTTTTTATAGATAACAAGATTTCAAGGGGTGAAGAAATTTCTGCATCTCTTTTGGAAGCTATTGAAGGATCCAAGATCTCCATTGTCATAATCTCTGAAAATTATGCTTCTTCCAGGTGGTGTTTGAATGAGCTGGTGAAAATCATTATGTGTAACAAATTGAGAGGACAAGTGGTTTTACCAATTTTCTACAAAGTGGATCCATCTGAAGTAAGAAAACAAAGTGGAAAATTTGGAGAAGAATTTGCCAAACTTGAAGTTAGATTCTCGTCGGAGAAGATGCAAGCATGGAGGGAGGCCATGATCTCTGTTTCTCATATGTCTGGATGGCCGGTTCCTAAGAAAGATGACGAGGCCAATTTGATTCAAAGAATTGTTCAAGAAGTCTGGAAGAAATTAAATCGTGGAACAAGAGAGATGCGTGTACCTAAATATCCAGTTGGAATAGATAGACAAGTTAATAATATACTCTCCCAAGTTATGTCTGATGAAATAATTACTATGGTTGGATTATATGGAATTGGAGGTATTGGCAAAACAACTTTGGCCAGAGCTTTGTACAATAAAATTGTGGATGACTTTGAAAGTTGTTGCTTTTTGGCAAATGTTAGAGAAGCTTCAAATCAATATCGGGGTCTTGTTGAACTCCAAAAGGAGCTACTTCGTGAGATTCTAATGGATGATTCAATCAAAGTTAGCAATCTCGATATAGGAATTAGCATCATAAGGGATCGACTATGCTCAAGAAAGATTCTTTTGATTCTTGATGATGTTGATACGAGTGAACAACTAGAAGCATTAGCAGGAGGACATGATTGGTTTGGACCAGGAAGTGTGGTCATTGCGACAACAAGAAACAAACACTTACTTGCTATTAATGAATTTGATATATTGCAAAGTGTTCAGGGATTGAATGATGTTGAAGCCTTCGAGCTTTTTAGCTGGCATGCTTTTAAGATGAGTTGTCCATCAAGTCATTATTTATACCTAATTTCAAAACGTGCCGTAAGTTATTGTAAAGGTCTTCCCTTGGCTTTGGAAGTTGTAGGTTCATTCCTTTATTCTATTGAGCCATCCAAGCTTAAACTTATATTGGATGAATATGAAAACCAATATCTTGACAAGGGCATCCAAGATCCTCTTCGAATAAGTTATGATGGACTTGAAGATGAAGTAAAAGAAATTTTTCTTTATATTTCTTGTTGCTTTGTAGGAGAAGACATCAACAAAGTTAAAATGAAGTTAGAAGCATGTGGTTGTTTATGTTTGGAAAAAGGAACAACAAAACTCATGAATCTATCACTTCTGACCATTGATAAATCCAATCGGGTTGAAATGCATAATTTAATACAACATATGGGTCGCACAATTCATCTTTTGAAGACTTCTACATCTCATAAAAGAAAAAGATTGTTGATTAAAGATGACGCTATGGATGTCTTAAATGGGAATAAGGAAGCAAAAGGAGTTAAAGCCATAAAATTAAGTTTTCCTAAAGCTACCGAGTTGGACATTGATTCAAGAGCTTTTGAAAAAGTGAAAAATGTGGTAGTACTCGAAGTTGGCAATGTCACATCTTCAAAAGGTACTGATCTTGAGTATCTACCTAGTAGCTTAAGGTGGATGAATTGGCCTCATTTTCCTTTTCCATCTTTGCCTACAACCTACACAATGGAGAACCTTATGGAATTGAAATTGCCATATAGCTCCATCAAACATTTTGGAAGAGGATTCATGAGTGGTGAACGGTTGAAGGAAATTGATCTTAGTGGCTCTGAGTTTTTAGTGGAAATTGCTGATTTATCTACTGCAACAAACCTTGAAAAGCTGAATCTTTTAGGCAATGTTAAGGGCTTTGAGCAGTTTCCACCCCACCTCAAGTTGAAATCCCTTAAACTTTTGTCAATGAAAAATTGTAGAATAGATGAATGGTGTCCTCAATTTAGCGAAGAAATGAAGTCTAGCCTAGAAGAATTGTTGATTCAATATAGTACTGTAATTAATCAGCTATCTCCAACAATTGGATATCTTACTAGCCTAAAACGTTTGTTTATCATAGAGTGCATGAAGCTCAAAACTCTTCCAAGTACAATTTATCGTTTAAGGAATCTTACTTTTTTAAGTGTCATTAAATCTGATCTATCAACCTTTCCTTCCTTAAATAATCCTTCTTCACCTTCCTTATTTCCCTACCTAACATCATTACACCTTTCCAATTGTAAGATAACAAATTTGGATTTCTTAGAAACAATGGTTCATGTTGCCCCTACATTGGAACGGTTGGACTTATCTAGAAACAACATTTGTAGATTACCCTCATGTATTATTAATTTTAAATTCTTGAAATCTCTTGTTACAATGGAATGCAAGTTGCTTGAAGAAATTCCAAAGGTTCCAAAAGGAGTAGTTTATATGAATGCTATAGGGTGCATATCATTGACCAGATTTCCTGACAACATACCTGATTTCATATGCTGTGATGATAATGTGGTGCGTATCATTGTTCTTTCTCATCACCTCATGACCTCATGA

>Csa012278

ATGGAGTTGTGTGCCGGTGCCATTGTTAATCCAATCGCAGAAAAAATCGCCAACTGCACGGTGGATCCGGTTTTCCGGCAACTAGATTATTTGCTCCACTTTAAAACCAATGTGAATGATCTCAAAGATCAAGGCAAGAAGCTGGTGGAAACCAGAGATTTTGTTCAACATTCTGTCGACTCCGCCAAAACCAATGGAAGAGCTACCAAATTGGCTGTGGCAGTTGATAAAGCCATTCAAGGTGGGAGTTTCGAGAGAGTTGGGTTCCGTGTAACTCCACAAGAAATTATGACGCTAAGGAACAATAAGAAGTTCGAAGCCTTTGAATCTAGGGTTTTGATTCTGAAGGAGATAATTGAAGCGGTTGGCGATGCTAATGCGAGGGTGATTGTGGTACATGGGATGGCGGGAGTTGGGAAAACCACCCTAGTTGAAGAAATTGCAAGATTGGCCAAGGAGGGGAAGCTTTTTGATGCTATAGCAATGGTGACTGTAAAGCACATTCCAAACATTAAGAAAATACAGGGGGAGATTGCTGATCAATTGGGGTTGAAATTTGAAGAGGAAAAGGAACGAATTAGGGCCGATCGACTACGTCGAAGGTTAGAGATGGAGAAGAAGGTGTTAGTGGTTTTGGATGATGTTTGGAGTAGGCTTGATTTGGAAGCTGTTGGAATTTCTAGCCATCACAAGGGATGTAAGATACTTGTAACTTCTAGAAAGGATGATTTGTTTTTCAATGATTTTGGTACTCAGAAAAATATATATATCAATATTCTGTCAAAAAAAGAAGCTAGGGATTTTTTCAACAAGGTGGCATGTGATTCTGTTGAATCTTCTGATGATACTGATCCTGAAATGGAAGCTGTTGCTACTGAATTGGCAGATGAATGTGGAGGATTGCCACTTTCTCTTGCAACTGTTGGACAAGCCTTGAAAGGTAAAGGGCTTCCAAGTTGGAATGATGCCTTGCAAGGAATGAAGTTTCCTGGCGAACCCAGTAACTATGGGGTGAATAAAGTGGCATATTTGTCTCTGAAAGTGAGTTATAGATCTCTAAACAGAGAAGAAGCCAGATCACTATTCTTACTATGTAGCTTGTTTCCAGAAGATTATCAAATTAACATCAAATACTTGTTGATGTATGCCATGGGTTTGGGGTTATTAAACGCCATGAGTTCTCTAGCAATGGCAAAATGGAGAATACTTTCTTTGGTTGATGAGCTCAAAACTTCTCACTTGTTGCTTGATGGGGTTGATAACGATTTTGTGAAAATGCACGATATAGTTCGAGATACAGCAATTTTGATTGCGTCGAAAATGAAGTCCAAGTATTTGGTTAGACATGGTGCTGGAGAGAGTTTGTGGCCCCCAATGGATGAGTTCAAAGATTACACTGCAATCTCATTAGGTTGCAGTGATCACTCGGAACTCCCAGAATTTATATGTCCACAGCTTAGATTCTTATTACTGGTAGGAAAAAGAACATCTTTGCGATTACCTGAAAAGTTCTTTGCAGGTATGCAGGAACTACGAGTTTTAGATCTCACTGGCTTATGTATTCAGCGGCTTCCACCATCAATCGACCAACTGGTAAATCTTCAAACATTGTGTTTAGATGACTGTGTTTTGCCAGACATGTCTGTAGTTGGTGAACTGAAAAAGCTTGAAATTCTTAGCTTGAGAGCATCTGATATTATTGCACTTCCTAGAGTAATTGGGGAACTTACCAATTTGAAAATGTTGAATTTGTCTGATTGTTCTAAACTCAAGGTGATCCCTGCTAACCTTTTATCTAGGTTGATAGGGTTGTCTGAGCTATACATGGACAATAGTTTTAAACATTGGAATGTAGGACAGATGGAAGGTTATGTTAATGCAAGGATTTCTGAACTAGACAACCTGCCACGGTTGACCACTCTACATGTCCATATTCCAAATCCCACCATTCTACCACATGCCTTTGTCTTTAGAAAATTGAGTGGTTACAGAATACTAATTGGAGATAGATGGGATTGGTCTGGCAATTATGAAACTTCAAGGACCTTGAAACTCAAGCTTGATAGTAGCATTCAGAGAGAGGATGCAATTCAAGCACTTCTAGAGAATATTGAAGATCTGTATTTAGATGAATTAGAAAGTGTCAAGAATATTCTATTCAGTCTAGACTATAAAGGCTTTCCGAAATTGAAAGGTTTGCGTGTCAAAAACAATGGTGAAATTGTGACTGTTGTCAACTCGGATAACATGCATCATCCACACAGTGCCTTTCCATTGTTGGAGTCCTTATTTCTGAAAAATCTAGCTGAACTTGGAAGCATTTGTCGTGGAAAGCTTCCACAAATGTCCTTCCGTAACTTGAAAAGAGTAAAAGTTGAAAGTTGTGACAGATTAAAATTTGTTTTCCCATCTTCTATGGTCAGAGGCCTTATACATCTTCAAAGCCTGGAGATTAGTGAATGTGGCATCATAGAAACTATAGTTTCGAAAAACAAAGAAACAGAAATGCAAATCAATGGTGATAAGTGGGATGAGAACATGATTGAGTTTCCTGAATTGCGTTCTCTGATACTTCAACATCTACCAGCCCTTATGGGTTTCTATTGTCATGATTGCATAACTGTGCCTTCAACCAAAGTGGATTCACGTCAAACAGTTTTTACTATTGAACCTAGTTTTCATCCACTTCTCAGTCAACAGGTTTCCTTCCCCAAATTGGAGACATTAAAATTACACGCTTTGAACTCAGGAAAGATATGGCAGGATCAACTTCCTTCTAGCTTTTATGGCTTTAAAAATCTAACTTCTTTGAGTGTGGAGGGTTGTGCTTCAATAAAATATTTAATGACAATCACTGTGGCTAGAAGCCTTGTGAATCTTGAACGCCTTGAACTAAACGACTGTAAGTTGATGAAAGCTATAATCATTTCAGAAGATCAAGATCTGGACAACAATTACCCTTCCAAATCTATCTTGCAGAACAAGGATGTTTTTGCGAACCTGGAGTCCCTCTTAATCTCTCGCATGGATGCTTTGGAGACATTATGGGTCAATGAAGCTGCTTCAGGATCCTTTACAAAGCTGAAAAAAGTGGACATCAGAAACTGCAAAAAACTTGAGACAATCTTTCCAAATTACATGCTTAACAGAGTGACAAATCTCGAGAGATTAAACGTTACAGATTGCAGTTCCCTAGTGGAGATCTTTCAAGTGAAAGTCCCAGTTAACAATGGCAACCAAGTAAGAGACATTGGAGCTAACCATTTGAAAGAGTTGAAGCTGCTTCGTCTACCTAAACTAAAGCACATATGGAGCTCAGATCCACACAATTTTTTACGCTATCCATCTCTCCAACTTGTTCATACAATTCATTGTCAAAGCCTTTTGAATCTCTTCCCTGTATCCATAGCTAAGGATCTCATACAACTTGAAGTGCTTAAAATACAGTTCTGTGGAGTTGAGGAAATTGTTGCGAAACGAGGAGACGATGGAGATGGAGATGATGCTGCGTCGTTTTTGTTGAGTGGTTTGACATCATTGACTCTTTGGAATTTGTTCGAGTTCAAGAGGTTTTATCCTGGGAAATATACTTTGGATTGTCCATCATTGACAGCGCTAGATGTACGCCATTGCAAATCATTTAAGTTGATGGAAGGAACTTTGGAAAATTCGTCATCAATCTCATCCGCTGTTGAAAAGGTAGAAGTTGAGCAATCTTCACTGAGGGGGGAGTTCGAAAGAAGAGAATCAAAGGAGACATCCACAGGGAAAGAAGAAATAACAACCATAGTGCAAGGTGTAGTTGATGCTGAGTTAATTGAATTAAGAGCTCAATTACGAGCTCTTGTGGCAGGTCAAAACCAAATGATGGAACGCTTAGCCCAACTTACAACAATTCCTCGTGAGCCTGTCTCAAAGTGA

>Csa012551

ATGGGTTCTTCTTTGGTTGGTTTAGCACAATCATCATCGTCTTGTTCTTCAAATTTGAAATGGAGTTATGATGTGTTTTTGAGTTTCAGAGGTGAGGATACTCGAAACAACTTCACTAGTCATCTTGACAGGGCCTTGCGTGAAAAGGGTGTCAATTTCTTCATAGATGACAAGCTAGAGAGGGGTGGTCAAATTTCTGAATCCCTTCTCAAATCTATTGATGGTTCTAAAATTTCCATCATTATTTTCTCCAAAAATTATGCATCTTCCACCTGGTGTTTGGATGAACTGGTGAAAATAGTTCAGTGCATGAAATCCATGGGACATATAGTTTTTCCTGTCTTCTACAAGGTGGATCCATCTGAGGTTCGAAAACAAACTGGTGGGTTTGGTGAAGCATTGGCCAAACATGAAGCTAATGAGTTAATGACCAACAAGGTTCAACCATGGAAGGAAGCTTTGACCACTGCTGCTTCTTTGTCTGGTTGGGATTTAGCAACTAGGAAGAATGAGGCTGATCTTATTCATGACCTTGTTAAGGAGGTGTTGTCTATATTAAATCAAACACAACTACTACATGTAGCCAAGCATCCAGTTGGAATTGATTCTCAACTTAGAGCTGTTGAGGAATTGGCCTCCCATGATGTGCCCGATGGTGTTAACATGGTGGGGATACATGGGATGGGAGGCATTGGTAAGACCACTCTGGCCAAAGCTTTATACAACAAAATCGCTTATCAATTTGAAGCTTGTTGCTTTCTTTCGAATGTTAGAGAAACCTTAGAGCAATTCAAAGACCTGGTTCAACTACAAGAAAAACTACTCAGTGAGATCTTAAAAGATAATGCTTGGAAGGTGGGCAACGTTCATAAAGGAAAGAATATCATTAGGGATCGGTTATGCTCAAAGAAAGTTCTTATCATTCTTGATGATGTGGATAAGGATGAACAATTAGACGCACTAGTTGGTGAACGTGATTGGTTCGGTCGAGGAAGTAAAATCATAGCAACAACAAGAGATCGACATTTACTAGAAAACCATTCATTTGATATAGTATATCCTATTCAGTTGTTGGATCCTAAGAAATCCCTTGAGCTTTTTAGCCTGCATGCTTTTAAGCAAAATCATCCCTCAAGTAATTATGTAGACCTTTCAAAATTTGCTGTAAGTTATTGCAAAGGTCTTCCATTGGCTCTTGTTATTTTGGGTTCTCTTCTCCATAAGAGAGAGCGAAAAATATGGAAAAGTAAATTACATGAACTTGAAAATTCCCTCGAACCAAGTGTTGAAGCTGTTTTTCAAATAGGTTTTAAGGAGCTTCACGAAAGAGTGAAGGAGATTTTTCTTGATATTTCTTGCTTTTTCGTGGGAGAGGATATTAACTACAGTAAGGATGTGTTAAAGGCATGTGATCTCAATCCAGACTATGGAATTATAATTCTTATGGATCTTTCCCTTGTTACTGTTGAAGATGGAAAGATACAAATGCATGATTTAATACAACAAATGGGTCAAACAATTGTTCGCCATGAATCTTTTGAGCCTGCAAAAAGGAGTAGGTTGTGGGAGGCAGAAGGAGCTATCAAGATATTGAAAGAGAAATCTGGAACTAAAGCAGTTAAAGCCATAAAGTTAGACTTGCACTACAAACCTTGGCTGAAAATTGTTGAAGCAGAAGCATTTAGAAACATGAAAAATCTTAGATTGCTTATCCTTCAAAGAGTAGCATACTTCCCTAAAAATATATTTGAGTATTTACCTAATTCGTTGAAGTGGATTGAGTGGTCTACATTTTATGTTAACCAGTCTTCGTCCATAAGTTTTTCTGTGAAAGGTCGGCTTGTCGGACTAGTTATGAAAGGTGTAGTCAACAAACAGCCAAGGATTGCATTTGAGAATTGTAAAACAATGAAGCATGTTGATCTGAGTTATTGTGGCACGTTAAAGGAAACTCCCAACTTCTCTGCCACATTAAACCTTGAGAAATTATATCTTAGGGGATGCACGAGTTTGAAAGTGATTCACGAGTCTGTGGCTTCTCTTAGTAAGCTTGTTACATTGGACCTTGAAGGTTGTGACAACCTAGAAAAGTTTCCAAGCAGCTATCTCATGTTAAAATCTCTTGAAGTTTTGAATCTTAGTAGGTGCAGAAAAATTGAAGAAATTCCTGACTTGTCTGCATCTTCAAACCTTAAGGAACTATATCTCAGGGAATGCGACCGTTTGAGAATAATTCACGACTCTATTGGTCGTTCTCTTGATAAGCTTATTATCTTGGATCTGGAAGGCTGTAAAAACCTTGAAAGGCTACCAATTTACACCAACAAGTTAGAGTCTCTTGAACTTTTGAATCTCGCTTCATGTCTAAAGCTTGAAACTTTTTTTGACAGCTCTTTTAGAAAGTTTCCAAGCCACCTGAAGTTCAAATCTCTTAAAGTTCTGAATCTACGGAATTGTCTAAATCTTGAAGAAATTATTGACTTTTCAATGGCATCAAACCTTGAGATATTAGATCTCAATACTTGCTTCTCTTTAAGAATAATTCACGAGTCTATTGGGTCTCTTGATAAACTTATCACCTTACAACTCGATTTATGCCATAACCTAGAAAAGCTTCCTAGCAGCCTGAAGTTGAAGTCTCTTGATTCTTTGAGTTTCACTAATTGTTACAAGCTTGAACAACTTCCAGAATTTGATGAAAACATGAAATCTTTAAGGGTGATGAATTTGAACGGTACAGCCATAAGGGTGTTACCTTCATCAATTGGATATCTTATTGGGCTCGAGAATTTAAACCTTAATGATTGTGCAAACCTGACTGCCCTTCCAAATGAAATTCATTGGCTAAAAAGTCTCGAGGAACTTCATCTTCGCGGGTGTTCTAAACTCGACATGTTTCCCCCGAGATCAAGCTTAAATTTTTCCCAAGAAAGCTCATATTTCAAGCTGACGGTATTGGATCTCAAAAATTGTAATATATCAAATTCTGATTTCCTCGAAACATTATCTAATGTCTGCACTTCCTTGGAGAAGCTAAATTTGTCAGGAAACACATTCTCTTGTCTACCCTCTCTCCAAAATTTTAAGTCATTAAGGTTTCTTGAATTAAGGAATTGCAAGTTTCTTCAAAATATAATAAAGCTTCCCCATCATTTAGCTCGGGTGAATGCCAGTGGTAGCGAATTGTTGGCTATACGTCCTGATTGCATTGCTGATATGATGTTCGGAAAACAGGACGCTGAATTTAGTGACTCAACAAAAGTGCTCTTCATAACAAACAATGAGATTCCAAAATACTGCAACAAACAAACTACGAGAAGTTCAATGAGTGTTAGGTTTCGTCACAATTTAGATAAGAACATACCAGCTTTGGTTCTGTGTGTAATTTTCAAAGCAGATGGAGATTCATGTGACGAAGCGGAGGGTTTTATTCATTTTGAAGTGTCAATCGACGGACTGAGAGGTATTGTTAAGAAGTCATTGCGGTTTTCACGTCGCTTTAAGTCAAGCCAGGGAGAGCTTCTAATCTTAGAGGATCAAGCTATTGACCGGATTCTGGGGTTGAGGGAAGTTGTAACCCAGAGATCTAGGAACCTAAACCTAGGTGAGGAAATGGATGTAGGCTGTAGTATTGGCTTCGAGCCTAGAAAAACCCTTGGAGGATATCTCTATGTGCACATAGGAGCATAG

>Csa012579

ATGAAAATACGAGAGACTTCCTCTTTTATTCTCAAGGGAGAAGTGGTTGGTAGGGATGATGACAAGAAAGCTATTATAGATTTTCTACTGGATACCAAAACCAAAAAGGATAATGTTGAAGTGGTTTCCATAGTTGGTATGAGAGGATTAGAAAATACTGCATTTGCTCAATCTATCTGTAAGGATGAGAAGATAAACAAACATTTTCAATTGAAATTACGGGTATGTATTTTGGAAGAATTTGATGTAAAAACAATTGTCGAAAAGATTATAGAGTCTATTGAAGAAAAGGAACCTAAGTCCCTTCAATTAGATAAATTACAAAGTATGCTTCGAGAAAAAATTAATGAAAAAAAGTACTTGTTGATCATGGATGATGTGTGGAACAAAAGCCATAAGAAATGA

>Csa017609

ATGGCTGATTTTATATGGACATTTGCACTGCAAGAGATTCTCAAGAAGACATTGCACCTTGCAACCCAACAAATCCGTCTGGCCTCCGGTTTCAACCACGACCTCTCTAAACTCCTCCACTCATTGCTCTTCTTCGAAGCCATTCTTCGCGATGTCGATCGAACAAAATCCGACCGACAGTCGGTCAAGATTTGGGTCACTAAGCTTCAGGATTTAGTGCTCGATGCTGAAGTTGTGCTGGACGAGCTCTCCTACGAGGACCTTAGGCGAGAAGTGGACGTCAATGGAAATTCGAAGAAAAGAGTACGCGATTTCTTTTCGTTCTCGAATCCCTTGATGTTTAGGTTGAAAATGGCGCGTAAAATTAGAACCATCACCCAAGTTTTGAATGAGATTAAAGGGGAGGCTAGTGCTGTTGGGGTTATTCCTAAAGGGGGCAATGATGAAATAGTGGCTGATAATGGCCATATTCCGGAGACTGACTCATTTCTTGATGAATTCGAAGTTGTAGGAAGAAGGGCTGATATATCTAGAATAGTGAACGTTGTTGTTGATAATGCCACTCATGAAAGGATCACTGTGATTCCTATTGTGGGAATGGGTGGTCTTGGAAAGACAACTTTGGCAAAAGCAGTTTTCAACCATGAGCTTGTGATAGCACATTTTGATGAAACTATTTGGGTGTGTGTGACTGCAACTTTTGATGAAAAGAAGATTTTAAGAGCAATTTTGGAATCTCTAACGAATTTTCCAAGTGGTTTGGATAGTAAGGATGCTATACTTAGAAGGCTACAAAAGGAGCTGGAAGGGAAAAGGTATTTTCTTGTGCTGGATGACGTGTGGAATGAAAATGTTAAACTGTGGAACAATTTCAAGAGTCTTCTGCTAAAGATTACAAATAGTATTGGGAACAGAGTTCTTGTGACAACTAGAAGTGAGGAAGCTGGAAAAATCATGGAAACATTTCCCAGTCATCATGTAGAAAAGTTATCGGATGATGAATGCTGGTCAATATTCAAGGAAAGAGCATCGGCAAATGGATTACCACTGACTCCAGAATTGGAAGTTATTAAGAATGTGCTTGCAGAGCAGTTTGGAGGCATTCCATTGGTTGCAAAAGTTCTGGGAGGGGCTGTACAATTTAAGAAAAGAACAGAGACTTGGTTGATGTCAACATTGGAAACCCTTATAATGAATCCACTTCAAAATGAAAATGACGTTTCATCTATTTTGAGATTAAGCGTGGATCATCTGCCAAACTCATCATTGAAACAATGCTTTGCCTACTTTTCTAATTTTCCCAAGGGTTTTAACTTTGAAAAGGAACAACTAATCCAATTTTGGATGGCAGAAGGGTTCATTCAACCTTCTGATAAAGTAAACCCCGAAACCATGGAAGATATAGGAGATAAATACTTCAATATCTTGCTGGCTCGTTCCTTATTTCAAGATATTGTTAAAGATGAGAATGGTAAAATTACACACTGTAAGATGCATCATCTTCTACATGATCTTGCTTATTCTGTCTCAAAATGTGAAGCACTGGGTTCGAATCTTAATGGTCTGGTTGATGATGTTCCTCAAATTCGACAATTATCCCTGATTGGCTGCGAGCAAAATGTAACGTTGCCTCCTAGAAGGAGCATGGAGAAGTTGCGTTCTCTATTTTTGGATAGAGATGTGTTTGGCCACAAGATTTTAGATTTCAAGCGCTTGCGTGTTCTGAACATGTCCCTATGTGAAATCCAAAACTTACCAACTTCAATCGGAAGGTTAAAGCATCTAAGGTATCTTGATGTCTCAAATAATATGATAAAGAAACTTCCAAAATCTATTGTTAAGCTTTATAAATTGCAGACCCTGAGGCTGGGTTGTTTCCGTGGAGAAGCCCCCAAAAAATTCATAAAATTGATCAGCTTGAGACATTTCTATATGAATGTTAAAAGACCAACAACTAGGCACATGCCTTCGTATTTAGGCAGGTTGGTTGATCTTCAATCCTTGCCTTTTTTTGTTGTTGGGACAAAGAAGGGTTTCCATATAGAAGAGCTTGGATACTTGAGGAATCTCAGAGGTAAATTAAAGCTTTACAATCTTGAATTAGTAAGAAATAAGGAGGAAGCCATGAGGGCAGATTTGGTGAAAAAGGATAAGGTGTACAAATTGAAACTGGTATGGAGTGAAAAAAGAGAAAATAATTATAACCATGACATTTCTGTTTTAGAAGGACTTCAACCACACATCAATCTTCAGTACTTGACAGTTGAAGCCTTTATGGGAGAACTTTTTCCAAATCTTACTTTTGTTGAAAATTTGGTACAAATTTCTCTAAAAAATTGTAGCAGATGTCGAAGAATTCCAACATTTGGACATCTACCCAATCTTAAGGTTCTTGAGATTTCTGGATTACACAACCTAAAATGTATAGGAACAGAGTTCTATGGGAATGAATATGGAGAAGGAAGTTTGTTTCCAAAATTGAAAAGATTTCATCTTTCAGACATGAATAATCTTGGACGTTGGGAAGAAGCAGCAGTGCCAACAGAAGTTGCAGTTTTTCCTTGTCTTGAAGAGTTGAAAATTCTCGACTGTCCTAGACTAGAAATTGCACCTGATTACTTCTCAACTCTTAGGACATTAGAAATTGATGATGTCAACAACCCAATTTCACAGATCACTCTTCAGACATTCAAACTACTTGGTATTATACACTCTGGCAACCTGAGTGGTTTGCCTGAGGAGTTACGTGGTAATCTGTCATCTCTTGAGGAGTTTAAGGTTTGGTATTATCTTCACTTGAAATCCTTTCCAACTATTCAGTGGCTCACTGATATTTTGAAAGGCAAGACCGGATATGACACAAAGTGGACAAATATTCAATCTCATGGGCTAGAATCGTACACTTCTGTGAATGAATTGTCCATTGTTGGGCACTCTGATCTCACATCAACCCCAGATATAAAAGCTTTATATAATCTTTCGTCTTTAACAATTAGTGGCTTGAAGAAATTGCCAAAAGGATTTCACTGCCTCACTTGCTTGAAAAGTTTGTCAATTGGTGGATTCATGGAGGGGTTTGATTTTAGGCCTCTTTTGCATCTCAAGTCTCTTGAAAATCTTGCAATGATAGACTTTGGCCTTGCAGAAAGCACACTTCCTGATGAGCTTCAACACCTAACTGGCTTAAAGCACTTGAAAATTGTTGGATTTCAGGGCATTGAATCTCTGCCAGAGTGGTTAGGAAATCTTAACTCATTGGAAAGTTTGCACATTGAGAGTTGCAGAAAATTGAGAGAGCTTCCAGAAGCCATGGGTTGCCTCGCCAAATTGGAGGAAGTGCGGAGTTTCAATTGCCCAGAGTTGAGGGTTTACCAAGACGAATCAGAATGGGCCAAGATTTCTTACATTCCAAGATTCATATCATTCAATTATTGGGTTGATGAGTAA

>Csa017644

ATGGCGGGTTCAACCGGCGCTAATTCTTCTTCCAATTCTTCTCCTGCTCCTCCTCCCCCCAAAATCCTCCTAGCAAAGCCTGGTCTCGTCCCCGGCGGACCTATTAACTCTAAGATTGGACGTGGCGCCGGCGCCGACGACGAACCAGCATCCATTCGCTCCCGTCTCCCTTCTCTTGGATCCCTTAATCTTCTCTCCGATTCATGGGATCTCCACATTGACCGTTTCCTCCCTTTTTTGACTGAGAATACGGAGTTCAAGGTGGTGGGGATAATTGGTCCACCGGGAGTTGGTAAGTCGACGATTATGAATGAGATTTATGGCTACGATGGAAGCTCTCCAGGTATGCTACCACCATTTCCAATACTATCTGAAGATGTTAGAGCAATGGCTAGGCATTGTACATTGGGTATTGAGCCTCGAATTTCTTCTGAGAGGATTATACTTCTGGATACTCAGCCTGTGTTCAGTCCTTCGGTTTTAGCTGAAATTATGAGACCCGATGGTTCGTCCACTGTTTCAGTTATTAATGGAGAATCTCCATCTGCTGAATTGGCTCATGAACTCATGAGTATCCAGCTGGGCATTCTTCTAGCGTCCATTTGCAACATTGTTCTTGTGATATCAGAGGGAGTTCATGATCTAAATATGTGGCATTTGATGTTAACTGTTGACTTGCTTAAACATGGCTTGCCTGACCCATCTTCTCCTATTTCTTCTCACGCACAAAATTCTAATGTAGCATCTGAGAAGGAATACAAAGAAAAAACTTCTACAAGTGAAGAATATATGGCAACTCCCATCTTTGTTCATGCCAAGTGA

>Csa018159

ATGGCTGATTTCCTATGGACTTTTGCTGTGGAAGAAACGTTGAAGAAGGTGTTGAAGGTTGCAGGGGAGCAAACTGGCCTAGCATGGGGCTTCCAGGAACATCTCTCCAACCTCCAAAAATGGCTACTCAAGGCTGAAGCTTTCTTACGCGATATCAACATGAGAAAATTACATCTTGATTCTGTGAGGATGTGGGTGGACGATCTTCAACATCTTGTTTATCAAGCCGATGATCTATTAGACGAAATTGTTTATGAAGATCTTCGACAAAAGGTCCAAACAAGAAAAATGAAGAAGGTGTGTGATTTCTTTTCTCCTTCTACCAATGTTTTGATCTTTCGTCTTAACATGGCAAAAAAAATGATGACTCTTATAGCATTGTTAGAAAAGCATTACCTTGAGGCTGCTCCTTTAGGACTAGTGGGAAATGAAAATGCAAGTCCTGAGTTCGATGTTATTGGTCAATATCGAGAGACAATTTCAGAACTCGAAGATCATAAGATTGTGGGGAGGGATGTTGAAGTTGAAAGTATAGTGAAACATGTGATTGATGCTAGCAATAATCAACTTACATCTATCCTACCCATTGTTGGTATGGGTGGATTGGGAAAAACAACTTTGGCAAAGTTAGTTTTCAACCATGAGTTGGTTAGACAACATTTTGATAAAACTGTTTGGGTTTGTGTCTCTGAACCATTTATTGTCAACAAGATTTTGCTAGATATTTTACAAAATCTAAAAGGCACCATTTCTAATGGAGGGGATAGTAAGGAAGTTTTACTTCGTGAACTCCAAAAGAAGATGCATGGCCAAAGATATTTTCTTGTGCTTGACGATGTTTGGAACGAAAATTCTTTTCTATGGGATGAGTTGAAATACTGTTTGCTCAAGATCACTGGAAACTCTAAAAATAGTATTGTTGTGACTACAAGGAGTGCTGAAGTTGCAAAAATCATGGGAACATGTTCTGGTCATCTTTTAAGTAAATTATCTGATGATCATTGTTGGTCCTTGTTTAAAGAAAGTGCAAATGCATATGGATTATCAATGACTTCAAACTTGGAGATCATTCAAAAAGAGTTAGTCAAAAAAATTGGTGGTATACCATTGGCTGCACGAGTTTTGGGAAGGGCAGTAAAATTTGAAGGAGATGTTGAGAGATGGGAGGAAATGTTGAAAAATGTGTTAAGCACTCCACTCAAAGAGGAAAATTTTATTTTGTCTATATTAAAATTAAGTGTGGATCGTCTACCGTCATCTGCATTAAAGCAATGTTTTTCATATTGTTCAATTTTTCCCAAGGATTTTGTGTTTGAAAAACAAGAACTAATTCACATGTGGATGGCACAAGGTTTTCTTCAACCACAAGAAGGAAGGAACATGACAATGGAAACTGTAGGAGACATATACTTCAAGATCTTGTTGTCACACTGCTTATTTGAAGATGCCCATGAAACAAAGACAGAGGAATATGAGATACCTGATCTGCTTGAATTTGAAACAAGGCCAGAAGAATATAAGATGCATGATCTTGTACATGATATTGCGATAGAAATTTCAAGAGATCAAAATTTGCAACTAAATCCTAGCAATATATCAAAGAAGGAACTTCAAAAGGAGATTAAAAAGGTTGCATGCAAGTTACCCATGGTTGATTTCATTCGACGGATTCCTTGCAATATAGGCCAACTAACATTTTTTGATGTTGAGATAAGGAACTTTGTTTGTTTGCGAGTTTTAAAGCTATCAACGCTGCCTAGTGATAAGTTACCGAAGTCAATTGGTCAATTGAAACACTTGAGATATCTAGAAATTGCATGTTATTTAGTCGAGGATGAGTTGAGCAACAATAGTGTAACACAAATTTCTGAACAACTTCAACACCTCACTGCCTTGGAATTTCTGTCCATTGAAAATTTTGGAGGCATTGAAGCTTTGCCAGAATGGTTAGGAAACTTTGTATGTTTGCAAACACTCAGTCTTTATAACTGCAAAAATTTGAAAAAACTGCCTTCTACAAAAGCAATGCTACGTCTCACTAAATTAAATCAATTGTATGCTTGCAAATGTCCGATGCTACTACTCGAAGAAGGTGATCCAGAGCGAGCAAAACTTTCCCACTTTCCAAACATGTTGGTTCAGCGCAACGGTTATCAGAAGTGTATTTAG

>Csa018210

ATGGACATTTTAGTTTCAGTCATTGCAGCAACAATTAAACCTATTGGACATCAATTAGGTTACCTTGTTTGCTATAACAGAAACAAGAAGGAGCTTAGAGAACAACTTGAAAATCTTGAGACTACTAAAAAGGATGTGAATCAAAGGGTTGAAGAGGCAAAAGGCAAATCGTATACAATCTCTGAGGAAGTTTCAAAGTGGTTGGCCGATGTGGATAATGCAATAACCCATGATGAGCTATCCAACTCCAACCCATCTTGCTTTAACTTGGCTCAACGATACCAGCTAAGTAGAAAAAGGGAGAAGCAAGTGAATTATATTCTTCAACTCATGAACAAAAGAAACAGCTTTGTCGAAGTTGGATATCGTGCACCTCTTCCGGATACTGAGAATACTGTTGTTCCCGGAGATTACCAAGTTTTGGAATCAAAAACATTATTGGCTAAAGATATCAAGAATGCGCTTTCGAAACCTGAGGTCAATAAGATTGGTGTTTATGGTATGGCAGGTGTTGGAAAAACTTATTTTCTTAACGAAGTTAAGAAATTGGTGTTGAAAGGGGAAGACAGATTGTTTGATCGAGTTATTGATGTGCGTGTAGGTCGATTTAATGATGTAACAGACATACAAGAACAAATTGGTGATCAATTGAACGTAGAATTGCCAAAAAGTAAAGAGGGAAGAGCGTCTTTTCTACGGAATAATTTGGCGAAAATGGAGGGTAATATCCTCATTTTATTAGATGATTTGTGGAAGGAATATGATCTTTTAAAAGAGATTGGGATTCCATTAAGTAAAGATGGATGTAAGGTACTCATTACAAGTCGATCACAAGATATATTAACCAATAATATGAATACACAGGAGTGTTTTCAGGTGAGTTCGTTATCTGAAGAAGAGTCTTGGAAGTTTTTTATGGCAATCATTGGTGATAAGTTTGATACAATTTATAAGAAAAACATTGCAAAGAATGTTGCAAAAGAATGTGGAGGGTTACCGCTTGCACTTGATACCATTGCAAAAGCATTGAAGGGGAAAGATATGCACCATTGGGAGGATGCTTTAACCAAATTGAGAAATTCTATTGGAATGGATATTAAAGGGGTAGCTCTAAAGAGTTTTGGACGAGGTATTGGAGTTGATATTTGTCATCAGCTCCTATAG

>Csa019939

ATGGCAGAATCCATTCTGTGCAGCCTTGCAGGAAGCATTATTACCAAATTGGGTTCTTTCGCACTTCAAGACCTTGGATTGTTGTGGGGTTTCCATGATGAACTTGACAAACTGAAAGGCACTGTTTCCGCCCTCGAAGCCGTACTTCTCGACGCAGAAGAGAAGCAGTCCAAAAGTCGTGCAGTGAAGGACTGGATTTTAAAGCTTAAAGATACTTTCTACGACATCGACGATTTGTTGGACGTGTTCTCCTATGAATCTTTGAAAAGACAAGTTATGACCAAACATAGAACTAATAACACCAAAAAAGTACGCATTTTCTTCTCAAAATCTAATCAAATTGCATTTCGTTTGAAAATGAGTCAAAAAATCAAAAGGGTCCGAGAGAAATTAGATGCAATTGCTATGGATAAAACTCAATTCAATCTTTATGAGAATACTAGGGAAATACAAGATGATGAATCGACAAAACGACTGGAGACTACCTCTTTCATACGTGAAGGAGAAATAATTGGTCGGGATGATGACAAAAAAAGTATTATACATTATCTATTGGATACCAACATCCACGAAGATAGTGTTGCAGTGATTGCTATTATTGGAATGGGAGGATTAGGAAAGACTGCTCTTGTTCAATCTATTTATGGTGACGAGAAGGTAAAGAAACATTTTGAGTTGACAATGTGGGTGTGTATTTCTGAAGAATTTGATGTCAAAGTAATTATTGAAAAAATTATAGAGTCTCTCACAAAAAAGAAACGTGAGCCTGACCTTCAGCTTGATACGTTGCAAAGCATGGTCCGAGAGAAAATTGATGGAAAAAGATACTTGCTTGTCATGGATGATGTGTGGAATGTTAATCGTGCAAAATGGATAAGTCTAAAAAGGTATCTCATGGGTGGAGCTAAGGGAAGTAGGATTTTGATCACAACCCGTACTCATCAACTTTTCAACTGTGAAAAATTGCAACAGCTTCCTCGATTTGATCGATTTCCTTTTCTCAAACATCTTCATCTGGAAGATTTACCGAGTATCGAGTATATTGCTATTAACAATTATGTTTCTTCATCAATGACTACTTTCTTTCCATCCCTTGAGAATCTAAGCATCATTAAGTTGCCTAACTTGAAAGAATGGTGGAAGGGGGAAAGCATTGATCAAAATACTTCATTTCCAACAATTTTACGTCATCTTTCTCAACTAAAGATTCATTATTGTAGACAATTGGCTTCTATTCCACAACATGGACCTTTGCAATCATTGGACATACGTGATATCAGTTTGCAACTTTTTGAGTTGGTAATCAAAATGACCGCTACGAACATTATTGTTGGGGAGGATTCTTCTTCTTCAGCGAATGATATGTTTATTAGATCCTCATCTTCTCTAAAGATCTGGAAGATTGATTGGGAGTTTCTACCAAATGACTTATTCTCCAATGTGACACATCTCCAGTCTCTTGTCATAGGACGTTGCTTCAATTTAAAAATGTCTTTTGATGATGATAATGTAAGATGGAAAGAACTCGGAAGTCTTCGCACACTTCGACTTTGTTTCATCCCCAAATTAGAGTATTTGCCTAAGGGTTTCCAATATTTGAAAGCACTCGAACATTTGGAACTACTTTGGTGTGAAAATTTGGCGTGTATTTTAGGGATTGAGCATCTCACTTCACTATCACGATTGGAAATTTCAAATTGTCCTAATTTAACTTCTTTGCCGGAAGGGATGACTCAACTTATTTCATTAACATGTTTGATAATCGATGATTGTCCCAATTTAAGTACCTTGCCAGAAGGGCTTCATCACCTCCTTAATACCCCGAGCCTTTCAGCAAATATTGCAACCAAATTGGGTTCTTTCTCGCTCCTAGAGCTTGGATTGTTGTGGATCGGTTTCCATGAAGAGCTTGACAAACTGAAAGACACTCTTTTCGCCATCCAAGTAGTACTTCTCGACGCAGAACATAAGCAGTACAAGAGTTATGCGGTGAAGGAATAG

>Csa020328

ATGGCTGATTTCCTATGGACTTTTGCTGTGGAAGAAATGTTGAAGAAGGTGTTGAAGGTTGCAGGGGAGCAAACTGGCCTAGCATGGGGCTTCCAGGAACATCTCTCCAACCTCCAAAAATGGCTACTCAACGCTCAAGCTTTCTTACGCGATATCAACACCAGAAAACTACATCTTCATTCTGTGAGCATCTGGGTGGACCATCTTCAGTTTCTTGTTTATCAAGCCGAGGATCTATTAGACGAAATTGTTTATGAACATCTTCGACAAAAGGTCCAAACAACAGAAATGAAGGTGTGTGATTTCTTCTCTCTTTCTACCGATAATGTTTTGATCTTTCGTCTTGACATGGCAAAAAAAATGATGACCCTTGTACAACTGTTAGAAAAGCATTACAATGAGGCTGCTCCTTTAGGACTAGTTGGGATTGAAACTGTAAGACCCGAGATCGATGTTATTAGTCAATATCGAGAGACAATTTCAGAACTCGAAGATCATAAGATTGTGGGGAGGGATGTTGAAGTTGAAAGTATAGTGAAACAAGTGATTGATGCTAGCAATAATCAACGTACATCTATCCTGCCCATTGTTGGTATGGGTGGATTAGGAAAAACAACTTTGGCAAAGTTAGTTTTCAACCATGAGTTGGTTAGACAACATTTTGATAAAACTGTATGGGTTTGTGTGTCTGAACCATTTATTGTCAACAAGATTTTGCTTGATATTTTAAAAAATGTAAAAGGTGCCTATATTTCTGATGGAAGGGATAGCAAGGAGGTTTTACTTCGTGAACTCCAAAAAGAGATGCTTGGGCAAAGGTATTTTCTTGTGCTTGACGATGTTTGGAACGAAACTTTTTTTCTATGGGATGACTTGAAATATTGTTTGCTCAAGATCACTGGAAACTCTAACAATAGTATCCTTGTGACTACAAGGAGTGCTGAAGTTGCAAAAATCATGGGAACATGTTCTGGTCATCTTTTAAGTAAATTATCTGATGATCATTGTTGGTCCTTGTTTAAAGAAAGTGCAAATGCATATGGACTATCAATGACTTCAAACTTGGGGATCATTCAAAAAGAGTTGGTCAAAAAAATTGGTGGCGTACCATTGGCTGCACGAGTTTTGGGTAGGGCAGTAAAATTTGAAGGAGATGTTGAGAGATGGGAGGAAATGTTGAAAAATGTGCTAACAACTCCACTGCAAGAGGAAAATTTTGTTTTATCTATATTAAAATTAAGTGTGGATCGTTTACCATCATCTTCAGTAAAGCAGTGTTTTGCATATTGTTCAATTTTTCCCAAAGACTTTGTGTTTGAAAAACAAGAATTGATTCAAATGTGGATGGCCCAAGGTTTTCTTCAACCACAACAAGGAAGATACAATAACACAACAATGGAAAATGTAGGAGATATATACTTCAACATCTTGTTGTCACGTTGCTTATTTGAATTCGAAGATGCCAATAAAACAAGGATAAGAGATATGATAGGTGATTATGAAACAAGAGAAGAATATAAGATGCATGATCTTGTACATGATATTGCAATGGAAACTTCAAGGTCGTATAAAGATTTGCATCTAAATCCTAGCAATATATCGAAGAAGGAACTTCAAAAGGAGATGATAAATGTTGCAGGCAAGTTACGCACAATTGATTTCATTCAAAAGATTCCTCACAATATAGATCAAACACTTTTTGACGTTGAGATAAGAAACTTTGTTTGTTTGCGTGTTTTGAAGATATCGGGTGATAAATTACCAAAGTCAATTGGTCAATTGAAACACTTGAGATATCTAGAAATTTTAAGTTATTCAATAGAATTAAAATTACCAGAGTCTATTGTTTCACTTCATAATTTGCAAACGCTAAAGTTCGTATACTCAGTGATTGAAGAATTTTCAATGAACTTTACAAATTTGGTAAGTTTAAGGCACTTGGAATTAGGGGCAAATGCTGACAAAACACCTCCACATTTAAGTCAATTGACTCAACTTCAAACATTGTCTCATTTTGTAATCGGGTTTGAAGAAGGTTTTAAGATTACTGAATTGGGTCCATTGAAAAACTTGAAAAGATGTCTGTGTGTTTTGTGTTTGGAGAAAGTTGAAAGTAAAGAGGAAGCAAAGGGAGCAGATTTGGCAGGAAAGGAGAATTTAATGGCGCTACACTTAGGGTGGTCCATGAATAGAAAAGATAATGATTTGGAAGTGTTGGAAGGACTTCAACCAAACATAAATCTCCAATCATTGAGAATCACCAACTTTGCTGGAAGACATTTGCCTAACAATATTTTTGTTGAGAATTTAAGAGAGATACATTTGTCTCATTGTAATAGTTGTGAAAAGCTTCCAATGCTTGGACAACTAAACAACCTAAAGGAACTTCAGATTTGCAGCTTTGAAGGCCTCCAAGTTATAGACAACGAGTTCTACGGCAATGATCCAAACCAAAGAAGGTTCTATGAATCATCAAATGTCACAATTTTCCCCAATCTCAAGTGCTTGAAAATATGGGGATGTCCCAAATTATTAAACATTCCAAAAGCTTTTGATGAGAATAATATGCAACACCTTGAATCATTGATCCTTTCATGTTGTAACAAATTGACAAAACTCCCAGATGGACTACAATTTTGCAGTTCTATTGAAGGGTTGACAATAGACAAATGTTCAAATTTGAGCATAAATATGAGAAATAAGCCGAAATTATGGTATTTAATCATTGGTTGGTTAGACAAGCTGCCGGAAGATTTATGTCATCTCATGAATTTGAGGGTAATGAGAATTATTGGAATTATGCAGAATTATGATTTTGGCATCCTTCAGCACCTTCCTTCCCTTAAACAACTTGTTTTGGAAGAGGATTTGTTGAGCAATAATAGTGTAACGCAAATACCTGAACAACTTCAACACCTCACTGCCTTACAATTTCTGTCTATTCAACATTTTAGACGCATTGAAGCTTTGCCAGAATGGTTAGGAAACTATGTATGTTTGCAAACACTCAATCTTTGGAATTGCAAAAAATTGAAAAAACTGCCTTCTACAGAAGCAATGCTACGTCTCACCAAATTAAATAAATTGCATGTTTGTGATTGTCCGCAACTACTACTTGAGGAAGGCGACATGGAGCGGGCGAAACTTTCCCACCTTCCAGAAATTCAGATCAATCGTTGGTTTATACATTTATTATGAPROTEIN

Protein sequences of the NBS-encoding genes from the 9930 cucumber (Cucumis sativus L.).

>CSA001236

MAEFLWTFAVQEILKKVLTLVAEQIILAREVKDVLQQLQKELVESQKIVSAITTQRQNHYSPDSLVTQWVNDLQLIVHEADDLLDLFVYEHLQQRVNPSAHGKIIKKVPHFLCASARTKKMKEIIALLNKHCTKLPHLLQLEPTPSNIAETEVAQIQETVSKPEDYVVGRNREVETIVDRVIDASKQELNSILPVFGMGGLGKTTLAKSVFNHDRIKNHFGITIWIYVSQPFVINNILQAILQKVEVHSSDCSNNREALLEKLTENMGEKTYFLVLDDVWNENKMLWEKLKECLMSITHMSGNSILVTTRSSGIAKMMEENIGSHELRKLSDDQCWSIFRNFANAKDVPMTSNLEFVQKEFDKRIGGLPLIAKVLGAAVPFSGDHDQWVANIKSVLTTPIKEEEFVKFTLKLSVDRLPNASVKQCFAYCSNFSKGCEFDKKQVIRMWMAQGFTQPDERNNETMEDTGERYFNILLSFCLFQDVVKNERGIIEKVRMHDLIHDIACQVSNDKKLRIDHIISSNWKDWTKDDKILVSKLRTINFYDRHHVVVQDKIGDFTGLRVLTIENYIVEELPNSIFKLKHLRYLDISYCYSIKKLPESIVLLYNLQTLRFHLLSKGFLPKNVGQMISLRHLEFSSIDKQMSPYLSQLIQLETLPKFAVGFEKGCKITELGVLRNLKGLLKLQRLEHVESKEEAETAKLVEKENLEEVHFVWTKERKRKVENKNDLEVLEGLQPPKNVEYLRIKYFLGGCLPNQTFVENLVKIELRDCGNCEKLPRLGQLGNLEILDISWFERVKSIGNEFYGNSSNNQRSLFPRLKELYVDEMRRIGEWEEVGSNVKAFPRLERLYIGCCRDLVKIPDVFGYCDEYGEKHLEVVEIIEHLWLDRPSNLWSFVTTQGGALANLLSRRTRSFFYYRKERRQMKEKIKEKLRFALYVQSAATQFIVKVPTPTPTPKPKIRSFSPCFF

>CSA001480

MAESILFSLAANIATKLGSLALQDLGLLWTGIHEEIDKLRDTLSAIQAVLHDAEQKQYKSSAVKEWVSRLKDAFYDMDDLMDEFSYESFQRQVMTKHRTNNCTKQVCIFFSKSNQIRFRLKMVHKIKKIREKLDTIDKDKTQFNLFDNTREIRNDEMTQRSETCSFILEGEVIGRDDDKKCIVHFLLDTNIIAKENIVVVAIIGMGGLGKTALAQSIYGHMKENKHFELTMWVCISEEFDVKVIVEKIIESLTKKRPEPNLTLDTLQSMLREKIDGKKYLLVMDDVWNDERTKWINLKKFLMGGAKGSRILITTRTHQVAHIFDTDLFHDLSELDKDKSEMLENSKLVGIGKEIVAKLKGSPLAIRVIGSYLYSKKSEKDWLSFKENELDTIMQQKNEIQSILKISFKKVFLKVAPMKGVMRFEKKGKLSPRFVGPFEILERVGVVAYRLALPPPLSAVHNVFHVSMLRKYVVDTSHVVDYEPLEIDEHLSYVEQPVEILAREVKMLRNRSIPLVKVLWRNHRIEEATWEREEEMRTRYPKLFQD

>CSA001907

MAEAILFQVAGEILMKLSSQAFQRLGMLFGLKGDLNKLTTTVSTIKDVLLDAEGRQTKSHLLQNWLHKLEEALYDAEDVLDELSTEALRRELMTRDHKNAKQVRIFFSKSNQIAFNYRMARQIKNIWERLDAIDAEKTQFHLRENCESRTQYGSFDRIMMGRETWSSSNDEEVIGRDDDIKEVKERLLDMNMNVTHNVSFIAIAGMGGIGKTTLAKSLYNDEEVSGFFDLKIWVWVSDQFEVQVVAEKMIESATKNNPSVKGMEALQAKLQKVIGERKYLLVMDDVWNESEEKWHGLKSLLMGGARGSKVLITKRDRKVATEIKSMTSLFTLEGLSESNSWLLFSKVAFKEGKESTDPSTIHLGKEILVRCGGVPLVIRHVGRMLYSKTSQEEWMSFKDNELLEVIQQDNDMTSILKLSYNHLPPNLKRCFAYSSLFPKGYKIEIKDLIRQWVAQGFIEVSNGRKSLEDTGKDYFNELCWRFFYANSSDECNINDIVCMHDVMCEFVRKVAGNKLYVRGNPNNDYVVSEQTLHISFDYGIQSWQDVLSKLCKAKGLRTILLLFRPYEKMNKIDKAILDELFSSFPRLRVLDLHFSQISVVPKSIKKLRHLRYLDLSENDMELIPHSIIELQNLQTLNLTECYELKELPRDIDNLVNLRHLTFEPCMEVTPTSEGMEKLTCLQTISLFVFDCKKTNKLWELNDLSYLTGELKIIGLEKLRSSPSEITLINLKDKKGWQGLNLEWKLGKDEYEGEADETIMEGLEPHPNVESLSINGYTGGALPNWVFNSLMKLTEIEIENCPRVQHLPQFNQLQDLRALHLVGLRSLEFIDKSDPYSSSVFFPSLKFLRLEDMPNLEGWWELGESKVVARETSGKAKWLPPTFPQVNFLRIYGCPKLSSMPKLASIGADVILHDIGVQMVSTIGPVSSFMFLSMHGMTNLKYLWEEFQQDLVSSSTSTMSSPISLRYLTISGCPYLMSLPEWIGVLTSLETLHIKECPKLKSLPEGMQQLKSLKELHIEDCPELEDRCKQGGEDWPNISHVPNFTYKNASDIDTPQSSSGFSHHPFSIVRISVI

>CSA002354

MANEFQAQHGDWTYDVFLSFRGEDTRKNFTDHLYYALKDAGINVFRDDPELQRGEDISSGLERAIEGSKVAVIVFSERYAESGWCLEELVKIMECRRTLRQMVLPVFYNVDPSCVRKQKGEFEEAFVKHEKGKDIDKVRRWRMALTEAANVAGLGLTQNANGYEAEFIRSIVKMISKEVKSNYLFIALYPVGIESRIKLVLPHLHIGSNDDVKFVGILGIGGLGKTTIAKALYNQLHHNFEAACFLANIKQTPNQPNGLVHLQKQLLSSITNSSNINFENMDRGIVVLQESLRRKKLLLILDDVDKISQLTALATRRECFGSGSRIVITTRHRRLLNQIEVDGICSIDVMDDAEALQLFSWHAFHNSYPSETFHQLSKRVVNYCGGLPLALQVLGCFLFGRSREEWQDTLKNLKKILDDQIQIKPKITFDTHNDHTCKDIYLVNQMLDGWGSFPRIGDINRLVTSDLLRDHTQLFLPKEVRLSVLGPKVSGDQRTMPQTEYLGISFPEDSHSQTQLLLGAPHDKRHSNNSDTQKAGLIELTL

>CSA002355

MADELRPQHGNWTYDVFLSFRGEDTRKNFTDHLYYAFKDAGINVFRDDPELERGEDISSELERAIEGSKVAVVVFSERYAESGWCLEELVKIMECRRTLRQLVFPIFYNVDPSCVRKQKGEFEEAFVKHEVRYFRDIDRVLKWRMALTEAANLSGWDLRNIANGHEAKFIRLIVEKVSKEVNSKYLFIALYPVGIESRLKLLLSHLHIGSNDVRFVGILGMGGLGKTTVAKALYNQLYHNFEAKCFLSNIKAETSNLIHLQKQLLSSITNSTNINLGNIDQGIAVLQERLRCKRLLLILDDVDDLSQLTALATTRDLFASGSRIIITTRDRHLLNQLEVDEICSIDEMDDDEALELFSWHAFRNSYPSETFHQLSKQVITYCGGLPLALEVLGSFLFGRSREEWEDTLKKLKKIPNDQIQKKLKISFDGLNDHTYKDIFLDVSCFFIGMERNYVEQILDGCGFFPRIGISVLLQRCLLTIGDKNRLMMHDLLRDMGREIVRENFPKYPERHSRLFLHEEVLSVLTRQKGTDATEGLSLKLPRFSKQKLSTKAFNEMQKLRLLQLNFVDVNGDFKHISEEIRWVCWHGFPLKFLPKEFHMDKLVAMDLRYSQIRFFWKESKFLKNLKFLNLGHSHYLTHTPNFSKLPNLEILSLKDCKNLIELHPTIGELKALISLNLKDCKSLNSLPNSFSNLKSLQTLIISGCSKLNSLPEDLGEITSLITLIADNTPIQKIPNTIINLKNLKYLSLCGCKGSPSKSSFSSMIWSWISPKKLSQNYTSILLPSSLQGLNSLRKLCLKNCNLSNNTIPKDIGSLSSLRELDLSENLFHSLPSTISGLLKLETLLLDNCPELQFIPNLPPHLSSLYASNCTSLERTSDLSNVKKMGSLSMSNCPKLMEIPGLDKLLDSIRVIHMEGCSNMSNSFKDTILQGWTVSGFGGVCLPGKEVPDWFAYKDEVSTDLPSLSVINYTKSSITTNKPLTNDVIMSTQDHLWQGHLSNKAFKMEPGDEVEIIVDFGAEITVKKIGISLVFDKYVDQTMLEFASTSNDDDVVVDNQDENVSEKDGEVGSKRGFDENDDEGLKNSYQIPKRLKCEIDSNMKIDEE

>CSA002384

MAEIGTFVVQEVLKRIVKYGAEQIVVAWELENEVSLLKDKLHDADTILEDINRKKSHPGNSVKRWVEKLEDIVHEADDLLDELVYEHLRRTVEHTEKFSKVSDSISSSINSFLFRRKMAKKIKNITDTLNQHYCAASAFGLVGVETVTEIELALNQIRETTSILDFQVEGREAEVLELLKLAIDSTNEHHMSVISIVGMGGLGKTTLAKMIFNHREIEGHFDKTIWGLTKTCSGLESNKEALLGRLRKEMQDKNYFLVLDDVWDNEKHLWDELRGCLKHIAGKPGNTIMVTTRNEEVATMVEPISIYRLKKLSNDQCWALFKESANANQLPMNSKLEIMKKELVRKMGGVPLVAKVLGGAVKFEETELEEEDHEISWMTKVESIVRNISLEDKDFVLSILKLSVDSLPNPVLKQCVAYCSNFSQDYDFQKDDLIKMWIAQGFIQPGQGRDKNLLMEDIGEQYFNFLLSRSIFQDVTRDANKRIVGFKMHDLMHDIACAISSHQNVESNPNNLSGKSVRKLRTLICNDEVINYLNQNDIVCLRVLKVIFQSHTDLWIPIDKLIHLRYLDISECSINKLLLESLSLLYNLQTLKLGQSGLPKNLRKLVNLRHLEFKMFGDTAMPSDMGNLIHLQSLSGFLVGFEKGCKIEELGPLKNLKGKLTLTNLWRVQNKDEAMAAKLVEKKNLRHLNLWFFETDKRGEDDEDGIVQVLEGLQPHKNLQSLEILGFRGKVLPTGIFVENLVKIRLGHFERCEVLPMLGQLPNLKELEIMYMESVRSIGNEFYGVDSSHQNSVAFPQLKKLSIYEMMNLEQWDEATVVLESNLFGCLKEVRIRRCNPLAKLPSGLEGCHSLEYLSIRGCFNLMLNVQNLHKLYHLEIDGLKRLPKGMDGLTRLKELKIGGCMQNYEFSSVIHLASQLVELELSGRYGSVDTQLPQQLQHLTNLQVLKITQFDCIEALPEWIGNLISLKTLKCSYCFKLKELPSREAILRLTKLENLDIFECPKLLVGEGDQERAKLSHLPSKCVHKSE

>CSA002436

MAHCIYTQAENILTKLKDSPPYQKRIEYAMSSLKAVLLDAEEKQEQNQRLQNWLKELQNVFYQVEDSIDEFKWEIFKQKDIGKQVLAPFSCSNQISANKLKQKCKRKQVCDELNNIATRMYEFHLKVKHIDSISMETTHTFPSASEISIRHLKPSWQLLYPLIDASRTYDEIYDGILNVFNECTHVFHIVGEAGIGKSTVARFLYNHNNVVGKYTSRYWVCVEEGFNKHRLVKEVYSHADNKEICEDLTTEQLLSKVKRLLKVETFLLVFQDLSITNLKDRSPLVLNELLEMGQHGSKIIVTTQTEEIANYLQDRGYKTERRSKENLANGDRVSAHNQSLTKNTQNQAVPGSQIEETADAIANKIYEHSLSLTEDAQNLEVPIPQVTEDTNIGLGINPDIPPIKQDDTEYQTIFKLEKLSKQSSSRLFKEYAFRNRQEVENPELTKIVDQLLEKCMGVPLAIKCLGSLLSSETSIAKWKNIEEKLSLQEKKENGILHVLRVCYDQMPSQLKPCFLHCSQLPNDRIISSNDMIQLWMANGLLHSPEEKNSTMENIGEKYFMELWSRYFIQEIEEHGLGYWIKLHPLIQKLAHKITQEQSEGSGGNNHPKEVTEIRSIAFQERNMVLPNASLTEKCIWKYKGLRLLYLSNADLQEIPNSIGTLKYLRYLDLHGNTKIKHLPNSICNLQSLQTLILGSCSALEDLPKDIRNLISLRYLWVTTNKLRLDKNGVGTMNSLRFLAIGGCNNLENLFERPDCLAGLETLMIYNCTTLKLLPDEMRYLKSLQNLMIWSCKQLTLNLKEVEFKLQRFTIKELPRVERLPQWLENSAETLRTLQIINCPIRIMERQGIEKYEAVENTIIYGAVRFEMAPPGYDFEHRNLAVRNGNEEMHIYP

>CSA002437

MAYCIYYRAENILSELKNLPNYPRRIEYTMLSLKSILMDAEEKQEQSRGLQNWLEELQNVFSQIEGFIDEHKEEAYEGIGKQVLAPFSCSSNQIARTWKMEKLFDHLNEVAAKMYEFNLTERHTGAIKTETTNSFLTATEVSTRLMKPSWKVLYPLTNAPKFYQDERYRKILNDFKNPTLGFFHIVGEAGIGKSTLAKFIYNDPEVEGMFPSRLWVCVKEEFDTQRLMKEILNFSYSPATCDNLTTKLCPTDQYLRERTFLLVFQDLSIKNLDNCSLFTSLLMMGKPGSKIIVTTQNEEIANAIELTMIYKVGQQSEQNRSQTALDTVTKETANVNNADQFVQANPLGKIDQSIPSQTIFKVKRLSEKDSLSLFKDYASTYEGNEKDIMKTLKKCNGIPLAIKCLGSMLSLGPPATKWMEDNERQKGDNESSSTFSILKLCYNEMPSHLKRCFLYCSQLPNDSILSSNDVIQLWMANGLLRSRQENYLSLEDIGEIYFKELCSRCFLQDVEEYGLGYWFKMHPLIRELARLVQKRTKDLISIKPVTNVTSIAFPVRDEVPSSSFLAEKCISKFQHLRLLYLGHTDLQEIPNTIETLNHLTYLDLQGNKNIKRLPNAICNLQHLQTLILASCSALEELPKDICKLSNLRYLWVTSNKLRLHKNGVGTMTSLRFLAIGGCDKLQDLFERPSCLVRLETLMIYDCNSLQLLPNEMGSLISLQNLVIWSCKQLTLKGLEKVDFSLQRFTIRELPEVNKLPEWLQRSTETLRVLEIIDCPIKVEEEGIKMYKAVESKIIQGAVDITGNLVRRSPMVTKKVQMTGNFY

>CSA002438

MACCIYEQAENILIELKKFPMYLRRMQYTMLSLKTILKDAEKEEYRHCLNDWLQKLQSVFLQIEELLYESNREVKKQEATGKWVFLPSFNFSQIDQTKKMMKLCDDLDEIASHMYGFNLTNMETTHSFLSATEVSTRLMKPSWQLLYSLTNAPKVFQDKRYHNFLDHFKKSTHGLFHIVGEPGIGKTTLAKFFYNNLVNTFPSRLWICVKEEFDPQRLIKEMLSFSHCQVTCDNLTEKQLCFAVQQFLRDKKFLIVFQDISIKNLGNCSIFKSLLGMGNRGSKIIVTTQNEKIADAVGLKKLYKNESQVVPSPEATKPSDVNKDNMKHQTIFKVERLSKENSLSLFKVHAFTETQEAQIPNLTKIQEVIEQKCHGVPLAIKCLGGLLSKTSIAEWNGVIDKLWEHEEEEDGNKSILPTLRLCYDQMPSHLQRCFLYCSQLKKDRILSSNDVIQLWIASDLLPKENYLSLEKIGENYFKELCSRCFLQELEEYGFGYWFKLHPLIEKLARLLTQKQVFEVTKTQSIAFTIRDKVPPSAFLANACIDKFKYLRLLHLGNANLQGIPSAVENLVQLRYLDLQGNKKIKRLPNSIFKLKNLQTLILASCSALKELPNDIRQLTNLRYLWVTANNLRLHKNGVGTMTSLRFLAIGGCQNLQDLFKKPSCLVRLETLMIYDCKTLKSLPNEIGSLISLKNLVIWSCKKLTLTLKGVEFRLQRFTIRELPIVKKLPEWTQRFTETLRVLEIIDCPIEWNDDVLKSYKSLERFSIHGAVRTKNQIGGYNIDYRNFVRSRKVKKEVKTCVYY

>CSA002698

MVGLLDSVAGNLLGRIIEAADRLEFRAIQSELKNLETDVLNLKARLRDAEEKQASNCELNELLKNLKNVFSRADIAIEELECDYLKWRVQNRKNDVDDKGCQFSSCFSSNFLISPFNTGSKFQEDLKIITSELRSIEKAMSKFSLVEDEDEYIKKLKGEMTLRTSITGSHAFARLLRLRREAILSNVDSIFGRDKIQESIIKELVNDEQKSPRILSIQGDGGMGKTALAKLVYNADEVFDHFDKRMWVCVSEDFDIRRILREVLMSATGENVTTVALTESRLRIRLQRYFFGKKILLVLDDFGNLDPERVSELKKIVKMGVGGSKIMITTRSDETLNVATTHKIDKLDETISMQIFEDTYGSEGLSEGLRDDLYLKNLVAECGGAPLAIKCLAGLLSSKPSDGAKSPNVKDLSEKWKQEEANNGGGVLCALRLSYDLMPSYLKPCFLCFSVLPKDNVFFSFELIQLWMAQGILPSGTKDNPEEVGEKYFKEFRDRRLLVDVEEHTLGYWFKIHSLVHDLAVQKATEQKNLGNFHMLSFVDCDSIPSSTNYDNTRFISIPVVGGAGPNINSDLFKCITQFRQLRFLYLCNSSLEEIPTSIDTLKHLRCLDLRGSQRLKRLPESICKLQSLQTLVLAFCSELEELPRNIKNLISLRFLWIQTKQARLEKDEIGSLTSLRFLAIGRSENLTHLFEDINKLNSLKTLIIYECKSLLTLPKGLENMKSICNMGIWECDRLRFTFSLASLHLKKLILRELTAVSTLPNWLSNLDGTLEVLEIGEFPTLRKLPIWLLNFWELRILGISNCPKLKHDSFPPELNYFCDKIEELRITFCGSLSKSLLKKSMKEIEPESRVIFYIHTIYVDSKRMTPPVESTDEPKEAETKQDDAYNNASPPGTEQPSKTKHDDANNNMSHPGIGLLSESKQEHTNNNINEIETVKVCLGDNDHAEAHQAMVTTYEGF

>CSA002745

MASTEISTIPQMIESLSELHKNLSTALGNHTAGDQIEEEHQTPKFEKLLNSIDHLKEALETTRELDKKLNDPIQSINTCLEEVINSVEAAQRIEGNFLDAISKDLKTLKFRIPSYHKFSVPARLIDRGSDTPGQSEFKLPNLHDDEVFDESPAFIEIQEIYNGFTDDLFKKCFLYFAVFPDNVVLKKRFLTHWWIGEGLLDSLDNGDETPEVLAGKILKEFAEKGLIVPVIEKEKKVKRRFRIPPLVRSAAIKLAKQKEFLDYDIGDNPTGKSSDCDRIFLVKGGGSHPPKAPTKDRNLEKTMEVIFNVSQPFPDSALEWLAKEGEVDMRTAKVVEWLRRLRNLKVLYLGRWQSAVDEQHIEVESLEFLKGLKKMKKLRLLSLQGISWINKLPKSIRTLSDLRVLDLKSCFNLEKIPHSIGSLKMLTHLDVSGCYMLNGMPKSISALTELRVLKGFVTGKSNLNDLKGLKKLRKLSINTSRQDFPNETDLCVLQGLGEHGKLRNLTISWGAEDVKQQSSSERNIIRQVSKKLSKQLSKTLTKQRSQFGYEIVELPKELEKLEMECLPKEELPPWLNPSKLTNLKRLYIRGGKLAGLGNETWNAEVVRLKYMADLKIDWRELQKILPNLSYFQRVKCPRVTFCPCDANGVWMKP

>CSA002747

MASPSLILHPQNSTPIPTIPQMIETLSDLYKKLSTALQKHTTQAEPNKPTQQIPGISGTIDNDKDPKVLKLQSNIMLLMEALETMINNRDKKLNVPIQSIETNLDIVIKRVNDAPPGSPLTQKIGEDYLDAIIQDVRTLKFRIPSYRKLSLAKTVAHSGGRGSHALTPIEFVLPNLQGDEVFDESPAFKEVQKIYYEFNDDIFRKCFLYFAVFPENVVLKKQFLTYWWIGEGILDVKGTGDSNLEDEAGGILLKFVEKGLIVPVKEEQKKVKKKFRMPPLVRFASIKLAIENKFLNFDDWGNPTYRSFGCDRILLVKGGGFHPPEAPTKYQNLEEKMVTIFNVSQPFPDSALEWLAKKGDVDMRTTKVVEWLLKLEHLKVLYLGRWQSEVDDEEHVIEVLSLEFLEGLRKMKKLRLLSLQGIFWINELPNSINMLCDLRVLDLKCCYNLEKLPGGIGSLKSLTHLDVTGCYMLNGMPKSISRLTQLRVLKGFVTGKSSLNDLKGLKKLRKLSINTNSPGFPDAKDLRVLRELGEHGELRNLSIMWAAEGLKFDQPPSKTEKGTFIRELTKQISKLTAQPNDETSELPKNLEKLELECLREKNLPNWLNPDNLTSLKKLYIRGGSLETLGNKKWEAAEVVRLKYMTELKIKWRELQNNFPKLSYLQKVKCPRVTLFPCDASGVWIKP

>CSA002916

MKDTTETMCKRYHESSPLGLIAMVREECIETHGNVDIIKQIRETTSNLNFDEVMIGREVEVSNIVKLVIEFSKEHQISIIPIVGMRGLGNTTLAFNHEPVKGHFDETIWLCVWLNMKTPRNLVT

>CSA002921

MAEFLWTFAAQELLKKTVKLAAEQIGLAWGFNNELSNLRDSLLMVEAILRDVDRIKAEHQAVKLWVEKLEAIIFEVDVLLDELAYEDLRRKVEPQKEMMVSNFISFSKTPLVFRLKMANKIKNIAKMLERHYSAASTVGLVAILSKQTEPDFSQIQETDSFLDEYGVIGRESEVLEIVNVSVDLSYRENLSVLPIVGMGGLGKTALAKVIFNHELIKGNFDRAVWVCVSEPFLIKKILRAILETLNSHFGGLDSKEALLQELQKLLNDKKYFLVLDDVWNENPILWNELKGCLLKISQRSGNVVVVTTRSDRVAEIMETHSRYHLTKLSDDHCWSLFKKYAFGNELLRIPELDIVQKELVKRFGGIPLAVKVMGGIVKFDENHEGLQKSLENLMRLQLQDENHVVSTIKLTVDRLPLPSLKQCFAYCSNFPKDFKFRKEALIQMWIAQGFIQPSLGSDEMMEDIGEKYFNVLLSRFLFQDIVKDNRGRIIFCKMHDLIHDVACAISNSPGLKWDPSDLFDGEPWRRQACFASLELKTPDCNENPSRKLHMLTFDSHVFHNKVTNFLYLRVLITHSWFICKLPNSIAKLKHLRYLDISYSTIRELPDSAVLLYNLQTLKLSRFLNGLPKNLRKLVSLRHLEFFSDPCNPKQMPQHLGKLIQLQTLSSFVVGFDDGCKIEELRSLRNLKGKLSLLCLERVKSKKEAMAANLVEKRNISYLSFYWALRCERSEGSNYNDLNVLEGLQPHKNLQALRIQNFLGKLLPNVIFVENLVEIYLHECEMCETLPTLGQLSKLEVLELRCLYSVRSIGEEFYGNYLEKMILFPTLKAFHICEMINLENWEEIMVVSNGTIFSNLESFNIVCCPRLTSIPNLFASQHESSFPSLQHSAKLRSLKILGCESLQKQPNGLEFCSSLENMWISNCSNLNYPPSLQNMQNLTSLSITEFRKLPDGLAQVCKLKSLSVHGYLQGYDWSPLVHLGSLENLVLVDLDGSGAIQLPQQLEQLTSLRSLHISHFSGIEALPEWFGNFTCLETLKLYNCVNLKDMASKEAMSKLTRLTSLRVYGCPQLKLNIGDFERVNISLVPTIS

>CSA002922

MAEFLWTFAVEETLKRTVNVAAQKISLVWGLEDELSNLSKWLLDAGALLRDIDREILRKESVKRWADGLEDIVSEAEDLLDELAYEDLRRKVETSSRVCNNFKFSSVLNPLVRHDMACKMKKITKMLKQHYRNSAPLGLVGKESMEKEDGGNNLRQIRETTSILNFDVVGRETEVLDILRLVIDSSSNEYELPLLIVPIVGMGGVGKTTLAKLVFRHELIKKHFHETIWICVSEHFNIDEILVAILESLTDKVPTKREAVLRRLQKELLDKRCFLVLDDVWNESSKLWEELEDCLKEIVGKFGITIIVTTRLDEVANIMGTVSGYRLEKLPEDHCWSLFKRSANANGVKMTPKLEAIRIKLLQKIDGIPLVAKVLGGAVEFEGDLDRWETTLESIVREIPMKQKSYVLSILQLSVDRLPFVEKQCFAYCSIFPKDCEVVKENLIRMWIAQGFIQPTEGENTMEDLGEGHFNFLLSRSLFQDVVKDKYGRITHFKMHDLIHDVALAILSTRQKSVLDPTHWNGKTSRKLRTLLYNNQEIHHKVADCVFLRVLEVNSLHMMNNLPDFIAKLKHLRYLDISSCSMWVMPHSVTTLFNLQTLKLGSIENLPMNLRNLVRLRHLEFHVYYNTRKMPSHMGELIHLQILSWFVAGFEEGCKIEELGNLKNLKGQLQLSNLEQVRSKEEALAAKLVNKKNLRELTFEWSIDILRECSSYNDFEVLEGLQPPKNLSSLKITNFGGKFLPAATFVENLVFLCLYGCTKCERLPMLGQLANLQELSICFMDSVRSIGSEFYGIDSNRRGYFPKLKKFDFCWMCNLEQWELEVANHESNHFGSLQTLKLDRCGKLTKLPNGLECCKSVHEVIISNCPNLTLNVEEMHNLSVLLIDGLKFLPKGLALHPNLKTIMIKGCIEDYDYSPFLNLPSLTKLYLNDGLGNATQLPKQLQHLTALKILAIENFYGIEVLPEWLRKLTCLETLDLVRCKNLKRLPSRGAMRCLTKLKDFKVIACPLLLLGGQADQEGAKYLHIPAYLCHVYQSRGSPLSKTSSI

>CSA002925

MAIAEFLWTYAVQQVLKKVLELAADQIGLAWGLDKELSNLSQWLLKAEAILADNLLDELVYEYLRTKVEKGSINKVCSSVSSLSNIFIIFRFKMAKKIKSIIEKLRKCYYEATPLGLVGEEFIETENDLSQIRETISKLDDFEVVGREFEVSSIVKQVVDASNQYVTSILPIMGMGGIGKTTLAKTIFNHEEIKGHFDETIWICVSEPFLINKILGAILQMIKGVSSGLDNKEVLLQELQKVMRGKRYFLVLDDVWNENIALWTELKKCLLCFTEKSGNGIIVTTRSIEVGKIMESTLPSHHLGKLFDEQCRSLFKESANADELPMDPELKDLQEELVTRFGGVPFVARVLGGAPKFEGVYEKWVMSLRTTTSIPLQDEDLVLSTLKLSVDRLPSFLLKQCFAYCSNFPKGFKFKKEELIEMWMAQGFIQLHEGRNDITMEENGEKYFNILLSRSLFQDIIKDDRGRITHCKMHDLIYEIACTILNSQKLQEEHIDLLDKGSHTNHRINNAQNLRTLICNRQVLHKTIFDKIANCTRLRVLVVDSSITKLPESIGKMKHLRYLDISSSNIEELPNSISLLYNLQTLKLGSSMKHLPYNLSKLVSLRHLKFSIPQTPPHLSRLTQLQTLSGFAVGFEKGCKIEELGFLKNFKGRLELSNLNGIKHKEEAMSSKLVEKNLCELFLEWDLHILREGSNYNDLEVLKGLQPHKNLQFLSIINYAGQILPPAIFVENLVVIHLRHCVRCETLPMLGELPNLEELNISNLHCLRCIGNEFYGSYDHPNNHKVLFRKLKKFVLSEMHNLEQWEELVFTSRKDAIFPLLEDLNIRDCPILTSIPNIFGCPLKKLHVCGCDEVTRLPKDRSTTLHFH

>CSA002928

MAEFLWTFAVQEVLKKVLKLAADQIGLAWGLDKELSNLSQWLLKAEAILGEINRKKLHPSSVRLWVEDLQLVVHEADDLLDELVYEDLRTKVEKGPINKVRSSISSLSNIFIIFRFKMAKKIKAIIQKLRKCYSEATPLGLVGEEFIETENDLSQIRETISKLDDFEVVGREFEVSSIVKQVVDASIDNVTSILPIVGMGGIGKTTLAKTIFNHEEIKGHFDETIWICVSEPFLINKILGAILQMIKGVSSGLDNREALLRELQKVMRGKRYFLVLDDVWNENLALWTELKHCLLSFTEKSGNAIIVTTRSFEVGKIMESTLSSHHLGKLSDEQCWSLFKKSANADELPKNLELKDLQEELVTRFGGAPLVARVLGGALKFEGVYEKWVMSLRTTTSIPLQDEDLVLSTLKLSVDRLPSFLLKQCFAYCSNFPKGFKFKKEELIEMWMAQGFIQLHEGRNEITMEENGEKYFNILLSRSLFQDIIKDDRGRITHCKMHDLIYEIACTILNSQKLQEEHIDLLDKGSHTNHRINNAQNLRTLICNRQVLHKTIFDKIANCTCLRVLVVDSSITKLPESIGKIKHLRYLDISNSKIEELPNSISLLYNLQTLKLGSSMKDLPQNLSKLVSLRHLKFSMPQTPPHLGRLTQLQTLSGFAVGFEKGFKIGELGFLKNLKGSVTEQLPQQLEHLIALRSLYINDFDGIEFPSKKAMQCLTQLIHVDVHNCPSSQILSHDLKAKAHAKANLVQW

>CSA002955

MADFLWSFAVDEVLKKTVKLVAEQIGMSWGFKKDLSKLRDSLLMVEAILRDVNRIKAEHQALRLWVEKLEHIVFEADVLLDELSYEDLRRKVDARPVRSFVSSSKNPLVFRLKMANKIKAIAKRLDEHYCAASIMGLVAITSKEVESEPSQILETDSFLDEIGVIGREAEVLEIVNKLLELSKQEAALSVLPIVGIGGLGKTSLAKAIFHHEMIRENFDRMIWVCVSEPFVINKILRAILETLNANFGGLDNKEALLQELQKLLRNKKYFLVLDDVWNENPDLWNELRACLLKANKKFGSVIVVTTRSDEVANIVETNHQRHRLRKLSNDYCWTLFEKCAFGSDLPVTPRVDHVIREELVKRFGGIPLVVKVFGGMVKLDKNKCCQGLRSTLENLIISPLQYENSILSTIKLSVDRLPSSSLKQCFAYCSNFPRGFLFIREPLVQMWIAQGFIHLPSGSNVTMEDIGANYFNTLLSRSLFQDVVKDDRERILYCKMHDVVHDVACAISNAQKLRLSGKSNGDKALSIGHEIRTLHCSENVVERFHLPTFDSHVFHNEISNFTYLCVLIIHSWFIHQLPDSIAKLKHLRYLDISHSLIRTLPDSIVSLYNLQTLRLGSKIMHLPTKLRKLVNLRHLEFSLSTQTKQMPQHLSRLLQLQTLSSFVVGFDKGCKIEELGPLNNLKGELSLFHLEHVKSKTEAMAANLAMKENISDLYFQWSLLSEREDCSNNDLNVLEGLRPHKNLQALKIENFGGVLPNGLFVENLVEVILYDCKRCETLPMLGHLSKLELLHIRCLDSVKSIGDEFYGNNNSYHNEWSSLLFPKLKTLHISQMKSLELWQEIGSSSNYGATFPHLESLSIVWCSKLMNIPNLFQVPPKLQSLKIFYCEKLTKLPHWLNLCSSIENMVICNCPNVNNNSLPNLKSMPNLSSLSIQAFEKLPEGLATIHNLKRLDVYGELQGLDWSPFMYLNSSIEILRLVNTGVSNLLLQLPRQLEYLTALRSLDIERFSDIDSLPEWLGNLTSLETLNLRYCKNLKSFPSIEAMSNLTKLSRLETYECFQLKLDEGSYERAKIAHVHDISC

>CSA003127

MNSLLQNSETQPTAPAETRTRISEWVTQTVDGSTVHGVENELLVLQKMLDIPPISGGGNGFRAIGIIGVRGIGKSTISRAFLQNPEVKSKFLPRIWISMSENFTEDADPKIALLKRILISLGVDTKFPGGETLGSLLYALRLQLRGKRYLIVLDDVQEFKTEEEQNDWYWDLNSCEKNGEKLRDGFPKGNGGAVILTSRSEKAAKAMVGEGNLRCLVPHKDPESFWEIFRQEVVKDGISIPDEILNFKELKVKLLKKCGGLPLIAKMMGEIQFKKELEKKKNTEQQRREEEDR

>CSA006282

MDIISPVVGPIVEYTLKPIGRQLSYLFFIRQHIQNLESQVELLKNTKESVVNKVNEAIRNAEKIESGVQSWLTKVDSIIERSETLLKNLSEQGGLCLNLVQRHQLSRKAVKLAEEVVVIKIEGNFDKVSSPVALSEVESSKAKNSDFVDFESRKPTIDKIIAALMDDNVHTIGVYGMGGVGKTMLVQEISKLAMEQKLFDEVITSTVSQTPDLRRIQGQLGDKLGLRFEQETEEGRALKLLNRLKMERQKILIVLDDVWKQIDLEKIGIPSIEDHSGCKILFTSRDNDVLFNDWRTYKNFEIKFLQEDETWNLFRKMAGEIVETSDFKSIAVEIVRECAHLPIAITTIARALRNKPASIWKDALIQLRNPVFVNIREINKKVYSSLKLSYDYLDSEEAKSLFLLCSMFPEDYIIDCQVLHVYAMGMGLLHGVESVAQARNRITKLVDDLISSSLLLKESNVDLVMYVKMHDIVRDVAIIIASKDDRIFTLSYSKGLLDESWDEKKLVGKHTAVCLNVKGLHNLPQKLMLPKVQLLVFCGTLLGEHELPGTFFEEMKGMRVLEIRSMKMPLLSPSLYSLTNLQSLHLFDCELENIDVICELNKLENLSLKGSHIIQIPATISQLTQLKVLDLSECYALKVIPPNILVNLTKLEELYLLNFDGWESEELNQGRRNASISELSYLSQLCALALHIPSEKVMPKELFSRFFNLEKFEIFIGRKPVGLHKRKFSRVLCLKMETTNSMDKGINMLLKRSERLHLVGSIGARVFPFELNENESSYLKYLYINYNSNFQHFIHGQNKTNLQKVLSNMERLELSYLENLESFFHGDIKDISFNNLKVIKLLSCNKLGSLFLDSNMNGMLLHLERINITDCEKVKTVILMESGNPSDPVEFTNLKRLRLNGLPQLQSFYSKIEQLSPDQEAEKDERSRNFNDGLLFNEQVSLPNLEDLNIEETHNLKMIWCNVLIPNSFSKLTSVKIINCESLEKLFSSSMMSRLTCLQSLYIGSCKLLEEVFEGQESGVTNKDIDLLPNLRRLDLIGLPKLQFICGKNDCEFLNFKSIPNLTIGGCPKLEAKYLIQVLDNMKDLTIDLRRLEEILNKEKSVVELDLSLETSKDGGELFGKLEFLDLCGSLSPDYKTITHLPMEIVPILHNLKSLIVKRTFLEEIFPMTRLGNVEEWQNKRFKLSSLALRELPKLKHLCNEDLQKNSSMLQNLKYFSIKGCGKLNMFVPSSMSFRNLVDLKVMECHKLIYLINPSVARTMGQLRQLEIRRCKRMTSVIAKEENDEILFNKLIYLVVVDLPKLLNFHSGKCTIRFPVLRRISVQNCPEMKDFCTGIVSTPHLLTESIIHYDDATNKYIPILKDYSKEAIVKDMNVAIRQVWENHYDFNLHCLFEVENLKEENQCESSSHVEQLETQCLTFHAFISVECFTLAIPTHVPSHTTALRSTHDDAVVVSVQSPIANAFKSNSFEF

>CSA006679

MQLRVPKYPVGIDIQVDNLLFHVVSDELITMVGLYGIGGIGKTTLARALYNKIVDDFESCCFLANVREASNQYRGLVGLQNELLREILVDDSIKVSNLDIGISIIRDRLCSKKILLILDDVDTSEQLEALAGGRDWFGPRSMVIATTRNKHLLAIHEFDILQSVKGLNDDEALELFSWHAFKTSCPSSDYLDLSKRVVRYCKGLPLALEVVGEDINKVKLMLEACGCLCLEKRTTKLMNLSLLTIDESNQVEKVVYKFGFLRNNGSCHPFIEKVGLVQKQLL

>CSA006693

MAGALIGGAALGVPFNELATLLKNFGERAWSFNSVLNETESKVNDIIPLVKEIDGLNESLDYPREETEKLKNLLEYAGKLLRRCLRVGKADLIRKSSHTEKLRELNARIKSFSDVVLFQTSRDGKKTLSLVTEIKEVVRRLDSKSGLSNPVDLVVTVPVISEESVGLEKPVEKLKAKLFRDGVRLLVVTAPGGCGKSTLAEIFCHDKQVKNKFQRNILFLVVSSKPETKRILISIIQRLGGPIESGSVSDDEAFRLLEVRVGELSPNPVLIVLDDVWDGSESNKLLEKFSRLPNCKVLVTSRFKFPAFGESYDLEPLDHKDAMELFRRWASRGNRVLQFPDERIVEKIVRGCKRFPLALKVIAGSLSGRATSVWEVTGRKLSRGDSILGSEKELQKCLKDTLDAIPDDKIVLKECFMDLGSFPEDQRIRAATFIDICAVLYEQDECETMSNLDELFTRTLVNTVSLRNKAHEDDYYSESYITQHDVLRELAVLLTNEQPVDQRTRLLVDINKNEFPKWWSVRQMQPVKARLLSITTDEKFSSCWPDMEAPEVEVLILNPGSETYKLPDFAKKMNRLKALIVRNYRSFPTELTSDYQLINCLSRLERISLERISISSFIDQNLKPLWHLKKLSFFMCKIDKAFTDCSTQISYMLPNLLEISIDFCNDLVAFPVGLCEVVTLEKLSITNCHILSSLPEEIGQLINLKILRLRSCIHLEKLPESISRLRELVYLDISHCVGLTKLPDKIGNLQKLEKLNMWSCPNMRKLPKSVGNLKNLKEVVCESEMKIWVNFVAPRLGNVVKEHKEEINLDFLN

>CSA006711

MGSSTAATESMAFEWSYDVFLSFRGEDTRTNFTSHLDMALRQKGVNVFIDDKLERGEQISESLFKSIQEASISIVIFSQNYASSSWCLDELVNIIECKKSKGQNVFPVFYKVDPSDIRKQTGSFGEALAKHQPKFQTKTQIWREALTTAANLSGWDLGAYRREADLIRDLVKEVLSTINRTRTPLYVAKYPVGIDSQLEYMKFHSHHLNKGNKFQYWTQNEYESDIGVYMVGIYGIGGLGKTTLAKALYNKIASQFEGCCFLSNVRQASNQFNGLVQLQQNLLYEILEDDLKFVNLDKGITIIRNRLRSKKVLIVLDDVDKLEQLEALVGGRDWFGQGSKIIVTTRNSHLLSSHGFDEMHNIQGLNQDRAIELFSWHAFKESHPSSNYLDLAERATSYCKGHPLALVVLGSFLCNRGQTEWRSILDKFENSLNNDIKDILQLSFDGLEGGVKDIFLDISCLFVGEKYNNCAKKMLSACHLNVDFGIMILMDLSLVTIEKDRVQMHGLIQQMGHSIVHNESFESGKRSRLWSERDIWNVFVNNSGTDAIKAIKLDLPNPINVNVDPKAFFRSMKNLRLLIIRNAQVCTKIKYLPNSLKWIEWQGFAHRTFPSCFITKNLVGLDLRRSFIKRFGKRLEDCERLKHVDLSYSTLLEKIPDLSAASNLEELYLINCTNLGMIDKSVFSLNKLTVLNFKGCSNLKKLPKGYFMFSSLKILNLSYCQELEKIPDLSSASNLQSLLLNGCTNLRVIHESVGSLNELVLLDLGQCTNLSKLPSYLRLKSLVYLVLFGCGKLESFPTIAENMKSLRCLDLHSTAIKELPSSLGYLTQLDKLHLTGCTNLISLPNTIYLLRNLNELHLGGCSRFEMFPHKWVPTIQPVCSPSKMMEAASWSLEFPHLVVPNESICSHFTLLDLKSCNISSAKFLEILCDVAPFLSDLRLSENKFSSLPSCLHKFMSLSNLELRNCKFLQEIPNLPQNIRNLDASGCKSLARSPDNIVDIISIKQDLELGEILREFLLTDIEIPEWFSYKTTSNLVSASFRHYPDMERTLAVAVSFKVNGDSSETCSLEVNDWNKVFVWFEVHEAHGVTVTRYGVHVTEQLHGIQTDVKWPMVNYADFYQLEKLRRDLDFEDLKASLKKSAVQIPKQHCMHFSMIQRQ

>CSA006724

MDSSTVAAESSTFKWSYDVCLSFRGEDTRDNFTSHLDMALRQKGVNVFIDDQLERGEQISETLFKSIHKTSISIVIFSENYASSTWCLDELVEIIECKKSKGQEVLPIFYKVDPSDVRKQTGWFGGALAKHEANFMEKIPIWRDALTTAANLAGWDLGTIRKEADLIQVIVERVLSILNQTHTPLKVAEYPVGIDYKIESLYWTQEMYKSECVDMVGIYGIRGIGKTTLAKALYNKIASQFEGCCFLSNVREASKQFNGLAQLQKKLLFQILKYDLEVVDLDRGHNIKQADNFLIATNSKMQAYDHVHDDIRFTPKRGMEGLTEITLSKSIWDKFVKDHNITSEILASNDSNALVRGYIDGDKLYLVTHDRQHFQEYLG

>CSA006742

MGSSVVGDESFSSSPNFNYDYDVFFSFRGEDTRSNFISHLHMALRLKEVNVFIDDKLKRGEQIYESLLKFIERSRLSLVIFSKDYASSTWCLDELVKIIECKKSKGQAVWPVFYKVDPSEVRKQTGGFGEALAKHEANKLLTNKIQPWREALTFAAGLSGWDLANSKDEAELIQKIVKRVLSAVNPMQLLHVAKHQVGVDSRLRKIEELVSHIGSEGVNLVGLYGIGGIGKTTLAKALYNKIATQFEGCCFLQDVRREASKHGLVQLQETLLNEILKEDLKVIVSRDRGINIIRSRLCSKKVLIVLDDVNDLEQLEALVGGRDWFGQGSKIIVTTRNEHLLSSHGFDAFQLACF

>CSA006744

MDSSTVATESPTFKWTYDVFLSFRGEDTRTNFTSHLDMALRQKGVNVFINDKLERGEQISESLFKSIQEASISIVIFSQNYASSSWCLDELVNIIECKKSKGQNVFPVFYKVDPSDIRKQTGSFGEALAKHQPKFQTKTQIWREALTTAANLSGWNLGTRKEADLIGDLVKKVLSVLNRTCTPLYVAKYPVGIDSKLEYMKLRSHSLFEKSNKFHYRKQHEYESDTGVYMVGLYGIGGIGKTTLAKALYNKIASQFEGCCFLSNVREASKQFNGLAQLQETLLYEILTVDLKVVNLDRGINIIRNRLCLKKVLIVLDDVDKLEQLEALVGGRDWFGQGSRIIVTTRNKHLLSSHGFDEMKNILGLDEDKAIELFSWHAFKKNHPSSNYLDLSKRATSYCKGHPLALVVLGSFLCTRDQGTDAVKGIKLDFPNSTRLDVDPQAFRKMKNLRLLIVQNARFSTKIEYLPDSLKWIKWHGFRQPTFPSFFTMKNLVGLDLQHSFIKTFGKRLEDCERLKLVDLSYSTFLEKIPNFSAASNLEELYLSNCTNLGMIDKSVFSLDKLTVLNLDGCSNLKKLPRGYFMLSSLKKLNLSYCKKLEKIPDLSSASNLTSLHIYECTNLRVIHESVGSLDKLEGLYLKQCTNLDLTMGEISREFLLTGIEIPEWFSYKTTSNLVSASFRHYPDMERTLAACVSFKVNGNSSEREYMWLVTTSLALGSMEVNDWNKVLVWFEVHEAHSEVNATITRYGVHVTEELHAIQTDVKWPMVNYADFYQLEKLQSLDIEELLLKRFFEEMSCWSNSQAMLYAANYDPEAIIDSNIQPMIFPLHVTYNGETFICGMEGMGDTTLANSLCNKFNWPNDNVRAREALDNSTSFLHFRGGKFNGGSWSSSHHRKRGDGERGTNITTRTISSKRYLILFHKAGSYDDLFNFAGSHRLIAGSRSYDSLNGRGDVRLLIERVDTSLL

>CSA006756

MQLDVAKYPVGIDIQVSNLLPHVMSNGITMFGLYGVGGMGKTTIAKALYNKIADEFEGCCFLSNIREASNQYGGLVQFQKELLCEILMDDSIKVSNLPRGITIIRNRLYSKKILLILDDVDTREQLQALAGGHDWFGHGSKVIATTRNKQLLVTHGFDKMQNVGGLDYDEALELFSWHCFRNSHPLNVYLELSKRAVDYCKGLPLALEVLGSFLHSIGDPSNFKRILDEYEKHYLDKDIQDSLRISYDGLEDEGITKLMNLSLLTIGRFNRVEMHNIIQQMGRTIHLSETSKSHKRKRLLIKDDAMDVLNGNKEARAVKVIKLNFPKPTKLDIDSRAFDKVKNLVVLEVGNATSSESSTLEYLPSSLRWMNWPQFPFSSLPTTYTMENLIELKLPYSSIKHFGQGYMSCERLKEINLSDSNLLVEIPDLSTAINLKYLNLVGCENLVKVHESIGSLSKLVALHFSSSVKGFEQFPSCLKLKSLKFLSMKNCRIDEWCPQFSEEMKSIEYLSIGYSTVTYQLSPTIGYLTSLKHLSLYYCKELTTLPSTIYRLTNLTSLTVLDSNLSTFPFLNHPSLPSSLFYLTKLRIVGCKITNLDFLETIVYVAPSLKELDLSENNFCRLPSCIINFKSLKYLYTMDCELLEEISKVPEGVICMSAAGSISLARFPNNLADFMSCDDSVEYCKGGELKQLVLMNCHIPDWYRYKSMSDSLTFFLPADYLSWKWKPLFAPCVKFEVTNDDWFQKLECKVFINDIQVWSSEEVYANQKERSGMFGKVSPGEYMWLIVLDPHTRFQSYSDDIMDRRSLKIIDLNQLSSEINSSQSILGKITVSFEVTPWYKDVVIKMCGVHVIMGE

>CSA006757

MNRATGSSSSHLRLPFDVFLSFRGEDTRSNFTSHLHMALCQKGINVFIDDDKLPRGEEICTSLLKAIEESKISIVIISENYASSHWCLDELMKIIMCNKSNNRQVVFPVFYKVNPSHVRRQRGVFGEEFAKLQVRFSNKMQAWSEALTFISTMSGWDLKNYENEASLIQIIVQEVRKKLRNSATTELDVAKYPVGIDIQVSNLLPHVMSNEITMVGLYGIGGMGKTTLAKALYNKIADEFEGCCFLSNVREASNQYWGLVELQKTLLREILMDDSIKVSNVGIGISIIRDRLCSKKIILILDDVDTHEQLQALAGGHHWFGHGSKVIATTRNKQLLASHGFNILKRVNGLNAIEGLELFSWHAFNNCHPSSDYLDVSKRAVHYCKGLPLALEVLGSFLNSIDDQSKFERILDEYENSYLDKGIQDILRISYDELEQDVKDIFLYISCCFVHEDKNEVQMMLKECDSRFRLEMGIKKLTDLSLLTIDKFNRVEMHDLIQQMGHTIHLLETSNSHKRKRLLFEKDVMDVLNGDMEARAVKVIKLNFHQPTELDIDSRGFEKVKNLVVLKVHNVTSSKSLEYLPSSLRWMIWPKFPFSSLPSTYSLEKLTELSMPSSFIKHFGNGYLNCKWLKRINLNYSKFLEEISDLSSAINLEELNLSECKKLEYADGKYKQLILMNNCDIPEWFHFKSTNNSITFPTTFNYPGWKLKVLAACVKVQVHDPVNGYHRGGDLECEVFFKDILVWSSGDWTNYLGYDSRWLPLGASPSEYTWFIVLNPHRDFSLDDWDDTMERSPETDLSQLCFGINSMEMDRNRSNDKWNSIGGSIWKNFTVLFEPRPLSRDTIISIKGCGVHVIME

>CSA006758

MCKELRGQVVLPIFYKVNPSQVRKQNGAFGEAFAELEVRFFDKMQAWGEALTAVSHMSGWVVLEKDDEANLIQKIVQQVWKKLTCSTMQLPVTKYPVGIDRQFENLLSHVMIDGTRMVGLHGIGGMGKTTLAKTLYNRIADDFEGCCFLANIREASKQHEGLVRLQEKLLYEILMDDFIRVSDLYKGINIIRNRLCSKKILLILDDIDTSEQLQVLAGGYDWFGYGSKVIVTTRNEHLLDIHGFNKLRSVPELNYGEALELFSWHAFQCSSPPTEYLQLSKDAVNYCKNLPLALEVLGSFLYSTDQSKFKGILEEFAISNLDKDIQNLLQVSYDELEGDVQEMFLFISCFFVGEDKTMVETMLKSCGCLCWEKGIQKLMNLSLLTINQWNKVEMHDLIQQLGHTIARSKTSISPSEKKLLVGDDAMHVLDGIKDARAVKAIKLEFPKPTKLDIIDSTAFRKVKNLVVLKVKNVISPKISTLDFLPNSLRWMSWSEFPFSSFPSSYSMENLIQLKLPHSAIQHFGRAFMHCERLKQLDLSNSFFLEEIPDLSAAINLENLSLSGCISLVKVHKSVGSLPKLIDLSLSSHVYGFKQFPSPLRLKSLKRFSTDHCTILQGYPQFSQEMKSSLEDLWFQSSSITKLSSTIRYLTSLKDLTIVDCKKLTTLPSTIYDLSKLTSIEVSQSDLSTFPSSYSCPSSLPLLTRLHLYENKITNLDFLETIAHAAPSLRELNLSNNNFSILPSCIVNFKSLRFLETFDCKFLEEIPKIPEGLISLGAYHWPNLPTT

>CSA006768

MGSSIVGAESSTSSSSSFKWSFDVFLSFRGDDTRSNFTGHLDMALRQKGVNVFIDDMLKRGEQISETLSKAIQEALISIVIFSQNYASSSWCLDELVKIVECKKSKGQLVLPIFYKVDPSDVRKQTGCFGEALAKHQANFMEKTQIWRDALTTVANFSGWDLGTRKEADFIQDLVKEVLSRLNCANGQLYVAKYPVGIDSQLEDMKLLSHQIRDAFDGVYMMGIYGIGGIGKTTLAKALYNKIANQFEVLIVLDDVDKLKQLEALVGERDWFGHGSKIIVTTRNSHLLSSHEFDEKYGVRELSHGHSLELFSWHAFKKSHPSSNYLDLSKRATNYCKGHPLALVVLGSFLCTRDQIKWRTILDEFENSLSEDIEHIIQISFDGLEEKIKEIFLDISCLFVGEKVNYVKSVLNTCHFSLDFGIIVLMDLSLITVENEEVQMHDLIRQMGQKIVNGESFEPGKRSRLWLVHDVLKVFADNSGTIAVKAIKLDLSNPTRLDVDSRAFRNMKNLRLLIVRNARFSTNVEYLPDNLKWIKWHGFSHRFLPLSFLKKNLVGLDLRHSLIRNLGKGFKDCKRLKHVDLSYSSLLEKIPDFPATSNLEELYLNNCTNLRTIPKSVVSLGKLLTLDLDHCSNLIKLPSYLMLKSLKVLKLAYCKKLEKLPDFSTASNLEKLYLKECTNLRMIHDSIGSLSKLVTLDLGKCSNLEKLPSYLTLKSLEYLNLAHCKKLEEIPDFSSALNLKSLYLEQCTNLRVIHESIGSLNSLVTLDLRQCTNLEKLPSYLKLKSLRHFELSGCHKLEMNCKFLQEIPNLPHCIQKMDATGCTLLGRSPDNIMDIISSKQDVALGDFTREFILMNTGIPEWFSYQSISNSIRVSFRHDLNMERILATYATLQVVGDSYQGMALVSCKIFIGYRLQSCFMRKFPSSTSEYTWLVTTSSPTFSTSLEMNEWNHVTVWFEVVKCSEATVTIKCCGVHLTEEVHGIQNDVKGPGVVYTVFDQLDKLPSRDVIKSFGQEVSAKSDCNAMLHAENFPVWNDSKMQQHMNFPLHVTSQGVTRIRGMEGMAETTLANSICNKYERSRNLFSAKKALNHSTGFLCGDGNGLSWEMVDRPILSDRLSSQKYLRIFDDRDRYGDLNDVAHGTGNRFRSRFLRMDDIKEDDIREEPYWKYMERLFQTDPIS

>CSA006769

MRKSLGRFGMGSNAAGAESSSSSPINWIYDVFLSFRGEDTRSNFTSHLHMFLRHKGVNVFIDDRIERGEQISEALLKTIQCSLISIVIFSENYASSTWCLDELVEIIECKKSKGQKVLPIFYKVDPSDVRKQNGCYGEGLAKHEANFMEKIPIWRNALTTAANLAGWDLGTIRNEADLIQVIVKEVSSTLNVTTPSDKPLLVGIDSKIESLYWPTEEMYKSECVDMLGIYGIRGIGKTTLAKALYNKIASQFEGCCFLSNVREASKQLNGLAQLQKKLLFQILKYDLEDVDDLDRRNNIIKHRLHSKKVLILLDDVDEMKQLKALAGGHDWFGQGSKIIVTTRDKHLLDSHGFGQTYEVEGLWEHNAFELFCWHAFKKSHPSSNYLDLSERATRHCKGHPLALVVLASFLCGRDQAEWSGLLDGFENSLRKGIKDVLQLSFDGLEDEVKKFFLDISCLLVGETVTYVKKMLSEFHSILDFKISNLRHLSLIRMEEYDDDRVQMHDLIKQMGHKIVYDECGDEPGKRSRSGWRRTFWRCLVTIQ

>CSA006843

MEVESIVQDVIEASQQQLNSILPVYGTGGSGKTTLAQLVFNDERIGKQFHHTVWVCVSQPFVINEILQSILKKVSKSNDNRSKDDKDTLIRNLKEVMGGKRYFLVLDNVWNENKMLWEKLKECLMSIVEELGSSVLVTTRSRKIAEMMKETLDTYHLNKLTDDQCWSVFSYFAKANAVPITSNLELVREEVVKKIGGLPLLARVLGEAAQFSGDYEKWVEILKSIPTTPLKYEESSYVKYILKLSVDRLPKASIKQCFAYCSNFPKGYWFDKKQVIKMWMAHGFTRPDEGNNETMEDTGKRYFNILLSYCLFQDADDDKWHIGRKFRMHDLIHDIACDVSSDKRLQLDHSSSSKWKVGFETGRKISELGGLRNLKGLLKLHRLEHVESKEEAKAAKLVEKEKVEGLNLSWRGKWKNSGESE

>CSA007358

MAEFIINVASVIVTPIGKYVIKPIGNQLGYIVFYNRNKNEIKEQLESLETTKKDLDLRVEDAKSKAYTIFTKVSEWLVAADDEIKKSDELFNSNPPCLNFLQRHQLSRKARKRATDIRRLKDGGNNFLEVGCPAPLPDTMNTIVPEAYQTLGSKTSMAKQIKDALAKPEVRKVGIYGMGGVGKTYLLKEVKKLVLEEKLFDLVIDVTVGQSNDVMNMQQQIGDFLNKELPKSKEGRTSFLRNALVEMKGNILITFDDLWNEFDIINDVGIPLSKEGCKTLVTSRFQNVLANKMNIKECFKVTCLDDEESWKFFKKIIGDEFDAKMENIAKEVAKQCGGLPLALDIIAKTLKRSRHINYYWEGVLSKLKNSIPVNIDVGEKVYASLKLSYEHLDGEEVKSLFLLCSVFPDDHGISVNDLQMYVMGMGLLKMVNTWKEARAEAHYLVEDLTSSSLLQRLKNRDVKMHDIVRDVAIYIGPDFNMSTLYYGYSTSSKGLDEDKCRSYRAIFVDCKKFCNLLPNLKLPKLELLILSFPFWGKDRNIDIMDAYFEGMENLKVLDIEGTSFLQPFWTPLKNLRTLCMSYCWCEDIDTIGHLKQLEILRISNCRGITELPTSMSELKQLKVLVVSHCFKLVVIHTNIISSMTKLEELDIQDCFKEWGEEVRYKNTWIPNAQLSELNCLSHLSILRVRVLKLTILSEALSSQMLKNLREFFIYVGTHEPKFHPFKSWSSFDKYEKNMSFNMKSQIVSVNPTKLSILLEGTKRLMILNDSKGFANDIFKAIGNGYPLLKCLEIHDNSETPHLRGNDFTSLKRLVLDRMVMLESIIPRHSPINPFNKLKFIKIGRCEQLRNFFPLSVFKGLSNLRQIEIYECNMMEEIVSIEIEDHITIYTSPLTSLRIERVNKLTSFCSTKSSIQQTIVPLFDERRVSFPELKYLSIGRANNLEMLWHKNGSSFSKLQTIEISDCKELRCVFPSNIATSLVFLDTLKIYGCELLEMIFEIEKQKTSGDTKVVPLRYLSLGFLKNLKYVWDKDVDDVVAFPNLKKVKVGRCPKLKIIFPASFTKYMKEIEELEMVEPFNYEIFPVDEASKLKEVALFQSLETLRMSCKQAVKERFWVMSKFFKLKSLELFGCEDGKMISLPMEMNEVLYSIEELTIRGCLQLVDVIGNDYYIQRCANLKKLKLYNLPKLMYVLKNMNQMTATTFSKLVYLQVGGCNGMINLFSPSVAKNLANLNSIEIYDCGEMRTVVAAKAEEEEENVEIVFSKLTGMEFHNLAGLECFYPGKCTLEFPLLDTLRISKCDDMKIFSYGITNTPTLKNIEIGEHNSLPVLPTQGINDIIHAFFTIEVFFTKVFSCTLESCSP

>CSA008307

MAEFLWTFAVQEVLKKIVNFGAEQISLAWGLEKELSHLKKWLLKAQTILADINTKKSHHHSVGLWVEELHDIIYEADDLLDEIVYEQIRQTVEQTGKLRKVRDSISPSKNSFLFGLKMAKKMKKITKTLYEHYCEASPLGLVGDESTTESEAALNQIRETTSILDFEVEGREAEVLEILKLVIDSTDEDHISVISIVGMGGLGKTTLAKMVFNHDAIKGHFDKTVWVCVSKPFIVMKILEAIFQGLTNTSSGLNSREALLNRLREEMQGKKYFLVLDDVWDKENCLWDELIGNLKYIAGKSGNSIMVTTRSVEVATMVKTVPIYHLKKLSDDHCWALLKKSANANQLQMNSKLENTKNILVRKIGGVPLIAKVLGGAVKFEEGGSESWMAKIESFARNISIEDKDFVLSILKLSVESLPHSALKQCFAYCSNFPQDYEFDKDEAIQMWIAEGFIQPEQERENLTMENIGEEYLNFLLSRSLFEDAIKYDGRIVTFKIHDLMHDIACAISNHHKMDSNPISWNGKSTRKLRTLICENEEAFHKIQTDIICLRVLVLKWFDTNTLSTIMDKLIHLRYLDISNCNINKLLRDSICALYNLQTLKLGYIECDLPKNLRNLVNLRHLEFKKFFDMGQMPSHMGNMIHLQTLSEFVVGLEKGCKIDELGPLKDLKGTLTLKNLQNVQNKDEAMAAKLVEKKYLRHLIFQWFLNLYDRGEYDEDDNKQVLEGLQPHKNVQSLDIRGFQGRVLNNNIFVENLVEIRLVDCGRCEVLPMLGQLPNLKKLEIISMNSVRSIGSEFYGVDCNDRNSSAFPQLNKFHICGLKKLQQWDEATVFASNRFGCLKELILSGCHQLAKLPSGLEGCYSIEYLAIDGCPNLMLNVQNLYNLYHLDIRGLKRLPDEFGKLTNLKKLRIGGCMQNYEFSPFIHLSSQLVELELTDDGSSGSETTQLPQQLQHLTNLKVLKIADFDDIEVLPEWLGNLTCLATLVFLECKNLKELPSREAIQRLTKLDDLVIDGCPKLLLGEGDQERAKLSHLPSKCVRYNNFGFRC

>CSA008754

MAEAILYIVTAHIIFKLGSFALQELGSLWRVNCELHKLKDSLSAIQVVLHDAEEQQSKNNQVKDWVLKLEDVLYEIDDLIDKFSYQTLRRQVMAKHQRYRKRVRILFSKFKSNWEIGFKIKEIRPGLLAINEDKNQFSFTKHVIERRDDDEGLRKSWETHSFEVIGRNDDKEAVINLLLNSNTKEDIAIASIVGMAGLGKAALVQSIYNSKRIMTRFQLKLWVCVYDEFDPKVIIQKKPKSFLQIDSLQRGKKNQ

>CSA009413

MAVTDFFVGEIATELLRMMVQLSTKSCLCKTTAAQIANSIQQILPIIEEIKYSGVELPAHRQFQLDRFSETLRRGIEISEKALQCGRLNIYRNLRLARKMEKLEKDICRFINGTMQAHILADVHHMRFQTTERFDRLEGVLLERRLESMKIRADASGEERWWVEEAFKKAEEEERYESNFVNIGTGLRVGKRKLKELVIGKEDLTAVGISGIGGSGKTTLAREFCKDPEVRRHFKERILFLTVSQSPDVEQLRRTIWEFVMGSDSVNSNNLILHGRPSNSALLVLDDVWSISVLENVIPNVTGCKTLVVSRFKFPEVLRETYEVELLKESEAIALFCHSAFGQQSIPLSANHNLVKQVVNECKCLPLALKVIGASLRGQSEMFWNNAKSRLSRGEPICESHENKLLQRMAISIERLSSKVRECFLDLGCFPEDKRIPLDILINVWKELHDLDDEEALAVLFELSQKNLLTLVKDARGGDIYSSYYEMYVTQHDVLRDLALHFSCQENVNDRKRLLMPKSDTELPKEWLRKSEQPFNAQLVSIHTGEMEEMDWAPMIFPEAKVLILNFSSSGYFLPSFLCNMPKIRALIVLNNNATHATLTNFSVFSSLVNLRGIWLEKISMTQLFDACTPLKHLRKLSLVFCKINNSLDEWAVDVSQIFPFLFELKIDHCNDLRKLPSSICEMQSLKCLSVTNCHNLSQLPTNLWKLKNLQILRLFACPLLKTLSPSICVLSCLKYIDISQCVYLTSLPEEIGKLTSLEKIDMRECSLIRRLPRSVVSLQSLCHVICEEDVSWLWEDLKSHMPNLYIQVAEKCFNLDWLKE

>CSA009587

METVIAILGTVCEYAVAPIGRQVGYVSSYKKNINDLKDQLQNLVDTKTRLQHMVNEARSSAYNIQSDVSSWLNQVDKIIEQSNDILYKNENESNSKYCSNKLNFIHQYQMSKKAKKMVKVISQIIEKRKLMFHQVGYPTPLSRIHGSSTSSSHGYDQILESRTSIAKQIRDALVDCNVNKVGVYGMGGVEKTTLLKQVTPLVMEEKLFDHVIIVNVGQTLGVEGIQAQIGDKLRLELNKKVESKEGRASLLQNKLEMESNVLLVLDDLWKGLDLEEVGIPCRSESCEKGCKILITSRDRDVLTNEMDTQVYFEVKPLSEKESWEFFKNMIGEFDNKCIELIGKEMVKKCGGLPIALATIVKTLKGKEVPIWKDALKQLKNPIAVDVKGVTEL

>CSA009602

MLLGIGLDDVRFVGIWGMGGIGKTTIARIIYKSVSHLFDGCYFLDNVKEALKKEDIASLQQKLLTGTLMKRNIDIPNADGATLIKRRISNIKALIILDDVNHLSQLQKLAGGLDWFGSGSRVIVTTRDEHLLISHGIERRYNVEVLKIEEGLQLFSQKAFGEEHTKEEYFDVCSQVVDYAGGLPLAIEVLGSSLRNKPMEDWINAVEKLWEVRDKEIIEKLKISYYMLEKSEQKIFLDIACFFKRKSKKQAIEILESFGFPAVLGLEILEEKCLITTPHDKLHMHDLIQEMGQEIVRQNFLNEPEKRTRLWLREDVNLALSRDQEGESHLNAKAFSEMTNLRVLKLNNVHLSKEIEYLSDQLRFLNWHGYPLKTLPSNFNPTNLLELELPNSSIHHLWTASKSMETLKVINLSDSQFLSKTPDFSGVPNLERLVLSGCVELHQLHHSLGNLNHLIQLDLRNCKKLTNIPFNISLESLKILVLSGCSNLTHFPKISSNMNHLLELHLDETSIKVLHSSIGHLTSLVLLNLKNCTDLLKLPSTIGSLTSLKTLNLNGCSKLDSLPESLGDISSLEKLDITSTCVNQAPMSFQLLTKLEILNCQGLSRKFLHSLFPTWKFTRKFSNYSQGLKVTNWFTFGCSLRILNLSDCNLWDGDLPNDLHSLASLQILHLSKNHFTKLPESICHLVNLRDLFLVECFHLLSLPKLPLSVREVEARDCVSLKEYYNKEKQIPSSELGITFIRCPISNEPSESYNIDQPHFSAIHVRTTTQRYIEVINFFTFLFSS

>CSA009604

MLLINYASNACTYLLSIGSLITTKKRDVLHQLNYRDKVLEYKVELLSRESAYSLFSKNAFGGGPSDKDELCNEIVEKVGRLPLALKTIGSYLHNKELDVWNETLKRLDGVEQDFCDTVLQKSQKNLH

>CSA009605

MEGCVSFRSFSFAITCESLETLVLSNCGLEFFQEFGCLMGYLTELHIDGTFINELSISITNLFSLILLNLRNCIRLPCLPTEIGSLSSLKTLILNGCKNLDKIPSSLGNVKPLEELDIGGTSISIIPFLENLRILNCERLKSNIWHSLASLPANYFSSRRDLNLSDCNLVDEDIPNDLKLFSSLEILDLSSNHFEKLSESIEQLINLKAFYLNDCPELKRVPKLPKSTKYVEGEKSLGTRSEMSPSQSDARLLKSTKYVEGEKSKGSTKEVHITKDMGKQTNHKLVLAHKTSLVGMENQVEKACNLLDLERSKNILFVGIFGSSGIGKTTIAEVVYNTIVDEFQSGYFLYLSSKQNSSVPLQHQMLSHLQSKETKIWDEDHGAQLIKHHMSNRKVVIYCS

>CSA009607

MLAGSPDWFGAGSRIIITTTNKNIFHHPNFKDKVQEYNVELLSHEAAFSLFCKLAFGDHPHTQNMDDLCNEMIEKVGRLPLALEKIAFSLYGQNIDVWEHTLKNFHQVVYDNIFSDVLKSSYEGLEAESQQIFLDLACFLNGEKVDRVIQILQGFGYTSPQTNLQLLVDRCLIDILDGHIQMHILILCMGQEIVHRELGNCQQTRIWLRDDARRLFHENNELKYIRGIVMDLEEEEELVLKAKAFADMSELRILRINNVQLSEDIECLSNKLTLLNWPGYPSKYLPSTFQPPSLLELHLPGSNVERLWNGTQNFKNLKEIDASDSKFLVETPNFSEAPKLRRLILRNCGRLNKVHSSINSLHRLILLDMEGCVSFRSFSFPVTCKSLKTLVLSNCGLEFFPEFGCVMGYLTELHIDGTSINKLSPSITNLLGLVLLNLRNCIRLSSLPTEICRLSSLKTLILNGCKNLDKIPPCLRYVKHLEELDIGGTSISTIPFLENLRILNCERLKSNIWHSLAGLAAQYLRSLNDLNLSDCNLVDEDIPNDLELFSSLEILDLSSNHFERLSESIKQLINLKVLYLNDCNKLKQVPKLPKSIKYVGGEKSLGMLRTSQGKVPLYAPGQRCHLHHLVIIRSHVLNMQYPSFQEV

>CSA009608

MVLAGSSSHASHLPFNVFIGFNRHEEDNKYKSSTRDLYKILCIQGMKVFMDDDGGGGGGSKKKVLMDEDNVSDKIVKAIETYSDSNSSTSSMASSTPKELSSFSSSPRFIFDVFLSFRGVDTRKNVTNRLYEALRRQGIIVFRDDDELERGKTIANTLTNSINQSRCTIVILSKRYADSKWCLRELVEIVKCKNTFKQLVLVVFYKIKPSDVNSPTGIFEKFFVDFENDVKENFEEVQDWRKAMEVVGGLPPWPVNEQTETEKVQKIVKHACDLLRPDLLSHDENLVGMNLRLKKMNMLMGIGLDDKRFIGIWGMGGIGKTTIAKAVFKSVAREFHGSCILENVKKTLKNVGGLVSLQEKLLSDTLMRGKVQIKDGDGVEMIKKNLGNQKVFVVLDGVDHFSQVKDLAGGEEWFGCGSRIIITTRDEGLLLSLGVDIRYNVESFDDEEALQLFCHEAFGVKFPKKGYLDLCMPFIEYAEGLPLAIKALGHSLHNRLFKSWEGAIRKLNNSLNRQVYENLKISYDALGKEERRIFLYIACFLKGQNKDQVIDTFVSFEIDAADGLLTRKNAADVLCIKETAADALKKLQEKSLITMLYDKIEMHNLHQKLGQEIFHEESSRKGSRLWHREDMNHALRHKQGVEAIETIVLDSKEHGESHLNAKFFSAMTGLKVLRVHNVFLSGVLEYLSNKLRLLSWHGYPFRNLPSDFKPSELLELNLQNSCIENIWRETEKLDKLKVINLSNSKFLLKTPDLSTVPNLERLVLNGCTRLQELHQSVGTLKHLIFLDLKDCKSLKSICSNISLESLKILILSGCSRLENFPEIVGNMKLVKELHLDGTAIRKLHVSIGKLTSLVLLDLRYCKNLRTLPNAIGCLTSIEHLALGGCSKLDKIPDSLGNISCLKKLDVSGTSISHIPFTLRLLKNLEVLNCEGLSRKLCYSLFLLWSTPRNNNSHSFGLWLITCLTNFSSVKVLNFSDCKLVDGDIPDDLSCLSSLHFLDLSRNLFTNLPHSLSQLINLRCLVLDNCSRLRSLPKFPVSLLYVLARDCVSLKEHYNYNKEDRGPMSQAEVRVLSYPSSAKDQNSKISQLMISSMCTACENGG

>CSA009775

MQSSSSSSLDRPKMNYDVFISFRGRDVRHTFAGYLYDALNRLGIKAFLDNKRFLIGDDLHDLFKIIDESRSAIVVLSEDYASAKWCLRELTKIMDSMGTSMERVLPVFYHIDPSIVKDQSGTFKTSFDEHEANVLKEIDNQEKEKRLKELQNWKSALKKIGNHTGVVITKNSSEVDIVNKIASQIFDAWRPKLEALNKNLVGMTSRLLHMNMHLGLGLDDVRFVAIVGMGGIGKTTIAQVVFDCILSKFEDCCFLTLPGGDSKQSLVSLQREMLSQIFHKEDFRIWHENHGVEMIKNRLSGRKVLIVLDGIEERRQLEMLAGSIEWFGPGSRIIITTRNKGLLCHPNYDEMKVYNVEELDHDSALQLFLKHAFGSNHQNNDSFMDLSNEIVEKAKRLPLALRVIGSSLYGKDITVWRETLKRLIKVDERNFFDVLKISYDGLGVESQQVFLDITCFFNGKNEDRVIEILESFGYSPNSEVQLLMQRCLIEVSHKKILVHDLILEMGREIVRKESLTQAEKQSRIWLHEDLYCRFAEKHDLMHIQGIVLSLAKEMEESIELDAESFSEMTKLRILEISNVELDEDIEYLSPLLRIINWLGYPSKSLPPTFQSRYLFELLLPHSHLLRIWDGKKRFPKLKLIDVSNSEHLRVTPDFSGVPNLERLVLCNCVRLCEIHPSINSLNKLILLDLEGCGDLKHFPANIRCKNLQTLKLSGTGLEIFPEIGHMEHLTHLHLDGSNITHFHPSIGYLTGLVFLDLSSCLGLSSLPCEIGNLKSLKTLLLKYCKKLDKIPPSLANAESLETLSISETSITHVPPSIIHCLKNLKTLDCEGLSHGIWKSLLPQFNINQTITTGLGCLKALNLMGCKLMDEDIPEDLHCFSSLETLDLSYNNFTTLPDSLSHLKKLKTLNLNCCTELKDLPKLPESLQYVGGIDCRSMSERYYNKILLIPSSSGHQLYLTFIIPSKDVDVECDMNEFQHSIFTRRSFELNIIEEKPSMIVHDAVDMFHWFGQINEGNWTNIQYEQEFSISKPLNIMYEDVDLSNVCGVFLSTNIEFPENLNHLAIGRFLVSFEIDGKCSGGTMNYEMSQFKAARFFWAAYIPIWMFKDHSVMVQRCCSMKVTISYCCDHIDASKVKIKACGVSSMLSWPNVAEYLAKLFTKRFCSKRNFYTMIRQHNDHQNECRCDELEVRKDDFSSSTFESNDSTFLLRKNLRAILGVMFEEKKRYYMKYFFPHTNIFGWFKNQNKKDKVAVKIPVNIEKDRKWMGLAMFVVFSISEKASCYCFEYEIQTKEKIISTQRHSISTDQVLEYSNQILFVAFEPRYNWYPYDELKSSSSNHVFINFNTNGARMRVEFCGARLVYQQNVEGLIHTIMNCIEESGDELYEYYNQQIVESHLNLINAHWYTISFRRNNSVKNQPSTAASTCTASSLSVEHLLYGSFPHPFFHKSLQERFRSKFDLLLHGDKIPKFFSNQSKGNMTEIKLPQYLEKFRESIGVAVCALVVVDKKRRKLNEIIPERERYTKVVDLICKFKVDSYQIMPEHCHFTSQQKLLSEYASQFLWLSYIPLHGFNINWHYCTQFEIALETSCDELFGVKNCGLHLIHKHERMMIDKMVMESTVPSSTSHKGKEPQIH

>CSA009776

DREGDKYTKEVQEWRSALTKVGELTGVVVTKDSLEAASIDKITEQLSSTLHQQKLVNLDELTELVDIERQLCKMDKLNDLEPNVLEQLVGNPNWFGQGSKIIITTRNRDVLRQPNYKDKMVEYKVEFLDNKSAMTLFCKQAFGSCDQFPSKNFEDFSKEIVERVKGHPQVLRQIGSSLYDKGIEIWKEQLKSLEEDYNNRIFKTLKISFDDLGKTSQEVFLDFA

>CSA009778

MGIGLDDVRLIGIWGMGGIGKTTIARIIYKSVSHLFDGCYFLDNVKETLKKEGIASLQQKLLTGALMKRNIDIPNAEGATLIKRRMSNIKALIILDDVDHLSQLQQLAGGSDWFGSGSRVIVTTREEHLLISHGIKRRYNVEVLKIEEGIQLFSQKAFGEDHPKKGYFDLCSQVVDYAGGLPLAIEVLGSSLRNKPMEDWIDAVKKLWEVRDKEIIEKLKISYYMLEKDDREIFLDIACFFKRKSKKQAIEILESFGFPAVFGLDILKEKSLITTPHEKIQMHDLIQEMGQKIVNEKFPDEPEKRSRLWLREDITRALSHDQGTEAIKGIMMDLDEEGESHLNAKAFFSMTNLRILKLNNVHLSEEIEYLSDQLRFLNWHEKQIPSSEMGMTFIRCPISKEPSESYNIDQPRLSAIHLRTMVQRYIEVLTWQQEKYFFVIPCPNCIGCFDKKKYGFSITACCEPDYISEENPRIGIALGAAFEVQKHEMRNNSNDAKICCEFIVKMETDECPPKSAIVFDGQRDELGSPVGLSVFYIPMKRISSWLNQSCCIDVSIVTDNPFVKIKWCGASILYEQNAGSFIGNIIKDLFGSPGKYHTSIVDHILNRQNRVDVSTLLDGGARYKTSWSNAFQRTIGSFPRLQPSRQPRKVIEDSSTMNTTFEVEENESDDNSIILKRKNLKATLLRTFEELKLYGEYYIFPKKEMPRSFFNFQLEEPEITIKIPPNLHKDKKWMGCAFFVVFSVDENSPKSHSFSYQVDNDEYTMERESVIRLNTELFDDSHQLWMFFEPRGVYPYRLNQWRHLCFTFVCNNPDFKAVLCGARLVYKQDVEGFVNTIVSNVLSLPVELLEFYDQMYVEGMLRNILYHKYDPKHKQWVEEQNSNPHNSQEDSSSCSSNMERSLILQLKESIPSFLQKDSKDRFGNTFDFVIPKRNFPPALLNQLSPENPTGVQLPPSLYTNNDWLGFVVCTLFQINKHPTAILNNVCSISRHELICQFAIEIGLIEPLHTHGITEDRSIWLQERQFVWLYYTPRHTYGEIFRQWSSVWAVIEADTPDLMVSCCGMSLVYKKDAAVIDKILMRAIQ

>CSA009781

MGGIGKTTIARVCYERIRDEFEAHCFLSNVRENYIRTLGNLSCLQTKLLSSMFSLKNNHIMDVEEGTAMINKAIFRKKTLLVLDDVDSSDQIKGLIPDNNSFGNGSRVIITTRNADFLSNEFGVKRIFEMDELKYEEALQLLSLSAFMKTCPKEGYLEHSKKIVKVVGGHPLALKLLGSSLRNKNLSVWNEVIEEVGGGGNIHEKIFKCLKVSYDGLDEREREIFLDVACFFNGKRREVVEEILNGCGFYAKTRIELLIQKSLLTLSYDNKLHMHNLLQEMGRKIVRDKHVRDRLMCHKDIKSVVTEALIQSIFFKSSSKNMVEFPILFSRMHQLRLLNFRNVRLKNKLEYSIPSELRYLKWKGYPLEFLPIDSSEECKLIELHMCHSNLKQFWQQEKNLVELKYIKLNSSQKLSKTPNFANIPNLKRLELEDCTSLVNIHPSIFTAEKLIFLSLKDCINLTNLPSHINIKVLEVLILSGCSKVKKVPEFSGNTNRLLQLHLDGTSISNLPSSIASLSHLTILSLANCKMLIDISNAIEMTSLQSLDVSGCSKLGSRKGKGDNVELGEVNVRETTRRRRNDDCNNIFKEIFLWLCNTPATGIFGIPSLAGLYSLTKLNLKDCNLEVIPQGIECMVSLVELDLSGNNFSHLPTSISRLHNLKRLRINQCKKLVHFPKLPPRILFLTSKDCISLKDFIDISKVDNLYIMKEVNLLNCYQMANNKDFHRLIISSMQKMFFRKGTFNIMIPGSEIPDWFTTRKMGSSVCMEWDPDAPNTNMIRFALCVVIGLSDKSDVCNVSSFTIIASVTGKDRNDTNLKNGDDLLVDGFLVSGMKKLDHIWMFVLPRTGTLLRKISNYKEIKFRFLLQAINNKSS

>CSA010121

MALELVGGAVLGAVVGELFKAILNLGERAISFNPVLKDIRSKLNAIMPLVKQIDELNDYLDYPKEETEKLRGLMDEGKQLLLQCGDVKLGDLNYLKRPSYTQKLRELDTALRSFMDVLMLQMARDQKKNMKMMNQMMEIICRLDNRGGSSKPMDLFVPPCLVPQLREETVGLEKPVKELKVKLLKNGVQMLVVTAPGGCGKTTLALKFCHDKEVKDIFQEKIFVPVSRKPDLKLILKDIIESLRGIQLPDLQSDERAFCYLELWLKQTSVNRPVLIVLDDVWSGQESEVLLDKLFQLPCCKILVTSRFYFPRFSESYYLEPLNHENAVQLFRRAASLDKGISKLPDDETIIGGCKRLPLALKVIGRSLSHKPTSVWKVTGRNLARSGSIFDSDNELLECLQSSLDVLDDNMVTKKSFMDLGSFHEDQRISASTFIDMCTVLYTLDESEAMVTLDELSSRSLVNFVTARKYGYDDDFYEEYSFTQHDILRDLAIHLMNMEPIEQRKRLILDINGNDLPKWWVDQEKHTSYARLISITTDKRFSASWPDMEAPEVEVLILNLQSRTYNLPGFIKRMNKLKVLIITYFGSFLTEVTSEDNQLLDSLTSLERIRFERISVPIFSNPNPKPLINLQKISFFMCKFGQTFMDPSTPISDLLPNLLEISIDFCNNLSEVPNRLCEIVSLQKLSITNCHGLSSLPEDVGKLINLKNLRLRSCIHLEEFPESTTKLRELVLLDISNCIGLAKLPEKIGEFHNLEKLDMRHCWSLSKLPLSIGKLKNVKFLCDREVGEWLRKVAPRLAKQVKVQEEEANLEWLGF

>CSA012256

MAEAILFNLTADIIFKLGSSALRQFGSLRGGVKDDFDKLWHSLSAIQAVLHDAEEKQFKDHAVEVWVSRLKDVLYEIDDLIDEFSYQILRRQVLQSNRKQVRTLFSKFITNWKIGHKIKEISQRLQNINEDKIQFSFCKHVIERRDDDDEGLRKRRETHSFILEDEVIGRNDDKEAVINLLLNSNTKEDIAIVSIVGMPGFGKTALAQFIYNHKRIMTQFQLKIWVCVSDEFDLKITIQKIIESATGKKPKSLLQMDPLQCELRKQIDGKKYLIVMDDVWNEKKEKWLHLKRLLMGGAKGSRILITTRSEQVAKTFDSTFVHLLQILDASNSWLLFQKMIGLEEHSNNQEIELDQKNSNLIQIGMEIVSTLRGVPLLIRTIGGLLKDNKSERFWLSFKNKELYQVLGRGQDALKEIQLFLELSYKYLPSSNLKQCFLYCALFPKDYRIKKDELILLWRAQGFIQQNGNNDDNSSLVDIGEDYFMELLSRSFFQEVEKNDFGDIITCKMHDLMHDLACSITNNECVRGLKGNVIDKRTHHLSFEKVSHEDQLMGSLSKATHLRTLFIQDVCSRCNLEETFHNIFQLRTLHLNLYSPTKFAKTWKFISKLKHLRYLHLKNSFCVTYLPDSILELYNLETFIFQSSLLKKLPSNVGNLINLKHLDLSSHLNLEFLPDSITKLYKLEALILHGCSNLKELPKYTKRLINLKSLVLYGCSALTHMPKGLSEMTNLQTLTTFVLGKNIGGELKELEGLTKLRGGLSIKHLESCTSIVDQQMKSKLLQLKSGLQKLELQWKKPKIGDDQLEDVMYESVLDCLQPHSNLKEIRIDGYGGVNLCNWVSSNKSLGCLVTIYLYRCKRLRHLFRLDQFPNLKYLTLQNLPNIEYMIVDNDDSVSSSTIFPCLKKFTISKMPKLVSWCKDSTSTKSPTVIFPHLSSLMIRGPCRLHMLKYWHAPKLKLLQISDSEDELNVVPLKIYENLTSLFLHNLSRVEYLPECWQHYMTSLQLLYLSKCENLKSLPGWIGNLTSLTGLKISTCDKLTMLPEEIDNLTSLTNLDISYCKNLAFLPEGIKHIHNLRSIAVIGCPILEEWCKKNRREDWPKIEYYISRLSHLIKNSFITS

>CSA012262

MVDAASSPTTRRWTYDVFLSFRGEDTRPKFTKHLYQALDAAGVNTFRDDVELRQGDAVRSELVVAIKKSRIAVVVFSSGYADSQWCLGEIAEIMDCRTADGQLVLPIFYEVDPSDVRKQMGRFAAAFEKVLRWRAALTEAASLSGWDLRQLADGHEGKFIQKIVERVQSELRVTYLEVAIYPVGIDLRLKHLISLMAISTNHSTLVLGIYGMSGIGKTTLSKALFNHFFHFFNSRSFLPNINSLSTSSPDGLLRLQQTLLSDLLIATNLRSRSSTTTDSTVVRMQERLQNKKVLVVLDDLDRIEQANALAIRDRRWFGDGSRIIITTRNKQILDTLKVDEVYNMESNLLNDEESLELFSYHAFREQNPPEELLECSKSIVSYCGSLPLALEILGGSFFGGRPMEEWRSAMERLKRIPAWDLQEKLRIGFEGLRDEMEREIFLDVCCYFVGMKEELVVKIMDGCGMYGESGLRGLKWRCLVGVEFWSGRLKMHDLVRDMGREIVRQTCVKEPARRSRVWLYHEALKILLHQNGSENIEGLAIDMGKGNNKEKFRLEAFGKMRNLRLLKLNYVHLIGSNFEHIISKELRWICWHGFPLKSIPSSFYQGNLVAIDMRYSSLIHPWTWRDSQILENLKVLNLSHSEKLKKSPNFTKLPNLEQLKLKNCTALSSLHPSIGQLCKLHLINLQNCTNLSSLPTSIYNLHSLQTFIISGCSKIDCLHDDLGHLESLTTLLADRTAISHIPFSIVKLKKLTDLSLCGCNCRSGSGSSASLPWRLVSWALPRPNQTCTALTLPSSLQGLSSLTELSLQNCNLESLPIDIGSLSELKKLNLGGNKNLRVLGTELCGLLKLNELNVENCGRLEFIQEFPKNMRSFCATSCKSLVRTPDVSMFERAPNMILTNCCALLEVCGLDKLECSTNIRMAGCSNLSTDFRMSLLEKWSGDGLGSLCVAGNQLPKCLHFFTTHPPLTFQVPNINNNILLGLTIFAIFTHLITDINHSPSLRIINRTSSRTHIYRMLGLHYDSLNIHAHHIWAIHLPFSYGYCLNPGDDIELHIPNANAYGVRLVYHLDEPQPIVSFAPSMVVEEQGGASNDHDNDDSTYHVVDQQMQESGSNTTSPWLLRVVD

>CSA012269

MALRQRGINVFIDNKISRGEEISASLLEAIEGSKISIVIISENYASSRWCLNELVKIIMCNKLRGQVVLPIFYKVDPSEVRKQSGKFGEEFAKLEVRFSSEKMQAWREAMISVSHMSGWPVPKKDDEANLIQRIVQEVWKKLNRGTREMRVPKYPVGIDRQVNNILSQVMSDEIITMVGLYGIGGIGKTTLARALYNKIVDDFESCCFLANVREASNQYRGLVELQKELLREILMDDSIKVSNLDIGISIIRDRLCSRKILLILDDVDTSEQLEALAGGHDWFGPGSVVIATTRNKHLLAINEFDILQSVQGLNDVEAFELFSWHAFKMSCPSSHYLYLISKRAVSYCKGLPLALEVVGSFLYSIEPSKLKLILDEYENQYLDKGIQDPLRISYDGLEDEVKEIFLYISCCFVGEDINKVKMKLEACGCLCLEKGTTKLMNLSLLTIDKSNRVEMHNLIQHMGRTIHLLKTSTSHKRKRLLIKDDAMDVLNGNKEAKGVKAIKLSFPKATELDIDSRAFEKVKNVVVLEVGNVTSSKGTDLEYLPSSLRWMNWPHFPFPSLPTTYTMENLMELKLPYSSIKHFGRGFMSGERLKEIDLSGSEFLVEIADLSTATNLEKLNLLGNVKGFEQFPPHLKLKSLKLLSMKNCRIDEWCPQFSEEMKSSLEELLIQYSTVINQLSPTIGYLTSLKRLFIIECMKLKTLPSTIYRLRNLTFLSVIKSDLSTFPSLNNPSSPSLFPYLTSLHLSNCKITNLDFLETMVHVAPTLERLDLSRNNICRLPSCIINFKFLKSLVTMECKLLEEIPKVPKGVVYMNAIGCISLTRFPDNIPDFICCDDNVVRIIVLSHHLMTS

>CSA012278

MELCAGAIVNPIAEKIANCTVDPVFRQLDYLLHFKTNVNDLKDQGKKLVETRDFVQHSVDSAKTNGRATKLAVAVDKAIQGGSFERVGFRVTPQEIMTLRNNKKFEAFESRVLILKEIIEAVGDANARVIVVHGMAGVGKTTLVEEIARLAKEGKLFDAIAMVTVKHIPNIKKIQGEIADQLGLKFEEEKERIRADRLRRRLEMEKKVLVVLDDVWSRLDLEAVGISSHHKGCKILVTSRKDDLFFNDFGTQKNIYINILSKKEARDFFNKVACDSVESSDDTDPEMEAVATELADECGGLPLSLATVGQALKGKGLPSWNDALQGMKFPGEPSNYGVNKVAYLSLKVSYRSLNREEARSLFLLCSLFPEDYQINIKYLLMYAMGLGLLNAMSSLAMAKWRILSLVDELKTSHLLLDGVDNDFVKMHDIVRDTAILIASKMKSKYLVRHGAGESLWPPMDEFKDYTAISLGCSDHSELPEFICPQLRFLLLVGKRTSLRLPEKFFAGMQELRVLDLTGLCIQRLPPSIDQLVNLQTLCLDDCVLPDMSVVGELKKLEILSLRASDIIALPRVIGELTNLKMLNLSDCSKLKVIPANLLSRLIGLSELYMDNSFKHWNVGQMEGYVNARISELDNLPRLTTLHVHIPNPTILPHAFVFRKLSGYRILIGDRWDWSGNYETSRTLKLKLDSSIQREDAIQALLENIEDLYLDELESVKNILFSLDYKGFPKLKGLRVKNNGEIVTVVNSDNMHHPHSAFPLLESLFLKNLAELGSICRGKLPQMSFRNLKRVKVESCDRLKFVFPSSMVRGLIHLQSLEISECGIIETIVSKNKETEMQINGDKWDENMIEFPELRSLILQHLPALMGFYCHDCITVPSTKVDSRQTVFTIEPSFHPLLSQQVSFPKLETLKLHALNSGKIWQDQLPSSFYGFKNLTSLSVEGCASIKYLMTITVARSLVNLERLELNDCKLMKAIIISEDQDLDNNYPSKSILQNKDVFANLESLLISRMDALETLWVNEAASGSFTKLKKVDIRNCKKLETIFPNYMLNRVTNLERLNVTDCSSLVEIFQVKVPVNNGNQVRDIGANHLKELKLLRLPKLKHIWSSDPHNFLRYPSLQLVHTIHCQSLLNLFPVSIAKDLIQLEVLKIQFCGVEEIVAKRGDDGDGDDAASFLLSGLTSLTLWNLFEFKRFYPGKYTLDCPSLTALDVRHCKSFKLMEGTLENSSSISSAVEKVEVEQSSLRGEFERRESKETSTGKEEITTIVQGVVDAELIELRAQLRALVAGQNQMMERLAQLTTIPREPVSK

>CSA012551

MGSSLVGLAQSSSSCSSNLKWSYDVFLSFRGEDTRNNFTSHLDRALREKGVNFFIDDKLERGGQISESLLKSIDGSKISIIIFSKNYASSTWCLDELVKIVQCMKSMGHIVFPVFYKVDPSEVRKQTGGFGEALAKHEANELMTNKVQPWKEALTTAASLSGWDLATRKNEADLIHDLVKEVLSILNQTQLLHVAKHPVGIDSQLRAVEELASHDVPDGVNMVGIHGMGGIGKTTLAKALYNKIAYQFEACCFLSNVRETLEQFKDLVQLQEKLLSEILKDNAWKVGNVHKGKNIIRDRLCSKKVLIILDDVDKDEQLDALVGERDWFGRGSKIIATTRDRHLLENHSFDIVYPIQLLDPKKSLELFSLHAFKQNHPSSNYVDLSKFAVSYCKGLPLALVILGSLLHKRERKIWKSKLHELENSLEPSVEAVFQIGFKELHERVKEIFLDISCFFVGEDINYSKDVLKACDLNPDYGIIILMDLSLVTVEDGKIQMHDLIQQMGQTIVRHESFEPAKRSRLWEAEGAIKILKEKSGTKAVKAIKLDLHYKPWLKIVEAEAFRNMKNLRLLILQRVAYFPKNIFEYLPNSLKWIEWSTFYVNQSSSISFSVKGRLVGLVMKGVVNKQPRIAFENCKTMKHVDLSYCGTLKETPNFSATLNLEKLYLRGCTSLKVIHESVASLSKLVTLDLEGCDNLEKFPSSYLMLKSLEVLNLSRCRKIEEIPDLSASSNLKELYLRECDRLRIIHDSIGRSLDKLIILDLEGCKNLERLPIYTNKLESLELLNLASCLKLETFFDSSFRKFPSHLKFKSLKVLNLRNCLNLEEIIDFSMASNLEILDLNTCFSLRIIHESIGSLDKLITLQLDLCHNLEKLPSSLKLKSLDSLSFTNCYKLEQLPEFDENMKSLRVMNLNGTAIRVLPSSIGYLIGLENLNLNDCANLTALPNEIHWLKSLEELHLRGCSKLDMFPPRSSLNFSQESSYFKLTVLDLKNCNISNSDFLETLSNVCTSLEKLNLSGNTFSCLPSLQNFKSLRFLELRNCKFLQNIIKLPHHLARVNASGSELLAIRPDCIADMMFGKQDAEFSDSTKVLFITNNEIPKYCNKQTTRSSMSVRFRHNLDKNIPALVLCVIFKADGDSCDEAEGFIHFEVSIDGLRGIVKKSLRFSRRFKSSQGELLILEDQAIDRILGLREVVTQRSRNLNLGEEMDVGCSIGFEPRKTLGGYLYVHIGA

>CSA012579

MKIRETSSFILKGEVVGRDDDKKAIIDFLLDTKTKKDNVEVVSIVGMRGLENTAFAQSICKDEKINKHFQLKLRVCILEEFDVKTIVEKIIESIEEKEPKSLQLDKLQSMLREKINEKKYLLIMDDVWNKSHKK

>CSA017609

MADFIWTFALQEILKKTLHLATQQIRLASGFNHDLSKLLHSLLFFEAILRDVDRTKSDRQSVKIWVTKLQDLVLDAEVVLDELSYEDLRREVDVNGNSKKRVRDFFSFSNPLMFRLKMARKIRTITQVLNEIKGEASAVGVIPKGGNDEIVADNGHIPETDSFLDEFEVVGRRADISRIVNVVVDNATHERITVIPIVGMGGLGKTTLAKAVFNHELVIAHFDETIWVCVTATFDEKKILRAILESLTNFPSGLDSKDAILRRLQKELEGKRYFLVLDDVWNENVKLWNNFKSLLLKITNSIGNRVLVTTRSEEAGKIMETFPSHHVEKLSDDECWSIFKERASANGLPLTPELEVIKNVLAEQFGGIPLVAKVLGGAVQFKKRTETWLMSTLETLIMNPLQNENDVSSILRLSVDHLPNSSLKQCFAYFSNFPKGFNFEKEQLIQFWMAEGFIQPSDKVNPETMEDIGDKYFNILLARSLFQDIVKDENGKITHCKMHHLLHDLAYSVSKCEALGSNLNGLVDDVPQIRQLSLIGCEQNVTLPPRRSMEKLRSLFLDRDVFGHKILDFKRLRVLNMSLCEIQNLPTSIGRLKHLRYLDVSNNMIKKLPKSIVKLYKLQTLRLGCFRGEAPKKFIKLISLRHFYMNVKRPTTRHMPSYLGRLVDLQSLPFFVVGTKKGFHIEELGYLRNLRGKLKLYNLELVRNKEEAMRADLVKKDKVYKLKLVWSEKRENNYNHDISVLEGLQPHINLQYLTVEAFMGELFPNLTFVENLVQISLKNCSRCRRIPTFGHLPNLKVLEISGLHNLKCIGTEFYGNEYGEGSLFPKLKRFHLSDMNNLGRWEEAAVPTEVAVFPCLEELKILDCPRLEIAPDYFSTLRTLEIDDVNNPISQITLQTFKLLGIIHSGNLSGLPEELRGNLSSLEEFKVWYYLHLKSFPTIQWLTDILKGKTGYDTKWTNIQSHGLESYTSVNELSIVGHSDLTSTPDIKALYNLSSLTISGLKKLPKGFHCLTCLKSLSIGGFMEGFDFRPLLHLKSLENLAMIDFGLAESTLPDELQHLTGLKHLKIVGFQGIESLPEWLGNLNSLESLHIESCRKLRELPEAMGCLAKLEEVRSFNCPELRVYQDESEWAKISYIPRFISFNYWVDE

>CSA017644

MAGSTGANSSSNSSPAPPPPKILLAKPGLVPGGPINSKIGRGAGADDEPASIRSRLPSLGSLNLLSDSWDLHIDRFLPFLTENTEFKVVGIIGPPGVGKSTIMNEIYGYDGSSPGMLPPFPILSEDVRAMARHCTLGIEPRISSERIILLDTQPVFSPSVLAEIMRPDGSSTVSVINGESPSAELAHELMSIQLGILLASICNIVLVISEGVHDLNMWHLMLTVDLLKHGLPDPSSPISSHAQNSNVASEKEYKEKTSTSEEYMATPIFVHAK

>CSA018159

MADFLWTFAVEETLKKVLKVAGEQTGLAWGFQEHLSNLQKWLLKAEAFLRDINMRKLHLDSVRMWVDDLQHLVYQADDLLDEIVYEDLRQKVQTRKMKKVCDFFSPSTNVLIFRLNMAKKMMTLIALLEKHYLEAAPLGLVGNENASPEFDVIGQYRETISELEDHKIVGRDVEVESIVKHVIDASNNQLTSILPIVGMGGLGKTTLAKLVFNHELVRQHFDKTVWVCVSEPFIVNKILLDILQNLKGTISNGGDSKEVLLRELQKKMHGQRYFLVLDDVWNENSFLWDELKYCLLKITGNSKNSIVVTTRSAEVAKIMGTCSGHLLSKLSDDHCWSLFKESANAYGLSMTSNLEIIQKELVKKIGGIPLAARVLGRAVKFEGDVERWEEMLKNVLSTPLKEENFILSILKLSVDRLPSSALKQCFSYCSIFPKDFVFEKQELIHMWMAQGFLQPQEGRNMTMETVGDIYFKILLSHCLFEDAHETKTEEYEIPDLLEFETRPEEYKMHDLVHDIAIEISRDQNLQLNPSNISKKELQKEIKKVACKLPMVDFIRRIPCNIGQLTFFDVEIRNFVCLRVLKLSTLPSDKLPKSIGQLKHLRYLEIACYLVEDELSNNSVTQISEQLQHLTALEFLSIENFGGIEALPEWLGNFVCLQTLSLYNCKNLKKLPSTKAMLRLTKLNQLYACKCPMLLLEEGDPERAKLSHFPNMLVQRNGYQKCI

>CSA018210

MDILVSVIAATIKPIGHQLGYLVCYNRNKKELREQLENLETTKKDVNQRVEEAKGKSYTISEEVSKWLADVDNAITHDELSNSNPSCFNLAQRYQLSRKREKQVNYILQLMNKRNSFVEVGYRAPLPDTENTVVPGDYQVLESKTLLAKDIKNALSKPEVNKIGVYGMAGVGKTYFLNEVKKLVLKGEDRLFDRVIDVRVGRFNDVTDIQEQIGDQLNVELPKSKEGRASFLRNNLAKMEGNILILLDDLWKEYDLLKEIGIPLSKDGCKVLITSRSQDILTNNMNTQECFQVSSLSEEESWKFFMAIIGDKFDTIYKKNIAKNVAKECGGLPLALDTIAKALKGKDMHHWEDALTKLRNSIGMDIKGVALKSFGRGIGVDICHQLL

>CSA019939

MAESILCSLAGSIITKLGSFALQDLGLLWGFHDELDKLKGTVSALEAVLLDAEEKQSKSRAVKDWILKLKDTFYDIDDLLDVFSYESLKRQVMTKHRTNNTKKVRIFFSKSNQIAFRLKMSQKIKRVREKLDAIAMDKTQFNLYENTREIQDDESTKRLETTSFIREGEIIGRDDDKKSIIHYLLDTNIHEDSVAVIAIIGMGGLGKTALVQSIYGDEKVKKHFELTMWVCISEEFDVKVIIEKIIESLTKKKREPDLQLDTLQSMVREKIDGKRYLLVMDDVWNVNRAKWISLKRYLMGGAKGSRILITTRTHQLFNCEKLQQLPRFDRFPFLKHLHLEDLPSIEYIAINNYVSSSMTTFFPSLENLSIIKLPNLKEWWKGESIDQNTSFPTILRHLSQLKIHYCRQLASIPQHGPLQSLDIRDISLQLFELVIKMTATNIIVGEDSSSSANDMFIRSSSSLKIWKIDWEFLPNDLFSNVTHLQSLVIGRCFNLKMSFDDDNVRWKELGSLRTLRLCFIPKLEYLPKGFQYLKALEHLELLWCENLACILGIEHLTSLSRLEISNCPNLTSLPEGMTQLISLTCLIIDDCPNLSTLPEGLHHLLNTPSLSANIATKLGSFSLLELGLLWIGFHEELDKLKDTLFAIQVVLLDAEHKQYKSYAVKE

>CSA020328
[truncated: 1,066 more chars]
