# Supplementary figures and images for: Genome-wide analysis of NBS-encoding disease resistance genes in Cucumis sativus and phylogenetic study of NBS-encoding genes in Cucurbitaceae crops
Source: BMC Genomics. 2013 Feb 19;14:109. doi: 10.1186/1471-2164-14-109 (PMC3599390; doi:10.1186/1471-2164-14-109)

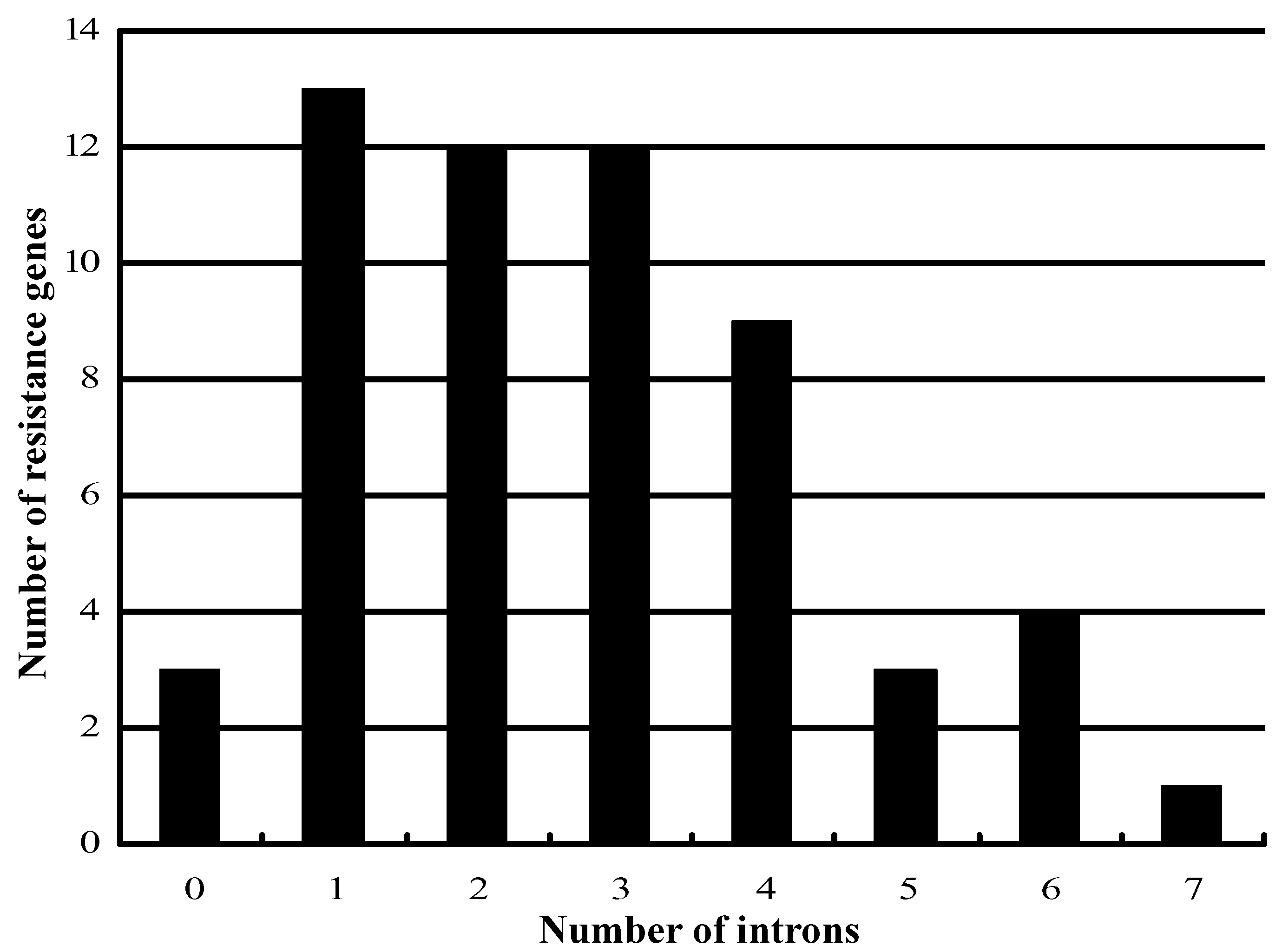

Supplement: Additional file 4 — Distribution of the NBS-encoding genes with different numbers of introns in cucumber. [file 1471-2164-14-109-S4.tiff]
