# Supplementary material for: Genome-wide analysis of NBS-encoding disease resistance genes in Cucumis sativus and phylogenetic study of NBS-encoding genes in Cucurbitaceae crops
Source: BMC Genomics. 2013 Feb 19;14:109. doi: 10.1186/1471-2164-14-109 (PMC3599390; doi:10.1186/1471-2164-14-109)
Supplement: Additional file 6 — (A) Motif sequence logos in the cucumber CC-NBS family of NBS-encoding genes. (B) Motif sequence logos in the cucumber TIR-NBS family of NBS-encoding genes. [file 1471-2164-14-109-S6.doc]

Additional file 6A The motif sequence Logos in the cucumber CC-NBS family of R proteins

**C1**

**C2**


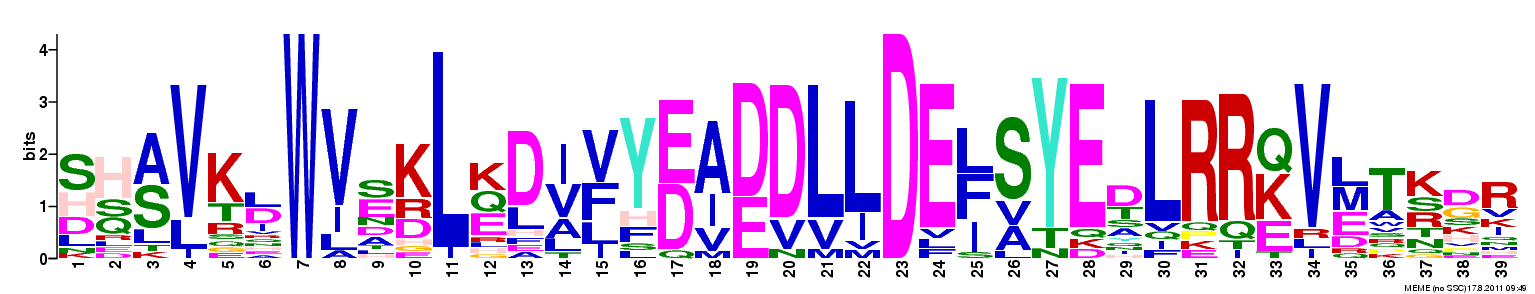

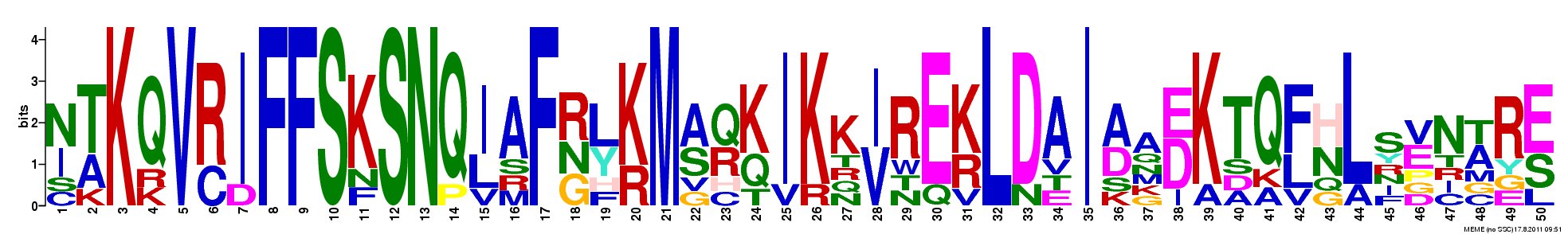


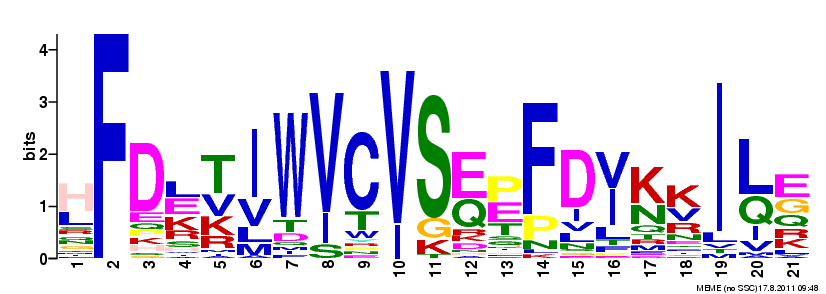

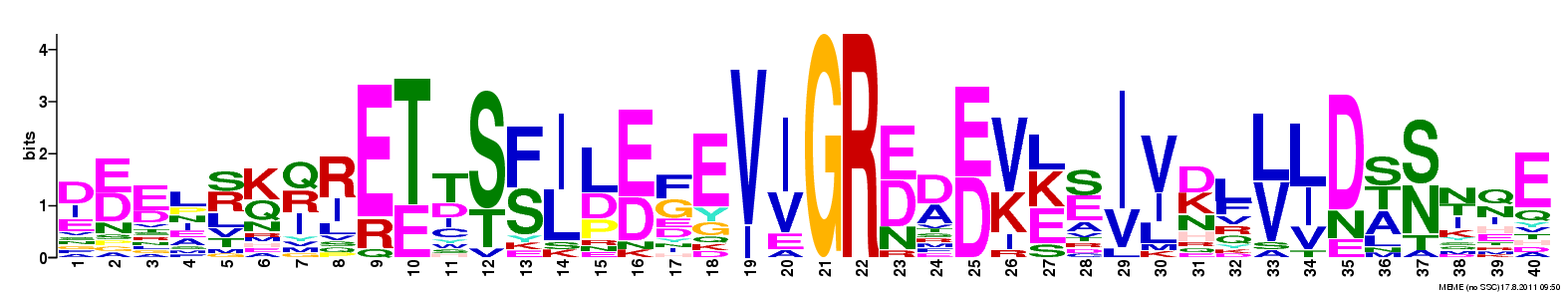


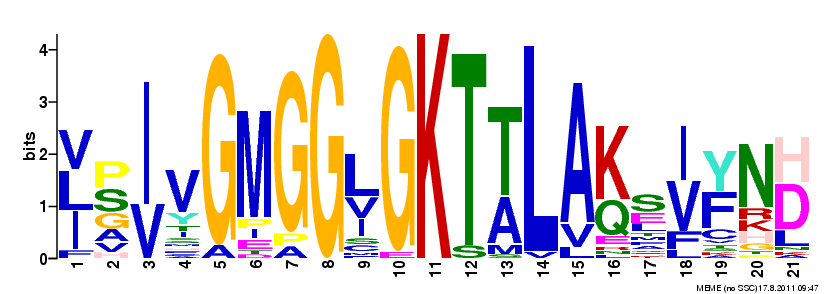


**P-loop**

**C3**

**RNBS-A-nonTIR**


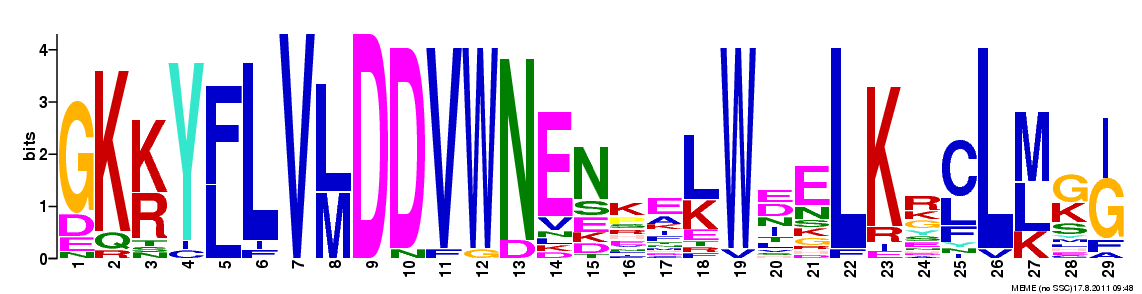


**Kinase-2**

**RNBS-B**

**RNBS-C**


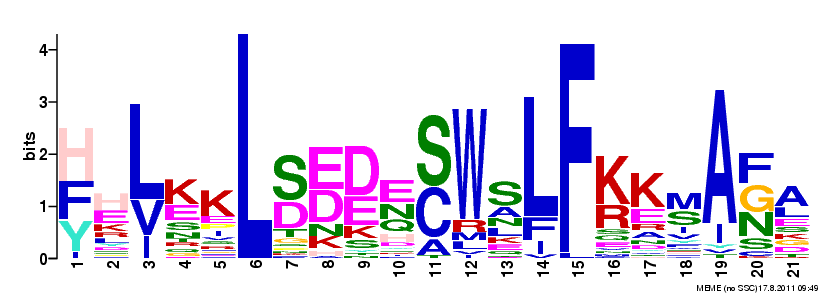

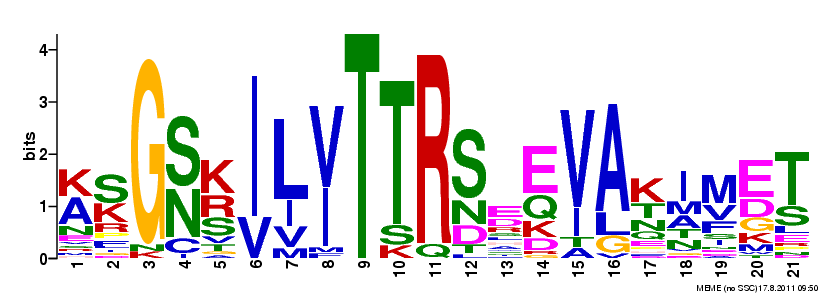


**RNBS-D-nonTIR**


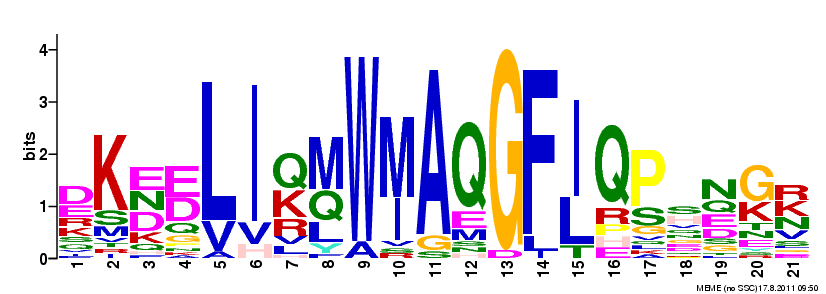

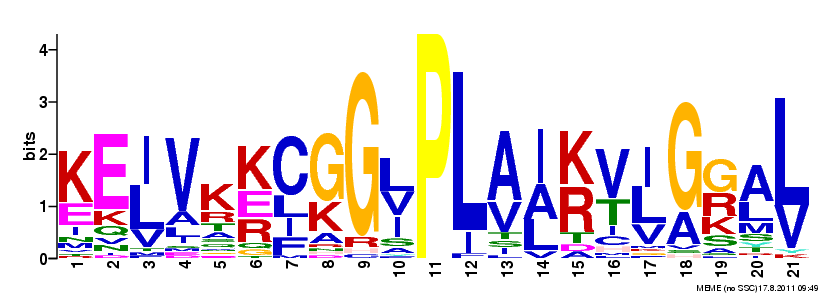

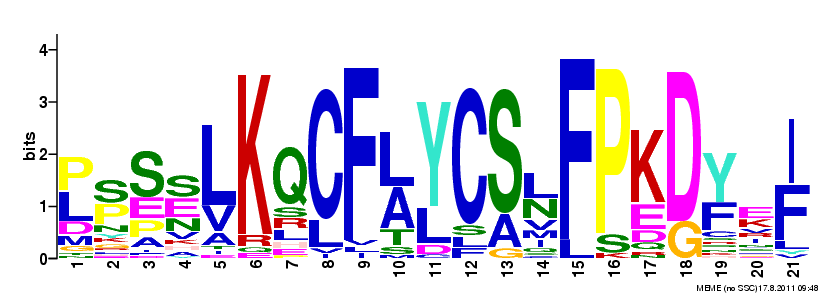


**GLPL**

**CNBS-1**

**MHDV**

**CNBS-2**

**L1**


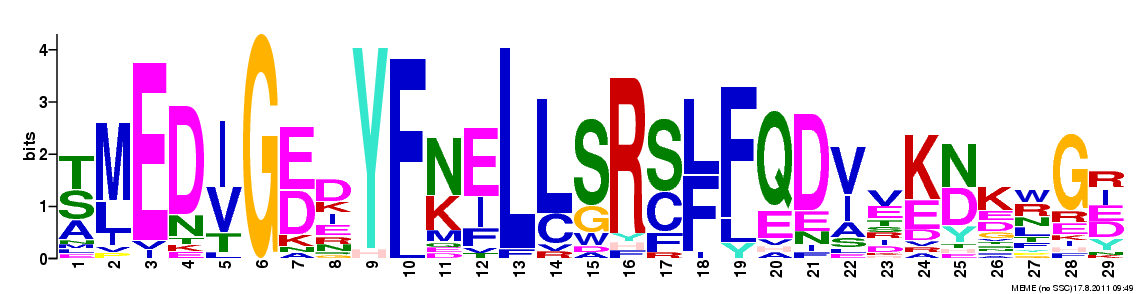

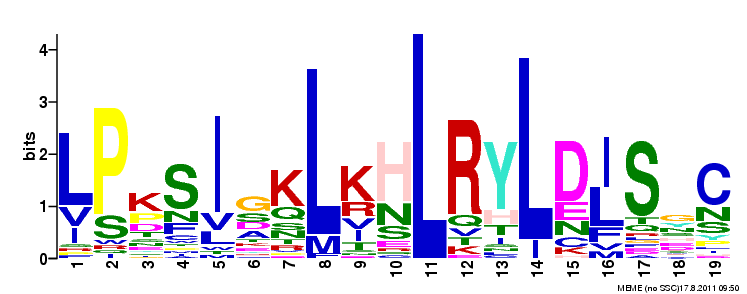

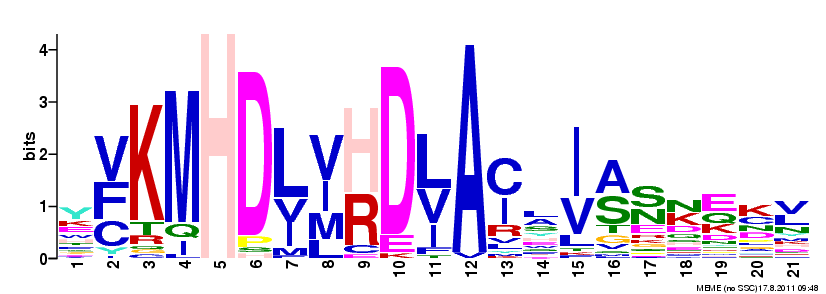


**L4**

**L3**

**L2**


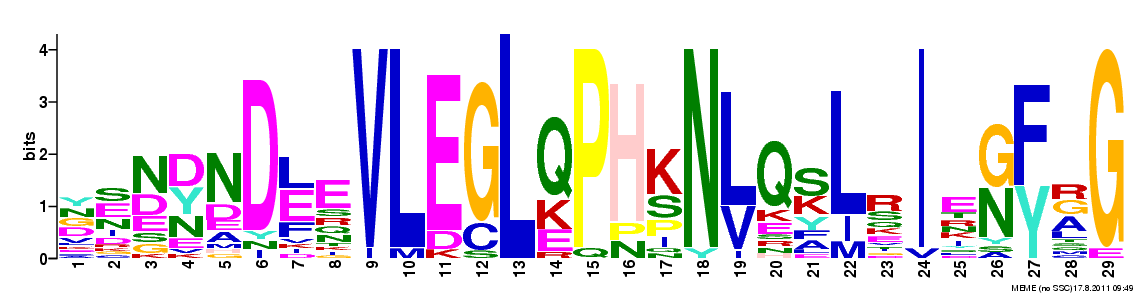

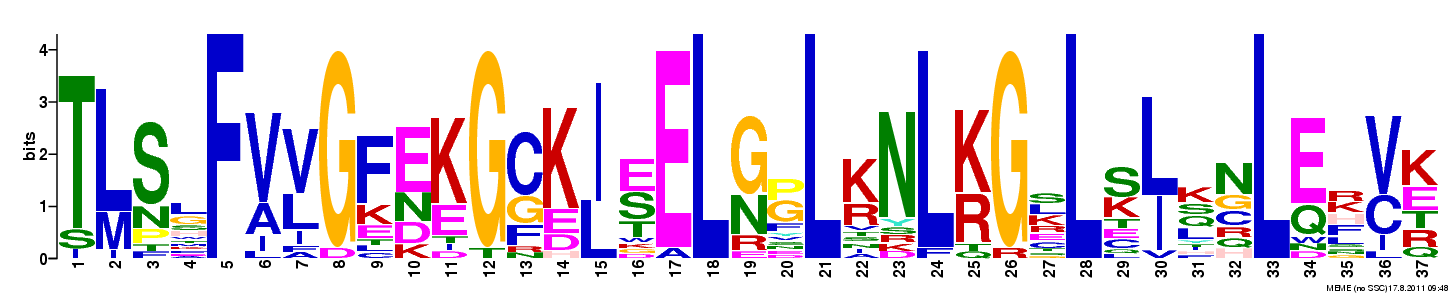

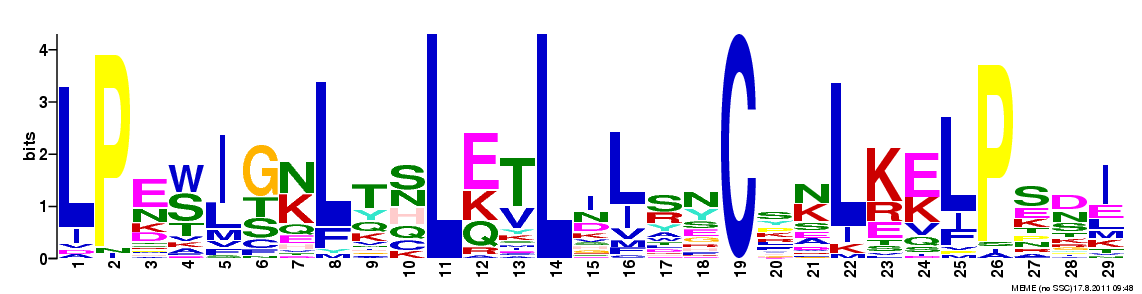


**L5**

**L6**

**L7**


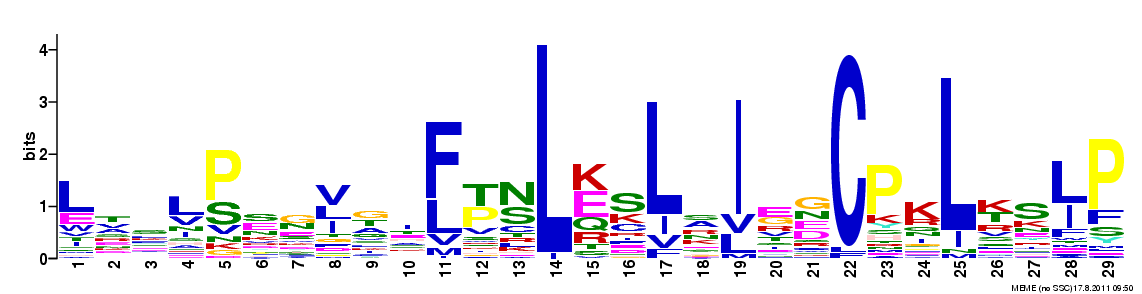

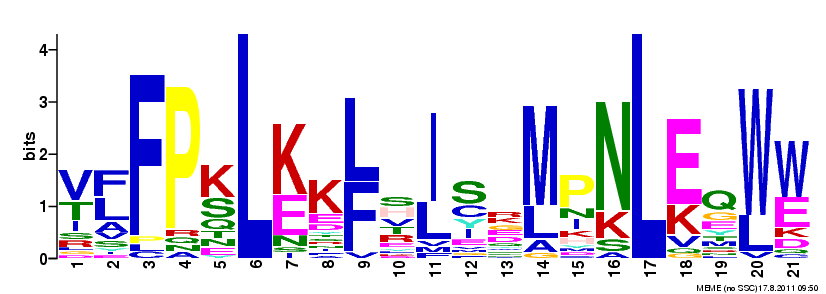

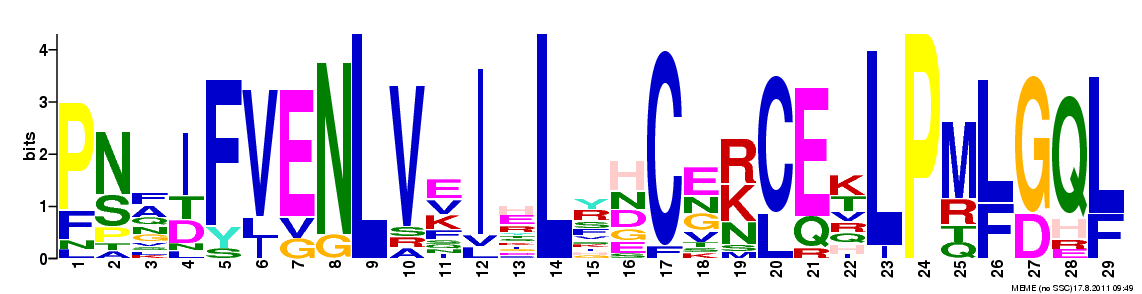


Additional file 6B The motif sequence Logos in the cucumber TIR-NBS family of R proteins


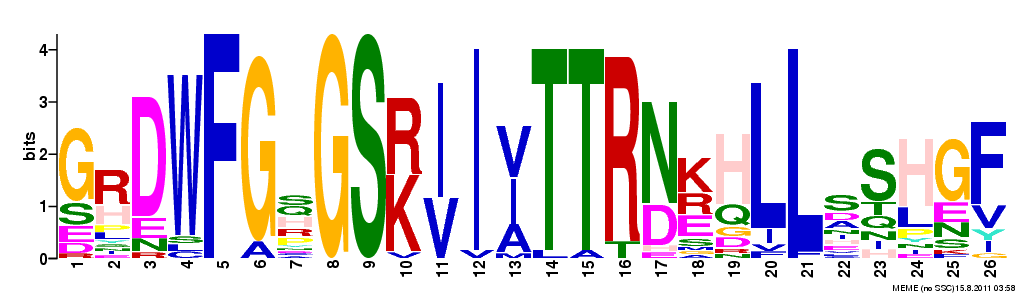

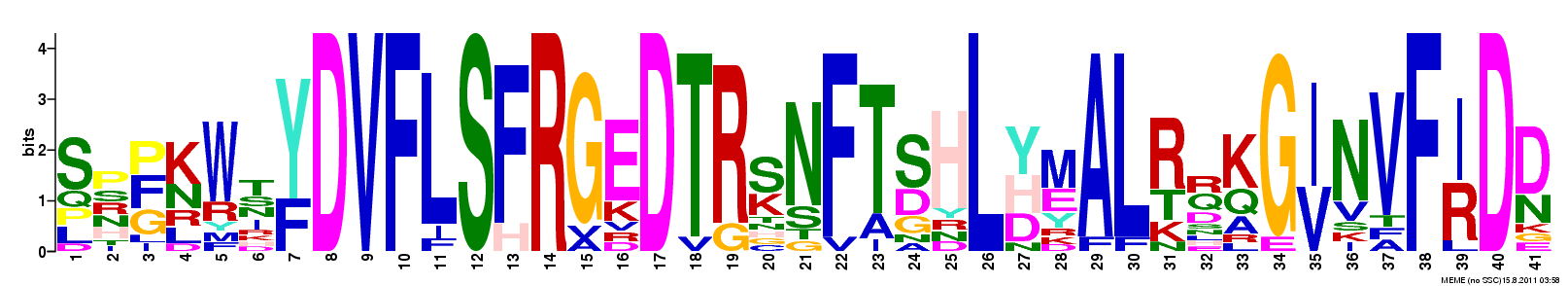

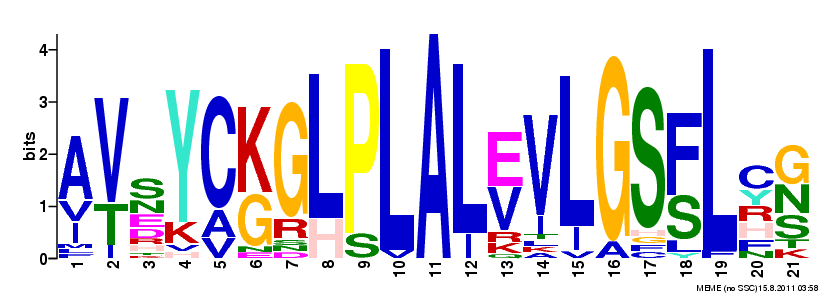

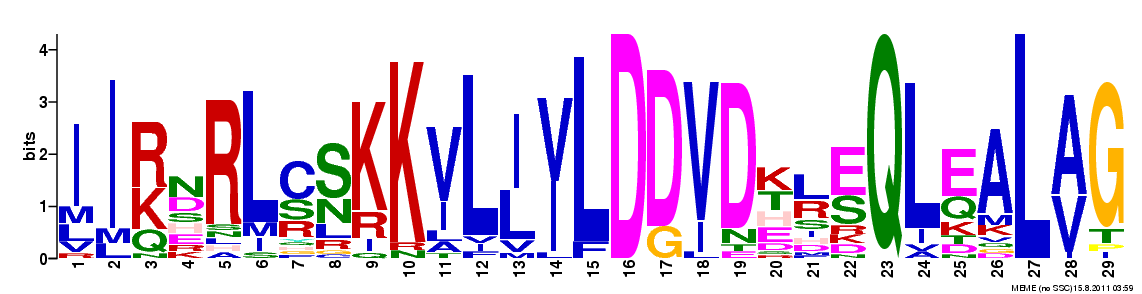

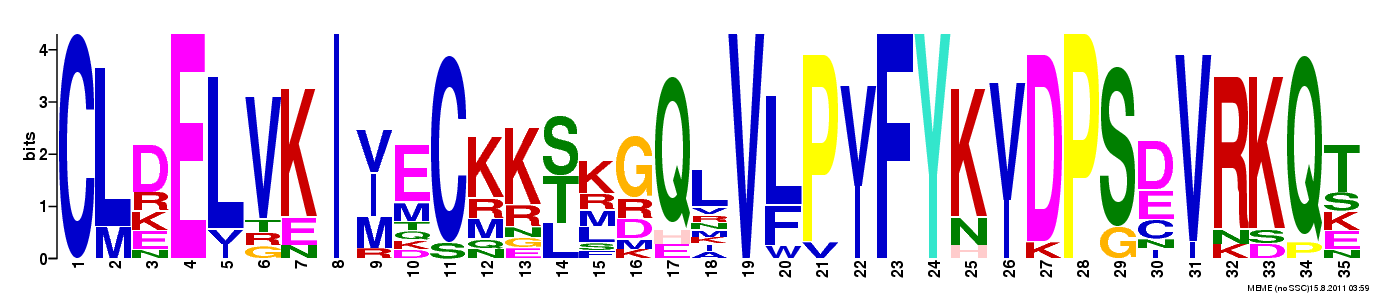

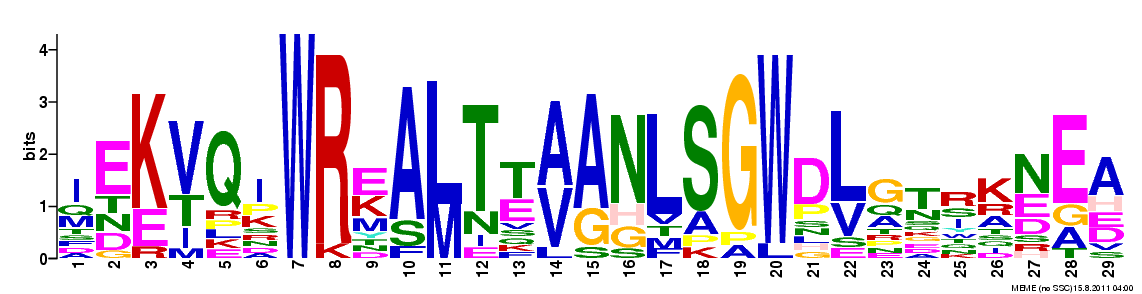

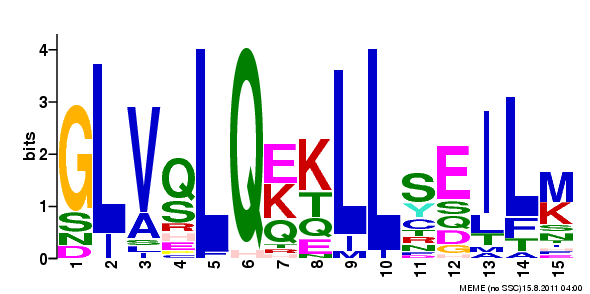

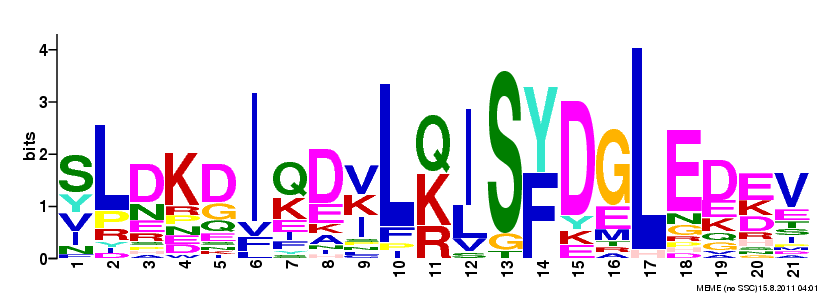

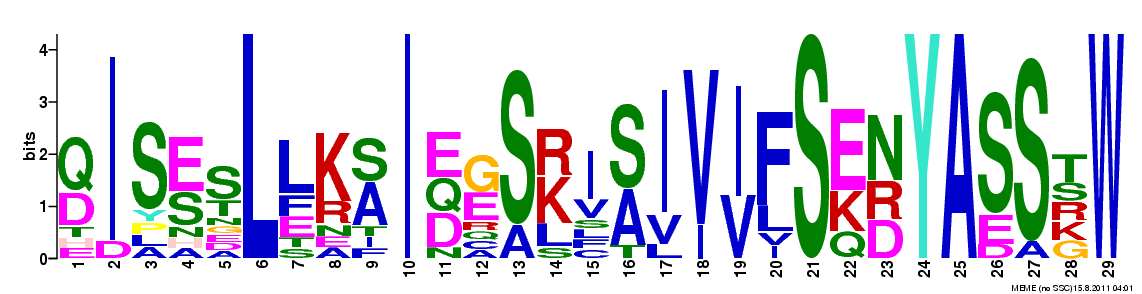


**T1**

**T2**

**T3**

**T4**


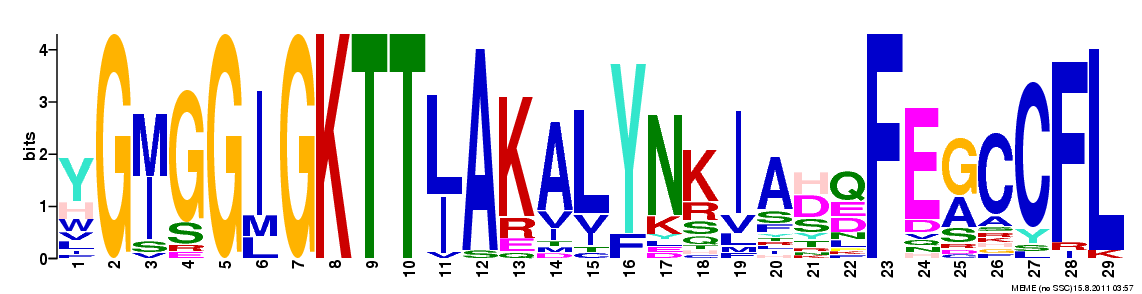


**P-loop**

**RNBS-A-TIR**

**RNBS-B**

**Kinase-2**


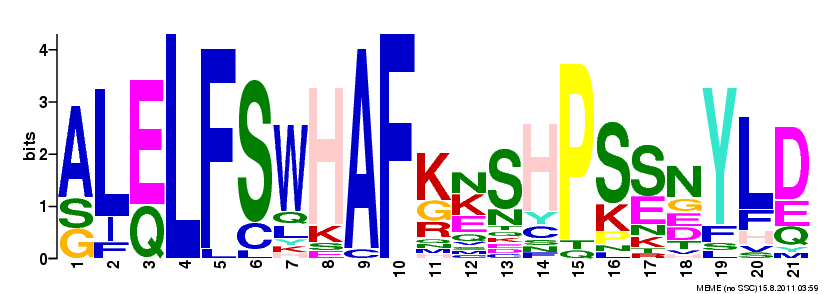


**RNBS-C**

**GLPL**


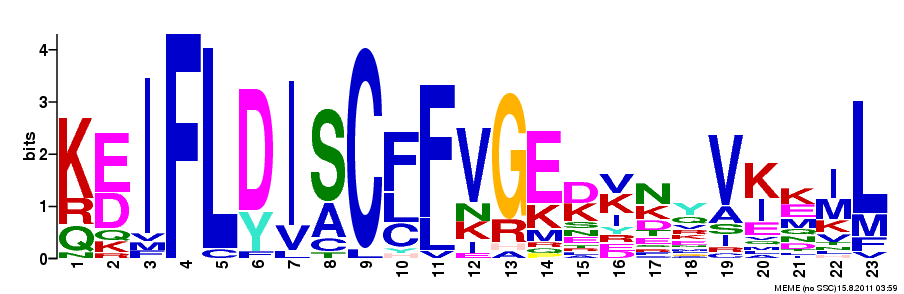


**RNBS-D-TIR**


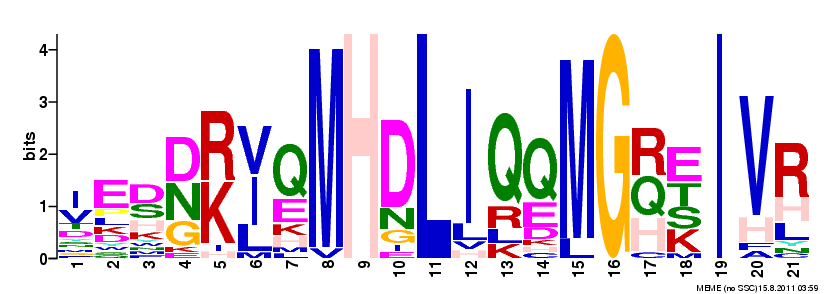


**MHDL**


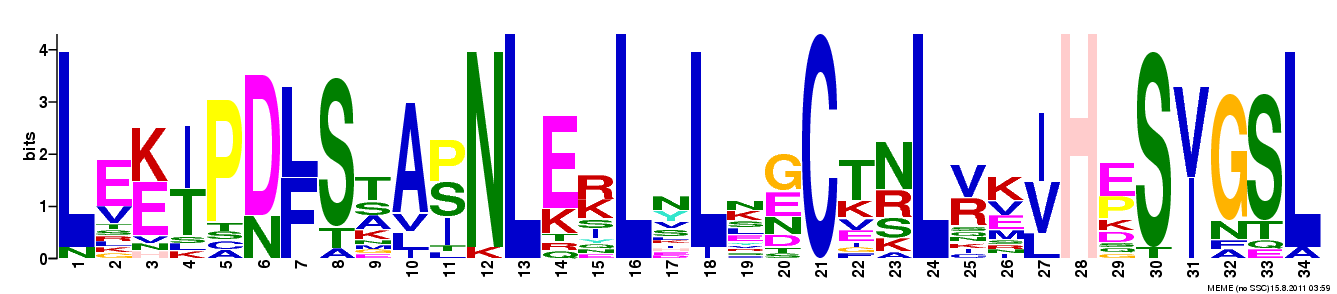


**L3**


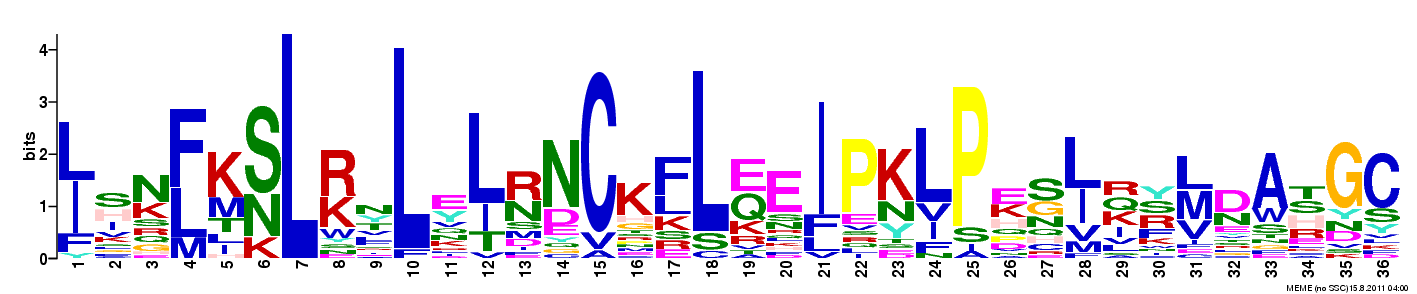


**L7**


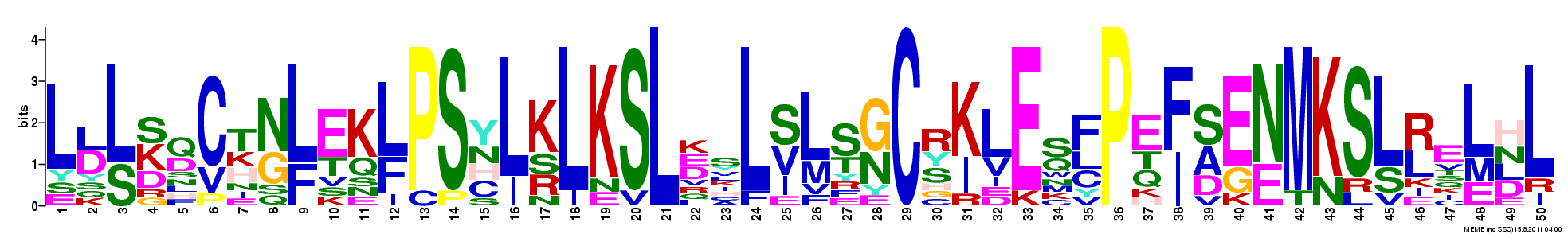


**L4**

**TNBS-I**


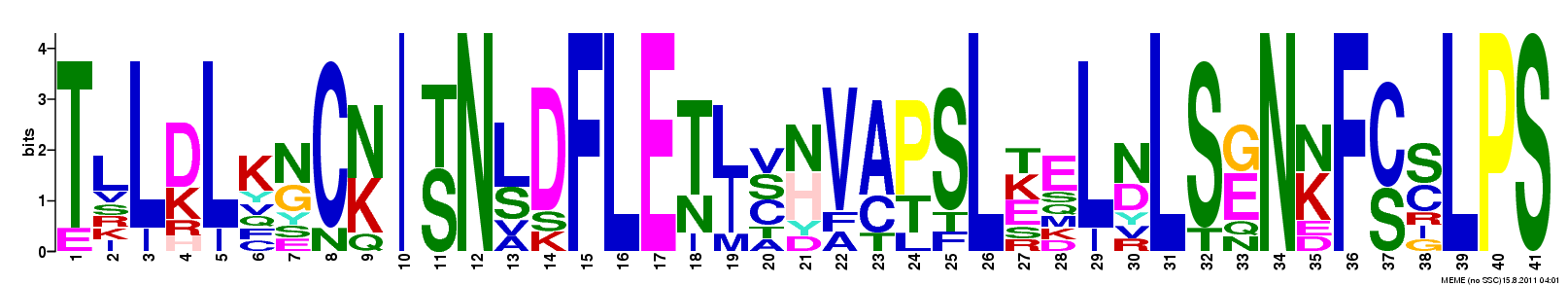


**L6**


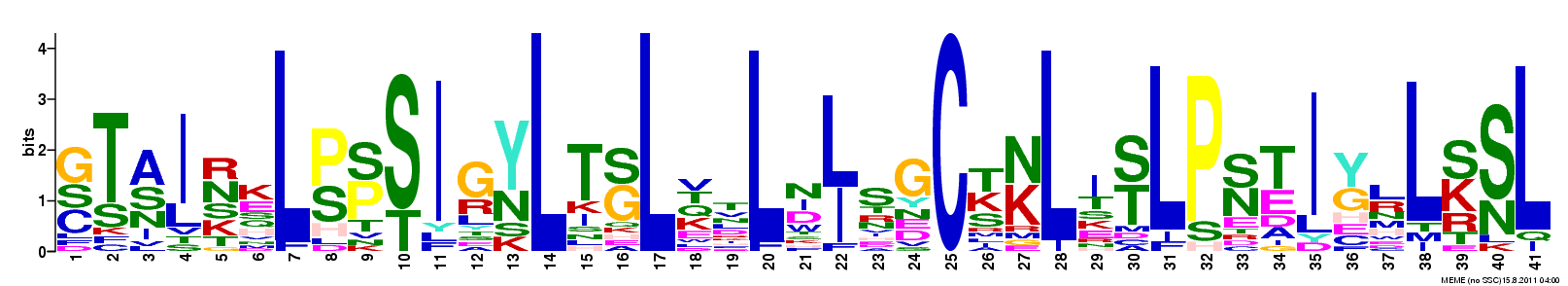


**L5**


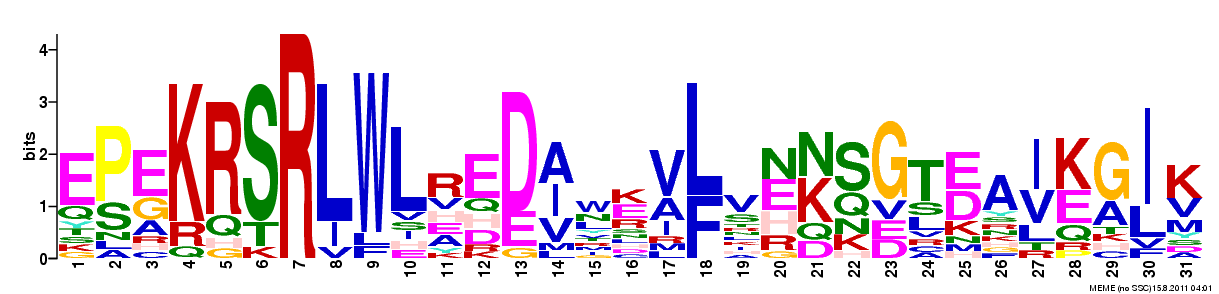


**L1**


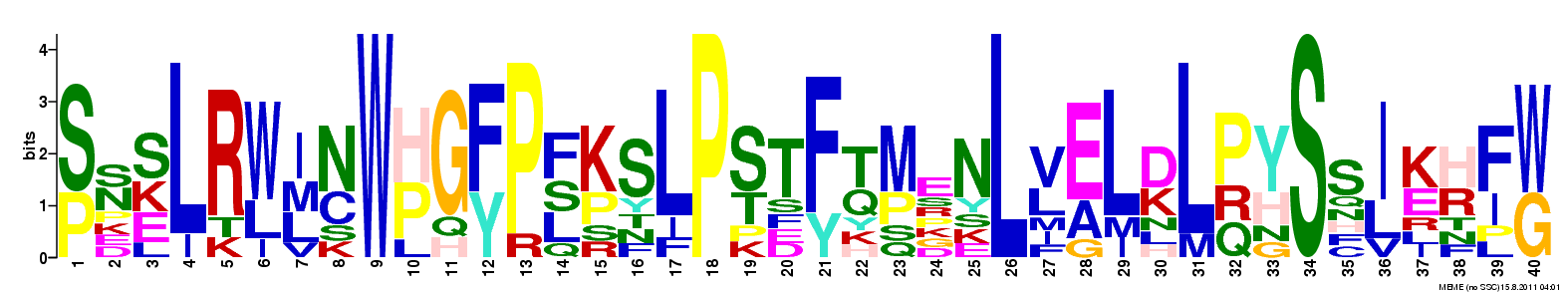


**L2**
