# Supplementary material for: Genome-wide analysis of NBS-encoding disease resistance genes in Cucumis sativus and phylogenetic study of NBS-encoding genes in Cucurbitaceae crops
Source: BMC Genomics. 2013 Feb 19;14:109. doi: 10.1186/1471-2164-14-109 (PMC3599390; doi:10.1186/1471-2164-14-109)
Supplement: Additional file 7 — PCR amplification products generated by the three selected resistance gene degenerate primers in the four major Cucurbitaceae crops. [file 1471-2164-14-109-S7.doc]

**Additional file 7** PCR amplification products generated by the three selected resistance gene degenerate primers in four major *Cucurbitaceae* crops. *Lanes 1*, *4*, *7* and *10* amplificons generated by primer combinations of F1 and R1; *Lanes 2*, 5, *8* and *11* amplificons generated by primer combinations of F2 and R2; *Lanes 3*, 6, *9* and *12* amplificons generated by primer combinations of F3 and R3. M: marker 2000


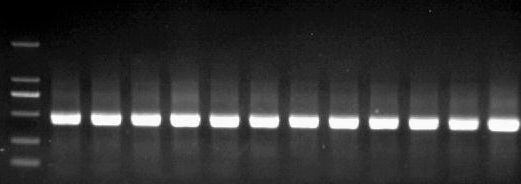


**M**

500bp

**Melon**

**Bottle gourd**

**Luffa**

**Watermelon**

**1**

**2**

**3**

**4**

**5**

**6**

**7**

**8**

**9**

**10**

**11**

**12**
