# Supplementary material for: Genome-wide analysis of NBS-encoding disease resistance genes in Cucumis sativus and phylogenetic study of NBS-encoding genes in Cucurbitaceae crops
Source: BMC Genomics. 2013 Feb 19;14:109. doi: 10.1186/1471-2164-14-109 (PMC3599390; doi:10.1186/1471-2164-14-109)
Supplement: Additional file 8 — Phylogenetic comparison of Cucurbitaceae NBS-encoding genes and RGHs. The TIR- and CC-NBS families are distinct. The former is divided into subfamilies TIR1 to TIR 9 and the latter is separated into subfamilies CC1 to CC4. [file 1471-2164-14-109-S8.doc]

**Additional file 8**

Cucumber

Melon

Squash

Watermelon

Bottle gourd

Luffa

TIR1

TIR2

TIR3

TIR9

TIR8

TIR7

TIR6

TIR5

TIR4

TIR-NBS

CC1

CC2

CC3

CC4

CC-NBS
