# Supplementary material for: Genome-wide analysis of NBS-encoding disease resistance genes in Cucumis sativus and phylogenetic study of NBS-encoding genes in Cucurbitaceae crops
Source: BMC Genomics. 2013 Feb 19;14:109. doi: 10.1186/1471-2164-14-109 (PMC3599390; doi:10.1186/1471-2164-14-109)
Supplement: Additional file 9 — The detailed phylogenetic tree from Figure 4. The tree consists of 63 Cucurbitaceae and 52 Arabidopsis CC-NBS protein sequences. Parentheses indicate the ancient CC family (N1 to N4) as defined by Cannon et al. [41]. [file 1471-2164-14-109-S9.doc]

**黄瓜**

**N3**

**N2**

**N1**

**N4**

**Additional file 9**

**甜瓜**

**葫芦**

**西瓜**

**丝瓜**

**CC4**

**南瓜**

Cucumber

Melon

Squash

Watermelon

Bottle gourd

Luffa

**CC1**

**CC2**

**CC3**
