# Supplementary material for: Genome-wide analysis of NBS-encoding disease resistance genes in Cucumis sativus and phylogenetic study of NBS-encoding genes in Cucurbitaceae crops
Source: BMC Genomics. 2013 Feb 19;14:109. doi: 10.1186/1471-2164-14-109 (PMC3599390; doi:10.1186/1471-2164-14-109)
Supplement: Additional file 10 — The detailed phylogenetic tree from Figure 5. The tree consists of 152 Cucurbitaceae and 106 Arabidopsis TIR-NBS sequences. Arabidopsis subfamilies are indicated by At-TIR-NBS-A to At-TIR-NBS-G corresponding to the seven TIR subfamilies identified by Richly et al. [52]. [file 1471-2164-14-109-S10.doc]

Cucumber

Melon

Squash

Watermelon

Bottle gourd

Arabidopsis

**Additional file 10**
