# Supplementary material for: Genome-wide analysis of NBS-encoding disease resistance genes in Cucumis sativus and phylogenetic study of NBS-encoding genes in Cucurbitaceae crops
Source: BMC Genomics. 2013 Feb 19;14:109. doi: 10.1186/1471-2164-14-109 (PMC3599390; doi:10.1186/1471-2164-14-109)
Supplement: Additional file 11 — Total number of predicted NBS-encoding genes identified in the six sequenced angiosperm genomes. The values for Carica papaya, Arabidopsis thaliana, Vitis vinifera, Oryza sativa, and Populus trichocarpa were previously summarized by Yang et al. [37] and Porter et al. [48]. The total number of predicted protein encoding genes and the genome size of each species is also shown. [file 1471-2164-14-109-S11.doc]

**Additional file 11** The total number of predicted NBS-encoding genes identified in the six sequenced angiosperm genomes. Value of *Carica papaya*, *Arabidopsis thaliana*, *Vitis vinifera*, *Oryza sativa*, and *Populus trichocarpa* were previously summarized by Yang et al. 2008 and Porter et al. 2009. The total number of predicted protein encoding genes and the genome size of each species is also provided.

| Plant species | Total number of predicted protein encoding genes | Total number of predicted NBS-encoding genes | Genome size (Mb) | Reference |
| --- | --- | --- | --- | --- |
| *Cucumis sativus* | 26,682 | 57 | 367 | In this study |
| *Carica papaya* | 24,746 | 54 | 372 | Ming et al. (2008) |
| *Arabidopsis thaliana* | 25,498 | 174 | 125 | Arabidopsis Genome Initiative (2000) |
| *Vitis vinifera* | 30,434 | 535 | 487 | Jaillon et al. (2007) |
| *Oryza sativa* | 37,544 | 519 | 389 | International Rice Genome Sequencing Project (2005) |
| *Populus trichocarpa* | 45,555 | 416 | 485 | Tuskan et al. (2006) |

**Reference**

Arabidopsis Genome Initiative (2000) Analysis of the genome sequence of the flowering plant *Arabidopsis thaliana*. Nature 408:796–815

Ming R, Hou S, Feng Y, Yu Q, Dionne-Laporte A, Saw JH et al (2008) The draft genome of the transgenic tropical fruit tree papaya (Carica papaya Linnaeus). Nature 452:991–996

Jaillon O, Aury JM, Noel B, Policriti A, Clepet C et al (2007) The grapevine genome sequence suggests ancestral hexaploidization in major angiosperm phyla. Nature 449:463–467

International Rice Genome Sequencing Project (2005) The map-based sequence of the rice genome. Nature 436:793–800

Tuskan GA, Difazio S, Jansson S, Bohlmann J, Grigoriev I et al (2006) The genome of black cottonwood, *Populus trichocarpa* (Torr. & Gray). Science 313:1596–1604
