# Supplementary material for: Genome-wide analysis of NBS-encoding disease resistance genes in Cucumis sativus and phylogenetic study of NBS-encoding genes in Cucurbitaceae crops
Source: BMC Genomics. 2013 Feb 19;14:109. doi: 10.1186/1471-2164-14-109 (PMC3599390; doi:10.1186/1471-2164-14-109)
Supplement: Additional file 12 — Amino acid sequence alignment of NBS-encoding genes. The conserved domains are highlighted and indicated by an arrow. The alignment was constructed using Clustal X software. [file 1471-2164-14-109-S12.rtf]

Additional file 12A

                           10          20         30          40         50          60          70         80          90         100        110         120       130                     
                 ....|....|....|....|....|....|....|....|....|....|....|....|....|....|....|....|....|....|....|....|....|....|....|....|....|....|
Cucsa.237410  MERRASIKSLSPPPYSISLPLPPLRNYDVFLSHRVKDTGSSFAADLHEALTNQGIVVFRDGIDDEDAEQPYVEEKMKAVEESRSSIVVFSENYG-SFVCMKEVGKIVTCKELMDQLVLPIFYKIDPGNVR 
Cucsa.237440  -MTSLSFPPPPPPPYSISLPLPPLRRYDVFLSHRAKDTGCSFTSNLHEALTSQGIVVF---IDKEDGGKP-LTEKMKAVDESRSSIVVFTKNYG-SLVCMKEIRKIRMCQKLRDQLVLPVFYKIDPGDVR 
Cucsa.237540  -----------MQSSSSSSLDRPKMNYDVFISFRGRDVRHTFAGYLYDALNRLGIKAFLD--NKRFLIGDDLHDLFKIIDESRSAIVVLSEDYASAKWCLRELTKIMDSMGTSMERVLPVFYHIDPSIVK 
Cucsa.237560  -------MASSTPKELSSFSSSPRFIFDVFLSFRGVDTRKNVTNRLYEALRRQGIIVFRD-DDELERGKTIANTLTNSINQSRCTIVILSKRYADSKWCLRELVEIVKCKNTFKQLVLVVFYKIKPSDVN 
Cucsa.237520  -------------------------------------------------------------------------------------------MCRPPYWCWDDS---------------WMFFDVGYTKFK 
Cucsa.237530  ---------------------------------------------------------------------------------------------------------------------------------- 
Cucsa.237390  MVPSSPSSSAASSSSPSSSPSIGKWKFDVFLSFRGEDTRGGFTDHLYKALTRKGISTFRD-ENEIEEGEHIPSNLLASIDASRFAIVVVSEDYASSRWCLEELARMFECK----KEVLPIFYKVDPSHVK 
Cucsa.338650  -------------MADELRPQHGNWTYDVFLSFRGEDTRKNFTDHLYYAFKDAGINVFRD-DPELERGEDISSELERAIEGSKVAVVVFSERYAESGWCLEELVKIMECRRTLRQLVFPIFYNVDPSCVR 
Cucsa.338660  -------------MANEFQAQHGDWTYDVFLSFRGEDTRKNFTDHLYYALKDAGINVFRD-DPELQRGEDISSGLERAIEGSKVAVIVFSERYAESGWCLEELVKIMECRRTLRQMVLPVFYNVDPSCVR 
Cucsa.155730  ---------------------------------------------------------------------------------------------------------------------------------- 
Cucsa.091690  ---------------------------------------------------------------------------------------------------------------------------------- 
Cucsa.178450  ----------MNQASGSSSSSRFRWHFDVFLSFRGEDTRSNFTSHLNMALRQRGINVFID--NKLSRGEEISASLLEAIEGSKISIVIISENYASSRWCLNELVKIIMCNKLRGQVVLPIFYKVDPSEVR 
Cucsa.091680  ---------------------------------------------------------------------------------------------------------------------------------- 
Cucsa.091710  ---------------------------------------------------------------------------------------------------------------------------------- 
Cucsa.275630  -------------QSSSSCSSNLKWSYDVFLSFRGEDTRNNFTSHLDRALREKGVNFFID--DKLERGGQISESLLKSIDGSKISIIIFSKNYASSTWCLDELVKIVQCMKSMGHIVFPVFYKVDPSEVR 
Cucsa.292710  ---------------------------------------------------------------------------------------------------------------------------------- 
Cucsa.091840  -----MGSNAAGAE-SSSSSP-INWIYDVFLSFRGEDTRSNFTSHLHMFLRHKGVNVFID--DRIERGEQISEALLKTIQCSLISIVIFSENYASSTWCLDELVEIIECKKSKGQKVLPIFYKVDPSDVR 
Cucsa.091470  ----------MDSSTVATESPTFKWTYDVFLSFRGEDTRTNFTSHLDMALRQKGVNVFID--NKLERGEQISESLFKSIQEASISIVIFSQNYASSSWCLDELVNIIECKKSKGQNVFPVFYKVDPSDIR 
Cucsa.091780  ---------------------------------------------------------------------------------------------------------------------------------- 
Cucsa.091820  -----MGSSIVGAESSTSSSSSFKWSFDVFLSFRGDDTRSNFTGHLDMALRQKGVNVFID--DMLKRGEQISETLSKAIQEALISIVIFSQNYASSSWCLDELVKIVECKKSKGQLVLPIFYKVDPSDVR 
Cucsa.091460  -----MGSSVVGDE-SFSSSPNFNYDYDVFFSFRGEDTRSNFISHLHMALRLKEVNVFID--DKLKRGEQIYESLLKFIERSRLSLVIFSKDYASSTWCLDELVKIIECKKSKGQAVWPVFYKVDPSEVR 
Cucsa.249360  ---------------------------------------------------------------------------------------------------------------------------------- 
Cucsa.089350  ---------------------------------------------------------------------------------------------------------------------------------- 

                          140        150        160         170        180        190         200        210         220        230        240         250       260            
                ....|....|....|....|....|....|....|....|....|....|....|....|....|....|....|....|....|....|....|....|....|....|....|....|....|....|
Cucsa.237410  KQEGNFKKYFNDHEANPKIDIEEVENWRYSMNQVGHLSGWHVQDS--------QSEEGSIINEVVKHIFNKLRPDLFRYDDK-LVGISPRLHQINMLLGIG-----------------------LDDVRF 
Cucsa.237440  KQEGSFEKYFNEHEVNPNISIEEVKKWRKSMNKVGNLSGW--------------SEEG-TINEVVNHIFNKLRPDLFRYDDK-LVGISRRLHEINKLMGIG-----------------------LDDVRL 
Cucsa.237540  DQSGTFKTSFDEHEANVLKEIDNQEKEKRLKELQNWKSALKKIGNHTGVVITKNSSEVDIVNKIASQIFDAWRPKLEALNKN-LVGMTSRLLHMNMHLGLG-----------------------LDDVRF 
Cucsa.237560  SPTGIFEKFFVDFENDVKENFEEVQDWRKAMEVVGGLPPWPVN----------EQTETEKVQKIVKHACDLLRPDLLSHDEN-LVGMNLRLKKMNMLMGIG-----------------------LDDKRF 
Cucsa.237520  IQS--------------------------------------------------------IANSIGDHLL---RLKLQAKEEN-LFEMPLRLRTMKMLLGLG-----------------------SNDVRF 
Cucsa.237530  -----------------------------------------------------------MGKQTDNKLV-------LSHKTS-LVGMENQVKKVCNLLDLER----------------------SKDILF 
Cucsa.237390  NQSGTFEEAFVKHEKRFGRGDGKVQSWRTFLTELANTKAWLSQS---------WSHESNIIEEITTKIWKRLKPNLTVIKEDQLVGINSKINKLSSLLIPNSD-----------------DDDADDDVIF 
Cucsa.338650  KQKGEFEEAFVKHEVRYFRDIDRVLKWRMALTEAANLSGWDLRNIAN-------GHEAKFIRLIVEKVSKEVNSKYLFIALY-PVGIESRLKLLLSHLHIG----------------------SND-VRF 
Cucsa.338660  KQKGEFEEAFVKHEKG--KDIDKVRRWRMALTEAANVAGLGLTQNAN-------GHEAEFIRSIVKMISKEVKSNYLFIALY-PVGIESRIKLVLPHLHIG----------------------SNDDVKF 
Cucsa.155730  -------------------------------------------------------------------------------------------------MAIS----------------------TNHSTLV 
Cucsa.091690  --------------------------------------------------------------------------------------------------------------------------MSN-EITM 
Cucsa.178450  KQSGKFGEEFAKLEVR-FSSEKMQAWREAMISVSHMSGWPVPKKDDEAN---------LIQRIVQEVWKKLNRGTREMRVPKYPVGIDRQVNNILSQV------------------------MSDEIITM 
Cucsa.091680  ---------------------------------------------------------------------------MQLDVAKYPVGIDIQVRNLLPHV------------------------MSNG-ITM 
Cucsa.091710  ---------------------------------------------------------------------------------------------------------------------------------M 
Cucsa.275630  KQTGGFGEALAKHEANELMTNKVQPWKEALTTAASLSGWDLATRKNEAD---------LIHDLVKEVLSILN-QTQLLHVAKHPVGIDSQLRAVEELASHDVPDG----------------------VNM 
Cucsa.292710  ---------------------------------------------------------------------------------------------------------------------------------M 
Cucsa.091840  KQNGCYGEGLAKHEAN--FMEKIPIWRNALTTAANLAGWDLGTIRNEAD---------LIQVIVKEVSSTLN--VTTPSDKPLLVGIDSKIESLYWPT------EEMYKSECVD---------------M 
Cucsa.091470  KQTGSFGEALAKHQPK--FQTKTQIWREALTTAANLSGWNLG-TRKEAD---------LIGDLVKKVLSVLNRTCTPLYVAKYPVGIDSKLEYMKLRS------HNLFEKSNKFHYRKQHEYESDTGVYM 
Cucsa.091780  ---------MAKHQPK--FQTKTQIWRKALTTAANLSGWDLGAYRREAD---------LIRDLVKEVLSTINRTRTPLYVAKYPVGIDSQLEYMKFHS------HHLNKGN-KFQYWTQNEYESDIGVYM 
Cucsa.091820  KQTGCFGEALAKHQAN--FMEKTQIWRDALTTVANFSGWDLG-TRKEAD---------FIQDLVKEVLSRLNCANGQLYVAKYPVGIDSQLEDMKLLS------HQIRDAF--------------DGVYM 
Cucsa.091460  KQTGGFGEALAKHEANKLLANKIQPWREALTFAAGLSGWDLANSKDEAE---------LIQKIVKRVLSAVNP-MQLLHVAKHQVGVDSRLRKIEELV------SHIG-SEGVN---------------L 
Cucsa.249360  ------------------------------------------------------------------------NLKRLLRVAKHLVEIDSQLREIEESV------SHIGSE-------------------- 
Cucsa.089350  --------------------------------------------------------------------------------------------------------------------------MSDKKIVM 

                          270        280        290         300        310         320        330        340         350        360         370        380       390            
                ....|....|....|....|....|....|....|....|....|....|....|....|....|....|....|....|....|....|....|....|....|....|....|....|....|....|
Cucsa.237410  VGIWGMGGIGKTTIARIIYKSVSHLFDGCYFLDNVKEALK-KED-IASLQQKLLTGTLMKRN---IDIPNADGATLIKRRISNIK-ALIILDDVNHLSQLQKLAGG-LDWFGSGSRVIVTTRDE---HLL 
Cucsa.237440  IGIWGMGGIGKTTIARIIYKSVSHLFDGCYFLDNVKETLK-KEG-IASLQQKLLTGALMKRN---IDIPNAEGATLIKRRMSNIK-ALIILDDVDHLSQLQQLAGG-SDWFGSGSRVIVTTREE---HLL 
Cucsa.237540  VAIVGMGGIGKTTIAQVVFDCILSKFEDCCFLTLPGGDS---KQSLVSLQREMLSQIFHKE--DFRIWHENHGVEMIKNRLSGRK-VLIVLDGIEERRQLEMLAGS-IEWFGPGSRIIITTRNKGLLCHP 
Cucsa.237560  IGIWGMGGIGKTTIAKAVFKSVAREFHGSCILENVKKTLK-NVGGLVSLQEKLLSDTLMRGK---VQIKDGDGVEMIKKNLGNQK-VFVVLDGVDHFSQVKDLAGG-EEWFGCGSRIIITTRDE---GLL 
Cucsa.237520  IGIVGMSGIGKTTLAEMTYLRIFKPFVSALRKPYFLHFV---GRSIVSLQQQLLDQLAFLKPIDIQVLDENHGVELIMQHLSSLKNVLIVFDGITERSQLEMLAGS-PDWFGAGSRIIITTTNKNIFHHP 
Cucsa.237530  VGIFGSSGIGKTTIAEVVYNTIIDEFQSGCFLYLSSK-----QNSLVPLQHQILSHLLSKET---KIWDEDHGAQLIKHHMSNRK-VVIVLDGVDERNQIEKLVGS-PNWFAPGSRVIITATNRDVLHQL 
Cucsa.237390  VGIHGMGGIGKTTIARVCYERIRDEFEAHCFLSNVRENYIRTLGNLSCLQTKLLSSMFSLKN--NHIMDVEEGTAMINKAIFRKK-TLLVLDDVDSSDQIKGLIPD-NNSFGNGSRVIITTRNAD--FLS 
Cucsa.338650  VGILGMGGLGKTTVAKALYNQLYHNFEAKCFLSNIK-AETSN---LIHLQKQLLSSITNSTNINLGN---IDQGIAVLQERLRCKRLLLILDDVDDLSQLTALATS-RDLFASGSRIIITTRDRH-LLNQ 
Cucsa.338660  VGILGIGGLGKTTIAKALYNQLHHNFEAACFLANIK-QTPNQPNGLVHLQKQLLSSITNSSNINFEN---MDRGIVVLQESLRRKKLLLILDDVDKISQLTALATR-RECFGSGSRIVITTRHRR-LLNQ 
Cucsa.155730  LGIYGMSGIGKTTLSKALFNHFFHFFNSRSFLPNINSLSTSSPDGLLRLQQTLLSDLLIATNLRSRSSTTTDSTVVRMQERLQNKKVLVVLDDLDRIEQANALAIRDRRWFGDGSRIIITTRNKQ-ILDT 
Cucsa.091690  VGLYGIGGMGKTTLAKALYNKISDDFEGCCFLANVR-EASNQYWGLVELQKTLIREILMDDS---IKVSNVGIGISIIRDRLCSKKIILILDDIDTHEQLQALAGG-HDWFGHGSKVIATTRNKQ---LL 
Cucsa.178450  VGLYGIGGIGKTTLAKALYNKIADDFEGCCFLINVR-EASNQYRGLVELQKELLREILMDDS---IKVSNLDIGISIIRDRLCSRKILLILDDVDTSEQLEALAGG-HDWFGPGSVVIATTRNKH---LL 
Cucsa.091680  FGLYGVGGMGKTTIAKALYNKIADEFEGCCFLSNIR-EASNQYGGLVQFQKELLCEILMDDS---IKVSNLPRGITIIRNRLYSKKILLILDDVDTREQLQALAGG-HDWFGHGSKVIATTRNKQ---LL 
Cucsa.091710  VGLHGIGGMGKTTLAKTLYNRIADDFEGCCFLANIR-EASKQHEGLVRLQEKLLYEILMDDF---IRVSDLYKGINIIRNRLCSKKILLILDDIDTSEQLQVLAGG-YDWFGYGSKVIVTTRNEH---LL 
Cucsa.275630  VGIHGMGGIGKTTLAKALYNKIAYQFEACCFLSNVR-ETLEQFKDLVQLQEKLLSEILKDNA---WKVGNVHKGKNIIRDRLCSKKVLIILDDVDKDEQLDALVGE-RDWFGRGSKIIATTRDRH---LL 
Cucsa.292710  VGIHGMGGIGKTTLAKALYNKITYQFEACCFLSNVR-ETSEQFNGLVQLQEKLLNEIFKDNN---LKVDNVDKGMNIIKDRLCSRKVLMVLDDVDKDDQLDALVGG-RDWFGRGSKIIVTTRDRH---LL 
Cucsa.091840  LGIYGIRGIGKTTLAKALYYKMASQFECCCFLSNVR-EASKQLNGLAQLQKKLLFQILKYDL---EDVDDLDRRNNIIKHRLHSKKVLILLDDVDEMKQLKALAGG-HDWFGQGSKIIVTTRDKH---LL 
Cucsa.091470  VGLYGIGGIGKTTLAKALYNKIASQFEACCFLSNVR-EASKQFNGLAQLQETLLYEILTVDL---KVIN-LDRGINIIRNRLCLKKVLIVLDDVDKLEQLEALVGG-RDWFGQGSRIIVTTRNKH---LL 
Cucsa.091780  VGIYGIGGLGKTTLAKALYNKIASQFEGCCFLSNVR-QASNQFNGLVQLQQNLLYEILEDDL---KFVN-LDKGITIIRNRLRSKKVLIVLDDVDKLEQLEALVGG-RDWFGQGSKIIVTTRNSH---LL 
Cucsa.091820  MGIYGIGGIGKTTLAKALYNKIANQFEGFCFLSNVR-ETSKQFNGLVQLQEKLLYEILKFDL---KIGN-LDEGINIIRSRLRSKKVLIVLDDVDKLKQLEALVGE-RDWFGHGSKIIVTTRNSH---LL 
Cucsa.091460  VGLYGIGGIGKTTLAKALYNKIATQFEGCCFLQDVRREASK--HGLVQLQETLLNEILKEDL---KVIVSRDRGINIIRSRLCSKKVLIVLDDVNDLEQLEALVGG-RDWFGQGSKIIVTTRNEH---LL 
Cucsa.249360  -------GIGKTTLAKDLYNKIATQFERCCFLQDVR-REASKQYGLVQLHETLLCEILKEDL---KVVN-CDKGINIIRSRLCLKKVLIVFDDVDHHRQLEALVGE-LDWFGRGSKIIMLTRNGH---LL 
Cucsa.089350  LGLYGIGGIGKTTLAKALYNRIAHDFEGCCFLEKIR-EASNQYDGLVQLQKKILCDILMDNS---INVSNLDIGVNIIRNRLCSKKILLILDDVDTREQLEALAGG-HDWFGHGSKIIATTRNMQ---LL 


                           400        410        420         430        440         450        460        470        480         490         500        510       520            
                 ....|....|....|....|....|....|....|....|....|....|....|....|....|....|....|....|....|....|....|....|....|....|....|....|....|....|
Cucsa.237410  ISHGIERRYNVEVLKIEEGLQLFSQKAFG-EEHTKEEYFDVCSQVVDYAGGLPLAIEVLG-SSLRNKPMEDWINAVEKLWEVR-DKEII-EKLKISYYML-EKS-EQKIFLDIACFFKRKS--------- 
Cucsa.237440  ISHGIKRRYNVEVLKIEEGIQLFSQKAFG-EDHPKKGYFDLCSQVVDYAGGLPLAIEVLG-SSLRNKPMEDWIDAVKKLWEVR-DKEII-EKLKISYYML-EKD-DREIFLDIACFFKRKS--------- 
Cucsa.237540  NY-DEMKVYNVEELDHDSALQLFLKHAFGSNHQNNDSFMDLSNEIVEKAKRLPLALRVIG-SSLYGKDITVWRETLKRLIKVD-ERNFF-DVLKISYDGL-GVE-SQQVFLDITCFFNGKN--------- 
Cucsa.237560  LSLGVDIRYNVESFDDEEALQLFCHEAFG-VKFPKKGYLDLCMPFIEYAEGLPLAIKALG-HSLHNRLFKSWEGAIRKLNNSL-NRQVY-ENLKISYDAL-GKE-ERRIFLYIACFLKGQN--------- 
Cucsa.237520  NFKDKVQEYNVELLSHEAAFSLFCKLAFG-DHPHTQNMDDLCNEMIEKVGRLPLALEKIA-FSLYGQNIDVWEHTLKNFHQVV-YDNIFSDVLKSSYEGL-EAE-SQQIFLDLACFLNGEK--------- 
Cucsa.237530  NYRDQVQEYKVELLSRESAYSLFCKNAFG-DGPSDK--NDLCSEIVEKVGRLPLALRTIG-SYLHNKDLDVWNETLKRLDEE--EQNYFDTILKRN---------------------------------- 
Cucsa.237390  NEFGVKRIFEMDELKYEEALQLLSLSAFM-KTCPKEGYLEHSKKIVKVVGGHPLALKLLG-SSLRNKNLSVWNEVIEEVGGGGNIHEKIFKCLKVSYDGL-DER-EREIFLDVACFFNGKR--------- 
Cucsa.338650  LEVDEICSID--EMDDDEALELFSWHAFR-NSYPSETFHQLSKQVVTYCGGLPLALEVLG-SFLFGRSREEWEDTLKKLKKIP--NDQIQKKLKISFDGLNDHT-YKDIFLDVSCFFIGME--------- 
Cucsa.338660  IEVDGICSID--VMDDAEALQLFSWHAFH-NSYPSETFHQLSKRVVNYCGGLPLALQVLG-CFLFGRSREEWQDTLKNLKKIL--DDQIQIKPKITFDTHNDHT-CKDIYL------------------- 
Cucsa.155730  LKVDEVYNMESNLLNDEESLELFSYHAFR-EQNPPEELLECSKSIVSYCGSLPLALEILGGSFFGGRPMEEWRSAMERLKRIP--AWDLQEKLRIGFEGLRDEM-EREIFLDVCCYFVGMK--------- 
Cucsa.091690  ASHGFNILKRVNGLNAIEGLELFSWHAFKNSHPSSDYLDVS-KRAVHYCKGLPLALEVLG-SFLNSIDDQSKFERILDEYENSYLDKGIQDILRISYDELEQD--VKEIFLYISCCFVHE---------- 
Cucsa.178450  AINEFDILQSVQGLNDDEAFELFSWHAFKMSCPSSHYLYLISKRAVSYCKGLPLALEVVG-SFLYSIE-PSKLKLILDEYENQYLDKGIQDPLRISYDGLEDE--VKEIFLYISCCFVGEDINKVKMKLE 
Cucsa.091680  VTHGFDKMQNVGGLDYDEALELFSWHCFRNSHPLNVYLELS-KRAVDYCKGLPLALEVLG-SFLHSIGDPSNFKRILDEYEKHYLDKDIQDSLRISYDGLEDE--VKEIFCYISCCF------------- 
Cucsa.091710  DIHGFNKLRSVPELNYGEALELFSWHAFQCSSPPTEYLQLS-KDAVNYCKNLPLALEVLG-SFLYST-DQSKFKGILEEFAISNLDKDIQNLLQVSYDELEGD--VQEMFLFISCFFVGE---------- 
Cucsa.275630  ENHSFDIVYPIQLLDPKKSLELFSLHAFKQNHPSSNYVDLS-KFAVSYCKGLPLALVILG-SLLHKR-ERKIWKSKLHELEN-SLEPSVEAVFQIGFKELHER--VKEIFLDISCFFVGED--------- 
Cucsa.292710  ETYSFDKIHPIQLLDCDKSLELFCWHAFKQSHPSRNYSELP-ELVR-YCNGLPLALVILG-SLLCKR-DQIIWKSKLDELKN-FPEPGIEAVFQISFKRLPENPPVKEIFLDICCFFVGED--------- 
Cucsa.091840  DSHGFGQTYEVEGLWEHNAFELFCWHAFKKSHPSSNYLDLS-ERATRHCKGHPLALVVLA-SFLCGR-DQAEWSGLLDGFEN-SLRKGIKDVLQLSFDGLEDE--VKKFFLDISCLLVGET--------- 
Cucsa.091470  SSHGFDEMENILGLDEDEAIELFSWHAFKKNHPSSNYLDLS-KRATSYCKGHSLALVVLG-SFLCTR-DQVEWCSILDEFEN-SLNKDIKDILQLSFDGLEDK--VKDIFLDISCLLVGEK--------- 
Cucsa.091780  SSHGFDEMHNIQGLNQDRAIELFSWHAFKESHPSSNYLDLA-ERATSYCKGHPLALVVLG-SFLCNR-GQTEWRSILDKFEN-SLNNDIKDILQLSFDGLEGG--VKDIFLDISCLFVGEK--------- 
Cucsa.091820  SSHEFDEKYGVRELSHGHSLELFSWHAFKKSHPSSNYLDLS-KRATNYCKGHPLALVVLG-SFLCTR-DQIKWRTILDEFEN-SLSEDIEHIIQISFDGLEEK--IKEIFLDISCLFVGEK--------- 
Cucsa.091460  SSHGFDEKHKIQELNQDHALELFSWHAFKKSHPSSNYLDFS-KRATSYCKGLSLALVVLG-SFLCGR-AKEEWNGILDEFEN-SLRKDIKDVLQLSFDGLEDK--IKDIFLDISCLFVGEE--------- 
Cucsa.249360  SSHGFDEKHKIHELDQDHALVLFS---------------LS-ESATNYCKGLSVALVVLG-SFLRGR-DQTRWSCILDEFEN-SLPKDIKDVLQLSFDGLEDK--ANDIFLDISLHVIRP---------- 
Cucsa.089350  ASHGFNKLEKVNGLNAIEGLELFSWHAFNNCHPSSDYLDLS-KRAVHYCKDLPLALEVLG-SFLNSIHDQSKFERILDEYKNFYLDKDIQDILRISYDELEQD--VKDIFLYISCCFVGE---------- 


                          530        540         550        560        570        580         590         600        610        620        630         640       650            
                ....|....|....|....|....|....|....|....|....|....|....|....|....|....|....|....|....|....|....|....|....|....|....|....|....|....|
Cucsa.237410  ----------------------------------------------------------------------KKQAIEILESFGFPAVLG--------------------LEILEEKCLITTPHD---KLHM 
Cucsa.237440  ----------------------------------------------------------------------KRQAIEILESFGFPAVFG--------------------LDILKEKSLITTPHE---KIQM 
Cucsa.237540  ----------------------------------------------------------------------EDRVIEILESFGYS-PNSEV-------------------QLLMQRCLIEVSHK---KILV 
Cucsa.237560  ----------------------------------------------------------------------KDQVIDTFVSFEIDAADGLLTRKNAADVLCIKETAADALKKLQEKSLITMLYD---KIEM 
Cucsa.237520  ----------------------------------------------------------------------VDRVIQILQGFGYTSPQTNL-------------------QLLVDRCLIDILDG---HIQM 
Cucsa.237530  ---------------------------------------------------------------------------------------------------------------------------------- 
Cucsa.237390  ----------------------------------------------------------------------REVVEEILNGCGFYAKTR--------------------IELLIQKSLLTLSYDN--KLHM 
Cucsa.338650  ----------------------------------------------------------------------RNYVEQILDGCGFFPRIGIS-------------------VLLQRCLL-TIGDKN--RLMM 
Cucsa.338660  -------------------------------------------------------------------------VNQMLDGWGSFPRIG---------------------------------DIN--RLVT 
Cucsa.155730  ----------------------------------------------------------------------EELVVKIMDGCGMYGESGLR-------------------GLKWRCLVGVEFWSG--RLKM 
Cucsa.091690  ---------------------------------------------------------------------DKNEVQMMLKECDSRFRLEMG------------------IKKLTDLSLLTIDKF--NRVEM 
Cucsa.178450  ACGCLCLEKGTTKLMNLSLLTIDKSNRVEMHNLIQHMGRTIHLSKTSTSHKRKRLLIKDDAMDVLNGNKEAKGVKAIKLSFPKATELDIDSR---------------AFEKVKNVVVLEVGNV--TSSKG 
Cucsa.091680  ---------------------------------------------------------------------EARAVKVIKLNFPKPTKLDIDSR---------------AFDKVKNLVVLEVGNA--TSSES 
Cucsa.091710  ---------------------------------------------------------------------DKTMVETMLKSCG-CLCWEKG------------------IQKLMNLSLLTINQW--NKVEM 
Cucsa.275630  ---------------------------------------------------------------------INYSKD-VLKACDLNPDYGII--------------------ILMDLSLVTVED---GKIQM 
Cucsa.292710  ---------------------------------------------------------------------VSYSKN-VLKACDPYLESRII--------------------ILMDLSLVTVED---GKIQM 
Cucsa.091840  ---------------------------------------------------------------------VTYVKK-MLSEFHSILDFKIS--------------------NLRHLSLIRMEEYDDDRVQM 
Cucsa.091470  ---------------------------------------------------------------------VKYVKN-MLSACHVNLDFGII--------------------VLTDLSFITIEN---DIMQM 
Cucsa.091780  ---------------------------------------------------------------------YNNCAKKMLSACHLNVDFGIM--------------------ILMDLSLVTIEK---DRVQM 
Cucsa.091820  ---------------------------------------------------------------------DCKRLK--------HVDLSYS--------------------SLLEKIPDFPAT---SNLEE 
Cucsa.091460  ---------------------------------------------------------------------YKCAKK-MLSACHLNIDFGIM--------------------ILMDLSLITVEM---DRVQM 
Cucsa.249360  ------------------------------------------------------------------------------------LDFGIM--------------------ILMDLSLIMIES---DRVQM 
Cucsa.089350  ---------------------------------------------------------------------DINEVKMKLEACG-CLCLEKG------------------TTKLMNLSLLTIES---NRIKM 

                           660        670        680         690        700        710         720        730         740        750        760         770       780            
                 ....|....|....|....|....|....|....|....|....|....|....|....|....|....|....|....|....|....|....|....|....|....|....|....|....|....|
Cucsa.237410  HDLIQEMG---------QEIVRQNFLNEPEKRTRLWLREDVNLALSRDQGTEAIEGIMMDLD-------------------------------------------------------------------- 
Cucsa.237440  HDLIQEMG---------QKIVNEKFPDEPEKRSRLWLREDITRALSHDQGTEAIKGIMMDLD-------------------------------------------------------------------- 
Cucsa.237540  HDLILEMG---------REIVRKESLTQAEKQSRIWLHEDLYCRFAEKHDLMHIQGIVLSLAK------------------------------------------------------------------- 
Cucsa.237560  HNLHQKLG---------QEIFHE---ESSRKGSRLWHREDMNHALRHKQGVEAIETIVLDSK-------------------------------------------------------------------- 
Cucsa.237520  HILILCMG---------QEIVHRELG--NCQQTRIWLRDDARRLFHENNELKYIRGIVMDLE-------------------------------------------------------------------- 
Cucsa.237530  ---------------------------------------------------------------------------------------------------------------------------------- 
Cucsa.237390  HNLLQEMG---------RKIVRD-----KHVRDRLMCHKDIKSVVT----EALIQSIFFKSS-------------------------------------------------------------------- 
Cucsa.338650  HDLLRDMG---------REIVRENFPKYPERHSRLFLHEEVLSVLTRQKGTDATEGLSLKLP-------------------------------------------------------------------- 
Cucsa.338660  SDLLRD-------------------------HTQLFLPKEVR--------------LSVLGP-------------------------------------------------------------------- 
Cucsa.155730  HDLVRDMG---------REIVRQTCVKEPARRSRVWLYHEALKILLHQNGSENIEGLAIDMG-------------------------------------------------------------------- 
Cucsa.091690  HDLIQQMG----------HTIHLLETSNSHKRKRLLFEKDVMDVLN-GDMN------------------------------------------------------------------------------- 
Cucsa.178450  TDLEYLPSSLRWMNWPHFPFPSLPTTYTMENLMELKLPYSSIKHFGRGFMS------------------------------------------------------------------------------- 
Cucsa.091680  STLEYLPSSLRWMNWPQFPFSSLPTTYTMENLIELKLPYSSIKHFGQGYMS------------------------------------------------------------------------------- 
Cucsa.091710  HDLIQQLG----------HTIARSKTSISPSEKKLLVGDDAMHVLD-GIKH------------------------------------------------------------------------------- 
Cucsa.275630  HDLIQQMG---------QTIVRHES-FEPAKRSRLWEAEGAIKILKEKSVSDFRQCFYLLIAKD----IYSEAFRNMKNLRLLILQRVAYFPRNIFEYLPNSLKWIEWS----------TFYVN------ 
Cucsa.292710  HDLIRQMG---------QMIVRRKS-FKPEKRSRLWVAKEAVKMLIEKSGTHKVKAIKLDLRNNGSLIVEAEAFRNMENLRLLILQNAAKLPTNIFKYLPN-IKWIEYSSSSVRWYFPISFVVNGGLVGL 
Cucsa.091840  HDLIKQMG---------HKIVYDECGDEPGKRSR------------SGKRC------------------------------------------------------------------------------- 
Cucsa.091470  HDLIKQMG---------HKIVCGES-LELGKRSRLWLVQDVWEVLVNNSGTDAVKGIKLDFPNSTRLDVDPQAFRKMKNLRLLIVQNARFSTK----IEYLPDSLKWIKWHGFRQPTFPSFFTMKNLVGL 
Cucsa.091780  HGLIQQMG---------HSIVHNES-FESGKRSRLWSERDIWNVFVNNSD-------------------------------------------------------------------------------- 
Cucsa.091820  LYLNNCTN---------LRTIPKSV-VSLGK--LLTLDLDHCSNLIKLPSY------------------------------------------------------------------------------- 
Cucsa.091460  HELIQQMG---------RSIVHNES-SEPGKRSRLWLEHDIWEVFVNNSGT------------------------------------------------------------------------------- 
Cucsa.249360  HGLIQQMG---------CSIVRNES-SQPEKRSRLWLVQDIGEVFVNKSVR------------------------------------------------------------------------------- 
Cucsa.089350  HDLIQQMG----------RSIHLSKTFTSHKRKRLLIKDDAMDVLN-GNK-------------------------------------------------------------------------------- 


                          790        800        810         820        830        840         850        860        870         880        890         900       910            
                ....|....|....|....|....|....|....|....|....|....|....|....|....|....|....|....|....|....|....|....|....|....|....|....|....|....|
Cucsa.237410  --EEGESHLNAKAFSEMTNLRVLKLNNVHLS-KEIEY-LSDQLRFLNWHGYPLKTLPS--NFNPTNLLELELPNSSIHHLWTAS---------------------------------------------- 
Cucsa.237440  --EEGESHLNAKAFFSMTNLRILKLNNVHLS-EEIEY-LSDQLRFLNWHGYPLKTLPS--NFNPTNLLELELPNSSIHHLWTAS---------------------------------------------- 
Cucsa.237540  -EMEESIELDAESFSEMTKLRILEISNVELD-EDIEY-LSPLLRIINWLGYPSKSLPP--TFQSRYLFELLLPHSHLLRIWDGK---------------------------------------------- 
Cucsa.237560  --EHGESHLNAKFFSAMTGLKVLRVHNVFLS-GVLEY-LSNKLRLLSWHGYPFRNLPS--DFKPSELLELNLQNSCIENIWRET---------------------------------------------- 
Cucsa.237520  --EEEELVLKAKAFADMSELRILRINNVQLS-EDIEC-LSNKLTLLNWPGYPSKYLPS--TFQPPSLLELHLPGSNVERLWNGT---------------------------------------------- 
Cucsa.237530  ---------------------------------------------------------------------------------------------------------------------------------- 
Cucsa.237390  ---SKNMVEFPILFSRMHQLRLLNFRNVRLK-NKLEYSIPSELRYLKWKGYPLEFLPID-SSEECKLIELHMCHSNLKQFWQQE---------------------------------------------- 
Cucsa.338650  -RFS-KQKLSTKAFNEMQKLRLLQLNFVDVNGD--FKHISEEIRWVCWHGFPLKFLPKEFHMDKLVAMDLRYS--QIRFFWKES---------------------------------------------- 
Cucsa.338660  -KVR-K---------------------------------------------------------------------------------------------------------------------------- 
Cucsa.155730  -KGNNKEKFRLEAFGKMRNLRLLKLNYVHLIGSNFEHIISKELRWICWHGFPLKSIPSSFYQGNLVAIDMRYSSLIHPWTWRDS---------------------------------------------- 
Cucsa.091690  ----------------CKWLKRINLNYSKFLEEISDLSSAINLEELNLSECKKLVRVHESVGSLGKLAKLELSSHPN----------------------------------------------------- 
Cucsa.178450  ----------------GERLKEIDLSGSEFLVEIADLSTATNLEKLNLLGCVNLVKVHDSVGSLTKLVTFSLSSNVK----------------------------------------------------- 
Cucsa.091680  ----------------CERLKEINLSDSNLLVEIPDLSTAINLKYLNLVGCENLVKVHESIG-------------------------------------------------------------------- 
Cucsa.091710  ----------------CERLKQLDLSNSFFLEEIPDLSAAINLENLSLSGCISLVKVHKSVGSLPKLIDLSLSS-------------------------------------------------------- 
Cucsa.275630  -------QSSSISFS-----------------ETPNFFATLNLEKLYLRGCTSLKGMR-----------------------PS----------------------------------------------- 
Cucsa.292710  VINGVSNKHPGIIFEDCKMLKHVDLSYWRLLEETPDFSAALNLEKLYLLSCKRLKMIHGSVASLSKLVTLDLEGCENLEKLPSSFLMLKSLEVLNLSGCIKLKEIPDLSASSNLKELHLRECYHLRIIHD 
Cucsa.091840  --------------------------------------SERHKVGVYLIPPRVINVNPEAFRSMKNLRILIVDG-------------------------------------------------------- 
Cucsa.091470  DLQHSFIKTFGKRLEDCERLKYVDLSYSTFLEKIPNFSAASNLEELYLTNCTNLGMIDKSVFSLDKLTVLNLDGCSNLKKLPRG---------------------------------------------- 
Cucsa.091780  ----------------CERLKHVDLSYSTLLEKIPDLSAASNLEELYLINCTNLGMIDKSVFSLNKLTVLNFKGCSNLKKLPKG---------------------------------------------- 
Cucsa.091820  --------------LMLKSLKVLKLAYCKKLEKLPDFSTASNLEKLYLKECTNLRMIHDSIGSLSKLVTLDLGKCSNLEKLPS----------------------------------------------- 
Cucsa.091460  --------------------------------------DAVKAIKLDLPKSTRLNVDPRAFGSMKNLRLLIIR--------------------------------------------------------- 
Cucsa.249360  --------------NSLPKLKHVDLSYSTLLEKILDFSAASNLEELYLTNCTNLGMLDKSILSLNKLTVLNFEGCSNLKMLSRG---------------------------------------------- 
Cucsa.089350  ----------------------------------CDLTTAINLEKLNLEGCEKLVKVHESVGSLSKLVEFYLSSSVE----------------------------------------------------- 

                          920        930        940         950        960         970        980        990        1000       1010       1020       1030      1040           
                ....|....|....|....|....|....|....|....|....|....|....|....|....|....|....|....|....|....|....|....|....|....|....|....|....|....|
Cucsa.237410  -----------------------------------------------------------K---------------------------------------------------------------------- 
Cucsa.237440  -----------------------------------------------------------KVHQNNSSN-------------------------------------------------------------- 
Cucsa.237540  -----------------------------------------------------------KRFPKLKLIDVSNSEHLRVTPDFSGVPNLERLVLCNCVRLCEIHPSINSLNKLILLDLEGCGDLKHFPANI 
Cucsa.237560  -----------------------------------------------------------EKLDKLKVINLSNSKFLLKTPDLSTVPNLERLVLNGCTRLQELHQSVGTLKHLIFLDLKDCKSLKSICSNI 
Cucsa.237520  -----------------------------------------------------------QNFKNLKEIDASDSKFLVETPNFSEAPKLRRLILRNCGRLNKVHSSINSLHRLILLDMEGCVSFRSFSFPV 
Cucsa.237530  ---------------------------------------------------------------------------------------------------------------------------------- 
Cucsa.237390  -----------------------------------------------------------KNLVELKYIKLNSSQKLSKTPNFANIPNLKRLELEDCTSLVNIHPSIFTAEKLIFLSLKDCINLTNLPSHI 
Cucsa.338650  -----------------------------------------------------------KFLKNLKFLNLGHSHYLTHTPNFSKLPNLEILSLKDCKNLIELHPTIGELKALISLNLKDCKSLNSLPNSF 
Cucsa.338660  ---------------------------------------------------------------------------------------------------------------------------------- 
Cucsa.155730  -----------------------------------------------------------QILENLKVLNLSHSEKLKKSPNFTKLPNLEQLKLKNCTALSSLHPSIGQLCKLHLINLQNCTNLSSLPTSI 
Cucsa.091690  -------------------------------------------------------------------GFTQFPSNLKLKSLQKLVMYECRIVESYPHFSEEMKSSLKELRIQSCSVTKLSPTIGNLTG-- 
Cucsa.178450  -------------------------------------------------------------------GFEQFPPHLKLK----------------------------------------SLKLLSMENCR 
Cucsa.091680  -----------------------------------------------------------------------------------------------------------------------SLKLTTLP--- 
Cucsa.091710  ---------------------------------------------------------------------------------------------------------------------HLSSTIRYLTS-- 
Cucsa.275630  ----------------------------------------------------FRKFPSHLKFKSLKVLNLRDCLNLEEITDFSMASNLEILDLNTCFSLRIIHESIGSLDKLITLQLDLCHNLEKLPSS- 
Cucsa.292710  SAVGRFLDKLVILDLEGCKILERLPRYISNSKSIEVMNLDSCRKIEQLFDNYFEKFPSHLKFESLKVLNLSYCQNLKEITDFSIASNLEIFDLRGCFSLRTIHKSVGSLDQLIALKLDFCHQLEELPSC- 
Cucsa.091840  --------------------------------------------------------------------NVRFCKKKKYVPNG----------LKWIKWHRFPHQTLPSC----------FITKDLLPSY- 
Cucsa.091470  ----------------------------------------------------------YFMLSSLKKLNLSYCKKLEKIPDLSSASNLTSLHIYECTNLRVIHESVGSLDKLEGLYLKQCTNLVKLPSY- 
Cucsa.091780  ----------------------------------------------------------YFMFSSLKILNLSYCQELEKIPDLSSASNLQSLLLNGCTNLRVIHESVGSLNELVLLDLGQCTNLSKLPSY- 
Cucsa.091820  ----------------------------------------------------------YLTLKSLEYLNLAHCKKLEEIPDFSSALNLKSLYLEQCTNLRVIHESIGSLNSLVTLDLRQCTNLEKLPSY- 
Cucsa.091460  --------------------------------------------------------------------NARFCTKIRYLPNS----------LKWIEWHGFAHRTLPSC----------FITKNLVG--- 
Cucsa.249360  ----------------------------------------------------------YFMLSSLKELRPSYYKKLEKIPDLSAASNLKRLYLQECTNLRVIHKSVGSLDKLGLLDLSQCTNLVKLPSY- 
Cucsa.089350  -------------------------------------------------------------------GFEKFPSCLKLNSLEALVVRYCRIEECCPQFSEEMN-SLEILEIDDSIINQLSPTIEYLTG-- 

                          1050        1060       1070       1080       1090       1100       1110       1120       1130        1140       1150       1160      1170           
                 ....|....|....|....|....|....|....|....|....|....|....|....|....|....|....|....|....|....|....|....|....|....|....|....|....|....|
Cucsa.237410  ---------------------------------------------------------------------------------------------------------------------------------- 
Cucsa.237440  ---------------------------------------------------------------------------------------------------------------------------------- 
Cucsa.237540  R----CKNLQTLKLSGTG-LEIFPEIG-HMEHLTHLHLDGSNITHFHPSIGYLTGLVFLDLSSCLGLSS-LPCEIGNLKSLKTLLLKYCKKLDKIPPSLANAESLETLSISETSITHVPPSIIHCLKNLK 
Cucsa.237560  S----LESLKILILSGCSRLENFPEIVGNMKLVKELHLDGTAIRKLHVSIGKLTSLVLLDLRYCKNLRT-LPNAIGCLTSIEHLALGGCSKLDKIPDSLGNISCLKKLDVSGTSISHIPFTLR-LLKNLE 
Cucsa.237520  T----CKSLKTLVLSNCG-LEFFPEFGCVMGYLTELHIDGTSINKLSPSITNLLGLVLLNLRNCIRLSS-LPTEICRLSSLKTLILNGCKNLDKIPPCLRYVKHLEELDIGGTSISTIP-----FLENLR 
Cucsa.237530  ---------------------------------------------------------------------------------------------------------------------------------- 
Cucsa.237390  N----IKVLEVLILSGCSKVKKVPEFSGNTNRLLQLHLDGTSISNLPSSIASLSHLTILSLANCKMLID-ISNAIEMTS-LQSLDVSGCSKLGSR-KGKGDNVELGEVNVRETTRRRRNDDCNNIFKEIF 
Cucsa.338650  SN---LKSLQTLIISGCSKLNSLPEDLGEITSLITLIADNTPIQKIPNTIINLKNLKYLSLCGCKGSPS-KSSFSSMIWSWISPKKLSQN--YTSILLPSSLQGLN------------------------ 
Cucsa.338660  ---------------------------------------------------------------------------------------------------------------------------------- 
Cucsa.155730  YN---LHSLQTFIISGCSKIHCLHDDLGHLESLTTLLADRTAISHIPFSIVKLKKLTDLSLCGCNCRSG-SGSSASLPWRLVSWALPRPNQTCTALTLPSSLQGLS------------------------ 
Cucsa.091690  --------LQHLWIDVCKELTTLPSTICHLSNLISLSVFRSEVSTFSFLYSRSLS---------------------LFPYLTLLKLCYCKITNL------------------------------------ 
Cucsa.178450  IDEWCPQFSEEMKSSLEELLIQYSTVINQLSPTIGYLTSLKRLFIIECMKLKTLPS--------------------TIYRLRNLTFL--SITNL------------------------------------ 
Cucsa.091680  ------------------------STIYRLTNLTSLTVLDSNLSTFPFLNHPSLPS--------------------SLFYLTKLRIVGCKITNL------------------------------------ 
Cucsa.091710  --------LKDLTIVDCKKLTTLPSTIYDLSKLTSIEVSQSDLSTFPSSYSCPSS----------------------LPLLTRLHLYENKITNL------------------------------------ 
Cucsa.275630  ---LKLKSLDSLSFTNCYKLEQLPEFDENMKSLRVMNLNGTAIRVLPSSIGYLIGLENLNLNDCANLTA-LPNEIHWLKSLEELHLRGCSKLDMFPPRSSLN---------------------------- 
Cucsa.292710  ---LRLKSLDSLSLTNCYKIEQLPEFDENMKSLREMNLKGTAIRKLPTSIRYLIGLENLILSYCTNLIS-LPSEIHLLKSLKELDLRECSRLDMLPSGSSLN---------------------------- 
Cucsa.091840  ---LKLKSLTDLDLSGCRKLETFPEIDENMKSLERLRLSYTAIRKLPSVFAGLVFLSVLLKCVPKFFHSRVPKEILFFEHLELLDLKGCNISNV------------------------------------ 
Cucsa.091470  ---LSLKSLLCLSLSGCCKLESFPTIAKNMKSLRTLDLDFTAIKELPSSIRYLTELWTLKLNGCTNLIS-LPNTIYLLRSLENLLLSGCS---------------------------------------- 
Cucsa.091780  ---LRLKSLVYLVLFGCGKLESFPTIAENMKSLRCLDLHSTAIKELPSSLGYLTQLDKLHLTGCTNLIS-LPNTIYLLRNLNELHLGGCSRFEMFPHKWVPTIQPVCSPSKMMEAASWSLEFP------- 
Cucsa.091820  ---LKLKSLRHFELSGCHKLEMFPKIAENMKSLISLHLDSTAIRELPSSIGYLTALLVLNLHGCTNLIS-LPSTIYLLKSLKHLYLGGCSRFQLFS---------------------------------- 
Cucsa.091460  -----------------------------------LDLQHSLIKRFG---------------------------------------KRLKVS-------------------------------------- 
Cucsa.249360  ---LRLKSLYTLYLSGCCKLESFPTIAENMKYLEELYLNFTAI--------------------------------------------------------------------------------------- 
Cucsa.089350  --------LKELWITECTKLETLPSTIYRLSNLTSLEVKKSDLSIFPSLNDPSSSSL-------------------LIPYLTSIKLFNCQITNL------------------------------------ 

                         1180       1190        1200       1210       1220       1230       1240       1250       1260        1270       1280       1290      1300           
                ....|....|....|....|....|....|....|....|....|....|....|....|....|....|....|....|....|....|....|....|....|....|....|....|....|....|
Cucsa.237410  ---------------------------------------------------------------------------------------------------------------------------------- 
Cucsa.237440  ---------------------------------------------------------------------------------------------------------------------------------- 
Cucsa.237540  TLDCEGLSHGIWKSLLPQFNINQTITTGLG------------CLKALNLMGCKLMDEDIPEDLHCFSSLETLDLSYNNFTTLP-DSLSHLKKLKTLNLNCCTELKDLPKLPESLQYERFRSKFDLLLHGD 
Cucsa.237560  VLNCEGLSRKLCYSLFLLWSTPRNNNSHSFGLWLITCLTNFSSVKVLNFSDCKLVDGDIPDDLSCLSSLHFLDLSRNLFTNLP-HSLSQLINLRCLVLDNCSRLRSLPKFPVSLLYVLARDCVSLKEHYN 
Cucsa.237520  ILNCERLKSNIWHSLAG------LAAQYLR------------SLNDLNLSDCNLVDEDIPNDLELFSSLEILDLSSNHFERLS-ESIKQLINLKVLYLNDCNKLKQVPKLPKSIKYVGGEKSLG------ 
Cucsa.237530  ---------------------------------------------------------------------------------------------------------------------------------- 
Cucsa.237390  LWLCNTPATGIFG---------------------IPSLAGLYSLTKLNLKDCN--LEVIPQGIECMVSLVELDLSGNNFSHLP-TSISRLHNLKRLRINQCKKLVHFPKLPPRILFLTSKDCISLKDFID 
Cucsa.338650  ------------------------------------------SLRKLCLKNCNLSNNTIPKDIGSLSSLRELDLSEN-LFHSLPSTISGLLKLETLLLDNCPELQLIPNLPPHLSSLYASNCTSLERTSD 
Cucsa.338660  ---------------------------------------------------------------------------------------------------------------------------------- 
Cucsa.155730  ------------------------------------------SLTELSLQNCNLES--LPIDIGSLSELKKLNLGGNKNLRVLGTELCGLLKLNELNVENCGRLEFIQEFPKNMRSFCATNCKSLVRTPD 
Cucsa.091690  ---------------------------------SFLETITHVAPSLTQLYLTGNDFCSLPSCIVNFKSLRYFDISYCGSLARFPDNIAEFISCDSEYVDGKYKQLILMNNCDIPEWFHFKSTNNSITFPT 
Cucsa.178450  ---------------------------------DFLETMVHVAPALKLLDLSGNNFCRLPSCIINFKSLKSLVTMECKLLEEIPKVPKGVVRMNAT---GCISLTRFPDN-----IPDFICCDDNVVR-- 
Cucsa.091680  ---------------------------------DFLETIVYVAPSLKELDLSENNFCRLPSCIINFKSLKYLYTMDCELLEEISKVPEGVICMSAA---GSISLARFPNN-----LADFMSCDDSVEYCK 
Cucsa.091710  ---------------------------------DFLETIAHAAPSLRELNLSNNNFSILPSCIVNFKSLRFLETFDCKFLEEIPKIPEGLISLVNH-----------------------VDRNRYMDLEC 
Cucsa.275630  ----------FSQESSYFKLTVLDLKNCNISNSDFLETLSNVCTSLEKLNLSGNKFSCLPS-LQNFKSLRFLELRNCKFLQNIIKLPHHLARVNAS---GSELLAIRPDC-----IADMIYLHANDR--- 
Cucsa.292710  ----------FPQRSLCSNLTILDLQNCNISNSDFLENLSNFCTTLKELNLSGNKFCCLPS-LKNFTSLRLLELRNCKFLRNIVKIPHCLKRMDAS---GCELLVISPDY-----IADMMFRNQRD---- 
Cucsa.091840  ---------------------------------DFLENLCNVALSLTSIVLSENEFCSLPSCLHKFMSLRNLQLRNCMFLQEIPNLPQSIQIVDAT---GCISLRRSPNI-----MWT------------ 
Cucsa.091470  ------------------------------------------------------IFGILPSCLHKFMSLWNLELRNCKFLQEIPSLPESIQKMDAC---GCESLSRIPDN-----IVDIISKKQDLTMGE 
Cucsa.091780  -------HLVVPNESICSHFTLLDLKSCNISNAKFLEILCDVAPFLSDLRLSENKFSSLPSCLHKFMSLSNLELRNCKFLQEIPNLPQNIRNLDAS---GCKSLARSPDN-----IVDIISIKQDLELGE 
Cucsa.091820  ---------------------------------HFLEILCNVAPFLSSILLSENKFSSLPPCLHKFMSLWNLQLRNCKFLQEIPNLPHCIQKMDAT---GCTLLGRSPDN-----IMDIISSKQ------ 
Cucsa.091460  ---------------------------------------------------SRN---SEPSSKHTNFECEWL---------------------------------------------------------- 
Cucsa.249360  ---------------------------------------------------------------------------------------------------------------------------------- 
Cucsa.089350  ---------------------------------DFLETMVHVTPSLEMLDLSQNNFCGLPSCIINFKSLKYLYIIECKSLEEILKVPKGVVRMDTR---GCVSLAKFPN--NIPD---FISCDDNVEYDT 

                         1310       1320       1330        1340       1350       1360       1370       1380       1390       1400       1410        1420      1430           
                ....|....|....|....|....|....|....|....|....|....|....|....|....|....|....|....|....|....|....|....|....|....|....|....|....|....|
Cucsa.237410  ---------------------------------------------------------------------------------------------------------------------------------- 
Cucsa.237440  ---------------------------------------------------------------------------------------------------------------------------------- 
Cucsa.237540  ---------------------------------------------------------------------------KIPKFFSNQSKGNMTEIKLPQYLEKFRESIGVAVCALVVVDKKRRKLNEIIPERE 
Cucsa.237560  ---------------------------------------------------------------------------YNKEDRGPMSQAEVRVLSYPSSAKDQNSKISQLMISSMCTACENGG--------- 
Cucsa.237520  ---------------------------------------------------------------------------------------------------------------------------------- 
Cucsa.237530  ---------------------------------------------------------------------------------------------------------------------------------- 
Cucsa.237390  ---------------------------------------------------------------------------ISKVDNLYIMKEVNLLNCYQMANNKDFHRLIISSMQKMFFRKGTFNIMIPGSEIP 
Cucsa.338650  ---------------------------------------------------------------------------LSNVKKMGSLSMSNCPKLMEIPGLDKLLDSIRVIHMEGCSNMSNSFKDTILQGWT 
Cucsa.338660  ---------------------------------------------------------------------------------------------------------------------------------- 
Cucsa.155730  ---------------------------------------------------------------------------VSMFERAPNMILTNCCALLEVCGLDKLECSTN-IRMAGCSNLSTDFRMSLLEVFS 
Cucsa.091690  TFNYPGWKLKVLAACVKVQVHDPVNGYHRGGDLECEVFFK-------------------------------DILVWSSGDWTNYLGYDSR----WLPLGASPSEYTWFIVLNPHRDFSLDDW-DDMMERS 
Cucsa.178450  -----------------------------------------------------------------------------IIVLSHDLMISR--------------------------VFRSYKN-------- 
Cucsa.091680  --------GGELKQLVLMNCHIPDWYRYKSMSDSLTFFLPADYLSWKWKPLFAPCVKFEVTNDDWFQKLECKVFINDIQVWSSEEVYANQKERSGMFGKVSPGEYMWLIVLDPHTRFQSYSDDIMDRRSL 
Cucsa.091710  ---------KMFINDIQILSR----------------------------------------------------------DHRSTLIYIS------QVLGSIPWEGTIIIVIFEHRILSTYKFFSFLPISS 
Cucsa.275630  --------------------------------------HHIKVL----------------------FPNTTSKFVSKRFKSANVIMRTQGVYMVDRRFSCYT---------------------------- 
Cucsa.292710  ---------------------------------------------------------------------------------PEILQQSN-----HRKFNKF----------------------------- 
Cucsa.091840  ---------------------------------------------------------------------------------------------------------------------------------- 
Cucsa.091470  ISREFLLTGIEIPEWFSYKTTSNLVSASFRHYPDMERTLAACVS----------------------FKVNGNSSERGARISCNIFVCNRLYFSLSRPFLPSKSEYMWLVTTS--LALGSMEVNDWNKVLV 
Cucsa.091780  ILREFLLTDIEIPEWFSYKTASNLVTASLRHYPDMERTLAVAVS----------------------FKVNGDSSESEAQISCNIFIYNKLRCLFSRSFLPSKSEYMWLVTIS--LAC-SLEVNDWNKVFV 
Cucsa.091820  -------------------------------------VPHFHFH----------------------FPLVGDSYQGMALVSCKIFIGYRLQSCFMRKFPSSTSEYTWLVTTSSPTFSTSLEMNEWNHVTV 
Cucsa.091460  ---------------------------------------------------------------------------------------------------------------------------------- 
Cucsa.249360  ---------------------------------------------------------------------------------------------------------------------------------- 
Cucsa.089350  -------KDGVIKQLILMNCDIP--------------------------------------------------------DWCNRMSISN------FNFWLSRGECLWMAVLHPCMHRLINPYGDDIMDIS 

                         1440       1450       1460        1470       1480       1490       1500       1510       1520       1530       1540        1550      1560           
                ....|....|....|....|....|....|....|....|....|....|....|....|....|....|....|....|....|....|....|....|....|....|....|....|....|....|
Cucsa.237410  ---------------------------------------------------------------------------------------------------------------------------------- 
Cucsa.237440  ---------------------------------------------------------------------------------------------------------------------------------- 
Cucsa.237540  RYTKVVDLICKFKVDSYQIMPEHCHFTSQQKLLSEYASQFLWLSYIPLHGFNINWHYCTQFEIALETSCDELFGVKNCGLHLIHKHERMMIDKMVMESTVPSSTSHKGKEPQIH---------------- 
Cucsa.237560  ---------------------------------------------------------------------------------------------------------------------------------- 
Cucsa.237520  ---------------------------------------------------------------------------------------------------------------------------------- 
Cucsa.237530  ---------------------------------------------------------------------------------------------------------------------------------- 
Cucsa.237390  DWFTTRKMGSSG---------------------------------------------------------------------------------------------------------------------- 
Cucsa.338650  VSGFGGVCLPGKEVPDWFAYKDEGHSIFLELPQYNNSNLEGFIVCIVYCSCFNNTVSTDLPSLSVINYTKSSITTNKPLTNDVIMSTQDHLWQGHLSNKAFKMEPGDEVEIIVDFGAEITVKKIGISLVF 
Cucsa.338660  ---------------------------------------------------------------------------------------------------------------------------------- 
Cucsa.155730  SS------------------------YLLLNYKFGFAVFHLYIVSI------------------------------------------------------------------------------------ 
Cucsa.091690  PETDLSQLCFGINSMEMDRNRSNDKWNSIGGSIWKNFTVLFEPRPLSPDTTI------------------------------------------------------------------------------ 
Cucsa.178450  ---------------------------------------------------------------------------------------------------------------------------------- 
Cucsa.091680  KIIDLNQLSSEINSSQSILG---------------KITVSFEVTP------------------------------------------------------------------------------------- 
Cucsa.091710  PK-------------------------------------------------------------------------------------------------------------------------------- 
Cucsa.275630  ---------------------------------------------------------------------------------------------------------------------------------- 
Cucsa.292710  ---------------------------------------------------------------------------------------------------------------------------------- 
Cucsa.091840  ---------------------------------------------------------------------------------------------------------------------------------- 
Cucsa.091470  WFEVHEAHSEVNATITRYGVHVTEELHAIQTDVKWPMVNYADFYQLEKLQSLDIEELLLKRLFEEMSCWSNSQAMLYAANYDPEAIIDSNIQPMIFPLHVTYNGETFICGMEGMGDTTLANSLCNKFNRS 
Cucsa.091780  WFEVHEAH---GVTVTRYGVHVTEQLHGIQTDVKWPMVNYADFYQLEKLRRDL----------------------------------------------------------------------------- 
Cucsa.091820  WFEVVKCS-EATVTIKCCGVHLTEEVHGIQNDVKGPGVVYTVFDQLDKLPSRIR-------------------------------------------------------GMEGMAETTLANSICNKYERS 
Cucsa.091460  ---------------------------------------------------------------------------------------------------------------------------------- 
Cucsa.249360  ---------------------------------------------------------------------------------------------------------------------------------- 
Cucsa.089350  PN-------FSIGILDN------------------KITLLFEVNPECKDT-------------------------------------------------------------------------------- 

                         1570       1580       1590       1600       1610        1620       1630       1640       1650       1660        1670       1680      1690           
                ....|....|....|....|....|....|....|....|....|....|....|....|....|....|....|....|....|....|....|....|....|....|....|....|....|....|
Cucsa.237410  ---------------------------------------------------------------------------------------------------------------------------------- 
Cucsa.237440  ---------------------------------------------------------------------------------------------------------------------------------- 
Cucsa.237540  ---------------------------------------------------------------------------------------------------------------------------------- 
Cucsa.237560  ---------------------------------------------------------------------------------------------------------------------------------- 
Cucsa.237520  ---------------------------------------------------------------------------------------------------------------------------------- 
Cucsa.237530  ---------------------------------------------------------------------------------------------------------------------------------- 
Cucsa.237390  ---------------------------------------------------------------------------------------------------------------------------------- 
Cucsa.338650  DKYVDQTMLEFASTCNDDDVVVDNQDETVSEKDGEVGSKRGFDENDDEGLKNSYQIPKRLKCEIDSNMKIDEE--------------------------------------------------------- 
Cucsa.338660  ---------------------------------------------------------------------------------------------------------------------------------- 
Cucsa.155730  ---------------------------------------------------------------------------------------------------------------------------------- 
Cucsa.091690  ---------------------------------------------------------------------------------------------------------------------------------- 
Cucsa.178450  ---------------------------------------------------------------------------------------------------------------------------------- 
Cucsa.091680  ---------------------------------------------------------------------------------------------------------------------------------- 
Cucsa.091710  ---------------------------------------------------------------------------------------------------------------------------------- 
Cucsa.275630  ---------------------------------------------------------------------------------------------------------------------------------- 
Cucsa.292710  ---------------------------------------------------------------------------------------------------------------------------------- 
Cucsa.091840  ---------------------------------------------------------------------------------------------------------------------------------- 
Cucsa.091470  NDNGWPREALDDSTSFLHFRGGKFYGGSWSLSHHRKRGDGERGTNITTRTISSKRYLILFHKAGSYNHLFNFAGSHRLIAGSGSYDSLNGRGDVRLLIERVDTSLL------------------------ 
Cucsa.091780  ---------------------------------------------------------------------------------------------------------------------------------- 
Cucsa.091820  RNLFSAKKALNHSTGFLCGDGNGLSWEMVDR-------------PILSDRLSSQKYLRIFDDRDRYGDLNDVAHGTGNRFRSRFLRMDDIKEDDIREEPYWKYME------------------------- 
Cucsa.091460  ---------------------------------------------------------------------------------------------------------------------------------- 
Cucsa.249360  ---------------------------------------------------------------------------------------------------------------------------------- 
Cucsa.089350  ---------------------------------------------------------------------------------------------------------------------------------- 


Additional file 12B

                           10          20         30          40          50         60          70         80          90         100        110        120        130                     
                 ....|....|....|....|....|....|....|....|....|....|....|....|....|....|....|....|....|....|....|....|....|....|....|....|....|....|
Cucsa.017460  --------------MAEFLWTFAVQEILKKVLTLVAEQIILAREVKDVLQQLQKELVESQKIVSAITTQRQNHYSPDSLVTQWVNDLQLIVHEADDLLDFILNKNQ--PIERLWSVIS---LSCLLYSSN 
Cucsa.017490  ---------------------------------------------------------------------------------------------------------------------------------- 
Cucsa.088220  ---------------------------------------------------------------------------------------------------------------------------------- 
Cucsa.091880  -----------MESIPISIIAKICEYTVKPVGRQLCYVCFIHSNFQKLKSQVEKLTDTKGSVEDKVFIARRNAEDIKPAVEKWLEKVDRLVRKSEKILAHEGRHGR----LCSTNLVQR----------- 
Cucsa.094560  ---------------------------------------------------------------------------------------------------------------------------------- 
Cucsa.094580  ------------MAIAEFLWTYAVQQ----------------------------VLKKVLELAADQIEKKLHHSS----VRLWVADLLLVVHEADNLLDELVYEYLRTKVEKGSINKV---CSSVSSLSN 
Cucsa.094650  --------------MAEFLWTFAVEETLKRTVNVAAQKISLVWGLEDELSNLSKWLLDAGALLRDIDREILRKES----VKRWADGLEDIVSEAEDLLDELAYEDLRRKVETS--SRV---CNNFKFSSV 
Cucsa.094660  --------------MADFLWSFAVDEVLKKTVKLVAEQIGMSWGFKKDLSKLRDSLLMVEAILRDVNRIKAEHQA----LRLWVEKLEHIVFEADVLLDELSYEDLRRKVDARP---V---RSFVS-SSK 
Cucsa.094670  ---------------MDLLYSKNYED--------------------KILSNLRDSLLMVEAILRDVDRIKAEHQA----VKLWVEKLEAIIFEVDVLLDELAYEDLRRKVEPQKEMMV---SNFIS-FSK 
Cucsa.102240  ---------MALELVGGAVLGAVVGELFKAILNLGERAISFNPVLKDIRSKLNAIMPLVKQIDELNDYLDYPKEE-TEKLRGLMDEGKQ-LLLQCGDVKLGDLNYLKRPSYTQKLRELDTALRSFMDVLM 
Cucsa.123410  ------------MAVTDFFVGEIATELLRMMVQLSTKSCLCKTTAAQIANSIQQILPIIEEIKYS--GVELPAHR-QFQLDRFSETLRRGIEISEKALQCGRLNIYRNLRLARKMEKLEKDICRFINGTM 
Cucsa.128030  -MAEA-----ILFQVAGEI-------LMKLSSQAFQRLGMLFG-LKGDLNKLTTTVSTIKDVLLDAEGRQTKS----HLLQNWLHKLEEALYDAEDVLDELSTEALRRELMTRDH--KNAKQVRIFFSKS 
Cucsa.128100  -MADS-----VLFNVAA-------SVITKLGSSALRELGSLWG-VNDELDKLQNTLSAIKAVLLDAEEQQSKS----HTVKDWIAKIKDVFYDIDDLIDEFSYETLRRQVLTKDRTIT--KQVRIFFSKS 
Cucsa.128110  -MADS-----ILFNVAA-------NVITKLGSSALRELGSLWG-VNDELGKLQNILSAIKAVLLDAEEQQSVS----HAVKDWISKLRDVFYDVDDLIDEFSYETLRRQVLTKDRTIT--KQVCIFFSKS 
Cucsa.128130  -MAIGDPQLPILHRAPVDKS----HGIKKLSSHALECLGMVCG-LNDDLNKLRSNVSSIQSVLRDAEQRQIKGND--HSLTDWLEKLGDVFYDVEDVLDEISTEALRREVMTRG---KNAKQVRIFFSNS 
Cucsa.128140  -MAEA-----ILYNVTA-------DIIFKLGSSALQELGLLWG-VNDELDKLKHSLSAIQAVLLDAEEQQSKS----LAVKAWVSRLKDALYEIDDLVDESSYETLRRQVLAKDQRKR--KLVRILFSK- 
Cucsa.132370  ---------------MGDLLTFGVQETLKQAVTLVAKKIIASSEFKVVLEELKDDLLHAEWILHAIKT--KHDHSLNDKITHWVNDLQLIVYEAEDMLDLFAYDD----VER------------KIRSNK 
Cucsa.133510  ---------MAGALIGGAALGVPFNELATLLKNFGERAWSFNSVLNETESKVNDIIPLVKEIDGLNESLDYPREE-TEKLKNLLEYAGK-LLRRC--LRVGKADLIRKSSHTEKLRELNARIKSFSDVVL 
Cucsa.163670  MIPHE-----ILSLFITSVYEYLTNIATKLGSLALQDLGLLWTGIHEEIDKLRDTLSAIQAVLHDAEQKQYKS----SAVKEWVSRLKDAFYDMDDLMDEFSYESFQRQVMTKHRTNNCTKQVCIFFSKS 
Cucsa.178360  -------MELCAGAIVNPIAEKIANCTVDPVFRQLDYLLHFKTNVNDLKDQGKKLVETRDFVQHSVDSAKTNGYEIEVMVTEWLGIADQFSEDVDRFFN-EADGRS----LRWWNMLSR----------- 
Cucsa.178620  -MAEA-----ILFNLTA-------DIIFKLGSSALRQFGSLRGGVKDDFDKLWHSLSAIQAVLHDAEEKQFKD----HAVEVWVSRLKDVLYEIDDLIDEFSYQILRRQVLRSN---R--KQVRTLFSK- 
Cucsa.189390  ------------MDIISPVVGPIVEYTLKPIGRQLSYLFFIRQHIQNLESQVELLKNTKESVVNKVNEAIRNAEKIESGVQSWLTKVDSIIERSETLLKNLSEQGG----LCLN-LVQR----------- 
Cucsa.237070  ------------METVIAILGTVCEYAVAPIGRQVGYVSSYKKNINDLKDQLQNLVDTKTRLQHMVNEARSSAYNIQSDVSSWLNQVDKIIEQSNDILYKNENESNSKYCSNKLNFIHQ----------- 
Cucsa.239860  ---------------------------------------------------------------------------------------------------------------------------------- 
Cucsa.248810  --------------MADFLWTFAVEEMLKNVLKVAGEQTGLAWGFQEHLSNLQKWLLNAQAFLRDINTRKLHLHS----VSIWVDHLQFLVYQAEDLLDEIVYEHLRQKVQTT-EMKV---CDFFSLSTD 
Cucsa.251930  --------------MADFIWTFALQEILKKTLHLATQQIRLASGFNHDLSKLLHSLLFFEAILRDVDRTKSDLQS----VKIWVTKLQDLVLDAEVVLDELSYEDLRREVDVNGNSKK---RVRDFFSFS 
Cucsa.277260  --------------MAEFLWTFAVQEVLKKIVNFGAEQISLAWGLEKELSHLKKWLLKAQTILADINTKKSHHHS----VGLWVEELHDIIYEADDLLDEIVYEQIRQTVEQTGKLRK---VRDSISPSK 
Cucsa.318890  ---------------------------------------------------------------------------------------------------------------------------------- 
Cucsa.326910  ---------------------------KIPILHQLDYLFHYKQNIKEVEKKVEALGTAKG------------------------NAVFDGVSKWLTIVKDVLEIAQQNENPSCFNFVER----------- 
Cucsa.328080  -MAES-----ILCSLAG-------SIITKLGSFALQDLGLLWG-FHDELDKLKGTVSALEAVLLDAEEKQSKS----RAVKDWILKLKDTFYDIDDLLDVFSYESLKRQVMTKHRTNN-TKKVRIFFSKS 
Cucsa.337180  --------------------------MACCIYEQAENILIELKKFPMYLRRMQYTMLSLKTILKDAE-KEEYRHC----LNDWLQKLQSVFLQIEELLYESNREVKKQEATGKWVFLP-----SFNFSQI 
Cucsa.337190  --------------------------MAYCIYYRAENILSELKNLPNYPRRIEYTMLSLKSILMDAEEKQEQSRG----LQNWLEELQNVFSQIEGFIDEHKEEAY--EGIGKQVLAPF----SCSSNQI 
Cucsa.338110  ---------------------------------------------------------------------------------------------------------------------------------- 
Cucsa.338190  -----------------MVGLLDSVAGNLLGRIIEAADRLEFRAIQSELKNLETDVLNLKARLRDAEEKQASN----CELNELLKNLKNVFSRADIAIEELECDYLKWRVQNRKNDVDDKGCQFSSCFSS 

                           140        150        160         170        180        190         200        210         220        230        240         250       260            
                 ....|....|....|....|....|....|....|....|....|....|....|....|....|....|....|....|....|....|....|....|....|....|....|....|....|....|
Cucsa.017460  P-------ETKKMKE----IIALLNKHCTKLPHLLQLEPTPSNIAETE----VAQIQETVSKPE-DYVVGRNREVE---------TIVDRVIDASK---QELNSILPVFGMGGLGKTTLAKSVFNHDR-I 
Cucsa.017490  --------------------------------------------------------------------------------------------------------------MAGVGKTYFLNEVKKLVLKG 
Cucsa.088220  --------------------------------------------------------------------------------------------------------------MGGVGKTMLVKEILRKIV-E 
Cucsa.091880  ----------HKASR---------KASKMADEVLEMKNQGESFDMVSF----KGRISLVESPLPKAPDFLDFGSRK---------STVEQIMDALS---DDNVHKIGVYGMGGVGKTMLVKEIVRKIE-E 
Cucsa.094560  --------------------------------------------------------------------------------------------------------------MGGIGKTTLAKTIFNHEE-I 
Cucsa.094580  IFIIFRFKMAKKIKS----IIEKLRKCYYEATPLGLVGEE-FIETEND----LSQIRETISKLDDFEVVGREFEVS---------SIVKQVVDAS---NQYVTSILPIMGMGGIGKTTLAKTIFNHEE-I 
Cucsa.094650  LNPLVRHDMACKMKK----ITKMLKQHYRNSAPLGLVGKESMEKEDGGNN--LRQIRETTSILN-FDVVGRETEVL---------DILRLVIDSSSNEYELPLLIVPIVGMGGVGKTTLAKLVFRHEL-I 
Cucsa.094660  NPLVFRLKMANKIKA----IAKRLDEHYCAASIMGLVAIT-SKEVESE----PSQILETDSFLDEIGVIGREAEVL---------EIVNKLLELSK--QEAALSVLPIVGIGGLGKTSLAKAIFHHEM-I 
Cucsa.094670  TPLVFRLKMANKIKN----IAKMLERHYSAASTVGLVAIL-SKQTEPD----FSQIQETDSFLDEYGVIGRESEVL---------EIVNVSVDLSY--REN-LSVLPIVGMGGLGKTALAKVIFNHEL-I 
Cucsa.102240  LQMARDQKKNMKMMN-----------QMMEIICRLDNRGGSSKPMDLFVPPCLVPQLREE-------TVGLEKPVK---------ELKVKLLKNG-------VQMLVVTAPGGCGKTTLALKFCHDKE-V 
Cucsa.123410  QAHILADVHHMRFQTTE-RFDRLEGVLLERRLESMKIRADASGEERWWVEEAFKKAEEEERYESNFVNIGTGLRVG---------KRKLKELVIGK----EDLTAVGISGIGGSGKTTLAREFCKDPE-V 
Cucsa.128030  NQIAFNYRMARQIKN----IWERLDAIDAEKTQFHLRENCESRTQYGSFDRIMMGRETWSS-SNDEEVIGRDDDIK---------EVKERLLDMNM-NVTHNVSFIAIAGMGGIGKTTLAKSLYNDEE-V 
Cucsa.128100  NQIAFGFKMGQTIKK----VREKLDAIAAIKAQLHLSVCARE-VRDNEP---RKVRETSSFIPEGE-IIGRDEDRK---------SVMDFLLNTSN-ITKDNVEVVSIVGMGGLGKTALAQTVYNDEK-I 
Cucsa.128110  NQVSFGHKMSQKIKQ----VREKLDAIANDKTQLHLSVRMRE-TRDDEL---RKMRETCSFIPKGE-VIGRDDDKK---------AIIDFLLDT-N-TMEDNVEVVSIVGMGGLGKTAVAQSVYNDEK-I 
Cucsa.128130  NQLAFNYRMACQVKK----INERLDVISQEKDKFQLNGIAYLGIQNVLSYPIGMERDTHSSLSGDQKIIGRDDEMN---------NLKKNLLAEDD-KVKANVSFIAIVGMGGIGKTTLAKSLYNDKQ-V 
Cucsa.128140  --FKSNWKIDHKIKD----IRQRLQSINDDKNQFSFSEHVIE-KRDDEE--LRKRRETYSYILEEE-VIGRNDDKE---------VVIDLLLNSN---ITEDIAIVSIVGMGGLGKTALAQSIYTHHN-M 
Cucsa.132370  V-------FPNSLCT----IKPMLD--CFSLVVFVHLDNTTR-KIESE----VEQVEETTSLLE-NYVVGREMEVE---------SIVQDVTEASQ---QQLNSILPVYGTGGSGKTTLAQLVFNDER-I 
Cucsa.133510  FQTSRDGKKTLSLVT-----------EIKEVVRRLDSKSGLSNPVDLVVT---VPVISEE-------SVGLEKPVE---------KLKAKLFRDG-------VRLLVVTAPGGCGKSTLAEIFCHDKQ-V 
Cucsa.163670  NQIRFRLKMVHKIKK----IREKLDTIDKDKTQFNLFDNTRE-IRNDEM---TKRSETCSFILEGE-VIGRDDDKK---------CIVHFLLDTNI-IAKENIVVVAIIGMGGLGKTALAQSIYGDMK-E 
Cucsa.178360  ----------HRFSR---------RATKLAVAVDKAIQGG-SFERVGF----RVTPQEIMTLR-NNKKFEAFESRV---------LILKEIIEAVG---DANARVIVVHGMAGVGKTTLVEEIARLAK-E 
Cucsa.178620  --FITNWKIGHKIKE----ISQRLQNINEDKIQFSFCKHVIE-RRDDDDEGLRKRRETHSFILEDE-VIGRNDDKE---------AVIDLLLNSN---TKEDIAIVSIVGMPGFGKTALAQSIYNHKR-I 
Cucsa.189390  ----------HQLSR---------KAVKLAEEVVVIKIEG-NFDKVSS----PVALSEVESSKAKNSDFVDFESRK---------PTIDKIIAALM---DDNVHTIGVYGMGGVGKTMLVQEISKLAM-E 
Cucsa.237070  ----------YQMSK---------KAKKMVK-VISQIIEKRKLMFHQVGYPTPLSRIHGSSTSSSHGYDQILESRT---------SIAKQIRDALV---DCNVNKVGVYGMGGVEKTTLLKQVTPLVM-- 
Cucsa.239860  --------------------------------------------------------------------------------------------------------------MGGVGKTYLLKEVKKLVL-- 
Cucsa.248810  NVLIFRLDMAKKMMT----LVQLLEKHYNEAAPLGLVGIETVRPEIDV----ISQYRETISELEDHKIAGRDVEVE---------SIVKQVIDASN---NQRTSILPIVGMGGLGKTTLAKLVFNHEL-V 
Cucsa.251930  NPLMFRLKMARKIRT----ITQVLNEIKGEASAVGAIPTGGSDEIVAD----NGHIPETDSFLDEFEVVGRRADIS---------RIVNVVVDNAT--HER-ITVIPIVGMGGLGKTTLAKAVFNHEL-V 
Cucsa.277260  NSFLFGLKMAKKMKK----ITKTLYEHYCEASPLGLVGDESPTESEAA----LNQIRETTSILD-FEVEGREAEVL---------EILKLVIDSTD---EDHISVISIVGMGGLGKTTLAKMVFNHDA-I 
Cucsa.318890  -------------------------------------------------------------------------------------------------------------------------LVFNHEL-V 
Cucsa.326910  ----------YQLSR---------KAKKRVENIIELINEGNGFNKDNVGYPVPSPDTN---SPTLPTDYQIIASRT---------SIVEEIKEALA---NPNVDTVGVCGMGGVGKTALLNEVKKLVL-- 
Cucsa.328080  NQIAFRLKMSQKIKR----VREKLDAIAMDKTQFNLYENTRE-IQDDES---TKRLETTSFIREGE-IIGRDDDKK---------SIIHYLLDTN--IHEDSVAVIAIIGMGGLGKTALVQSIYGDEK-V 
Cucsa.337180  DQTKKMMKLCDDLDE----IASHMYGFNLTN-------METTHSFLSATEVSTRLMKPSWQLLYSLTNAPKVFQDK---------RYHNFLDHFKK----STHGLFHIVGEPGIGKTTLAKFFYNN---- 
Cucsa.337190  ARTWKMEKLFDHLNE----VAAKMYEFNLTERHTGAIKTETTNSFLTATEVSTRLMKPSWKVLYPLTNAPKFYQDE---------RYRKILNDFKN----PTLGFFHIVGEAGIGKSTLAKFIYNDPE-- 
Cucsa.338110  --------MAKKIKN----ITDTLNQHYCAASAFGLVGVETVTEIELA----LNQIRETTSILD-FQVEGREAEVL---------ELLKLAIDSTN---EHHMSVISIVGMGGLGKTTLAKMIFNHRE-I 
Cucsa.338190  NFLISPFNTGSKFQEDLKIITSELRSIEKAMSKFSLVEDEDEYIKKLKGEMTLRTSITGSHAFARLLRLRREAILSNVDSIFGRDKIQESIIKELVNDEQKSPRILSIQGDGGMGKTALAKLVYNADE-- 


                           270        280         290        300        310        320         330        340         350        360        370         380       390            
                 ....|....|....|....|....|....|....|....|....|....|....|....|....|....|....|....|....|....|....|....|....|....|....|....|....|....|
Cucsa.017460  KNH-FGITIWIYVSQPFVINNILQAILQKVEVHSSDCS-NNREA---LLEKLTENMGEKTYFLVLDDVWNENKMLWEKLKECLMSITHMSGNSILVTTRSSGIAKMMEENIGSH---------------- 
Cucsa.017490  EDRLFDRVIDVRVGRFNDVTDIQEQIGDQLNVELPK---SKEGR--ASFLRNNLAKMEGNILILLDDLWKEYDLLKEIGIPLSKD-----GCKVLITSRSQDILTNNMNTQECF---------------- 
Cucsa.088220  SK-SFDEVVTSTISQTPDFKSIQGQLADKLGLKFER--ETIEGR--APSLRKRLKMERRIL-VVLDDIWEYIDLETIGIPSVEDHTG----CKILFTSRNKHLISNQMCANQIF---------------- 
Cucsa.091880  SKKSFDKVVTSTISQTPDFKRIQGQLADKIGLKFEQ--ETIEGR--ATFLRRWLKAERR-------------------IPSVEDHKGI---CKILFTSRNKQLISNDMGANKIF---------------- 
Cucsa.094560  KGH-FDETIWICVSEPFLINKILGAILQMIKGVSSGLDN--REA---LLRELQKVMRGKRYFLVLDDVWNENLALWTELKHCLLSFTEKSGNAIIVTTRSFEVGKIMESTLSSH---------------- 
Cucsa.094580  KRH-FDETIWICVSEPFLINKILGAILQMIKGVSSGLDN--KEV---LLQELQKVMRGKRYFLVLDDVWNENIALWTELKKCLLCFTEKSGNGIIVTTRSIEVGKIMESTLPSH---------------- 
Cucsa.094650  KKH-FHETIWICVSEHFNIDEILVAILESLTDKVP---TK-REA---VLRRLQKELLDKRCFLVLDDVWNESSKLWEELEDCLKEIVGKFGITIIVTTRLDEVANIMGT-VSGY---------------- 
Cucsa.094660  REN-FDRMIWVCVSEPFVINKILRAILETLNANFGGLDN--KEA---LLQELQKLLRNKKYFLVLDDVWNENPDLWNELRACLLKANKKFGSVIVVTTRSDEVANIVETNHQRH---------------- 
Cucsa.094670  KGN-FDRAVWVCVSEPFLIKKILRAILETLNSHFGGLDS--KEA---LLQELQKLLNDKKYFLVLDDVWNENPILWNELKGCLLKISQRSGNVVVVTTRSDRVAEIMET-HSRY---------------- 
Cucsa.102240  KDIFQ-EKIFVPVSRKPDLKLILKDIIESLRGIQLPDLQSDERAFCYLELWLKQTSVNRPVLIVLDDVWSGQ-----ESEVLLDKLFQLPCCKILVTSRFYFPRFS-ESYYLEP---------------- 
Cucsa.123410  RRHFKERILFLTVSQSPDVEQLRRTIWEFVMGSDSVNSNN----------LILHGRPSNSALLVLDDVWS--------ISVLENVIPNVTGCKTLVVSRFKFPEVLRETYEVEL---------------- 
Cucsa.128030  SGF-FDLKIWVWVSDQFEVQVVAEKMIESATKNNPS--VKGMEA---LQAKLQKVIGERKYLLVMDDVWNESEEKWHGLKSLLMGGAR--GSKVLITKRDRKVATEIKSMTSLF---------------- 
Cucsa.128100  NNR-FKWKIWVCISQEFDIKVIVEKILESITKTKQE--SLQLDI---LQSMLQEKIYGKKYLLVMDDVWNVDHEKWIGLKRFLMGGAS--GSKILVTTRNLQTAQASDTVWFH----------------- 
Cucsa.128110  NEH-FKLKLWVCISQEFDIKVIVEKIIEFIAKKKPD--SLQLDI---LQSMLQEKIDGKKYLLVMDDVWNESHETWVSLKRFLMGGAK--GSRILITTRNLQVAQASDTVQFH----------------- 
Cucsa.128130  SDG-FSSRIWIWVSNQFDTKTILKKIIESATEKKPK--VEEMEP---LKTKLEEVIGGKKYLLVMDDVWNENENEWENLKNLLMLGAR--GSKVLITKRDSKAVPGVETIP------------------- 
Cucsa.128140  TNSGFELKLWVCVSEEFDLKVIIQKMIESATGTKPKP-YLQIDS---LQSELRKKIDGKKYLFVMDDVWNEKKEEWLRLKRLLMGGAK--GSRILITTRSEQVAKTFDSTFIH----------------- 
Cucsa.132370  GKQ-FHHTVWVCVSQPFVINEILQSILKKVSKSNDNRSKDDKDT---LIRNLKEVMGGKRYFLVLDNVWNENKIFWEKLKECLMSIVEELGSSVLVTTRSRKIAEMMKETLDTY---------------- 
Cucsa.133510  KNKFQRNILFLVVSSKPETKRILISIIQRLGGPIESGSVSDDEAFRLLEVRVGELSPN-PVLIVLDDVWDGS-----ESNKLLEKFSRLPNCKVLVTSRFKFPAFG-ESYDLEP---------------- 
Cucsa.163670  NKH-FELTMWVCISEEFDVKVIVEKIIESLTKKRPKP-NLTLDT---LQSMLREKIDGKKYLLVMDDVWNDERTKWINLKKFLMGGAK--GSRILITTRTHQVAHIFDTDLFH----------------- 
Cucsa.178360  GK-LFDAIAMVTVKHIPNIKKIQGEIADQLGLKFEE--EKERIR--ADRLRRRLEMEKK-VLVVLDDVWSR---LDLEAVGISSHHKG---CKILVTSRKDDLFFNDFGTQKNI---------------- 
Cucsa.178620  MTQ-FQLKIWVCVSDEFDLKITIQKIIESATGKKPKS-FLQMDP---LQCELRKQIDGKKYLIVMDDVWNEKKEKWLHLKRLLMGGAK--GSRILITTRSEQVAKTFDSTFVH----------------- 
Cucsa.189390  QK-LFDEVITSTVSQTPDLRRIQGQLGDKLGLRFEQ--ETEEGR--ALKLLNRLKMERQKILIVLDDVWKQIDLEKIGIPSIEDHSG----CKILFTSRDNDVLFNDWRTYKNF---------------- 
Cucsa.237070  EEKLFDHVIIVNVGQTLGVEGIQAQIGDKLRLELNKKVESKEGR--ASLLQN-KLEMESNVLLVLDDLWKGLD-LEEVGIPCRSESCEK-GCKILITSRDRDVLTNEMDTQVYF---------------- 
Cucsa.239860  EEKLFDLVIDVTVGQSNDVMNMQQQIGDFLNKELPK---SKEGR--TSFLRNALVEMKGNILITFDDLWNEFDIINDVGIPLSKE-----GCKTLVTSRFQNVLANKMNIKECF---------------- 
Cucsa.248810  RQR-FDKTVWVCVSEPFIVNKILLDILKNVKGAYISDGRDSKEV---LLRELQKEMLGQSYFLVLDDVWNETFFLWDDLKYCLLKITGNSNNSILVTTRSAEVAKIMGT-CPSH---------------- 
Cucsa.251930  IAH-FDETIWVCVTATFDEKKILRAILESLTNFPSGLDS--KDA---ILRRLQKELEGKRYFLVLDDVWNENVKLWNNFKSLLLKITNSIGNRVLVTTRSEEAGKIMET-FPSH---------------- 
Cucsa.277260  KGH-FDKTVWVCVSKPFIVMKILEAIFQGLTNTSSGLNS--REA---LLNRLREEMQGKKYFLVLDDVWDKENCLWDELIGNLKYIAGKSGNSIMVTTRSVEVATMVKT-VPIY---------------- 
Cucsa.318890  RQH-FDKTVWVCVSEPFIVNKILLDILQNLKGT-ISNGGDSKEV---LLRELQKKMHGQRYFLVLDDVWNENSFLWDELKYCLLKITGNSKNSIVVTTRSAEVAKIMGT-CSGH---------------- 
Cucsa.326910  EKNLFDRVIQVEVGESKSVFNIQEQIKDELNMELNI--ECEEVR--ACRLRTHIAERKENMLFMLDDIWKEHDVEKEFGIP------------------FKHVLNNEMNTEKTF---------------- 
Cucsa.328080  KKH-FELTMWVCISEEFDVKVIIEKIIESLTKKKREP-DLQLDT---LQSMVREKIDGKRYLLVMDDVWNVNRAKWISLKRYLMGGAK--GSRILITTRTHQVAQTFETILSH----------------- 
Cucsa.337180  LVNTFPSRLWICVKEEFDPQRLIKEMLS-FSHCQVTCDNLTEKQ---LCFAVQQFLRDKKFLIVFQDISIKNLGNCSIFKSLLGMGNR--GSKIIVTTQNEKIADAVGLKKLYK-------NESQVVPSP 
Cucsa.337190  VEGMFPSRLWVCVKEEFDTQRLMKEILN-FSYSPATCDNLTTK-----LCPTDQYLRERTFLLVFQDLSIKNLDNCSLFTSLLMMGKP--GSKIIVTTQNEEIANAIELTMIYKVGQQSEQNRSQTALDT 
Cucsa.338110  EGH-FDKTIWVCVSKPFIVTKILEKIFQGLTKTCSGLESN-KEA---LLGRLRKEMQDKNYFLVLDDVWDNEKHLWDELRGCLKHIAGKPGNTIVMTTRNEEVATMVEP-ISIY---------------- 
Cucsa.338190  VFDHFDKRMWVCVSEDFDIRRILREVLMSATGENVTTVALTESR---LRIRLQRYFFGKKILLVLDDFGNLDPERVSELKKIVKMGVG--GSKIMITTRSDETLNVATTHKIDK---------------- 

  

                        400        410        420         430        440        450         460        470         480        490         500        510       520            
                ....|....|....|....|....|....|....|....|....|....|....|....|....|....|....|....|....|....|....|....|....|....|....|....|....|....|
Cucsa.017460  ---------------------------------ELRKLSDDQCWSIFRNFANAKDVP--------MTSNLE-FVQKEFDKRIGGLPLIAKVLGAAVPFSGDHDQ--------WVANIKSVLTTPIKEEEF 
Cucsa.017490  ---------------------------------QVSSLSEEESWKFFMAIIGD---KFDTI--------YKKNIAKNVAKECGGLPLALDTIAKALKG--KDMHHWE-DALTKLR--NSIGMDIKGVSDK 
Cucsa.088220  ---------------------------------EIKVLGENESWNLFKAMAGKIVEASD-----------LKPIAIQVVRECAGLPIAITTVAKALRN--KPSDIWN-DALDQLKSVDVFMTNIGEMDKK 
Cucsa.091880  ---------------------------------EIKVLGEDESWNLFKAMAGEIVEATD-----------LKPIAIQIMRECAGLPIAITTVAKALLN--KPSDIWN-DALDQLKSVDVGMANIGEMDKK 
Cucsa.094560  ---------------------------------HLGKLSDEQCWSLFKKSANADELP--------KNLELK-DLQEELVTRFGGAPLVARMK-------------------------------------- 
Cucsa.094580  ---------------------------------HLGKLFDEQCRSLFKESANADELP--------MDPELK-DLQEELVTRFGGVPFVAR---------------------------------------- 
Cucsa.094650  ---------------------------------RLEKLPEDHCWSLFKRSANANGVK--------MTPKLE-AIRIKLLQKIDGIPLVAKVLGGAVEFEGDLDR-------WETTLESIVREIPMKQKSY 
Cucsa.094660  ---------------------------------RLRKLSNDYCWTLFEKCAFGSDLP--------VTPRVDHVIREELVKRFGGIPLVVKVFGGMVKLDKNKCC------QGLRSTLENLIISPLQYENS 
Cucsa.094670  ---------------------------------HLTKLSDDHCWSLFKKYAFGNELL--------RIPELD-IVQKELVKRFGGIPLAVKVMGGIVKFDEN--H------EGLQKSLENLMRLQLQDENH 
Cucsa.102240  -------------------------------------LNHENAVQLFRRAASLDKGIS---------KLPDDETVEKIIGGCKRLPLALKVIGRSLSHKPTSVWK-----VTGRNLARSGSIFDSDNELL 
Cucsa.123410  -------------------------------------LKESEAIALFCHSAFGQQSIP---------LSANHNLVKQVVNECKCLPLALKVIGASLRGQSEMFWN-----NAKSRLSRGEPICES--HEN 
Cucsa.128030  ---------------------------------TLEGLSESNSWLLFSKVAF-KEG-------KESTDPSTIHLGKEILVRCGGVPLVIRHVGRMLYSKTSQEE----WMSFKDNELLEVIQQDND---- 
Cucsa.128100  ---------------------------------HLKELDKDNSWALFRKMAFLNKEE-------ELENSNLVRIGKEIVAKLKGYPLSIRVVGRLLYFKNTEMD----WSSFKDNELDSILQEDD----Q 
Cucsa.128110  ---------------------------------HLKELDNESSWALFRKMAFLNEEE-------EIENSNKVRIGKEIIAKLKGSPLTIRIVGRLLYFKNTEMD----WLSFKDNDLGTILQQEN----Q 
Cucsa.128130  ----------------------------------LKDLTEDFSWLLFKEVAF-KESD------LESINQNLIKMGKEISKRCGGIPLVIRHIGRLLYGKTSAED----WEFIKENELLNVTREKNNNDGH 
Cucsa.128140  ---------------------------------FLQILDEYNSWLLFQKITCLEGHPSNPE-KLDQ-SSSLIQIGREIVSKLKGVPLTIRTIGGLLKDNKSKRV----WLSFKDNELHRILGQGQDNLKE 
Cucsa.132370  ---------------------------------HLNKLTDDQCWSVFSYFAKANAVP--------ITSNLE-LVREE----------------------------------------------------- 
Cucsa.133510  -------------------------------------LDHKDAMELFRRWASRGNRVL---------QFPDERIVEKIVRGCKRFPLALKVIAGSLSGRATSVWE-----VTGRKLSRGDSILGSEKELQ 
Cucsa.163670  ---------------------------------DLSELDKDNSWELFRKMAFSNESE-------MLENSKLVGIGKEIVTKLKGSPLAIRVIGSYLYSKKSEKD----WLSFKENELDTIMQQEN----E 
Cucsa.178360  ---------------------------------YINILSKKEARDFFNKVACDSVESSDDTD------PEMEAVATELADECGGLPLSLATVGQALKG--KGLPSWN-DALQGMKFPGEPSN--YGVNKV 
Cucsa.178620  ---------------------------------LLQILDASNSWLLFQKMIGLEEHSDNQEVELDQKNSNLIQIGMEIVSTLRGVPLLIRTIGGLLKDNKSERF----WLSFKDKELYQVLGRGQDALKE 
Cucsa.189390  ---------------------------------EIKFLQEDETWNLFRKMAGEIVETSD-----------FKSIAVEIVRECAHLPIAITTIARALRN--KPASIWK-DALIQLR--NPVFVNIREINKK 
Cucsa.237070  ---------------------------------EVKPLSEKESWEFFKNMIG----EFDNK--------CIELIGKEMVKKCGGLPIALATIVKTLKG--KEVPIWK-DALKQLK--NPIAVDVK----- 
Cucsa.239860  ---------------------------------KVTCLDDEESWKFFKKIIGD---EFDAK--------MEN-IAKEVAKQCGGLPLALDIIAKTLKRSRHINYYWE-GVLSKLK--NSIPVNID-VGEK 
Cucsa.248810  ---------------------------------LLSKLSDDQCWSLFKESANAYGLS--------MTSNLG-IIQKELVKKIGGVPLAARVLGRAVKFEGDVER--------WEEMLKNVLTTPLQEENF 
Cucsa.251930  ---------------------------------HVEKLSDDECWSIFKERASANGLP--------LTPELE-VIKNVLAEQFGGIPLVAKVLGGAVQFKKR-TE------TWLMSTLETLIMNPLQNEND 
Cucsa.277260  ---------------------------------HLKKLSDDHCWALLKKSANANQLQ--------MNSKLE-NTKNILVRKIGGVPLIAKVLGGAVKFEEGGSE------SWMAKIESFARNISIEDKDF 
Cucsa.318890  ---------------------------------LLSKLSDDHCWSLFKESANAYGLS--------MTSNLE-IIQKELVKKIGGIPLAARVLGRAVKFEGDVER--------WEEMLKNVLSTPLKEENF 
Cucsa.326910  ---------------------------------EVNSLTNEESRNFFVTIVGESSCVEDGH--------NIQQIAEDVVKECGGLPLALKILGKALKG--KRVQIWK-DALKSLK--NPVTVTISGVSEQ 
Cucsa.328080  ---------------------------------HLKELDEEKSWKLFRKMAFSNESE-------VLENSKLVVIGKEIVTKLKGSPLAIRVIGSYLYSKKSEKD----WLSFKDHELDTIMQQEN----E 
Cucsa.337180  EATKPSDVN--------------KDNMKHQTIFKVERLSKENSLSLFKVHAFTETQEAQ--------IPNLTKIQEVIEQKCHGVPLAIKCLGGLLSKTSIAEW-------NGVIDKLWEHEEEEDGNKS 
Cucsa.337190  VTKETANVNNADQFVQANPLGKIDQSIPSQTIFKVKRLSEKDSLSLFKDYAS--TYEGN--------EKDIMKT----LKKCNGIPLAIKCLGSMLSLGPPATK-------WMEDN---ERQKGDNESSS 
Cucsa.338110  ---------------------------------RLKKLSNDQCWALFKESANANQLP--------MNSKLE-IMKKELVKKMGGVPLVAKVLGGAVKFEETELEEEDHEISWMTKVESIVRNISLEDKDF 
Cucsa.338190  -------------------------------------LDETISMQIFEDTYGSEGLSEG---------LRDDLYLKNLVAECGGAPLAIKCLAGLLSSKPSDGAKSP----NVKDLSEKWKQEEANNGGG 


                          530        540         550        560        570         580        590        600        610         620         630        640       650            
                ....|....|....|....|....|....|....|....|....|....|....|....|....|....|....|....|....|....|....|....|....|....|....|....|....|....|
Cucsa.017460  VKFTLKLSVDRLPNASVKQCFAYCSNFSKG--CEFDKKQVIRMWMAQGFTQPDERNN--ETMEDTGERYFNILLSFCLFQDVVK------------NERGIIEKVRMHDLIHDIACQVSNDKKLRIDHI- 
Cucsa.017490  VYASLRLSYDHLDGEETKLIFLLCSVFPDD--YKISIKNLQMYAMCMRLLNKVKTWE---DSKNRVMKLVNDLISSSLLLEAES-------------D-SKDKYVKMHDVVRDVAIHIASKEGNMSTLN- 
Cucsa.088220  VYLSLKLSYDCLGYEEVKLLFLLCSMFPED--FSIDMEELHVYAMGMGFLHGVDTVV---KGRRRIKKLVDDLISSSLLQQYS--------------EYG-YNYVKMHDMVRDVAIFIASKNDHIRTLS- 
Cucsa.091880  VYLSLKLSYDYLGYEEVKLLFLLCSMFPED--FNIDVEKLHVYAMSMGFLRGVDTVV---KGRRRIKKLVDDLISSSLLQQYS--------------EYG-NNYVKIHDMVRDVAILIASQNDHIRTLS- 
Cucsa.094560  ---------------------------------------------------------------------------------------------------------------------------------- 
Cucsa.094580  ---------------------------------------------------------------------------------------------------------------------------------- 
Cucsa.094650  VLSILQLSVDRLP-FVEKQCFAYCSIFPKD--CEVVKENLIRMWIAQGFIQPTEGEN---TMEDLGEGHFNFLLSRSLFQDVVK------------DKYGRITHFKMHDLIHDVALAILSTRQKSVL--- 
Cucsa.094660  ILSTIKLSVDRLPSSSLKQCFAYCSNFPRG--FLFIREPLVQMWIAQGFIHLPSGSN--VTMEDIGANYFNTLLSRSLFQDVVK------------DDRERILYCKMHDVVHDVACAISNAQKLRLS--- 
Cucsa.094670  VVSTIKLTVDRLPLPSLKQCFAYCSNFPKD--FKFRKEALIQMWIAQGFIQPSLGSD--EMMEDIGEKYFNVLLSRFLFQDIVK------------DNRGRIIFCKMHDLIHDVACAISNSPGLKWDPSD 
Cucsa.102240  ECLQSSLDVL-DDNMVTKKSFMDLGSFHED--QRISASTFIDMCTVLYTLDES------EAMVTLDELSSRSLVNFVTARKYGY-----------DDDFYEEYSFTQHDILRDLAIHLMNME-------- 
Cucsa.123410  KLLQRMAISIERLSSKVRECFLDLGCFPED--KRIPLDILINVWKELHDLDDE------EALAVLFELSQKNLLTLVKDARGGD-----------IYSSYYEMYVTQHDVLRDLALHFSCQE-------- 
Cucsa.128030  MTSILKLSYNHLP-PNLKRCFAYSSLFPKG--YKIEIKDLIRQWVAQGFIEVS---NGRKSLEDTGKDYFNELCWR-FFYANSS------------DECNINDIVCMHDVMCEFVRKVAGNKLYVRGNP- 
Cucsa.128100  IQPILKISFNHLP-PKLKQCFTYCALFPKD--YEFKKNGLVKQWMAQGFIQA----HNKKAIEDVGDDYFQELVGRSFFQDIRK------------NKWGDLKYCKMHDLLHDLACSIGENECVVVS--- 
Cucsa.128110  IQPILKISFNHLP-SNLKHCFTYCALFPKD--YEFQKDGLVKQWMAQGFIQS----HSNKEIEDVGDDYFKELLGRSFFHNVKV------------NKWGDVKECKMHDLIHDLACWIVENECVDAS--- 
Cucsa.128130  VISTLKLSYNHLS-PNLKQCFSYSSLFPKG--YKIRMNELIRQWIAQGFIESS---NGGKSVENIGKEYLDELCWR-FFYEISI------------EDVPFEEVG-MHDLMCDLAREVAGQKLYIRGYP- 
Cucsa.128140  VRLILELSYKYLP-ANLKQCFLYCALFPKD--YEIKTHELILMWSAQGFIQPNGSKDN--SLIDIGNDYFMELLSRSFFQEVTK------------NERGDIIACKMHDLMHDLACWIADNECN------ 
Cucsa.132370  ------LSVDRLPKASIKQCFAYCSNFPKG--YWFDKKQVIKMWMAHGFTRPDEGNN--ETMEDTGERYFNILLSYCLFQDADD------------DKWHIGRKFRMHDLIHDIACDVSSDKRLQLDHS- 
Cucsa.133510  KCLKDTLDAIPDDKIVLKECFMDLGSFPED--QRIRAATFIDICAVLYEQDEC------ETMSNLDELFTRTLVNTVSLRNKAH-----------EDDYYSESYITQHDVLRELAVLLTNEQ-------- 
Cucsa.163670  IQSILKISFNHLS-SSLKQCITYCALFPKD--FEIDKDDLIKQWMGEGFIQP----HNKKAMEDVGDEYFKELLGRSFFQDISK------------NQLGEIMKFKMHDFMHDLACFVGENDYVFAT--- 
Cucsa.178360  AYLSLKVSYRSLNREEARSLFLLCSLFPED--YQINIKYLLMYAMGLGLLNAMSSLA---MAKWRILSLVDELKTSHLLLDGV--------------DND---FVKMHDIVRDTAILIASKMKSKYLVR- 
Cucsa.178620  IQLFLELSYKYLPSSNLKQCFLYCALFPKD--YRIKKDELILLWRAQGFIQQNGNNDDNSSLVDIGEDYFMELLSRSFFQEVEK------------NDFGDIITCKMHDLMHDLACSITNNECVRG---- 
Cucsa.189390  VYSSLKLSYDYLDSEEAKSLFLLCSMFPED--YIIDCQVLHVYAMGMGLLHGVESVA---QARNRITKLVDDLISSSLLLKES--------------NVDLVMYVKMHDIVRDVAIIIASKDDRIFTLS- 
Cucsa.237070  -------------G--------VTDVFPDD--YEISVEDLQIYAMSLRLLNQVNTWD---EARNRVIKLVDDLKASSLLLESN----------------SRDNHVKMHDIVRDVAIYIASKEANMSTLS- 
Cucsa.239860  VYASLKLSYEHLDGEEVKSLFLLCSVFPDD--HGISVNDLQMYVMGMGLLKMVNTWK---EARAEAHYLVEDLTSSSLLQRLK------------------NRDVKMHDIVRDVAIYIG-PDFNMSTLY- 
Cucsa.248810  VLSILKLSVDRLPSSSVKQCFAYCSIFPKD--FVFEKQELIQMWMAQGFLQPQQGRYNNTAMENVGDIYFNILLSRCLFEFEDANKTRIRDMIG--DYETR-EEYKMHDLVHDIAMETSRSYKDLHLNP- 
Cucsa.251930  VSSILRLSVDHLPNSSLKQCFAYFSNFPKG--FNFEKEQLIQFWMAEGFIQPSDKVNP-ETMEDIGDKYFNILLARSLFQDIVK------------DENGKITHCKMHHLLHDLAYSVSKCEALGSNLNG 
Cucsa.277260  VLSILKLSVESLPHSALKQCFAYCSNFPQD--YEFDKDEAIQMWIAEGFIQPEQERE-NLTMENIGEEYLNFLLSRSLFEDAIK------------YDG-RIVTFKIHDLMHDIACAISN-HHKMDS--- 
Cucsa.318890  ILSILKLSVDRLPSSALKQCFSYCSIFPKD--FVFEKQELIHMWMAQGFLQPQEGR--NMTMETVGDIYFKILLSHCLFEDAHETKTEEYEIPDLLEFETRPEEYKMHDLVHDIAIEISRD-QNLQLNP- 
Cucsa.326910  LYSCLQFSYDS-TEDEAEQVLLLCSVFPDD--YKIEVKDLQMYAMGMGLVKHINTWE---DAGNRVIKLVDDLKSCYLLQDEQS-------------KKGSDDCVQMHDVVHDFAKYVASKKDKMTSLT- 
Cucsa.328080  IQSILKISFNHLS-SSLKHCFTYCALFSKDYHYEIRKNDLIKQWMAQGFIQP----HNKKAMEDVGDDYFEELLGRSFFQDIRK------------NKWGEIKKFKMHDIIHDLACSVVENDCVLAN--- 
Cucsa.337180  ILPTLRLCYDQMP-SHLQRCFLYCSQLKKD--RILSSNDVIQLWIASDLLP----KENYLSLEKIGENYFKELCSRCFLQELEE--------------YGFGYWFKLHPLIEKLARLLTQK--------- 
Cucsa.337190  TFSILKLCYNEMP-SHLKRCFLYCSQLPND--SILSSNDVIQLWMANGLLRSR--QENYLSLEDIGEIYFKELCSRCFLQDVEE--------------YGLGYWFKMHPLIRELARLVQKRTKDLI---- 
Cucsa.338110  VLSILKLSVDSLPNPVLKQCVAYCSNFSQD--YDFQKDDLIKMWIAQGFIQPGQGRDKNLLMEDIGEQYFNFLLSRSIFQDVTR------------DANKRIVGFKMHDLMHDIACAISS-HQNVES--- 
Cucsa.338190  VLCALRLSYDLMP-SYLKPCFLCFSVLPKD--NVFFSFELIQLWMAQGILPSGTKDN----PEEVGEKYFKEFRDRRLLVDVEE--------------HTLGYWFKIHSLVHDLAVQKATEQKNLGNFHM 


                          660        670        680         690        700         710        720        730        740         750         760        770       780            
                ....|....|....|....|....|....|....|....|....|....|....|....|....|....|....|....|....|....|....|....|....|....|....|....|....|....|
Cucsa.017460  ---------ISSNWKDWTKDDKILVSKLRTINFYDRHHVVVQDKIGDFTGLRVLTIENYI--------------------------------VEELPNSIFKLKHLRYLDISYCYSIKKLPESIVLLYNL 
Cucsa.017490  --------IGYNKVNEWE---DECRSGSHRAIFANCDNLNNLPLKMNFPQLELLILRVS---YWLVEDNLQIPYAFFDGMVKLKVLDLTGMCCLRPL-WTTPS-LNNLQALCMLRCEFNDIDTIGELKKL 
Cucsa.088220  --------YVK-RLDEEWK--EERLLGNHTVVS--IHGLHYP-LPKLMLPKVQLLRLDG---QWLNNTYVSVVQTFFEEMKELKGLVLEKMNISLLQRPFDLYFLANIRVLRLRGCELGSIDMIGELKRL 
Cucsa.091880  --------YVK-RSNEEWK--EEKLSGNHTVVFLIIQELDSPDFSKLMLPKVQLFVLFGPSPSIYNRHVVSVVETFYKEMKELEGLVIERVKISLS--PQALYSFANLRLLRLHDCELGSIDMIGELKKL 
Cucsa.094560  --------------------------------------------------------------------------------------------------------HLRYLDISNSKI-EELPNSISLLYNL 
Cucsa.094580  ---------------------------------------------------------------------------------------------------------------------------------- 
Cucsa.094650  -----------------DPTHWNGKTSRKLRTLLYNNQEIHHK-VADCVFLRVLEVNSLHM-------------------------------MNNLPDFIAKLKHLRYLDISSCSM-WVMPHSVTTLFNL 
Cucsa.094660  ---GKSNGDKALSIGHEIRTLHCSENVVERFHLPTFDSHVFHNEISNFTYLCVLII-HSWF-------------------------------IHQLPDSIAKLKHLRYLDISHSLI-RTLPDSIVSLYNL 
Cucsa.094670  LFDGEPWRRQACFASLELKTPDCNENPSRKLHMLTFDSHVFHNKVTNFLYLRVLIT-HSWF-------------------------------ICKLPNSIAKLKHLRYLDISYSTI-RELPDSAVLLYNL 
Cucsa.102240  ------------------PIEQRKRLILDINGNDLPKWWVDQEKHTSYARLISITTDKRFS----------------------------------------------------------ASWPDMEAPEV 
Cucsa.123410  ------------------NVNDRKRLLMPKSDTELPKEWLRKSEQPFNAQLVSIHTGEMEE----------------------------------------------------------MDWAPMIFPEA 
Cucsa.128030  --------------NNDYVVSEQTLHISFDYG--IQSWQDVLSKLCKAKGLRTILLLFR-PYEKMNKIDKAILDELFSSFPRLRVLDLHFSQISVVPKSIKKLRHLRYLDLSENDMELIPHSIIELQNLQ 
Cucsa.128100  --------------DDVGSIDKRTRHASFLLS-KRLTREVVSKSSIEVTSLRTLDIDSRASFRSF-------KKTCHMNLFQLRTLNLDRCCCHP-PKFVDKLKHLRYLNLSGLNVTFLPNSITTLYNLE 
Cucsa.128110  --------------DKTKSIDKRTRHVSFPSNYSRKSWELEAKSLTEVKNLRTLHGPP---------------FLLSENHLRLRSLNLGYSKFQKIPKFISQLRHLRYLDISDHDMKFLPKFITKLYNLE 
Cucsa.128130  --------------ESGYVVSEQTRHISFEYE--PRSWIDDVSKLQQAKGLRTFLLFTKNPFFTRNPIEKVLLDRLFSHFPRLRVL-----QIPNVSKSIKKLRHLRYLELG-EDAKSVPNSITKLQNLQ 
Cucsa.128140  ------------------VINIGTRHFAWKDQYSHKDQLLR--SLSKVTNLRTFFMLDSAN------------------------------------------------DLKWEFTKILHD--------- 
Cucsa.132370  ---------SSSKWKGLTEEKKKIESKLRTVIDFGRN-----GKIKDFVCLRVLTIAEN---------------------------------VRELPNSISKLKHLRYLDISRCYSIKKLPESIVGHLEI 
Cucsa.133510  ------------------PVDQRTRLLVDINKNEFPKWWSVRQMQPVKARLLSITTDEKFS----------------------------------------------------------SCWPDMEAPEV 
Cucsa.163670  --------------DDTKFIDKRTRHLSISPFISKTRWEVIKESLIAAKNLRTLNYACHNYDGDE-------IEIDFSNHLRLRTLNLIFSTHVP--KCIGKMKHLRYINFTRCYFDFLPKVVTKLYHLE 
Cucsa.178360  --------HGA--GESLWP--PMDEFKDYTAIS--LGCSDHSELPEFICPQLRFLLLVG------KRTSLRLPEKFFAGMQELRVLDLTGLCIQRLP--PSIDQLVNLQTLCLDDCVLPDMSVVGELKKL 
Cucsa.178620  --------------LKGNVIDKRTHHLSF-EKVSHEDQLMG--SLSKATHLRTLFSQDVHSRCN--------LEETFHNIFQLRTLHLNSYGPPK-------------CAKTLEFIKFLPDSITKLYKLE 
Cucsa.189390  --------YSKGLLDESWD--EKKLVGKHTAVCLNVKGLHNL-PQKLMLPKVQLLVFCG---TLLGEHELP--GTFFEEMKGMRVLEIRSMKMPLLS--PSLYSLTNLQSLHLFDCELENIDVICELNKL 
Cucsa.237070  --------YGFG-LSEWQ---EKDRHGFYRAIFGNCHNFYNFPQNLEFPKLELLILDGH---DWRGEK-LQICYSFFEGMKELKVLNLSRMCFQLLR-RPSIHSLENLQTLCMSHCTFNDIDAISHLKKL 
Cucsa.239860  --------YGYSTSSKGL---DEDKCRSYRAIFVDCKKFCNLLPNLKLPKLELLILSFP---FWGKDRNIDIMDAYFEGMENLKVLDIEGTSFLQPF-WTP---LKNLRTLCMSYCWCEDIDTIGHLKQL 
Cucsa.248810  ---SNISKKELQKEMINVAGKLRTIDFIQKIPHNIDQ-TLFDVEIRNFVCLRVLKIS-----------------------------------GDKLPKSIGQLKHLRYLEILSYSIELKLPESIVSLHNL 
Cucsa.251930  LVDDVPQIRRLSLIGCEQNVTLPPRRSMVKLRSLFLDRDVFGHKILDFKRLRVLNM-SLCE-------------------------------IQNLPTSIGRLKHLRYLDVSNNMI-KKLPKSIVKLYKL 
Cucsa.277260  -----------------NPISWNGKSTRKLRTLICENEEAFHKIQTDIICLRVLVL-KWFD-------------------------------TNTLSTIMAKLIHLRYLDISNCNINKLLRDSICALYNL 
Cucsa.318890  ---SNISKKELQKEIKKVACKLRMVDFIRRIPCNIGQLTFFDVEIRNFVCLRVLKLSTL-P-------------------------------SDKLPKSIGQLKHLRYLEIACYLGRLKFPESIVSLHNL 
Cucsa.326910  --------YRSGQRLEYWQEEDDDMHESYKAIYADCAKYCVYLP---------PKVGVS---EPSIGNKIRIPTAFFERMKALRVLSVETMSISFE---------------PSSWASINNLEALYELIKL 
Cucsa.328080  --------------DDTKSIDKRTRHVSISAFNSMTRWKLITKSLIEAKNLRTLNYARRHH-------------IDLSNHLRLRTLNLEFH-FVP--KCIGKMKHLRYINITYCYIDFLPKAVTKLYHLE 
Cucsa.337180  ---------------QVFEVTKTQSIAFTIRDKVPPSAFLANACIDKFKYLRLLHLGNAN--------------------------------LQGIPSAVENLVQLRYLDLQGNKKIKRLPNSIFKLKNL 
Cucsa.337190  ---------------SIKPVTNVTSIAFPVRDEVPSSSFLAEKCISKFQHLRLLYLGHTD--------------------------------LQEIPNTIETLNHLTYLDLQGNKNIKRLPNAICNLQHL 
Cucsa.338110  -----------------NPNNLSGKSVRKLRTLICNDEVINYLNQKDIVCLRVLKV-IFQS-------------------------------HTDLWIPIDKLIHLRYLDISECSINKLLLESLSLLYNL 
Cucsa.338190  LS----FVDCDSIPSSTNYDNTRFISIPVVGGAGPNINSDLFKCITQFRQLRFLYLCN--------------------------------SSLEEIPTSIDTLKHLRCLDLRGSQRLKRLPESICKLQSL 

                          790        800        810         820        830         840        850        860         870        880         890        900       910            
                ....|....|....|....|....|....|....|....|....|....|....|....|....|....|....|....|....|....|....|....|....|....|....|....|....|....|
Cucsa.017460  QTLRFHLL-SKGFLPKNVGQMISLRHLEFSSI---DKQMSPYLSQLIQLETLPKFAVGFEKGCKITELGVLRNLKG-LLKLQRLEHVES-----KEEAETAKLVEKENLEEVHFVWT------------- 
Cucsa.017490  EVLRIVKCNMLDHLPPTMSQLTHLKVLEVL-NCPKLEVVPANIFSSMTKLEELKLQDSFCRWGEEVWYKDRLVKNVTVSELNCLPCLSNLSLESWNVKILSEISSQTCKKLKEFWICSNESDDFIQPKVS 
Cucsa.088220  EILDLSGSN-IIQIPTTMGQLTQLKVLNLSNCFNKLEIIPPNILSKLTKLEELRMGTFGSWEGEEWYEGRKNASLSELRFLPHLFDLDLTIQDEKIMPKHLFSAEELNLEKFHITIGCKRERVKNYDGII 
Cucsa.091880  EILDFSKSN-IVEIPMTFSKLTQLKVLNLS-FCDELEVIPPNILSKLTKLEELHLETFDSWEGEEWYEGRKNASLSELRYLPHLYALNLTIQDDEIMPKHLFLAGELNLENFHITIGCQRQ-KRHID--- 
Cucsa.094560  QTLKLGS--SMKDLPQNLSKLVSLRHLKFSMP-----QTPPHLGRLTQLQTLSGFAVGFEKGFKIGELGFLKNLKG-RLELSNLDRIKH-----KEEAMSSKLVEK-NLCELFLEWDMHILR--EGN--N 
Cucsa.094580  ----LGS--SMKHLPYNLSKLVSLRHLKFSIP-----QTPPHLSRLTQLQTLSGFAVGFEKGCKIEELGFLKNFKG-RLELSNLNGIKH-----KEEAMSSKLVEK-NLCELFLEWDLHILR--EGS--N 
Cucsa.094650  QTLKLG---SIENLPMNLRNLVRLRHLEFHVY-YNTRKMPSHMGELIHLQILSWFVAGFEEGCKIEELGNLKNLKG-QLQLSNLEQVRS-----KEEALAAKLVNKKNLRELTFEWSIDILR--ECS--S 
Cucsa.094660  QTLRLGS--KIMHLPTKLRKLVNLRHLEFSLS-TQTKQMPQHLSRLLQLQTLSSFVVGFDKGCKIEELGPLNNLKG-ELSLFHLEHVKS-----KTEAMAANLAMKENISDLYFQWSLLSERE-DCS--N 
Cucsa.094670  QTLKLSR--FLNGLPKNLRKLVSLRHLEFFSDPCNPKQMPQHLGKLIQLQTLSSFVVGFDDGCKIEELRSLRNLKG-KLSLLCLERVKS-----KKEAMAANLVEKRNISYLSFYWALRCERS-EGS--N 
Cucsa.102240  EVLILNLQSRTYNLPGFIKRMNKLKVLIITYF---------------------GSFLTEVTSEDNQLLDSLTSLERIRFERISVPIFSN----------------------------------------- 
Cucsa.123410  KVLILNFSSSGYFLPSFLCNMPKIRALIVLNN---------------------NATHATLTN--FSVFSSLVNLRGIWLEKISMTQLFD----------------------------------------- 
Cucsa.128030  TLNLTECY-ELKELPRDIDNLVNLRHLTFEPCME-VTPTSEGMEKLTCLQTISLFVFDCKKTNKLWELNDLSYLTG-ELKIIGLEKLRS----SPSEITLINLKDKKGWQGLNLEWKLGK-DEYEG---- 
Cucsa.128100  TLILRYCL-WLRKLPKDINNLINLRHLDIYDCSS-LTHMPKGLGGMTSLQTMSMFVLGKNKGGDLSALNGLKSLRG-LLCIKGLQFCTT-----ADLKN------------LELHWDIKMDHEDALDDGD 
Cucsa.128110  TLILRHCS-DLRELPTDINNLINLKHLDVHGCYR-LTHMPKGLGGLTSLQTMNLFVLGKDKGCDLSELNELARLRG-SLLIKGLELCTT-----TDLKNAKYMEEKFGIQKLKLRWNRDLYDAETDYASE 
Cucsa.128130  TLDLTKCY-DLKELPRDINNFVNLRHLLCDSRLM-NMLQGT-MEKLTSLQTLSSFLFDCKRFDKVKEFSERSYFIEFDLKIKGLEQLRF----SPSDVKSVNLKNKK-VPLLRLKWKFENGNEYEG---- 
Cucsa.128140  -----------------------------------------------------------------------------------------------------HLQLR------ALYFKNLKN--------- 
Cucsa.132370  LLMGIDLP-PK-------------------------FEMPPYLSELVQLQTLFAFAVGFETGRKISELRGLRNLKG-LLKLHRLEHVES-----KEEAKAAKLVEKEKVEGLNLSWR------------- 
Cucsa.133510  EVLILNPGSETYKLPDFAKKMNRLKALIVRNY---------------------RSFPTELTS-DYQLINCLSRLERISLERISISSFID----------------------------------------- 
Cucsa.163670  TLIFRECF-KLRELPSDITNLINLRHLGINSLIEGLSYMPKGMGSMTTLQTMNLFILGENEGGELSELNGLINLRG-SLSIQQLQFCKP-----IGIENAKHLEEKSGIQKLKLYWYLLERKYEID---- 
Cucsa.178360  EILSLRASD-IIALPRVIGELTNLKMLNLS-DCSKLKVIPANLLSRLIGLSELYMDNSFKHWNVGQMEGYVNARISELDNLPRLTTLHVHIPNPTILPHAFVFRKLSGYRILIGDRWDWSG--------- 
Cucsa.178620  ALILDGCS-NLKELPKYTKRLINLKRLVLYGCSA-LTHMPKGLSEMTNLQTLTTFVLGKNIGGELKELEGLTKLRG-GLSIKHLESCTSIVDQQMKSKNSKFLQLKSGLQNLELQWKKLKIGDDQLE--D 
Cucsa.189390  ENLSLKGSH-IIQIPATISQLTQLKVLDLS-ECYALKVIPPNILVNLTKLEELYLLNFDGWESEELNQGRRNASISELSYLSQLCALALHIPSEKVMPKELFS--------------------------- 
Cucsa.237070  QILRIDKCP-ITLLPKSMSQLTQLKVLQVS-NCP-LKVIPPNTLSSLLKLQALDIWTSFNGWGEEVSHNNKLINNARLSELKCLPHLTN----------------------------------------- 
Cucsa.239860  EILRISNCRGITELPTSMSELKQLKVLVVS-HCFKLVVIHTNIISSMTKLEELDIQDCFKEWGEEVRYKNTWIPNAQLSELNCLSHLS------------------------------------------ 
Cucsa.248810  QTLKFVYS-VIEEFPMNFTNLVSLRHLELGEN---ADKTPPHLSQLTQLQTLSHFVIGFEEGFKITELGPLKNLKR-CLCVLCLEKVES-----KEEAKGADLAGKENLMALHLGWS---MNRKDN---- 
Cucsa.251930  QTLRLGC--FRGEAPKKFIKLISLRHFYMNVKRPTTRHMPSYLGRLVDLQSLPFFVVGTKKGFHIEELGYLRNLRG-KLKLYNLELVRN-----KEEAMRADLVKKDKVYKLKLVWS---EKR-ENN--N 
Cucsa.277260  QTLKLGY--IECDLPKNLRNLVNLRHLEFKKF-FDMGQMPSHMGNMIHLQTLSEFVVGLEKGCKIDELGPLKDLKG-TLTLKNLQNVQN-----KDEAMAAKLVEKKYLRHLIFQWFLNLYDRGEYD--E 
Cucsa.318890  QTLKFLYS-YVEKFPMNFTNLT-----------------------------LSHFVIGFEEGCKITELGPLKNLQG-CLSLLCLEKVES-----KEEANGTNLAEKEKLKDLHLSWS---NERKDNN--N 
Cucsa.326910  KVLHVLKCDDFNPS-----------------------EFPPNIIESMTQLEELKFDG---------------FKMNELSELNRLTRLFS----------------------------------------- 
Cucsa.328080  TLIIRGCL-ELRELSSDIKNLINLRHLDIKDFKHVWSYMPKGMGSMTTLQTMNLFILGENKGGELSELNGLVNLRG-SLSIQQLQFCKP-----IGLENVKYLEEKSRIQKLELHWKTYQRESKID---- 
Cucsa.337180  QTLILASCSALKELPNDIRQLTNLRYLWVTAN----------------NLRLHKNGVGTMTSLRFLAIGGCKKLTLTLKG--VEFRLQR----------------------------------------- 
Cucsa.337190  QTLILASCSALEELPKDICKLSNLRYLWVTSN----------------KLRLHKNGVGTMTSLRFLAIGGCDKLQDLFERPSCLVRLET----------------------------------------- 
Cucsa.338110  QTLKLG----QSGLPKNLRKLVNLRHLEFKMF-GDT-AMPSDMGNLIHLQSLSGFLVGFEKGCKIEELGPLKNLKG-KLTLTNLWRVQN-----KDEAMAAKLVEKKNLRHLN-LWFFETDKRGEDD--E 
Cucsa.338190  QTLVLAFCSELEELPRNIKNLISLRFLWIQTK----------------QARLEKDEIGSLTSLRFLAIGRSENLTHLFEDINKLNSLKT---------------------------------------LI 

                          920        930        940         950        960         970        980        990        1000       1010       1020        1030      1040           
                ....|....|....|....|....|....|....|....|....|....|....|....|....|....|....|....|....|....|....|....|....|....|....|....|....|....|
Cucsa.017460  ---KERKRKVE----------------------------------------------------------------------------------------------------------------------- 
Cucsa.017490  NEYARTLMLNIESQVGSIDEGLEILLQRSERLIVSDSKGNFINAMFKPNGNGYP----------------------------------------------------------CLKYLWMIDENGNSEMAH 
Cucsa.088220  KMNYSRILEVKMESEMCLDDWIKFLLKRSEEVHLEGSICSKVLN--SELLDANG-----------------------------------------------------------FLHLKNLWIFYNSDIQH 
Cucsa.091880  --NKTNFFRIKMESERCLDDWIKTLLKRSEEVHLKGSICSKVLHDANEFLHLKESLFSKLKSVVVTKCNKLEKLFFNCILDDILSLEEIAIHYCEKMEVMIVMENEEATNHIEFTHLKYLFLTYVPQLQK 
Cucsa.094560  YNDFEVLEGLQPHKNLQFLSIINFAGQLLPPAIFVE----NLAVIHLRHCVRCEILPM----------------------------------------------------LGQLPNLEELNISY-LLCLR 
Cucsa.094580  YNDLEVLKGLQPHKNLQFLSIINYAGQILPPAIFVE----NLVVIHLRHCVRCETLPM----------------------------------------------------LGELPNLEELNISN-LHCLR 
Cucsa.094650  YNDFEVLEGLQPPKNLSSLKITNFGGKFLPAATFVE----NLVFLCLYGCTKCERLPM----------------------------------------------------LGQLANLQELSICF-MDSVR 
Cucsa.094660  -NDLNVLEGLRPHKNLQALKIENFGG-VLPNGLFVE----NLVEVILYDCKRCETLPM----------------------------------------------------LGHLSKLELLHIRC-LDSVK 
Cucsa.094670  YNDLNVLEGLQPHKNLQALRIQNFLGKLLPNVIFVE----NLVEIYLHECEMCETLPT----------------------------------------------------LGQLSKLEVLELRC-LYSVR 
Cucsa.102240  -PNPKPLINLQKISFFMCKFGQTFMDPSTPISDLLP----NLLEISIDFCNNLSEVPNR---------------------------------------------------LCEIVSLQKLSITN------ 
Cucsa.123410  -ACT-PLKHLRKLSLVFCKINNSLDEWAVDVSQIFP----FLFELKIDHCNDLRKLPSS---------------------------------------------------ICEMQSLKCLSVTN------ 
Cucsa.128030  EADETIMEGLEPHPNVESLSINGYTGGALPNWVFN--SLMKLTEIEIENCPRVQHLPQ----------------------------------------------------FNQLQDLRALHLVGLRSLEF 
Cucsa.128100  NDDEGVLEGLKPHSNIRKMIIKGYRGMKLCDWFSSN-FLGGLVSIELSHCEKLEHLPQ----------------------------------------------------FDQFLYLKHLLLGYLPNIEY 
Cucsa.128110  NDDERVLDCLKPHSNVHKMQIRGYRGVKLCNWLSFD-YLGGLVNIELQSCEKLQHLPQ----------------------------------------------------FDQFPFLKHLLLENLPSIEY 
Cucsa.128130  DADDIVLEGLEPHPYVNLLQIEGYCGVGLPNWVST--SIL-LRGIRIGNCDR-LHLNQ----------------------------------------------------LSHLHALEILNLEGLKS--- 
Cucsa.128140  ---------------------------------------AMIVLEFTGKLKHLRYLSI----------------------------------------------------MDSF---------------- 
Cucsa.132370  ---GKWKNRLEPHKNLKDLKIQSFLGGCFPKETFVE----NLVTITLHKCGNCEKLPM----------------------------------------------------LGQLSKLEALIIISNFPKVK 
Cucsa.133510  -QNLKPLWHLKKLSFFMCKIDKAFTDCSTQISYMLP----NLLEISIDFCNDLVAFPVG---------------------------------------------------LCEVVTLEKLSITN------ 
Cucsa.163670  DEDEKVLECLKPHPNLQKIVINGYGGVKLCNWFSFD-YIVNLVIIDLFNCNKLQQLPR----------------------------------------------------FDQFPFLKHLKLQYLPNVEF 
Cucsa.178360  NYETSRTLKLKLDSSIQREDAIQALLENIEDLYLDELESVKNILFSLDYKG--------------------------------------------------------------FPKLKGLRVKNNGEIVT 
Cucsa.178620  VMYESVLDCLQPHSNLKEIRIDGYGGVNLCNWVSSNKSLGCLVTTYLYRCKRLRHLFR----------------------------------------------------LDQFPNLKYLTLQNLPNIEY 
Cucsa.189390  -----RVFPFELNENE-------------------------------------------------------------------------------------------------SSYLKYLYINYNSNFQH 
Cucsa.237070  --------LKIHILDIKILSDLIFLKNLKLERFVIHVGELKMSQRLQG-CEQYA----------------------------------------------------------TTLMLKIITS--SSQIVS 
Cucsa.239860  -------ILRIVSVNG---TKLSILLEGTKRLMILNDSKGFANDIFKAIGNGYP----------------------------------------------------------LLKCLEIHD---NSETPH 
Cucsa.248810  --DLEVLEGLQPNINLQSLRITNFAGRHLPNNIFVE----NLREIHLSHCNSCEKLPM----------------------------------------------------LGQLNNLKELQICS-FEGLQ 
Cucsa.251930  NHDISVLEGLQPHINLQYLTVEAFMGELFPNLTFVE----NLVQISLKNCSRCRRIPT----------------------------------------------------FGHLPNLKVLEISG-LHNLK 
Cucsa.277260  DDNKQVLEGLQPHKNVQSLDIRGFQGRVLNNNIFVE----NLVEIRLVDCGRCEVLPM----------------------------------------------------LGQLPNLKKLEIIS-MNSVR 
Cucsa.318890  YNDLEVLEGLQPNQNLQSLGIYNFAERRLPNKIFVE----NLSVIGLYGCNNCEKLPM----------------------------------------------------LGQLNNLKKLEIYS-FHGVQ 
Cucsa.326910  -----------------------LELRIQNVEILLNELSVEKAEKLEEFSFCVD----------------------------------------------------------SVGFTNLLCN----NNHT 
Cucsa.328080  DEDERVLESLKPHSNLQKIRIEGYRGLKLCNWFSFD-SIVNLVFIKLFNCEKLQQLPR----------------------------------------------------FDRFPFLKHLHLEDLPSIEY 
Cucsa.337180  --------------------------------------------FTIRELPIVKKLP------------------------------------------------------------------------- 
Cucsa.337190  --------------------------------------------LMIYDCNSLQLLP------------------------------------------------------------------------- 
Cucsa.338110  DGIVQVLEGLQPHKNLQSLEILGFRGKVLPTGIFVE----NLVKIRLGHFERCEVLPM----------------------------------------------------LGQLPNLKELEIMY-MESVR 
Cucsa.338190  IYECKSLLTLPKGLENMKSICNMGIWECDRLRFTFSLASLHLKKLILRELTAVSTLPN--------------------------------------------------WLSNLDGTLEVLEIG------- 

                         1050       1060       1070        1080       1090       1100       1110       1120       1130       1140        1150       1160      1170           
                ....|....|....|....|....|....|....|....|....|....|....|....|....|....|....|....|....|....|....|....|....|....|....|....|....|....|
Cucsa.017460  ---------------------------------------------------------------------------------------------------------------------------------- 
Cucsa.017490  LIGS--------------------------------------DFTSLKYLIIFGMKRLEN------------------------------------------------IVPRHISLSP------------ 
Cucsa.088220  FIHEKNKP-------------------------------LRKCLSKLEFLYLKNLENLES-----------------------------------------------VIHGYNHGESP------------ 
Cucsa.091880  FCSKIEKFGQLSQDNSISNTVDIGIFEVQESSITDTSLIVLKNLRELKLYNLPNLEYVWSKNPCELLSFV-------------------------------------NIKGLAIDECPRLRREYSVKILK 
Cucsa.094560  SIG------------------------------------HKVLFPKLKKFVLSQMPNLEQWEEVVF---------------------------ISKKDAIFPLLEDLNISFCPILTSIPNIFRRP----- 
Cucsa.094580  CIGNEFYGSYD------------------------HPNNHKVLFRKLKKFVLSEMHNLEQWEELVF---------------------------TSRKDAIFPLLEDLNIRDCPILTNCP----------- 
Cucsa.094650  SIGSEFYGIDS-------------------------NRRG--YFPKLKKFDFCWMCNLEQWELEVA---------------------------NHESNHFG-SLQTLKLDRCGKLTKLP----------- 
Cucsa.094660  SIGDEFYGNNNSY----------------------HNEWSSLLFPKLKTLHISQMKSLELWQEIGS---------------------------SSNYGATFPHLESLSIVWCSKLMNIPNLFQVP----- 
Cucsa.094670  SIGEEFYGN---------------------------YLEKMILFPTLKAFHICEMINLENWEEIMV---------------------------VSN-GTIFSNLESFNIVCCPRLTSIPNLFASQHESSF 
Cucsa.102240  ---------------------------------------------------------------------------------------------------------------------------------- 
Cucsa.123410  ---------------------------------------------------------------------------------------------------------------------------------- 
Cucsa.128030  IDKSDPYSSSV-------------------------------FFPSLKFLRLEDMPNLEGWWELGESK----------------------------VVARETSGKAKWLPPTFPQLSSMPKLASIG---- 
Cucsa.128100  IDSGNSVSSST------------------------------TFFPSLEKLRIESMPKLKGWWKGEIS--------FPTTILHQLSELCIFYCPLLASIPQHPSLESLRICGVSVQLFQMVIRMATD---- 
Cucsa.128110  IDNNNSLSSS-------------------------------TFFPSLEKLTIMTMPNLKGWWKGETPPESARYSALFPTILHHLSRLDISNCPQLASIPQHPPLRSLALNDVSVQLFDMVIKMATT---- 
Cucsa.128130  ---------------------------------------------------------------------------------------------------------------------------------- 
Cucsa.128140  ------------------------------------------------------------------------------------------------------------ILNLPDSITEL----------- 
Cucsa.132370  SIGNEFYGNYND-----------------------GQSKSSVVFPKLKEFYVIAMYSLVEWEEVVN------------------------------NVKAFPRLECLHIVKCTKLTSALKIVFSSLINFD 
Cucsa.133510  ---------------------------------------------------------------------------------------------------------------------------------- 
Cucsa.163670  IDNNDSVSSSLT-----------------------------TFFPSLEKLRIFRLPKLKEWWKRKLID---------------------------QTIPQHRRLESLNISGVSLQVFELVMEMATTN--- 
Cucsa.178360  VVNSDNMHHP------------------------------HSAFPLLESLFLKNLAELGS-----------------------------------------------ICRGKLPQMS------------- 
Cucsa.178620  MIVDNDDSVSSS-----------------------------TIFPYLKKFTISKMPKLVSWCKDSTST----------------------------KSPTYWHAPKLKLLQISDSEDELNVVPLKI---- 
Cucsa.189390  FIHGQNKTN------------------------------LQKVLSNMERLELSYLENLES-----------------------------------------------FFHGDIKDIS------------- 
Cucsa.237070  IDHH--------------------------------------EFLSLE-----KMESLEN------------------------------------------------IVHADVFTSP------------ 
Cucsa.239860  LRGN--------------------------------------DFTSLKRLVLDRMVMLES------------------------------------------------IIPRHSPINP------------ 
Cucsa.248810  VIDNEFYGNDP---------------------------NQRRFFPKLEKFEISYMINLEQWKEVITND-------------------------ESSNVTIFPNLKCLKIWGCPKLLNIP----------- 
Cucsa.251930  CIGTEFYGN---------------------------EYGEGSLFPKLKRFHLSDMNNLGRWEEAA----------------------------VPTEVAVFPCLEELKILDCPRLEIAPDYFSTLRTLEI 
Cucsa.277260  SIGSEFYGVDC-------------------------NDRNSSAFPQLNKFHICGLKKLQQWDEAT----------------------------VFASNRFG-CLKELILSGCHQLAKLP----------- 
Cucsa.318890  IIDNEFYGNDL---------------------------NQRRFFPKLEIFVMCDMINLEQWKEVMTND-------------------------ASSNVTIFSNLKCLEIRGCP----------------- 
Cucsa.326910  VPYG--------------------------------------NYNWYPRLKELQIYIHNN------------------------------------------------QNQYLDMPRG------------ 
Cucsa.328080  IAINNYVSSSMT-----------------------------TFFPSLENLSIIKLPNLKEWWKGESID---------------------------QNTSFPTILR--HLSQLKIHYCRQLASIPQHG--- 
Cucsa.337180  ---------------------------------------------------------------------------------------------------------------------------------- 
Cucsa.337190  ---------------------------------------------------------------------------------------------------------------------------------- 
Cucsa.338110  SIGNEFYGVDS-------------------------SHQNSVAFPQLKKVSIYEMMNLEQWDEATV---------------------------VLASNLFG-CLKEVRIRRCNPLAKLP----------- 
Cucsa.338190  ------------------------------------------EFPTLRKLPIWLLN-------------------------------------------------------------------------- 


                         1180       1190       1200        1210       1220       1230       1240       1250       1260        1270       1280       1290      1300           
                ....|....|....|....|....|....|....|....|....|....|....|....|....|....|....|....|....|....|....|....|....|....|....|....|....|....|
Cucsa.017460  ---------------------------------------------------------------------------------------------------------------------------------- 
Cucsa.017490  ---------------------------------------------------------------------------------------------------------------------------------- 
Cucsa.088220  ---------------------------------------------------------------------------------------------------------------------------------- 
Cucsa.091880  QLERLTMDIKQLMEVIENQKSTDHNMVKSKQLETSSKDNSTHLPVEIVQILYQLEHFELEGAYIEEVFPSNILIPMKKQYYARSKNSVRSWFLSKLPKLRHLWSECSQKNAFPILQDLNVIRISECGGLS 
Cucsa.094560  ---------------------------------------------------------------LKKLHVYGCHEVTGLPKDLQLCTSIEDLKIVGCRKMT------------------------------ 
Cucsa.094580  ------------------------------------------------------------------------------KIDLQLCTSIEDLKIVGCLEMI------------------------------ 
Cucsa.094650  -------------------------------------------------------------------------------NGLECCKSVHEVIISNCPNLT------------------------------ 
Cucsa.094660  -------------------------------------------------------------PKLQSLKIFYCEKLTKLPHWLNLCSSIENMVICNCPNVNN----------------------------- 
Cucsa.094670  PSLQ-------------------------------------------------------HSAKLRSLKILGCESLQKQPNGLEFCSSLENMWISNCSNLN------------------------------ 
Cucsa.102240  ---------------------------------------------------------------------------------------------------------------------------------- 
Cucsa.123410  ---------------------------------------------------------------------------------------------------------------------------------- 
Cucsa.128030  ----------------------------------------------------------------------------------------ADVILHDIGVQM------------------------------ 
Cucsa.128100  ----------------------------------------------------------------------------------LSEHSSSSSTLSKLSFLE------------------------------ 
Cucsa.128110  ----------------------------------------------------------------------------------PAADSSS--ALSKLSILH------------------------------ 
Cucsa.128130  ---------------------------------------------------------------------------------------------------------------------------------- 
Cucsa.128140  ---------------------------------------------------------------------------------------------YNLETLI------------------------------ 
Cucsa.132370  HSIS--------------------------------------YLNLLPNSLKILLSCCLFQDVEDESEIGQKFLMHDLIHDIACHVSNDEKLPSDHSLLSMRKHWT------------------------ 
Cucsa.133510  ---------------------------------------------------------------------------------------------------------------------------------- 
Cucsa.163670  -------------------------------------------------------------------------------IIVGSQDSSSSTTSISLSFLS------------------------------ 
Cucsa.178360  ---------------------------------------------------------------------------------------------------------------------------------- 
Cucsa.178620  --------------------------------------------------------------------------------------------YENLTFLF------------------------------ 
Cucsa.189390  ---------------------------------------------------------------------------------------------------------------------------------- 
Cucsa.237070  ---------------------------------------------------------------------------------------------------------------------------------- 
Cucsa.239860  ---------------------------------------------------------------------------------------------------------------------------------- 
Cucsa.248810  -------------------------------------------------------------------------------KAFDEN--------------------------------------------- 
Cucsa.251930  DDVNNPISQITLQTFKLLGIIHSGNLSGLPEELRGNLSSLEEFKVWYYLHLKSFPTIQWLTDILKGKTGYDTKWTNIQSHGLESYTSVNELSIVGHSDLT------------------------------ 
Cucsa.277260  -------------------------------------------------------------------------------SGLEGCYSIEYLAIDGCPNLM------------------------------ 
Cucsa.318890  ---------------------------------------------------------------------------------------------------------------------------------- 
Cucsa.326910  ---------------------------------------------------------------------------------------------------------------------------------- 
Cucsa.328080  -------------------------------------------------------------------------------PLQ-SLDIRDISLQLFELVIK------------------------------ 
Cucsa.337180  ---------------------------------------------------------------------------------------------------------------------------------- 
Cucsa.337190  ---------------------------------------------------------------------------------------------------------------------------------- 
Cucsa.338110  -------------------------------------------------------------------------------SGLEGCHSLEYLSIRGCFNLM------------------------------ 
Cucsa.338190  ---------------------------------------------------------------------------------------------------------------------------------- 

                         1310       1320       1330        1340       1350       1360       1370       1380       1390        1400       1410       1420      1430           
                ....|....|....|....|....|....|....|....|....|....|....|....|....|....|....|....|....|....|....|....|....|....|....|....|....|....|
Cucsa.017460  -------------------NKNDLEVLEGLQPPKNVEYLRIKYFLGGCLPNQTFVENLVKIELRDCG-----------------------------NCEKLPRLGQLGN-LEILDISWFERVKSIGNEFY 
Cucsa.017490  --------FKKVKTIAIQFCGQIRNLFSF-SIFKDLLDLQEIEVINCGKMEGIIFMEIGDQLNICSCPLTSLQLENVDKLTSFC-TKDLIQESSQSIIPFFDG-QVSFPELNDLSIVGGNNLETLWHKNN 
Cucsa.088220  --------LNNLKNVIVWNCNKLKTLFLN-CMLDDVLNLEEIEINYCKKMEVMIT----------------------------------------VKENEETTNHVEFTHLKSLCLWTLPQLHKFCSKVS 
Cucsa.091880  SLVSSSVSFTNLTVLKVDKCDRLTYLLNP-LVATTLVQLEELTLRECKMMSSVIEGG---SA--------------------------------EEDGNEETTNQIEFTHLKSLFLKDLPRLQKFYSKIE 
Cucsa.094560  ------LNVQNMDSLSRFSMNGLQKFPQGLANLKNLKEMTIIECSQDCDFSPLMQLSSLVKLHLVI--------------------------FPGSVTEQLPQQLEHLIALRSLYINDFDGIEVLP-EWL 
Cucsa.094580  ------LNVQNMHTLSRFSMNGLQKFPQGLSHLKNLKEMIITECSQDCDFTPLMQLSSLVNLDLVL--------------------------FAGNGAVQLPQQLQHLTALRSLIINDFDGIEVLP-EWL 
Cucsa.094650  ------LNVEEMHNLSVLLIDGLKFLPKGLALHPNLKTIMIKGCIEDYDYSPFLNLPS-LTKLYLN-------------------------DGLGN-ATQLPKQLQHLTALKILAIENFYGIEVLP-EWL 
Cucsa.094660  ---NSLPNLKSMPNLSSLSIQAFEKLPEGLATIHNLKRLDVYGELQGLDWSPFMYLNS--------------------------------------------------------SIEILRDIDSLP-EWL 
Cucsa.094670  ----YPPSLQNMQNLTSLSITEFRKLPDGLAQVCKLKSLSVHGYLQGYDWSPLVHLGSLENLVLVD--------------------------LDGSGAIQLPQQLEQLTSLRSLHISHFSGIEALP-EWF 
Cucsa.102240  -------------------CHGLSSLPEDVGKLINLKNLRLRS---------------------------------------------------CIHLEEFPESTTKLRELVLLDISNCIGLAKLPEKIG 
Cucsa.123410  -------------------CHNLSQLPTNLWKLKNLQILRLFA---------------------------------------------------CPLLKTLSPSICVLSCLKYIDISQCVYLTSLPEEIG 
Cucsa.128030  ------VSTIGPVSSFMFLSMHGMTNLKYLWEEFQQDLVSSS--TSTMSSP----------ISLRYLT-----------------------ISGCPYLMSLPEWIGVLTSLETLHIKECPKLKSLPEGMQ 
Cucsa.128100  ------IGTIDLEFLP-VELFCNMTHLESLIIERCKSLQMSSPHP-VDEDNDVVWK---KLSNLRTLR-----------------------LESILKLEYFPKSLKYITSLETLKLSNCENLVST-EGIG 
Cucsa.128110  ------IQNIDLEFLP-EELFGSTTDLEIFTVELKYMTTLERLDL-YNCPNIVSLEGISHLTSLSSLR-----------------------ICNCSNLTSLPEGISHLTSLSYLTIY-CVNLTSLPEGVS 
Cucsa.128130  -------------------------------------------------------------------------------------------------VMSISEWIGTLTSLVSLEIEECPKLKSLPKEMQ 
Cucsa.128140  ------LRNSS--------------------------FKMLP--------DNIGN-----LINLKHLD-----------------------LSNNRNLKFLPDSISDLCKLEELILHGCLRLEEFPEDTK 
Cucsa.132370  ---NDDKIVASKLRTNIVKNENDFEVLEGLEQHNNLKYLEIESFSGGQFPNQIFVENLVKITLIECG-----------------------------NCEKLPMLGQLTKYLEILVIFRLRKVESIGNEFY 
Cucsa.133510  -------------------CHALSSLPEEIGQLINLKILRLRS---------------------------------------------------CIHLEKLPESISRLRELVYLDISHCVGLTKLPDKIG 
Cucsa.163670  ------IEDIDFEFLQFHDLFSNMTHLKSLWIINCKNIKMS------SSLDAVTWK---GLGSLRELM-----------------------LSSIPDLEYLPKSLQCVTTLQSLQIYNCPNLVSI-ESIR 
Cucsa.178360  --------FRNLKRVKVESCDRLKFVFPS-SMVRGLIHLQSLEISECGIIETIVSKNKETEM--------------------------------QINGDKWDENMIEFPELRSLILQHLPALMGFYCHDC 
Cucsa.178620  ------LHNLSRVEYLPECWQHYMTSLQLLCLSKCNNLKSLP--------GWIRN-----LTSLTNLN-----------------------ISYCEKLAFLPEGIQHVHNLQSIAVVDCPILKEWCKKNR 
Cucsa.189390  --------FNNLKVIKLLSCNKLGSLFLDSNMNGMLLHLERINITDCEKVKTVIL-----------------------------------------MESGNPSDPVEFTNLKRLRLNGLPQLQSFYSKIE 
Cucsa.237070  --------FRKLRSIKVISCKRLRYLFSF-SIFKGLVDLQRVFIFDCNMMDEILCMDSED---------STIAVE---------------------------GNSIECPQLKDLTIIGAHNLKMLWHKNG 
Cucsa.239860  --------FNKLKFIKIGRCEQLRNFFPL-SVFKGLSNLRQIEIYECNMMEEIVSIEIEDHITIYTSPLTSLRIERVNKLTSFCSTKSSIQ---QTIVPLFDERRVSFPELKYLSIGRANNLEMLWHKNG 
Cucsa.248810  -------NMQHLESLILSCCNKLTKLPDGLQFCSSIEG--LTIDKCSNLSINMRNKPKLWYLIIGC-------------------------------VTQIPEQLQHLTALQFLSIQHFRCIEALP-EWL 
Cucsa.251930  ----STPDIKALYNLSSLTISGLKKLPKGFHCLTCLKSLSIGGFMEGFDFRPLLHLKSLENLAMID--------------------------FILAES-TLPDELQHLTGLKHLKIVGFQGIESLP-EWL 
Cucsa.277260  ------LNVQNLYNLYHLDIRGLKRLPDEFGKLTNLKKLRIGGCMQNYEFSPFIHLSSQLVELELTDD-----------------------GSSGSETTQLPQQLQHLTNLKVLKIADFDDIEVLP-EWL 
Cucsa.318890  ---------------------KLTKLPNGLHFCSSIRRHLLSLKKITLVEDELSNNS----------------------------------------VTQISEQLQHLTALEFLSIENFGGIEALP-EWL 
Cucsa.326910  --------IENNPCILIFSCNKLTYVFPS-HMLTLLVFLNTLEVHHCKLVERIFEIEEWSGS---------------------------------------GGAGDVNQVLVPFTILHLSFLPNLKHVWN 
Cucsa.328080  ------MTATNIIFLP-NDLFSNVTHLQSLVIGRCFNLKMS------FDDDNVRWK---ELGSLRTLR-----------------------LCFIPKLEYLPKGFQYLKALEHLELLWCENLACI-LGIE 
Cucsa.337180  -----------------------------------------------------------------------------------------------------EWTQRFTETLRVLEIIDCPIEWNDDVLKS 
Cucsa.337190  -----------------------------------------------------------------------------------------------------NEMGSLIS-LQNLVIWSCKQLTLKGLEKV 
Cucsa.338110  ------LNVQNLHKLYHLEIDGLKRLPKGMDGLTRLKELKIGGCMQNYEFSSVIHLASQLVELELS-------------------------GRYGSVDTQLPQQLQHLTNLQVLKITQFDCIEALP-EWI 
Cucsa.338190  --------FWELRILGISNCPKLKHDSFPPELNYFCDKIEELRITFCGSLSKSLLKKS---------------------------------------MKEIEPESRVIFYIHTIYVDSKRMTPPVESTDE 

                          1440        1450       1460       1470       1480       1490       1500       1510        1520       1530       1540      1550      1560           
                 ....|....|....|....|....|....|....|....|....|....|....|....|....|....|....|....|....|....|....|....|....|....|....|....|....|....|
Cucsa.017460  GNSSNNQRS-----------------------------------------------LFPRLKELYVDEMRRIGEWEEVGS--NVKAFPRLERLYIGCCRDLVKIPDVFGYCDEYGEKHLEVVEIIEHL-- 
Cucsa.017490  NPTTG------------------------------------------------------SFCKLQSIRIEQCTQLRCMFPSNMLTSLASLHTIQIISCASLKRIFEIENQSFNDTTVLWSLNELHLLNLP 
Cucsa.088220  --------------------------------LPNLEKLKIWCTKDLKKIWSNNVLIPNSFSKLKEIDIYSCNNLQKALFSPNMMSILTCLKVLRIEDCKLLEGIFEVQEPISVVEASP---IALQTLSE 
Cucsa.091880  TFGQLSRDNSENPETTTIHNRIGDSFFSEQESLPNLETLRIDGAENLRMIWSNNVLIPNSFSKLEEVEIYSCNNLQDVLFHPNIINMLTCLNTLRIKNCELLEGIFEVQEPISVTKTKTNAIVLPNNLIE 
Cucsa.094560  GN----------------------------------------------------------LTSLEVLGLYYCINLKQFPSKKAMQCLTQLVHVDVHNCPSSQILSHDLKAKAHAKANLVQW--------- 
Cucsa.094580  GN----------------------------------------------------------LASLEVLGLYYCRSLKQFPSKKAIAMSHPISPCG------CLWLSTTTQVRRFCAMMF------------ 
Cucsa.094650  RK----------------------------------------------------------LTCLETLDLVH--LVKSLPS---LQGEDNRVAEIAYETGEMETYECDNKRSWFRLHLNFYGCTLVELV-- 
Cucsa.094660  GN----------------------------------------------------------LTSLETLNLRYCKNLKSFPSIEA----------------------------------------------- 
Cucsa.094670  GN----------------------------------------------------------FTCLETLKLYNCVNLKDMASKEAMSKLTRLTSLRVYGCPQLNTFMFNGMSPNSSTSSSRTLSTLE----- 
Cucsa.102240  EFH-----------------------------------------------------------NLEKLDMRHCWSLSKLPLSIGKLKNVK-FLCDREVG-EWLRKVAPRLAKQVKVQEEEANLEWLGF--- 
Cucsa.123410  KLT-----------------------------------------------------------SLEKIDMRECSLIRRLPRSVVSLQSLCHVICEEDVSWLWEDLKSHMPNLYIQVAEKCFNLDWLKE--- 
Cucsa.128030  QLK-----------------------------------------------------------SLKELHIEDCPELEDRCKQGGEDWPN----ISHVPNFTYKNASDIDTPQSSSGFSHHPFSIVRISVI- 
Cucsa.128100  ELI-----------------------------------------------------------SLSHLEIDRCPNLPILSEDVGDLISLSHLLIWNCPKLTSLS-EGITRLTSLSSLCLEDCPNLVSLPQE 
Cucsa.128110  HLT-----------------------------------------------------------SLSSFTIEECPCLTSLPEGVSHLTSLSTLIIRRCVNLTSLP-EGIGHLTSLSIFTIEECLNLTSLPEG 
Cucsa.128130  QLK-----------------------------------------------------------SLVQLNIIKCPQLGERCKEGGEDWPN----ISHIPDVLID---------------------------- 
Cucsa.128140  -----------------------------------------------------------------KLINLKHLNEK-HADAL--WRDKEKGFNEGLCSLIKA-VEKDFHSVHKCKLFF---CSVVFLM-- 
Cucsa.132370  GNQRRSSSS-----------------------------------------------VFPKLKEFYVDEMDSLVEWEEAVSNYNVKAFPRLECLHIISCKKLLKIPDTK--VQICGLLLLQLQEEAPWLIC 
Cucsa.133510  NLQ-----------------------------------------------------------KLEKLNMWSCPNMRKLPKSVGNLKNLKEVVCESEMK-IWVNFVAPRLGNVVKEHKEEINLDFLN---- 
Cucsa.163670  HLTT----------------------------------------------------------SLSVLEIHGCPNITFYPHEMSQLASLAITFQNRGWSDNYDPGEGRKEDDDQKQFGRDEQHEGTTH--- 
Cucsa.178360  IT-----------------------------------------------------------VPSTKVDSRQTVFTIEPSFHPLLSQQVSFPKLETLKLHALNSGKIWQDQLPSSFYGFKNLTSLSVEGCA 
Cucsa.178620  REDW----------------------------------------------------------PKIKYYISEHIWEN-ICSLTGSWSSRNKIFSDHFRSVIALNCSACFYLIYICRCYYELISSPFFIILY 
Cucsa.189390  Q------------------------------------------------------LSPD---QEAEKDERSRNFNDGLLFNEQ----------------------------------------------- 
Cucsa.237070  LAPN-------------------------------------------------------FFSKLQRISINSCNTLRYRG--------------------------------------------------- 
Cucsa.239860  S----------------------------------------------------------SFSKLQTIEISDCKELRCVFPSNIATSLVFLDTLKIYGCELLEMIFEIEKQKTSGDTKVVPLRYLSLGFLK 
Cucsa.248810  GN----------------------------------------------------------YVCLQTLNLWNCKKLKKLPSTEAMLRLTKLNKLHDLN---ICLS------PSFSSLLCFLIN-------- 
Cucsa.251930  GN----------------------------------------------------------LNSLESLHIESCRKLRELP--EAMGCLAKLEEVRSFNCPELRVYQDESEWAKISYIPRFISFNYWVDEDQ 
Cucsa.277260  GN----------------------------------------------------------LTCLATLVFLECKNLKELPSREAIQRLTKLDDLVIDGCPKLLLGEGDQERAKLSHLPSKCGLGLKSVMIN 
Cucsa.318890  GN----------------------------------------------------------FVCLQTLSLYNCKNLKKLPSTKAMLRLTKLNQLYACKCPMLLLEEGDPERAKLSHFPNMLVQRNGYLKCI 
Cucsa.326910  TDPNP------------------------------------------------------TTSDLLPQCKESGDYKVSDA--------------------------------------------------- 
Cucsa.328080  HLT-----------------------------------------------------------SLSRLEISNCPNLTSLPEGMTQLISLTCLIIDDCPNLSTLP-EGLHHLLNTPRYAPLIFSH------- 
Cucsa.337180  YKS------------------------------------------------------------LERFSIHGAVRTKNQIGGYNIDYRNFVR-------SRKVKKEVKTCVYY------------------ 
Cucsa.337190  DFS------------------------------------------------------------LQRFTIR-ELPEVNKLPEWLQRSTETLRVLEIIDCPIKVEEEGIKIHHWEFGTA------------- 
Cucsa.338110  GN----------------------------------------------------------LISLKTLKCSYCFKLKELPSREAILRLTKLENLDIFECPKLLVGEGDQERAKLSHLPSKCVHKSE----- 
Cucsa.338190  PKE-------------------------------------------------------------AETKQDDAYNNASPPGTEQPSKTKHDDANNNMSHPGIGLLSESKQEHTNNNINEIETVKVCLGDND 

                         1570        1580       1590      1600        1610       1620       1630       1640       1650       1660        1670       1680      1690           
                ....|....|....|....|....|....|....|....|....|....|....|....|....|....|....|....|....|....|....|....|....|....|....|....|....|....|
Cucsa.017460  ---------------------------------------------------------------------------------------------------------------------------------- 
Cucsa.017490  NLKHVWRKDIIKILTFPSLKRVKIHGCTKLTHVWKD-NNKVTRS-----------------------------------FDSLERIE------------------------------------------- 
Cucsa.088220  LKLYKLPNLEYVWSKDS--CELQSLVNIKRLTMDECPRLRREYSVKILKQLEALSIDIKQLMEVIGKKKSTDYNRILINKLVIGQVEVLQLGDGSELFPKLKTLKLYGFVEDNSTHLPMEIVQNLYQFEK 
Cucsa.091880  LELYNLPNLEYLWSKNPNFERLVTFESIRSLSIEKCSKLKGEY--------------------------------FLSIKTFKQLVEVLQLRDGSKLFSNLKELKLYGFVEYNSTHLPMEIVQVLNQLEK 
Cucsa.094560  ---------------------------------------------------------------------------------------------------------------------------------- 
Cucsa.094580  ---------------------------------------------------------------------------------------------------------------------------------- 
Cucsa.094650  ---------------------------------------------------------------------------------------------------------------------------------- 
Cucsa.094660  ---------------------------------------------------------------------------------------------------------------------------------- 
Cucsa.094670  ---------------------------------------------------------------------------------------------------------------------------------- 
Cucsa.102240  ---------------------------------------------------------------------------------------------------------------------------------- 
Cucsa.123410  ---------------------------------------------------------------------------------------------------------------------------------- 
Cucsa.128030  ---------------------------------------------------------------------------------------------------------------------------------- 
Cucsa.128100  FLHHHSSLPGGRFLRILNCPKLQIQDKKQKEEEEEDQEDWNELIHVLTGCR------------------------------------------------------------------------------- 
Cucsa.128110  LLH-LSSLRGS--LTVSKCPKLSKTWKKLNK--------------------------------------------------------------------------------------------------- 
Cucsa.128130  ---------------------------------------------------------------------------------------------------------------------------------- 
Cucsa.128140  ---------------------------------------------------------------------------------------------------------------------------------- 
Cucsa.132370  YSNVRSFFLKEDTGPS------------------------------------------------------------------------------------------------------------------ 
Cucsa.133510  ---------------------------------------------------------------------------------------------------------------------------------- 
Cucsa.163670  ---------------------------------------------------------------------------------------------------------------------------------- 
Cucsa.178360  SIKYLMTITVARSLVNLERLELNDCKLMKAIIISEDQDLDNNYP-------------------------------------------SKSILQNKDVFANLESLLISRMDALETLWVNEAASGSFTKLKK 
Cucsa.178620  HVLFNE---------------------------------------------------------------------------------------------------------------------------- 
Cucsa.189390  ---------------------------------------------------------------------------------------------------------------------------------- 
Cucsa.237070  ---------------------------------------------------------------------------------------------------------------------------------- 
Cucsa.239860  NLKYVWDKDVDDVVAFPNLKKVKVGRCPKLKIIFPASFTKYMKE-----------------------------------IEELEMVEPFNYEIFPVDEASKLKEVALFQSLETLRMSCKQAVKERFWVMS 
Cucsa.248810  ---------------------------------------------------------------------------------------------------------------------------------- 
Cucsa.251930  QRIQFKVCHKPS---------------------------------------------------------------------------------------------------------------------- 
Cucsa.277260  LLPKKSF--------------------------------------------------------------------------------------------------------------------------- 
Cucsa.318890  ---------------------------------------------------------------------------------------------------------------------------------- 
Cucsa.326910  ---------------------------------------------------------------------------------------------------------------------------------- 
Cucsa.328080  ---------------------------------------------------------------------------------------------------------------------------------- 
Cucsa.337180  ---------------------------------------------------------------------------------------------------------------------------------- 
Cucsa.337190  ---------------------------------------------------------------------------------------------------------------------------------- 
Cucsa.338110  ---------------------------------------------------------------------------------------------------------------------------------- 
Cucsa.338190  HAEAHQAM-------------------------------------------------------------------------------------------------------------------------- 

                          1700      1710        1720      1730        1740       1750       1760        1770      1780       1790        1800       1810      1820           
                ....|....|....|....|....|....|....|....|....|....|....|....|....|....|....|....|....|....|....|....|....|....|....|....|....|....|
Cucsa.017460  ---------------------------------------------------------------------------------------------------------------------------------- 
Cucsa.017490  ----------------------------------------------------VEKCKNLK----YLLP------------SSIAFLNLKELHIKKCNGMINLFSSTVTKKLVNLSSIKVSYCKGMRCMVE 
Cucsa.088220  FELEGAFIEEILPSNILIPMKKQYNARRSKTSQRSWVLSKLPKLRHLGSECSQKNNDSILQDLTSLSISECGGLSSLVSSSVS-FTNLTFLKLNKCDGLTHLLDPSMATTLVQLKQLRIGECKRMSRIIE 
Cucsa.091880  FELKGMFIEEIFPSNILIP---------SYMVLRKLTLSKLSKLRHLWGECSQKNNDSLLRDLTFLFISKCGGLSSLVSSSVSSFTNLRILEVEKCDGLSHLLSPSVATTLVHLEELRIEECKRMSSVIE 
Cucsa.094560  ---------------------------------------------------------------------------------------------------------------------------------- 
Cucsa.094580  ---------------------------------------------------------------------------------------------------------------------------------- 
Cucsa.094650  ---------------------------------------------------------------------------------------------------------------------------------- 
Cucsa.094660  ---------------------------------------------------------------------------------------------------------------------------------- 
Cucsa.094670  ---------------------------------------------------------------------------------------------------------------------------------- 
Cucsa.102240  ---------------------------------------------------------------------------------------------------------------------------------- 
Cucsa.123410  ---------------------------------------------------------------------------------------------------------------------------------- 
Cucsa.128030  ---------------------------------------------------------------------------------------------------------------------------------- 
Cucsa.128100  ---------------------------------------------------------------------------------------------------------------------------------- 
Cucsa.128110  ---------------------------------------------------------------------------------------------------------------------------------- 
Cucsa.128130  ---------------------------------------------------------------------------------------------------------------------------------- 
Cucsa.128140  ---------------------------------------------------------------------------------------------------------------------------------- 
Cucsa.132370  ---------------------------------------------------------------------------------------------------------------------------------- 
Cucsa.133510  ---------------------------------------------------------------------------------------------------------------------------------- 
Cucsa.163670  ---------------------------------------------------------------------------------------------------------------------------------- 
Cucsa.178360  VDIRNCKKLETIFPNYMLN-RVTNLERLNVTDCSSLVEIFQVKVPVNNGNQVRDIGANHLKELKLLRLPKLKHIWSSDPHNFLRYPSLQLVHTIHCQSLLNLFPVSIAKDLIQLEVLKIQFCGVEEIVAK 
Cucsa.178620  ---------------------------------------------------------------------------------------------------------------------------------- 
Cucsa.189390  ---------------------------------------------------------------------------------------------------------------------------------- 
Cucsa.237070  ---------------------------------------------------------------------------------------------------------------------------------- 
Cucsa.239860  KFFKLKSLELFGCEDGKMISLPMEMNEVLYSIEELTIRGCLQLVDVIGNDYYIQRCANLKKLKLYNLPKLMYVLKNMNQMTATTFSKLVYLQVGGCNGMINLFSPSVAKNLANLNSIEIYDCGEMRTVVA 
Cucsa.248810  ---------------------------------------------------------------------------------------------------------------------------------- 
Cucsa.251930  ---------------------------------------------------------------------------------------------------------------------------------- 
Cucsa.277260  ---------------------------------------------------------------------------------------------------------------------------------- 
Cucsa.318890  ---------------------------------------------------------------------------------------------------------------------------------- 
Cucsa.326910  ---------------------------------------------------------------------------------------------------------------------------------- 
Cucsa.328080  ---------------------------------------------------------------------------------------------------------------------------------- 
Cucsa.337180  ---------------------------------------------------------------------------------------------------------------------------------- 
Cucsa.337190  ---------------------------------------------------------------------------------------------------------------------------------- 
Cucsa.338110  ---------------------------------------------------------------------------------------------------------------------------------- 
Cucsa.338190  ---------------------------------------------------------------------------------------------------------------------------------- 

                         1830      1840        1850       1860        1870      1880        1890       1900       1910       1920       1930       1940       1950           
                ....|....|....|....|....|....|....|....|....|....|....|....|....|....|....|....|....|....|....|....|....|....|....|....|....|....|
Cucsa.017460  ---------------------------------------------------------------------------------------------------------------------------------- 
Cucsa.017490  --VDQAENDE--IITFKKLSTLELDYLPRLDSFYSGKCMLEFPCLESLVIKRCPEMKTFSYGVIIAPRLQTLWMNDKEFGVSSPACGINETIQNFPRRVVCMFNSN------------------------ 
Cucsa.088220  GGSSGEEDGNGEIIVFNNLQLLIITSCSNLTSFYRGRCIIQFPCLKHVSLEKCPKMKSFSFGIVSTSHSKYENVSLKNDDDD-THYRPKESKER------------------------------------ 
Cucsa.091880  GGSS-EEDGNDEIIVFNNLQHLIISSCSNLTSFHCGRCIIQFPCLKQVYINKCTELKVFSLGIVSTPPLKYENIYLKNDDDDDTWHHPKESIEMVVETDMNVIIREYWDDNIDTRISNLFGEE------- 
Cucsa.094560  ---------------------------------------------------------------------------------------------------------------------------------- 
Cucsa.094580  ---------------------------------------------------------------------------------------------------------------------------------- 
Cucsa.094650  ---------------------------------------------------------------------------------------------------------------------------------- 
Cucsa.094660  ---------------------------------------------------------------------------------------------------------------------------------- 
Cucsa.094670  ---------------------------------------------------------------------------------------------------------------------------------- 
Cucsa.102240  ---------------------------------------------------------------------------------------------------------------------------------- 
Cucsa.123410  ---------------------------------------------------------------------------------------------------------------------------------- 
Cucsa.128030  ---------------------------------------------------------------------------------------------------------------------------------- 
Cucsa.128100  ---------------------------------------------------------------------------------------------------------------------------------- 
Cucsa.128110  ---------------------------------------------------------------------------------------------------------------------------------- 
Cucsa.128130  ---------------------------------------------------------------------------------------------------------------------------------- 
Cucsa.128140  ---------------------------------------------------------------------------------------------------------------------------------- 
Cucsa.132370  ---------------------------------------------------------------------------------------------------------------------------------- 
Cucsa.133510  ---------------------------------------------------------------------------------------------------------------------------------- 
Cucsa.163670  ---------------------------------------------------------------------------------------------------------------------------------- 
Cucsa.178360  RGDDGDGDDAASFLLSG-LTSLTLWNLFEFKRFYPGKYTLDCPSLTALDVRHCKSFKLMEGTLENSSSISSAVEKVLHSL-------------------------------------------------- 
Cucsa.178620  ---------------------------------------------------------------------------------------------------------------------------------- 
Cucsa.189390  ---------------------------------------------------------------------------------------------------------------------------------- 
Cucsa.237070  ---------------------------------------------------------------------------------------------------------------------------------- 
Cucsa.239860  --AKAEEEEENVEIVFSKLTGMEFHNLAGLECFYPGKCTLEFPLLDTLRISKCDDMKIFSYGITNTPTLKNIEIGEHNSLPFTSICGVHTILSEKEGIINLMA--------------------------- 
Cucsa.248810  ---------------------------------------------------------------------------------------------------------------------------------- 
Cucsa.251930  ---------------------------------------------------------------------------------------------------------------------------------- 
Cucsa.277260  ---------------------------------------------------------------------------------------------------------------------------------- 
Cucsa.318890  ---------------------------------------------------------------------------------------------------------------------------------- 
Cucsa.326910  ---------------------------------------------------------------------------------------------------------------------------------- 
Cucsa.328080  ---------------------------------------------------------------------------------------------------------------------------------- 
Cucsa.337180  ---------------------------------------------------------------------------------------------------------------------------------- 
Cucsa.337190  ---------------------------------------------------------------------------------------------------------------------------------- 
Cucsa.338110  ---------------------------------------------------------------------------------------------------------------------------------- 
Cucsa.338190  ---------------------------------------------------------------------------------------------------------------------------------- 
