# Supplementary material for: Genome-wide analysis of NBS-encoding disease resistance genes in Cucumis sativus and phylogenetic study of NBS-encoding genes in Cucurbitaceae crops
Source: BMC Genomics. 2013 Feb 19;14:109. doi: 10.1186/1471-2164-14-109 (PMC3599390; doi:10.1186/1471-2164-14-109)
Supplement: Additional file 13 — Degenerate primers used for PCR amplification from the four major Cucurbitaceae crops (melon, bottle gourd, luffa and watermelon). [file 1471-2164-14-109-S13.doc]

**Additional file 13** Degenerate primers used for PCR amplification from four major *Cucurbitaceae* crops (melon, bottle gourd, luffa and watermelon)

| Primer | Conserved motif | Primer sequence | Reference |
| --- | --- | --- | --- |
| F1 | P-loop | TGSSRGGHWYRGGBAAAACTAC | Zhang et al. (2008) |
| R1 | GLPL | HRCWARAGGVARCCCTYBACA |
| F2 | P-loop | GGDGTDGGNAARACWAC | Deng et al. (2000) |
| R2 | GLPL | IARIGCIARIGGIARNCC |
| F3 | P-loop | GGWATGGGWGGWRTHGGWAARACHAC | Lee et al. (2003) |
| R3 | GLPL | ARNWYYTTVARDGCVARWGGVARWCC |

The sequences are coded according to the International Units of Biochemistry: N = A or C or G or T; R = A or G; S = C or G; Y = C or T; W= A/T; Y= T/C; B= G/C/T; H=A/T/C; V = A or C or G; I = Inosine

**Reference**

Lee SY, Seo JS, Rodriguez-Lanetty M, Lee DH: Comparative analysis of superfamilies of NBS-encoding disease resistance gene analogs in cultivated and wild apple species, Mol. Genet. Genome (2003) 269: 101–108.

Deng Z, Huang S, Ling P, Chen C, Yu C, Weber CA, Moore GA, Gmitter FG: Cloning and characterization of NBS-LRR class resistance-gene candidate sequences in citrus, Thero Appl Genet (2000) 101: 814-822.

Zhang LY, Chen RG, Zhang JH: Cloning and analysis of resistance gene analogs from pepper (*Capsicum annuum* L.), Agr Sci China (in Chinese) (2008) 41(1):169-175.
